# Transition metal-free direct dehydrogenative arylation of activated C(sp^3^)–H bonds: synthetic ambit and DFT reactivity predictions†
†Electronic supplementary information (ESI) available: Complete experimental and computational results, procedures and characterization including ^1^H and ^13^C spectra and X-ray crystallographic data. CCDC 1859486 and 1859487. For ESI and crystallographic data in CIF or other electronic format see DOI: 10.1039/c8sc02758g


**DOI:** 10.1039/c8sc02758g

**Published:** 2018-08-27

**Authors:** Kaitlyn Lovato, Lirong Guo, Qing-Long Xu, Fengting Liu, Muhammed Yousufuddin, Daniel H. Ess, László Kürti, Hongyin Gao

**Affiliations:** a Ministry of Education Key Laboratory of Colloid and Interface Chemistry , School of Chemistry and Chemical Engineering , Shandong University , Ji'nan 250100 , China . Email: hygao@sdu.edu.cn; b Department of Chemistry , Rice University , BioScience Research Collaborative , Houston , Texas 77005 , USA . Email: kurti.laszlo@rice.edu; c Jiangsu Key Laboratory of Drug Discovery for Metabolic Disease , State Key Laboratory of Natural Medicines , China Pharmaceutical University , 24 Tongjia Xiang , Nanjing 210009 , China; d Life and Health Sciences Department , The University of North Texas at Dallas , Dallas , Texas 76016 , USA; e Department of Chemistry and Biochemistry , Brigham Young University , Provo , Utah 84602 , USA . Email: dhe@chem.byu.edu

## Abstract

A transition metal-free dehydrogenative method for the direct mono-arylation of a wide range of activated C(sp^3^)–H bonds has been developed. This operationally simple and environmentally friendly aerobic arylation uses *tert*-BuOK as the base and nitroarenes as electrophiles to prepare up to gram quantities of structurally diverse sets (>60 examples) of α-arylated esters, amides, nitriles, sulfones and triaryl methanes. DFT calculations provided a predictive model, which states that substrates containing a C(sp^3^)–H bond with a sufficiently low p*K*_a_ value should readily undergo arylation. The DFT prediction was confirmed through experimental testing of nearly a dozen substrates containing activated C(sp^3^)–H bonds. This arylation method was also used in a one-pot protocol to synthesize over twenty compounds containing all-carbon quaternary centers.

## Abstract 2

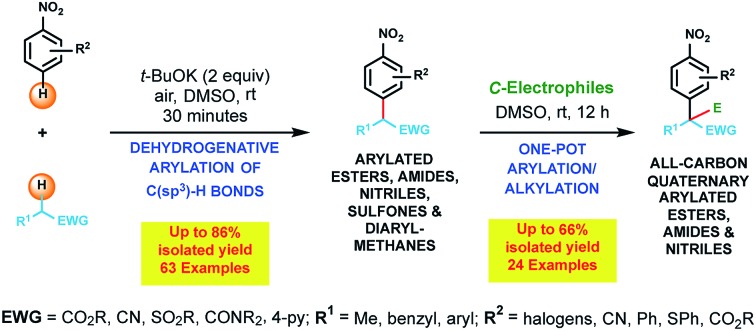
A direct and general mono-arylation of activated C(sp^3^)–H bonds with nitroarenes under transition metal-free conditions has been developed.

## Introduction

α-Arylated carbonyl derivatives and triaryl methanes are versatile structural motifs present in a large number of natural products and active pharmaceutical ingredients.^1^ These motifs have also been identified as important intermediates for the preparation of substituted heterocycles.^2^ Given their importance, it is not surprising that several synthetic methods have been developed to access these valuable building blocks.

**Scheme 1:**
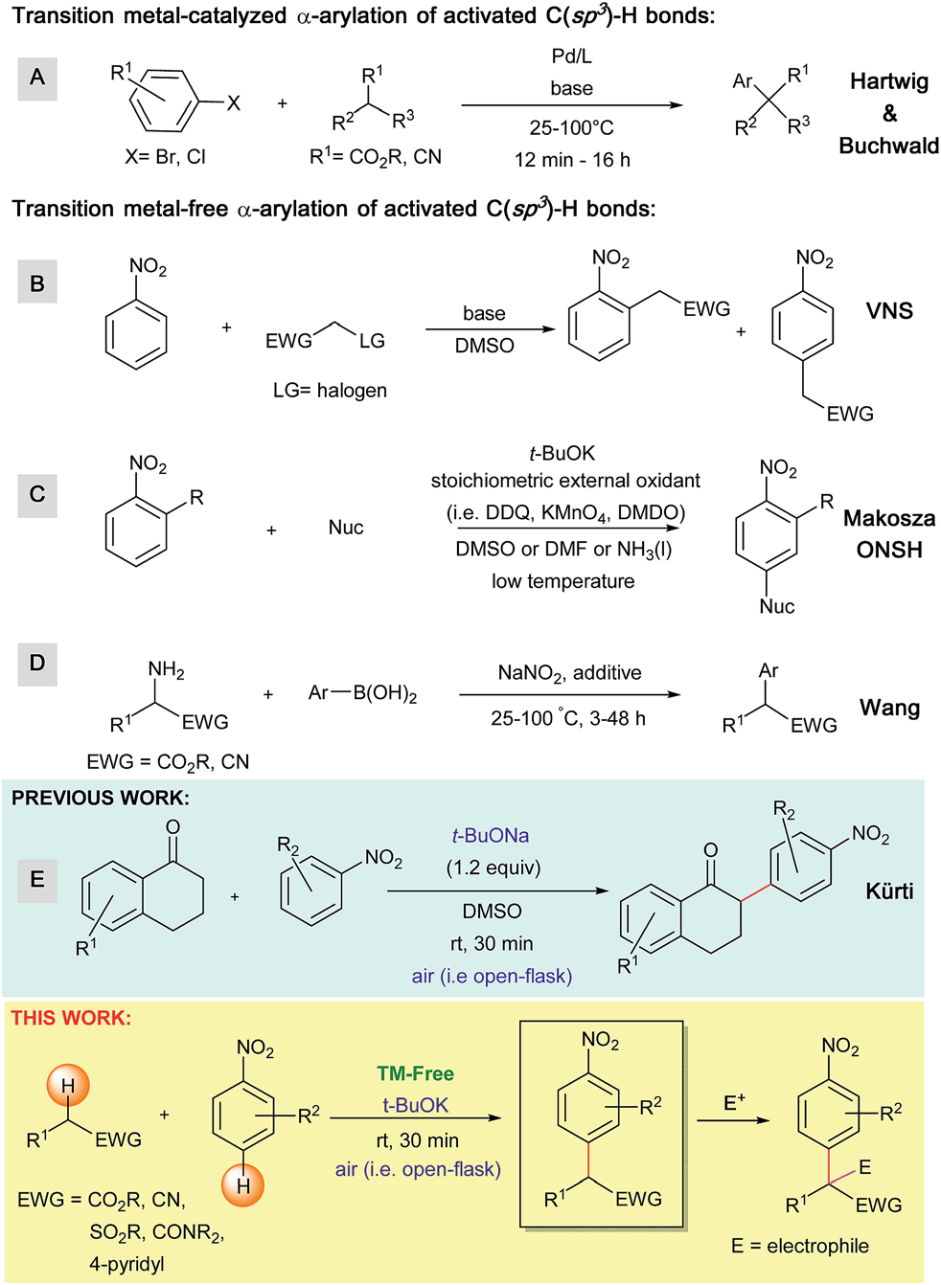
Transition metal-catalyzed and transition metal-free arylations of activated C(sp^3^)–H bonds.

Over the past two decades, the transition metal-catalyzed α-arylation of activated C(sp^3^)–H bonds has become a robust strategy for the construction of sp^3^–sp^2^ carbon–carbon bonds (Scheme 1).^3^ One prominent example is the palladium-catalyzed α-arylation of esters and nitriles first reported by Hartwig and Buchwald (Scheme 1A).^4^,^5^ Hartwig improved these α-arylation reactions by utilizing zinc enolates,^6^ α-silyl nitriles, and zinc cyanoalkyls^7^ as enolate precursors, thus making this transformation feasible under less basic conditions. Similar transition metal-catalyzed α-arylation conditions have also been reported by Hartwig *et al.* for amide substrates.^8^ In addition to these methods, several other transition metal-catalyzed α-arylation conditions have emerged over the past decade.^9^,^10^ Although transition metal-catalyzed α-arylations are widely used, they still suffer from a number of drawbacks, including; (1) the need for expensive catalysts, ligands and additives, (2) the use of high reaction temperatures and (3) sub-optimal functional group tolerance (*e.g.*, halogens). Moreover, the reactions generate toxic heavy metal waste and also face a limited availability of pre-functionalized coupling partners. Because of these drawbacks, the development of practical transition metal-free approaches is highly desirable.

To date, several transition metal-free methods have been developed to obtain α-arylated carbonyls and their derivatives, including: (a) the arylation of activated C(sp^3^)–H bonds *via* vicarious nucleophilic substitution (VNS, Scheme 1B),^11^ (b) oxidative nucleophilic substitution of hydrogen (ONSH, Scheme 1C),^12^ (c) a recently developed deaminative arylation of α-aminoesters and α-aminoacetonitriles (Scheme 1D)^13^ and (d) the coupling of arylaectates with nitroarenes to produce diaryl ketones.^14^ However, these methods still possess various drawbacks. From a practical standpoint, VNS requires leaving groups (*e.g.* halogens) installed in the α-position of the carbanion precursors. Additionally, ONSH requires stoichiometric amounts of harsh oxidizing agents (*e.g.* KMnO_4_, DMDO and DDQ) as well as cryogenic conditions (*e.g.* below –40 °C, in liquid ammonia).^15^ These conditions result in sub-optimal functional group tolerance and limited structural diversity of the obtained products.

**Scheme 2:**
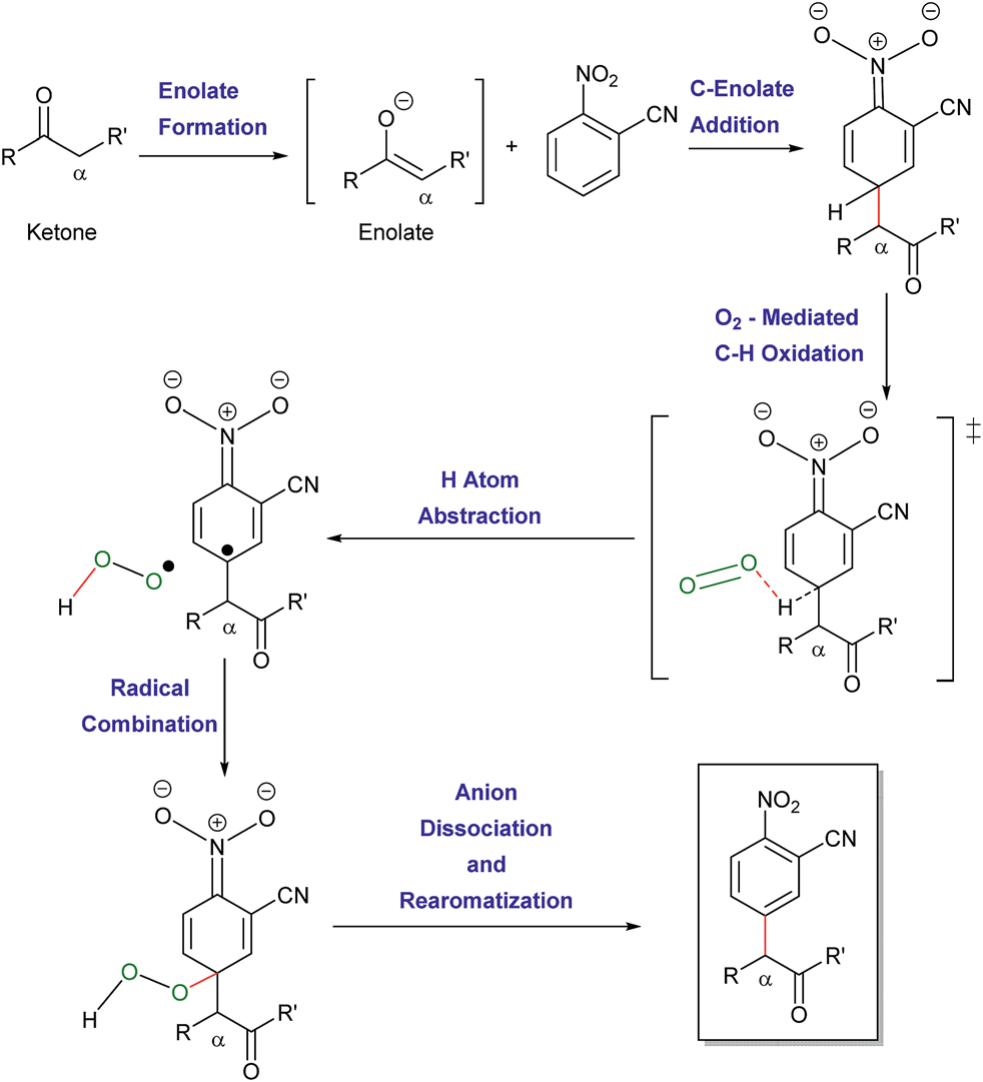
Proposed mechanism for the arylation of ketones with nitroarenes under basic conditions supported by DFT calculations.

The work presented herein builds on the transition metal-free, direct and general mono-α-arylation of ketones developed by our group in 2013 (Scheme 1E),^16^ which doesn't require substrates with a built-in leaving group, low temperatures or harsh external oxidants. For this transformation, density functional theory (DFT) calculations proposed and experiments supported a novel mechanistic pathway (Scheme 2). In this pathway the enolate first adds to the nitroarene. Subsequent O_2_-induced hydrogen atom abstraction leads to oxidation *via* air, which is then followed by radical combination and rearomatization. Given the fact that O_2_ is a mild, abundant and environmentally friendly oxidant, we became intrigued by the possibility that this mechanism could be extended to a variety of other activated C(sp^3^)–H bonds.

In this manuscript, we describe extensive synthetic efforts and a DFT-based reactivity model for this transition metal-free direct arylation of activated C(sp^3^)–H bonds. Through this work, we aim to provide a general and operationally simple arylation method and also a description of the reactivity trends in transformations of this type.

## Results and discussion

We began our synthetic arylation studies using methyl 2-phenylacetate (**1**) and nitrobenzene (**2**) as substrates (Table 1). Screening a series of bases in DMSO indicated that *t*-BuOK was most effective at room temperature (Table 1, entries 1–5).

**Table 1:** Base, solvent and nitroarene coupling partner optimization studies for the α-arylation of methyl 2-phenylacetate with nitrobenzene

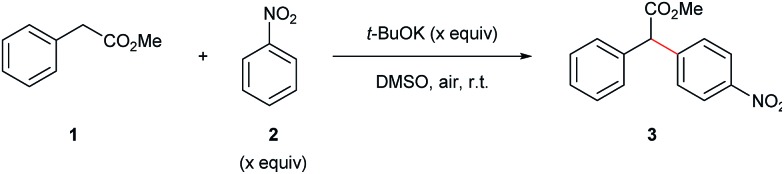					
Entry^*a*^	**2** equiv.	Base (equiv.)	Solvent	Time (h)	Yield^*b*^ (%)
1	2.0	*t*-BuOK (1.2)	DMSO	0.5	30
2	2.0	*t*-BuONa (1.2)	DMSO	0.5	25
3	2.0	*t*-BuOLi (1.2)	DMSO	0.5	16
4	2.0	NaOH (1.2)	DMSO	12	<5
5	2.0	NaH (1.2)	DMSO	12	<5
6	2.0	*t*-BuOK (1.5)	DMSO	0.5	40
**7**	**2.0**	***t*-BuOK (2.0)**	**DMSO**	**0.5**	**53**
8	2.0	*t*-BuOK (2.5)	DMSO	0.5	54
9	2.0	*t*-BuOK (2.0)	DMF	0.5	28
10	2.0	*t*-BuOK (2.0)	DMA	0.5	<5
11	2.0	*t*-BuOK (2.0)	NMP	0.5	<5
12	2.0	*t*-BuOK (2.0)	Dioxane	12	N.R.
13	2.0	*t*-BuOK (2.0)	CF_3_CH_2_OH	12	N.R.
14	1.0	*t*-BuOK (2.0)	DMSO	0.5	26^*c*^
15	1.2	*t*-BuOK (2.0)	DMSO	0.5	24^*c*^
16	1.5	*t*-BuOK (2.0)	DMSO	0.5	25^*c*^
17	3.0	*t*-BuOK (2.0)	DMSO	0.5	51

^*a*^Reaction conditions: **1** (1.0 mmol), **2** (1.0–3.0 equiv.), base (1.2–2.5 equiv.), solvent (5 mL), open flask, room temperature.

^*b*^Isolated yield after column chromatography.

^*c*^Yield calculated from crude NMR spectra using dibromomethane as an internal standard.

Screening for the optimal amounts of base (Table 1, entries 6–8) and nitrobenzene coupling partner (Table 1, entries 14–17) revealed that 2.0 equivalents of *t*-BuOK and 2.0 equivalents of nitrobenzene was sufficient to afford a synthetically useful isolated yield of the α-arylated product. A solvent screen (Table 1, entries 9–13) showed DMSO to be the best solvent for this transformation.

**Scheme 3:**
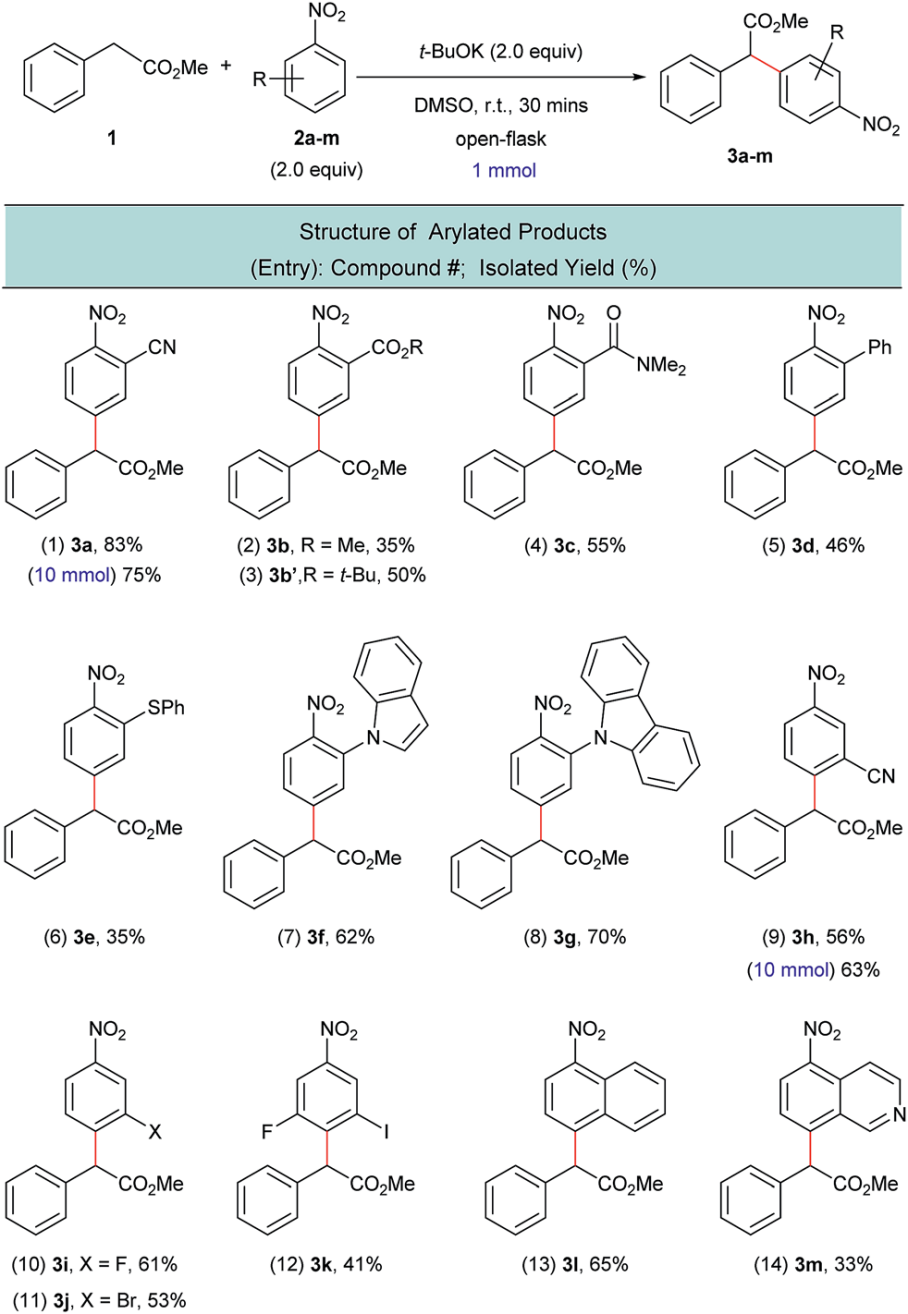
α-Arylation of **1** with various nitroarenes. ^a^Reaction conditions: **1** (1.0 mmol), **2a–m** (2.0 equiv.), *t*-BuOK (2.0 equiv.), DMSO (5 mL), open flask, room temperature, 30 minutes.

With the optimized reaction conditions in hand, we next investigated the scope of nitroarene coupling partners (**2a–m**) capable of undergoing this mono α-arylation (Scheme 3). It was found that nitroarenes containing an electron-withdrawing group in the *ortho* position successfully reacted with methyl phenylacetate (**1**) to give the desired arylated products **3a–3e** in moderate to good yields (Scheme 3, entries 1–6). Nitroarenes with heteroatoms in the *ortho* position also acted as efficient arylating agents, leading to the corresponding products **3f** and **3g** in 62% and 70% yields, respectively (Scheme 3, entries 7 and 8).

*Meta*-substituted nitroarenes were also suitable coupling partners for this transformation and afforded the *para* substituted products **3h–3k** in moderate to good yields and with complete regioselectivity (Scheme 3, entries 9–12). It is worth noting that the bromo-substituted nitroarene (Scheme 3, entry 11) was an effective arylating agent, which highlights the complementarity of this protocol to traditional transition metal-catalyzed cross-coupling reactions. Additionally, the halogenated products can be further functionalized using a myriad of available C–C and C–X bond-forming transformations.^17^

The scope of the nitroarene coupling partners could also be extended to fused systems, which were exclusively arylated *para* to the nitro group (Scheme 3, entries 13 and 14). Gram-scale experiments have been carried out with **2a** and **2h**, which afforded the arylated products **3a** and **3h** in good isolated yields (Scheme 3, entries 1 and 9).

Greater than a dozen successful electrophilic coupling partners were identified, however several substrate classes were unable to produce the desired arylated products. For example, it was observed that the S_N_Ar pathway, not the dehydrogenative arylation pathway, is the major pathway when the nitroarene coupling partner possesses a halogen atom in the *ortho*- or *para*-position (see ESI S25†). Interestingly, it was previously found that ketone enolates do not undergo S_N_Ar with halogenated nitroarenes.^16^ When there is a *para*-non-halogen atom in the nitroarene, dehydrogenative arylation at the *ortho*-position occurs in some cases but because other S_N_Ar-type reaction pathways are also possible these reactions have low yields (see ESI S26†). In addition, other electron deficient arenes (*i.e.* not nitroarenes) were tested as substrates. In these reactions, the arylated products were not observed and the electrophilic substrates were recovered (see ESI S27†).

**Scheme 4:**
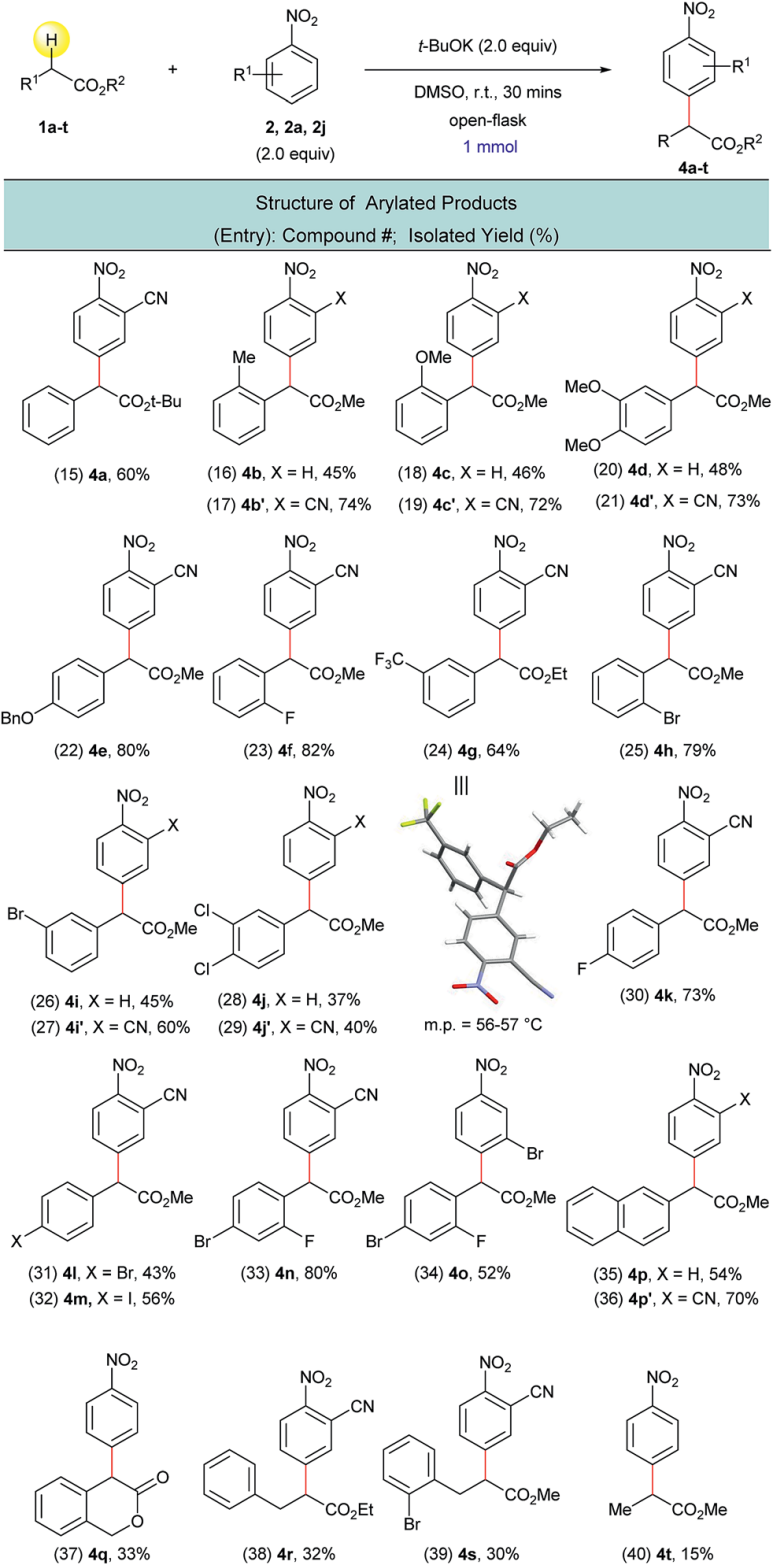
α-arylation of various ester substrates with nitroarenes. ^a^Reaction conditions: **1a–t** (1.0 mmol), **2**, **2a**, **2j** (2.0 equiv.), *t*-BuOK (2.0 equiv.), DMSO (5 mL), open flask, room temperature, 30 minutes.

Next, the arylation of twenty ring-substituted 2-phenylacetates was evaluated (Scheme 4) with three nitroarenes. Both electron-donating and electron-withdrawing substituents were tolerated on the aryl rings. The α-arylated products were obtained with high chemo- and regioselectivity (Scheme 4, entries 15–34). For arylated product **4g** the structure was confirmed using single-crystal X-ray crystallography (Scheme 4, entry 24). More complex coupling partners such as 2-(naphthalen-2-yl)acetate (**1p**), isochroman-3-one (**1q**), 3-phenylpropanoates (**1r**, **1s**) and methyl propionate (**1t**) were also able to undergo the present transformation and produced the corresponding α-arylated products **4p–4t** (Scheme 4, entries 35–40) in fair to moderate yields. In general, the reactions of 2-cyanonitrobenzene (**2a**) with 2-phenylacetates (**1a–t**) gave higher yields than the reactions of unsubstituted nitrobenzene (**2**) with the same 2-phenylacetates. This is presumably due to the stronger electrophilicity of 2-cyanonitrobenzene (Scheme 4, entries 16 *vs.* 17, 18 *vs.* 19, 20 *vs.* 21, 26 *vs.* 27, 28 *vs.* 29, and 35 *vs.* 36).

**Scheme 5:**
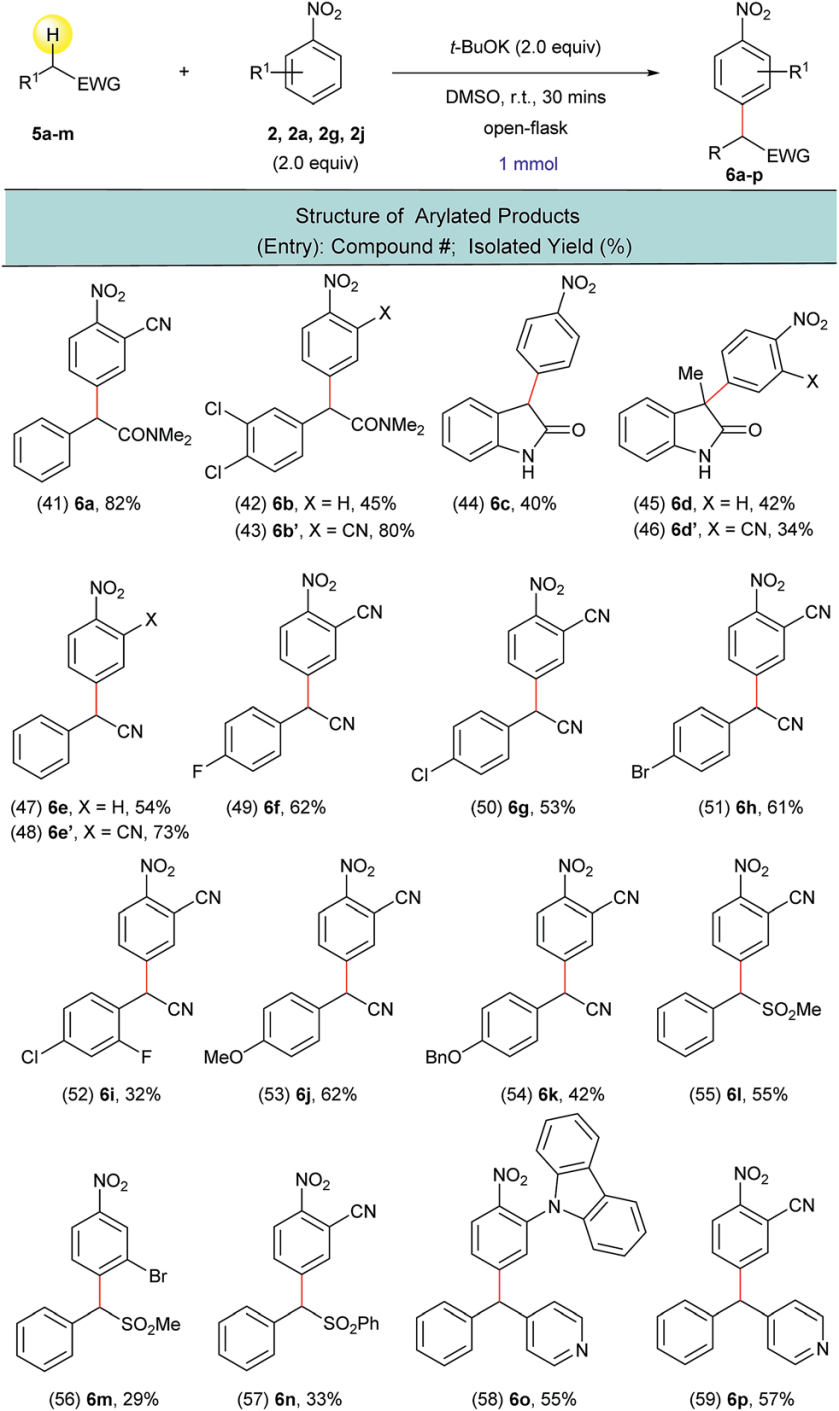
Arylation of various activated C(sp^3^)–H bonds with nitroarenes. ^a^Reaction conditions: **5a–m** (1.0 mmol), **2**, **2a**, **2g**, **2j** (2.0 equiv.), *t*-BuOK (2.0 equiv.), DMSO (5 mL), open flask, room temperature, 30 minutes.

In order to extend this transition metal-free direct dehydrogenative arylation to other types of substrates, we examined four new classes of activated C(sp^3^)–H bonds in amides, nitriles, sulfones and diaryl methanes. We were pleased to find that not only 2-arylacetamides but also oxindoles were suitable substrates for this transformation (Scheme 5, entries 41–46). It was determined that this synthetic protocol could also be applied to the α-arylation of 2-aryl acetonitriles (Scheme 5, entries 47–54). The presence of halogenated arene rings in these substrates (Scheme 5, entries 49–52) further implies that this arylation method is complementary to the currently used transition metal-catalyzed methods for the arylation of nitriles. The dehydrogenative arylation of sulfones, benzyl sulfones and benzyl pyridine was also possible and gave the desired diaryl sulfones and triarylmethane products in synthetically useful isolated yields (Scheme 5, entries 55–59).

**Scheme 6:**
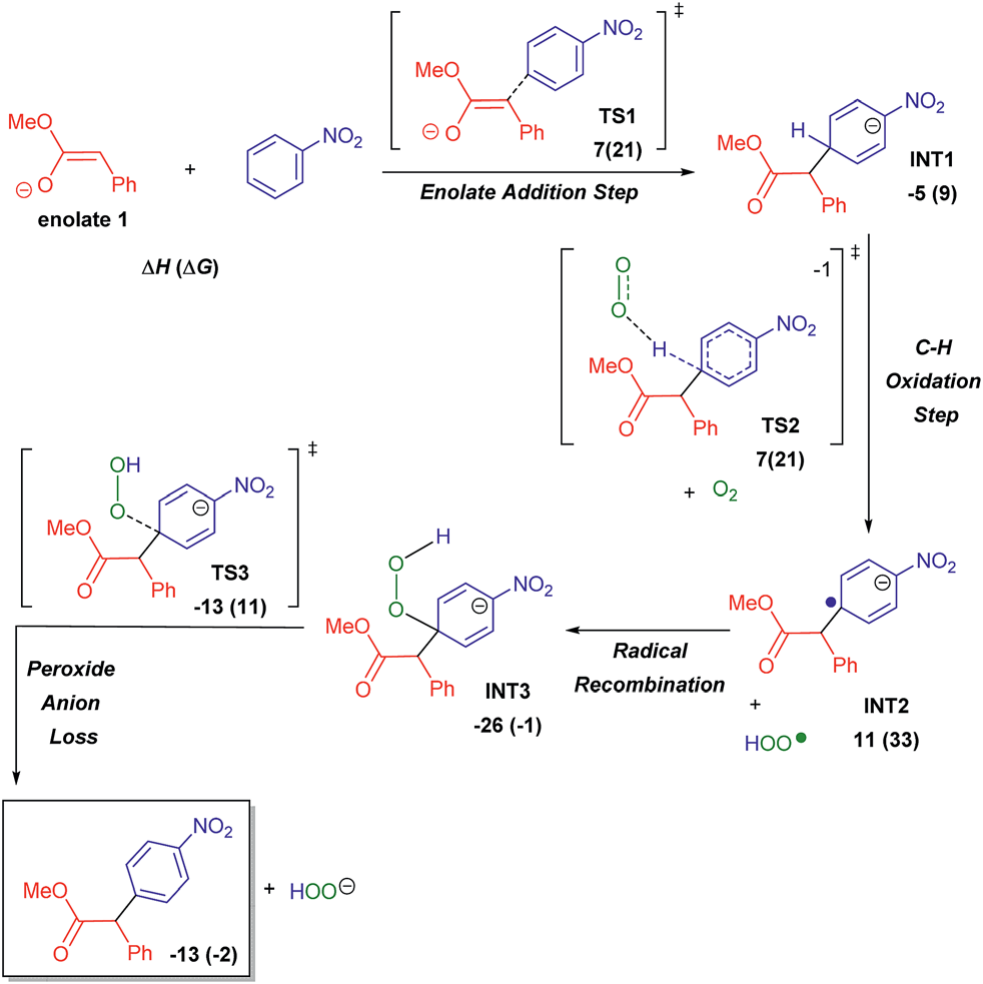
Ester arylation pathway examined by DFT calculations to compare reactivity with ketones. Enthalpies include SMD estimate of (Δ*G*_solv_).

Simultaneous to synthetic studies, we undertook DFT calculations to compare the reactivity of esters, amides and nitriles to the previously reported ketones.^16^ We also aimed to create a simple predictive model for the arylation of unexplored compound classes. We began by calculating the arylation reaction pathway outlined by our previous DFT calculations.^16^ This involved calculating the C–H oxidation reaction between methoxy-phenylethenolate (**enolate 1**) and nitrobenzene using M06-2X/def2-TZVPD//M06-2X/631+G** level of theory with the continuum SMD solvent model for DMSO (see Scheme 6 energy surface, the Z-enolate conformation is favored throughout the pathway).^18^**Enolate 1** and nitrobenzene can initially form a slightly exothermic, but endergonic, charge-transfer complex due to the ability of *t*-BuOK to act as an electron-transfer agent^19^ (see ESI S66†). However, we believe that an electron-transfer (ET)/radical recombination pathway for C–C bond-formation is not as viable because the enthalpy for enolate ET to nitrobenzene would require 19 kcal mol^–1^.

In the proposed C–H oxidation pathway, **enolate 1** adds to nitrobenzene in the *para*-position to give **INT1** (Scheme 6). Then, O_2_-induced C–H oxidation of **INT1** occurs through **TS2** (the Δ*H*^‡^ for **TS2** is 11 kcal mol^–1^). This oxidation leads to an aryl anion radical (**INT2**) as well as an HOO˙ radical that combine to form the oxidized intermediate **INT3**. The loss of the hydrogen peroxide anion by **TS3** gives the final arylation product.

Since our initial discovery and proposal of this O_2_-mediated oxidation pathway, it was proposed by Kumar that DMSO serves as the major oxidant in a similar cross coupling of aryl acetamides with nitroarenes.^20^ This proposal was made based on the GC and NMR detection of dimethyl sulfide (DMS) in a crude reaction mixture. This proposal is interesting because the two-electron reduction potential for DMSO has been estimated to be ∼0.2 V (ref. 21) while the two-electron reduction potential for O_2_ is 0.7 V. Therefore, we examined the thermodynamics for the oxidation of **INT1** by DMSO. Hydride transfer from **INT1** to DMSO to give dimethyl sulfide, hydroxide and the arylated product is slightly endothermic by ∼2 kcal mol^–1^. Unfortunately, extensive searching of the potential energy surface did not locate a hydride transfer or hydrogen atom transfer transition state between **INT1** and DMSO. Because our calculations could not locate a kinetic barrier for **INT1** oxidation by DMSO, we also decided to experimentally examine this oxidation possibility. We conducted a NMR study of the reaction of methyl 2-phenylacetate (**1**) with 2-cyanonitrobenzene (**2a**) under our standard conditions (see ESI S43†). While we did observe some dimethyl sulfide in the crude reaction mixture after 30 minutes, it was in a small 1 : 7 ratio of DMS: arylated product (**3a**). This result does not support DMSO acting as the major (*i.e.* stoichiometric) oxidant. Additionally, experiments without exposure to air result in a massively diminished yield of arylated product. It has also been proposed by Makosza that **INT1** could be deprotonated to generate a dianion prior to oxidation.12*b* However, our calculations suggest that proton transfer to either *tert*-butoxide (Δ*H* > 35 kcal mol^–1^) or DMSO is highly endothermic. In addition to the C–H oxidation pathway, we also examined several off-pathway reactions including addition to the *ortho*-position. After the formation of **enolate 1**, the enthalpy barrier (Δ*H*^‡^) for C–C bond-formation at the *para*-position is 7 kcal mol^–1^ by **TS1** relative to reactants to give **INT1**. For ketone arylation, we previously showed that there is a similar barrier for C–C formation at the *ortho*-position to the nitro group.^16^ While an *ortho* addition intermediate is likely in equilibrium with **INT1**, we have found that there is generally a higher barrier for its subsequent C–H oxidation.^16^ Therefore, the *ortho*-enolate addition is reversible and should be considered a minor, non-productive, off-reaction-pathway intermediate and not a key intermediate.^22^ Because the enolate–nitrobenzene addition intermediates prior to C–H oxidation are endergonic it is also unlikely that the formation of this off-pathway *ortho*-addition (and ipso-addition) intermediate greatly impacts reaction rates. Similarly, we also located addition intermediates between *tert*-butoxide and nitrobenzene, which are also off-pathway and non-productive.

**Scheme 7:**
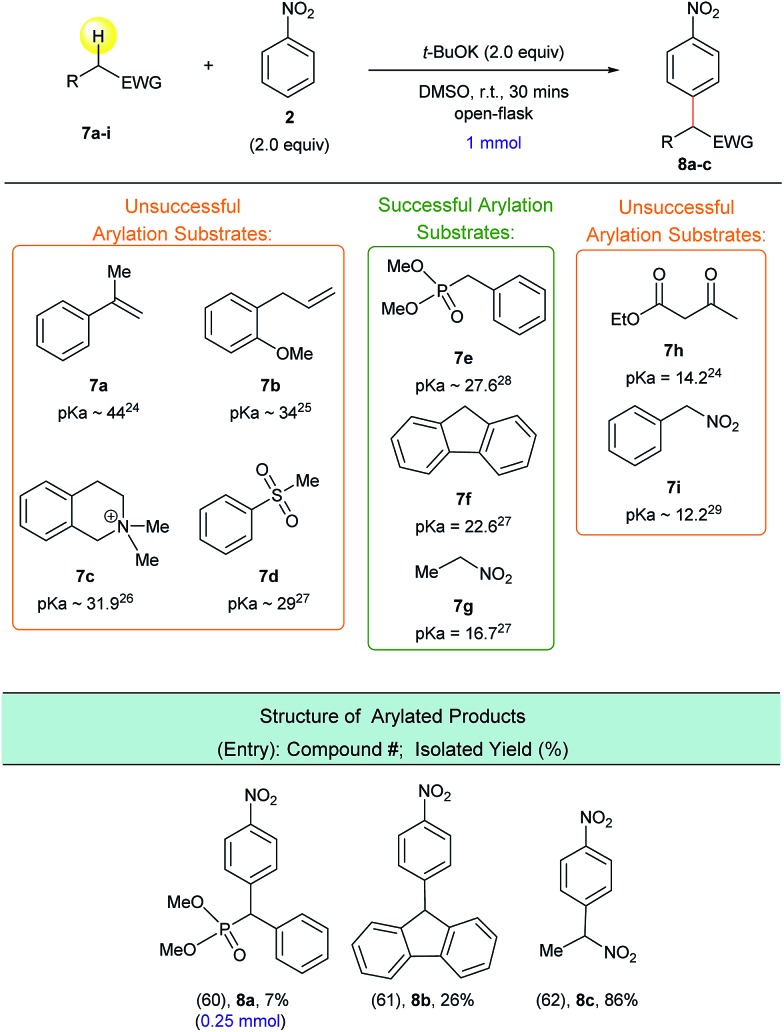
Predicting the success of the arylation reaction based on the p*K*_a_ value of the substrate's activated C(sp^3^)–H bond. ^a^Reaction conditions: **7a–i** (1.0 mmol), **2** (2.0 equiv.), *t*-BuOK (2.0 equiv.), DMSO (5 mL), open flask, room temperature, 30 minutes. ^b^Isolated yield after column chromatography.

In order to understand the reactivity trends for these types of reactions, we compared the barriers of the arylations of other activated C(sp^3^)–H bond substrates. Overall, the barriers for ester arylations are either equal to or lower than the barriers for the ketone arylations we previously reported.^16^ We also calculated enolate addition/O_2_-mediated C–H oxidation for the aminophenylethenolate (Scheme 5, **5a**) and cyano(phenyl)methanide (Scheme 5, **5d**) anions. These enolates have enthalpy barriers within ∼2–3 kcal mol^–1^ of the ester and ketone arylations. For example, the Δ*H*^‡^ for C–C bond-formation between aminophenylethenolate (Scheme 5, **5a**) and nitrobenzene (**2**) is 5 kcal mol^–1^. We then examined the barriers for arylation of (methylsulfonyl)benzene (Scheme 7, **7d**) and (nitromethyl)benzene (Scheme 7, **7i**), both of which did not undergo arylation with nitrobenzene. In these cases, enolate addition has Δ*H*^‡^ = 12 kcal mol^–1^ for C–C bond-formation. While this is ∼5 kcal mol^–1^ higher than ester enolate addition, the barriers are low enough to suggest that if the enolate is formed, arylation would likely proceed. This suggested to us that unsuccessful substrates might result from thermodynamic inaccessibility of the enolate rather than failure of the addition and/or oxidation reaction steps. This is consistent with the report by Xiao that showed a kinetic isotope effect value of ∼6 for the methyl C–H bonds for arylation of 2-methylazaarenes.^23^ Therefore, we estimated the p*K*_a_ values of reagents that did not undergo arylation. For example, we estimated the p*K*_a_ of (methylsulfonyl)benzene (Scheme 7, **7d**) relative to *N*,*N*-dimethyl-2-phenylacetamide (Scheme 5, **5a**, p*K*_a_ value of ∼26) and determined an experimental p*K*_a_ value of 30. Computationally we found that, in general, substrates that underwent successful arylation had p*K*_a_ values less than 27.

Based on this possible p*K*_a_-predictor, we experimentally examined a wide variety of substrates with activated C(sp^3^)–H bonds (Scheme 7). Indeed, experiments determined that substrates which have p*K*_a_ values greater than 27 (ref. 24, 25, 26 and 27) (*i.e.***7a–7d**) do not produce the corresponding α-arylated products, while substrates with p*K*_a_ values less than 27 (ref. 27) (*i.e.***7f** and **7g**), were successfully arylated to afford products **8b** and **8c**. We had to establish a new p*K*_a_ threshold value of 28 due to the fact that substrate **7e**, which has a C(sp^3^)–H p*K*_a_ value of 27.6 (ref. 28) produced a minimal yield of arylated product **8a**. It is important to note that the reaction conditions were not optimized for compounds **8a–8c**, but it is clear that with proper solvent and temperature screens, these yields can presumably be improved.

Experimentally, we examined the lower p*K*_a_ limit for viable arylation substrates, which allowed us to determine an optimal C(sp^3^)–H bond p*K*_a_ range for suitable arylation substrates. The testing of substrates **7h** and **7i**, which had C(sp^3^)–H p*K*_a_ values of 14.2 and 12.2, respectively,^24^,^29^ allowed us to determine the lower limit of the optimal C(sp^3^)–H bond p*K*_a_ range. Based on our experimental results, we determined that an activated C(sp^3^)–H bond with a p*K*_a_ value less than 28 and greater than 16 should successfully participate in this arylation process. Presumably, substrates with p*K*_a_ values lower than 16 form a highly stabilized enolate – apparently this stabilization impedes the addition to the nitroarene electrophile and thus limits arylated product formation.

This optimal p*K*_a_ range establishes a guide for the potential substrate scope of this reaction. This guide can be used to identify substrate classes that are likely to produce the corresponding arylated products without committing valuable resources and time on substrates that are unlikely to undergo this transformation.

**Scheme 8:**
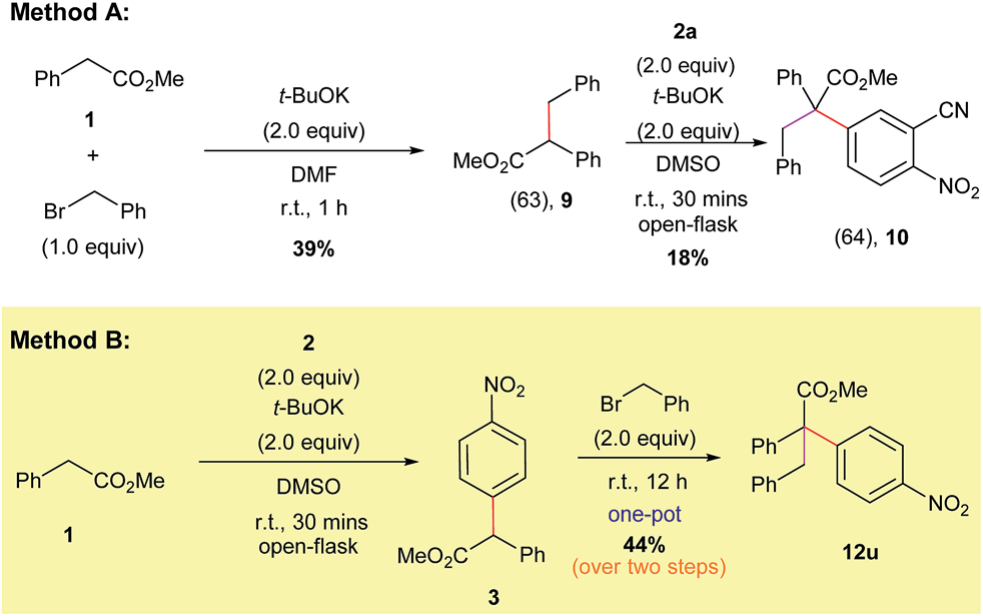
Different routes for the synthesis of compounds featuring all carbon quaternary centers.

Naturally, we also explored the preparation of compounds containing all-carbon quaternary centers using our direct α-arylation protocol (Scheme 8). Initially we tried to form the highly substituted products *via* a two-step process. Accordingly, benzyl bromide was added to methyl 2-phenylacetate (**1**) to form the alkylated derivative (**9**).^30^ Compound **9** was purified *via* column chromatography and then subjected to our α-arylation conditions, which furnished the desired product **10** in a moderate yield (Scheme 8, Method A).

The low yield and the need for multiple purification steps compelled us to pursue the preparation of the all carbon quaternary center-containing products in a one-pot fashion and without the use of a pre-functionalized starting material (Scheme 8, Method B). We found that subjecting methyl 2-phenylacetate (**1**) to our optimized α-arylation conditions, followed by the addition of benzyl bromide to the same pot, was able to produce the highly-substituted product **12u** in a significantly higher yield compared to Method A.

**Scheme 9:**
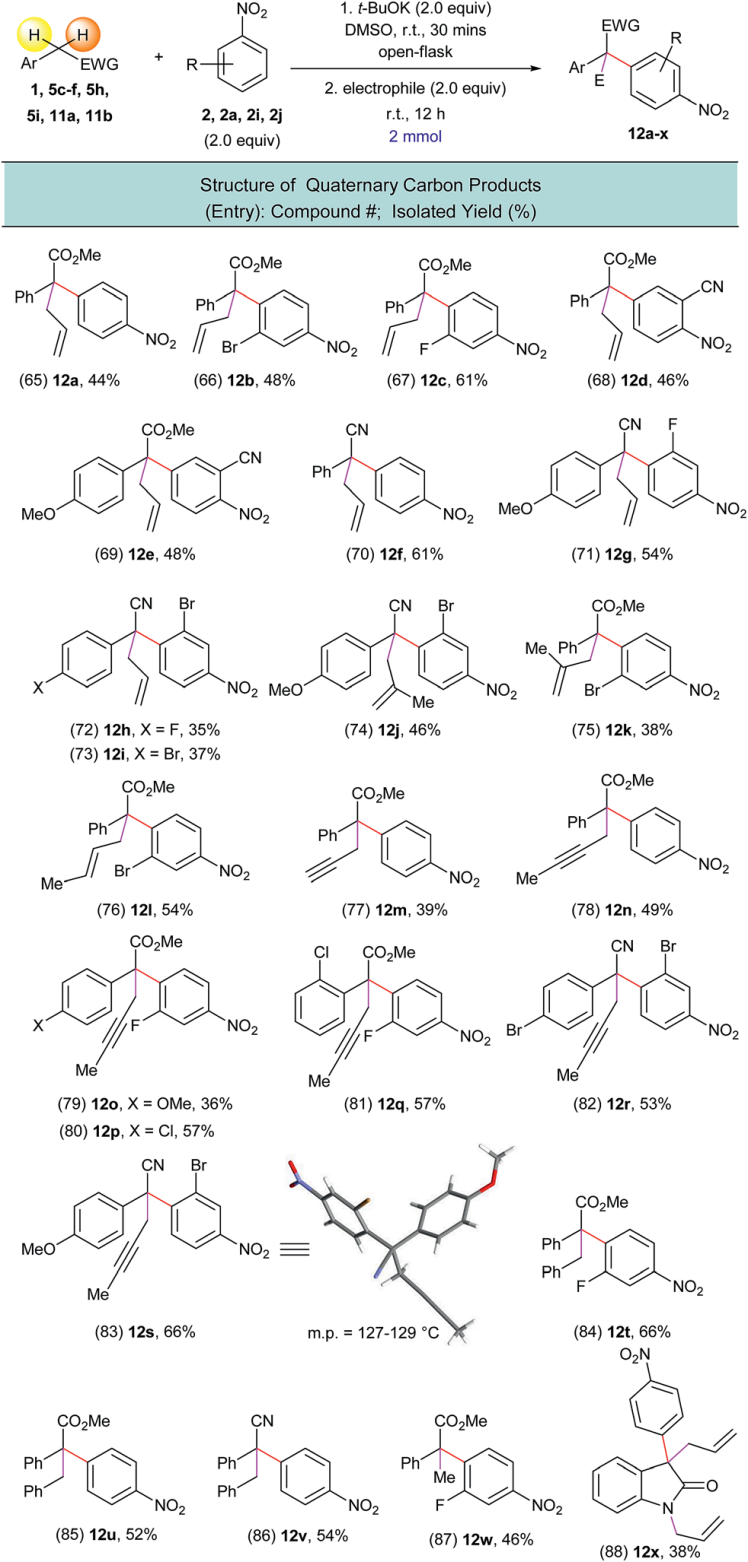
Preparation of all-carbon quaternary center containing compounds using the one-pot α-arylation/alkylation process. ^a^Reaction conditions: (1) **1**, **5c–f**, **5h**, **5i**, **11a**, **11b** (2.0 mmol), **2**, **2a**, **2i**, **2j** (2.0 equiv.), *t*-BuOK (2.0 equiv.), DMSO (10 mL), open flask, room temperature, 30 minutes; (2) electrophiles (2.0 equiv.), room temperature, 12 h.

Encouraged by this result we tested a series of electrophiles, including allylbromide, propargyl bromide, benzyl bromide and iodomethane. It was found that these electrophiles could be efficiently attached to the α-position of the arylated substrates *via* this one-pot process (Scheme 9, entries 65–88).

**Scheme 10:**
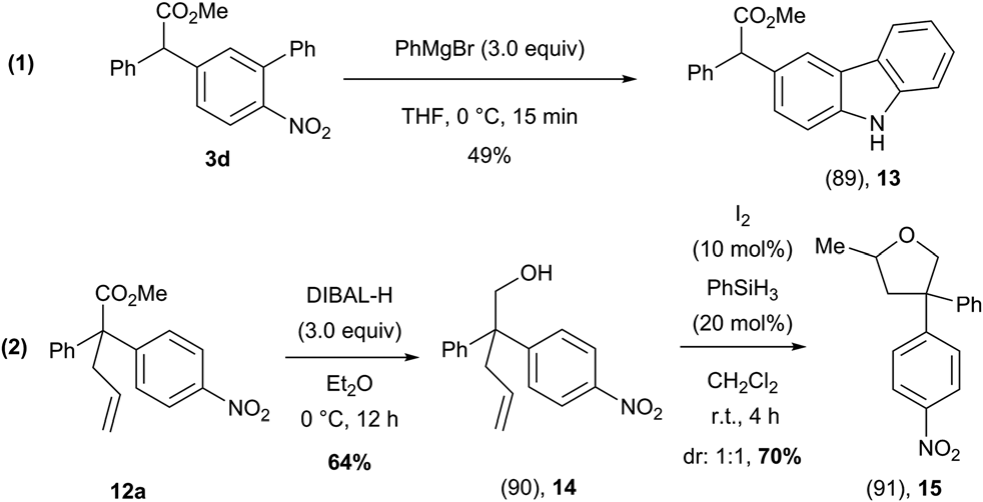
Synthetic utility of α-arylated products **3d** and **12a** for the preparation of heterocycles.

To further show the synthetic utility of these arylated products, we have demonstrated that these compounds could be transformed into atypical heterocyclic motifs (Scheme 10). For example, compound **3d** could be rapidly converted to an unusually substituted carbazole (**13**) using PhMgBr, while the methyl ester functionality remained intact (Scheme 10, eqn (1))^31^ Additionally, compound **12a** could be transformed, in two steps into a structurally complex tetrahydrofuran (**15**) which features an all carbon quaternary center (Scheme 10, eqn (2)).^32^

## Conclusions

In conclusion, we have developed a direct and general mono-arylation of activated C(sp^3^)–H bonds with nitroarenes under transition metal-free conditions. This environmentally friendly aerobic arylation method is capable of delivering up to gram quantities of a wide variety of arylated esters, amides, nitriles, sulfones as well as triaryl methanes. DFT calculations provided a reactivity guide for the identification of suitable arylation substrates. This predictive guide states that C(sp^3^)–H bonds within the optimal p*K*_a_ range of 16 to 28 will readily undergo arylation. Our studies have confirmed the robustness of this reactivity guide. These optimized arylation conditions can also be employed in the one-pot synthesis of a diverse collection of compounds featuring all-carbon quaternary centers. Finally, the arylated products can be further functionalized and used as valuable starting materials in the synthesis of complex heterocycles.

## Conflicts of interest

There are no conflicts to declare.

## Supplementary Material

Supplementary information

Supplementary information

Crystal structure data
