# Supplementary material for: Transition metal-free direct dehydrogenative arylation of activated C(sp3)–H bonds: synthetic ambit and DFT reactivity predictions
Source: Chem Sci. 2018 Aug 27;9(41):7992–9. doi: 10.1039/c8sc02758g (PMC6202766; doi:10.1039/c8sc02758g)
Supplement: Supplementary file 2 [file SC-009-C8SC02758G-s002.pdf]

Electronic Supplementary Material (ESI) for Chemical Science.
This journal is © The Royal Society of Chemistry 2018

Supporting Information

Table of Contents

General Remarks S2

General Procedure for the Dehydrogenative mono-Arylation........ S2

Analysis Data of Arylated Products S3

Formation of S_NAr Products with *ortho*- and *para*- halogenated Nitroarene Substrates S25

Utilizing *para*-subsituted Nitroarenes as Arylation Substrates S26

Utilizing other Electron Deficient Arenes (i.e. not nitroarenes) as Electrophiles S27

General Procedure for the Arylation of Substrates for Reactivity Predictions........ S28

Analysis Data of pK_a Reactivity Prediction Products S28

General Two-step Preparation of All Carbon Quaternary Center Containing Compounds S29

Analysis Data for All Carbon Quaternary Center Containing Compounds Prepared in Two Steps S30

General One-pot Preparation of all Carbon Quaternary Center Containing Compounds S30

Analysis Data of all Carbon Quaternary Center Containing Compounds Prepared via One-pot Method .. S31

Experimental Procedures for the Formation of Heterocycles and Intermediates S39

Analysis Data of Heterocycles and Intermediates........ S40

Analysis Data of Arylation Products Formed with *para*-substituted Nitroarenes........ S41

^{1}H NMR Study of DMS in Crude Reaction Mixture........ S43

Refrences........ S46

X-Ray Diffraction Data – Compound 4g S47

X-Ray Diffraction Data – Compound 12s........ S59

Computational details and xyz coordinates........ S64

^{1}H and ^{13}C NMR of Products........ S85

General Remarks

All reactions were carried out in oven-dried glassware under air with magnetic stirring. All nitroarene compounds, except **2b** and **2c**, were purchased from Sigma-Aldrich Co. or Oakwood Chemicals and used without further purification. Nitroarene coupling partners **2b** and **2c** were synthesized via literature protocols.[1] All activated C(sp^3)-H bond substrates, with the exception of **1a**, **1s**, **5b**, **7c** and **7e**, were purchased from Sigma-Aldrich Co. or Oakwood Chemicals and used without further purification. Substrates **1a**, **1s**, **5b**, **7c** and **7e** were synthesized via literature protocols.[2] All reactions were monitored by thin-layer chromatography (TLC) with E. Merck silica gel 60 F254 pre-coated plates (0.25 mm). Silica gel (particle size 0.032-0.063 mm) purchased from SiliCycle was used for flash chromatography. Proton (^{1}H) and carbon (^{13}C) NMR spectra were recorded on a Bruker AV-400 (or a Bruker DRX-600) spectrometer operating at 400 MHz (or 600 MHz) for proton and 100 MHz (or 151 MHz) for carbon nuclei using $CDCl_3$ [or DMSO-d6] as solvent. Chemical shifts are expressed as parts per million (δ, ppm) and are referenced to 7.26 ($CDCl_3$) or 2.50 (DMSO-d6) for ^{1}H NMR and 77.00 ($CDCl_3$) or 40.45 (DMSO-d6) for ^{13}C NMR. Proton signal data uses the following abbreviations: s = singlet, d = doublet, dd = doublet of doublets, dddd = doublet of doublet of doublet of doublets, ddt = doublet of doublets of triplets, dt = doublet of triplets, t = triplet, td = triplet of doublets, q = quartet, dq = doublet of quartets, m = multiplet and *J* = coupling constant. High Resolution Mass Spectrometry was performed on a Shimadzu LCMS-IT-TOF under the conditions of electrospray ionization (ESI) in both positive and negative mode. Melting points and ranges were recorded on Mettler Toledo MP50 melting point system.

General Procedure for the Dehydrogenative mono-Arylation

t-BuOK (224.0 mg, 2.0 mmol, 2.0 equiv) was added in one portion to a solution of methyl phenylacetate **1** (150 mg, 1.0 mmol) and nitrobenzene **2** (246 mg, 2.0 mmol, 2.0 equiv) in dry DMSO (5 mL) at room temperature. Once the base was added, the mixture was stirred in an open flask at room temperature for 30 minutes. The reaction was quenched via the addition of saturated NH_4Cl solution (5 mL). The resulting mixture was extracted with ethyl acetate (3×10 mL). The organic layers were combined, washed with brine (10 mL), dried over anhydrous Na_2SO_4, filtered and concentrated in *vacuo*. The crude product was purified by column chromatography (Hexanes:Ethyl Acetate = 10:1) to give methyl 2-(4-nitrophenyl)-2-phenylacetate **3** (146.9 mg, 54% yield) as a yellow oil.

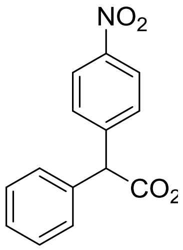

3

^{1}H NMR (400 MHz, $CDCl_3$) δ 8.17 (d, *J* = 9.2 Hz, 2H), 7.50 (d, *J* = 8.8 Hz, 2H), 7.37-7.29 (m, 5H), 5.13 (s, 1H), 3.78 (s, 3H); ^{13}C NMR (100 MHz, $CDCl_3$) δ 171.8, 147.0, 145.8, 137.1, 129.6, 128.9, 128.4, 127.8, 123.7, 56.5, 52.6; HRMS (ESI): Exact mass calcd. for $C_{15}H_{14}NO_4$ $[M+H]^+$: 27222.0917. Found: 272.0912.

Analysis Data of Arylated Products

1. methyl 2-(3-cyano-4-nitrophenyl)-2-phenylacetate (**3a**)

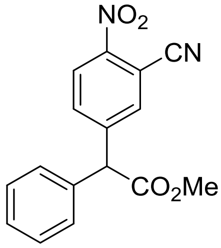

3a

Yellow oil, 72% yield. ^{1}H NMR (400 MHz, $CDCl_3$) δ 8.26 (d, *J* = 8.8 Hz, 1H), 7.86 (d, *J* = 1.6 Hz, 1H), 7.77 (dd, *J* = 2.0, 8.4 Hz, 1H), 7.39-7.27 (m, 5H), 5.15 (s, 1H), 3.79 (s, 3H); ^{13}C NMR (100 MHz, $CDCl_3$) δ 170.9, 147.2, 146.2, 136.0, 135.7, 133.9, 129.3, 128.4, 128.2, 125.6, 114.8, 108.1, 55.9, 52.9; HRMS (ESI): Exact mass calcd. for $C_{16}H_{12}N_2O_4Na$ $[M+Na]^+$: 319.0686. Found: 319.0689.

2. methyl 5-(2-methoxy-2-oxo-1-phenylethyl)-2-nitrobenzoate (**3b**)

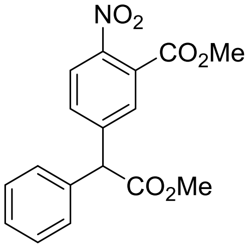

3b

Yellow oil, 35% yield. ^{1}H NMR (400 MHz, $CDCl_3$) δ 7.87 (d, *J* = 8.4 Hz, 1H), 7.67 (d, J = 1.6 Hz, 1H), 7.41 (dd, *J* = 2.0, 8.0 Hz, 1H), 7.38-7.25 (m, 5H), 5.10 (s, 1H), 3.90 (s, 3H), 3.77 (s, 3H); ^{13}C NMR (100 MHz, $CDCl_3$) δ 171.4, 165.7, 146.9, 144.4, 136.7, 131.8, 130.0, 129.1, 128.3, 128.1,

127.9, 124.2, 56.3, 53.3, 52.8; HRMS (ESI): Exact mass calcd. for $C_{17}H_{15}NO_6Na$ $[M+Na]^+$: 352.0789. Found: 352.0792.

3. *tert*-butyl 5-(2-methoxy-2-oxo-1-phenylethyl)-2-nitrobenzoate (**3b'**)

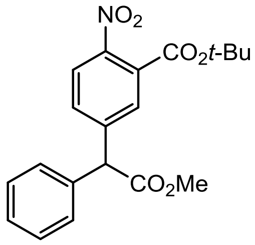

3b'

Colorless oil, 50% yield. ^1H NMR (600 MHz, $CDCl_3$) δ 7.79 (d, *J* = 8.4 Hz, 1H), 7.66 (d, J = 2.0 Hz, 1H), 7.55 (dd, *J* = 2.0, 8.4 Hz, 1H), 7.35 (t, *J* = 7.5 Hz, 1H), 7.28-7.32 (m, 3H), 5.10 (s, 1H), 3.77 (s, 3H), 1.55 (s, 9H); ^{13}C NMR (151 MHz, $CDCl_3$) δ 171.5, 164.0, 147.3, 143.9, 136.8, 131.4, 130.2, 129.12, 129.10 128.4, 128.0, 123.9, 83.9, 56.3, 52.7, 27.7; HRMS (ESI): Exact mass calcd. for $C_{20}H_{21}NO_6Na$ $[M+Na]^+$: 394.1261. Found: 394.1257.

4. methyl 2-(3-(dimethylcarbamoyl)-4-nitrophenyl)-2-phenylacetate (**3c**)

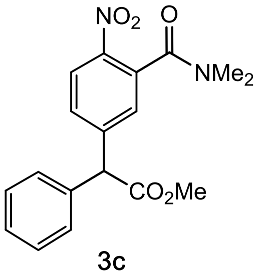

3c

Yellow oil, 55% yield. ^1H NMR (600 MHz, DMSO-d6) δ 8.16 (d, *J* = 8.6 Hz, 1H), 7.63 (dd, *J* = 8.6, 2.0 Hz, 1H), 7.48 (d, *J* = 2.0 Hz, 1H), 7.38-7.33 (m, 4H), 7.30 (t, *J* = 7.0 Hz, 1H) 5.49 (s, 1H), 3.70 (s, 3H), 3.00 (s, 3H), 2.76 (s, 3H); ^{13}C NMR (151 MHz, DMSO-d6) δ 171.9, 166.9, 146.7, 144.3, 138.3, 133.4, 130.5, 129.4 (2C), 128.8 (2C), 128.6, 128.0, 125.4, 55.3, 53.0, 38.3, 34.7; HRMS (ESI): Exact mass calcd. for $C_{18}H_{19}N_2O_5$ $[M+H]^+$: 343.1288. Found: 343.1281.

5. methyl 2-(6-nitro-[1,1'-biphenyl]-3-yl)-2-phenylacetate (**3d**)

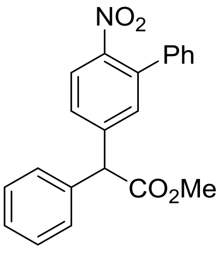

3d

Yellow oil, 46% yield. ^1H NMR (400 MHz, $CDCl_3$) δ 7.83 (d, *J* = 8.8 Hz, 1H), 7.47-7.29 (m, 12H), 5.12 (s, 1H), 3.78 (s, 3H), 2.27 (s, 3H); ^{13}C NMR (100 MHz, $CDCl_3$) δ 171.9, 148.1, 143.3,

137.2, 137.1, 136.6, 132.2, 129.0, 128.6 128.4, 128.3, 127.9, 124.4, 56.5, 52.6; HRMS (ESI): Exact mass calcd. for $C_{21}H_{18}NO_4$ $[M+H]^+$: 348.1230. Found: 348.1224.

6. methyl 2-(4-nitro-3-(phenylthio)phenyl)-2-phenylacetate (**3e**)

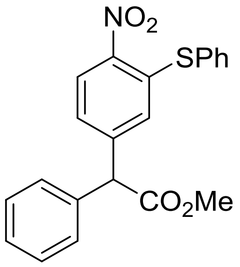

3e

Yellow solid, m.p. 95-96 °C. 35% yield. ^1H NMR (400 MHz, $CDCl_3$) δ 8.17 (d, *J* = 8.8 Hz, 1H), 7.50-7.40 (m, 5H), 7.26-7.23 (m, 3H), 7.12 (dd, *J* = 1.6, 8.4 Hz, 1H), 7.06-7.03 (m, 2H), 6.73 (d, *J* = 1.6 Hz, 1H), 4.83 (s, 1H), 3.62 (s, 3H); ^{13}C NMR (100 MHz, $CDCl_3$) δ 171.4, 144.7, 143.7, 139.9, 136.6, 135.8, 130.6, 130.04, 129.96, 128.9, 128.3, 127.7, 125.8, 125.2, 56.4, 52.5; HRMS (ESI): Exact mass calcd. for $C_{21}H_{17}NO_4S$ $[M-H]^-$: 378.0806. Found: 378.0816.

7. methyl 2-(3-(1H-indol-1-yl)-4-nitrophenyl)-2-phenylacetate (**3f**)

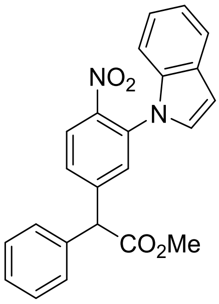

3f

Yellow oil, 62% yield. ^1H NMR (400 MHz, $CDCl_3$) δ 8.00 (d, *J* = 8.0 Hz, 1H), 7.70-7.67 (m, 1H), 7.58 (d, *J* = 2.0 Hz, 1H), 7.51 (dd, *J* = 1.6, 8.4 Hz, 1H), 7.40-7.33 (m, 6H), 7.22-7.14 (m, 3H), 6.73 (d, *J* = 2.8 Hz, 1H), 5.15 (s, 1H), 3.80 (s, 3H); ^{13}C NMR (100 MHz, $CDCl_3$) δ 171.5, 145.2, 144.6, 136.8, 136.4, 132.8, 129.8, 129.1, 128.9, 128.31, 128.25, 128.1, 127.8, 125.7, 122.9, 121.3, 120.9, 109.4, 105.1, 56.3, 52.8; HRMS (ESI): Exact mass calcd. for $C_{23}H_{18}N_2O_4$ $[M-H]^-$: 385.1194. Found: 385.1200.

8. methyl 2-(3-(9H-carbazol-9-yl)-4-nitrophenyl)-2-phenylacetate (**3g**)

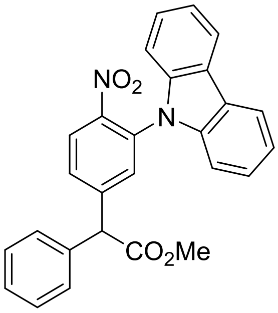

3g

Yellow oil, 70% yield. ^{1}H NMR (400 MHz, $CDCl_3$) δ 8.15-8.11 (m, 3H), 7.67 (d, *J* = 1.6 Hz, 1H), 7.60 (dd, J = 2.0, 8.8 Hz, 1H), 7.41-7.29 (m, 9H), 7.14 (d, *J* = 8.4 Hz, 1H), 7.10 (d, *J* = 8.0 Hz, 1H), 5.17 (s, 1H), 3.79 (s, 3H); ^{13}C NMR (100 MHz, $CDCl_3$) δ 171.5, 145.9, 145.6, 140.42, 140.39, 136.8, 131.4, 131.2, 129.2, 129.0, 128.3, 128.1, 126.30, 126.27, 126.2, 123.8, 120.7, 120.5, 108.99, 108.96, 56.3, 52.8; HRMS (ESI): Exact mass calcd. for $C_{27}H_{20}N_2O_4$ $[M-H]^-$: 435.1350. Found: 435.1360.

9. methyl 2-(2-cyano-4-nitrophenyl)-2-phenylacetate (**3h**)

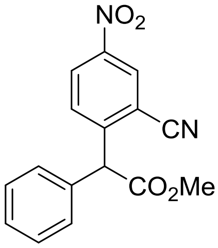

3h

Yellow oil, 56% yield. ^{1}H NMR (400 MHz, $CDCl_3$) δ 8.49 (d, *J* = 2.4 Hz, 1H), 8.36 (dd, *J* = 2.4, 8.8 Hz, 1H), 7.79 (d, *J* = 8.8 Hz, 1H), 7.39-7.32 (m, 5H), 5.55 (s, 1H), 3.80 (s, 3H); ^{13}C NMR (100 MHz, $CDCl_3$) δ 170.5, 148.6, 146.6, 137.5, 135.4, 130.9, 129.3, 128.4, 127.8, 127.4, 115.5, 114.2, 54.5, 53.0; HRMS (ESI): Exact mass calcd. for $C_{16}H_{12}N_2O_4Na$ $[M+Na]^+$: 319.0680. Found: 319.0689.

10. methyl 2-(2-fluoro-4-nitrophenyl)-2-phenylacetate (**3i**)

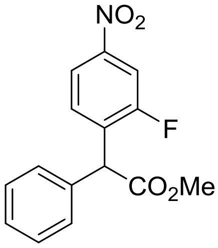

3i

Yellow oil, 61% yield. ^{1}H NMR (600 MHz, $CDCl_3$) δ 7.96 (dd, *J* = 8.4, 1.8 Hz, 1H), 7.91 (dd, *J* = 9.6, 1.8 Hz, 1H), 7.49-7.44 (m, 1H), 7.41-7.37 (m, 2H), 7.36-7.30 (m, 3H), 5.36 (s, 1H), 3.78 (s, 3H); ^{13}C NMR (151 MHz, $CDCl_3$) δ 171.0, 159.6 (d, *J* = 252.0 Hz), 147.7 (d, *J* = 8.8 Hz), 135.5, 133.6 (d, *J* = 14.2 Hz), 130.7 (d, *J* = 3.2 Hz), 129.0, 128.5, 128.0, 119.1 (d, *J* = 4.4 Hz), 111.0 (d, *J* = 27.6 Hz), 52.6, 49.7 (d, *J* = 3.3 Hz); HRMS (ESI): Exact mass calcd. for $C_{15}H_{13}FNO_4$ $[M+H]^+$: 290.0823. Found: 290.0810.

11. methyl 2-(2-bromo-4-nitrophenyl)-2-phenylacetate (**3j**)

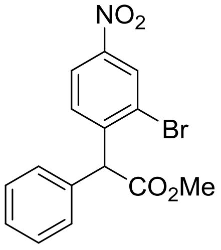

3j

Yellow oil, 53% yield. ^{1}H NMR (600 MHz, $CDCl_3$) δ 8.45 (d, *J* = 1.8 Hz, 1H), 8.09 (dd, *J* = 9.0, 1.8 Hz, 1H), 7.43 (d, *J* = 9.0 Hz, 1H), 7.41-7.37 (m, 2H), 7.36-7.32 (m, 1H), 7.29 (d, *J* = 7.2 Hz, 2H), 5.54 (s, 1H), 3.79 (s, 3H); ^{13}C NMR (151 MHz, $CDCl_3$) δ 171.1, 147.1, 145.3, 135.9, 131.0, 129.1, 128.7, 128.1, 127.8, 125.0, 122.2, 56.2, 52.8; HRMS (ESI): Exact mass calcd. for $C_{15}H_{13}BrNO_4$ $[M+H]^+$: 350.0022. Found: 350.0005.

12. methyl 2-(2-fluoro-6-iodo-4-nitrophenyl)-2-phenylacetate (**3k**)

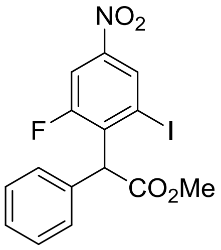

3k

Yellow oil, 41% yield. ^{1}H NMR (600 MHz, $CDCl_3$) δ 8.56 (dd, *J* = 1.6, 0.8 Hz, 1H), 7.97 (dd, *J* = 9.6, 1.8 Hz, 1H), 7.39-7.28 (m, 5H), 5.45 (s, 1H), 3.78 (s, 3H); ^{13}C NMR (151 MHz, $CDCl_3$) δ 170.2, 158.9 (d, *J* = 256.4 Hz), 147.7 (d, *J* = 10.0 Hz), 137.9 (d, *J* = 15.4 Hz), 134.7, 130.5 (d, *J* = 3.3 Hz), 129.2 (d, *J* = 3.3 Hz), 128.7, 128.1, 112.2 (d, *J* = 28.5 Hz), 101.6 (d, *J* = 4.4 Hz), 58.0, 52.9; HRMS (ESI): Exact mass calcd. for $C_{15}H_{12}FINO_4$ $[M+H]^+$: 415.9795. Found: 415.9794.

13. methyl 2-(4-nitronaphthalen-1-yl)-2-phenylacetate (**3l**)

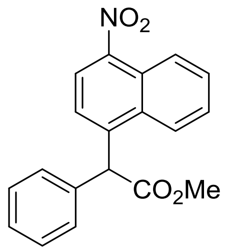

3l

Yellow oil, 65% yield. ^{1}H NMR (400 MHz, $CDCl_3$) δ 8.53 (d, *J* = 9.6 Hz, 1H), 8.15 (d, *J* = 8.0 Hz, 1H), 8.10 (d, *J* = 7.6 Hz, 1H), 7.71-7.64 (m, 2H), 7.74-7.31 (m, 6H), 5.86 (s, 1H), 3.79 (s, 3H); ^{13}C NMR (100 MHz, $CDCl_3$) δ 172.1, 146.5, 141.2, 136.6, 132.3, 129.0, 128.83, 128.81, 127.94, 127.90, 125.3, 125.0, 123.8, 123.6, 123.0, 53.7, 52.7; HRMS (EI): Exact mass calcd. for $C_{19}H_{15}NO_4Na$ $[M+Na]^+$: 344.0885. Found: 344.0893.

14. methyl 2-(5-nitroisoquinolin-8-yl)-2-phenylacetate (**3m**)

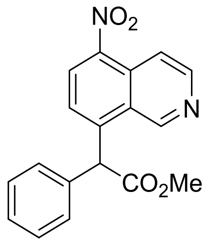

3m

Yellow soild, m.p. 120-122 °C. 33% yield. ^{1}H NMR (600 MHz, $CDCl_3$) δ 9.65 (br s, 1H), 8.76 (d, *J* = 6.6 Hz, 1H), 8.48 (d, *J* = 6.0 Hz, 1H), 8.42 (d, *J* = 9.0 Hz, 1H), 7.57 (d, *J* = 7.8 Hz, 1H), 7.42-7.37 (m, 2H), 7.36-7.30 (m, 3H), 5.98 (s, 1H), 3.80 (s, 3H); ^{13}C NMR (151 MHz, $CDCl_3$) δ 171.5, 148.6, 145.9, 144.4, 143.1, 136.0, 129.2, 128.79, 128.76, 128.7, 128.3, 127.8, 126.6, 116.2, 53.0, 52.9; HRMS (EI): Exact mass calcd. for $C_{18}H_{15}N_2O_4$ $[M+H]^+$: 323.1026. Found: 323.1117.

15. tert-butyl 2-(3-cyano-4-nitrophenyl)-2-phenylacetate (**4a**)

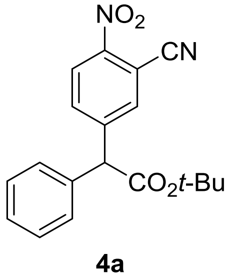

4a

Tan solid, m.p. 99.9-101.7 °C. 60% yield. ^{1}H NMR (600 MHz, $CDCl_3$) δ 8.26 (d, *J* = 8.6 Hz, 1H), 7.83 (d, *J* = 2.0 Hz, 1H), 7.74 (dd, *J* = 8.7, 2.0 Hz, 1H), 7.39 (t, *J* = 7.4 Hz, 2H), 7.34 (t, *J* = 7.3 Hz, 1H), 7.27 (d, *J* = 8.9 Hz, 2H), 5.01 (s, 1H), 1.46 (s, 9H); ^{13}C NMR (151 MHz, $CDCl_3$) δ 169.7,

147.3, 147.0, 136.7, 135.9, 134.0 (2C), 129.4 (2C), 128.3 (2C), 125.6, 115.0, 108.2, 83.1, 57.2, 28.0 (3C); HRMS (EI): Exact mass calcd. for $C_{19}H_{19}N_2O_4$ [M+H]$^+$: 339.1339. Found: 339.1332.

16. methyl 2-(4-nitrophenyl)-2-(o-tolyl)acetate (**4b**)

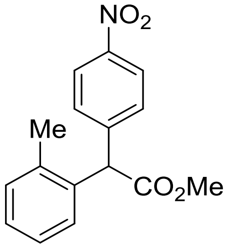

4b

Yellow oil, 45% yield. ^{1}H NMR (400 MHz, $CDCl_3$) δ 8.17 (d, *J* = 8.8 Hz, 2H), 7.41 (d, *J* = 8.8 Hz, 2H), 7.25-7.23 (m, 4H), 5.32 (s, 1H), 3.78 (s, 3H), 2.27 (s, 3H); ^{13}C NMR (100 MHz, $CDCl_3$) δ 172.1, 147.0, 145.2, 136.3, 135.5, 131.0, 129.9, 128.0, 127.8, 126.6, 123.6, 53.3, 52.6, 19.7; HRMS (ESI): Exact mass calcd. for $C_{16}H_{15}NO_4Na$ [M+Na]$^+$: 308.0885. Found: 308.0893.

17. methyl 2-(3-cyano-4-nitrophenyl)-2-(o-tolyl)acetate (**4b'**)

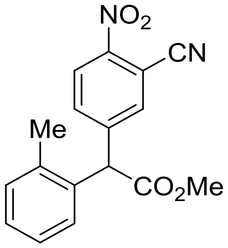

4b'

Yellow solid, m.p. 87-88 °C. 74% yield. ^{1}H NMR (400 MHz, $CDCl_3$) δ 8.26 (d, *J* = 8.8 Hz, 2H), 7.75 (d, *J* = 2.0 Hz, 1H), 7.67 (dd, *J* = 2.0, 8.4 Hz, 1H), 7.29-7.21 (m, 4H), 5.33 (s, 1H), 3.79 (s, 3H), 2.26 (s, 3H); ^{13}C NMR (100 MHz, $CDCl_3$) δ 171.4, 147.2, 145.6, 136.2, 136.0, 134.3, 134.2, 131.4, 128.6, 127.6, 127.0, 125.5, 114.9, 108.1, 53.0, 52.7, 19.7; HRMS (ESI): Exact mass calcd. for $C_{17}H_{14}N_2O_4$ [M-H]$^-$: 309.0881. Found: 309.0871.

18. methyl 2-(2-methoxyphenyl)-2-(4-nitrophenyl)acetate (**4c**)

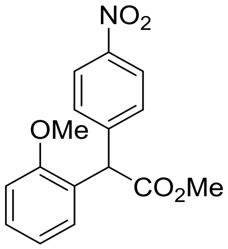

4c

Yellow oil, 46% yield. ^{1}H NMR (400 MHz, $CDCl_3$) δ 8.16 (d, *J* = 9.2 Hz, 2H), 7.48 (d, *J* = 8.4 Hz, 2H), 7.32-7.27 (m, 1H), 7.12 (dd, *J* = 1.2, 8.0 Hz, 1H), 6.97-6.90 (m, 2H), 5.44 (s, 1H), 3.82 (s, 3H), 3.75 (s, 3H); ^{13}C NMR (100 MHz, $CDCl_3$) δ 172.3, 156.6, 147.0, 145.5, 129.9, 129.1, 128.7,

126.1, 123.5, 120.8, 110.8, 55.5, 52.5, 50.4; HRMS (ESI): Exact mass calcd. for $C_{16}H_{14}NO_5$ $[M-H]^-$: 300.0882. Found: 300.0877.

19. methyl 2-(3-cyano-4-nitrophenyl)-2-(2-methoxyphenyl)acetate (**4c'**)

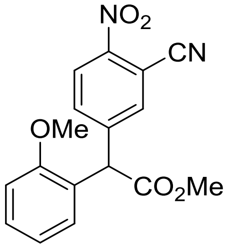

4c'

Yellow oil, 72% yield. ^{1}H NMR (400 MHz, $CDCl_3$) δ 8.24 (d, J = 8.4 Hz, 1H), 7.82 (d, J = 1.6 Hz, 1H), 7.74 (dd, J = 2.0, 8.8 Hz, 1H), 7.36-7.31 (m, 1H), 7.21-7.19 (m, 1H), 7.01-6.97 (m, 1H), 6.92 (d, J = 8.4 Hz, 1H), 5.42 (s, 1H), 3.81 (s, 3H), 3.77 (s, 3H); ^{13}C NMR (100 MHz, $CDCl_3$) δ 171.4, 156.4, 147.1, 146.1, 136.1, 134.2, 129.8, 128.5, 125.4, 124.9, 121.2, 115.1, 111.2, 107.9, 55.5, 52.8, 50.1; HRMS (ESI): Exact mass calcd. for $C_{17}H_{14}N_2O_5$ $[M-H]^-$: 325.0830. Found: 325.0846.

20. methyl 2-(3,4-dimethoxyphenyl)-2-(4-nitrophenyl)acetate (**4d**)

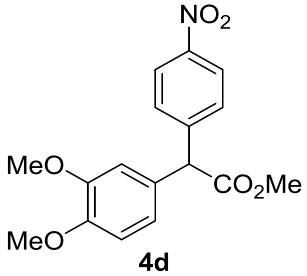

4d

Yellow oil, 48% yield. ^{1}H NMR (400 MHz, $CDCl_3$) δ 8.15 (d, J = 8.4 Hz, 2H), 7.46 (d, J = 9.2 Hz, 2H), 6.83 J (s, 2H), 6.81 (s, 1H), 5.05 (s, 1H), 3.85 (s, 3H), 3.83 (s, 3H), 3.76 (s, 3H); ^{13}C NMR (100 MHz, $CDCl_3$) δ 172.0, 149.2, 148.7, 147.0, 146.0, 129.4, 129.3, 123.6, 120.7, 111.5, 111.2, 56.00, 55.96, 55.8, 52.6; HRMS (EI): Exact mass calcd. for $C_{17}H_{17}NO_6Na$ $[M+Na]^+$: 354.0935. Found: 354.0948.

21. methyl 2-(3-cyano-4-nitrophenyl)-2-(3,4-dimethoxyphenyl)acetate (**4d'**)

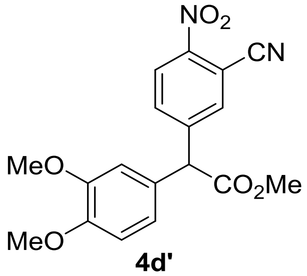

4d'

Yellow oil, 73% yield. ^{1}H NMR (400 MHz, $CDCl_3$) δ 8.25 (d, J = 8.8 Hz, 1H), 7.82 (d, J = 2.0 Hz, 1H), 7.24 (dd, J = 2.0, 8.8 Hz, 1H), 6.87-6.83 (m, 2H), 6.76 (d, J = 1.6 Hz, 1H), 5.06 (s, 1H), 3.86 (s,

3H), 3.84 (s, 3H), 3.78 (s, 3H); ^{13}C NMR (100 MHz, $CDCl_3$) δ 171.1, 149.5, 149.1, 147.1, 146.5, 135.5, 133.7, 128.2, 125.6, 120.6, 114.8, 111.5, 111.3, 108.0, 55.92, 55.87, 55.4, 52.9; HRMS (ESI): Exact mass calcd. for $C_{18}H_{16}N_2O_6$ $[M-H]^-$: 355.0936. Found: 355.0949.

22. methyl 2-(4-(benzyloxy)phenyl)-2-(3-cyano-4-nitrophenyl)acetate (**4e**)

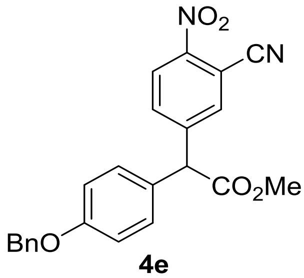

4e

Yellow oil, 80% yield. ^{1}H NMR (600 MHz, $CDCl_3$) δ 8.26 (d, *J* = 8.4 Hz, 1H), 7.82 (d, *J* = 1.8 Hz, 1H), 7.73 (dd, *J* = 8.4, 1.2 Hz, 1H), 7.44-7.37 (m, 4H), 7.37 (t, *J* = 7.2 Hz, 1H), 7.22-7.16 (m, 2H), 7.02-6.96 (m, 2H), 5.07 (s, 3H), 3.79 (s, 3H); ^{13}C NMR (151 MHz, $CDCl_3$) δ 171.2, 158.8, 147.3, 146.6, 136.5, 135.7, 133.7, 129.5, 128.7, 128.2, 128.1, 127.5, 125.6, 115.7, 114.9, 108.2, 70.1, 55.2, 53.0; HRMS (ESI): Exact mass calcd. for $C_{17}H_{13}N_2O_5$ $[M-C_6H_5]^-$: 325.0800. Found: 325.0793.

23. methyl 2-(3-cyano-4-nitrophenyl)-2-(2-fluorophenyl)acetate (**4f**)

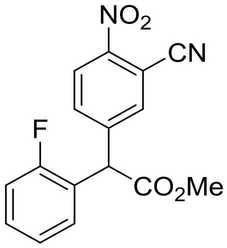

4f

Yellow oil, 82% yield. ^{1}H NMR (600 MHz, $CDCl_3$) δ 8.27 (d, *J* = 8.4 Hz, 1H), 7.85 (d, *J* = 1.8 Hz, 1H), 7.76 (dd, *J* = 9.0, 3.0 Hz, 1H), 7.39-7.29 (m, 2H), 7.23-7.17 (m, 1H), 7.13-7.08 (m, 1H), 5.39 (s, 1H), 3.80 (s, 3H); ^{13}C NMR (151 MHz, $CDCl_3$) δ 170.3, 160.1 (d, *J* = 247.8 Hz), 147.4, 145.0, 135.8, 134.0, 130.4 (d, *J* = 8.8 Hz), 129.1 (d, *J* = 2.3 Hz), 125.7, 125.0 (d, *J* = 3.3 Hz), 123.7 (d, *J* = 14.3 Hz), 116.1 (d, *J* = 21.9 Hz), 114.8, 108.2, 53.1, 49.2 (d, *J* = 3.3 Hz); HRMS (ESI): Exact mass calcd. for $C_{16}H_{12}FN_2O_4$ $[M+H]^+$: 315.0776. Found: 315.0913.

24. methyl 2-(3-cyano-4-nitrophenyl)-2-(3-(trifluoromethyl)phenyl)acetate (**4g**)

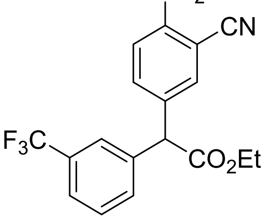

4g

Yellow solid, m.p. 56-57 °C. 64% yield. ^{1}H NMR (600 MHz, $CDCl_3$) δ 8.29 (d, *J* = 8.4 Hz, 1H), 7.86 (d, *J* = 1.8 Hz, 1H), 7.77 (dd, *J* = 9.0, 1.8 Hz, 1H), 7.62 (d, *J* = 7.2 Hz, 1H), 7.57-7.50 (m, 3H), 5.19 (s, 1H), 4.27 (q, *J* = 7.2 Hz, 2H), 1.28 (t, *J* = 7.2 Hz, 3H); ^{13}C NMR (151 MHz, $CDCl_3$) δ 169.8, 147.5, 145.4, 137.1, 135.6, 133.8, 131.67, 131.66 (q, *J* = 32.0 Hz), 129.9, 125.9, 125.3 (q, *J* = 3.2 Hz), 125.2 (q, *J* = 4.5 Hz), 123.6 (q, *J* = 272.0 Hz), 114.7, 108.4, 62.5, 55.8, 13.9; HRMS (ESI): Exact mass calcd. for $C_{18}H_{13}F_3N_2O_4Na$ $[M+Na]^+$: 401.0720 Found: 401.0679.

25. methyl 2-(2-bromophenyl)-2-(3-cyano-4-nitrophenyl)acetate (**4h**)

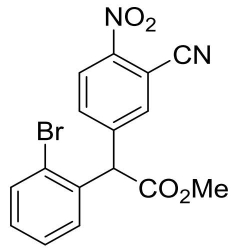

4h

Yellow oil, 79% yield. ^{1}H NMR (600 MHz, $CDCl_3$) δ 8.19 (d, *J* = 9.0 Hz, 1H), 7.82 (d, *J* = 1.8 Hz, 1H), 7.66 (dd, *J* = 9.0, 1.8 Hz, 1H), 7.54 (dd, *J* = 8.4, 1.2 Hz, 1H), 7.34-7.27 (m, 2H), 7.20-7.13 (m, 1H), 5.55 (s, 1H), 3.72 (s, 3H); ^{13}C NMR (151 MHz, $CDCl_3$) δ 170.5, 147.3, 144.7, 136.0, 135.5, 134.3, 133.7, 130.0, 129.2, 128.3, 125.5, 124.8, 114.8, 108.1, 55.1, 53.1; HRMS (ESI): Exact mass calcd. for $C_{16}H_{12}BrN_2O_4$ $[M+H]^+$: 374.9975. Found: 374.9926.

26. methyl 2-(3-bromophenyl)-2-(4-nitrophenyl)acetate (**4i**)

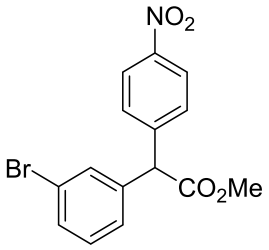

4i

Yellow oil, 45% yield. ^{1}H NMR (400 MHz, $CDCl_3$) δ 8.18 (d, *J* = 8.4 Hz, 2H), 7.49-7.44 (m, 4H), 7.24-7.22 (m, 2H), 5.07 (s, 1H), 3.78 (s, 3H); ^{13}C NMR (100 MHz, $CDCl_3$) δ 171.2, 147.3, 145.0, 139.3, 131.5, 131.1, 130.5, 129.5, 127.1, 123.9, 123.0, 56.1, 52.8; HRMS (ESI): Exact mass calcd. for $C_{15}H_{11}NO_4Br$ $[M-H]^-$: 347.9889. Found: 347.9877.

27. methyl 2-(3-bromophenyl)-2-(3-cyano-4-nitrophenyl)acetate (**4i'**)

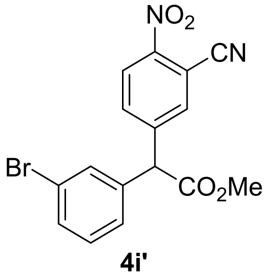

4i'

Yellow oil, 60% yield. ^1H NMR (400 MHz, $CDCl_3$) δ 8.28 (d, *J* = 8.4 Hz, 1H), 7.84 (d, *J* = 2.0 Hz, 1H), 7.45 (dd, *J* = 8.8, 2.0 Hz, 1H), 7.48 (d, *J* = 7.6 Hz, 1H), 7.43 (d, *J* = 1.6 Hz, 1H), 7.28-7.21 (m, 2H), 5.09 (s, 1H), 3.80 (s, 3H); ^{13}C NMR (100 MHz, $CDCl_3$) δ 170.4, 147.4, 145.4, 138.1, 135.6, 133.8, 131.6, 131.4, 130.9, 126.9, 125.8, 123.3, 114.7, 108.3, 55.4, 53.2; HRMS (ESI): Exact mass calcd. for $C_{16}H_{11}N_2O_4Br$ $[M-H]^-$: 372.9829. Found: 372.9832.

28. methyl 2-(3,4-dichlorophenyl)-2-(4-nitrophenyl)acetate (**4j**)

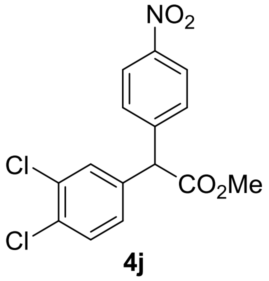

4j

Yellow oil, 37% yield. ^1H NMR (400 MHz, $CDCl_3$) δ 8.18 (d, *J* = 8.8 Hz, 2H), 7.47 (d, *J* = 8.8 Hz, 2H), 7.43-7.39 (m, 2H), 7.14 (dd, *J* = 1.8, 8.8 Hz, 1H), 5.06 (s, 1H), 3.78 (s, 3H); ^{13}C NMR (100 MHz, $CDCl_3$) δ 171.0, 147.3, 144.6, 137.2, 133.0, 132.2, 130.8, 130.4, 129.4, 127.8, 123.9, 55.5, 52.9; HRMS (EI): Exact mass calcd. for $C_{15}H_{10}NO_4Cl_2$ $[M-H]^-$: 337.9995. Found: 337.9992.

29. methyl 2-(3-cyano-4-nitrophenyl)-2-(3,4-dichlorophenyl)acetate (**4j'**)

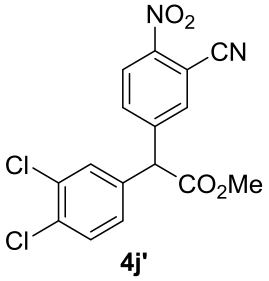

4j'

Yellow solid, m.p. 139-140 °C. 40% yield. ^1H NMR (400 MHz, $CDCl_3$) δ 8.29 (d, *J* = 8.4 Hz, 1H), 7.84 (d, *J* = 2.4 Hz, 1H), 7.75 (dd, *J* = 8.8, 2.0 Hz, 1H), 7.46 (d, *J* = 8.4 Hz, 1H), 7.39 (d, *J* = 2.4 Hz, 1H), 7.14 (dd, *J* = 8.4, 2.0 Hz, 1H), 5.09 (s, 1H), 3.81 (s, 3H); ^{13}C NMR (100 MHz, $CDCl_3$) δ 170.2, 147.5, 145.0, 136.0, 135.5, 133.7, 133.5, 133.0, 131.2, 130.3, 127.6, 125.9, 114.6, 108.5, 54.8, 53.3; HRMS (EI): Exact mass calcd. for $C_{16}H_{11}Cl_2N_2O_4$ $[M+H]^+$: 365.0090. Found: 365.0053.

30. methyl 2-(3-cyano-4-nitrophenyl)-2-(4-fluorophenyl)acetate (**4k**)

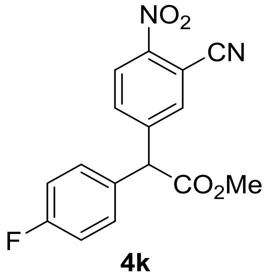

4k

Yellow soild, m.p. 89-91 °C. 73% yield. ^{1}H NMR (600 MHz, $CDCl_3$) δ 8.30 (d, *J* = 9.0 Hz, 1H), 7.86 (d, *J* = 1.8 Hz, 1H), 7.76 (dd, *J* = 9.0, 1.8 Hz, 1H), 7.29 (dt, *J* = 5.4, 1.8 Hz, 2H), 7.13-7.08 (m, 2H), 5.14 (s, 1H), 3.82 (s, 3H); ^{13}C NMR (151 MHz, $CDCl_3$) δ 170.8, 162.5 (d, *J* = 248.8 Hz), 147.4, 146.0, 135.6, 133.7, 131.8 (d, *J* = 3.3 Hz), 130.1 (d, *J* = 8.9 Hz), 125.7, 116.4 (d, *J* = 20.8 Hz), 114.7, 108.3, 55.1, 53.1; HRMS (EI): Exact mass calcd. for $C_{16}H_{12}FN_2O_4$ $[M+H]^+$: 315.0776. Found: 315.0772.

31. methyl 2-(4-bromophenyl)-2-(3-cyano-4-nitrophenyl)acetate (**4l**)

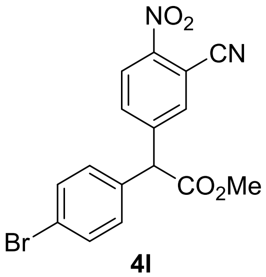

4l

Yellow solid, m.p. 78-80 °C. 60% yield. ^{1}H NMR (600 MHz, $CDCl_3$) δ 8.27 (d, *J* = 9.0 Hz, 1H), 7.83 (d, *J* = 1.8 Hz, 1H), 7.73 (dd, *J* = 9.0, 1.8 Hz, 1H), 7.52 (d, *J* = 8.4 Hz, 2H), 7.17 (d, *J* = 8.4 Hz, 2H), 5.09 (s, 1H), 3.79 (s, 3H); ^{13}C NMR (151 MHz, $CDCl_3$) δ 170.5, 147.4, 145.6, 135.6, 135.0, 133.8, 132.5, 130.0, 125.8, 122.7, 114.7, 108.3, 55.3, 53.1; HRMS (ESI): Exact mass calcd. for $C_{16}H_{11}N_2O_4Br$ $[M-H]^-$: 372.9829. Found: 372.9837.

32. methyl 2-(3-cyano-4-nitrophenyl)-2-(4-iodophenyl)acetate (**4m**)

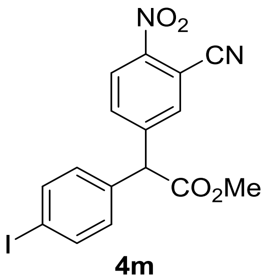

4m

Yellow solid, m.p. 77-78 °C. 56% yield. ^{1}H NMR (600 MHz, $CDCl_3$) δ 8.27 (d, *J* = 9.0 Hz, 1H), 7.83 (d, *J* = 1.8 Hz, 1H), 7.76-7.70 (m, 3H), 7.03 (dd, *J* = 9.0, 1.8 Hz, 2H), 5.07 (s, 1H), 3.79 (s, 3H); ^{13}C NMR (151 MHz, $CDCl_3$) δ 170.5, 147.4, 145.5, 138.5, 135.65, 135.60, 133.8, 130.2, 125.8, 114.7, 108.3, 94.3, 55.4, 53.1; HRMS (ESI): Exact mass calcd. for $C_{16}H_{11}IN_2NaO_4$ $[M+Na]^+$: 444.9656. Found: 444.9650.

33. methyl 2-(4-bromo-2-fluorophenyl)-2-(3-cyano-4-nitrophenyl)acetate (**4n**)

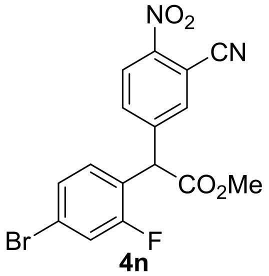

4n

Yellow oil, 80% yield. ^{1}H NMR (600 MHz, $CDCl_3$) δ 8.28 (d, *J* = 8.4 Hz, 1H), 7.83 (d, *J* = 1.8 Hz, 1H), 7.74 (dd, *J* = 8.4, 1.8 Hz, 1H), 7.35 (dd, *J* = 8.4, 1.2 Hz, 1H), 7.32-7.19 (m, 1H), 5.33 (s, 1H), 3.80 (s, 3H); ^{13}C NMR (151 MHz, $CDCl_3$) δ 169.8, 159.9 (d, *J* = 252.0 Hz), 147.5, 144.3, 135.7, 133.9, 130.3 (d, *J* = 3.2 Hz), 128.3 (d, *J* = 4.4 Hz), 125.8, 123.0, 122.9 (d, *J* = 14.2 Hz), 119.8 (d, *J* = 25.4 Hz), 114.6, 108.4, 53.3, 48.8 (d, *J* = 2.1 Hz); HRMS (ESI): Exact mass calcd. for $C_{16}H_{11}Br FN_2O_4$ $[M+H]^+$: 392.9881. Found: 392.9714.

34. methyl 2-(4-bromo-2-fluorophenyl)-2-(2-bromo-4-nitrophenyl)acetate (**4o**)

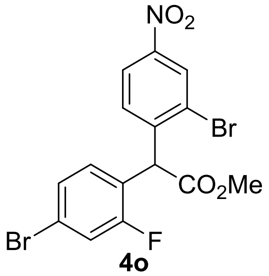

4o

Yellow oil, 52% yield. ^{1}H NMR (600 MHz, $CDCl_3$) δ 8.48 (d, *J* = 2.4 Hz, 1H), 8.48 (dd, *J* = 8.4, 2.4 Hz, 1H), 7.38 (d, *J* = 9.0 Hz, 1H), 7.33-7.28 (m, 2H), 7.03 (t, *J* = 7.8 Hz, 1H), 5.68 (s, 1H), 3.80 (s, 3H); ^{13}C NMR (151 MHz, $CDCl_3$) δ 170.2, 160.3 (d, *J* = 253.2 Hz), 147.6, 143.4, 130.9 (d, *J* = 4.4 Hz), 130.6, 128.2, 128.0 (d, *J* = 3.2 Hz), 125.3, 122.8 (d, *J* = 7.7 Hz), 122.7 (d, *J* = 3.2 Hz), 122.5, 119.7 (d, *J* = 25.4 Hz), 53.1, 49.8 (d, *J* = 3.3 Hz); HRMS (ESI): Exact mass calcd. for $C_{15}H_{10}Br_2FNNaO_4$ $[M+Na]^+$: 467.8853. Found: 467.8846.

35. methyl 2-(naphthalen-2-yl)-2-(4-nitrophenyl)acetate (**4p**)

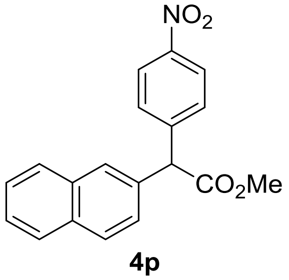

4p

Yellow oil, 54% yield. ^{1}H NMR (400 MHz, $CDCl_3$) δ 8.18 (d, *J* = 8.4 Hz, 2H), 7.86-7.78 (m, 4H), 7.55-7.49 (m, 4H), 7.40 (dd, *J* = 1.2, 8.4 Hz, 1H), 5.30 (s, 1H), 3.82 (s, 3H); ^{13}C NMR (100 MHz, $CDCl_3$) δ 171.8, 147.1, 145.7, 134.5, 133.2, 132.6, 129.6, 128.8, 127.9, 127.6, 127.3, 126.5, 126.4,

126.1, 123.7, 56.6, 52.7; HRMS (ESI): Exact mass calcd. for $C_{19}H_{15}NO_4Na$ $[M+Na]^+$: 344.0891. Found: 344.0893.

36. methyl 2-(3-cyano-4-nitrophenyl)-2-(naphthalen-2-yl)acetate (**4p'**)

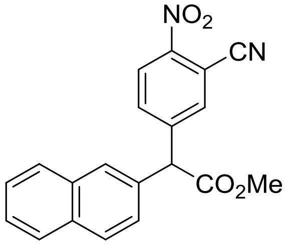

4p'

Yellow oil, 70% yield. ^{1}H NMR (400 MHz, $CDCl_3$) δ 8.26 (d, *J* = 8.8 Hz, 1H), 7.91-7.76 (m, 6H), 7.54-7.51 (m, 2H), 7.34 (dd, *J* =1.6, 8.4 Hz, 1H), 5.30 (s, 1H), 3.82 (s, 3H); ^{13}C NMR (100 MHz, $CDCl_3$) δ 171.0, 147.3, 146.1, 135.8, 134.0, 133.3, 132.8, 129.4, 127.9, 127.7, 127.5, 126.9, 126.8, 125.7, 125.6, 114.8, 108.2, 56.1, 53.1; HRMS (ESI): Exact mass calcd. for $C_{20}H_{15}N_2O_4$ $[M+H]^+$: 347.1026. Found: 347.0956.

37. 4-(4-nitrophenyl)isochroman-3-one (**4q**)

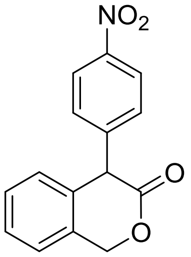

4q

Yellow solid, m.p. 176-177 °C. 33% yield. ^{1}H NMR (400 MHz, $CDCl_3$) δ 8.21 (d, *J* = 8.8 Hz, 2H), 7.40-7.32 (m, 5H), 6.99 (d, *J* = 7.2 Hz, 1H), 5.34 (AB, J_{AB} = 14.0 Hz, 1H), 5.26 (AB, J_{BA} = 14.0 Hz, 1H), 5.07 (s, 1H); ^{13}C NMR (100 MHz, $CDCl_3$) δ 170.1, 147.5, 141.8, 132.8, 131.6, 129.8, 129.3, 128.3, 127.4, 125.1, 124.0, 69.6, 51.4; HRMS (ESI): Exact mass calcd. for $C_{15}H_{10}NO_4$ $[M-H]^-$: 268.0615. Found: 268.0620.

38. ethyl 2-(3-cyano-4-nitrophenyl)-3-phenylpropanoate (**4r**)

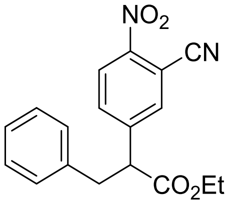

4r

Yellow oil, 32% yield. ^{1}H NMR (600 MHz, $CDCl_3$) δ 8.15 (d, *J* = 9.0 Hz, 1H), 7.74 (d, *J* = 1.8 Hz, 1H), 7.62 (dd, *J* = 8.4, 1.8 Hz, 1H), 7.19-7.14 (m, 2H), 7.13-7.10 (m, 1H), 6.99-6.95 (m, 2H), 4.10-4.00 (m, 2H), 3.93 (t, *J* = 7.8 Hz, 1H), 3.37 (dd, *J* = 14.4, 7.8 Hz, 1H), 2.98 (dd, *J* = 14.4, 7.8 Hz, 1H), 1.09 (t, *J* = 7.8 Hz, 1H); ^{13}C NMR (151 MHz, $CDCl_3$) δ 171.2, 147.3, 146.0, 136.8, 135.2, 133.4, 128.7, 128.6, 127.0, 125.6, 114.8, 108.1, 61.7, 52.9, 39.7, 13.9. HRMS (ESI): Exact mass calcd. for $C_{18}H_{17}N_2O_4$ $[M+H]^+$: 325.1183. Found: 325.1207.

39. methyl 3-(2-bromophenyl)-2-(3-cyano-4-nitrophenyl)propanoate (**4s**)

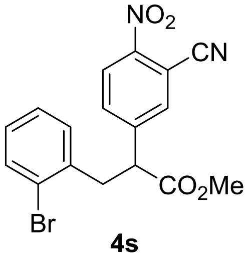

4s

Orange solid, m.p. 111-113 °C. 30% yield. ^{1}H NMR (600 MHz, $CDCl_3$) δ 8.24 (d, *J* = 9.0 Hz, 1H), 7.83 (d, *J* = 1.8 Hz, 1H), 7.72 (dd, *J* = 9.0, 1.8 Hz, 1H), 7.53 (dd, *J* = 8.4, 1.8 Hz, 1H), 7.18-7.14 (m, 1H), 7.12-7.08 (m, 1H), 7.05-7.00 (m, 1H), 4.21 (t, *J* = 8.4 Hz, 1H), 3.67 (s, 3H), 3.53 (dd, *J* = 13.8, 7.8 Hz, 1H), 3.17 (dd, *J* = 13.8, 7.2 Hz, 1H); ^{13}C NMR (151 MHz, $CDCl_3$) δ 171.5, 147.4, 145.7, 136.3, 135.1, 133.3, 133.1, 131.3, 129.0, 127.6, 125.7, 124.4, 114.7, 108.2, 52.7, 50.5, 39.9. HRMS (ESI): Exact mass calcd. for $C_{17}H_{13}BrN_2NaO_4$ $[M+Na]^+$: 410.9951. Found: 410.9957.

40. methyl 2-(4-nitrophenyl)propanoate (**4t**)

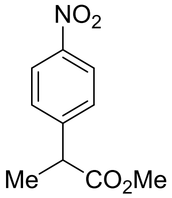

4t

Yellow oil, 15% yield. ^{1}H NMR (400 MHz, $CDCl_3$) δ 8.18 (d, *J* = 8.8 Hz, 2H), 7.47 (d, *J* = 8.8 Hz, 2H), 3.84 (q, *J* = 7.2 Hz, 1H), 3.68 (s, 3H), 1.53 (d, *J* = 7.2 Hz, 3H); ^{13}C NMR (100 MHz, $CDCl_3$) δ 173.6, 147.7, 147.1, 128.5, 123.8, 52.4, 45.2, 18.4.

41. 2-(3-cyano-4-nitrophenyl)-*N*,*N*-dimethyl-2-phenylacetamide (**6a**)

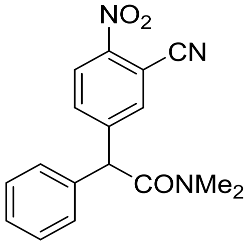

6a

Yellow oil, 82% yield. ^{1}H NMR (400 MHz, $CDCl_3$) δ 8.18 (d, *J* = 8.4 Hz, 1H), 7.73 (d, *J* = 2.0 Hz, 1H), 7.63 (dd, *J* = 2.0, 8.4 Hz, 1H), 7.40-7.25 (m, 5H), 5.36 (s, 1H), 3.01 (s, 3H), 2.99 (s, 3H); ^{13}C NMR (100 MHz, $CDCl_3$) δ 169.4, 148.1, 146.8, 136.4, 136.2, 134.6, 129.5, 128.21, 128.16, 125.1 115.1, 107.4, 54.1, 37.5, 36.1; HRMS (ESI): Exact mass calcd. for $C_{17}H_{15}N_3O_3$ $[M-H]^-$: 308.1041. Found: 308.1045.

42. 2-(3,4-dichlorophenyl)-N,N-dimethyl-2-(4-nitrophenyl)acetamide (**6b**)

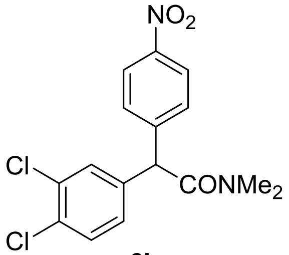

6b

Yellow solid, m.p. 121-122 °C. 45% yield. ^{1}H NMR (400 MHz, $CDCl_3$) δ 8.14 (d, *J* = 8.4 Hz, 2H), 7.40-7.34 (m, 4H), 7.09 (dd, *J* = 2.0, 8.8 Hz, 1H), 5.28 (s, 1H), 3.01 (s, 6H); ^{13}C NMR (100 MHz, $CDCl_3$) δ 169.5, 147.0, 145.9, 138.1, 132.9, 131.8, 130.72, 130.65, 129.8, 128.1, 123.7, 53.2, 37.5, 36.1; HRMS (ESI): Exact mass calcd. for $C_{16}H_{14}Cl_2N_2O_3$ $[M-H]^-$: 351.0309. Found: 351.0313.

43. 2-(3-cyano-4-nitrophenyl)-2-(3,4-dichlorophenyl)-N,N-dimethylacetamide (**6b'**)

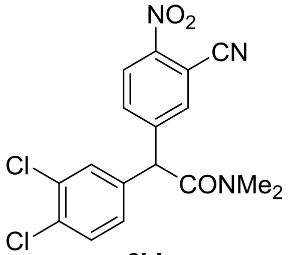

6b'

Yellow solid, m.p. 172-174 °C. 79% yield. ^{1}H NMR (600 MHz, $CDCl_3$) δ 8.24 (d, *J* = 8.4 Hz, 1H), 7.73 (d, *J* = 1.8 Hz, 1H), 7.63 (dd, *J* = 9.0, 1.8 Hz, 1H), 7.48 (d, *J* = 8.4 Hz, 1H), 7.39 (d, *J* = 2.4 Hz, 1H), 7.14 (dd, *J* = 7.8, 2.4 Hz, 1H), 5.30 (s, 1H), 3.04 (s, 6H); ^{13}C NMR (151 MHz, $CDCl_3$) δ 168.6, 147.3, 146.6, 136.5, 136.2, 134.4, 133.8, 132.9, 131.5, 130.4, 127.7, 125.5, 114.8, 108.0, 52.9, 37.7, 36.4; HRMS (ESI): Exact mass calcd. for $C_{16}H_{10}Cl_2N_3O_3$ $[M-CH_3]^-$: 378.0407. Found: 378.0465.

44. 3-(4-nitrophenyl)indolin-2-one (**6c**)

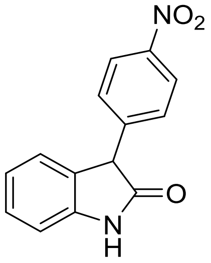

6c

Yellow solid, m.p. 134.9 °C. 40% yield. ^{1}H NMR (400 MHz, $CDCl_3$) δ 8.35 (br s, 1H), 8.21 (d, *J* = 8.4 Hz, 2H), 7.42 (d, *J* = 8.8 Hz, 2H), 7.31 (t, *J* = 7.2 Hz, 1H), 7.14-7.06 (m, 2H), 6.97 (d, *J* = 7.6 Hz, 1H), 4.75 (s, 1H); ^{13}C NMR (100 MHz, $CDCl_3$) δ 177.4, 147.4, 143.6, 141.6, 129.4, 129.1, 127.9, 125.2, 124.1, 123.1, 110.5, 52.2; HRMS (ESI): Exact mass calcd. for $C_{14}H_{11}N_2O_3$ $[M+H]^+$: 256.0842. Found: 256.0824.

45. 3-methyl-3-(4-nitrophenyl)indolin-2-one (**6d**)

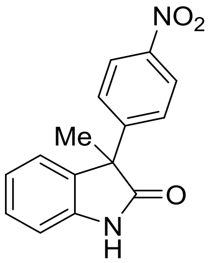

6d

Yellow solid, m.p. 185-186 °C. 42% yield. ^{1}H NMR (400 MHz, $CDCl_3$) δ 9.54 (br s, 1H), 8.15 (d, *J* = 9.2 Hz, 2H), 7.51 (d, *J* = 9.2 Hz, 2H), 7.29-7.25 (m, 1H), 7.13-7.07 (m, 2H), 7.00 (d, *J* = 7.2 Hz, 1H), 1.86 (s, 3H); ^{13}C NMR (100 MHz, $CDCl_3$) δ 181.2, 147.7, 147.0, 140.3, 134.0, 128.7, 127.8, 124.2, 123.6, 123.2, 110.7, 52.9, 23.5; HRMS (ESI): Exact mass calcd. for $C_{15}H_{11}N_2O_3$ $[M-H]^-$: 267.0785. Found: 267.0775.

46. 5-(3-methyl-2-oxoindolin-3-yl)-2-nitrobenzonitrile (**6d’**)

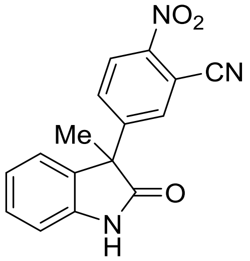

6d'

Yellow solid, m.p. 209-210 °C. 34% yield. ^{1}H NMR (600 MHz, $CDCl_3$) δ 8.27 (d, *J* = 9.0 Hz, 2H), 7.86-7.80 (m, 2H), 7.39-7.35 (m, 1H), 7.19-7.15 (m, 2H), 7.05 (d, *J* = 7.8 Hz, 1H), 1.87 (s, 3H);

^{13}C NMR (151 MHz, $CDCl_3$) δ 178.9, 148.2, 147.4, 140.0, 134.1, 132.4, 132.3, 129.5, 125.7, 124.5, 123.7, 114.9, 110.9, 108.3, 52.3, 24.1; HRMS (ESI): Exact mass calcd. for $C_{16}H_{12}N_3O_3$ $[M+H]^+$: 294.0873. Found: 294.0854.

47. 2-(4-nitrophenyl)-2-phenylacetonitrile (**6e**)

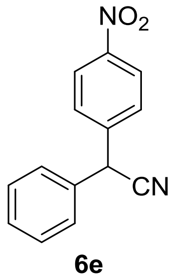

6e

Yellow solid, m.p. 69.4 °C. 54% yield. ^1H NMR (400 MHz, $CDCl_3$) δ 8.22 (d, *J* = 8.8 Hz, 2H), 7.55 (d, *J* = 8.8 Hz, 2H), 7.43-7.33 (m, 5H), 5.26 (s, 1H); ^{13}C NMR (100 MHz, $CDCl_3$) δ 147.7, 142.7, 134.4, 129.5, 128.8, 128.7, 127.7, 124.3, 118.5, 42.2; HRMS (ESI): Exact mass calcd. for $C_{14}H_{11}N_2O_2$ $[M+H]^+$: 239.0815. Found: 239.0816.

48. 5-(cyano(phenyl)methyl)-2-nitrobenzonitrile (**6e'**)

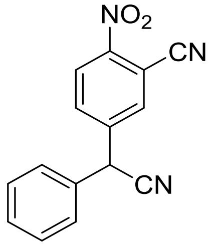

6e'

Orange oil, 59% yield. ^1H NMR (600 MHz, $CDCl_3$) δ 8.35 (d, *J* = 9.0 Hz, 1H), 7.87-7.82 (m, 2H), 7.49-7.41 (m, 3H), 7.37-7.31 (m, 2H), 5.28 (s, 1H); ^{13}C NMR (151 MHz, $CDCl_3$) δ 148.0, 143.2, 134.6, 133.1, 132.7, 130.0, 129.5, 127.7, 126.5, 117.5, 114.2, 109.1, 41.9. HRMS (CI): Exact mass calcd. for $C_{15}H_8N_3O_2$ $[M-H]^-$: 262.0622. Found: 262.0593.

49. 5-(cyano(4-fluorophenyl)methyl)-2-nitrobenzonitrile (**6f**)

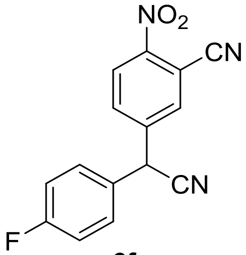

6f

Orange oil, 62% yield. ^1H NMR (600 MHz, $CDCl_3$) δ 8.36 (d, *J* = 8.4 Hz, 1H), 7.87-7.81 (m, 2H), 7.36-7.31 (m, 2H), 7.18-7.11 (m, 2H), 5.30 (s, 1H); ^{13}C NMR (151 MHz, $CDCl_3$) δ 163.0 (d, *J* = 249.9 Hz), 148.1, 143.0, 134.5, 132.7, 129.7 (d, *J* = 10.3 Hz), 129.0 (d, *J* = 3.2 Hz), 126.5, 117.3 (d,

J = 28.5 Hz), 117.0, 114.2, 109.1, 41.1. HRMS (ESI): Exact mass calcd. for $C_{15}H_9FN_3O_2$ $[M+H]^+$: 282.0673. Found: 282.0665.

50. 5-((4-chlorophenyl)(cyano)methyl)-2-nitrobenzonitrile (**6g**)

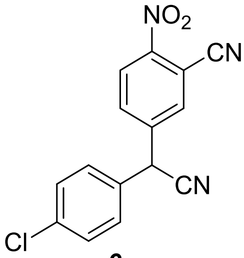

6g

Green oil, 53% yield. ^{1}H NMR (600 MHz, $CDCl_3$) δ 8.36 (d, J = 9.0 Hz, 1H), 7.87-7.80 (m, 2H), 7.43 (dd, J = 6.6, 2.4 Hz, 2H), 7.29 (dd, J = 6.6, 1.8 Hz, 2H), 5.28 (s, 1H); ^{13}C NMR (151 MHz, $CDCl_3$) δ 148.1, 142.7, 135.8, 134.5, 132.7, 131.6, 130.2, 129.1, 126.6, 117.1, 114.1, 109.2, 41.3. HRMS (ESI): Exact mass calcd. for $C_{15}H_9ClN_3O_2$ $[M+H]^+$: 298.0378. Found: 298.0357.

51. 5-((4-bromophenyl)(cyano)methyl)-2-nitrobenzonitrile (**6h**)

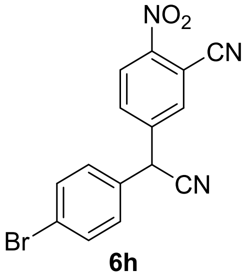

6h

Orange oil, 61% yield. ^{1}H NMR (600 MHz, $CDCl_3$) δ 8.37 (d, J = 8.4 Hz, 1H), 7.86-7.80 (m, 2H), 7.60 (dd, J = 6.6, 1.8 Hz, 2H), 7.24-7.20 (m, 2H), 5.24 (s, 1H); ^{13}C NMR (151 MHz, $CDCl_3$) δ 148.2, 142.6, 134.5, 133.2, 132.7, 132.1, 129.3, 126.6, 124.0, 117.0, 114.1, 109.3, 41.4. HRMS (CI): Exact mass calcd. for $C_{15}H_7BrN_3O_2$ $[M-H]^-$: 339.9727. Found: 339.9712.

52. 5-((4-chloro-2-fluorophenyl)(cyano)methyl)-2-nitrobenzonitrile (**6i**)

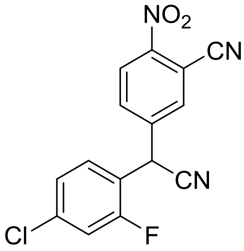

6i

Yellow oil, 32% yield. ^{1}H NMR (600 MHz, $CDCl_3$) δ 8.36 (d, J = 8.4 Hz, 1H), 7.89-7.84 (m, 2H), 7.47 (t, J = 8.4 Hz, 1H), 7.29 (dd, J = 8.4, 1.2 Hz, 1H), 7.21 (dd, J = 9.6, 1.8 Hz, 1H), 5.53 (s, 1H); ^{13}C NMR (151 MHz, $CDCl_3$) δ 159.3 (d, J = 253.2 Hz), 148.2, 141.4, 137.2 (d, J = 10.0 Hz), 134.3, 132.6, 129.8 (d, J = 3.3 Hz), 126.6, 126.2 (d, J = 3.3 Hz), 119.5 (d, J = 14.3 Hz), 117.5 (d, J =

24.2 Hz), 116.2, 114.1, 109.2, 35.6 (d, J = 3.3 Hz). HRMS (ESI): Exact mass calcd. for $C_{15}H_8ClFN_3O_2$ $[M+H]^+$: 316.0284. Found: 316.0270.

53. 5-(cyano(4-methoxyphenyl)methyl)-2-nitrobenzonitrile (**6j**)

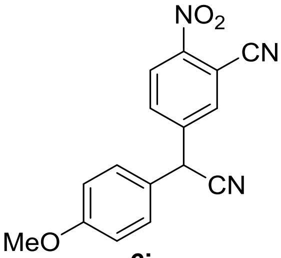

6j

Orange oil, 62% yield. ^1H NMR (400 MHz, $CDCl_3$) δ 8.26 (d, J = 9.0 Hz, 1H), 7.79-7.75 (m, 2H), 7.17 (d, J = 8.5 Hz, 2H), 6.86 (d, J = 8.5 Hz, 2H), 5.19 (s, 1H), 3.74 (s, 3H); ^{13}C NMR (100 MHz, $CDCl_3$) δ 160.2, 147.8, 143.7, 134.4, 132.6, 129.0, 126.4, 125.0, 117.8, 115.2, 114.3, 108.8, 55.4, 41.1. HRMS (ESI): Exact mass calcd. for $C_{16}H_{11}N_3NaO_3$ $[M+Na]^-$: 316.0693. Found: 316.0691.

54. 5-((4-(benzyloxy)phenyl)(cyano)methyl)-2-nitrobenzonitrile (**6k**)

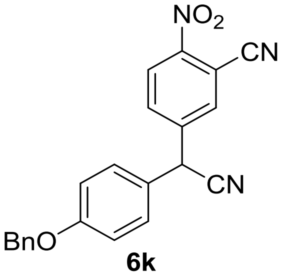

6k

Orange oil, 42% yield. ^1H NMR (600 MHz, $CDCl_3$) δ 8.34 (d, J = 13.2 Hz, 2H), 7.84 (d, J = 1.8 Hz, 1H), 7.82 (s, 1H), 7.44-7.37 (m, 4H), 7.36-7.33 (m, 1H), 7.23 (dd, J = 6.6, 1.8 Hz, 2H), 7.02 (dd, J = 6.6, 1.8 Hz, 1H), 5.22 (s, 1H), 5.08 (s, 2H); ^{13}C NMR (151 MHz, $CDCl_3$) δ 159.5, 147.9, 143.6, 136.2, 134.5, 132.6, 129.1, 128.7, 128.2, 127.4, 126.4, 125.2, 117.7, 116.2, 114.3, 109.0, 70.2, 41.2. HRMS (ESI): Exact mass calcd. for $C_{22}H_{15}N_3NaO_3$ $[M+Na]^+$: 392.1011. Found: 392.1010.

55. 5-((methylsulfonyl)(phenyl)methyl)-2-nitrobenzonitrile (**6l**)

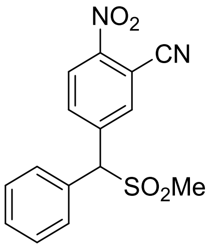

6l

Orange solid, m.p. 185-186 °C. 55% yield. ^1H NMR (600 MHz, $CDCl_3$) δ 8.34 (d, J = 8.4 Hz, 1H), 8.12-8.07 (m, 2H), 7.65 (dd, J = 7.8, 1.8 Hz, 2H), 7.54-7.48 (m, 3H), 5.47 (s, 1H), 2.85 (s, 3H);

^{13}C NMR (151 MHz, $CDCl_3$) δ 148.3, 139.2, 136.9, 135.1, 131.2, 130.2, 130.0, 129.2, 125.8, 114.5, 108.5, 73.0, 40.2. HRMS (ESI): Exact mass calcd. for $C_{15}H_{12}N_2NaO_4S$ $[M+Na]^+$: 339.0410. Found: 339.0415.

56. 2-bromo-1-((methylsulfonyl)(phenyl)methyl)-4-nitrobenzene (**6m**)

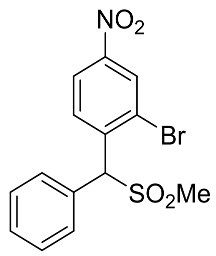

6m

Orange oil, 29% yield. ^{1}H NMR (600 MHz, $CDCl_3$) δ 8.50 (d, *J* = 2.4 Hz, 1H), 8.39 (d, *J* = 9.0 Hz, 1H), 8.26 (dd, *J* = 8.4, 2.4 Hz, 1H), 7.63 (dd, *J* = 7.8, 1.8 Hz, 2H), 7.45-7.41 (m, 3H), 6.07 (s, 1H), 2.88 (s, 3H); ^{13}C NMR (151 MHz, $CDCl_3$) δ 147.9, 139.7, 131.1, 130.8, 129.8, 129.7, 129.4, 128.4, 126.0, 122.8, 71.5, 40.3. HRMS (ESI): Exact mass calcd. for $C_{14}H_{12}BrNNaO_4S$ $[M+Na]^+$: 391.9563. Found: 391.9559.

57. 2-nitro-5-(phenyl(phenylsulfonyl)methyl)benzonitrile (**6n**)

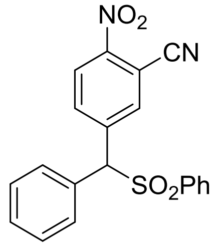

6n

Yellow solid, m.p. 176-178 °C. 33% yield. ^{1}H NMR (600 MHz, $CDCl_3$) δ 8.32 (d, *J* = 9.0 Hz, 1H), 8.13 (dd, *J* = 8.4, 1.8 Hz, 1H), 8.08 (d, *J* = 1.8 Hz, 1H), 7.66-7.62 (m, 2H), 7.60 (t, *J* = 7.2 Hz, 1H), 7.46-7.38 (m, 4H), 7.37-7.31 (m, 3H), 5.41 (s, 1H); ^{13}C NMR (151 MHz, $CDCl_3$) δ 148.0, 140.5, 137.0, 136.8, 135.0, 134.4, 130.9, 129.7, 129.3, 129.1, 129.0, 125.7, 114.5, 108.4, 74.7. HRMS (ESI): Exact mass calcd. for $C_{20}H_{14}N_2NaO_4S$ $[M+Na]^+$: 401.0566. Found: 401.0568.

58. 9-(2-nitro-5-(phenyl(pyridin-4-yl)methyl)phenyl)-9H-carbazole (**6o**)

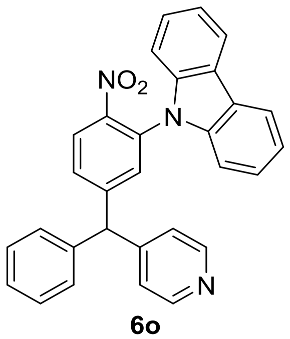

6o

Yellow solid, m.p. 173-174 °C. 55% yield. ^{1}H NMR (600 MHz, $CDCl_3$) δ 8.62 (br s, 2H), 8.14-8.07 (m, 3H), 7.44 (d, *J* = 1.8 Hz, 1H), 7.41-7.28 (m, 8H), 7.15 (d, *J* = 7.8 Hz, 2H), 7.12-7.05 (m, 4H), 5.62 (s, 1H); ^{13}C NMR (151 MHz, $CDCl_3$) δ 150.5, 150.0, 149.6, 145.2, 140.3, 140.0, 131.7, 131.2, 129.4, 129.0, 127.6, 126.22, 126.20, 126.19, 124.2, 123.7, 120.6, 120.5, 108.7, 55.5. HRMS (ESI): Exact mass calcd. for $C_{30}H_{21}N_3O_2$ $[M+H]^+$: 456.1707. Found: 456.1714.

59. 2-nitro-5-(phenyl(pyridin-4-yl)methyl)benzonitrile (**6p**)

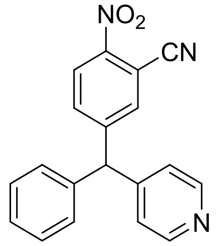

6p

Yellow solid, m.p. 156-158 °C. 57% yield. ^{1}H NMR (600 MHz, $CDCl_3$) δ 8.55 (d, *J* = 4.8 Hz, 2H), 8.25 (d, *J* = 8.4 Hz, 1H), 7.62 (d, *J* = 1.8 Hz, 1H), 7.54 (d, *J* = 8.4, 1.8 Hz, 1H), 7.38-7.33 (m, 2H), 7.32-7.28 (m, 1H), 7.08-7.04 (m, 2H), 7.02 (d, *J* = 6.0 Hz, 2H), 5.64 (s, 1H); ^{13}C NMR (151 MHz, $CDCl_3$) δ 150.2, 150.0, 149.8, 146.9, 139.2, 136.0, 134.3, 129.2, 129.0, 127.9, 125.7, 124.1, 114.7, 108.2, 55.3. HRMS (ESI): Exact mass calcd. for $C_{19}H_{14}N_3O_2$ $[M+H]^+$: 316.1081. Found: 316.1085.

Formation of S_NAr Products with *ortho-* and *para-* halogenated Nitroarene Substrates

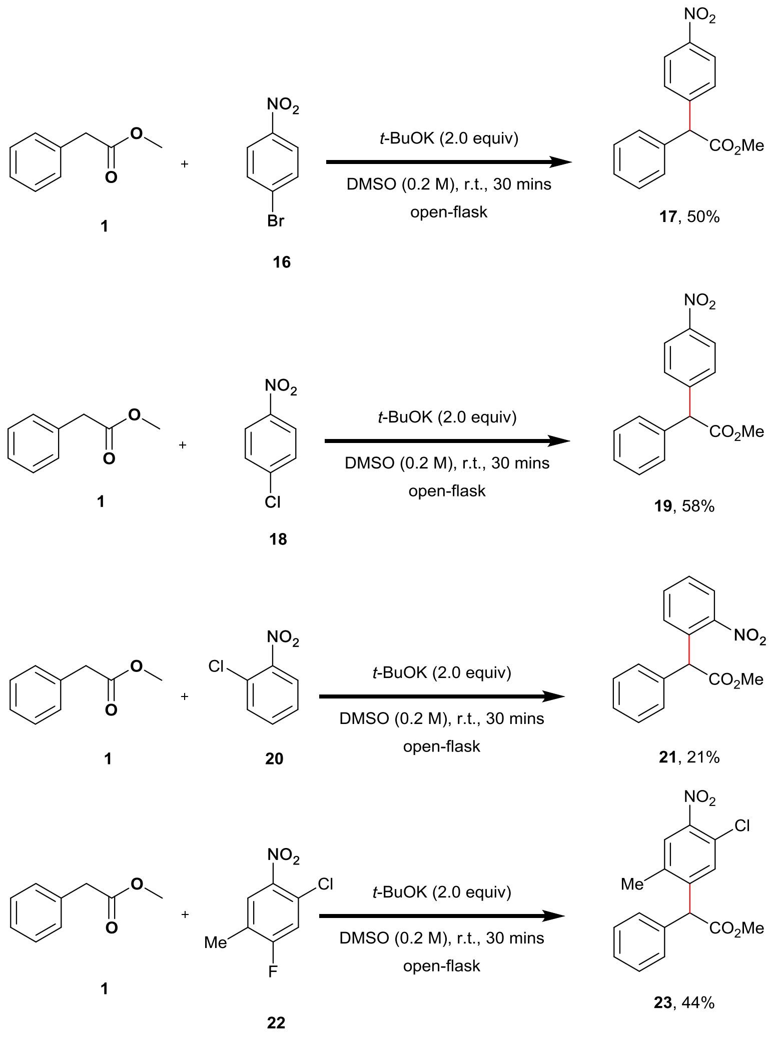

Scheme S1. Formation of S_NAr products when utilizing *ortho-* or *para-* halogenated nitroarenes as arylation substrates

Utilizing *para*-subsituted Nitroarenes as Arylation Substrates

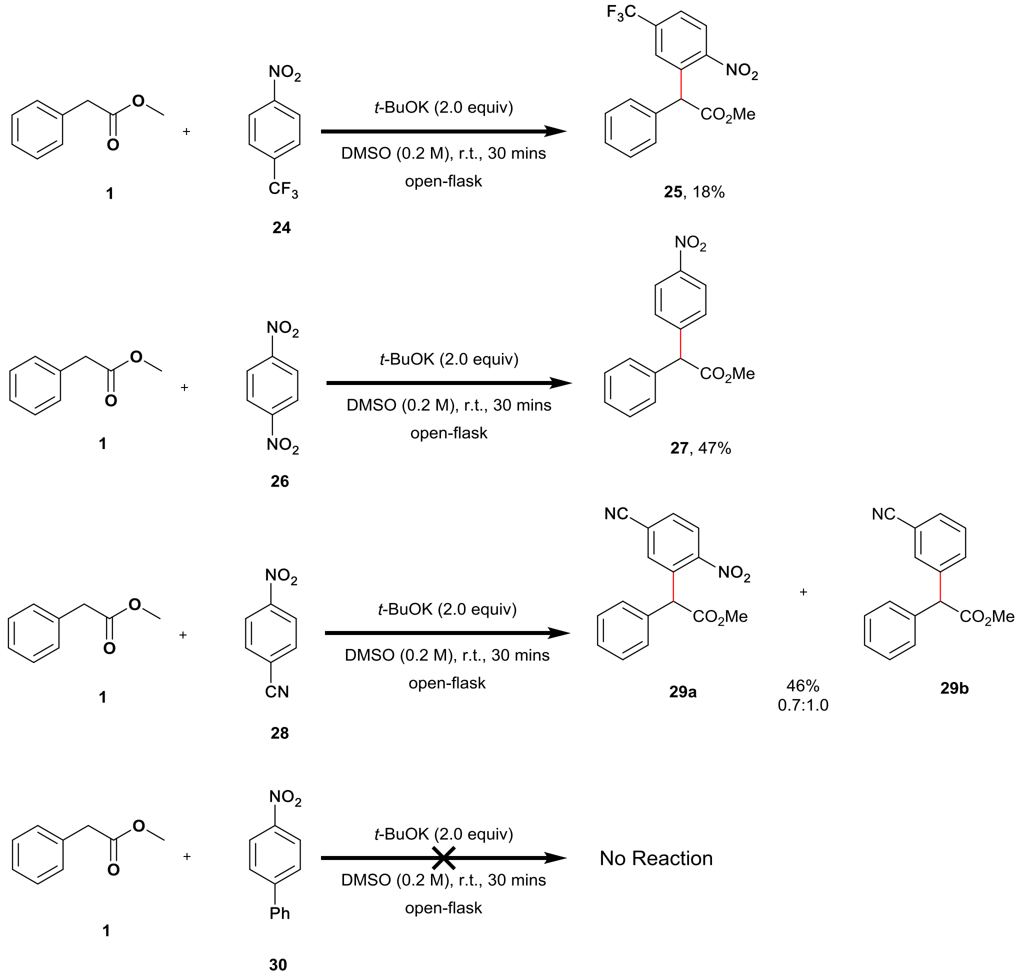

Scheme S2. Arylation of methyl 2-phenylacetate with *para*-substituted nitroarene substrates

These results indicate that when the *para*-position is occupied by another strongly electron-withdrawing group, dehydrogenative arylation at the *ortho*-position is possible, but with low yields. Additionally with these types of substrates, the nitro group can actually serve as a leaving group and an S_NAr-type reaction occurs. If the group in the *para*-position is not an electron-withdrawing group then no reaction takes place.

Utilizing other Electron Deficient Arenes (i.e. not nitroarenes) as Electrophiles

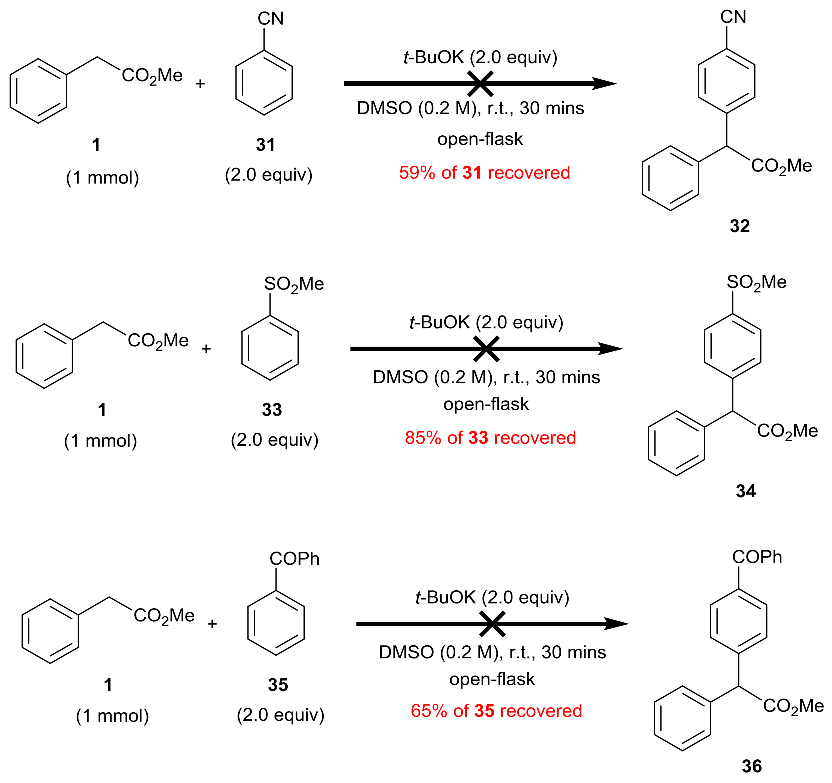

Scheme S3. α-arylation reactions conducted under standard conditions with a variety of electron deficicient arene substrates

The electron-withdrawing groups in substrates **31**, **33** and **35** are not as powerful as the nitro group. Because of this, the initial addition of the ester enolate to the aromatic ring apparently does not take place. The expected arylated products were not observed and the electron deficient arenes could be recovered.

General Procedure for the Arylation of Substrates for Reactivity Predictions

t-BuOK (224 mg, 2.0 mmol, 2.0 equiv) was added in one portion to a solution of the arylation substrates **7a-d** and **7f-i** (1.0 mmol, 1.0 equiv, (0.25 mmol, 1.0 equiv for **7e**)) and nitrobenzene **2** (246 mg, 2.0 mmol, 2.0 equiv) in dry DMSO (0.2 M) at room temperature. Once the base was added, the mixture was stirred in an open flask at room temperature for 30 minutes. The reaction was quenched via the addition of saturated NH_4Cl solution (5 mL). The resulting mixture was extracted with ethyl acetate (3×10 mL). The organic layers were combined, washed with brine (10 mL), dried over anhydrous Na_2SO_4, filtered and concentrated in *vacuo*. The crude products **8a-8c** were purified by column chromatography.

Analysis Data of pK_a Reactivity Prediction Products

60. dimethyl((4-nitrophenyl)(phenyl)methyl)phosphonate (**8a**)

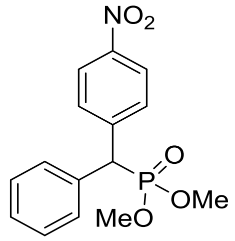

8a

Yellow oil, 7% yield. ^{1}H NMR (600 MHz, $CDCl_3$) δ 8.19 (d, *J* = 9.0 Hz, 2 H), 7.70 (d, *J* = 6.0 Hz, 2H), 7.49 (d, *J* = 7.8 Hz, 2H), 7.36 (t, *J* = 7.5 Hz, 2H), 7.30 (t, *J* = 7.5 Hz, 1H), 4.54 (d, *J* = 25.2 Hz, 1H), 3.60 (dd, *J* = 36.0, 10.8 Hz, 6H); ^{13}C NMR (151 MHz, $CDCl_3$) δ 147.2 (d, *J* = 2.5 Hz), 144.3 (d, *J* = 5.1 Hz), 135.2 (d, *J* = 5.7 Hz), 130.4 (d, *J* = 7.8 Hz, 2C), 129.5 (d, *J* = 7.9 Hz 2C), 129.2 (2C), 128.0, 123.9 (2C), 53.7 (dd, *J* = 89.1, 7.2 Hz, 2C), 50.8; HRMS (ESI): Exact mass calcd. for $C_{15}H_{17}NO_5P$ $[M+H]^+$: 322.0844. Found: 322.0844. Product purified by column chromatography (Hexanes:Ethyl Acetate = 1:1).

61. 9-(4-nitrophenyl)-9*H*-fluorene (**8b**)

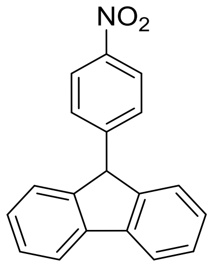

8b

Yellow solid, m.p. 123.1-124.4 °C. 26% yield. ^{1}H NMR (600 MHz, $CDCl_3$) δ 8.13 (d, *J* = 9.0 Hz, 2H), 7.83 (d, *J* = 7.8 Hz, 2H), 7.43 (t, *J* = 7.2 Hz, 2H), 7.29-7.23 (m, 6H), 5.15 (s, 1H); ^{13}C NMR (151 MHz, $CDCl_3$) δ 149.8, 147.1, 146.6 (2C), 141.2 (2C), 129.2 (2C), 128.1 (2C), 127.8 (2C), 125.3 (2C), 124.1 (2C), 120.3 (2C), 54.0; HRMS (ESI): Exact mass calcd. for $C_{19}H_{12}NO_2$ $[M^-]$: 286.0874. Found: 286.0866. Product purified by column chromatography (Hexanes:Ethyl Acetate = 20:1).

62. 1-nitro-4-(1-nitroethyl)benzene (**8c**)

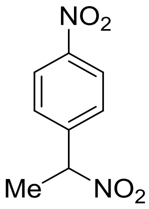

8c

Yellow oil, 86% yield. ^{1}H NMR (600 MHz, $CDCl_3$) δ 8.26 (d, *J* = 9.0 Hz, 2H), 7.65 (d, *J* = 9.0 Hz, 2H), 5.71 (q, *J* = 6.0 Hz, 1H), 1.94 (d, *J* = 7.2 Hz, 3H); ^{13}C NMR (151 MHz, $CDCl_3$) δ 148.6, 141.7, 128.6 (2C), 124.2 (2C), 85.1, 19.5; HRMS (ESI): Exact mass calcd. for $C_8H_7N_2O_4$ $[M-H]^-$: 195.0411. Found: 195.0423. Product purified by column chromatography (Hexanes:Ethyl Acetate = 10:1).

General Two-step Preparation of All Carbon Quaternary Center Containing Compounds

Step 1: *t*-BuOK (1.2 g, 10.9 mmol, 1.09 equiv) was suspended in 8 mL of dry DMF under an argon atmosphere. At 0 °C methyl phenylacetate (1.5 g, 10 mmol, 1.0 equiv) was added followed by benzyl bromide (1.71 g, 10 mmol, 1.0 equiv) after 5 minutes. The reaction was warmed to room temperature and stirred for 1 h. Water (12 mL) was added and the solution was extracted with DCM (2 ×12 mL). The organic layers were combined and washed with saturated NH_4CL (12 mL) and water (12 mL). The combined organic layers were died over $MgSO_4$, filtered and concentrated in *vacuo*. The crude product was purified by column chromatography (Hexanes:Ethyl Acetate = 10:1) [3] to give methyl 2,3-diphenylpropanoate **9** (941 mg, 39% yield) as a colorless oil.

Step 2: *t*-BuOK (224 mg, 2.0 mmol, 2.0 equiv) was added in one portion to the solution of **9** (240 mg, 1.0 mmol, 1.0 equiv) and 2-nitro benzonitrile **2a** (296 mg, 2.0 mmol, 2.0 equiv) in dry DMSO (0.2 M) at room temperature. Once the base was added the mixture was stirred in an open flask at room temperature for 30 minutes, followed by the addition of saturated NH_4Cl solution (5 mL). The resulting mixture was extracted with ethyl acetate (3×10 mL). The organic layers were combined, washed with brine (10 mL), dried over anhydrous Na_2SO_4, filtered and concentrated in *vacuo*. The crude product was purified by column chromatography (Hexanes:Ethyl Acetate = 10:1) to give methyl 2-(3-cyano-4-nitrophenyl)-2,3-diphenylpropanoate **10** (67.4 mg, 18% yield) as a yellow oil.

Analysis Data for All Carbon Quaternary Center Containing Compounds Prepared in Two Steps

63. methyl 2,3-diphenylpropanoate (**9**)

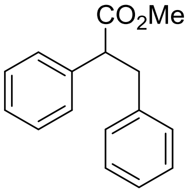

9

Colorless oil, 39% yield. ^{1}H NMR (600 MHz, $CDCl_3$) δ 7.44-7.39 (m, 4H), 7.36-7.32 (m, 3H), 7.29-7.27 (m, 1H), 7.24-7.23 (m, 2H), 3.98 (dd, *J* = 8.8, 6.7 Hz, 1H), 3.67 (s, 3H), 3.55 (dd, *J* = 13.7, 8.8 Hz, 1H), 3.15 (dd, *J* = 13.8, 6.7 Hz, 1H); ^{13}C NMR (151 MHz, $CDCl_3$) δ 173.7, 139.0, 138.6, 128.9 (2C), 128.6 (2C), 128.3 (2C), 127.9 (2C), 127.4, 126.3, 53.6, 51.9, 39.8; These shifts are consistant with literature reported values. HRMS (ESI): Exact mass calcd. for $C_{16}H_{17}O_2$ $[M+H]^+$: 241.1223. Found: 241.1224.

64. methyl 2-(3-cyano-4-nitrophenyl)-2,3-diphenylpropanoate (**10**)

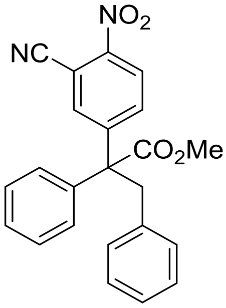

10

Yellow oil, 18% yield. ^{1}H NMR (600 MHz, $CDCl_3$) δ 8.04 (d, *J* = 8.8 Hz, 1H), 7.43-7.39 (m, 3H), 7.36 (d, *J* = 2.1 Hz, 1H), 7.34 (dd, *J* = 7.6, 2.1 Hz, 2H), 7.28 (dd, *J* = 8.8, 2.2 Hz, 1H), 7.21 (t, *J* = 7.5 Hz, 1H), 7.14 (t, *J* = 7.6, 2H), 6.64 (d, *J* = 7.2 Hz, 2H), 4.23 (d, *J* = 13.0 Hz, 1H), 3.78 (s, 3H), 2.28 (d, *J* = 13.0 Hz, 1H) ; ^{13}C NMR (151 MHz, $CDCl_3$) δ 172.4, 150.4, 146.6, 140.89, 137.1, 135.5, 135.2, 130.3 (2C), 129.1 (2C), 128.6, 128.5 (2C), 128.0 (2C), 127.6, 124.1, 115.1, 106.7, 62.0, 53.0, 44.1; HRMS (ESI): Exact mass calcd. for $C_{23}H_{19}N_2O_4$ $[M+H]^+$: 387.1339. Found: 387.1338.

General One-pot Preparation of all Carbon Quaternary Center Containing Compounds

t-BuOK (448 mg, 4.0 mmol, 2.0 equiv) was added in one portion to a solution of methyl phenylacetate **1** (300 mg, 2.0 mmol) and nitrobenzene **2** (492 mg, 4.0 mmol, 2.0 equiv) in dry DMSO (10 mL) at room temperature. Once the base was added completely, the mixture was stirred

in an open flask at room temperature for 30 minutes. After 30 minutes allylic bromide (480 mg, 4.0 mmol, 2.0 equiv) was added. The reaction mixture was stirred under N_2 at room temperature for 12 hours. The reaction was quenched via the addition of saturated NH_4Cl solution (10 mL). The resulting mixture was extracted with ethyl acetate (3×20 mL). The organic layers were combined and washed with brine (20 mL), dried over anhydrous Na_2SO_4, filtered and concentrated in *vacuo*. The crude product was purified by column chromatography (Hexanes:Ethyl Acetate = 10:1) to give methyl 2-(4-nitrophenyl)-2-phenylpent-4-enoate **12a** (275.8 mg, 44% yield) as a yellow oil.

Analysis Data of all Carbon Quaternary Center Containing Compounds Prepared via One-pot Method

65. methyl 2-(4-nitrophenyl)-2-phenylpent-4-enoate (**12a**)

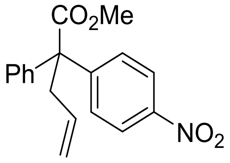

12a

Yellow oil. 44% yield. ^1H NMR (400 MHz, $CDCl_3$) δ 8.16-8.10 (m, 2H), 7.42-7.25 (m, 7H), 5.64-5.51 (m, 1H), 5.00-4.89 (m, 2H), 3.73 (s, 3H), 3.29 (dd, *J* = 14.0, 6.8 Hz, 1H), 3.10 (dd, *J* = 14.0, 7.2 Hz, 1H); ^{13}C NMR (100 MHz, $CDCl_3$) δ 173.3, 150.0, 146.5, 141.2, 133.0, 130.2, 128.44, 128.37, 127.6, 122.8, 119.3, 60.4, 52.7, 42.5. HRMS (ESI): Exact mass calcd. for $C_{18}H_{18}NO_4$ $[M+H]^+$: 312.1230. Found: 312.1232.

66. methyl 2-(2-bromo-4-nitrophenyl)-2-phenylpent-4-enoate (**12b**)

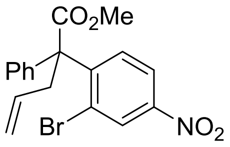

12b

Yellow oil. 48% yield. ^1H NMR (400 MHz, $CDCl_3$) δ 8.44 (d, *J* = 2.0 Hz, 1H), 8.05 (dd, *J* = 8.8, 2.4 Hz, 1H), 7.54-7.50 (m, 2H), 7.40-7.29 (m, 3H), 7.27 (d, *J* = 8.8 Hz, 1H), 5.60-5.47 (m, 1H), 5.05-4.90 (m, 2H), 3.70 (s, 3H), 3.52 (dd, *J* = 15.2, 6.4 Hz, 1H), 3.36 (dd, *J* = 15.2, 6.8 Hz, 1H); ^{13}C NMR (100 MHz, $CDCl_3$) δ 172.6, 149.2, 146.7, 138.9, 132.8, 132.1, 129.3, 129.0, 128.4, 127.8, 124.8, 121.2, 119.0, 60.9, 52.7, 39.2. HRMS (ESI): Exact mass calcd. for $C_{18}H_{17}BrNO_4$ $[M+H]^+$: 390.0335. Found: 390.0327.

67. methyl 2-(2-fluoro-4-nitrophenyl)-2-phenylpent-4-enoate (**12c**)

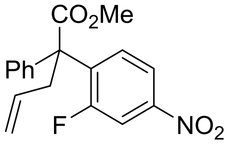

12c

Yellow oil. 61% yield. ^{1}H NMR (300 MHz, $CDCl_3$) δ 7.91-7.86 (m, 2H), 7.50-7.45 (m, 2H), 7.42-7.31 (m, 3H), 7.19-7.11 (m, 1H), 5.69-5.57 (m, 1H), 4.98-4.89 (m, 2H), 3.72 (s, 3H), 3.31-3.25 (m, 2H); ^{13}C NMR (755 MHz, $CDCl_3$) δ 172.5, 160.2 (d, *J* = 250.4 Hz), 147.6 (d, *J* = 9.2 Hz), 138.39, 138.36 (d, *J* = 21.2 Hz), 133.0, 131.1 (d, *J* = 4.4 Hz), 128.5 (d, *J* = 3.2 Hz), 127.9, 121.1, 119.0, 118.4 (d, *J* = 3.3 Hz), 111.5 (d, *J* = 28.4 Hz), 57.4 (d, *J* = 2.2 Hz), 52.7, 39.9 (d, *J* = 2.2 Hz). HRMS (ESI): Exact mass calcd. for $C_{18}H_{17}FNO_4$ $[M+H]^+$: 330.1136. Found: 330.1126.

68. methyl 2-(3-cyano-4-nitrophenyl)-2-phenylpent-4-enoate (**12d**)

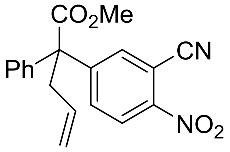

12d

Yellow oil. 46% yield. ^{1}H NMR (400 MHz, $CDCl_3$) δ 8.21 (d, *J* = 8.8 Hz, 1H), 7.72 (d, *J* = 2.0 Hz, 1H), 7.62 (dd, *J* = 8.8, 1.6 Hz, 1H), 7.43-7.34 (m, 3H), 7.28-7.23 (m, 2H), 5.62-5.52 (m, 1H), 5.04 (d, *J* = 10.0 Hz, 1H), 4.93 (dd, *J* = 16.8, 1.2 Hz, 1H), 3.75 (s, 3H), 3.37 (dd, *J* = 14.8, 7.2 Hz, 1H), 3.01 (dd, *J* = 14.0, 7.2 Hz, 1H); ^{13}C NMR (100 MHz, $CDCl_3$) δ 172.5, 150.6, 146.7, 140.2, 136.5, 134.6, 132.2, 128.9, 128.2, 128.0, 124.6, 120.3, 115.1, 107.2, 60.2, 53.0, 42.4. HRMS (ESI): Exact mass calcd. for $C_{19}H_{17}N_2O4$ $[M+H]^+$: 337.1183. Found: 337.1174.

69. methyl 2-(3-cyano-4-nitrophenyl)-2-(4-methoxyphenyl)pent-4-enoate (**12e**)

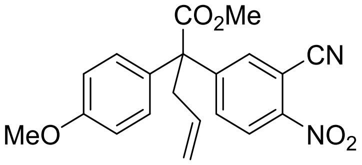

12e

Yellow oil. 48% yield. ^{1}H NMR (500 MHz, $CDCl_3$) δ 8.18 (d, *J* = 8.5 Hz, 1H), 7.69 (d, *J* = 2.0 Hz, 1H), 7.61 (dd, *J* = 9.0, 2.0 Hz, 1H), 7.21-7.13 (m, 2H), 6.90-6.82 (m, 2H), 5.60-5.50 (m, 1H), 4.99 (dd, *J* = 10.5, 1.0 Hz, 1H), 4.88 (dd, *J* = 17.0, 1.5 Hz, 1H), 3.77 (s, 3H), 3.71 (s, 3H), 3.33 (dd, *J* = 13.5, 6.5 Hz, 1H), 2.95 (dd, *J* = 14.0, 7.5 Hz, 1H); ^{13}C NMR (125 MHz, $CDCl_3$) δ 172.5, 159.0, 150.8, 146.3, 136.2, 134.4, 132.2, 131.8, 129.0, 124.4, 119.9, 115.0, 114.0, 106.8, 59.3, 55.0, 52.7, 42.2. HRMS (ESI): Exact mass calcd. for $C_{20}H_{19}N_2O_5$ $[M+H]^+$: 367.1288. Found: 367.1296.

70. 2-(4-nitrophenyl)-2-phenylpent-4-enenitrile (**12f**)

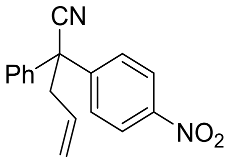

12f

Yellow oil. 61% yield. ^{1}H NMR (400 MHz, $CDCl_3$) δ 8.21 (dd, *J* = 8.8, 2.8 Hz, 2H), 7.61-7.55 (m, 2H), 7.44-7.32 (m, 5H), 5.75-5.64 (m, 1H), 5.25-5.18 (m, 2H), 3.22 (dd, *J* = 14.0, 6.8 Hz, 1H), 3.13 (dd, *J* = 14.0, 7.2 Hz, 1H); ^{13}C NMR (100 MHz, $CDCl_3$) δ 147.4, 146.7, 138.2, 130.7, 129.2, 128.6, 128.1, 126.9, 124.0, 121.3, 120.8, 51.6, 43.6. HRMS (ESI): Exact mass calcd. for $C_{17}H_{15}N_2O_2$ $[M+H]^+$: 279.1128. Found: 279.1125.

71. 2-(2-fluoro-4-nitrophenyl)-2-(4-methoxyphenyl)pent-4-enenitrile (**12g**)

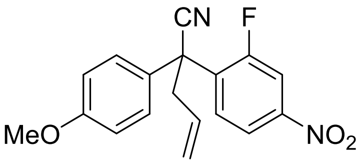

12g

Yellow oil. 54% yield. ^{1}H NMR (500 MHz, $CDCl_3$) δ 7.99-7.94 (m, 1H), 7.76 (dd, *J* = 11.0, 2.5 Hz, 1H), 7.67 (t, *J* = 9.0 Hz, 1H), 7.18-7.12 (m, 2H), 6.80-6.74 (m, 2H), 5.64-5.55 (m, 1H), 5.17-5.04 (m, 2H), 3.67 (s, 3H), 3.19 (dd, *J* = 14.0, 9.0 Hz, 1H), 3.08 (dd, *J* = 14.0, 6.5 Hz, 1H); ^{13}C NMR (125 MHz, $CDCl_3$) δ 159.39 (d, *J* = 254.6 Hz), 159.38, 148.5 (d, *J* = 9.0 Hz), 134.1 (d, *J* = 5.4 Hz), 130.6, 129.3 (d, *J* = 3.6 Hz), 128.8, 127.7, 121.0, 119.8, 119.2 (d, *J* = 3.6 Hz), 114.2, 112.4 (d, *J* = 27.0 Hz), 55.1, 48.3 (d, *J* = 1.8 Hz), 41.6 (d, *J* = 2.8 Hz). HRMS (ESI): Exact mass calcd. for $C_{18}H_{16}FN_2O_3$ $[M+H]^+$: 327.1139. Found: 327.1136.

72. 2-(2-bromo-4-nitrophenyl)-2-(4-fluorophenyl)pent-4-enenitrile (**12h**)

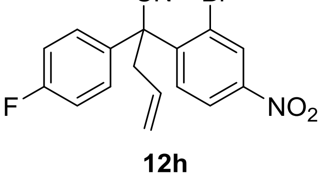

12h

Yellow oil. 35% yield. ^{1}H NMR (300 MHz, $CDCl_3$) δ 8.45 (d, *J* = 2.4 Hz, 1H), 8.30 (dd, *J* = 8.7, 2.4 Hz, 1H), 7.96 (d, *J* = 8.7 Hz, 1H), 7.28-7.16 (m, 2H), 7.09-7.00 (m, 2H), 5.75-5.61 (m, 1H), 5.25-5.16 (m, 2H), 3.46 (dd, *J* = 13.5, 7.2 Hz, 1H), 3.11 (dd, *J* = 13.5, 6.9 Hz, 1H); ^{13}C NMR (75 MHz, $CDCl_3$) δ 162.2 (d, *J* = 247.7 Hz), 147.9, 143.6, 133.2 (d, *J* = 3.8 Hz), 130.6, 130.2 (d, *J* = 15.8

Hz), 128.9 (d, J = 8.7 Hz), 124.5, 122.2, 121.6, 119.0, 116.0, 115.7, 51.4, 43.7. HRMS (ESI): Exact mass calcd. for $C_{17}H_{13}BrFN_2O_2$ $[M+H]^+$: 375.0139. Found: 375.0156.

73. 2-(2-bromo-4-nitrophenyl)-2-(4-bromophenyl)pent-4-enenitrile (**12i**)

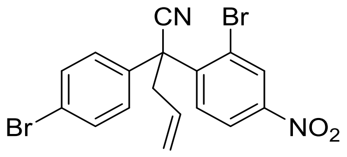

12i

Yellow oil. 37% yield. ^{1}H NMR (300 MHz, $CDCl_3$) δ 8.44 (d, J = 2.1 Hz, 1H), 8.30 (dd, J = 8.4, 2.4 Hz, 1H), 7.95 (d, J = 9.0 Hz, 1H), 7.51-7.44 (m, 2H), 7.14-7.05 (m, 2H), 5.77-5.60 (m, 1H), 5.25-5.17 (m, 2H), 3.44 (dd, J = 13.5, 7.5 Hz, 1H), 3.10 (dd, J = 13.5, 6.9 Hz, 1H); ^{13}C NMR (75 MHz, $CDCl_3$) δ 147.8, 143.2, 136.4, 131.9, 130.5, 130.1, 130.0, 128.7, 124.5, 122.3, 122.2, 121.7, 118.6, 51.5, 43.6. HRMS (ESI): Exact mass calcd. for $C_{17}H_{12}Br_2N_2NaO_2$ $[M+H]^+$: 456.9158. Found: 456.9171.

74. 2-(2-bromo-4-nitrophenyl)-2-(4-methoxyphenyl)-4-methylpent-4-enenitrile (**12j**)

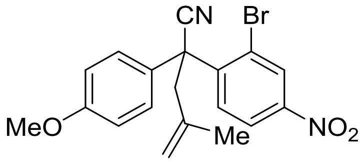

12j

Yellow oil. 46% yield. ^{1}H NMR (500 MHz, $CDCl_3$) δ 8.36 (d, J = 2.5 Hz, 1H), 8.19 (dd, J = 8.5, 2.5 Hz, 1H), 7.92 (d, J = 9.0 Hz, 1H), 7.05 (dd, J = 6.5, 2.0 Hz, 2H), 6.79 (dd, J = 6.5, 2.0 Hz, 2H), 4.87 (t, J = 1.5 Hz, 1H), 4.75 (s, 1H), 3.74 (s, 3H), 3.46 (d, J = 14.0 Hz, 1H), 3.03 (d, J = 13.5 Hz, 1H), 1.47 (s, 3H); ^{13}C NMR (125 MHz, $CDCl_3$) δ 159.4, 147.7, 144.3, 138.5, 130.70, 130.67, 129.8, 128.4, 124.1, 122.0, 120.3, 118.0, 114.3, 55.3, 51.1, 45.2, 23.6. HRMS (ESI): Exact mass calcd. for $C_{19}H_{18}BrN_2O_3$ $[M+H]^+$: 401.0495. Found: 401.0491.

75. methyl 2-(2-bromo-4-nitrophenyl)-4-methyl-2-phenylpent-4-enoate (**12k**)

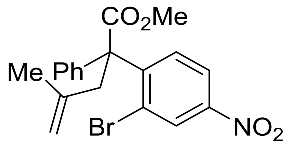

12k

Yellow oil. 38% yield. ^{1}H NMR (500 MHz, $CDCl_3$) δ 8.40 (d, J = 2.5 Hz, 1H), 8.13 (dd, J = 9.0, 2.5 Hz, 1H), 7.66 (d, J = 8.5 Hz, 1H), 7.58-7.53 (m, 2H), 7.32-7.26 (m, 3H), 4.71 (t, J = 1.5 Hz, 1H), 4.48 (s, 1H), 3.69 (s, 3H), 3.44 (d, J = 14.0 Hz, 1H), 3.38 (d, J = 14.5 Hz, 1H), 1.43 (d, J = 5.5 Hz, 3H); ^{13}C NMR (125 MHz, $CDCl_3$) δ 172.2, 149.4, 146.6, 140.8, 139.6, 131.5, 129.6, 129.0,

128.1, 127.5, 125.2, 121.2, 116.3, 61.1, 52.6, 44.1, 24.2. HRMS (ESI): Exact mass calcd. for $C_{19}H_{19}BrNO_4$ [M+H]$^+$: 404.0492. Found: 404.0489.

76. methyl (E)-2-(2-bromo-4-nitrophenyl)-2-phenylhex-4-enoate (**12l**)

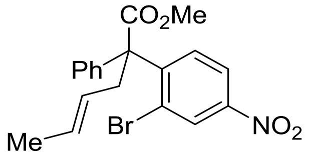

12l

Yellow oil. 54% yield. ^{1}H NMR (300 MHz, $CDCl_3$) δ 8.44 (d, *J* = 2.4 Hz, 1H), 8.05 (dd, *J* = 9.0, 2.4 Hz, 1H), 7.54-7.48 (m, 2H), 7.40-7.26 (m, 4H), 5.45-5.31 (m, 1H), 5.26-5.16 (m, 1H), 3.69 (s, 3H), 3.45-3.36 (m, 1H), 3.35-3.23 (m, 1H), 1.47 (dd, *J* = 6.3, 1.2 Hz, 3H); ^{13}C NMR (75 MHz, $CDCl_3$) δ 172.7, 149.5, 146.8, 139.2, 132.1, 129.8, 129.3, 129.0, 128.3, 127.7, 125.1, 124.9, 121.1, 61.2, 52.7, 38.4, 17.9. HRMS (ESI): Exact mass calcd. for $C_{19}H_{19}BrNO_4$ [M+H]$^+$: 404.0492. Found: 404.0503.

77. methyl 2-(4-nitrophenyl)-2-phenylpent-4-ynoate (**12m**)

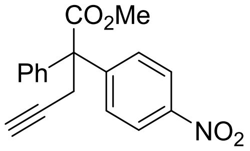

12m

Yellow oil. 39% yield. ^{1}H NMR (400 MHz, $CDCl_3$) δ 8.19-8.12 (m, 2H), 7.53-7.45 (m, 2H), 7.40-7.26 (m, 5H), 3.76 (s, 3H), 3.37 (dd, *J* = 16.8, 2.8 Hz, 1H), 3.25 (dd, *J* = 16.8, 2.8 Hz, 1H), 1.96 (t, *J* = 2.8 Hz, 1H); ^{13}C NMR (100 MHz, $CDCl_3$) δ 172.7, 148.8, 146.9, 140.0, 130.2, 128.4, 128.3, 127.9, 122.8, 79.9, 72.7, 59.9, 53.0, 29.0. HRMS (ESI): Exact mass calcd. for $C_{18}H_{16}NO_4$ [M+H]$^+$: 310.1074. Found: 310.1064.

78. methyl 2-(4-nitrophenyl)-2-phenylhex-4-ynoate (**12n**)

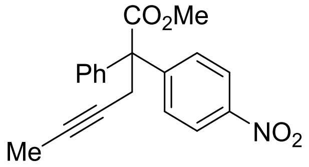

12n

Yellow oil. 49% yield. ^{1}H NMR (400 MHz, $CDCl_3$) δ 8.05-7.98 (m, 2H), 7.39-7.31 (m, 2H), 7.28-7.15 (m, 5H), 3.62 (s, 2H), 3.20 (dd, *J* = 16.8, 2.0 Hz, 1H), 3.07 (dd, *J* = 16.0, 2.0 Hz, 1H), 1.52 (t, *J* = 2.0 Hz, 3H); ^{13}C NMR (100 MHz, $CDCl_3$) δ 172.8, 149.2, 146.6, 140.4, 130.1, 128.14, 128.12, 127.5, 122.4, 79.9, 74.5, 60.1, 52.7, 29.1, 3.2. HRMS (ESI): Exact mass calcd. for $C_{19}H_{18}NO_4$ [M+H]$^+$: 324.1230. Found: 324.1240.

79. methyl 2-(2-fluoro-4-nitrophenyl)-2-(4-methoxyphenyl)hex-4-ynoate (**12o**)

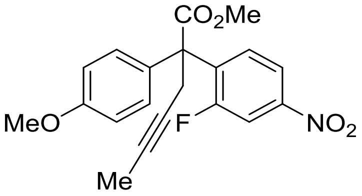

12o

Yellow oil. 36% yield. ^{1}H NMR (400 MHz, $CDCl_3$) δ 7.96-7.87 (m, 2H), 7.46 (d, *J* = 8.8 Hz, 2H), 7.12 (t, *J* = 8.0 Hz, 1H), 6.93 (d, *J* = 8.8 Hz, 2H), 3.79 (s, 3H), 3.60 (s, 3H), 3.45 (dd, *J* = 16.8, 2.0 Hz, 1H), 3.16 (dd, *J* = 17.6, 2.8 Hz, 1H), 1.55 (t, *J* = 2.4 Hz, 3H); ^{13}C NMR (100 MHz, $CDCl_3$) δ 172.5, 159.1 (d, *J* = 32.0 Hz), 147.7, 138.4 (d, *J* = 12.4 Hz), 132.1 (d, *J* = 4.3 Hz), 130.3, 129.8, 129.1, 118.0 (d, *J* = 3.6 Hz), 113.9, 111.3 (d, *J* = 28.4 Hz), 80.0, 74.5, 56.4 (d, *J* = 1.4 Hz), 55.3, 52.9, 26.2 (d, *J* = 2.2 Hz), 3.3. HRMS (ESI): Exact mass calcd. for $C_{20}H_{19}FNO_5$ $[M+H]^+$: 372.1242. Found: 372.1237.

80. methyl 2-(4-chlorophenyl)-2-(2-fluoro-4-nitrophenyl)hex-4-ynoate (**12p**)

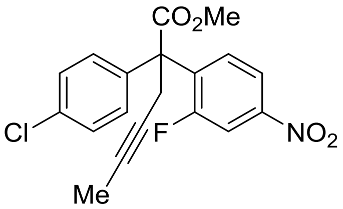

12p

Yellow oil. 57% yield. ^{1}H NMR (400 MHz, $CDCl_3$) δ 7.98-7.89 (m, 2H), 7.49 (d, *J* = 8.8 Hz, 2H), 7.37 (d, *J* = 8.4 Hz, 2H), 7.15-7.06 (m, 1H), 3.73 (s, 3H), 3.44 (dd, *J* = 16.8, 2.0 Hz, 1H), 3.16 (dd, *J* = 17.6, 2.0 Hz, 1H), 1.55 (t, *J* = 2.4 Hz, 3H); ^{13}C NMR (100 MHz, $CDCl_3$) δ 171.9, 160.2 (d, *J* = 251.0 Hz), 148.0 (d, *J* = 8.7 Hz), 137.6 (d, *J* = 12.4 Hz), 136.0, 134.3, 131.9 (d, *J* = 3.6 Hz), 130.1, 128.7, 118.2 (d, *J* = 3.7 Hz), 111.5 (d, *J* = 28.3 Hz), 80.4, 74.0, 56.7 (d, *J* = 1.4 Hz), 53.1, 26.3 (d, *J* = 2.2 Hz), 3.3. HRMS (ESI): Exact mass calcd. for $C_{17}H_{16}ClFNO_4$ $[M+H]^+$: 376.0746. Found: 376.0727.

81. methyl 2-(2-chlorophenyl)-2-(2-fluoro-4-nitrophenyl)hex-4-ynoate (**12q**)

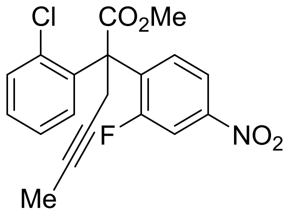

12q

Yellow oil. 57% yield. ^{1}H NMR (400 MHz, $CDCl_3$) δ 7.97 (dd, *J* = 8.8, 2.4 Hz, 1H), 7.87 (dd, *J* = 10.8, 2.4 Hz, 1H), 7.68 (t, *J* = 8.8 Hz, 1H), 7.59 (d, *J* = 7.2 Hz, 1H), 7.40-7.27 (m, 3H), 3.76 (s,

3H), 3.56-3.40 (m, 2H), 1.60 (t, J = 2.0 Hz, 3H); ^{13}C NMR (100 MHz, $CDCl_3$) δ 171.1, 160.5 (d, J = 253.8 Hz), 148.0 (d, J = 9.5 Hz), 137.0, 134.3 (d, J = 10.2 Hz), 133.7, 132.0 (d, J = 3.6 Hz), 131.2, 130.2 (d, J = 2.2 Hz), 129.2, 126.8, 118.2 (d, J = 3.7 Hz), 112.0 (d, J = 29.1 Hz), 80.4, 74.1, 58.4 (d, J = 2.9 Hz), 53.1, 27.6 (d, J = 4.4 Hz), 3.4. HRMS (ESI): Exact mass calcd. for $C_{19}H_{16}ClFNO_4$ $[M+H]^+$: 376.0746. Found: 376.0738.

82. 2-(2-bromo-4-nitrophenyl)-2-(4-bromophenyl)hex-4-ynenitrile (**12r**)

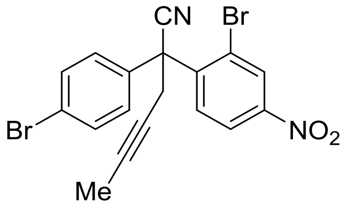

12r

Yellow oil. 53% yield. ^{1}H NMR (300 MHz, $CDCl_3$) δ 8.45 (d, J = 2.4 Hz, 1H), 8.31 (dd, J = 8.7, 2.4 Hz, 1H), 8.03 (d, J = 8.7 Hz, 1H), 7.52-7.43 (m, 2H), 7.14-7.06 (m, 2H), 3.40-3.20 (m, 2H),1.74 (t, J = 2.4 Hz, 3H); ^{13}C NMR (75 MHz, $CDCl_3$) δ 148.0, 142.2, 136.5, 132.0, 130.42, 130.36, 128.4, 124.8, 122.6, 122.1, 118.5, 82.7, 72.0, 51.8, 32.2, 3.4. HRMS (ESI): Exact mass calcd. for $C_{18}H_{13}Br_2N_2O_2$ $[M+H]^+$: 446.9338. Found: 446.9332.

83. 2-(2-bromo-4-nitrophenyl)-2-(4-methoxyphenyl)hex-4-ynenitrile (**12s**)

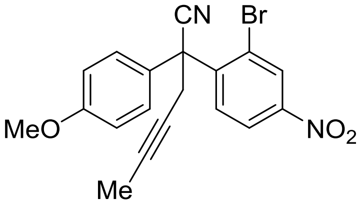

12s

Pale yellow solid, m.p. 127-129 °C. 66% yield. ^{1}H NMR (400 MHz, $CDCl_3$) δ 8.47(d, J = 2.4 Hz, 1H), 8.31 (dd, J = 8.8, 2.4 Hz, 1H), 8.02 (d, J = 8.8 Hz, 1H), 7.16-7.09 (m, 2H), 6.90-6.82 (m, 2H), 3.81 (s, 3H), 3.37-3.20 (m, 2H),1.75 (t, J = 2.4 Hz, 3H); ^{13}C NMR (100 MHz, $CDCl_3$) δ 159.5, 147.8, 143.1, 130.5, 130.4, 129.3, 128.0, 124.8, 122.0, 119.2, 114.3, 82.2, 72.5, 55.3, 51.8, 32.2, 3.5. HRMS (ESI): Exact mass calcd. for $C_{19}H_{15}BrN_2NaO_3$ $[M+Na]^+$: 421.0158. Found: 421.0150.

84. methyl 2-(2-fluoro-4-nitrophenyl)-2,3-diphenylpropanoate (**12t**)

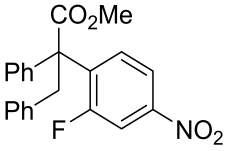

12t

Yellow oil. 66% yield. ^{1}H NMR (400 MHz, $CDCl_3$) δ 7.82 (dd, *J* = 8.8, 2.0 Hz, 1H), 7.71 (dd, *J* = 11.2, 2.4 Hz, 1H), 7.54-7.47 (m, 2H), 7.40-7.31 (m, 3H), 7.19 (t, *J* = 7.2 Hz, 1H), 7.14-7.00 (m, 3H), 6.80 (d, *J* = 6.4 Hz, 2H), 4.19 (d, *J* = 13.2 Hz, 1H), 3.69 (s, 3H), 3.47 (d, *J* = 14.0 Hz, 1H); ^{13}C NMR (100 MHz, $CDCl_3$) δ 172.2, 160.4 (d, *J* = 251.7 Hz), 147.5 (d, *J* = 8.7 Hz), 140.1, 137.9 (d, *J* = 11.6 Hz), 136.1, 131.7 (d, *J* = 4.3 Hz), 130.3, 128.4, 128.3, 127.9, 127.8, 126.8, 118.1 (d, *J* = 3.6 Hz), 111.4 (d, *J* = 29.1 Hz), 59.1 (d, *J* = 2.2 Hz), 52.6, 42.1. HRMS (ESI): Exact mass calcd. for $C_{22}H_{18}FnaO_4$ $[M+Na]^+$: 402.1112. Found: 402.1122.

85. methyl 2-(4-nitrophenyl)-2,3-diphenylpropanoate (**12u**)

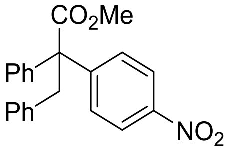

12u

Yellow oil. 52% yield. ^{1}H NMR (300 MHz, $CDCl_3$) δ 8.02-7.95 (m, 2H), 7.38-7.22 (m, 6H), 7.20-7.13 (m, 2H), 7.12-7.00 (m, 2H), 6.70-6.61 (m, 2H), 4.02 (d, *J* = 13.2 Hz, 1H), 3.71 (s, 3H), 3.47 (d, *J* = 12.6 Hz, 1H); ^{13}C NMR (75 MHz, $CDCl_3$) δ 172.8, 150.0, 146.4, 141.8, 136.2, 130.6, 130.4, 128.4, 128.3, 127.8, 127.6, 126.7, 122.3, 62.0, 52.4, 44.1. HRMS (ESI): Exact mass calcd. for $C_{22}H_{19}NNaO_4$ $[M+Na]^+$: 384.1206. Found: 384.1201.

86. 2-(4-nitrophenyl)-2,3-diphenylpropanenitrile (**12v**)

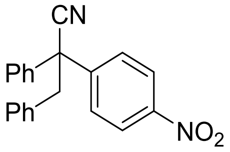

12v

Yellow oil. 54% yield. ^{1}H NMR (400 MHz, $CDCl_3$) δ 8.20-8.12 (m, 2H), 7.49-7.42 (m, 2H), 7.41-7.32 (m, 5H), 7.25-7.13 (m, 3H), 6.91-6.86 (m, 2H), 3.75 (d, *J* = 13.2 Hz, 1H), 3.64 (d, *J* = 13.2 Hz, 1H); ^{13}C NMR (100 MHz, $CDCl_3$) δ 147.4, 147.0, 138.7, 133.6, 130.4, 129.1, 128.7, 128.5, 128.2, 127.7, 127.3, 123.8, 120.8, 52.9, 45.1. HRMS (ESI): Exact mass calcd. for $C_{21}H_{17}N_2O_2$ $[M+H]^+$: 329.1285. Found: 329.1287.

87. methyl 2-(2-fluoro-4-nitrophenyl)-2-phenylpropanoate (**12w**)

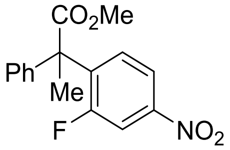

12w

Yellow oil. 46% yield. ^{1}H NMR (400 MHz, $CDCl_3$) δ 7.91 (dd, *J* = 10.4, 2.4 Hz, 2H), 7.84 (dd, *J* = 8.8, 2.0 Hz, 2H), 6.89 (t, *J* = 8.0 Hz, 1H), 3.75 (s, 3H), 1.99 (s, 3H); ^{13}C NMR (100 MHz, $CDCl_3$) δ 173.4, 160.0 (d, *J* = 251.0 Hz), 147.5 (d, *J* = 8.8 Hz), 140.9 (d, *J* = 12.4 Hz), 139.2, 129.9 (d, *J* = 4.4

Hz), 128.7, 128.0, 127.6, 118.7 (d, *J* = 3.6 Hz), 111.4 (d, *J* = 28.4 Hz), 53.5 (d, *J* = 1.4 Hz), 52.7, 23.6 (d, *J* = 2.2 Hz). HRMS (ESI): Exact mass calcd. for $C_{16}H_{15}FNO_4$ $[M+H]^+$: 304.0980. Found: 304.0976.

88. 1,3-diallyl-3-(4-nitrophenyl)indolin-2-one (**12x**)

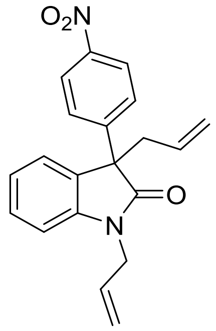

12x

Yellow solid, m.p. 97.0-98.6 °C. 36% yield. ^{1}H NMR (600 MHz, $CDCl_3$) δ 8.16 (d, *J* = 8.9 Hz, 2H), 7.59 (d, *J* =8.9 Hz, 2H), 7.35 (td, *J* = 7.7, 1.3 Hz, 1H), 7.27 (d, *J* = 7.4 Hz, 1H), 7.15 (t, *J* = 7.5 Hz, 1H), 6.93 (d, J = 7.9 Hz, 1H), 5.79 (ddt, *J* = 7.9 Hz, 1H), 5.39 (dddd, *J* = 7.9 Hz, 1H), 5.20 (m, 2H), 5.07 (dq, *J* = 17.1, 1.5 Hz, 1H), 4.98 (d, *J* = 10.9 Hz, 1H), 4.34 (m, 2H), 3.05 (m, 2H) ; ^{13}C NMR (151 MHz, $CDCl_3$) δ 176.5, 147.2, 146.9, 143.0, 131.5, 131.2, 130.4, 128.9, 128.3 (2C), 125.3, 123.7 (2C), 122.9, 120.3, 117.9, 109.7, 56.5, 42.7, 42.4. HRMS (ESI): Exact mass calcd. for $C_{20}H_{19}N_2O_3$ $[M+H]^+$: 335.1369. Found: 335.1365.

Experimental Procedures for the Formation of Heterocycles and Intermediates

Substituted Carbazole Synthesis

PhMgBr (0.92 M in THF solution) (3.3 mL, 3 mmol) was slowly (0.3 mL/min) added to the mixture of methyl 2-(6-nitro-[1,1'-biphenyl]-3-yl)-2-phenylacetate **3d** (348 mg, 1 mmol) and dry THF (10 mL) at 0 °C in 10 minutes. Then the mixture was stirred at 0 °C for 5 minutes followed by the slow addition of saturated NH_4Cl aqueous solution (0.5 mL). Then 50 mL water was added and the resulting mixture was extracted with ethyl acetate (3×30mL). The combined organic layers were washed with brine (50 mL), dried over anhydrous Na_2SO_4, filtered and concentrated in vacuo. The crude product was purified by column chromatography (Hexanes:Ethyl Acetate = 10:1) [4] to give methyl 2-(9*H*-carbazol-3-yl)-2-phenylacetate **13** (155 mg, 49%) as a white solid.

Substituted Tetrahydrofuran Synthesis

Step 1: To a solution of Diisobutylaluminium hydride (4 mL, 1.5 M in toluene, 6.0 mmol) in THF (10 mL), methyl 2-(4-nitrophenyl)-2-phenylpent-4-enoate **12a** (620 mg, 2 mmol) in Et_2O (10 mL) was slowly added at 0 °C. After stirred for 12 h, the reaction mixture was carefully quenched by the

slow addition of saturated NH_4Cl aqueous solution (0.5 mL). Then 50 mL water was added and the resulting mixture was extracted with ethyl acetate (3×30mL). The combined organic layers were washed with brine (50 mL), dried over Na_2SO_4 and concentrated under reduced pressure. The residue was purified by flash column chromatography (Hexanes:Ethyl Acetate = 10:1)[5] to give 2-(4-nitrophenyl)-2-phenylpent-4-en-1-ol **14** (364 mg, 64%) as a colorless oil.

Step 2: A solution of I_2 (12.8 mg, 0.05 mmol) and $PhSiH_3$ (12.3 μL, 0.10 mmol) in CH_2Cl_2 (5 mL) was stirred for 15 ~30 min and 2-(4-nitrophenyl)-2-phenylpent-4-en-1-ol **14** (140 mg, 0.5 mmol) was then added under air. The resulting mixture was quenched with saturated aqueous Na_2CO_3 and extracted with EtOAc. The organic layer was washed with brine, dried over Na_2SO_4 and concentrated under reduced pressure. The residue was purified by flash column chromatography (Hexane:EtOAc = 10:1)[5] to give 2-methyl-4-(4-nitrophenyl)-4-phenyltetrahydrofuran **15** (99.6 mg, 70%) as pale yellow oil (*dr* = 1:1).

Analysis Data of Heterocycles and Intermediates

89. methyl 2-(9H-carbazol-3-yl)-2-phenylacetate (**13**)

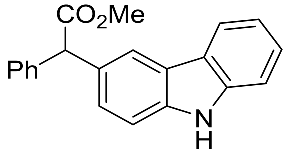

13

White solid, m.p. 165-167 °C. 49% yield. ^1H NMR (400 MHz, $CDCl_3$) δ 8.00 (d, *J* = 7.6 Hz, 3H), 7.39-7.22 (m, 9H), 7.21-7.15 (m, 1H), 5.22 (s, 1H), 3.76 (s, 3H); ^{13}C NMR (75 MHz, $CDCl_3$) δ 173.7, 139.8, 139.4, 138.7, 129.6, 128.6 (2C), 127.1, 126.6, 125.9, 123.5, 123.1, 120.4, 120.3, 119.4, 110.7, 110.6, 57.0, 52.3. HRMS (ESI): Exact mass calcd. for $C_{21}H_{18}NO_2$ $[M+H]^+$: 316.1332. Found: 316.1337.

90. 2-(4-nitrophenyl)-2-phenylpent-4-en-1-ol (**14**)

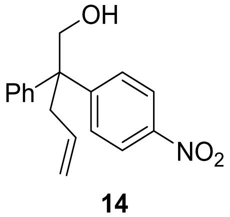

14

Pale yellow oil. 64% yield. ^1H NMR (400 MHz, $CDCl_3$) δ 8.17-8.10 (m, 2H), 7.40-7.20 (m, 5H), 7.18-7.11 (m, 2H), 5.48-5.32 (m, 1H), 5.13-4.98 (m, 2H), 4.17 (q, *J* = 10.8 Hz, 2H), 3.06-2.91 (m, 2H); ^{13}C NMR (100 MHz, $CDCl_3$) δ 153.3, 146.3, 143.9, 133.4, 129.2, 128.5, 128.0, 127.0, 123.1, 119.0, 67.5, 52.0, 40.9. HRMS (ESI): Exact mass calcd. for $C_{17}H_{18}NO_3$ $[M+H]^+$: 284.1281. Found: 284.1283.

91. 2-methyl-4-(4-nitrophenyl)-4-phenyltetrahydrofuran (**15**)

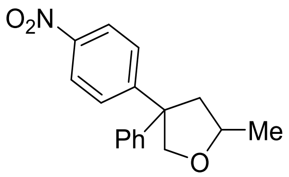

15

Pale yellow oil. 64% yield. ^{1}H NMR (300 MHz, $CDCl_3$) δ 8.13-8.03 (m, 2H), 7.46 (dd, *J* = 6.9, 2.1 Hz, 1H), 7.38 (dd, *J* = 6.9, 2.1 Hz, 1H), 7.34-7.15 (m, 5H), 4.61-4.45 (m, 1H), 4.28-4.12 (m, 2H), 2.83-2.55 (m, 1H), 2.40-2.17 (m, 1H), 1.30 (t, *J* = 6.0 Hz, 3H),; ^{13}C NMR (75 MHz, $CDCl_3$) δ 154.04, 153.95, 146.1, 146.0, 144.5, 144.2, 128.5, 128.4, 127.9, 127.8, 126.89, 126.86, 126.8, 126.6, 123.3, 123.2, 76.4, 76.0, 74.8, 74.4, 56.6, 56.4, 46.2, 46.1, 21.1, 21.0. HRMS (ESI): Exact mass calcd. for $C_{17}H_{18}NO_3$ $[M+H]^+$: 284.1281. Found: 284.1289.

Analysis Data of Arylation Products Formed with *para*-substituted Nitroarenes

methyl 2-(2-nitro-5-(trifluoromethyl)phenyl)-2-phenylacetate (**25**)

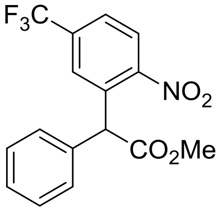

25

Yellow oil. 18% yield. ^{1}H NMR (600 MHz, Chloroform-*d*) δ 8.10 (d, *J* = 8.4 Hz, 1H), 7.70 (dd, *J* = 8.5, 1.9 Hz, 1H), 7.45 – 7.38 (m, 4H), 7.29 – 7.24 (m, 2H), 5.68 (s, 1H), 3.77 (s, 3H). ^{13}C NMR (151 MHz, $CDCl_3$) δ 171.25, 150.88, 135.41, 134.87, 134.59 (d, *J* = 33.4 Hz), 129.44, 129.04, 128.78 (q, *J* = 3.8 Hz), 128.52, 125.54, 125.51 (q, *J* = 3.8 Hz), 122.75 (d, *J* = 273.2 Hz), 52.89, 52.84.

methyl 2-(4-nitrophenyl)-2-phenylacetate (**27**)

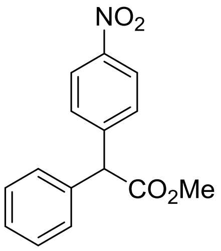

27

Pale yellow oil. 47% yield. ^{1}H NMR (600 MHz, Chloroform-*d*) δ 8.20 – 8.15 (m, 2H), 7.52 – 7.46 (m, 2H), 7.39 – 7.34 (m, 2H), 7.34 – 7.27 (m, 3H), 5.12 (s, 1H), 3.78 (s, 3H). ^{13}C NMR (151 MHz, $CDCl_3$) δ 171.84, 147.18, 145.84, 137.22, 129.63 (2C), 129.04 (2C), 128.45 (2C), 127.95, 123.77 (2C), 56.66, 52.70.

methyl 2-(5-cyano-2-nitrophenyl)-2-phenylacetate (**29a**) and methyl 2-(3-cyanophenyl)-2-phenylacetate (**29 b**)

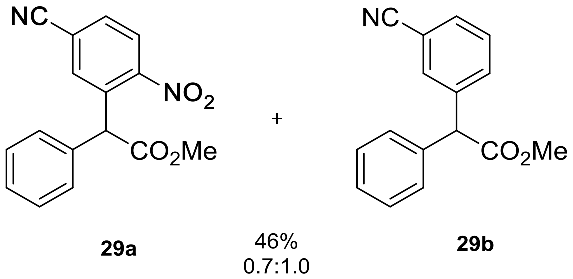

Yellow oil. 46% yield (isolated as a mixture) ^{1}H NMR (600 MHz, Chloroform-d) δ 8.09 (d, J = 8.4 Hz, 1H), 7.73 (dd, J = 8.4, 1.8 Hz, 1H), 7.64 – 7.58 (m, 2H), 7.47 – 7.40 (m, 6H), 7.37 – 7.33 (m, 2H), 7.33 – 7.26 (m, 3H), 7.26 – 7.23 (m, 2H), 5.64 (s, 1H), 5.06 (s, 1H), 3.77 (s, 3H), 3.76 (s, 3H). ^{13}C NMR (151 MHz, $CDCl_3$) δ 171.94, 171.01, 150.99, 143.89, 137.30, 135.65, 135.38, 135.01, 132.37 (2C), 131.98, 129.67 (2C), 129.49 (2C), 129.01(2C), 128.98 (2C), 128.76, 128.47 (2C), 127.87, 125.46, 118.62, 116.95, 116.79, 111.33, 56.86, 52.95, 52.79, 52.64. (**29a**)HRMS (ESI): Exact mass calcd. for $C_{16}H_{12}N_2O_4Na$ $[M+Na]^+$: 319.0689. Found: 319.0667. (**29b**)HRMS (ESI): Exact mass calcd. for $C_{16}H_{14}NO_2$ $[M+H]^+$: 252.1019. Found: 252.2019.

¹H NMR Study of DMS in Crude Reaction Mixture

Methyl 2-phenylacetate (**1**) was submitted to the standard arylation conditions using 2-cyanonitrobenzene (**2a**) as the coupling partner. The presence or absence of DMS was observed in crude ¹H NMR spectra. To make the NMR samples, 250 μL alliquots were removed from the crude reaction mixture and combined with the internal standard maleic acid (6.1 mg, 0.0525 mmol). Crude ¹H NMR was taken on a Bruker DRX-600 spectrometer operating at 600 MHz.

Pure Standards in DMSO-d6

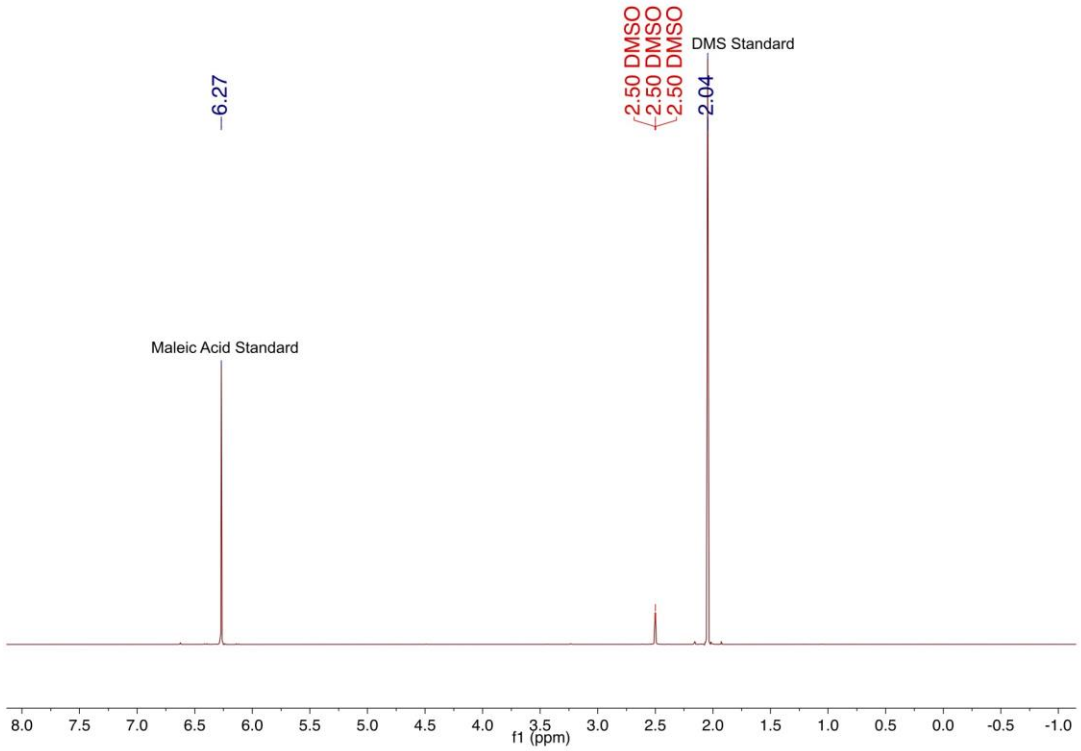

Crude Reaction Mixture at the Begining of the Reaction in DMSO

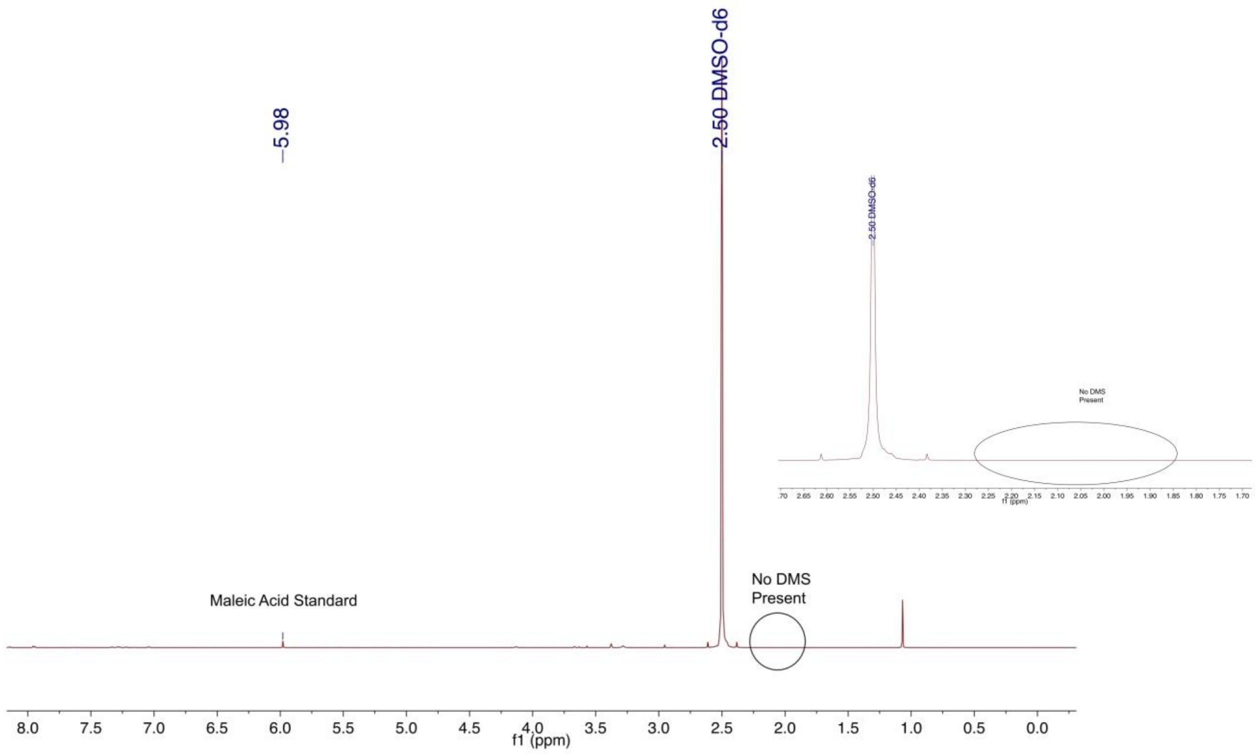

Crude Reaction Mixture After 30 Minutes in DMSO

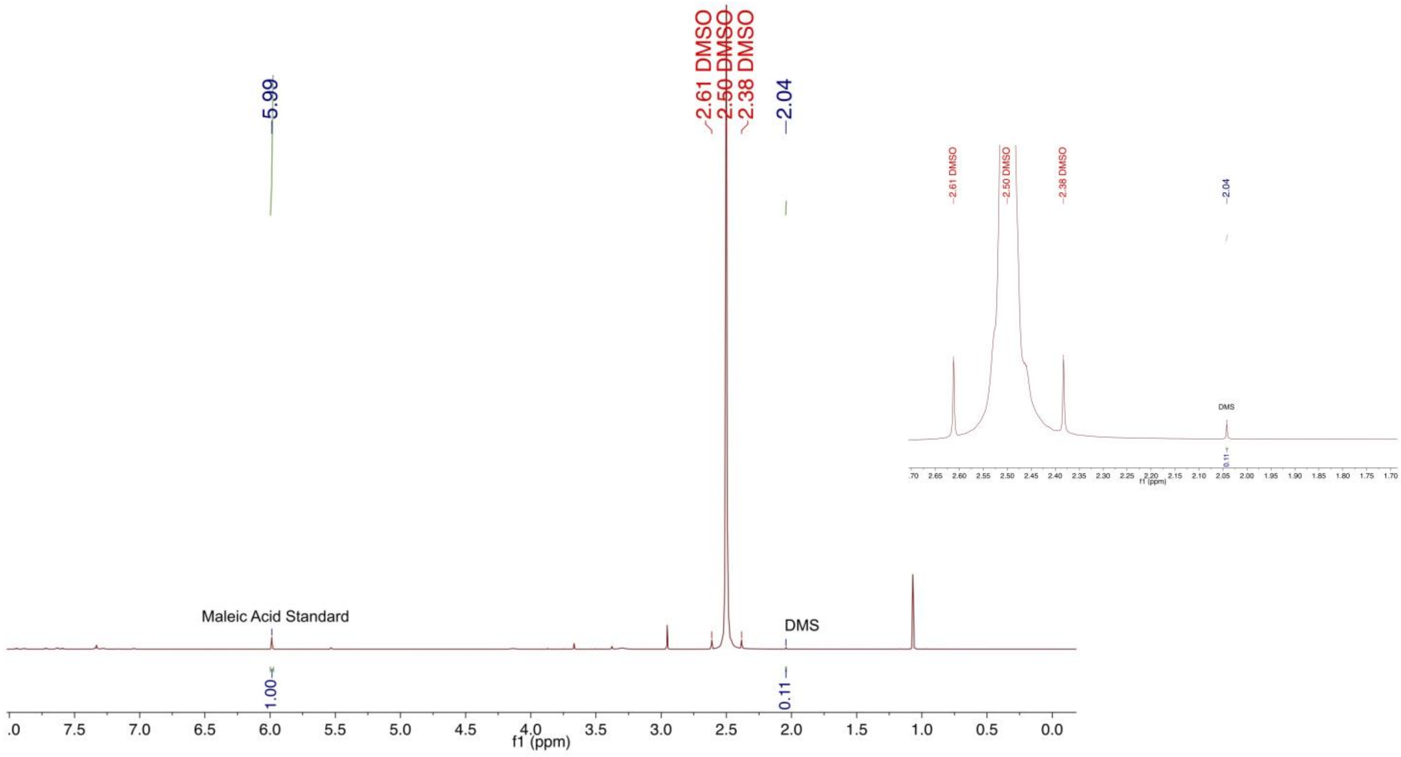

Crude Reaction Mixture After 30 Minutes in DMSO Showing Arylated Product **3a**

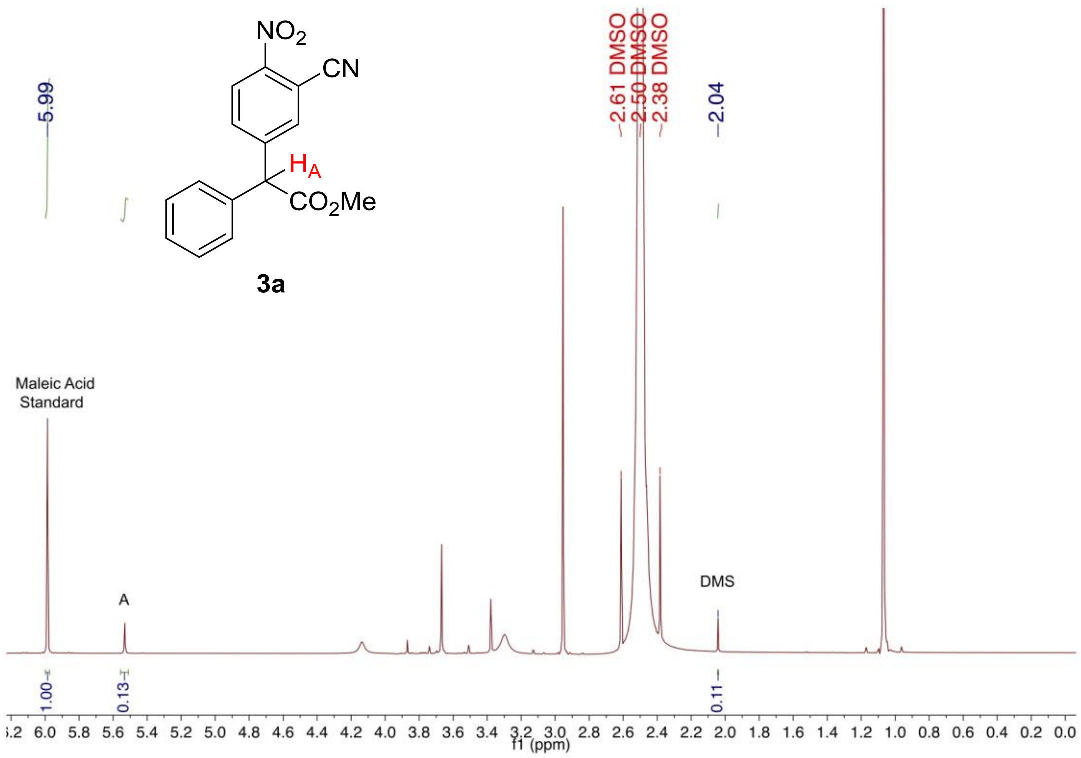

Based on peak integrations, 0.00190 mmol of DMS was present in the sample after 30 minutes of reaction time. In the same sample, 0.0136 mmol of arylated product **3a** was calculated to be present based on peak integrations. Although DMS was present, there was not a 1:1 molar ratio of DMS to product **3a**. Therefore, DMS cannot be acting as the major oxidizing agent.

DMS : Arylated Product **3a** = 0.00190 mmol : 0.0136 mmol = 1 : 7 molar ratio

Crude Reaction Mixture After 30 Minutes Doped with DMS Standard in DMSO

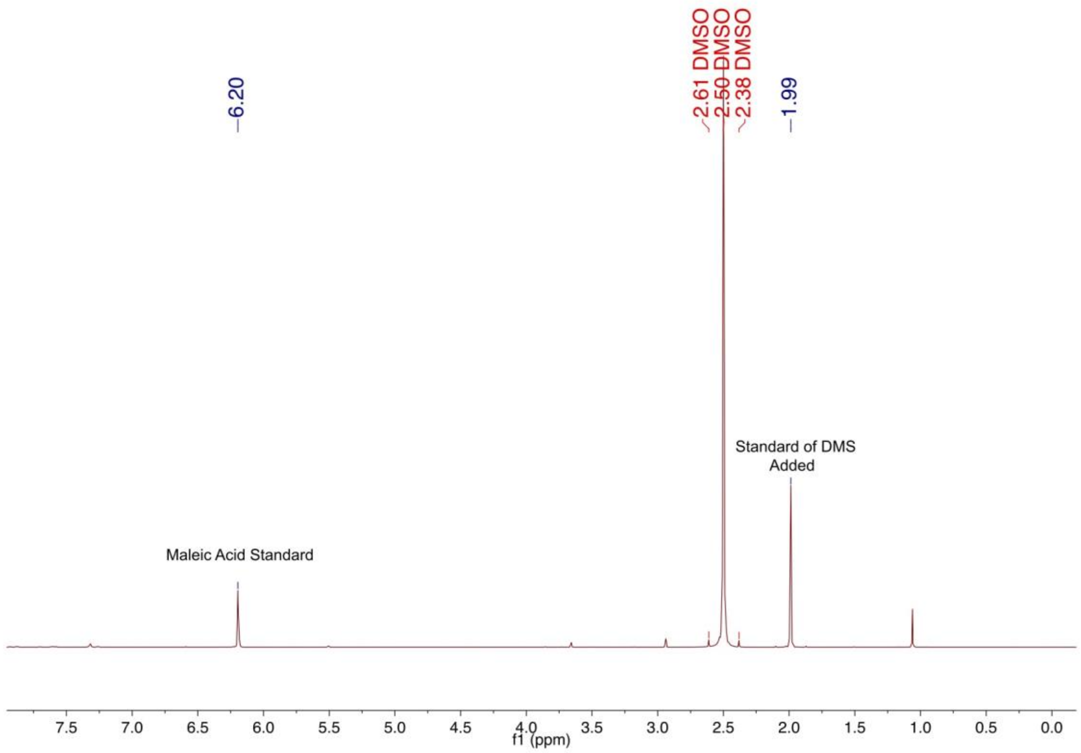

Refrences

1. (a) WO2007107539A1, 2007. (b) WO2008122667A2, 2008.
2. (a) C. Bourgeois, E. Werfel, F. Galla, K. Lehmkuhl, H. Torres-Gomez, D. Schepmann, B. Kogel, T. Christoph, W. Strassburger, W. Englberger, M. Soeberdt, S. Huwel, H. J. Galla and B. Wunsch, *J. Med. Chem.*, 2014, **57**, 6845-6860. (b) D. A. Campbell, *J. Org. Chem.*, 1992, **57**, 1523-1527. (c) H. Huang, W. Lu, X. Li, X. Cong, H. Ma, X. Liu, Y. Zhang, P. Che, R. Ma, H. Li, X. Shen, H. Jiang, J. Huang and J. Zhu, *Bioorg. Med. Chem. Lett.*, 2012, **22**, 958-962**.** (d) WO2011060397A1, 2011. (e) A. T. Soldatenkov, S. A. Soldatova, J. A. Mamyrbekova-Bekro, G. S. Gimranova, A. V. Malkova, K. B. Polyanskii, N. M. Kolyadina and V. N. Khrustalev, *Chem. Heterocycl. Compd.*, 2012, **48**, 1332-1339**.** (f) S. W. Wright, D. L. Hageman, A. S. Wright and L. D. McClure, *Tetrahedron Lett.*, 1997, **38**, 7345-7348.
3. Adam, W.; Bosio, S. G.; Turro, N. J., *J. Am. Chem. Soc.* **2002**, *124*, 8814-8815.
4. H. Gao, Q. L. Xu, M. Yousufuddin, D. H. Ess and L. Kürti, *Angew. Chem. Int. Ed. Engl.*, 2014, **53**, 2701-2705.
5. S. Fujita, M. Abe, M. Shibuya and Y. Yamamoto, *Org. Lett.*, 2015, **17**, 3822-3825.

X-Ray Diffraction Data – Compound 4g

Diffraction data were collected on a colorless plate (approximate dimensions 0.40 x 0.35 x 0.20 mm3) at room temperature (T = 23 °C) on a Bruker APEX-II CCD diffractometer with graphite-monochromated Mo Kα radiation (λ = 0.71073 Å). The cell parameters for the organic complex were obtained from the least-squares refinement of the spots (from 36 collected frames) using the APEX2 program. A hemisphere of the crystal data was collected up to a resolution of 0.75 Å, and the intensity data were processed using the APEX2 program. All calculations for structure determination were carried out using the SHELXL-2014 package. Initial atomic positions were located using Intrinsic Phasing, and the structure was refined by least-squares methods using SHELX with 6515 independent reflections and within the range of Θ = 2.20-33.13 (completeness 100%). Calculated hydrogen positions were input and refined in a riding manner along with the attached carbons. A CF_3 group demonstrated disorder and this was treated with a combination DELU, SIMU, EADP, and ISOR restraints.

Table 1. Crystal data and structure refinement for C18 H13 F3 N2 O4.

Identification code	kl128_a	
Empirical formula	C18 H13 F3 N2 O4	
Formula weight	378.30	
Temperature	296(2) K	
Wavelength	0.71073 Å	
Crystal system	Monoclinic	
Space group	$P2_1/c$	
Unit cell dimensions	a = 18.290(2) Å	α= 90°.
	b = 10.7507(14) Å	β= 98.659(2)°.
	c = 9.2330(12) Å	γ = 90°.
Volume	1794.8(4) $Å^3$	
Z	4	
Density (calculated)	1.400 Mg/m^3	
Absorption coefficient	0.120 mm^{-1}	
F(000)	776	
Crystal size	0.400 x 0.350 x 0.200 mm^3	
Theta range for data collection	2.204 to 33.126°.	

Index ranges	-27<=h<=27, -16<=k<=16, -14<=l<=13
Reflections collected	23928
Independent reflections	6515 [R(int) = 0.0201]
Completeness to theta = 25.000°	100.0 %
Absorption correction	Semi-empirical from equivalents
Max. and min. transmission	0.746 and 0.686
Refinement method	Full-matrix least-squares on F^2
Data / restraints / parameters	6515 / 72 / 255
Goodness-of-fit on F^2	1.059
Final R indices [I>2sigma(I)]	R1 = 0.0696, wR2 = 0.2152
R indices (all data)	R1 = 0.1075, wR2 = 0.2452
Extinction coefficient	n/a
Largest diff. peak and hole	0.383 and -0.355 e.Å^{-3}

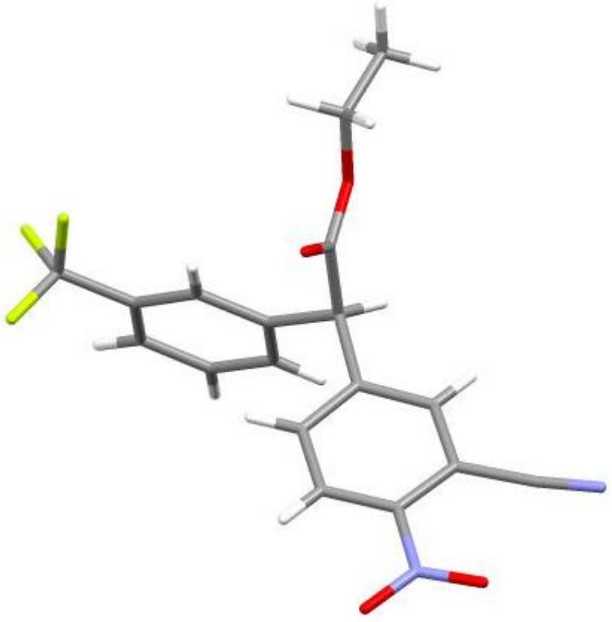

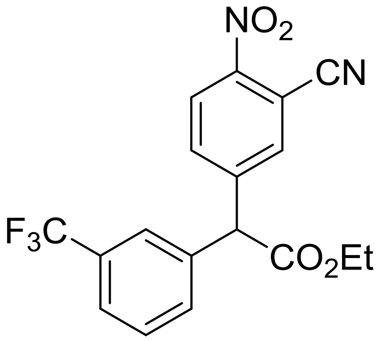

4g

Table 2. Atomic coordinates (x 10^4) and equivalent isotropic displacement parameters ($Å^2$x 10^3) for C18 H13 F3 N2 O4. U(eq) is defined as one third of the trace of the orthogonalized U^{ij} tensor.

	x	y	z	U(eq)
F(1)	9714(3)	6512(5)	3697(7)	150(2)
F(2)	10308(2)	5043(5)	3085(8)	160(2)
F(3)	9709(4)	6198(7)	1504(6)	169(2)
F(1A)	10068(8)	5808(14)	3772(12)	169(2)
F(2A)	10018(5)	5368(10)	1529(12)	150(2)
F(3A)	9424(3)	6837(9)	2247(15)	160(2)
O(1)	4770(1)	7435(2)	-672(2)	98(1)
O(2)	4049(1)	6620(2)	652(2)	101(1)
O(3)	7331(1)	6796(1)	5057(2)	65(1)
O(4)	7689(1)	5160(1)	6482(1)	66(1)
N(1)	4658(1)	6786(1)	338(2)	60(1)
N(2)	3965(1)	4570(3)	3082(3)	94(1)
C(1)	7170(1)	4718(1)	4057(2)	45(1)
C(2)	7831(1)	4354(2)	3324(2)	48(1)
C(3)	7832(1)	3210(2)	2618(3)	68(1)
C(4)	8427(2)	2861(2)	1941(3)	85(1)
C(5)	9023(1)	3630(2)	1958(3)	80(1)
C(6)	9027(1)	4758(2)	2641(3)	69(1)
C(7)	8438(1)	5130(2)	3332(2)	57(1)
C(8)	9673(2)	5621(4)	2651(4)	105(1)
C(9)	6517(1)	5207(1)	3002(2)	44(1)
C(10)	6599(1)	6008(2)	1856(2)	53(1)

C(11)	5992(1)	6508(2)	981(2)	54(1)
C(12)	5292(1)	6200(1)	1248(2)	47(1)
C(13)	5186(1)	5379(2)	2368(2)	47(1)
C(14)	5808(1)	4888(2)	3234(2)	47(1)
C(15)	4481(1)	4979(2)	2700(2)	62(1)
C(16)	7395(1)	5693(2)	5246(2)	50(1)
C(17)	7895(2)	6001(3)	7723(2)	87(1)
C(18)	8042(3)	5286(4)	9030(3)	136(2)

Table 3. Bond lengths [Å] and angles [°] for C18 H13 F3 N2 O4

F(1)-C(8)	1.354(5)
F(2)-C(8)	1.325(5)
F(3)-C(8)	1.238(5)
F(1A)-C(8)	1.186(10)
F(2A)-C(8)	1.320(8)
F(3A)-C(8)	1.415(10)
O(1)-N(1)	1.206(2)
O(2)-N(1)	1.206(2)
O(3)-C(16)	1.202(2)
O(4)-C(16)	1.317(2)
O(4)-C(17)	1.464(3)
N(1)-C(12)	1.468(2)
N(2)-C(15)	1.144(3)
C(1)-C(9)	1.516(2)
C(1)-C(2)	1.523(2)
C(1)-C(16)	1.528(2)
C(1)-H(1)	0.9800
C(2)-C(7)	1.387(2)
C(2)-C(3)	1.392(2)
C(3)-C(4)	1.387(3)
C(3)-H(3)	0.9300
C(4)-C(5)	1.366(4)
C(4)-H(4)	0.9300
C(5)-C(6)	1.366(3)
C(5)-H(5)	0.9300

C(6)-C(7)	1.391(3)
C(6)-C(8)	1.502(4)
C(7)-H(7)	0.9300
C(9)-C(14)	1.389(2)
C(9)-C(10)	1.391(2)
C(10)-C(11)	1.380(2)
C(10)-H(10)	0.9300
C(11)-C(12)	1.379(2)
C(11)-H(11)	0.9300
C(12)-C(13)	1.395(2)
C(13)-C(14)	1.392(2)
C(13)-C(15)	1.436(2)
C(14)-H(14)	0.9300
C(17)-C(18)	1.422(4)
C(17)-H(17A)	0.9700
C(17)-H(17B)	0.9700
C(18)-H(18A)	0.9600
C(18)-H(18B)	0.9600
C(18)-H(18C)	0.9600
C(16)-O(4)-C(17)	115.66(16)
O(1)-N(1)-O(2)	122.82(18)
O(1)-N(1)-C(12)	118.62(17)
O(2)-N(1)-C(12)	118.53(17)
C(9)-C(1)-C(2)	113.86(13)
C(9)-C(1)-C(16)	108.94(13)
C(2)-C(1)-C(16)	110.44(12)
C(9)-C(1)-H(1)	107.8

C(2)-C(1)-H(1)	107.8
C(16)-C(1)-H(1)	107.8
C(7)-C(2)-C(3)	118.46(17)
C(7)-C(2)-C(1)	122.09(14)
C(3)-C(2)-C(1)	119.45(16)
C(4)-C(3)-C(2)	120.4(2)
C(4)-C(3)-H(3)	119.8
C(2)-C(3)-H(3)	119.8
C(5)-C(4)-C(3)	120.7(2)
C(5)-C(4)-H(4)	119.6
C(3)-C(4)-H(4)	119.6
C(4)-C(5)-C(6)	119.4(2)
C(4)-C(5)-H(5)	120.3
C(6)-C(5)-H(5)	120.3
C(5)-C(6)-C(7)	121.1(2)
C(5)-C(6)-C(8)	120.1(2)
C(7)-C(6)-C(8)	118.9(2)
C(2)-C(7)-C(6)	119.93(18)
C(2)-C(7)-H(7)	120.0
C(6)-C(7)-H(7)	120.0
F(1A)-C(8)-F(2A)	114.8(8)
F(3)-C(8)-F(2)	109.6(4)
F(3)-C(8)-F(1)	104.6(5)
F(2)-C(8)-F(1)	99.5(4)
F(1A)-C(8)-F(3A)	101.8(8)
F(2A)-C(8)-F(3A)	99.0(7)
F(1A)-C(8)-C(6)	119.0(5)

F(3)-C(8)-C(6)	116.6(4)
F(2A)-C(8)-C(6)	109.6(4)
F(2)-C(8)-C(6)	111.4(4)
F(1)-C(8)-C(6)	113.6(3)
F(3A)-C(8)-C(6)	110.3(3)
C(14)-C(9)-C(10)	118.72(15)
C(14)-C(9)-C(1)	118.62(14)
C(10)-C(9)-C(1)	122.59(14)
C(11)-C(10)-C(9)	121.10(15)
C(11)-C(10)-H(10)	119.5
C(9)-C(10)-H(10)	119.5
C(12)-C(11)-C(10)	119.27(15)
C(12)-C(11)-H(11)	120.4
C(10)-C(11)-H(11)	120.4
C(11)-C(12)-C(13)	121.40(15)
C(11)-C(12)-N(1)	117.95(15)
C(13)-C(12)-N(1)	120.63(15)
C(14)-C(13)-C(12)	118.22(14)
C(14)-C(13)-C(15)	116.47(15)
C(12)-C(13)-C(15)	125.31(16)
C(9)-C(14)-C(13)	121.27(15)
C(9)-C(14)-H(14)	119.4
C(13)-C(14)-H(14)	119.4
N(2)-C(15)-C(13)	172.0(2)
O(3)-C(16)-O(4)	124.92(16)
O(3)-C(16)-C(1)	124.26(15)
O(4)-C(16)-C(1)	110.79(14)

C(18)-C(17)-O(4)	108.9(2)
C(18)-C(17)-H(17A)	109.9
O(4)-C(17)-H(17A)	109.9
C(18)-C(17)-H(17B)	109.9
O(4)-C(17)-H(17B)	109.9
H(17A)-C(17)-H(17B)	108.3
C(17)-C(18)-H(18A)	109.5
C(17)-C(18)-H(18B)	109.5
H(18A)-C(18)-H(18B)	109.5
C(17)-C(18)-H(18C)	109.5
H(18A)-C(18)-H(18C)	109.5
H(18B)-C(18)-H(18C)	109.5

Symmetry transformations used to generate equivalent atoms:

Table 4. Anisotropic displacement parameters ($Å^2 x 10^3$) C18 H13 F3 N2 O4. The anisotropic displacement factor exponent takes the form: $-2\pi^2[h^2 a^{*2}U^{11} + ... + 2 h k a^* b^* U^{12}]$

	U^{11}	U^{22}	U^{33}	U^{23}	U^{13}	U^{12}
F(1)	109(2)	160(3)	195(4)	-63(3)	70(3)	-67(2)
F(2)	55(1)	189(3)	232(4)	-18(3)	14(2)	-14(2)
F(3)	174(4)	192(4)	141(3)	38(3)	25(3)	-84(3)
F(1A)	174(4)	192(4)	141(3)	38(3)	25(3)	-84(3)
F(2A)	109(2)	160(3)	195(4)	-63(3)	70(3)	-67(2)
F(3A)	55(1)	189(3)	232(4)	-18(3)	14(2)	-14(2)
O(1)	93(1)	95(1)	100(1)	45(1)	-4(1)	10(1)
O(2)	56(1)	121(2)	121(2)	29(1)	-1(1)	13(1)
O(3)	79(1)	53(1)	64(1)	-9(1)	13(1)	0(1)
O(4)	71(1)	74(1)	50(1)	-5(1)	-3(1)	14(1)
N(1)	62(1)	49(1)	66(1)	-3(1)	-4(1)	4(1)
N(2)	57(1)	125(2)	102(2)	12(1)	18(1)	-22(1)
C(1)	45(1)	44(1)	48(1)	1(1)	7(1)	-3(1)
C(2)	48(1)	44(1)	51(1)	-1(1)	5(1)	3(1)
C(3)	72(1)	49(1)	82(1)	-12(1)	13(1)	1(1)
C(4)	97(2)	60(1)	100(2)	-20(1)	26(1)	18(1)
C(5)	69(1)	86(2)	88(2)	-6(1)	22(1)	28(1)
C(6)	50(1)	80(1)	79(1)	-2(1)	14(1)	4(1)
C(7)	49(1)	56(1)	66(1)	-9(1)	11(1)	-3(1)
C(8)	58(1)	150(3)	114(2)	-10(2)	31(2)	-8(2)
C(9)	45(1)	42(1)	46(1)	-1(1)	8(1)	-5(1)
C(10)	48(1)	56(1)	56(1)	8(1)	12(1)	-7(1)

C(11)	59(1)	50(1)	54(1)	10(1)	9(1)	-5(1)
C(12)	51(1)	41(1)	48(1)	-4(1)	2(1)	-1(1)
C(13)	44(1)	49(1)	48(1)	-7(1)	8(1)	-8(1)
C(14)	47(1)	48(1)	47(1)	2(1)	8(1)	-10(1)
C(15)	47(1)	76(1)	64(1)	1(1)	8(1)	-10(1)
C(16)	44(1)	56(1)	50(1)	-4(1)	9(1)	2(1)
C(17)	96(2)	102(2)	60(1)	-21(1)	-7(1)	10(1)
C(18)	191(4)	151(3)	58(2)	-8(2)	-11(2)	50(3)

Table 5. Hydrogen coordinates (x 10^4) and isotropic displacement parameters ($Å^2$x 10^3) for C18 H13 F3 N2 O4.

	x	y	z	U(eq)
H(1)	7006	3974	4532	55
H(3)	7430	2677	2601	81
H(4)	8420	2096	1469	102
H(5)	9423	3387	1510	96
H(7)	8451	5898	3799	68
H(10)	7072	6211	1675	63
H(11)	6053	7046	220	65
H(14)	5747	4335	3981	57
H(17A)	8331	6474	7586	105
H(17B)	7495	6581	7791	105
H(18A)	7604	4840	9174	204
H(18B)	8188	5829	9848	204
H(18C)	8433	4706	8949	204

X-Ray Diffraction Data – Compound 12s

Table 6 Crystal data and structure refinement for lft-1-19(compound 12s).

Identification code	lft-1-19(compound 12s)
Empirical formula	$C_{19}H_{15}BrN_2O_3$
Formula weight	399.24
Temperature/K	298.15
Crystal system	monoclinic
Space group	$P2_1/c$
a/Å	10.499(3)
b/Å	20.581(6)
c/Å	8.367(2)
α/°	90
β/°	108.054(3)
γ/°	90
Volume/Å3	1718.9(8)
Z	4
ρcalcg/cm3	1.543
μ/mm-1	2.412
F(000)	808.0
Crystal size/mm3	0.15 × 0.1 × 0.08
Radiation	MoKα (λ = 0.710)
2Θ range for data collection/ °	3.954 to 49.98
Index ranges	$-12 \leq h \leq 12$, $-24 \leq k \leq 24$, $-9 \leq l \leq 9$
Reflections collected	15229
Independent reflections	3039 [Rint = 0.0709, Rsigma = 0.0592]
Data/restraints/parameters	3039/0/228
Goodness-of-fit on F2	1.026
Final R indexes [I>=2σ (I)]	R1 = 0.0398, wR2 = 0.0688
Final R indexes [all data]	R1 = 0.0796, wR2 = 0.0814
Largest diff. peak/hole / e Å-3	0.26/-0.43

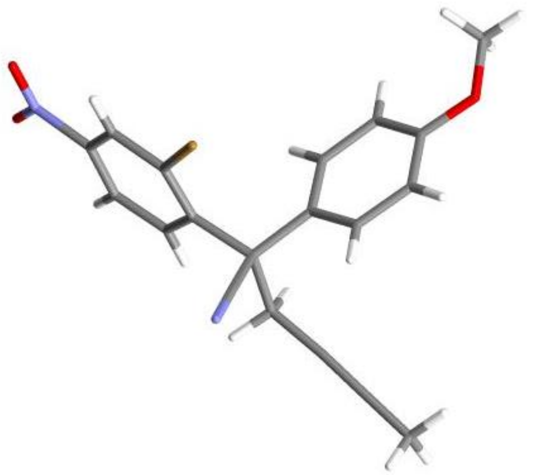

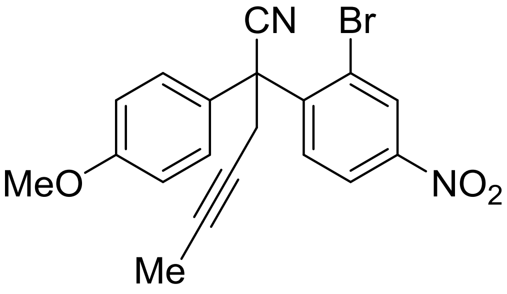

12s

Table 7 Fractional Atomic Coordinates (×104) and Equivalent Isotropic Displacement Parameters (Å2×103) for lft-1-19(compound 12s). Ueq is defined as 1/3 of of the trace of the orthogonalised UIJ tensor.

Atom	x	y	z	U(eq)
Br1	5803.2(4)	4178.0(2)	5909.4(5)	50.47(15)
C1	7955(4)	5502(2)	-1392(5)	67.6(13)
C2	8300(4)	5104(2)	126(5)	48.9(10)
C3	8583(4)	4780.0(19)	1338(5)	45.3(10)
C4	9003(4)	4398.1(18)	2889(5)	44.3(10)
C5	7863(3)	4306.2(16)	3717(4)	36.1(9)
C6	7452(4)	4974.3(19)	3987(4)	37.0(9)
C7	8402(4)	3975.7(15)	5462(4)	34.2(9)
C8	9715(4)	3748.8(17)	6048(4)	39.2(9)
C9	10210(4)	3436.4(16)	7572(5)	43(1)
C10	9371(4)	3350.4(16)	8509(4)	39.2(9)
C11	8077(4)	3572.5(16)	8021(5)	41.9(9)
C12	7600(3)	3888.8(16)	6496(4)	35.5(9)
C13	6719(3)	3917.6(17)	2518(4)	35.5(9)
C14	5703(4)	4220.5(19)	1278(4)	48.3(10)
C15	4716(4)	3867.2(19)	156(5)	51.9(11)
C16	4700(4)	3199.8(19)	250(5)	43.4(10)
C17	5714(4)	2889.3(18)	1461(5)	50.5(11)
C18	6718(4)	3251.5(18)	2572(5)	46.1(10)
C19	3501(4)	2214(2)	-694(6)	69.3(13)
N1	7234(3)	5502.8(17)	4207(4)	53.4(9)
N2	9864(5)	3004.6(16)	10127(5)	57.8(10)
O1	3643(3)	2895.6(13)	-877(3)	60.2(8)
O2	9117(4)	2939.5(17)	10959(4)	95.9(13)
O3	10981(4)	2787.3(19)	10528(4)	100.5(13)

Table 8 Anisotropic Displacement Parameters (Å2×103) for lft-1-19(compound 12s). The Anisotropic displacement factor exponent takes the form: -2π2[h2a*2U11+2hka*b*U12+…].

Atom	U11	U22	U33	U23	U13	U12
Br1	37.9(2)	54.9(2)	62.1(3)	5.9(2)	20.62(19)	5.7(2)
C1	69(3)	82(3)	52(3)	16(3)	19(2)	-3(3)
C2	52(3)	56(3)	43(3)	-7(2)	22(2)	-12(2)
C3	44(3)	55(3)	40(3)	-7(2)	18(2)	-7(2)
C4	37(2)	55(2)	42(2)	-6.8(19)	13.3(19)	-3.9(19)
C5	33(2)	37(2)	39(2)	1.2(17)	11.0(17)	2.0(17)
C6	33(2)	46(2)	30(2)	6.1(18)	8.3(17)	-1.7(19)
C7	34(2)	33(2)	34(2)	-4.1(16)	7.0(18)	-2.1(16)
C8	34(2)	47(2)	37(2)	0.6(18)	11.0(18)	-0.2(19)
C9	36(2)	42(2)	42(2)	-2.3(19)	-1(2)	1.7(19)
C10	51(3)	28.6(19)	30(2)	-1.4(16)	2(2)	0.5(19)
C11	54(3)	36(2)	38(2)	-0.1(18)	18(2)	2(2)
C12	33(2)	33.5(19)	40(2)	-2.7(17)	11.5(18)	2.3(17)
C13	28(2)	41(2)	36(2)	0.4(17)	8.1(18)	2.7(17)
C14	45(2)	43(2)	48(3)	9(2)	2(2)	0(2)
C15	43(3)	54(3)	45(3)	13(2)	-5(2)	3(2)
C16	33(2)	53(3)	39(2)	-4(2)	4.3(19)	-2(2)
C17	46(2)	37(2)	55(3)	-4.4(19)	-4(2)	3(2)
C18	34(2)	51(3)	42(2)	-3(2)	-3.5(19)	9(2)
C19	59(3)	53(3)	78(3)	-11(2)	-6(3)	-8(2)
N1	57(2)	48(2)	56(2)	2.6(18)	17.8(18)	2.8(19)
N2	74(3)	43(2)	49(2)	5.1(18)	8(2)	3(2)
O1	45.4(17)	54.5(18)	60.0(19)	-1.4(15)	-13.8(15)	-4.4(15)
O2	141(4)	93(3)	69(2)	36(2)	57(3)	53(2)
O3	62(2)	131(3)	87(3)	60(2)	-7(2)	8(2)

Table 9 Bond Lengths for lft-1-19(compound 12s).

Atom	Atom	Length/Å	Atom	Atom	Length/Å
Br1	C12	1.892(3)	C10	C11	1.371(5)
C1	C2	1.459(5)	C10	N2	1.474(5)
C2	C3	1.173(5)	C11	C12	1.380(5)
C3	C4	1.463(5)	C13	C14	1.385(5)
C4	C5	1.569(5)	C13	C18	1.372(5)
C5	C6	1.479(5)	C14	C15	1.372(5)
C5	C7	1.550(5)	C15	C16	1.376(5)
C5	C13	1.531(5)	C16	C17	1.379(5)
C6	N1	1.138(4)	C16	O1	1.365(4)
C7	C8	1.392(5)	C17	C18	1.387(5)
C7	C12	1.394(5)	C19	O1	1.424(4)
C8	C9	1.378(5)	N2	O2	1.206(4)
C9	C10	1.359(5)	N2	O3	1.202(4)

Table 10 Bond Angles for lft-1-19(compound 12s).

Atom	Atom	Atom	Angle/˚	Atom	Atom	Atom	Angle/˚
C3	C2	C1	179.4(5)	C10	C11	C12	118.4(3)
C2	C3	C4	176.8(4)	C7	C12	Br1	123.0(3)
C3	C4	C5	113.3(3)	C11	C12	Br1	115.8(3)
C6	C5	C4	104.7(3)	C11	C12	C7	121.2(3)
C6	C5	C7	107.6(3)	C14	C13	C5	121.5(3)
C6	C5	C13	112.3(3)	C18	C13	C5	120.6(3)
C7	C5	C4	111.3(3)	C18	C13	C14	117.8(3)
C13	C5	C4	109.0(3)	C15	C14	C13	121.2(4)
C13	C5	C7	111.8(3)	C14	C15	C16	120.7(4)
N1	C6	C5	174.7(4)	C15	C16	C17	119.0(4)
C8	C7	C5	120.8(3)	O1	C16	C15	116.1(3)
C8	C7	C12	117.5(3)	O1	C16	C17	124.9(3)
C12	C7	C5	121.8(3)	C16	C17	C18	119.8(3)
C9	C8	C7	122.0(3)	C13	C18	C17	121.5(3)
C10	C9	C8	117.9(4)	O2	N2	C10	118.6(4)
C9	C10	C11	122.9(3)	O3	N2	C10	118.0(4)
C9	C10	N2	119.2(4)	O3	N2	O2	123.4(4)
C11	C10	N2	117.9(4)	C16	O1	C19	117.8(3)

Table 11 Hydrogen Atom Coordinates (Å×104) and Isotropic Displacement Parameters (Å2×103) for lft-1-19(compound 12s).

Atom	x	y	z	U(eq)
H1A	7074	5389	-2101	101
H1B	8592	5427	-1984	101
H1C	7973	5953	-1089	101
H4A	9759	4612	3687	53
H4B	9300	3974	2645	53
H8	10275	3810	5390	47
H9	11090	3289	7948	52
H11	7533	3512	8699	50
H14	5690	4671	1204	58
H15	4050	4081	-677	62
H17	5725	2438	1533	61
H18	7407	3038	3373	55
H19A	2664	2071	-1462	104
H19B	3521	2120	437	104
H19C	4224	1990	-935	104

Computational details and xyz coordinates

All calculations were performed in Gaussian 09. Geometry optimizations were carried out with M06-2X/6-31+G(d,p), which is highly accurate for small moleulcar organic strucutres. Energies and optimizations were performed with the SMD continuum solvent model for DMSO. Vibrational frequencies were calculated to verify stationary points as minima or first-order saddle points (transition states). Intrinsic reaction coordinate (IRC) calculations were used to verify connection between transition states and intermediates. Solvated single point energies were carried out with M06-2X/def2-TZVPD downloaded from the EMSL basis set exchange. Enthalpies reported are the sum of $E_{(large)} + \Delta E_{ZPE(small)} + \Delta U_{vib(small)} + \Delta U_{rot(small)} + \Delta U_{trans(small)}$ + nRT + $\Delta G_{solv(large)}$. E is the total SCF energy. $\Delta E_{ZPE(small)}$ is the zero-point energy correction. $\Delta U_{vib(small)}$, $\Delta U_{rot(small)}$, and $\Delta U_{trans(small)}$ are thermal energy vibrational, rotational, and translational corrections at 298 K. R is the gas constant. No concentration corrections were applied. $\Delta G_{solv(large)}$ is the standard state solvation free energy change.

Because C-H oxidation occurs with triplet oxygen, after the hydrogen atom abstraction transition state a pair of radicals are formed and then rebound to form a C-O bond on the singlet potential energy surface.

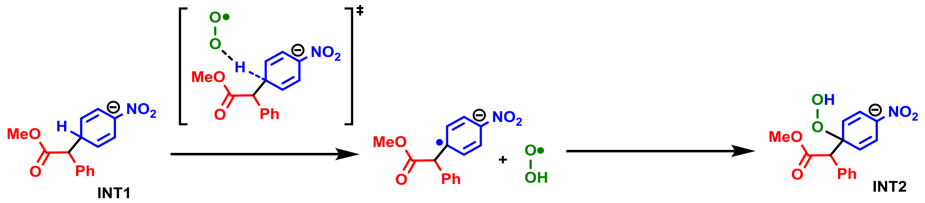

Relative pK_a value estimates were based on M06-2X/def2-TZVP//M06-2X/6-31+G(d,p) free energies (which include $-T\Delta S$ corrections) of enolates using the reaction equation shown below. The amide enolate was set to the reference pK_a value of 26.6. The calculated pK_a value the ester enolate was 22. For $PhCH_2CN$, the calculated pK_a value was 19. For $PhSO_2Me$, the calculated pK_a value was 30.

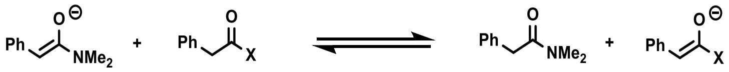

Full G09 Reference: Frisch, M. J.; Trucks, G. W.; Schlegel, H. B.; Scuseria, G. E.; Robb, M. A.; Cheeseman, J. R.; Scalmani, G.; Barone, V.; Mennucci, B.; Petersson, G. A.; Nakatsuji, H.; Caricato, M.; Li, X.; Hratchian, H. P.; Izmaylov, A. F.; Bloino, J.; Zheng, G.; Sonnenberg, J. L.; Hada, M.; Ehara, M.; Toyota, K.; Fukuda, R.; Hasegawa, J.; Ishida, M.; Nakajima, T.; Honda, Y.; Kitao, O.; Nakai, H.; Vreven, T.; Montgomery, J. A.; Peralta, J. E.; Ogliaro, F.; Bearpark, M.; Heyd, J. J.; Brothers, E.; Kudin, K. N.; Staroverov, V. N.; Kobayashi, R.; Normand, J.; Raghavachari, K.; Rendell, A.; Burant, J. C.; Iyengar, S. S.; Tomasi, J.; Cossi, M.; Rega, N.; Millam, J. M.; Klene, M.; Knox, J. E.; Cross, J. B.; Bakken, V.; Adamo, C.; Jaramillo, J.; Gomperts, R.; Stratmann, R. E.; Yazyev, O.; Austin, A. J.; Cammi, R.; Pomelli, C.; Ochterski, J. W.; Martin, R. L.; Morokuma, K.;

Zakrzewski, V. G.; Voth, G. A.; Salvador, P.; Dannenberg, J. J.; Dapprich, S.; Daniels, A. D.; Farkas; Foresman, J. B.; Ortiz, J. V.; Cioslowski, J.; Fox, D. J., Gaussian 09, Revision B.01. Wallingford CT, 2009.

Ester enoalte

C	1.7391080	0.0181050	-0.0000940
O	1.8049930	1.2614700	-0.0000100
C	0.6074610	-0.8146110	-0.0003460
C	-0.7567710	-0.3670350	-0.0000900
C	-1.8079760	-1.3276150	0.0000150
C	-1.1653310	0.9960870	-0.0001720
C	-3.1472880	-0.9620610	0.0001370
H	-1.5448120	-2.3839500	-0.0000110
C	-2.5130150	1.3512130	-0.0000110
H	-0.4008900	1.7643080	-0.0002590
C	-3.5243390	0.3871000	0.0001800
H	-3.9094520	-1.7382290	0.0002360
H	-2.7769630	2.4068770	-0.0000720
H	-4.5713850	0.6740670	0.0004010
H	0.7855970	-1.8855090	-0.0004510
O	2.9239590	-0.7105610	0.0000770
C	4.1145440	0.0574700	0.0001930
H	4.1857580	0.6917030	0.8896090
H	4.9367820	-0.6608030	-0.0001220
H	4.1853910	0.6923560	-0.8887370

Ester CT complex

C	-2.0790370	0.8739400	0.7794300
C	-3.0406060	0.3845980	-0.1224480
H	-2.3195020	0.8119070	1.8365240
O	-3.0000370	0.3247120	-1.3652000
C	-0.4506470	-2.3265200	0.5125600
C	-0.0835920	-2.0984890	-0.8149270
C	0.4124330	-1.9772070	1.5538950
H	-1.4141460	-2.7746490	0.7369390
C	1.1456830	-1.5175520	-1.1111930
H	-0.7574400	-2.3648180	-1.6223510
C	1.6455630	-1.3959690	1.2760720
H	0.1221100	-2.1479700	2.5854540
C	1.9883330	-1.1816410	-0.0563360
H	1.4442250	-1.3205720	-2.1340920
N	3.2850760	-0.5691030	-0.3599460
O	3.5734170	-0.3786420	-1.5320010
O	4.0252570	-0.2927880	0.5725470
C	-0.7848000	1.3787380	0.4198180
C	0.1281990	1.7600450	1.4446190
C	-0.3159790	1.5308090	-0.9156270
C	1.4052130	2.2272300	1.1628200
H	-0.1872420	1.6640180	2.4824420
C	0.9677910	1.9987940	-1.1859450
H	-0.9769220	1.2528710	-1.7285050
C	1.8500070	2.3465140	-0.1598420
H	2.0687070	2.4898980	1.9840620
H	1.2899730	2.0806670	-2.2223080

H	2.8543920	2.6955660	-0.3810100
H	2.3261460	-1.1096060	2.0692700
O	-4.1780630	-0.0819210	0.5287640
C	-5.1907160	-0.6068700	-0.3118030
H	-4.8387160	-1.4824630	-0.8668510
H	-6.0097370	-0.9002340	0.3478940
H	-5.5490470	0.1396970	-1.0272760

Ester C-C addition TS

C	-1.7084210	0.4570220	0.7258980
C	-2.8372640	0.2613830	-0.1498360
H	-1.9460920	0.3784870	1.7832360
O	-2.9051360	0.4834780	-1.3560550
C	-0.8892530	-1.5360210	0.5657820
C	-0.4255010	-1.6451110	-0.7910030
C	0.1156580	-1.5512630	1.5957990
H	-1.8620090	-1.9559750	0.8072780
C	0.8942670	-1.4404590	-1.0977680
H	-1.1436630	-1.8138710	-1.5875580
C	1.4354590	-1.3507510	1.2936610
H	-0.1911730	-1.6476210	2.6334080
C	1.8425960	-1.2466510	-0.0613910
H	1.2345700	-1.4505230	-2.1278750
N	3.1840010	-1.0020010	-0.3738820
O	3.5356970	-0.9492380	-1.5689380
O	4.0079120	-0.8410410	0.5480980
C	-0.5729670	1.2916920	0.3927610
C	0.2918620	1.6974420	1.4360600

C	-0.1894950	1.6144080	-0.9302120
C	1.4934840	2.3457470	1.1743470
H	0.0129860	1.4752750	2.4638050
C	1.0137400	2.2688420	-1.1845720
H	-0.8376220	1.3287390	-1.7495660
C	1.8747810	2.6205790	-0.1425510
H	2.1424020	2.6280540	1.9995520
H	1.2903410	2.4925190	-2.2120470
H	2.8186310	3.1165820	-0.3501310
O	-3.8829070	-0.3171960	0.5108490
C	-5.0201440	-0.6345430	-0.2876570
H	-5.7418060	-1.0926040	0.3889960
H	-5.4498290	0.2670170	-0.7322950
H	-4.7572780	-1.3368900	-1.0830440
H	2.1851830	-1.2970930	2.0758700

INT1

C	0.7754880	0.4251960	0.1347750
C	1.4121090	1.7872930	-0.0653160
H	0.2761250	0.4216220	1.1084640
O	1.7140520	2.2579930	-1.1421290
C	-0.3028540	0.1744640	-0.9687900
C	-0.9120690	-1.1981010	-0.8069300
C	-1.3574300	1.2563290	-0.9140690
H	0.2377550	0.2317750	-1.9249130
C	-2.1769920	-1.3967870	-0.3832810
H	-0.2778410	-2.0555310	-1.0201630
C	-2.6163310	1.0363000	-0.4826090

H	-1.0585500	2.2610430	-1.2051580
C	-3.0694100	-0.2891100	-0.1338780
H	-2.5634930	-2.4021610	-0.2500020
N	-4.3241110	-0.4962960	0.3236190
O	-4.7256140	-1.6744760	0.5849400
O	-5.1117320	0.4860870	0.5030880
C	1.8596100	-0.6381050	0.1443720
C	2.7516800	-0.7689180	-0.9270100
C	1.9463520	-1.5366950	1.2119750
C	3.7121700	-1.7794790	-0.9272040
H	2.6950720	-0.0760510	-1.7629100
C	2.9062650	-2.5496600	1.2119150
H	1.2536180	-1.4435720	2.0448220
C	3.7920420	-2.6736680	0.1419890
H	4.3992840	-1.8686320	-1.7636950
H	2.9597840	-3.2416060	2.0474470
H	4.5400620	-3.4609700	0.1401310
O	1.6295550	2.4153570	1.0942390
C	2.2560530	3.7035130	1.0025560
H	2.3292620	4.0699000	2.0252490
H	3.2509140	3.6114900	0.5616720
H	1.6458660	4.3795170	0.4003150
H	-3.3292710	1.8521300	-0.4186800

Ester hydrogen atom abstraction TS

C	-1.7084210	0.4570220	0.7258980
C	-2.8372640	0.2613830	-0.1498360
H	-1.9460920	0.3784870	1.7832360
O	-2.9051360	0.4834780	-1.3560550
C	-0.8892530	-1.5360210	0.5657820
C	-0.4255010	-1.6451110	-0.7910030
C	0.1156580	-1.5512630	1.5957990
H	-1.8620090	-1.9559750	0.8072780
C	0.8942670	-1.4404590	-1.0977680
H	-1.1436630	-1.8138710	-1.5875580
C	1.4354590	-1.3507510	1.2936610
H	-0.1911730	-1.6476210	2.6334080
C	1.8425960	-1.2466510	-0.0613910
H	1.2345700	-1.4505230	-2.1278750
N	3.1840010	-1.0020010	-0.3738820
O	3.5356970	-0.9492380	-1.5689380
O	4.0079120	-0.8410410	0.5480980
C	-0.5729670	1.2916920	0.3927610
C	0.2918620	1.6974420	1.4360600
C	-0.1894950	1.6144080	-0.9302120
C	1.4934840	2.3457470	1.1743470
H	0.0129860	1.4752750	2.4638050
C	1.0137400	2.2688420	-1.1845720
H	-0.8376220	1.3287390	-1.7495660
C	1.8747810	2.6205790	-0.1425510
H	2.1424020	2.6280540	1.9995520
H	1.2903410	2.4925190	-2.2120470

H	2.8186310	3.1165820	-0.3501310
O	-3.8829070	-0.3171960	0.5108490
C	-5.0201440	-0.6345430	-0.2876570
H	-5.7418060	-1.0926040	0.3889960
H	-5.4498290	0.2670170	-0.7322950
H	-4.7572780	-1.3368900	-1.0830440
H	2.1851830	-1.2970930	2.0758700

Ester radical pair intermediates

C	1.1809230	0.5221750	-0.6567570
C	1.5832090	1.8207360	0.0297680
O	2.1942250	1.9127030	1.0714050
C	-0.3016050	0.2555660	-0.4294410
C	-1.0316720	-0.4389940	-1.4053050
C	-0.9647300	0.6392150	0.7447560
C	-2.3720190	-0.7488760	-1.2265240
H	-0.5335950	-0.7423160	-2.3241160
C	-2.3066670	0.3438970	0.9486840
H	-0.4284340	1.1821860	1.5211460
C	-3.0292070	-0.3590950	-0.0387540
H	-2.9313340	-1.2829460	-1.9849130
H	-2.8147410	0.6461760	1.8561390
N	-4.3718930	-0.6602840	0.1530240
O	-5.0091230	-1.2983420	-0.7649820
O	-4.9471410	-0.2944200	1.2443840
C	2.0649160	-0.6363240	-0.2284210
C	3.0126970	-1.1485410	-1.1175140
C	1.9469590	-1.2117950	1.0418310

C	3.8330330	-2.2168110	-0.7479400
H	3.1074610	-0.7100330	-2.1081460
C	2.7676690	-2.2735080	1.4154390
H	1.2088870	-0.8287940	1.7417260
C	3.7133070	-2.7806840	0.5206330
H	4.5612720	-2.6068340	-1.4531060
H	2.6674450	-2.7094760	2.4052240
H	4.3481400	-3.6125110	0.8112300
H	1.3276610	0.6821250	-1.7302010
O	1.1430770	2.8798180	-0.6573450
C	1.3690110	4.1602320	-0.0518090
H	0.9580220	4.8908790	-0.7466320
H	0.8519290	4.2188150	0.9087700
H	2.4372950	4.3334850	0.0921570

INT2

C	-1.4123490	-0.1878550	-0.5283920
C	-2.4897750	-0.8149300	0.3398960
O	-2.4631140	-0.9006960	1.5498170
C	-0.0531910	-0.9530560	-0.3967820
C	0.8935070	-0.5115220	-1.4703200
C	0.5724990	-0.9156950	0.9652210
C	2.2011030	-0.2536860	-1.2462920
H	0.4836360	-0.4315040	-2.4751300
C	1.8904970	-0.6661150	1.1601460
H	-0.0729430	-1.1329760	1.8109860
C	2.7588580	-0.3397480	0.0717610
H	2.8517100	0.0352640	-2.0655420

H	2.3062730	-0.6889980	2.1625870
N	4.0857330	-0.0772330	0.2908180
O	4.8343860	0.2253540	-0.6752610
O	4.5566210	-0.1413440	1.4571260
O	-0.5317030	-2.3247980	-0.6681140
O	0.5609080	-3.2288120	-0.6405900
H	0.8644100	-3.1633510	0.2843630
C	-1.2681090	1.2983560	-0.2605420
C	-1.2594820	2.1821550	-1.3452020
C	-1.1097150	1.8134500	1.0318660
C	-1.0936470	3.5531460	-1.1481310
H	-1.3831870	1.7915550	-2.3522300
C	-0.9470400	3.1844830	1.2298370
H	-1.1210280	1.1418700	1.8839290
C	-0.9363690	4.0584590	0.1419980
H	-1.0894340	4.2238190	-2.0023590
H	-0.8272820	3.5697060	2.2382940
H	-0.8080290	5.1252470	0.2997110
H	-1.7261060	-0.3129470	-1.5681640
O	-3.5165390	-1.2521370	-0.3962460
C	-4.5861560	-1.8729730	0.3300400
H	-5.3228460	-2.1600950	-0.4183330
H	-4.2205960	-2.7550440	0.8601330
H	-5.0229650	-1.1692680	1.0415610

Ester OOH anion TS

C	-1.2263950	-0.3551030	-0.5056860
C	-1.9200670	-1.4230310	0.3205970
O	-1.7913910	-1.5944890	1.5152630
C	0.2793520	-0.3224840	-0.3144900
C	1.0482030	0.2263160	-1.3742710
C	0.8941960	-0.4134330	0.9585670
C	2.3805940	0.5376360	-1.2159080
H	0.5759610	0.3740110	-2.3418990
C	2.2341890	-0.1074040	1.1282470
H	0.3145730	-0.7596430	1.8060500
C	2.9788090	0.3617560	0.0420350
H	2.9638490	0.9284200	-2.0420160
H	2.7032080	-0.2090370	2.1007180
N	4.3638410	0.6884470	0.2208980
O	5.0010780	1.1101860	-0.7445950
O	4.8717220	0.5354600	1.3321720
O	0.1683910	-2.4544860	-0.9050610
O	1.4522410	-2.9857980	-0.5339970
H	1.6428050	-2.5123400	0.2925830
C	-1.8553030	1.0059330	-0.2222550
C	-2.3898250	1.7539360	-1.2749100
C	-1.8843420	1.5382130	1.0726130
C	-2.9427400	3.0145550	-1.0422800
H	-2.3707960	1.3472010	-2.2831090
C	-2.4389810	2.7952630	1.3065970
H	-1.4748570	0.9640460	1.8994020
C	-2.9687110	3.5382570	0.2493940

H	-3.3528240	3.5844250	-1.8709670
H	-2.4566600	3.1960370	2.3159380
H	-3.3984880	4.5185640	0.4328760
H	-1.4064150	-0.5980010	-1.5544610
O	-2.8159500	-2.0899920	-0.4152810
C	-3.5497110	-3.1064200	0.2744540
H	-4.2175360	-3.5457300	-0.4653680
H	-2.8669890	-3.8645420	0.6652480
H	-4.1270470	-2.6735160	1.0944780

Ester arylation product

C	1.0567430	0.5343710	0.6935440
C	1.6009000	1.7715550	-0.0104420
O	1.0487110	2.3789640	-0.8997730
C	-0.8827060	-0.0050440	-0.8516070
C	-1.3404700	0.4960010	1.4673370
C	-2.2369620	-0.1943420	-1.0892130
H	-0.1791060	-0.1324140	-1.6686580
C	-2.7025440	0.3059660	1.2522840
H	-0.9900220	0.7643970	2.4595820
C	-3.1226540	-0.0357920	-0.0262880
H	-2.6013550	-0.4617390	-2.0738350
N	-4.5547170	-0.2431350	-0.2651890
O	-5.3205710	-0.1118650	0.6774360
O	-4.9128570	-0.5378800	-1.3952370
C	-0.4230210	0.3430790	0.4249200
C	1.8620220	-0.7057730	0.3171350
C	2.5413630	-0.8122100	-0.8993350

C	1.8766040	-1.7875640	1.2034110
C	3.2255120	-1.9845040	-1.2246490
H	2.5441700	0.0166800	-1.6047490
C	2.5586510	-2.9585090	0.8791880
H	1.3490000	-1.7079190	2.1512500
C	3.2357220	-3.0597630	-0.3373390
H	3.7516620	-2.0537770	-2.1720180
H	2.5644710	-3.7897370	1.5780390
H	3.7700760	-3.9705170	-0.5902620
H	1.1915720	0.7057610	1.7667760
O	2.7956020	2.1044290	0.4809580
C	3.4451640	3.2210660	-0.1462450
H	4.3960430	3.3352350	0.3711950
H	3.6116820	3.0128340	-1.2053930
H	2.8405040	4.1232850	-0.0361880
H	-3.4187030	0.4194540	2.0572930

Amide enolate

C	-1.4239760	-0.3506970	-0.1134090
O	-1.2805270	-1.6038200	-0.0815790
C	-3.0350470	1.5457860	-0.0322230
H	-4.0954440	1.7236150	-0.2300830
H	-2.8193950	1.8760170	0.9977650
H	-2.4621270	2.1696020	-0.7218240
C	1.0334820	0.2695080	-0.0206030
C	1.9820400	1.3313960	0.0418350
C	1.5827600	-1.0436000	-0.0413270
C	3.3513060	1.1069010	0.0773340

H	1.6124870	2.3553500	0.0607810
C	2.9602320	-1.2572460	-0.0070440
H	0.8995200	-1.8827110	-0.0840660
C	3.8665080	-0.1959590	0.0525670
H	4.0282870	1.9571020	0.1239650
H	3.3318290	-2.2799440	-0.0266920
H	4.9372100	-0.3740900	0.0794720
C	-0.3690160	0.5905390	-0.0569840
H	-0.5997630	1.6485570	-0.0438020
N	-2.7536780	0.1378720	-0.2434940
C	-3.7970520	-0.7362350	0.2674710
H	-3.8753610	-0.6887560	1.3675440
H	-4.7603850	-0.4366890	-0.1549890
H	-3.5843230	-1.7649570	-0.0166830

Amide TS

C	-1.5250960	0.4719880	0.5794290
C	-2.6085850	0.3707850	-0.3837820
H	-1.7787110	0.3001780	1.6193510
O	-2.5345460	0.7980850	-1.5531640
C	-0.7004800	-1.5553890	0.2617760
C	-0.1531160	-1.5080350	-1.0625120
C	0.2258240	-1.6927830	1.3488930
H	-1.6996960	-1.9640260	0.3841410
C	1.1872870	-1.2961800	-1.2578330
H	-0.8207740	-1.5636290	-1.9166700
C	1.5668250	-1.4866950	1.1593780
H	-0.1532920	-1.8884850	2.3482220

C	2.0646880	-1.2461150	-0.1461770
H	1.5959470	-1.1917900	-2.2572460
N	3.4302950	-1.0052150	-0.3411890
O	3.8607660	-0.8187540	-1.4962250
O	4.1955140	-0.9869650	0.6428860
C	-0.3485960	1.2915060	0.3924360
C	0.4609490	1.5613250	1.5247550
C	0.1394040	1.7331500	-0.8628360
C	1.6949050	2.1908340	1.4115420
H	0.1080680	1.2485590	2.5053710
C	1.3742780	2.3701260	-0.9675830
H	-0.4615630	1.5547470	-1.7452030
C	2.1733150	2.5861530	0.1578880
H	2.2927140	2.3659890	2.3025560
H	1.7262270	2.6859200	-1.9470090
H	3.1413340	3.0700260	0.0637060
C	-4.9020890	-0.2820540	-0.8632940
H	-5.5110290	-1.1755140	-0.7046120
H	-5.5235760	0.6030250	-0.6597990
H	-4.5785430	-0.2463210	-1.9018060
N	-3.7398080	-0.3347610	0.0080030
C	-4.0261310	-0.6236060	1.4053000
H	-4.2309090	0.2865080	1.9871090
H	-4.9106400	-1.2623630	1.4497070
H	-3.2052510	-1.1648020	1.8813010
H	2.2621160	-1.5271700	1.9909260

Cyano enolate

C	-0.1676240	-0.4151120	-0.0000530
C	0.9438750	-1.3000870	-0.0001370
C	0.1364830	0.9724840	-0.0000520
C	2.2520120	-0.8318270	0.0000450
H	0.7575700	-2.3720580	-0.0002660
C	1.4499920	1.4307300	-0.0000290
H	-0.6785180	1.6932890	-0.0001190
C	2.5287580	0.5405920	0.0000870
H	3.0699830	-1.5487320	0.0000270
H	1.6339110	2.5027480	-0.0000320
H	3.5517690	0.9032180	0.0002160
C	-1.5093080	-0.9250290	0.0001320
H	-1.6832800	-1.9952620	0.0002790
C	-2.6138780	-0.0874660	-0.0000270
N	-3.5390420	0.6444410	0.0000140

Cyano TS

C	2.5971850	0.3639980	0.5702170
C	1.2993230	-1.1088810	1.5304980
C	0.4727960	-0.3017920	2.3769290
C	0.6450920	-1.7789130	0.4483160
H	2.1716330	-1.5910610	1.9607480
C	-0.7853470	0.0510100	1.9781400
H	0.8751510	0.0922230	3.3050690
C	-0.6141620	-1.4144640	0.0416680
H	1.1743950	-2.5471330	-0.1059420
C	-1.3638610	-0.4493460	0.7783730

H	-1.3873140	0.7176860	2.5844280
N	-2.6400800	0.0334570	0.4233830
O	-3.1958960	0.8506470	1.1796910
O	-3.1909620	-0.3512510	-0.6162990
Cl	-1.2387700	-2.2669650	-1.3524830
C	1.6433820	1.1590250	-0.1782200
C	1.1974760	0.7804930	-1.4632070
C	1.0152020	2.2607370	0.4413410
C	0.1453700	1.4506100	-2.0786530
H	1.6700480	-0.0583540	-1.9696000
C	-0.0373430	2.9263000	-0.1790250
H	1.3542880	2.5770630	1.4248570
C	-0.4930850	2.5147910	-1.4346840
H	-0.1903280	1.1313530	-3.0619230
H	-0.5139970	3.7632040	0.3245900
H	-1.3233220	3.0267430	-1.9122770
H	3.0219470	0.7973780	1.4718640
C	3.4560620	-0.5173430	-0.1229970
N	4.1019250	-1.3167720	-0.6789410

(Methylsulfonyl)benzene enolate

C	-2.1434970	-0.0665850	1.5068450
H	-1.8327200	0.8199190	2.0638810
H	-1.8301740	-0.9989330	1.9817920
S	-1.5277110	0.0037290	-0.0638530
O	-1.8839760	-1.2304980	-0.8101840
O	-1.8791920	1.3010860	-0.6965640
C	0.2860810	0.0022030	-0.0781230

C	0.9782870	1.2120810	-0.0364940
C	0.9738130	-1.2111630	-0.0443490
C	2.3714170	1.2060450	0.0385030
H	0.4271280	2.1470780	-0.0741760
C	2.3663410	-1.2111550	0.0304330
H	0.4203130	-2.1448660	-0.0872950
C	3.0665340	-0.0037860	0.0740090
H	2.9139200	2.1466240	0.0647540
H	2.9054190	-2.1538230	0.0509060
H	4.1509770	-0.0062010	0.1308350

(Methylsulfonyl)benzene enolate addition TS

C	0.6026160	1.1177380	1.0376600
H	1.1473310	2.0614930	1.0934460
C	-1.6057250	1.7796550	1.0435110
C	-2.0683230	1.8521790	-0.3049610
C	-2.2601300	0.8578590	1.9164590
H	-1.1473860	2.6589800	1.4769760
C	-2.9456530	0.9189770	-0.8014280
H	-1.6832240	2.6281150	-0.9582230
C	-3.1332420	-0.0831570	1.4245570
H	-2.0298040	0.8734280	2.9773380
C	-3.4556150	-0.0805920	0.0528320
H	-3.2706240	0.9529480	-1.8354030
N	-4.3674980	-1.0419130	-0.4557400
O	-4.6717530	-1.0062240	-1.6546710
O	-4.8336950	-1.8940350	0.3112670
H	0.6617490	0.5193130	1.9477830

S	1.1290430	0.2231880	-0.3131010
O	0.4408200	-1.0884970	-0.3489580
O	1.0542440	1.0777280	-1.5211940
C	2.8819450	-0.1937440	-0.1622900
C	3.8412150	0.6764410	-0.6793120
C	3.2574540	-1.3432850	0.5333550
C	5.1939370	0.3876100	-0.5006740
H	3.5273620	1.5622190	-1.2235720
C	4.6116200	-1.6250570	0.7079440
H	2.4954780	-2.0122260	0.9229770
C	5.5799630	-0.7607810	0.1926120
H	5.9460370	1.0571450	-0.9074200
H	4.9108290	-2.5218460	1.2423770
H	6.6338490	-0.9840720	0.3288450
H	-3.6010920	-0.8079600	2.0815510

(Nitromethyl)benzene enolate

C	1.1563460	-0.7646130	0.0000550
C	-0.2178170	-0.3143490	0.0001140
C	-0.6669680	1.0262610	0.0001270
C	-1.2113260	-1.3254480	-0.0000150
C	-2.0314920	1.3209670	0.0000470
H	0.0626870	1.8241130	0.0002390
C	-2.5656340	-1.0207940	-0.0000940
H	-0.8977930	-2.3670040	0.0000010
C	-2.9929580	0.3117200	-0.0000250
H	-2.3429550	2.3626060	0.0000580
H	-3.2945250	-1.8269940	-0.0001400

H	-4.0515010	0.5533960	-0.0001560
H	1.3669110	-1.8251600	-0.0000110
N	2.2492240	0.0047940	-0.0000450
O	2.1699220	1.2760780	-0.0001790
O	3.4040410	-0.5457000	0.0000630

(Nitromethyl)benzene enolate addition TS

C	2.0258040	0.0627230	0.5862260
C	1.0001080	-1.6399070	0.6390710
C	0.0177560	-1.4347750	1.6845970
C	0.4720000	-1.8629330	-0.6899430
H	1.8933330	-2.2029210	0.9125310
C	-1.2799300	-1.1296050	1.3838660
H	0.3431290	-1.4526190	2.7207790
C	-0.8257890	-1.5452830	-0.9903960
H	1.1319500	-2.2297750	-1.4694030
C	-1.7118190	-1.1273780	0.0327870
H	-1.9977170	-0.9163350	2.1687850
N	-3.0372910	-0.7955890	-0.2801550
O	-3.8198800	-0.4798440	0.6346580
O	-3.4076300	-0.8162280	-1.4681410
C	1.0901430	1.1371800	0.2347800
C	0.6513990	1.4559640	-1.0642930
C	0.4928350	1.8083740	1.3196850
C	-0.3456660	2.4124990	-1.2559060
H	1.0859210	0.9527680	-1.9169950
C	-0.5071100	2.7554270	1.1223130
H	0.8184070	1.5732180	2.3301920

C	-0.9363650	3.0602110	-0.1709160
H	-0.6698920	2.6438690	-2.2668650
H	-0.9521140	3.2544350	1.9784280
H	-1.7175370	3.7976840	-0.3302140
H	2.3833670	0.0662700	1.6101000
H	-1.2015190	-1.6483680	-2.0029930
N	3.0594830	-0.3375590	-0.2720360
O	2.9566920	-0.1722710	-1.4987180
O	4.0042080	-0.9843040	0.2246710

^{1}H and ^{13}C NMR of Products

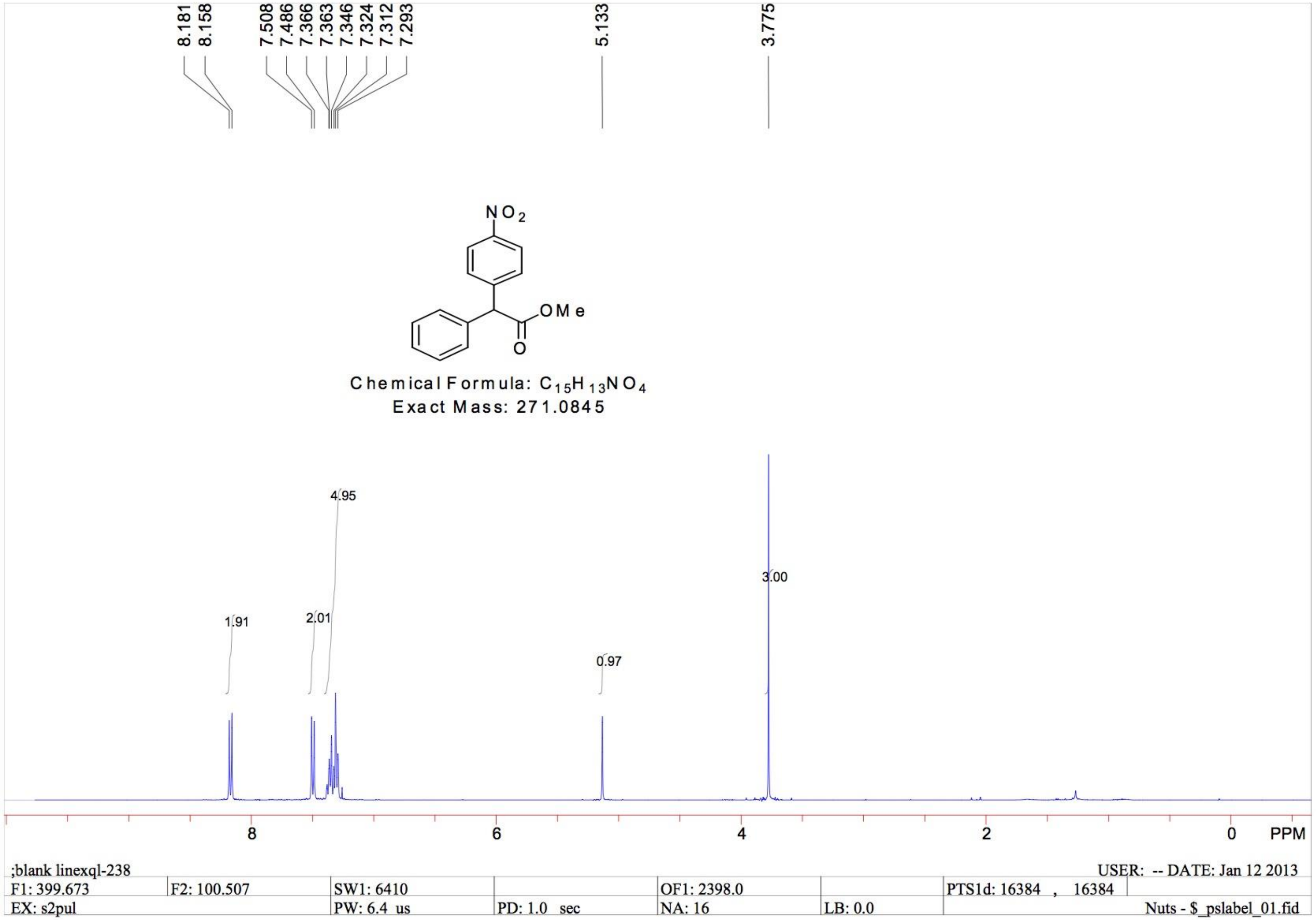

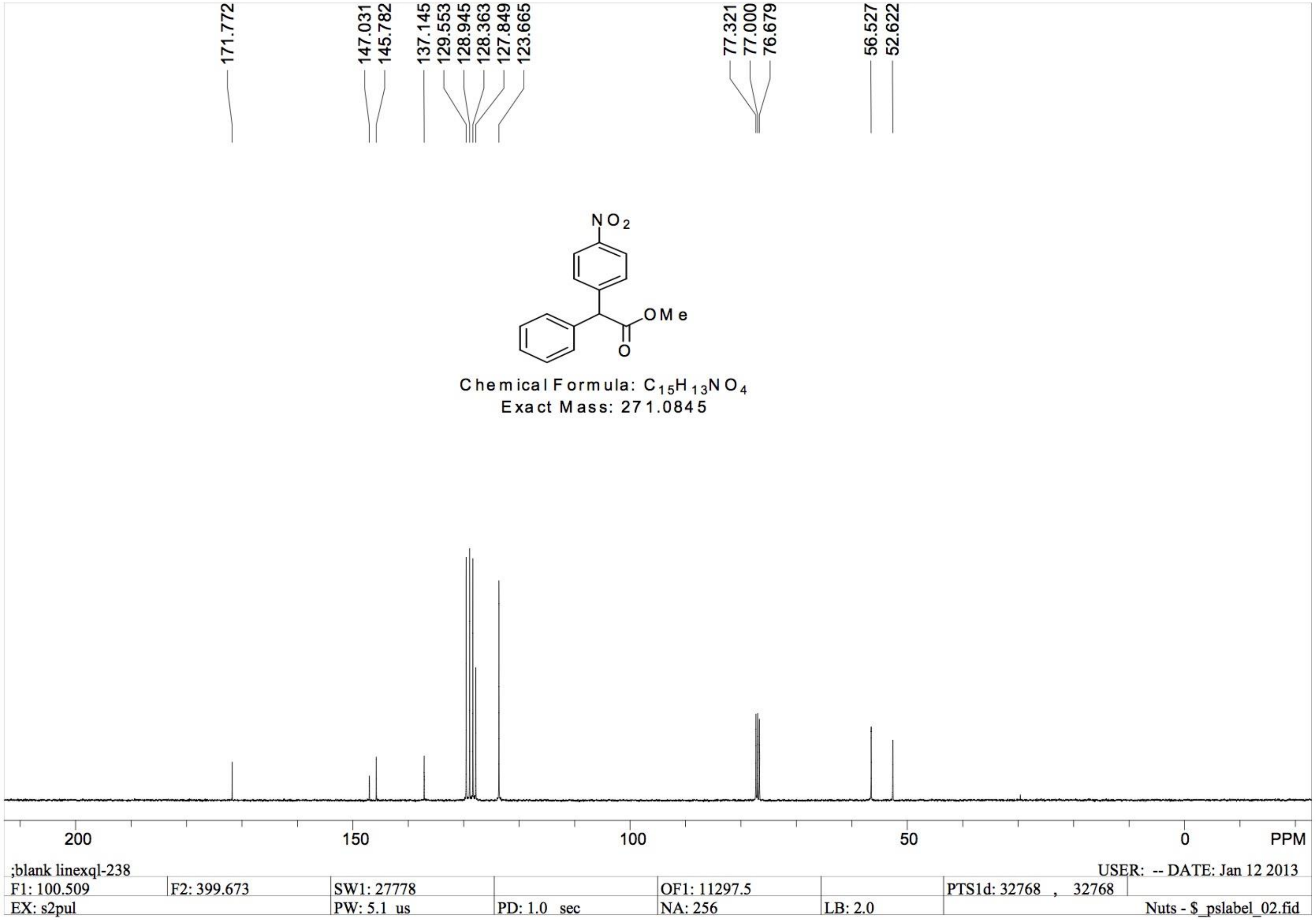
171.772
147.031
145.782
137.145
129.553
128.945
128.363
127.849
123.665
77.321
77.000
76.679
56.527
52.622
NO2
OMe
O
Chemical Formula: C15H13NO4
Exact Mass: 271.0845
200
150
100
50
0
PPM
;blank linexql-238
USER: -- DATE: Jan 12 2013
F1: 100.509
F2: 399.673
SW1: 27778
OF1: 11297.5
PTS1d: 32768 , 32768
EX: s2pul
PW: 5.1 us
PD: 1.0 sec
NA: 256
LB: 2.0
Nuts - $_pslabel_02.fid

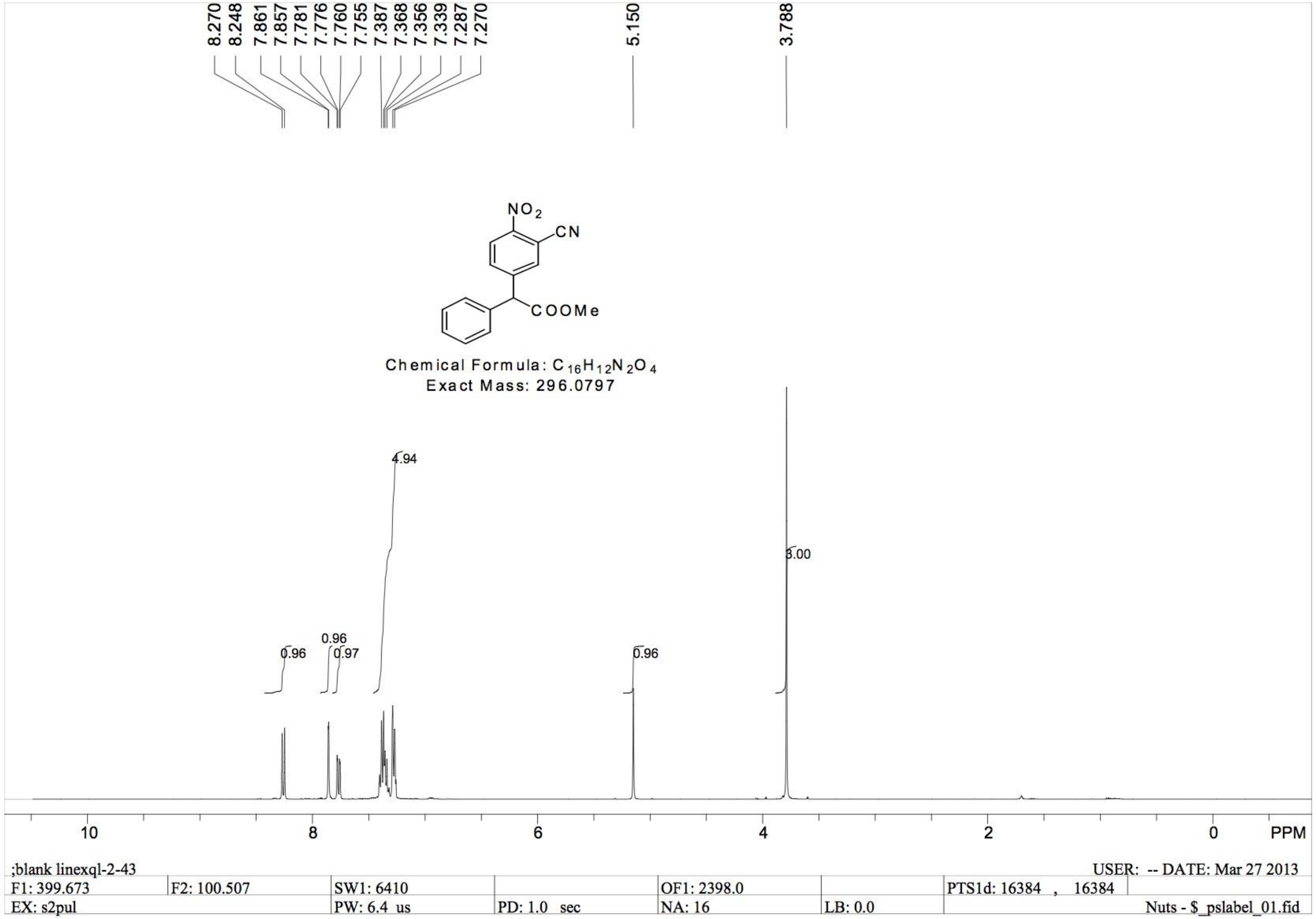

8.270
8.248
7.861
7.857
7.781
7.776
7.760
7.755
7.387
7.368
7.356
7.339
7.287
7.270
5.150
3.788
NO2
CN
COOMe
Chemical Formula: C16H12N2O4
Exact Mass: 296.0797
4.94
3.00
0.96
0.96
0.97
0.96
10
8
6
4
2
0
PPM
;blank linexql-2-43
USER: -- DATE: Mar 27 2013
F1: 399.673
F2: 100.507
SW1: 6410
OF1: 2398.0
PTS1d: 16384 , 16384
EX: s2pul
PW: 6.4 us
PD: 1.0 sec
NA: 16
LB: 0.0
Nuts - $_pslabel_01.fid

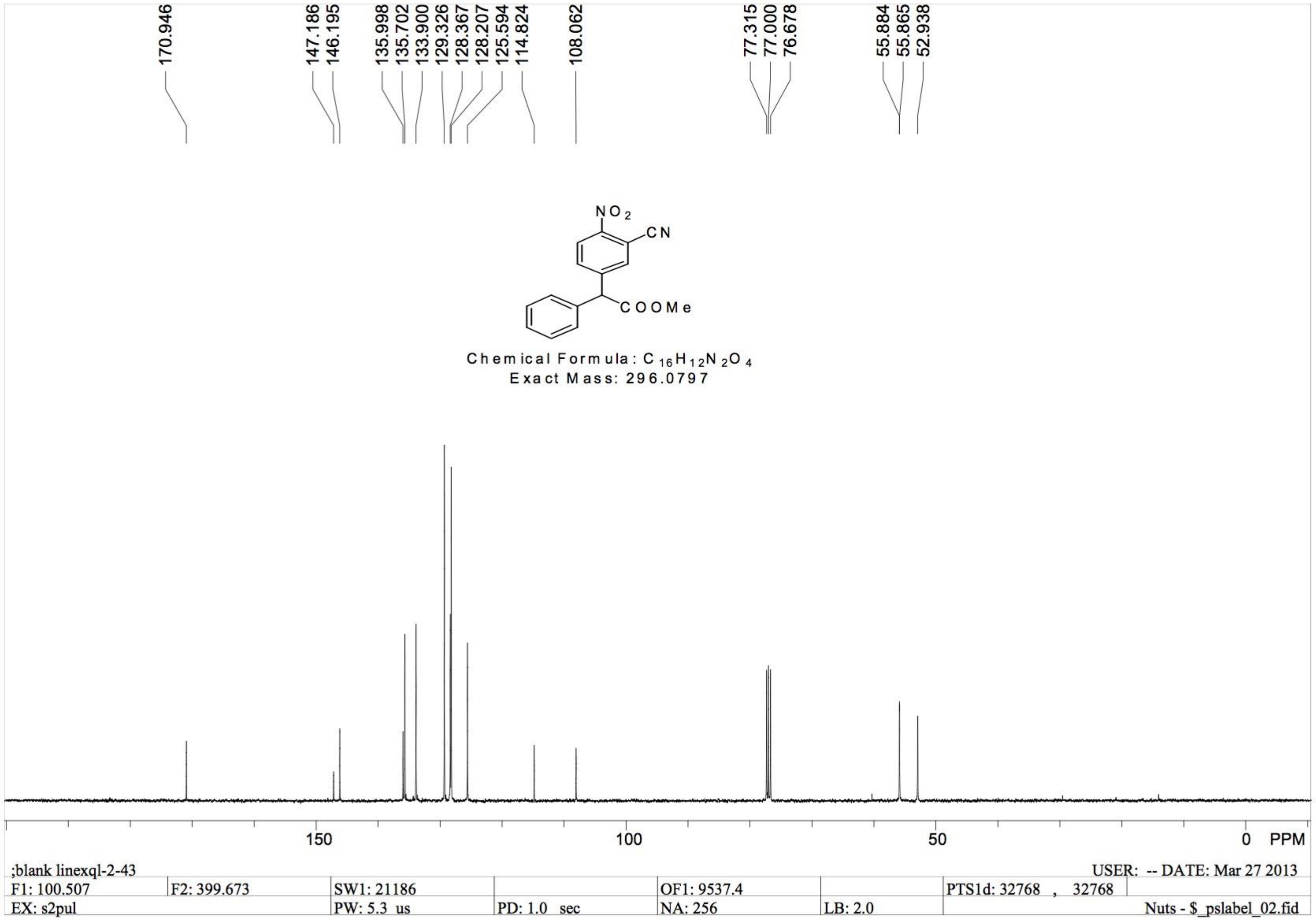
170.946
147.186
146.195
135.998
135.702
133.900
129.326
128.367
128.207
125.594
114.824
108.062
77.315
77.000
76.678
55.884
55.865
52.938
NO2
CN
COOMe
Chemical Formula: C16H12N2O4
Exact Mass: 296.0797
150
100
50
0 PPM
;blank linexql-2-43
USER: -- DATE: Mar 27 2013
F1: 100.507
F2: 399.673
SW1: 21186
OF1: 9537.4
PTS1d: 32768 , 32768
EX: s2pul
PW: 5.3 us
PD: 1.0 sec
NA: 256
LB: 2.0
Nuts - $_pslabel_02.fid

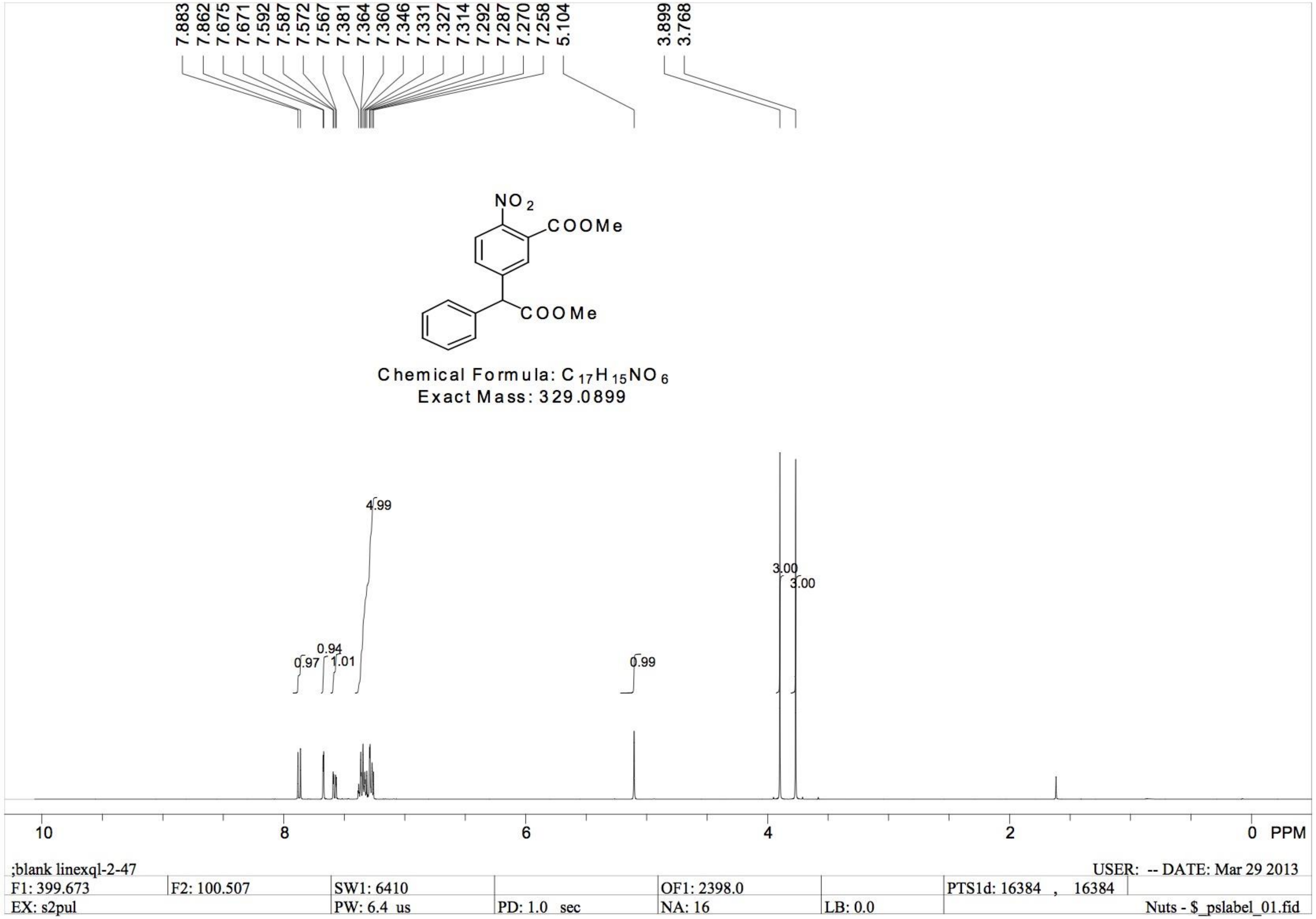
7.883
7.862
7.675
7.671
7.592
7.587
7.572
7.567
7.381
7.364
7.360
7.346
7.331
7.327
7.314
7.292
7.287
7.270
7.258
5.104
3.899
3.768
NO2
COOMe
COOMe
Chemical Formula: C17H15NO6
Exact Mass: 329.0899
4.99
0.94
0.97
1.01
0.99
3.00
3.00
10
8
6
4
2
0 PPM
;blank linexql-2-47
USER: -- DATE: Mar 29 2013
F1: 399.673
F2: 100.507
SW1: 6410
OF1: 2398.0
PTS1d: 16384 , 16384
EX: s2pul
PW: 6.4 us
PD: 1.0 sec
NA: 16
LB: 0.0
Nuts - $_pslabel_01.fid

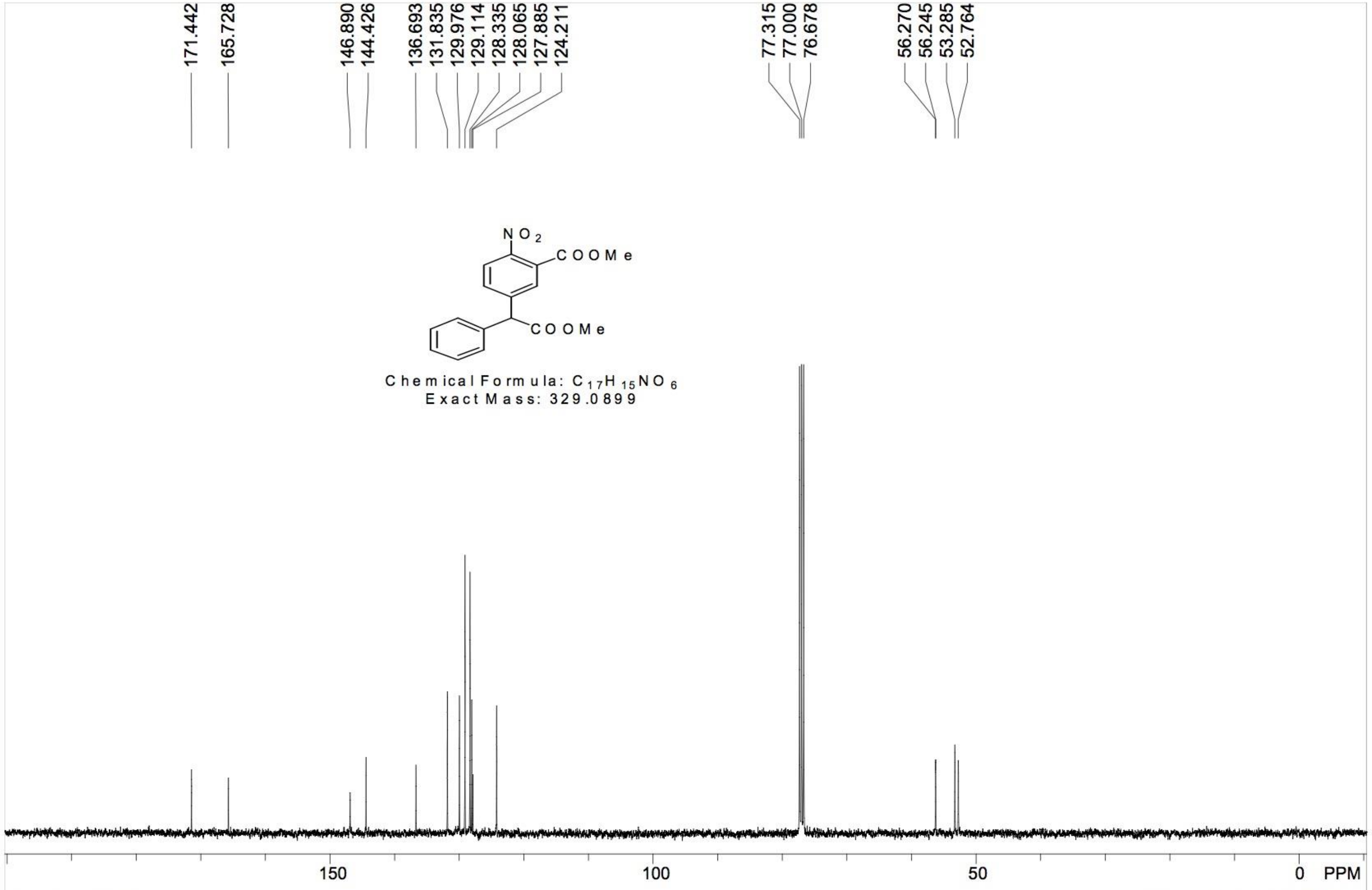

;blank linexql-2-47 USER: -- DATE: Mar 29 2013

F1: 100.507	F2: 399.673	SW1: 21186		OF1: 9543.2		PTS1d: 32768 , 32768	
EX: s2pul		PW: 5.3 us	PD: 5.0 sec	NA: 64	LB: 2.0		Nuts - $_pslabel_02.fid

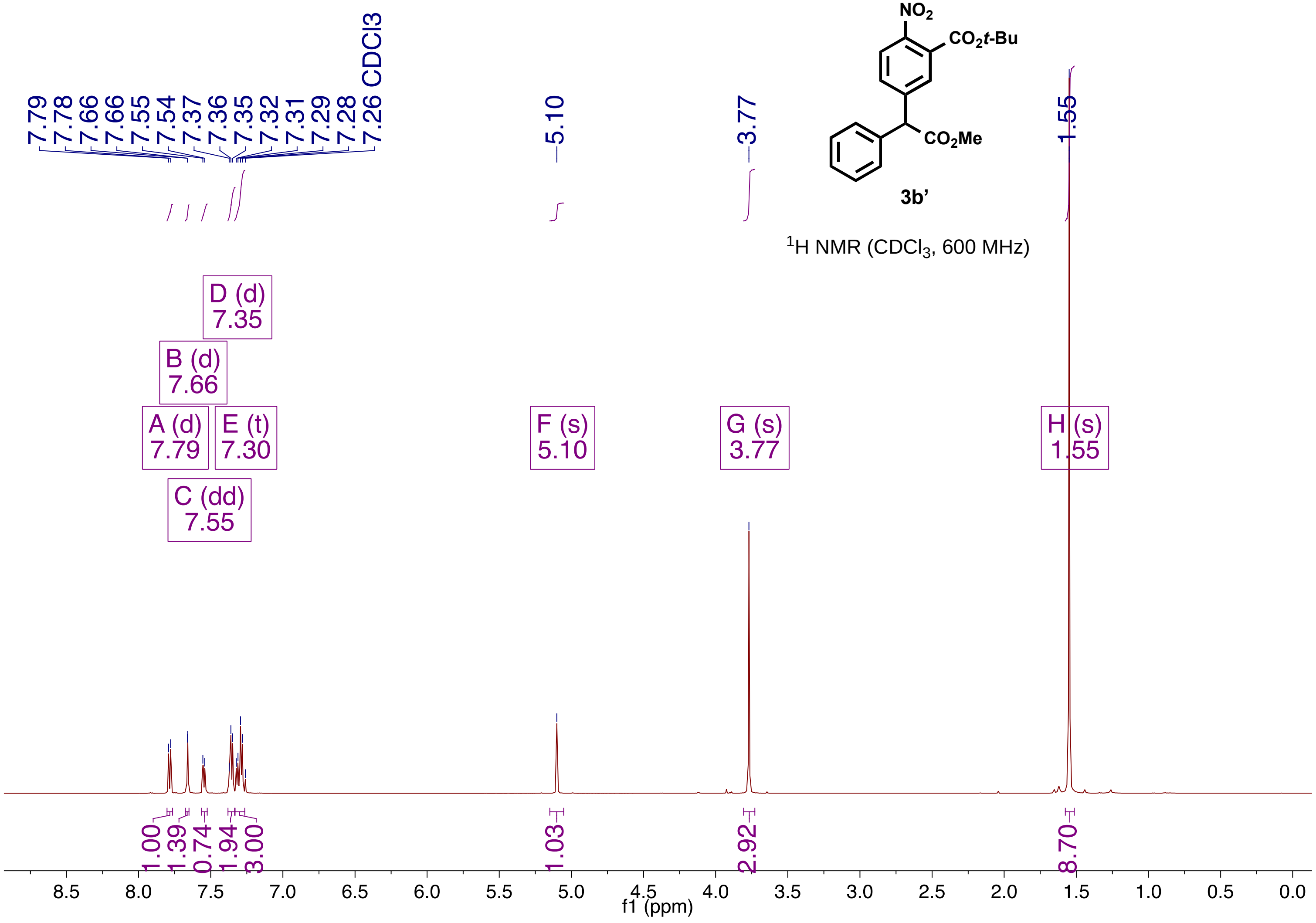
7.79
7.78
7.66
7.66
7.55
7.54
7.37
7.36
7.35
7.32
7.31
7.29
7.28
7.26 CDCl3
5.10
3.77
1.55
NO2
CO2t-Bu
CO2Me
3b'
1H NMR (CDCl3, 600 MHz)
D (d) 7.35
B (d) 7.66
A (d) 7.79
E (t) 7.30
C (dd) 7.55
F (s) 5.10
G (s) 3.77
H (s) 1.55
1.00
1.39
0.74
1.94
3.00
1.03
2.92
8.70
8.5 8.0 7.5 7.0 6.5 6.0 5.5 5.0 4.5 4.0 3.5 3.0 2.5 2.0 1.5 1.0 0.5 0.0
f1 (ppm)

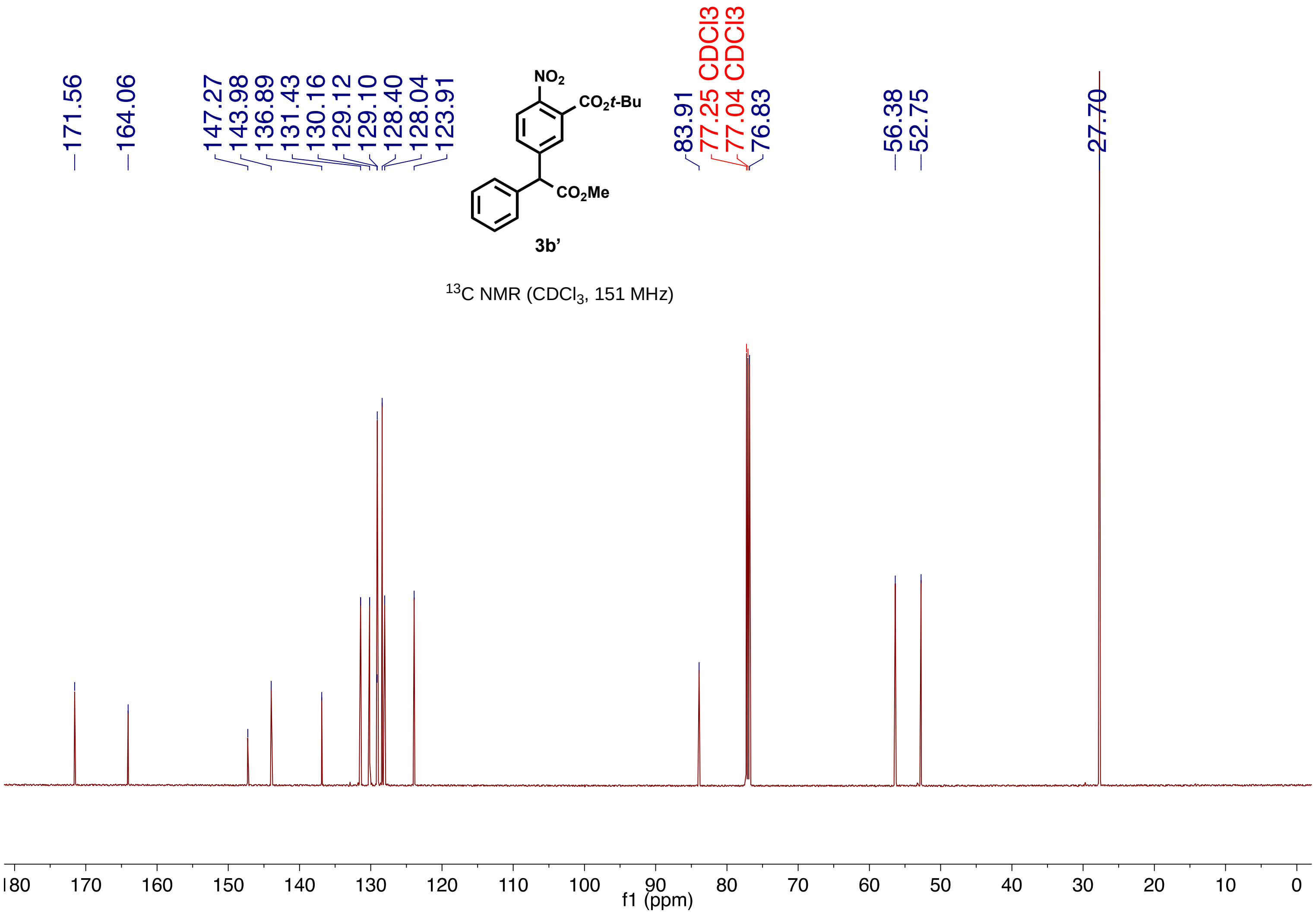

171.56
164.06
147.27
143.98
136.89
131.43
130.16
129.12
129.10
128.40
128.04
123.91
NO_2
CO_2t-Bu
CO_2Me
3b'
83.91
77.25 CDCl3
77.04 CDCl3
76.83
56.38
52.75
27.70
^{13}C NMR ($CDCl_3$, 151 MHz)
f1 (ppm)

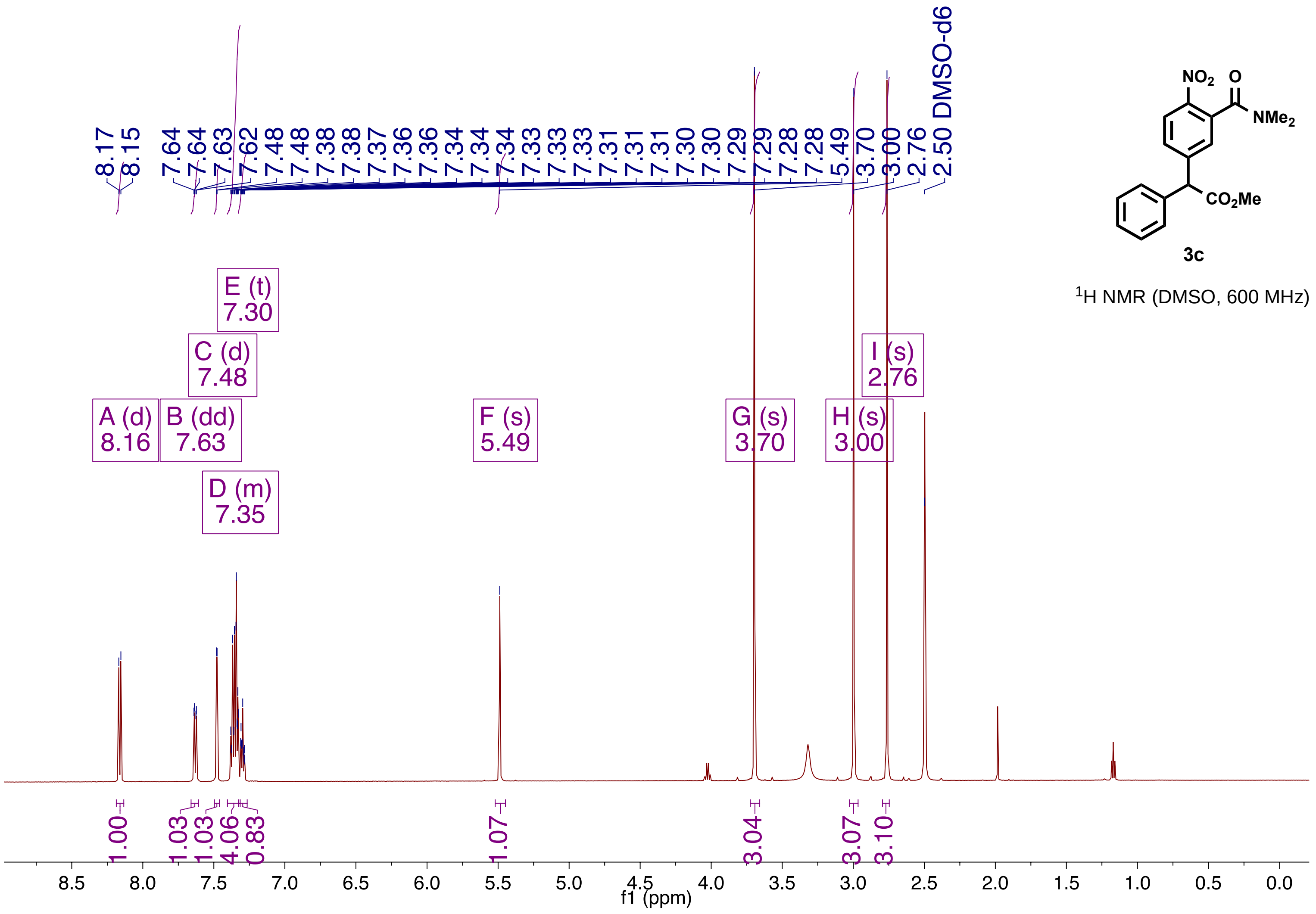

8.17
8.15
7.64
7.64
7.63
7.62
7.48
7.48
7.38
7.38
7.37
7.36
7.36
7.34
7.34
7.34
7.33
7.33
7.33
7.31
7.31
7.31
7.30
7.30
7.29
7.29
7.28
7.28
5.49
3.70
3.00
2.76
2.50 DMSO-d6
E (t) 7.30
C (d) 7.48
A (d) 8.16
B (dd) 7.63
D (m) 7.35
F (s) 5.49
G (s) 3.70
H (s) 3.00
I (s) 2.76
1.00
1.03
1.03
4.06
0.83
1.07
3.04
3.07
3.10
f1 (ppm)
NO2
O
NMe2
CO2Me
3c
1H NMR (DMSO, 600 MHz)

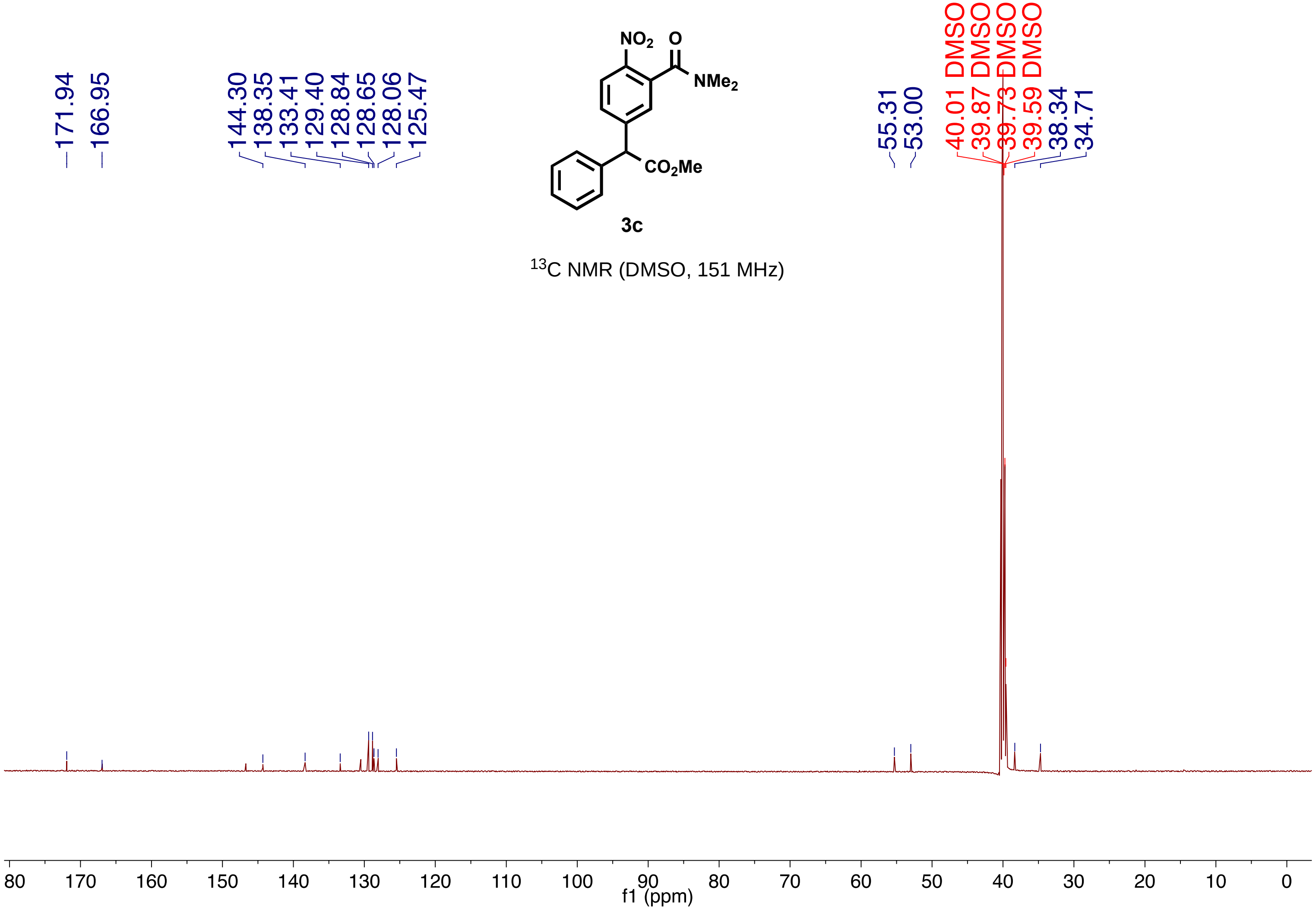
171.94
166.95
144.30
138.35
133.41
129.40
128.84
128.65
128.06
125.47
55.31
53.00
40.01 DMSO
39.87 DMSO
39.73 DMSO
39.59 DMSO
38.34
34.71
NO2
O
NMe2
CO2Me
3c
13C NMR (DMSO, 151 MHz)
180 170 160 150 140 130 120 110 100 90 80 70 60 50 40 30 20 10 0
f1 (ppm)

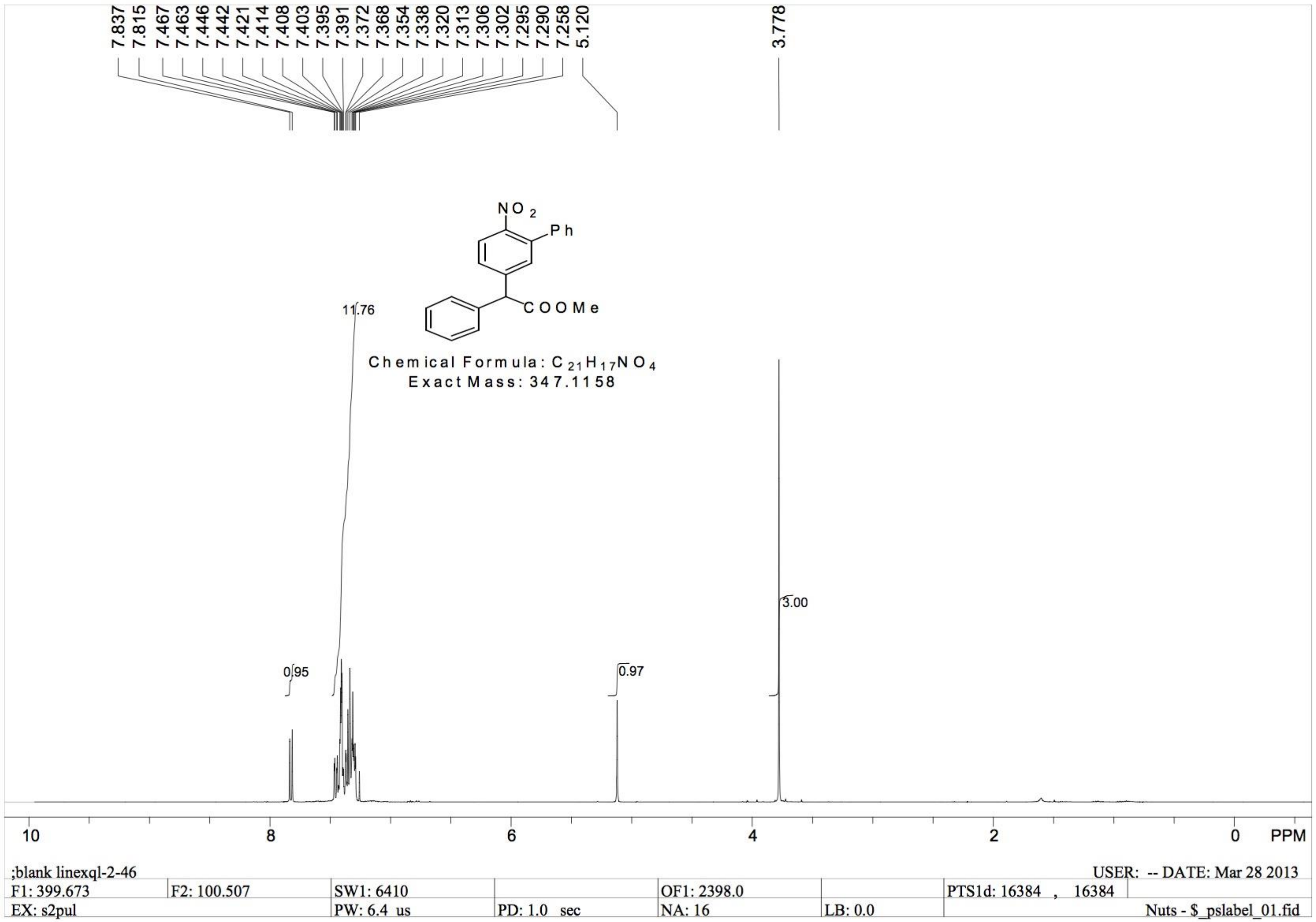
7.837 7.815 7.467 7.463 7.446 7.442 7.421 7.414 7.408 7.403 7.395 7.391 7.372 7.368 7.354 7.338 7.320 7.313 7.306 7.302 7.295 7.290 7.258 5.120
3.778
NO2
Ph
COOMe
Chemical Formula: C21H17NO4
Exact Mass: 347.1158
11.76
0.95
0.97
3.00
10
8
6
4
2
0
PPM
;blank linexql-2-46
USER: -- DATE: Mar 28 2013
F1: 399.673
F2: 100.507
SW1: 6410
OF1: 2398.0
PTS1d: 16384 , 16384
EX: s2pul
PW: 6.4 us
PD: 1.0 sec
NA: 16
LB: 0.0
Nuts - $_pslabel_01.fid

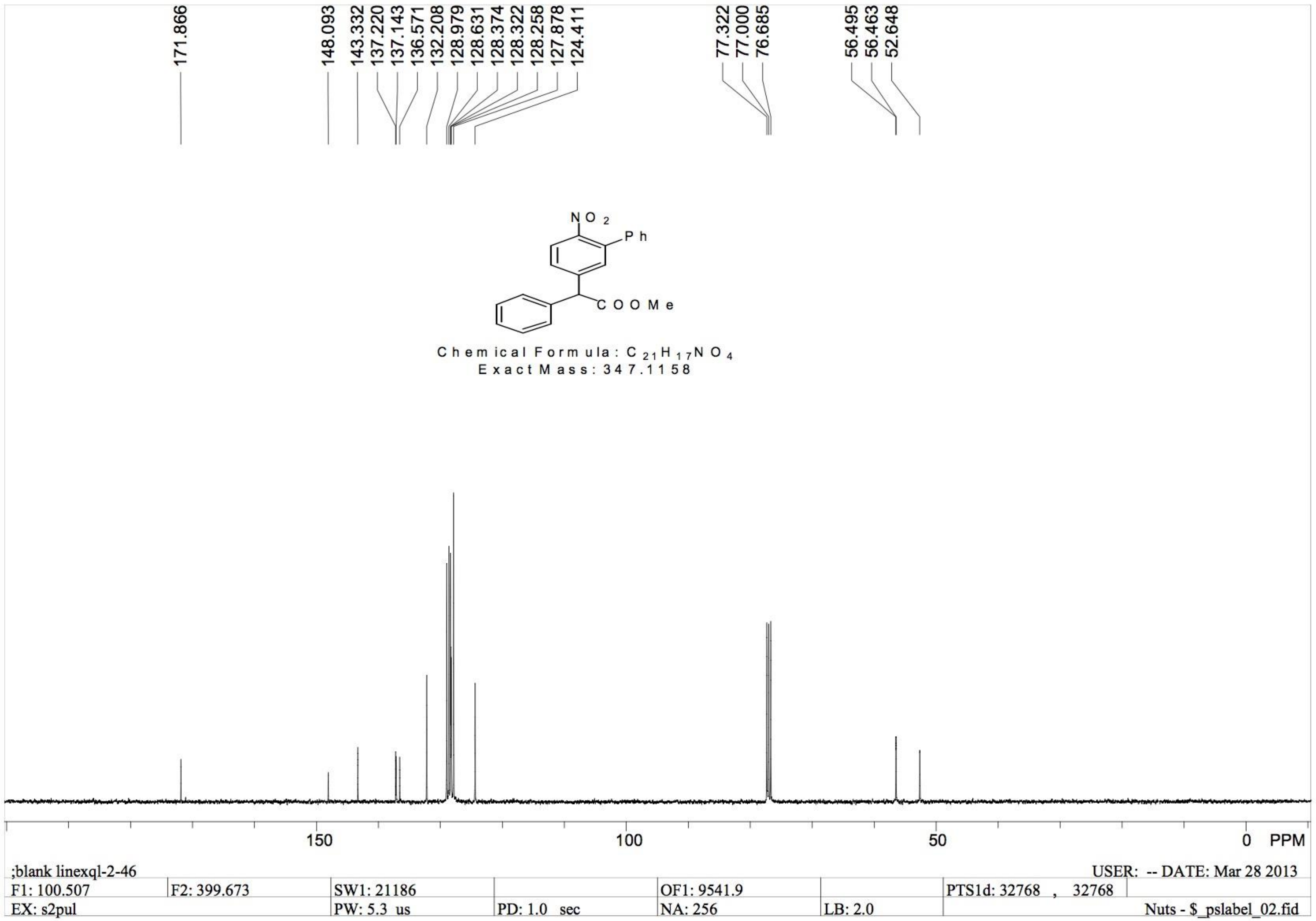
171.866
148.093
143.332
137.220
137.143
136.571
132.208
128.979
128.631
128.374
128.322
128.258
127.878
124.411
77.322
77.000
76.685
56.495
56.463
52.648
NO2
Ph
COOMe
Chemical Formula: C21H17NO4
Exact Mass: 347.1158
150
100
50
0 PPM
;blank linexql-2-46
USER: -- DATE: Mar 28 2013
F1: 100.507
F2: 399.673
SW1: 21186
OF1: 9541.9
PTS1d: 32768 , 32768
EX: s2pul
PW: 5.3 us
PD: 1.0 sec
NA: 256
LB: 2.0
Nuts - $_pslabel_02.fid

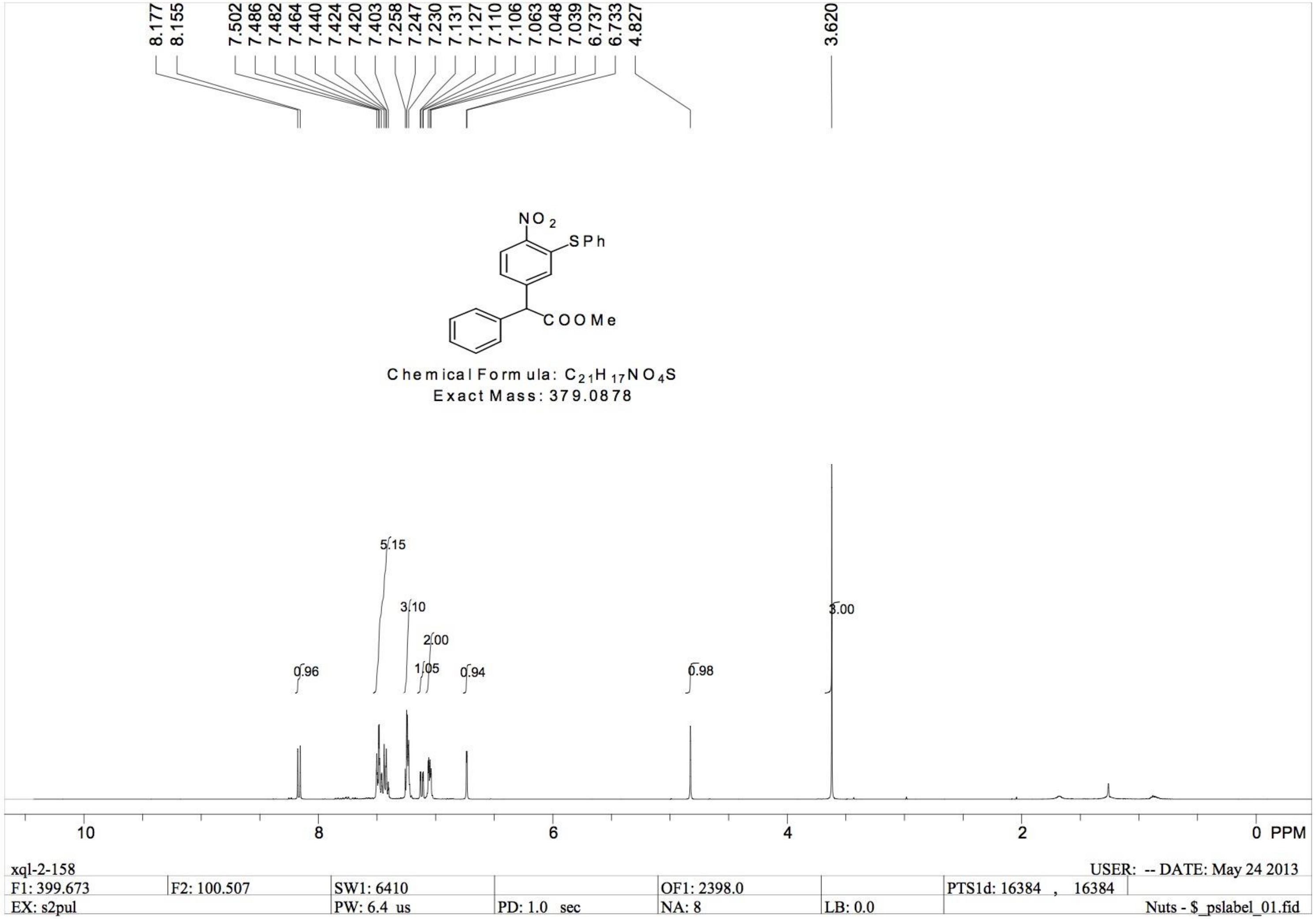
8.177
8.155
7.502
7.486
7.482
7.464
7.440
7.424
7.420
7.403
7.258
7.247
7.230
7.131
7.127
7.110
7.106
7.063
7.048
7.039
6.737
6.733
4.827
3.620
NO 2
SPh
COOMe
Chemical Formula: C21H17NO4S
Exact Mass: 379.0878
5.15
3.10
2.00
1.05
0.96
0.94
0.98
3.00
10
8
6
4
2
0 PPM
xql-2-158
USER: -- DATE: May 24 2013
F1: 399.673
F2: 100.507
SW1: 6410
OF1: 2398.0
PTS1d: 16384 , 16384
EX: s2pul
PW: 6.4 us
PD: 1.0 sec
NA: 8
LB: 0.0
Nuts - $_pslabel_01.fid

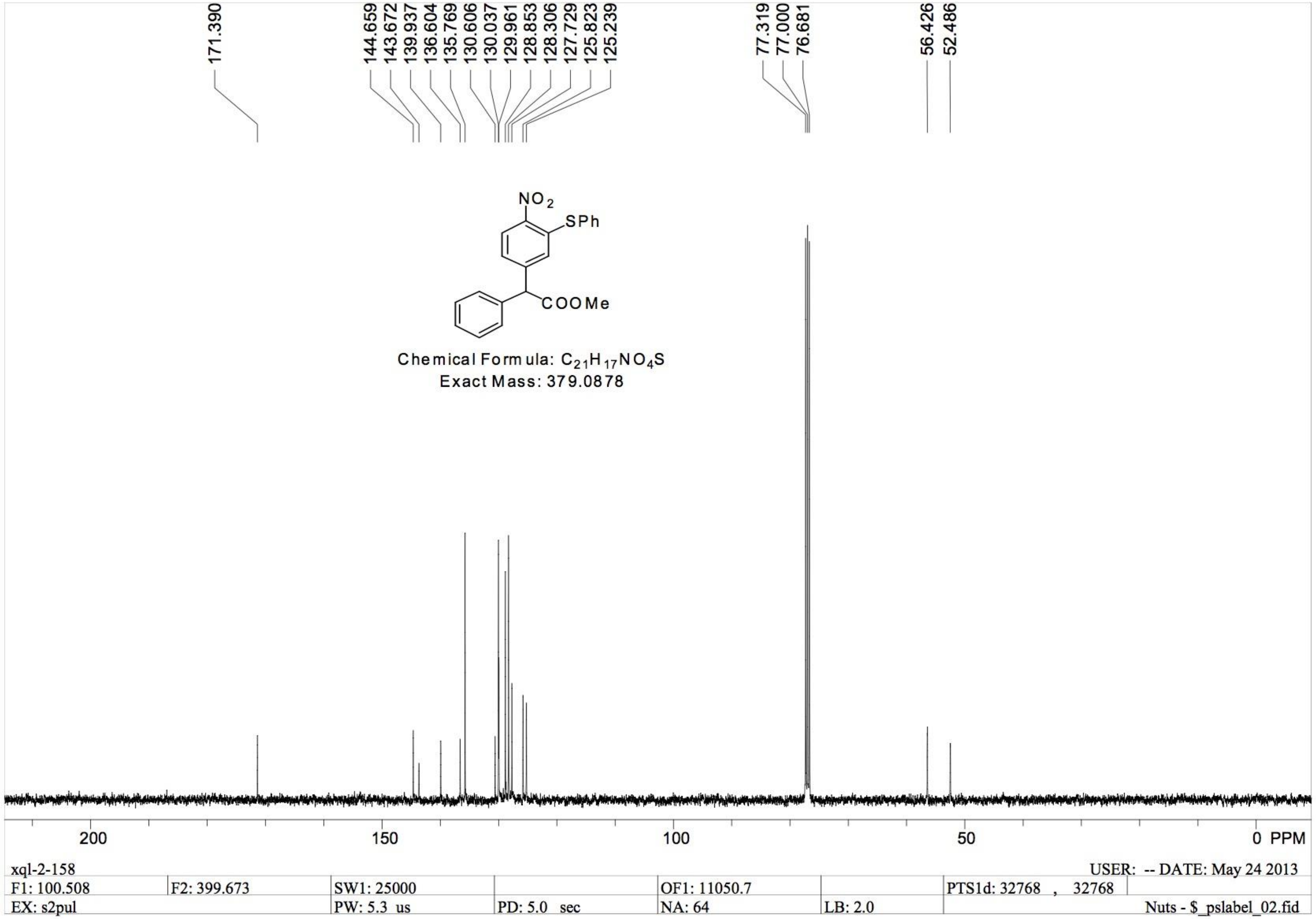
171.390
144.659
143.672
139.937
136.604
135.769
130.606
130.037
129.961
128.853
128.306
127.729
125.823
125.239
77.319
77.000
76.681
56.426
52.486
NO2
SPh
COOMe
Chemical Formula: C21H17NO4S
Exact Mass: 379.0878
200
150
100
50
0 PPM
xql-2-158
USER: -- DATE: May 24 2013
F1: 100.508
F2: 399.673
SW1: 25000
OF1: 11050.7
PTS1d: 32768 , 32768
EX: s2pul
PW: 5.3 us
PD: 5.0 sec
NA: 64
LB: 2.0
Nuts - $_pslabel_02.fid

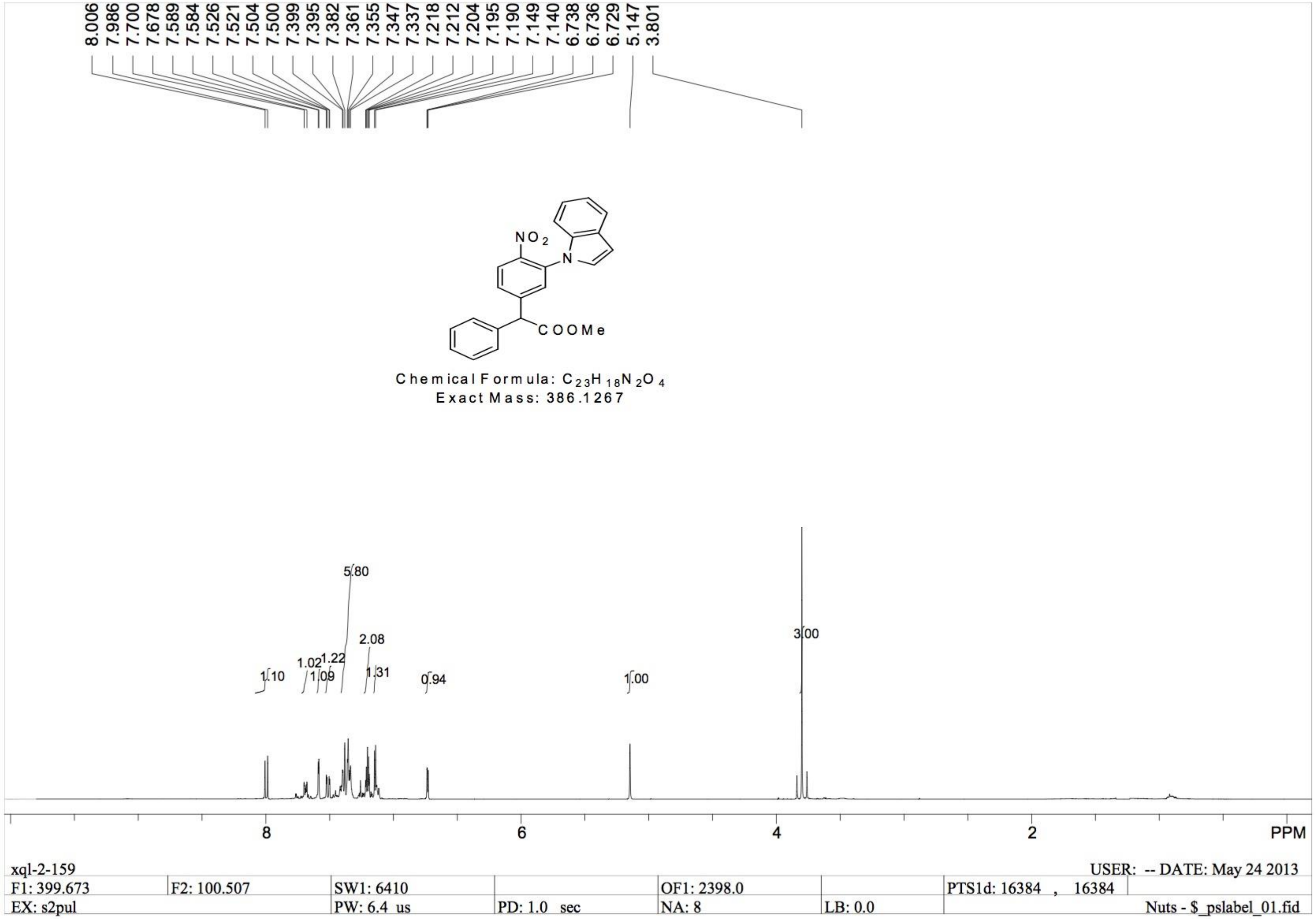
8.006 7.986 7.700 7.678 7.589 7.584 7.526 7.521 7.504 7.500 7.399 7.395 7.382 7.361 7.355 7.347 7.337 7.218 7.212 7.204 7.195 7.190 7.149 7.140 6.738 6.736 6.729 5.147 3.801
NO2
N
COOMe
Chemical Formula: C23H18N2O4
Exact Mass: 386.1267
5.80
2.08
1.02 1.22
1.10 1.09 1.31 0.94 1.00
3.00
8 6 4 2 PPM
xql-2-159
USER: -- DATE: May 24 2013
F1: 399.673 F2: 100.507 SW1: 6410 OF1: 2398.0 PTS1d: 16384 , 16384
EX: s2pul PW: 6.4 us PD: 1.0 sec NA: 8 LB: 0.0 Nuts - $_pslabel_01.fid

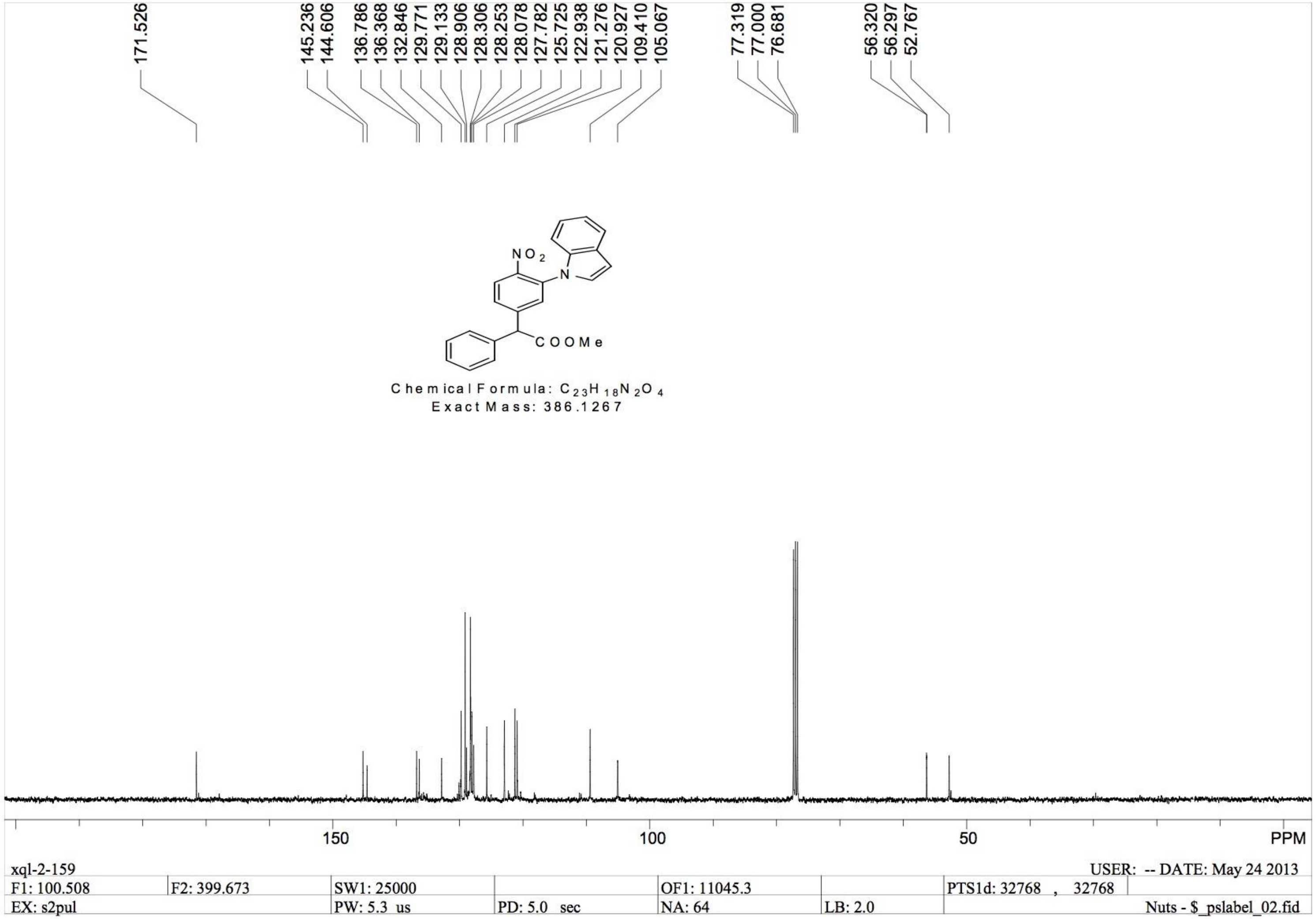
171.526
145.236
144.606
136.786
136.368
132.846
129.771
129.133
128.906
128.306
128.253
128.078
127.782
125.725
122.938
121.276
120.927
109.410
105.067
77.319
77.000
76.681
56.320
56.297
52.767
NO2
N
COOMe
Chemical Formula: C23H18N2O4
Exact Mass: 386.1267
150
100
50
PPM
xql-2-159
USER: -- DATE: May 24 2013
F1: 100.508
F2: 399.673
SW1: 25000
OF1: 11045.3
PTS1d: 32768 , 32768
EX: s2pul
PW: 5.3 us
PD: 5.0 sec
NA: 64
LB: 2.0
Nuts - $_pslabel_02.fid

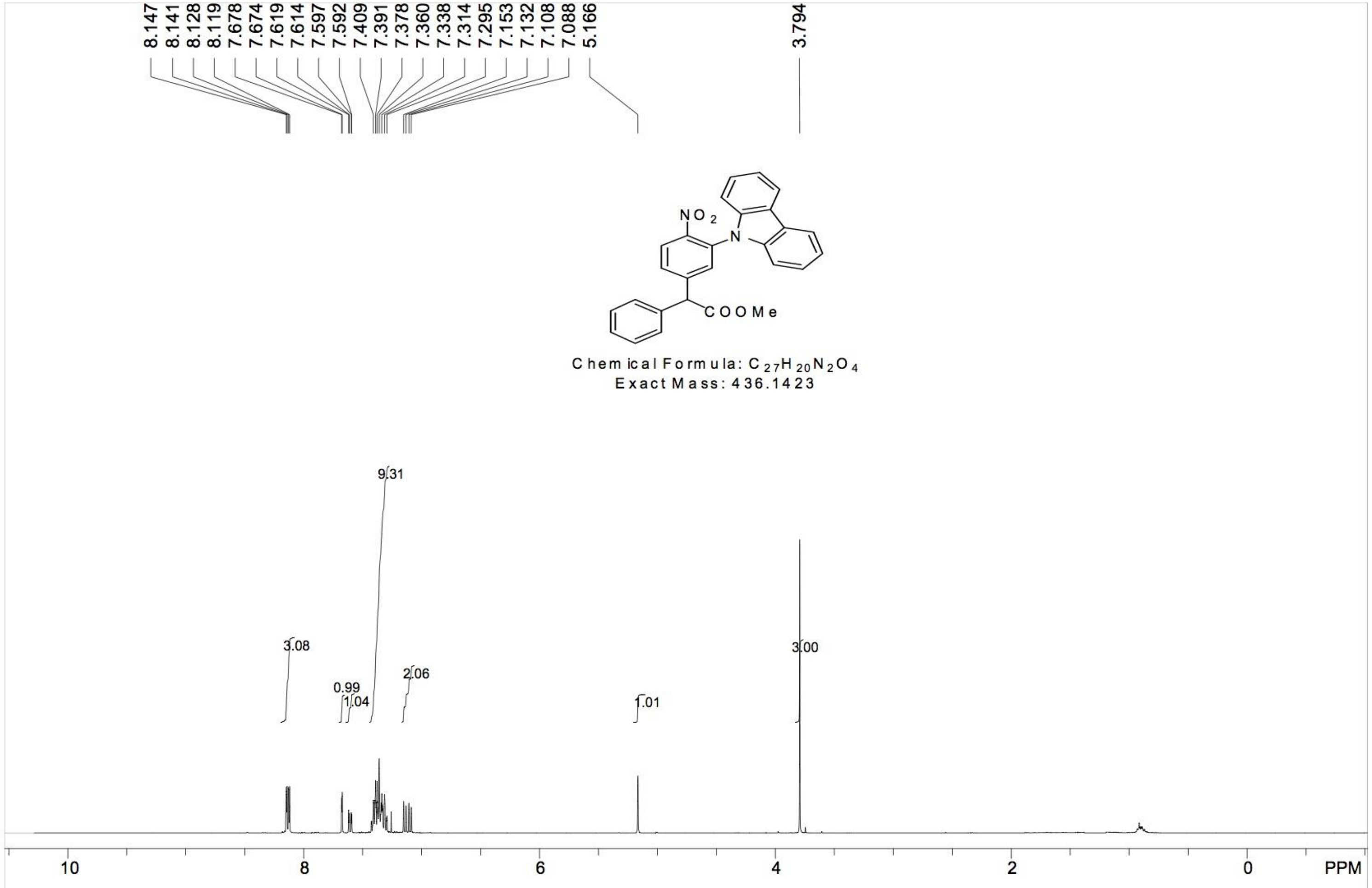

xql-2-160							USER: -- DATE: May 24 2013
F1: 399.673	F2: 100.507	SW1: 6410		OF1: 2398.0		PTS1d: 16384 , 16384	
EX: s2pul		PW: 6.4 us	PD: 1.0 sec	NA: 8	LB: 0.0		Nuts - $_pslabel_01.fid

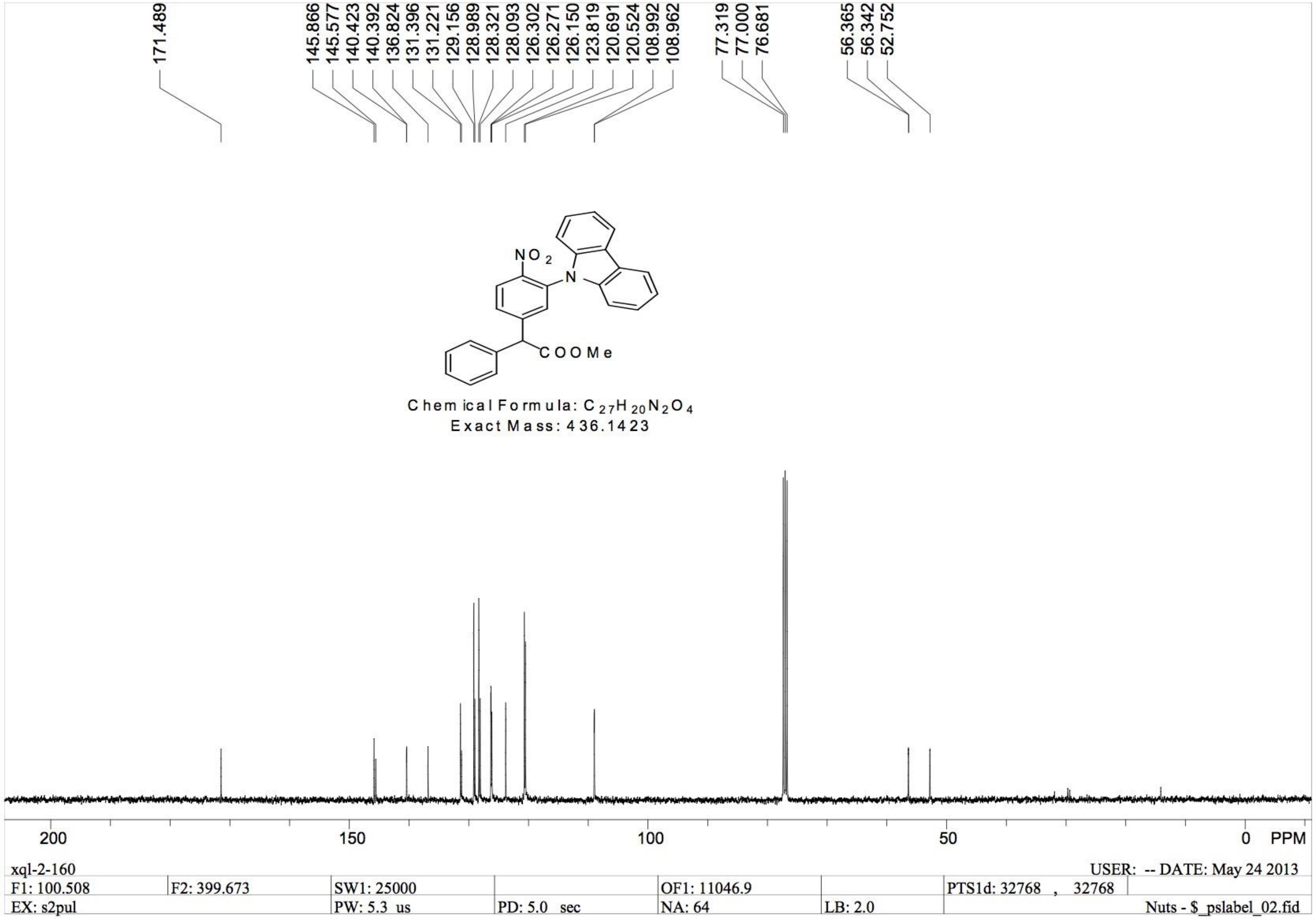

171.489
145.866
145.577
140.423
140.392
136.824
131.396
131.221
129.156
128.989
128.321
128.093
126.302
126.271
126.150
123.819
120.691
120.524
108.992
108.962
77.319
77.000
76.681
56.365
56.342
52.752
NO2
N
COOMe
Chemical Formula: C27H20N2O4
Exact Mass: 436.1423
200
150
100
50
0 PPM
xql-2-160
USER: -- DATE: May 24 2013
F1: 100.508
F2: 399.673
SW1: 25000
OF1: 11046.9
PTS1d: 32768 , 32768
EX: s2pul
PW: 5.3 us
PD: 5.0 sec
NA: 64
LB: 2.0
Nuts - $_pslabel_02.fid

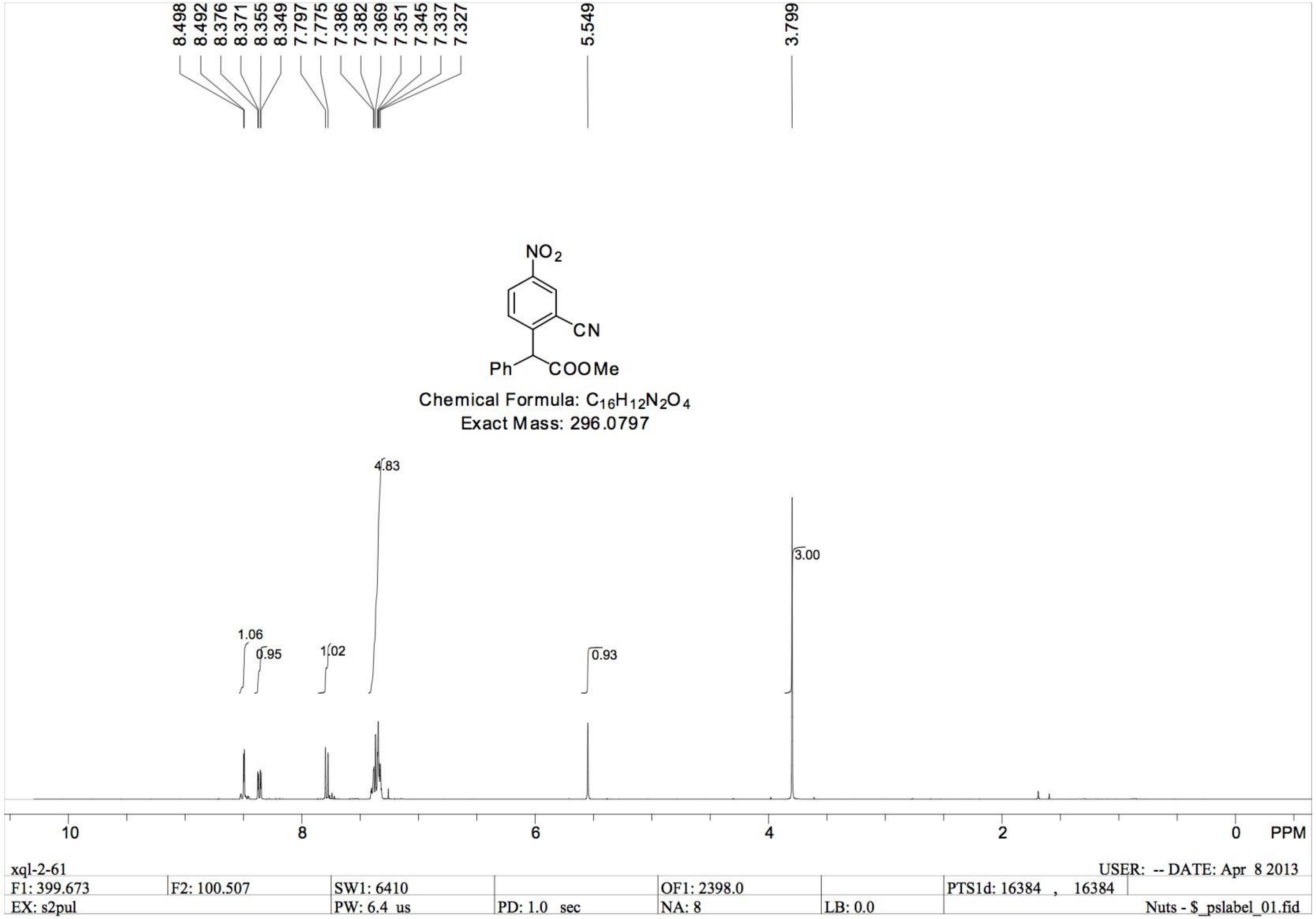
8.498
8.492
8.376
8.371
8.355
8.349
7.797
7.775
7.386
7.382
7.369
7.351
7.345
7.337
7.327
5.549
3.799
NO2
CN
Ph
COOMe
Chemical Formula: C16H12N2O4
Exact Mass: 296.0797
4.83
3.00
1.06
0.95
1.02
0.93
10
8
6
4
2
0
PPM
xql-2-61
USER: -- DATE: Apr 8 2013
F1: 399.673
F2: 100.507
SW1: 6410
OF1: 2398.0
PTS1d: 16384 , 16384
EX: s2pul
PW: 6.4 us
PD: 1.0 sec
NA: 8
LB: 0.0
Nuts - $_pslabel_01.fid

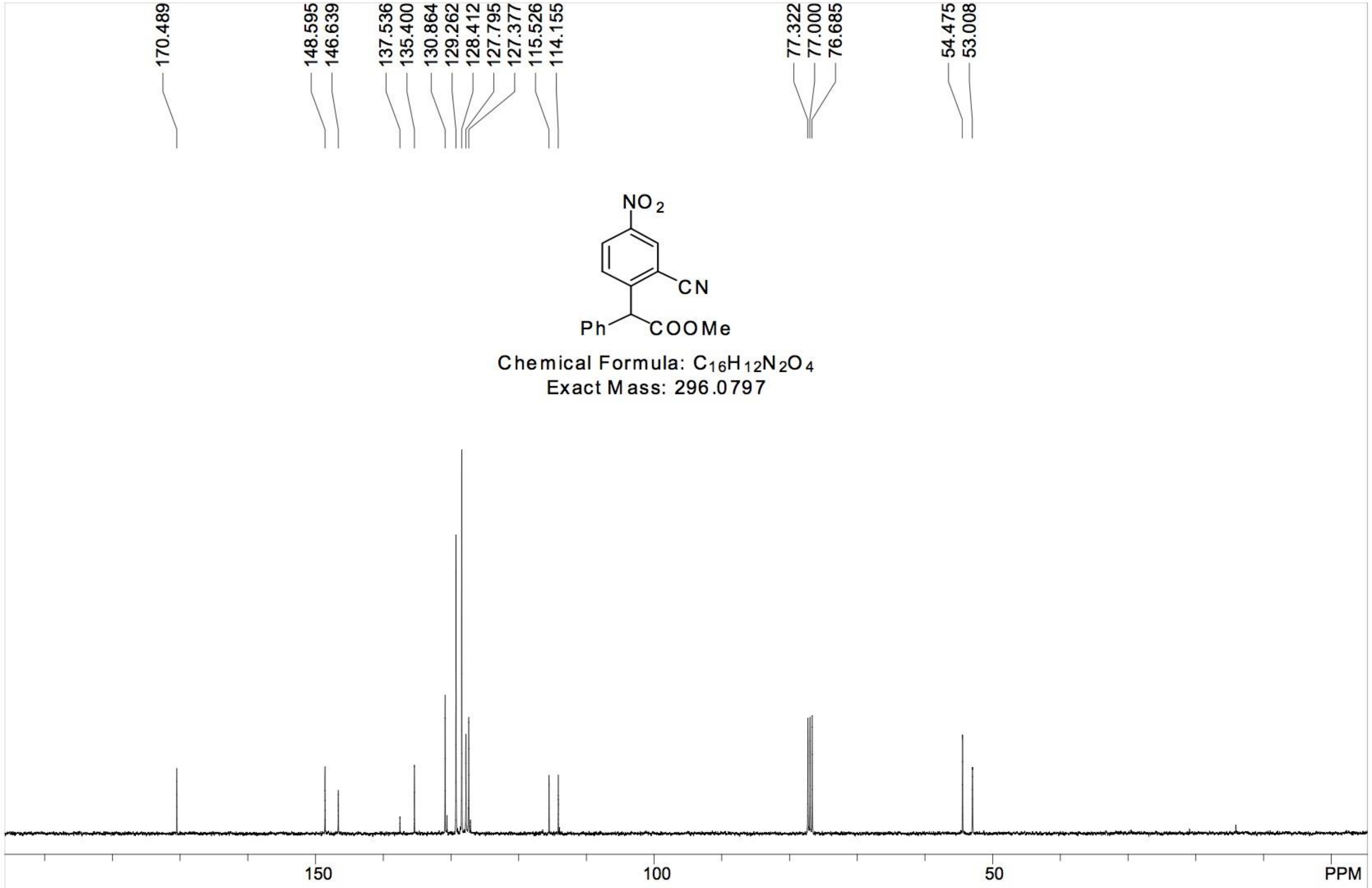

xql-2-61 USER: -- DATE: Apr 8 2013

F1: 100.507	F2: 399.673	SW1: 21186		OF1: 9536.1		PTS1d: 32768 , 32768	
EX: s2pul		PW: 5.3 us	PD: 3.0 sec	NA: 64	LB: 2.0		Nuts - $_pslabel_02.fid

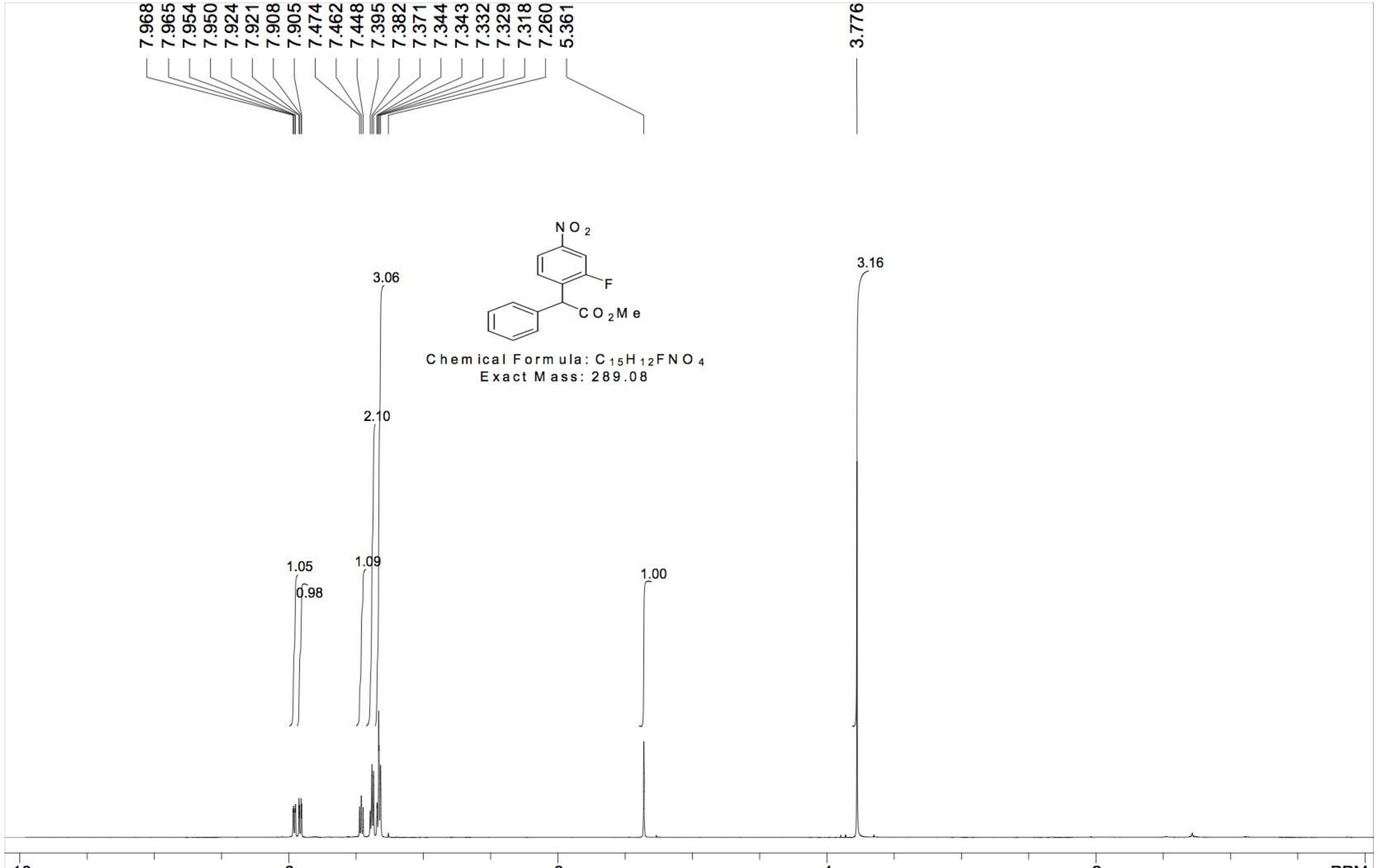

, , PROTON CDCl3 {C:\DATA_Kurti} Gao 22, , USER: nmrsu -- DATE: Wed Sep 28 20:15:44 2016

F1: 600.184	F2: 1.000	SW1: 12019		OF1: 3693.6		PTS1d: 32768 , 32768	
EX: zg30		PW: 10.0 us	PD: 1.0 sec	NA: 8	LB: 0.0		Nuts - $cab-1-8_1

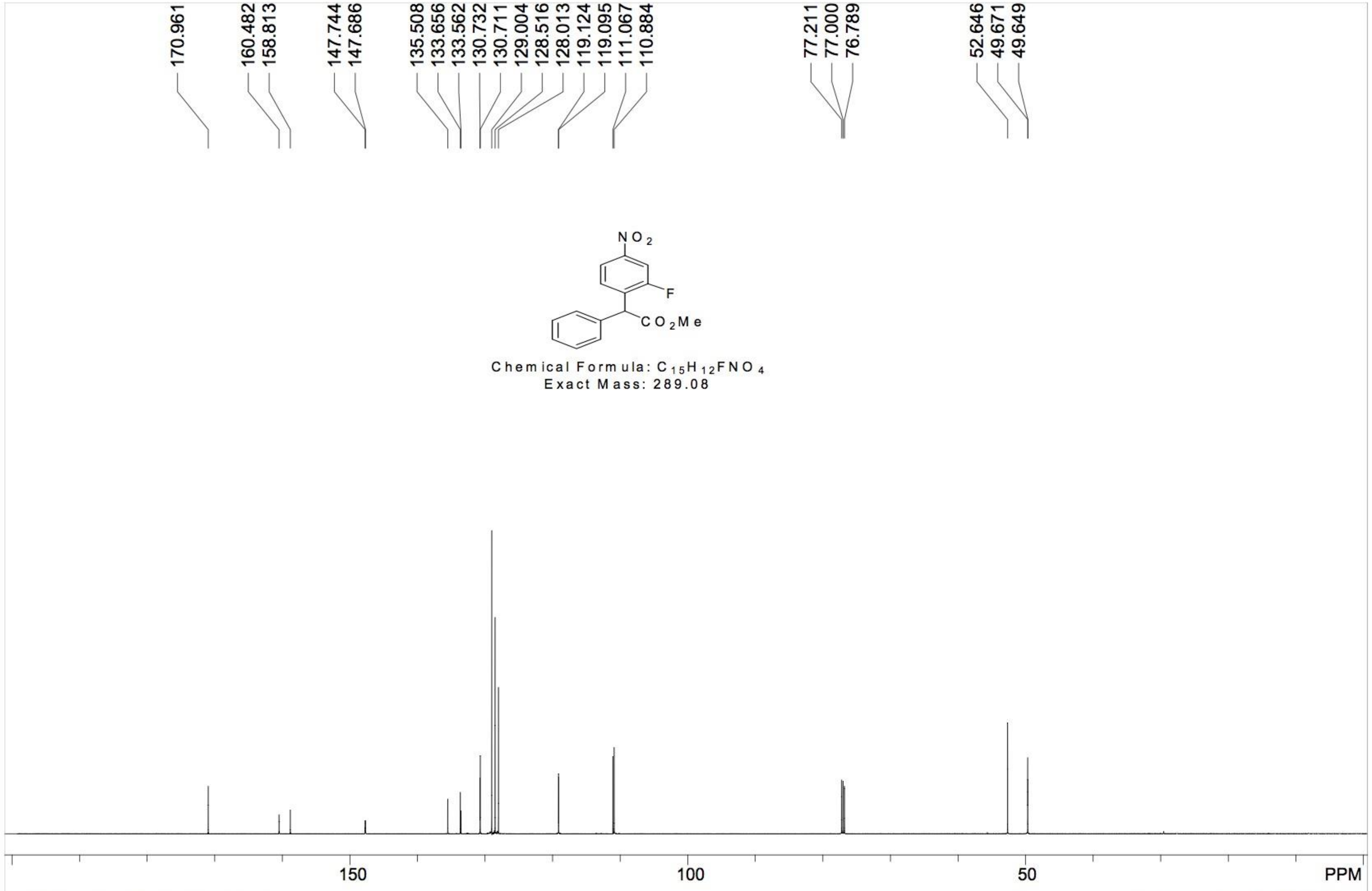

, , C13CPD CDCl3 {C:\DATA_Kurti} Gao 22, ,							USER: nmrsu -- DATE: Wed Sep 28 20:18:56 2016
F1: 150.930	F2: 1.000	SW1: 36058		OF1: 15063.2		PTS1d: 32768 , 32768	
EX: zgpg30		PW: 10.0 us	PD: 2.0 sec	NA: 59	LB: 0.2		Nuts - $cab-1-8_2

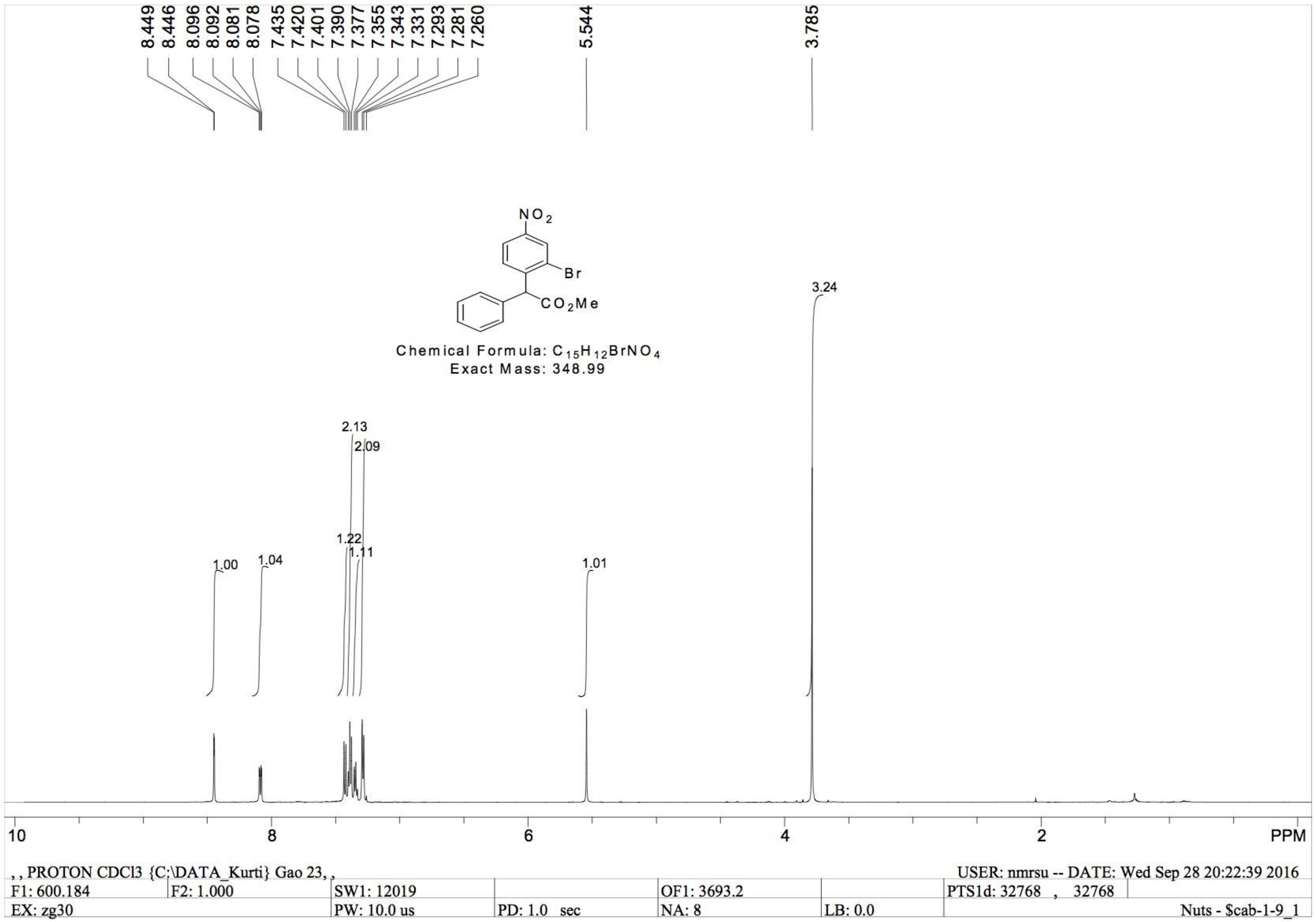
8.449 8.446 8.096 8.092 8.081 8.078 7.435 7.420 7.401 7.390 7.377 7.355 7.343 7.331 7.293 7.281 7.260
5.544
3.785
NO2
Br
CO2Me
Chemical Formula: C15H12BrNO4
Exact Mass: 348.99
3.24
2.13
2.09
1.22
1.11
1.00
1.04
1.01
10
8
6
4
2
PPM
, , PROTON CDCl3 {C:\DATA_Kurti} Gao 23, ,
USER: nmrsu -- DATE: Wed Sep 28 20:22:39 2016
F1: 600.184
F2: 1.000
SW1: 12019
OF1: 3693.2
PTS1d: 32768 , 32768
EX: zg30
PW: 10.0 us
PD: 1.0 sec
NA: 8
LB: 0.0
Nuts - $cab-1-9_1

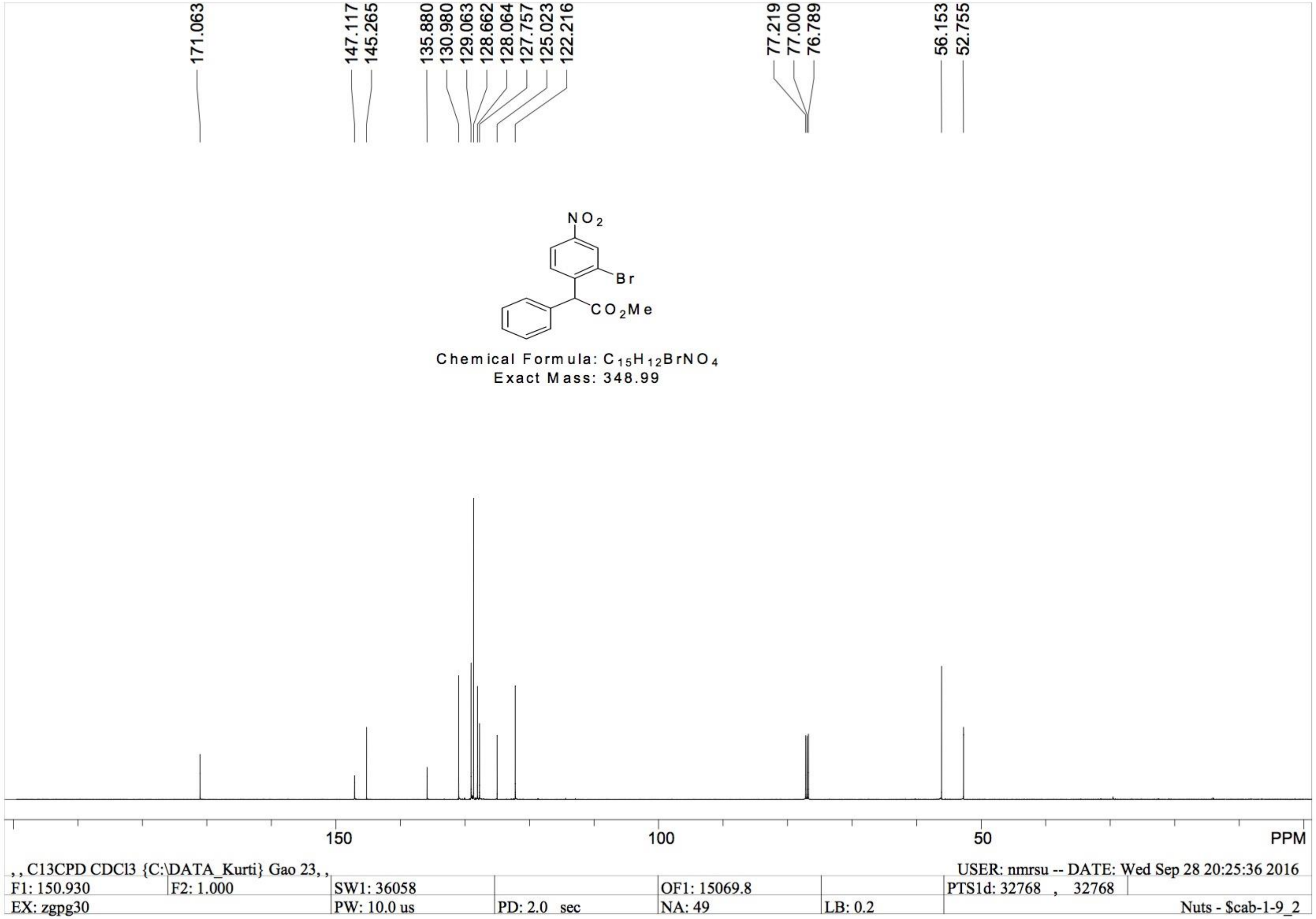
171.063
147.117
145.265
135.880
130.980
129.063
128.662
128.064
127.757
125.023
122.216
77.219
77.000
76.789
56.153
52.755
NO2
Br
CO2Me
Chemical Formula: C15H12BrNO4
Exact Mass: 348.99
150
100
50
PPM
, , C13CPD CDCl3 {C:\DATA_Kurti} Gao 23, ,
USER: nmrsu -- DATE: Wed Sep 28 20:25:36 2016
F1: 150.930
F2: 1.000
SW1: 36058
OF1: 15069.8
PTS1d: 32768 , 32768
EX: zgpg30
PW: 10.0 us
PD: 2.0 sec
NA: 49
LB: 0.2
Nuts - $cab-1-9_2

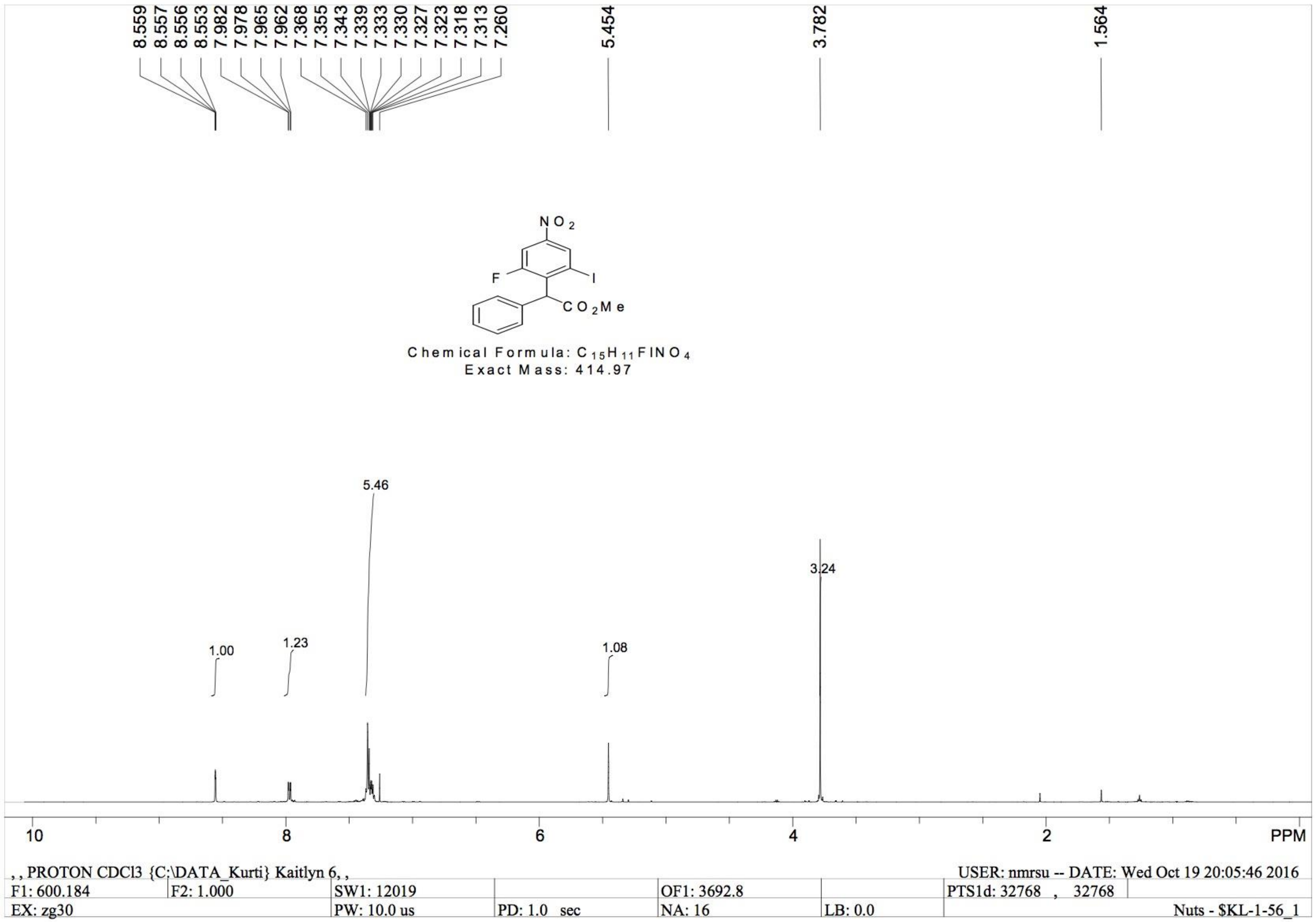
8.559
8.557
8.556
8.553
7.982
7.978
7.965
7.962
7.368
7.355
7.343
7.339
7.333
7.330
7.327
7.323
7.318
7.313
7.260
5.454
3.782
1.564
NO2
F
I
CO2Me
Chemical Formula: C15H11FINO4
Exact Mass: 414.97
5.46
3.24
1.00
1.23
1.08
10
8
6
4
2
PPM
, , PROTON CDCl3 {C:\DATA_Kurti} Kaitlyn 6, ,
USER: nmrsu -- DATE: Wed Oct 19 20:05:46 2016
F1: 600.184
F2: 1.000
SW1: 12019
OF1: 3692.8
PTS1d: 32768 , 32768
EX: zg30
PW: 10.0 us
PD: 1.0 sec
NA: 16
LB: 0.0
Nuts - $KL-1-56_1

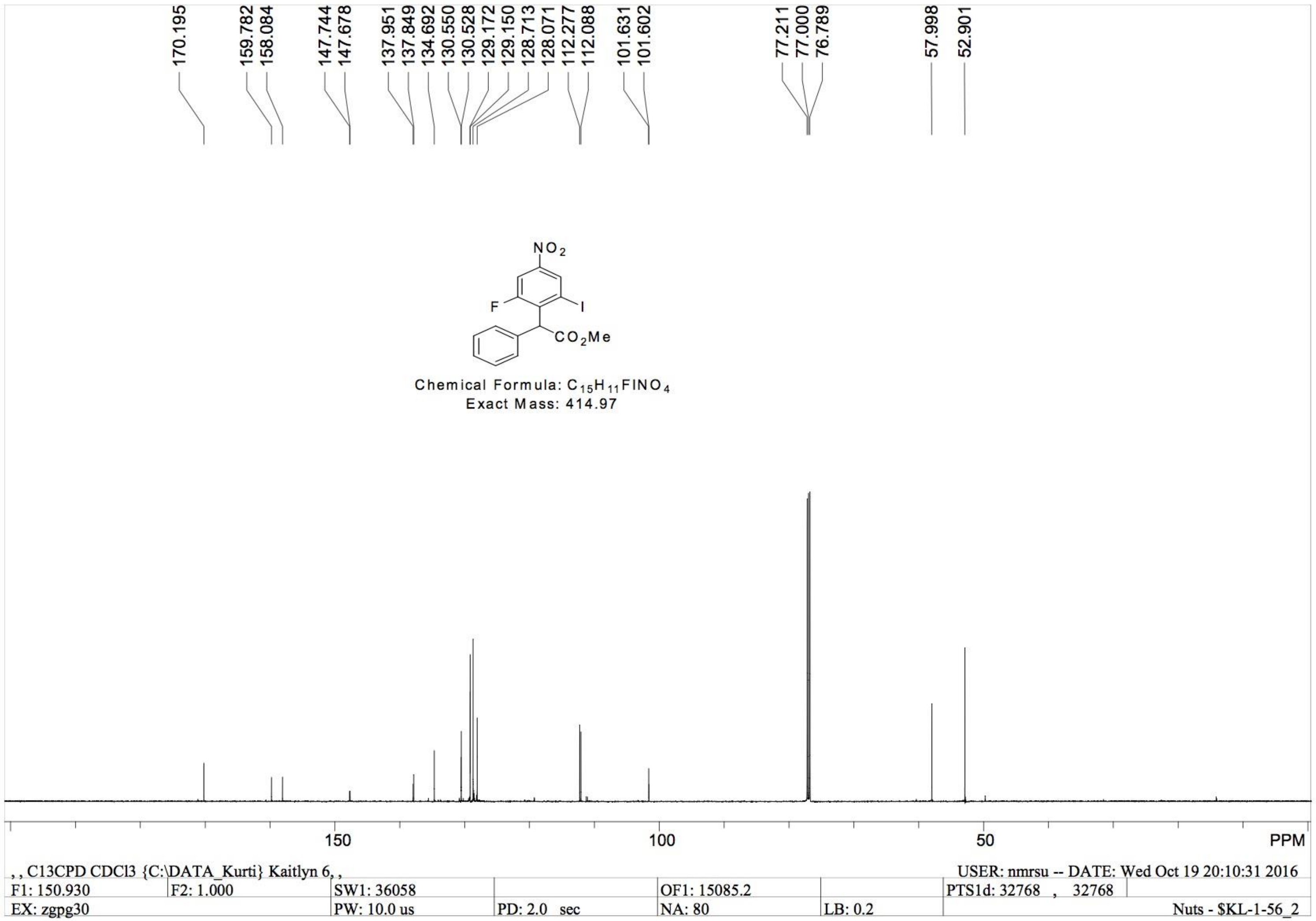
170.195
159.782
158.084
147.744
147.678
137.951
137.849
134.692
130.550
130.528
129.172
129.150
128.713
128.071
112.277
112.088
101.631
101.602
77.211
77.000
76.789
57.998
52.901
NO2
F
I
CO2Me
Chemical Formula: C15H11FINO4
Exact Mass: 414.97
150
100
50
PPM
, , C13CPD CDCl3 {C:\DATA_Kurti} Kaitlyn 6, ,
USER: nmrsu -- DATE: Wed Oct 19 20:10:31 2016
F1: 150.930
F2: 1.000
SW1: 36058
OF1: 15085.2
PTS1d: 32768 , 32768
EX: zgpg30
PW: 10.0 us
PD: 2.0 sec
NA: 80
LB: 0.2
Nuts - $KL-1-56_2

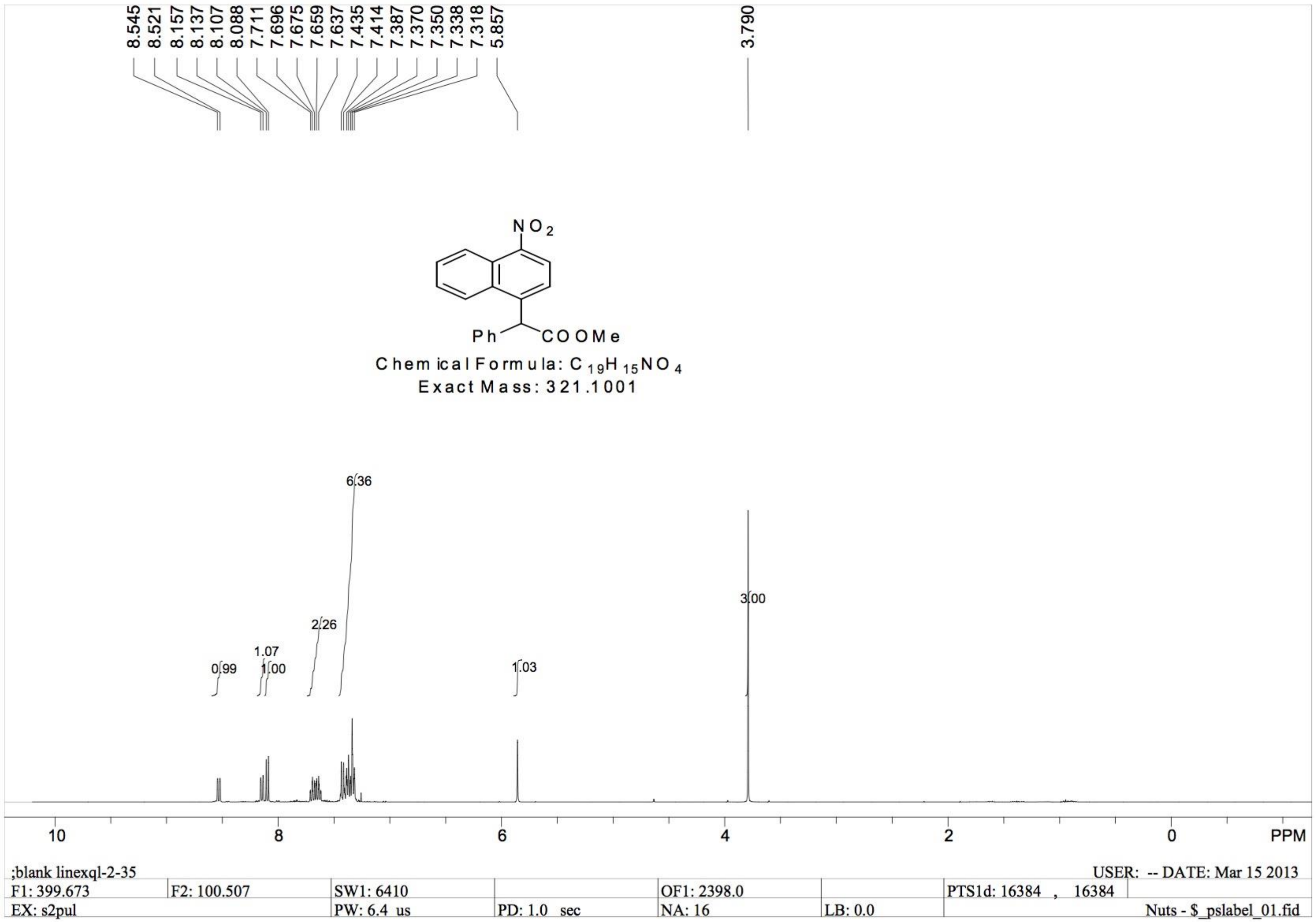
8.545
8.521
8.157
8.137
8.107
8.088
7.711
7.696
7.675
7.659
7.637
7.435
7.414
7.387
7.370
7.350
7.338
7.318
5.857
3.790
NO2
Ph
COOMe
Chemical Formula: C19H15NO4
Exact Mass: 321.1001
6.36
3.00
2.26
1.07
0.99
1.00
1.03
10
8
6
4
2
0
PPM
;blank linexql-2-35
USER: -- DATE: Mar 15 2013
F1: 399.673
F2: 100.507
SW1: 6410
OF1: 2398.0
PTS1d: 16384 , 16384
EX: s2pul
PW: 6.4 us
PD: 1.0 sec
NA: 16
LB: 0.0
Nuts - $_pslabel_01.fid

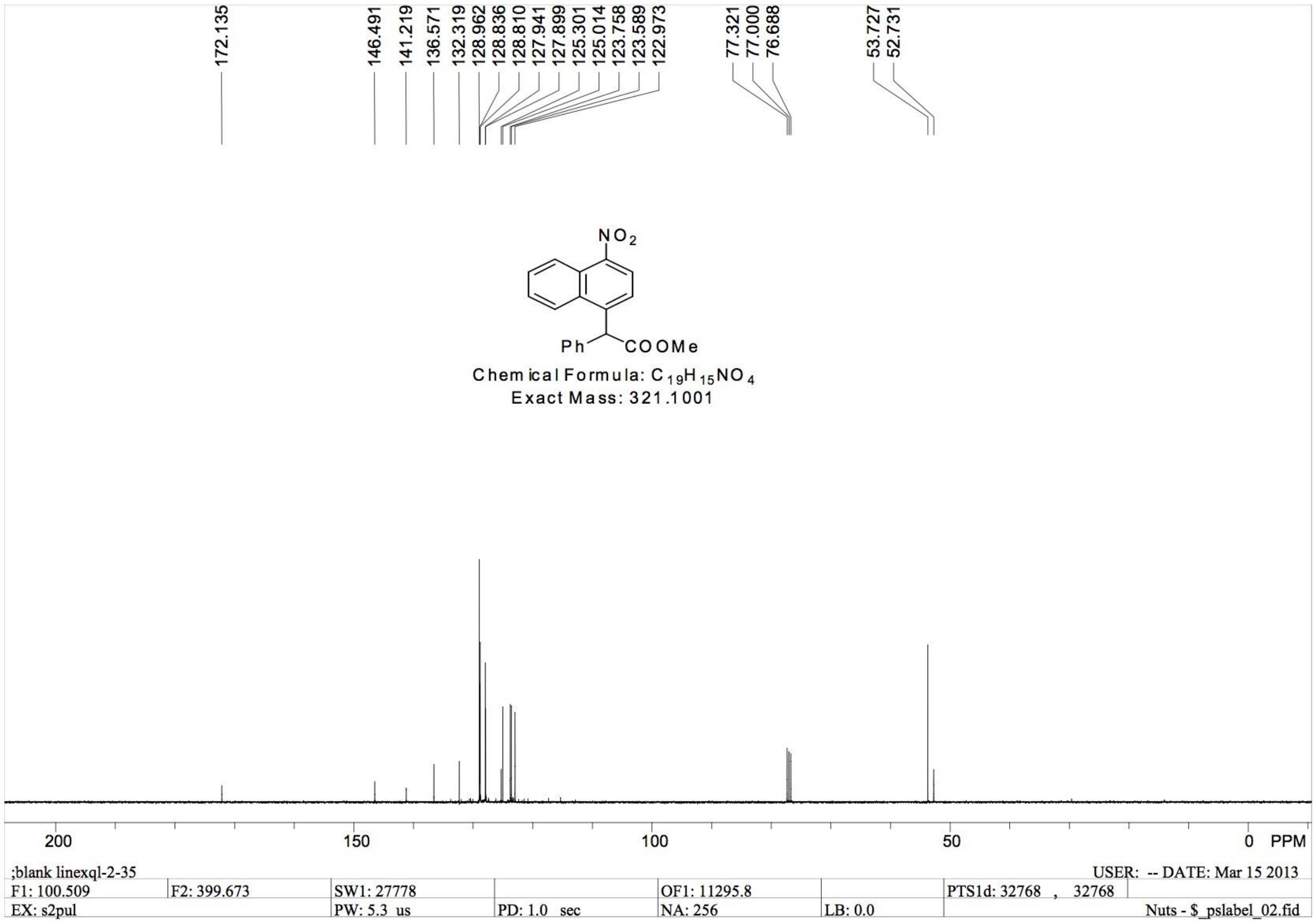

172.135
146.491
141.219
136.571
132.319
128.962
128.836
128.810
127.941
127.899
125.301
125.014
123.758
123.589
122.973
77.321
77.000
76.688
53.727
52.731
NO2
Ph
COOMe
Chemical Formula: C19H15NO4
Exact Mass: 321.1001
200
150
100
50
0 PPM
;blank linexql-2-35
USER: -- DATE: Mar 15 2013
F1: 100.509
F2: 399.673
SW1: 27778
OF1: 11295.8
PTS1d: 32768 , 32768
EX: s2pul
PW: 5.3 us
PD: 1.0 sec
NA: 256
LB: 0.0
Nuts - $_pslabel_02.fid

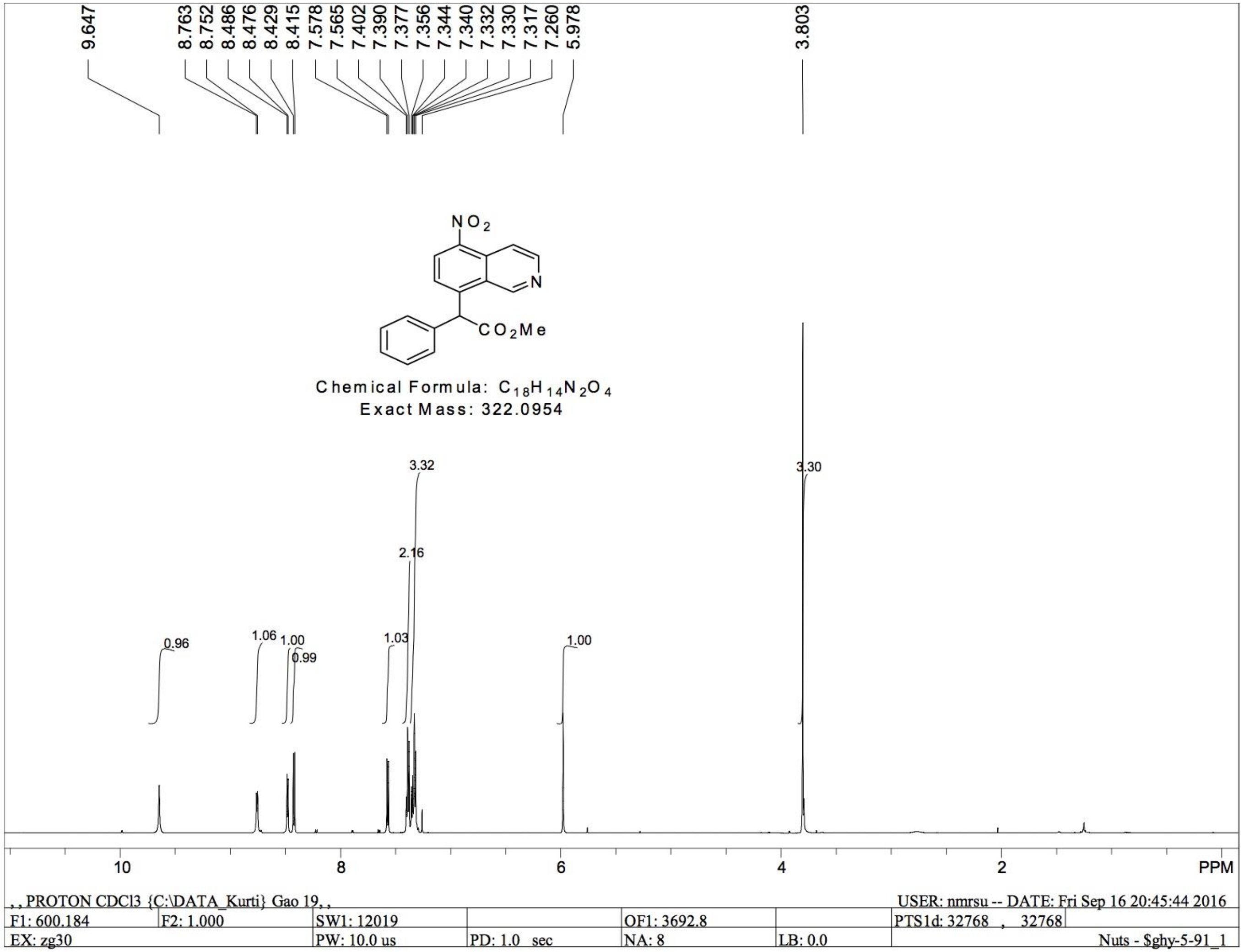
9.647
8.763
8.752
8.486
8.476
8.429
8.415
7.578
7.565
7.402
7.390
7.377
7.356
7.344
7.340
7.332
7.330
7.317
7.260
5.978
3.803
NO2
N
CO2Me
Chemical Formula: C18H14N2O4
Exact Mass: 322.0954
0.96
1.06
1.00
0.99
1.03
2.16
3.32
1.00
3.30
10
8
6
4
2
PPM
, , PROTON CDCl3 {C:\DATA_Kurti} Gao 19, ,
USER: nmrsu -- DATE: Fri Sep 16 20:45:44 2016
F1: 600.184
F2: 1.000
SW1: 12019
OF1: 3692.8
PTS1d: 32768 , 32768
EX: zg30
PW: 10.0 us
PD: 1.0 sec
NA: 8
LB: 0.0
Nuts - $ghy-5-91_1

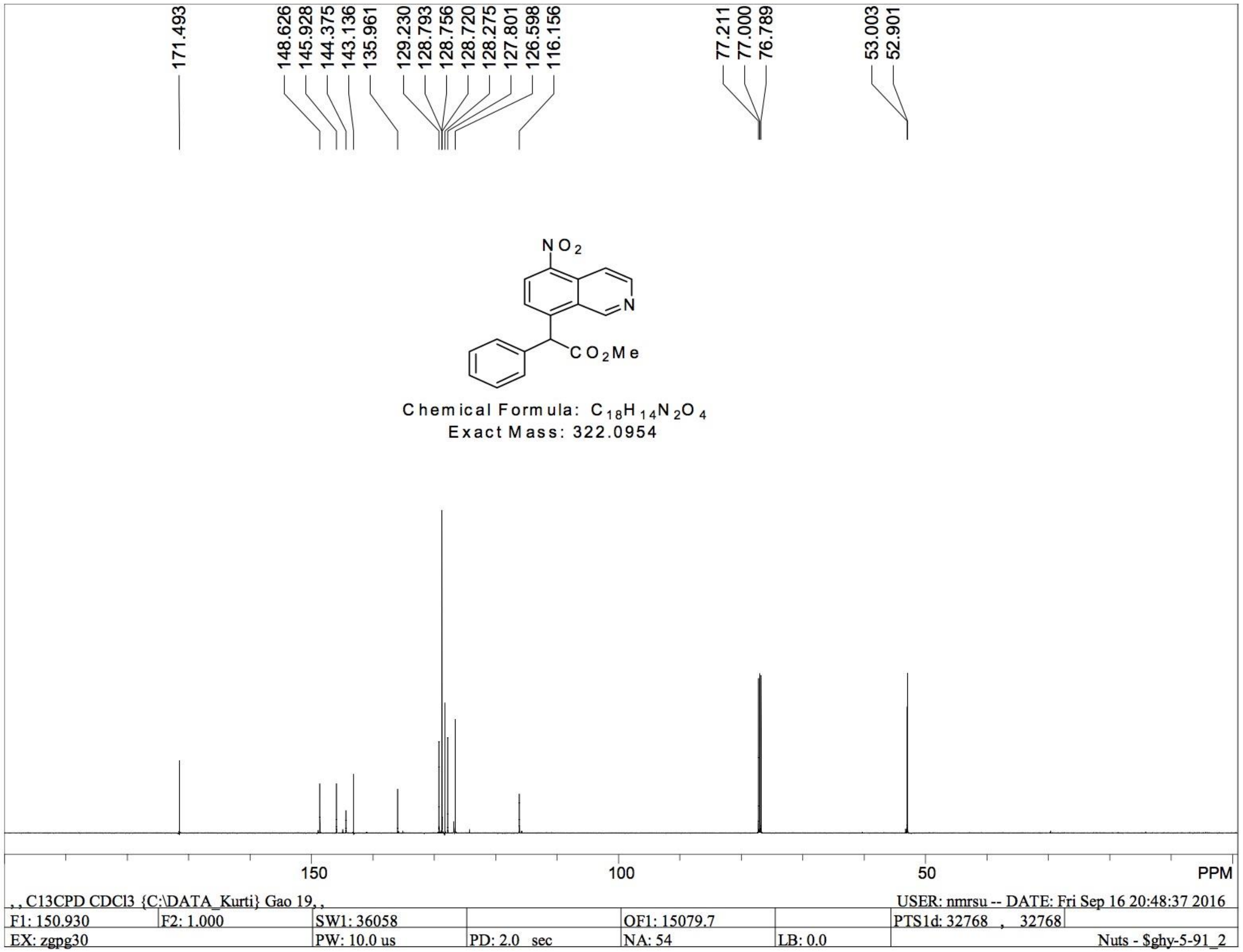
171.493
148.626
145.928
144.375
143.136
135.961
129.230
128.793
128.756
128.720
128.275
127.801
126.598
116.156
77.211
77.000
76.789
53.003
52.901
NO2
N
CO2Me
Chemical Formula: C18H14N2O4
Exact Mass: 322.0954
150
100
50
PPM
, , C13CPD CDCl3 {C:\DATA_Kurti} Gao 19, ,
USER: nmrsu -- DATE: Fri Sep 16 20:48:37 2016
F1: 150.930
F2: 1.000
SW1: 36058
OF1: 15079.7
PTS1d: 32768 , 32768
EX: zgpg30
PW: 10.0 us
PD: 2.0 sec
NA: 54
LB: 0.0
Nuts - $ghy-5-91_2

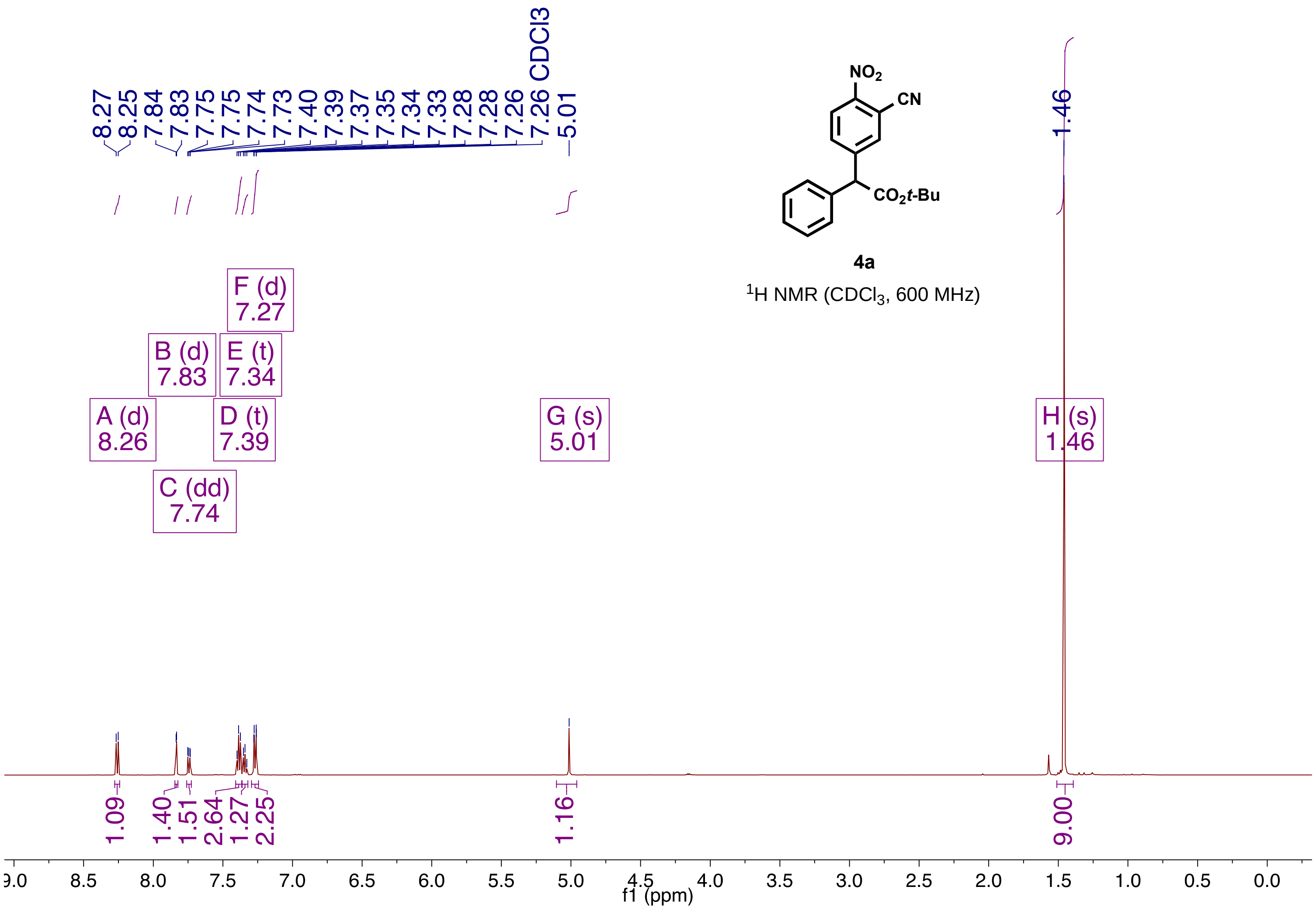

8.27
8.25
7.84
7.83
7.75
7.75
7.74
7.73
7.40
7.39
7.37
7.35
7.34
7.33
7.28
7.28
7.26
7.26 CDCl3
5.01
1.46
NO2
CN
CO2t-Bu
4a
1H NMR (CDCl3, 600 MHz)
F (d) 7.27
B (d) 7.83
E (t) 7.34
A (d) 8.26
D (t) 7.39
C (dd) 7.74
G (s) 5.01
H (s) 1.46
1.09
1.40
1.51
2.64
1.27
2.25
1.16
9.00
9.0
8.5
8.0
7.5
7.0
6.5
6.0
5.5
5.0
4.5
4.0
3.5
3.0
2.5
2.0
1.5
1.0
0.5
0.0
f1 (ppm)

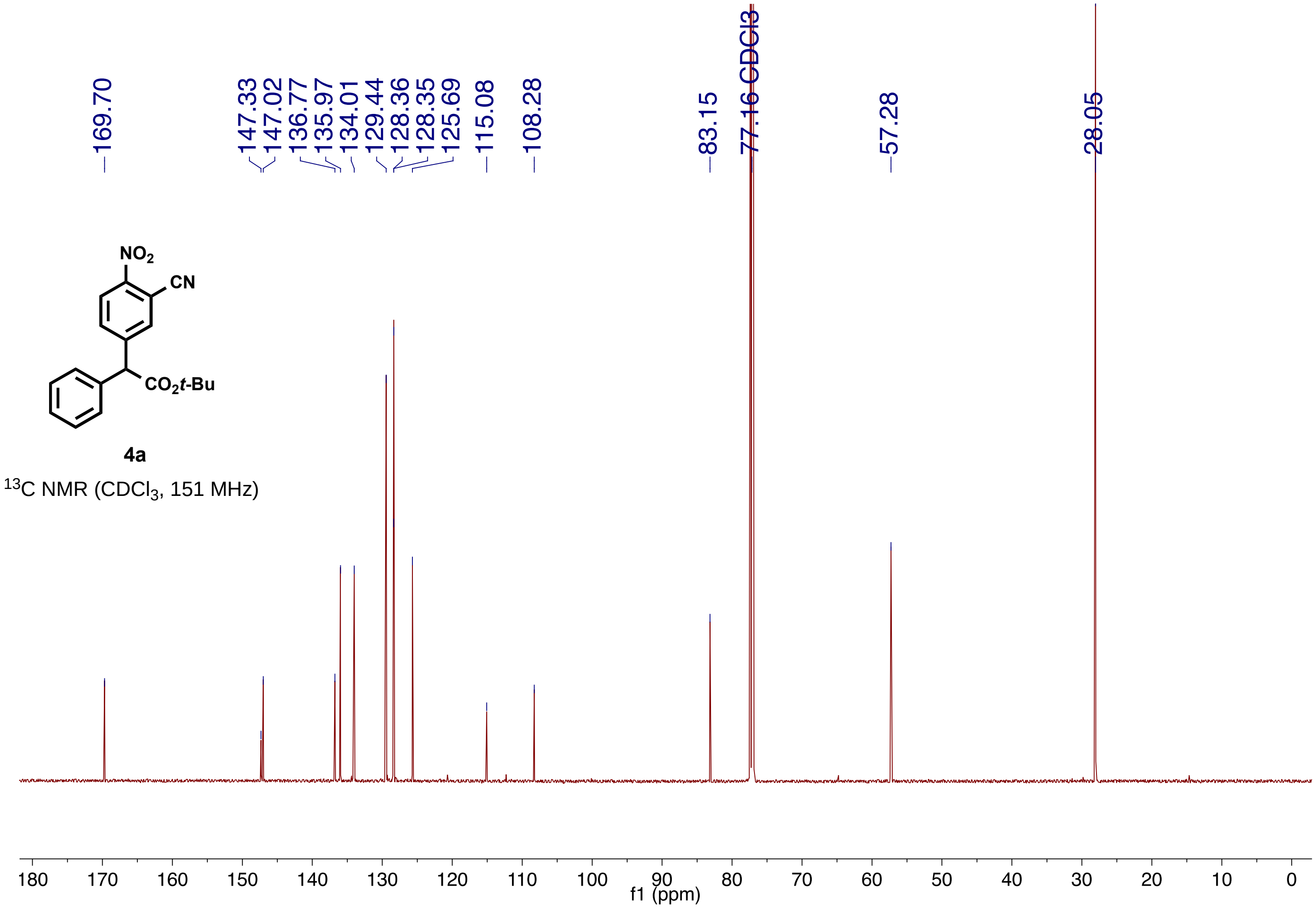

169.70
147.33
147.02
136.77
135.97
134.01
129.44
128.36
128.35
125.69
115.08
108.28
83.15
77.16 CDCl3
57.28
28.05
NO2
CN
CO2t-Bu
4a
13C NMR (CDCl3, 151 MHz)
180
170
160
150
140
130
120
110
100
90
80
70
60
50
40
30
20
10
0
f1 (ppm)

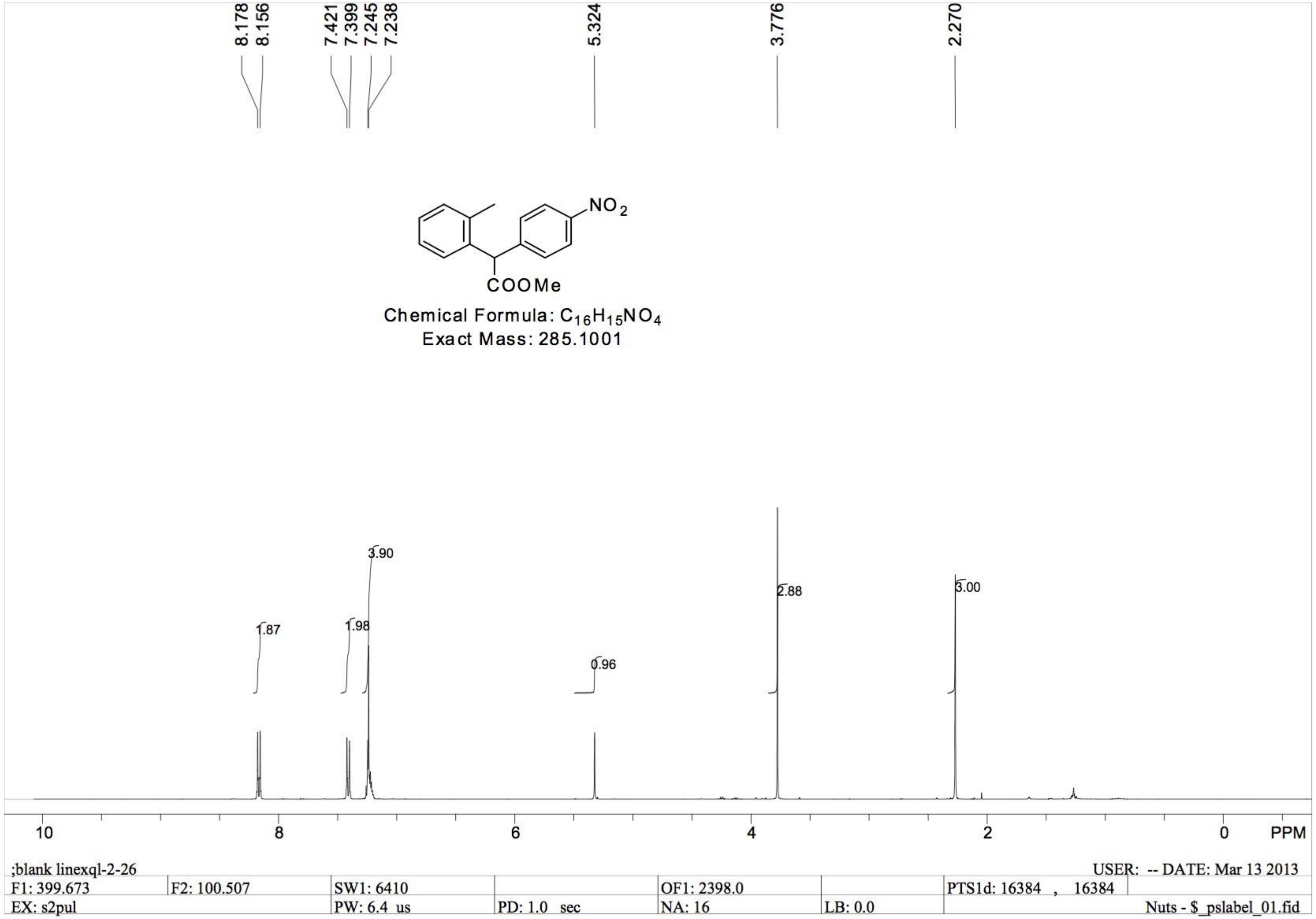
8.178
8.156
7.421
7.399
7.245
7.238
5.324
3.776
2.270
NO2
COOMe
Chemical Formula: C16H15NO4
Exact Mass: 285.1001
1.87
1.98
3.90
0.96
2.88
3.00
10
8
6
4
2
0
PPM
;blank linexql-2-26
USER: -- DATE: Mar 13 2013
F1: 399.673
F2: 100.507
SW1: 6410
OF1: 2398.0
PTS1d: 16384 , 16384
EX: s2pul
PW: 6.4 us
PD: 1.0 sec
NA: 16
LB: 0.0
Nuts - $_pslabel_01.fid

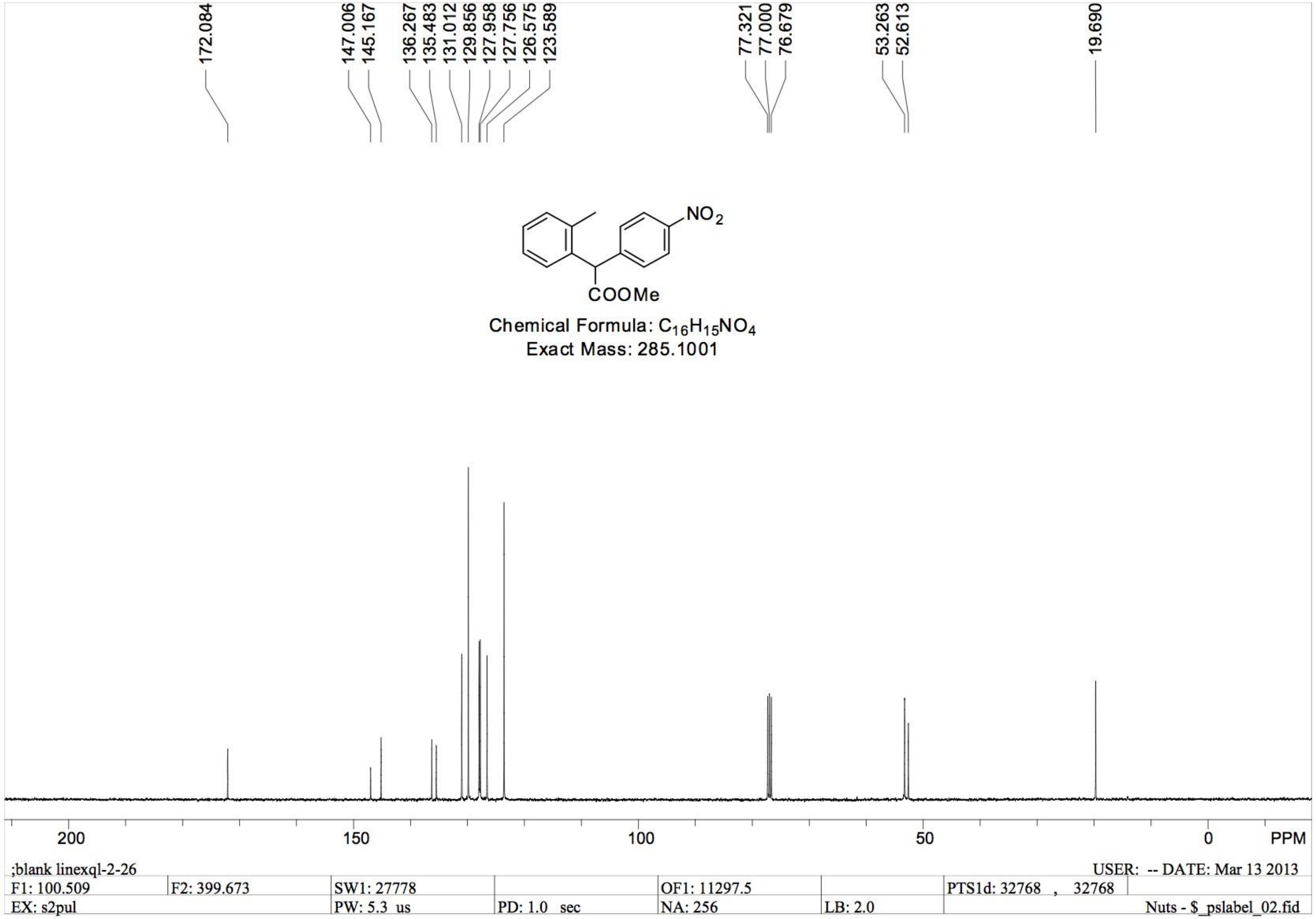
172.084
147.006
145.167
136.267
135.483
131.012
129.856
127.958
127.756
126.575
123.589
77.321
77.000
76.679
53.263
52.613
19.690
NO2
COOMe
Chemical Formula: C16H15NO4
Exact Mass: 285.1001
200
150
100
50
0
PPM
;blank linexql-2-26
USER: -- DATE: Mar 13 2013
F1: 100.509
F2: 399.673
SW1: 27778
OF1: 11297.5
PTS1d: 32768 , 32768
EX: s2pul
PW: 5.3 us
PD: 1.0 sec
NA: 256
LB: 2.0
Nuts - $_pslabel_02.fid

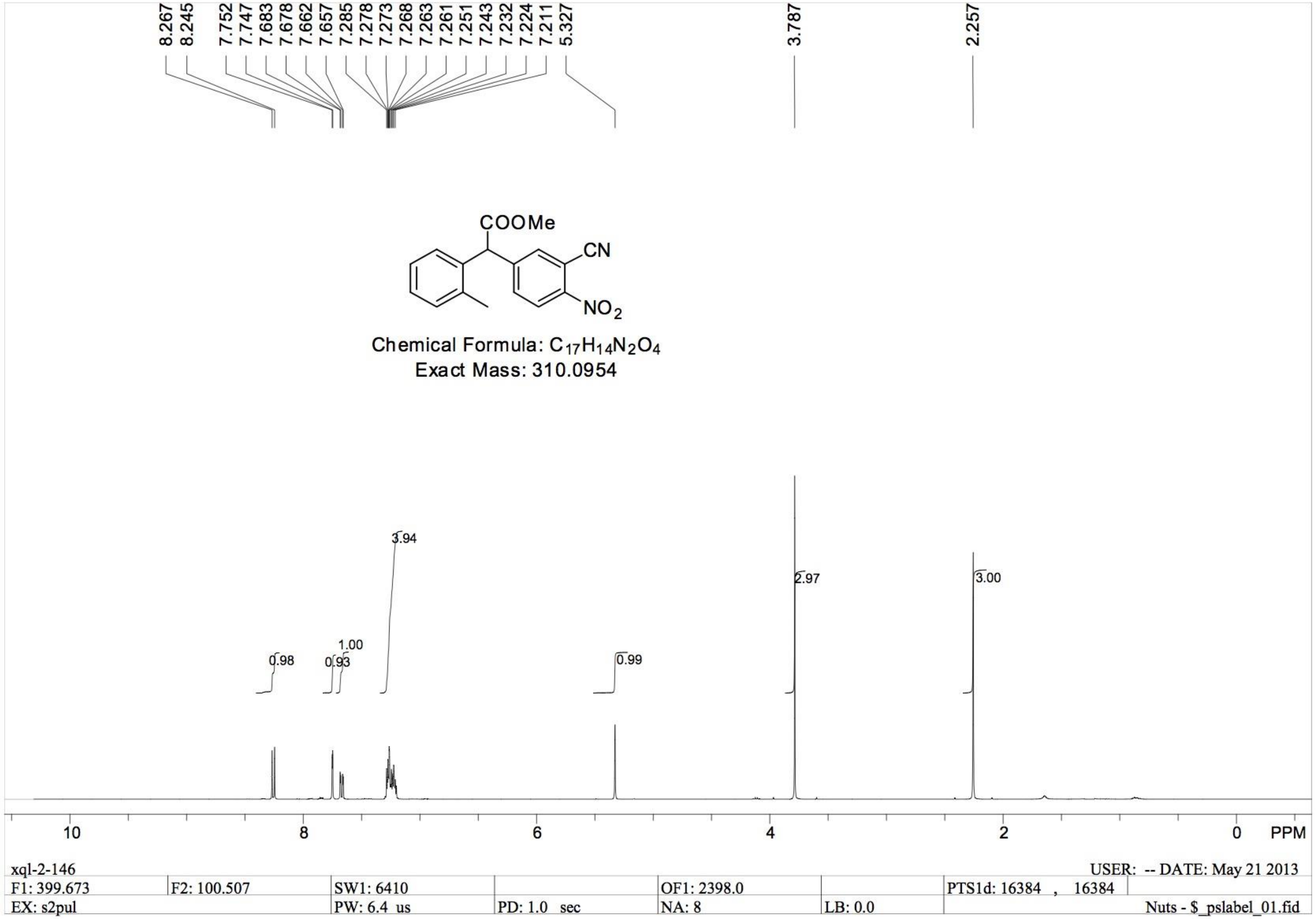
8.267
8.245
7.752
7.747
7.683
7.678
7.662
7.657
7.285
7.278
7.273
7.268
7.263
7.261
7.251
7.243
7.232
7.224
7.211
5.327
3.787
2.257
COOMe
CN
NO2
Chemical Formula: C17H14N2O4
Exact Mass: 310.0954
3.94
2.97
3.00
1.00
0.98
0.93
0.99
10
8
6
4
2
0
PPM
xql-2-146
USER: -- DATE: May 21 2013
F1: 399.673
F2: 100.507
SW1: 6410
OF1: 2398.0
PTS1d: 16384 , 16384
EX: s2pul
PW: 6.4 us
PD: 1.0 sec
NA: 8
LB: 0.0
Nuts - $_pslabel_01.fid

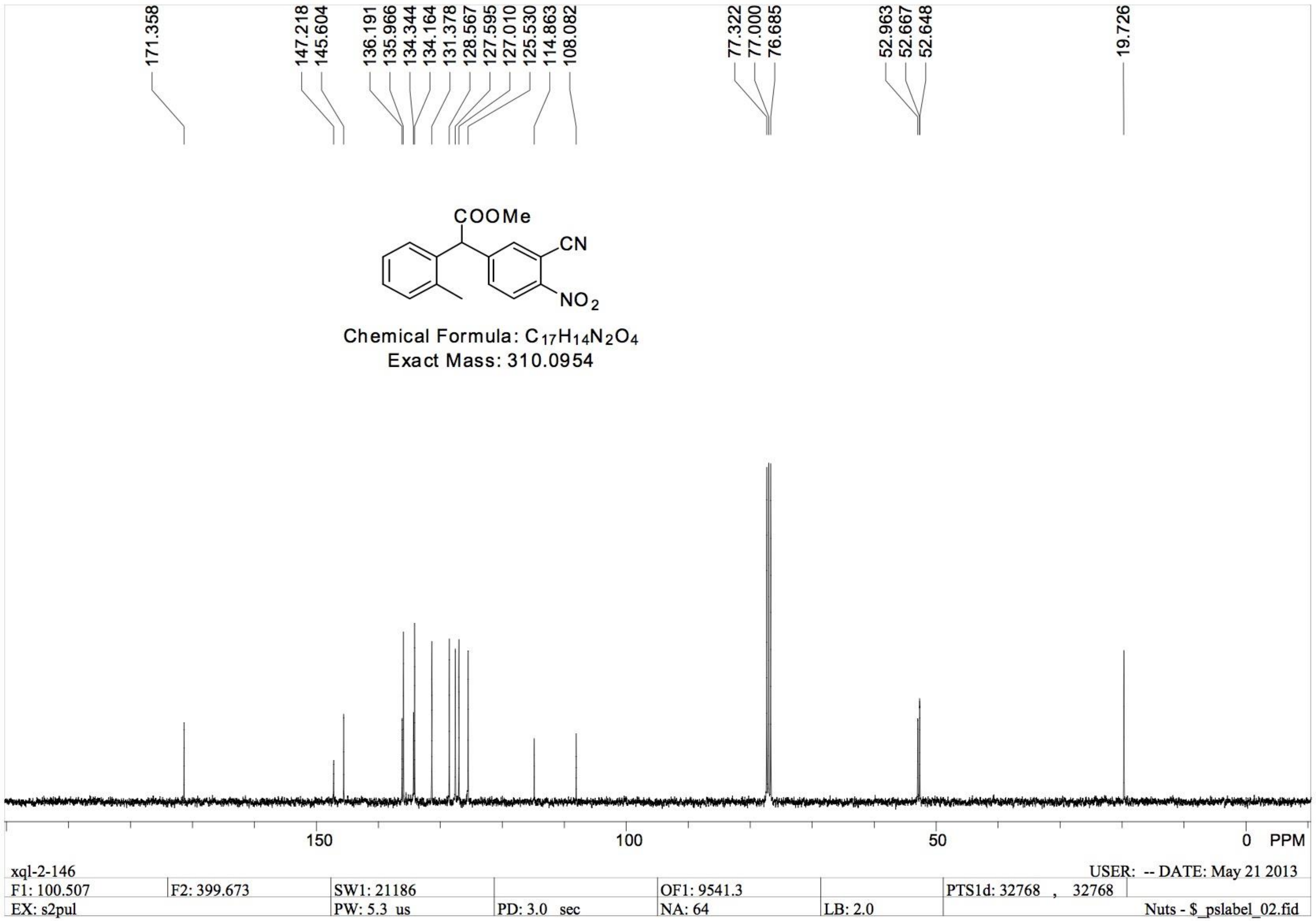
171.358
147.218
145.604
136.191
135.966
134.344
134.164
131.378
128.567
127.595
127.010
125.530
114.863
108.082
77.322
77.000
76.685
52.963
52.667
52.648
19.726
COOMe
CN
NO2
Chemical Formula: C17H14N2O4
Exact Mass: 310.0954
150
100
50
0 PPM
xql-2-146
USER: -- DATE: May 21 2013
F1: 100.507
F2: 399.673
SW1: 21186
OF1: 9541.3
PTS1d: 32768 , 32768
EX: s2pul
PW: 5.3 us
PD: 3.0 sec
NA: 64
LB: 2.0
Nuts - $_pslabel_02.fid

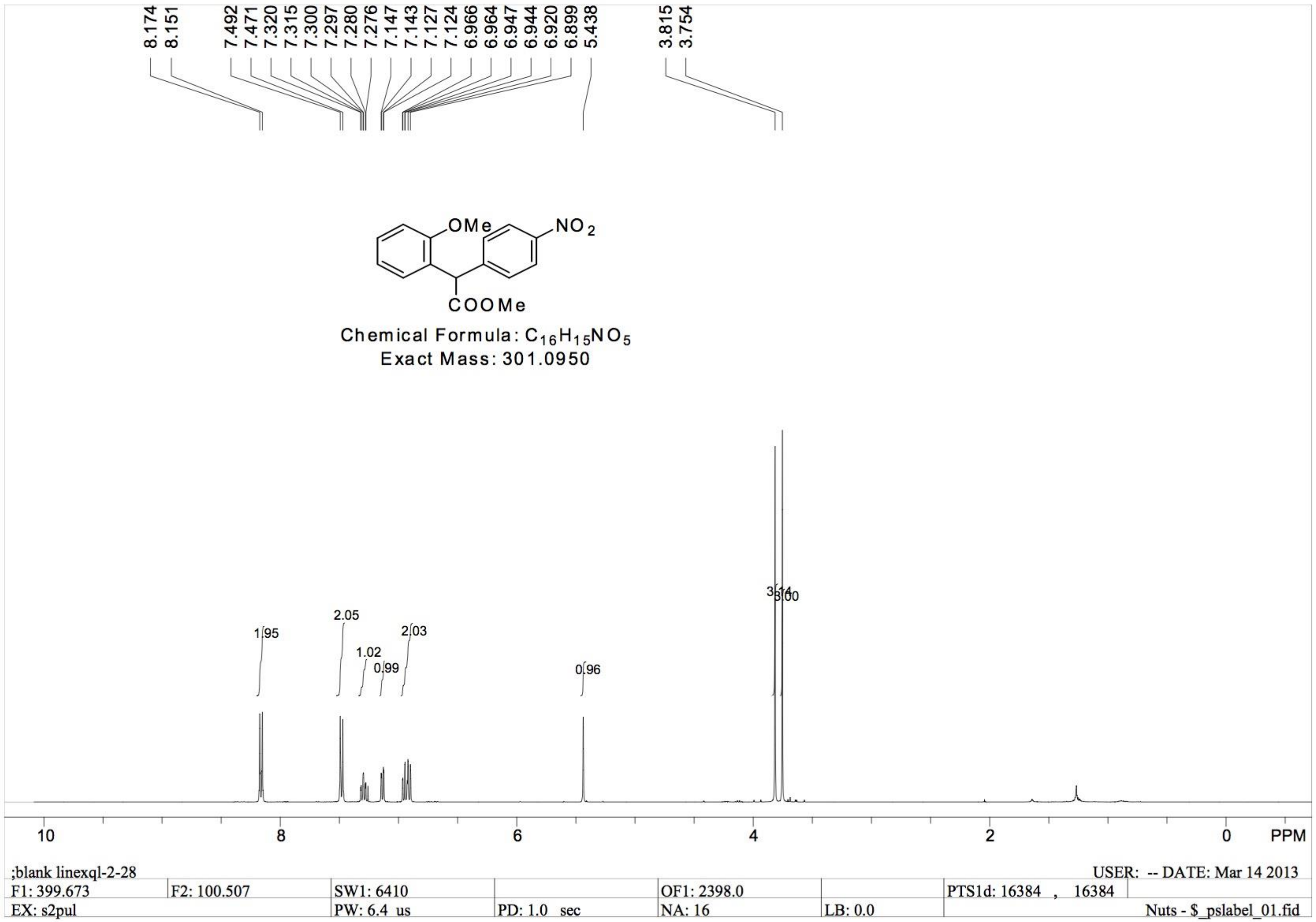

8.174
8.151
7.492
7.471
7.320
7.315
7.300
7.297
7.280
7.276
7.147
7.143
7.127
7.124
6.966
6.964
6.947
6.944
6.920
6.899
5.438
3.815
3.754
OMe
NO2
COOMe
Chemical Formula: C16H15NO5
Exact Mass: 301.0950
1.95
2.05
1.02
0.99
2.03
0.96
3.14
3.00
10
8
6
4
2
0
PPM
;blank linexql-2-28
USER: -- DATE: Mar 14 2013
F1: 399.673
F2: 100.507
SW1: 6410
OF1: 2398.0
PTS1d: 16384 , 16384
EX: s2pul
PW: 6.4 us
PD: 1.0 sec
NA: 16
LB: 0.0
Nuts - $_pslabel_01.fid

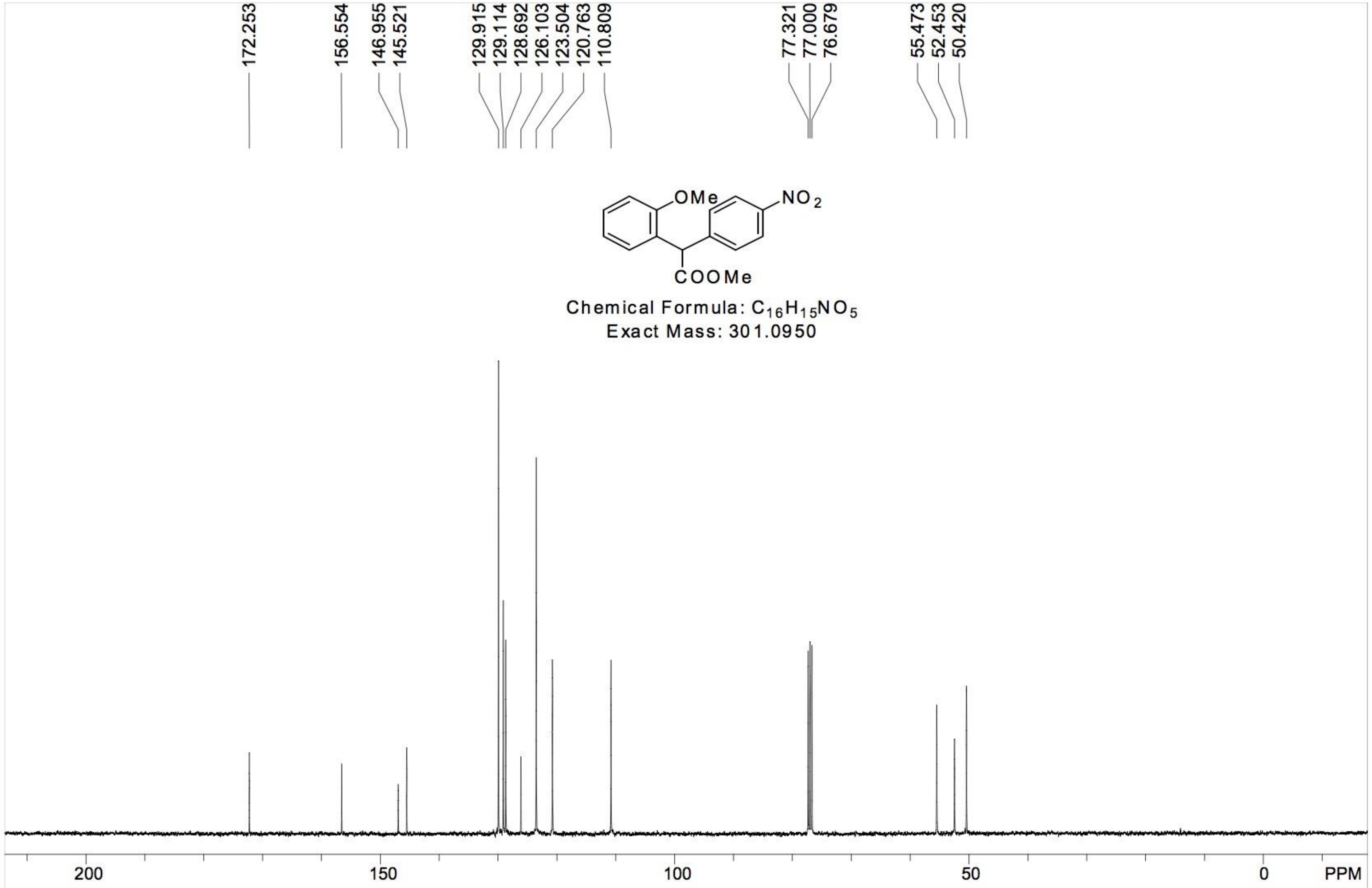

;blank linexql-2-28 USER: -- DATE: Mar 14 2013

F1: 100.509	F2: 399.673	SW1: 27778		OF1: 11298.4		PTS1d: 32768 , 32768	
EX: s2pul		PW: 5.3 us	PD: 1.0 sec	NA: 256	LB: 2.0		Nuts - $_pslabel_02.fid

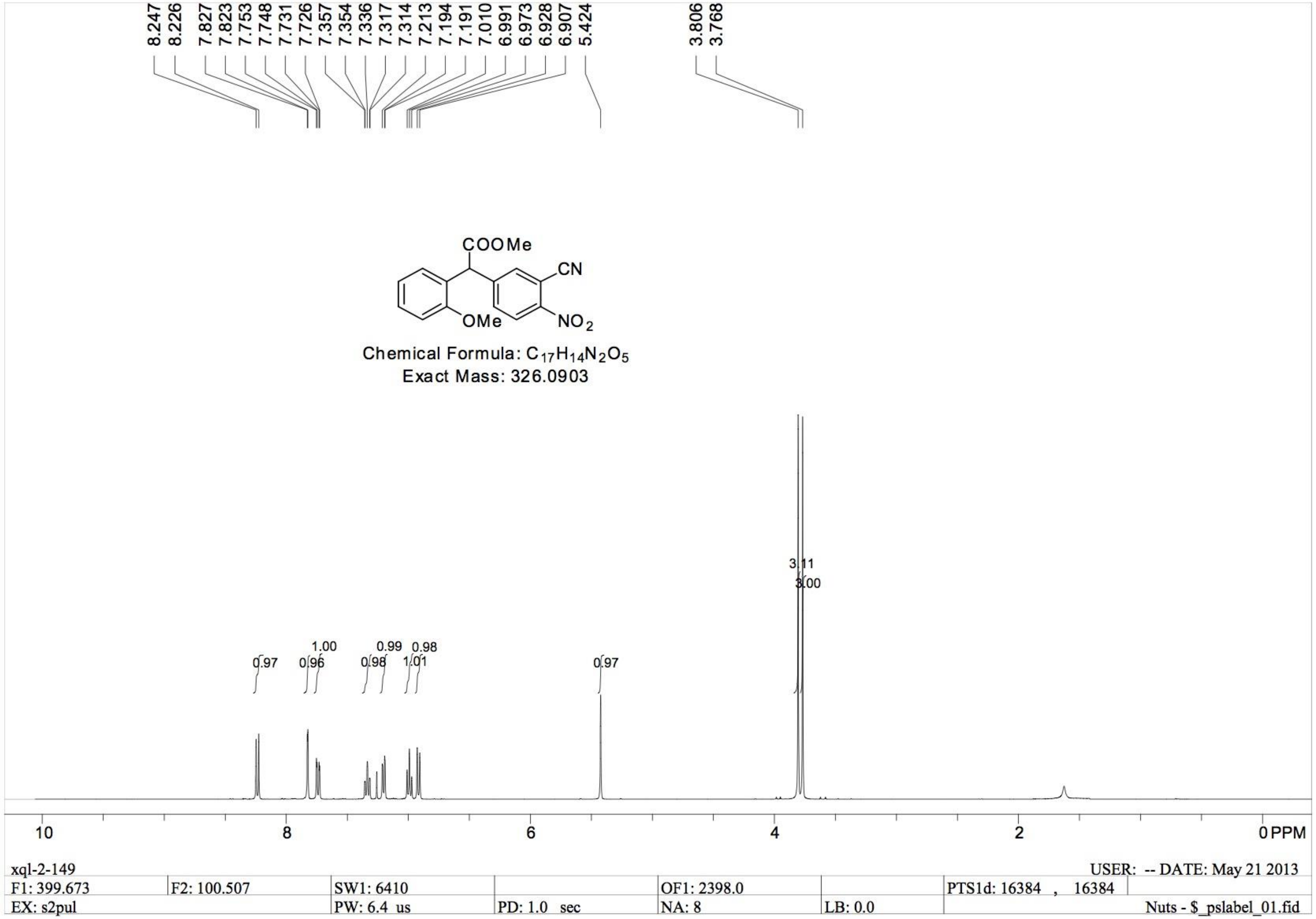

8.247 8.226 7.827 7.823 7.753 7.748 7.731 7.726 7.357 7.354 7.336 7.317 7.314 7.213 7.194 7.191 7.010 6.991 6.973 6.928 6.907 5.424
3.806 3.768
COOMe
CN
OMe
NO2
Chemical Formula: C17H14N2O5
Exact Mass: 326.0903
3.11
3.00
0.97 0.96 1.00 0.98 0.99 1.01 0.98 0.97
10 8 6 4 2 0 PPM
xql-2-149
USER: -- DATE: May 21 2013
F1: 399.673
F2: 100.507
SW1: 6410
OF1: 2398.0
PTS1d: 16384 , 16384
EX: s2pul
PW: 6.4 us
PD: 1.0 sec
NA: 8
LB: 0.0
Nuts - $_pslabel_01.fid

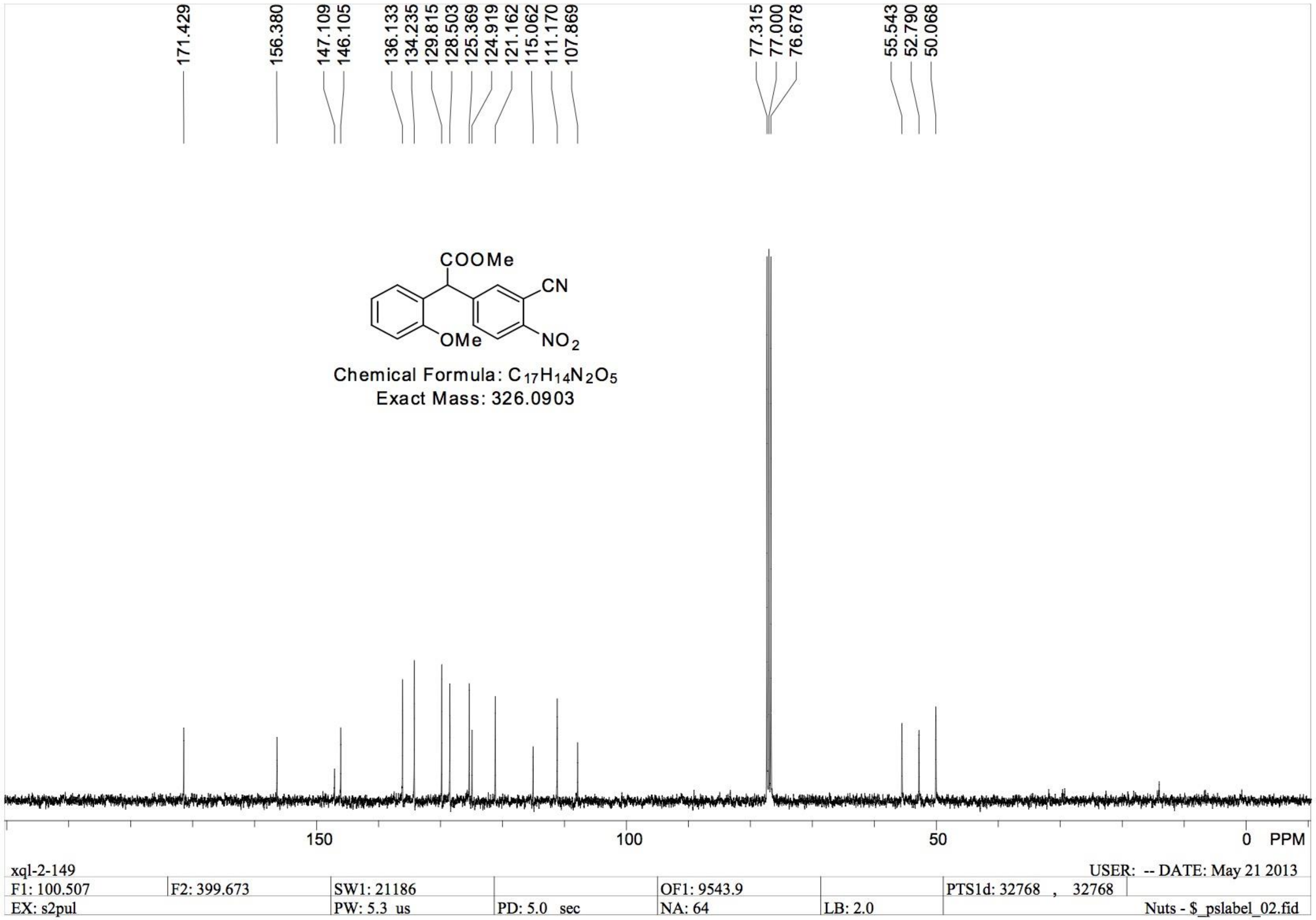
171.429
156.380
147.109
146.105
136.133
134.235
129.815
128.503
125.369
124.919
121.162
115.062
111.170
107.869
77.315
77.000
76.678
55.543
52.790
50.068
COOMe
CN
OMe
NO2
Chemical Formula: C17H14N2O5
Exact Mass: 326.0903
150
100
50
0 PPM
xql-2-149
USER: -- DATE: May 21 2013
F1: 100.507
F2: 399.673
SW1: 21186
OF1: 9543.9
PTS1d: 32768 , 32768
EX: s2pul
PW: 5.3 us
PD: 5.0 sec
NA: 64
LB: 2.0
Nuts - $_pslabel_02.fid

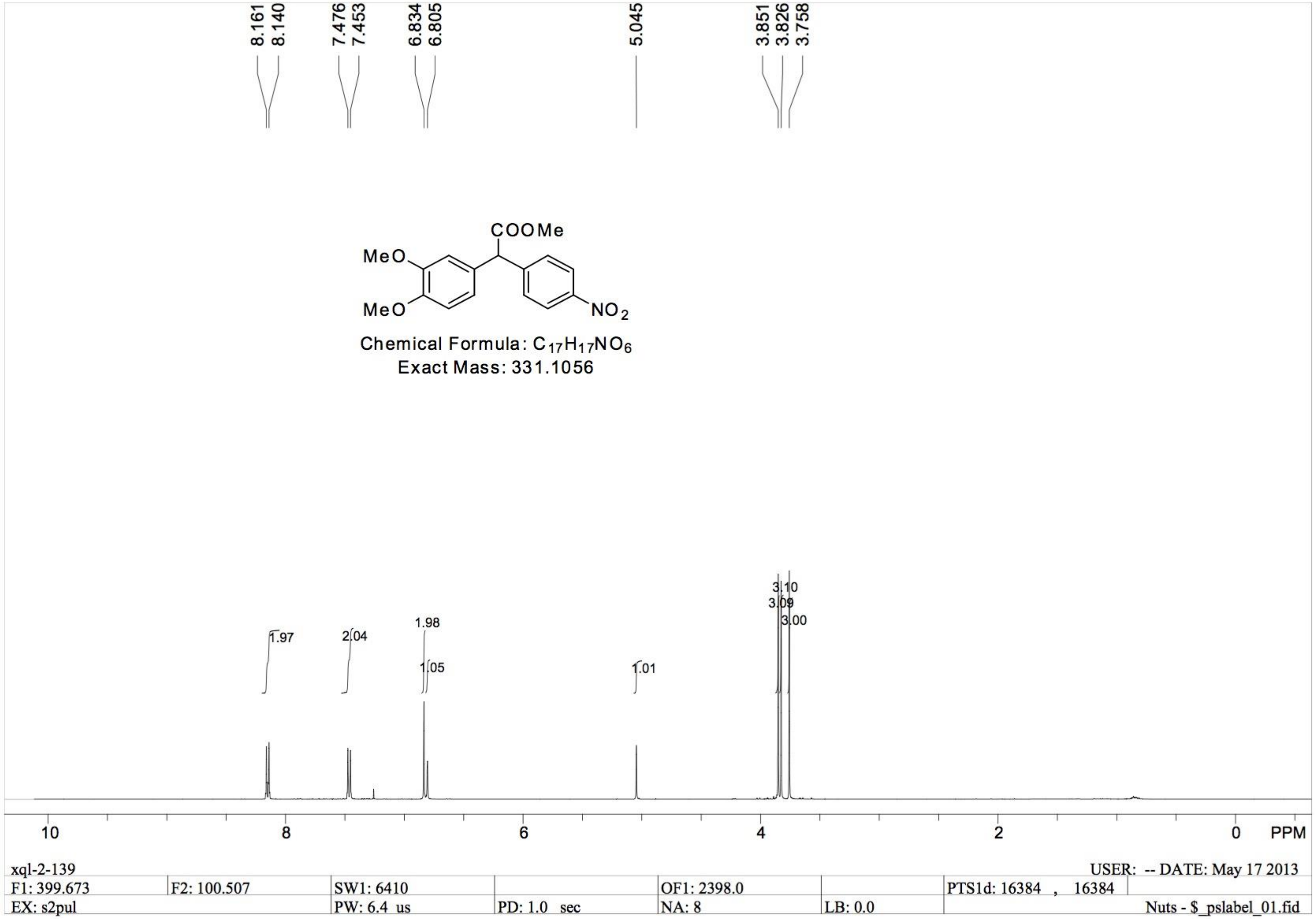
8.161
8.140
7.476
7.453
6.834
6.805
5.045
3.851
3.826
3.758
COOMe
MeO
MeO
NO2
Chemical Formula: C17H17NO6
Exact Mass: 331.1056
1.97
2.04
1.98
1.05
1.01
3.10
3.09
3.00
10
8
6
4
2
0
PPM
xql-2-139
USER: -- DATE: May 17 2013
F1: 399.673
F2: 100.507
SW1: 6410
OF1: 2398.0
PTS1d: 16384 , 16384
EX: s2pul
PW: 6.4 us
PD: 1.0 sec
NA: 8
LB: 0.0
Nuts - $_pslabel_01.fid

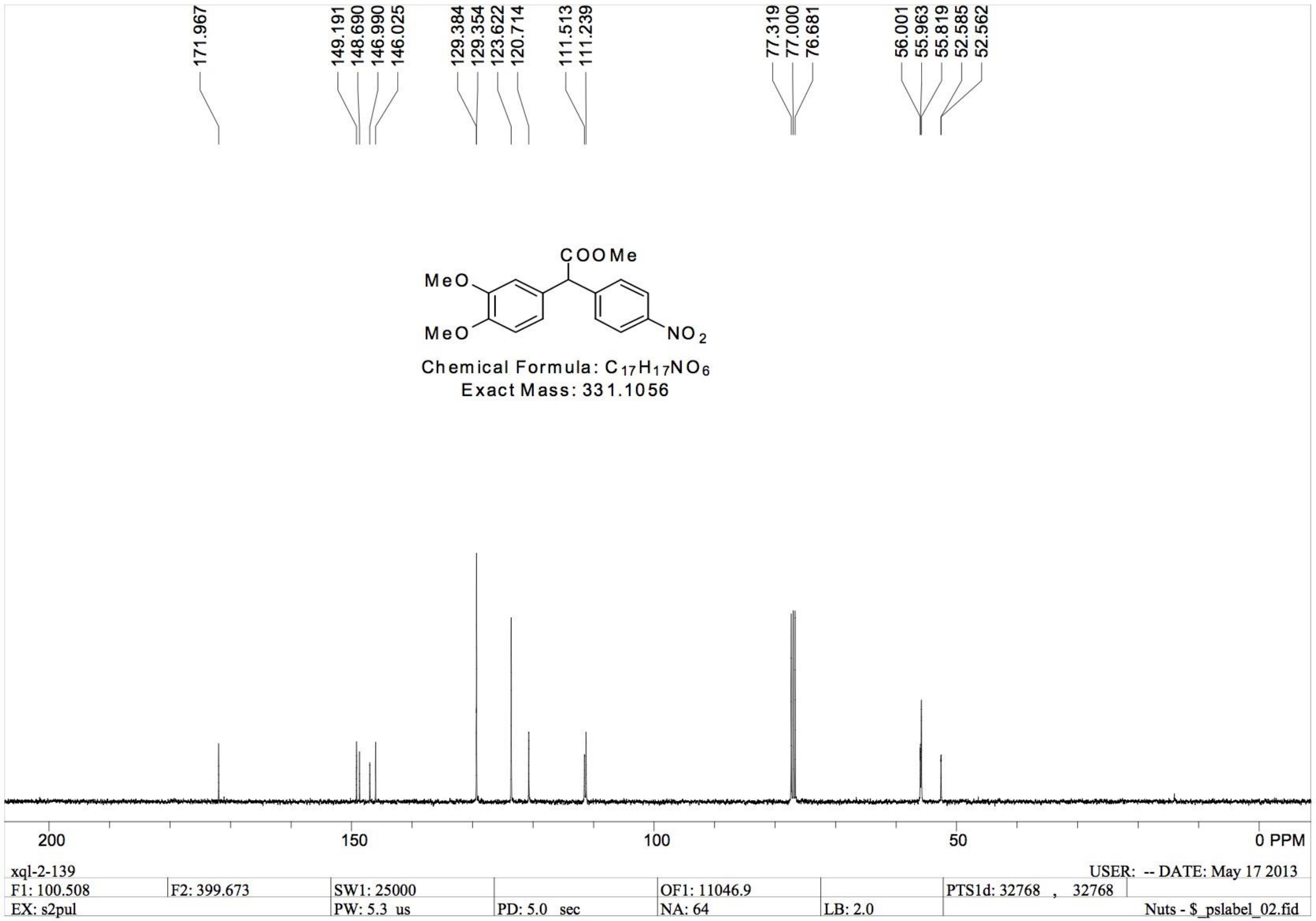

171.967
149.191
148.690
146.990
146.025
129.384
129.354
123.622
120.714
111.513
111.239
77.319
77.000
76.681
56.001
55.963
55.819
52.585
52.562
COOMe
MeO
MeO
NO2
Chemical Formula: C17H17NO6
Exact Mass: 331.1056
200
150
100
50
0 PPM
xql-2-139
USER: -- DATE: May 17 2013
F1: 100.508
F2: 399.673
SW1: 25000
OF1: 11046.9
PTS1d: 32768 , 32768
EX: s2pul
PW: 5.3 us
PD: 5.0 sec
NA: 64
LB: 2.0
Nuts - $_pslabel_02.fid

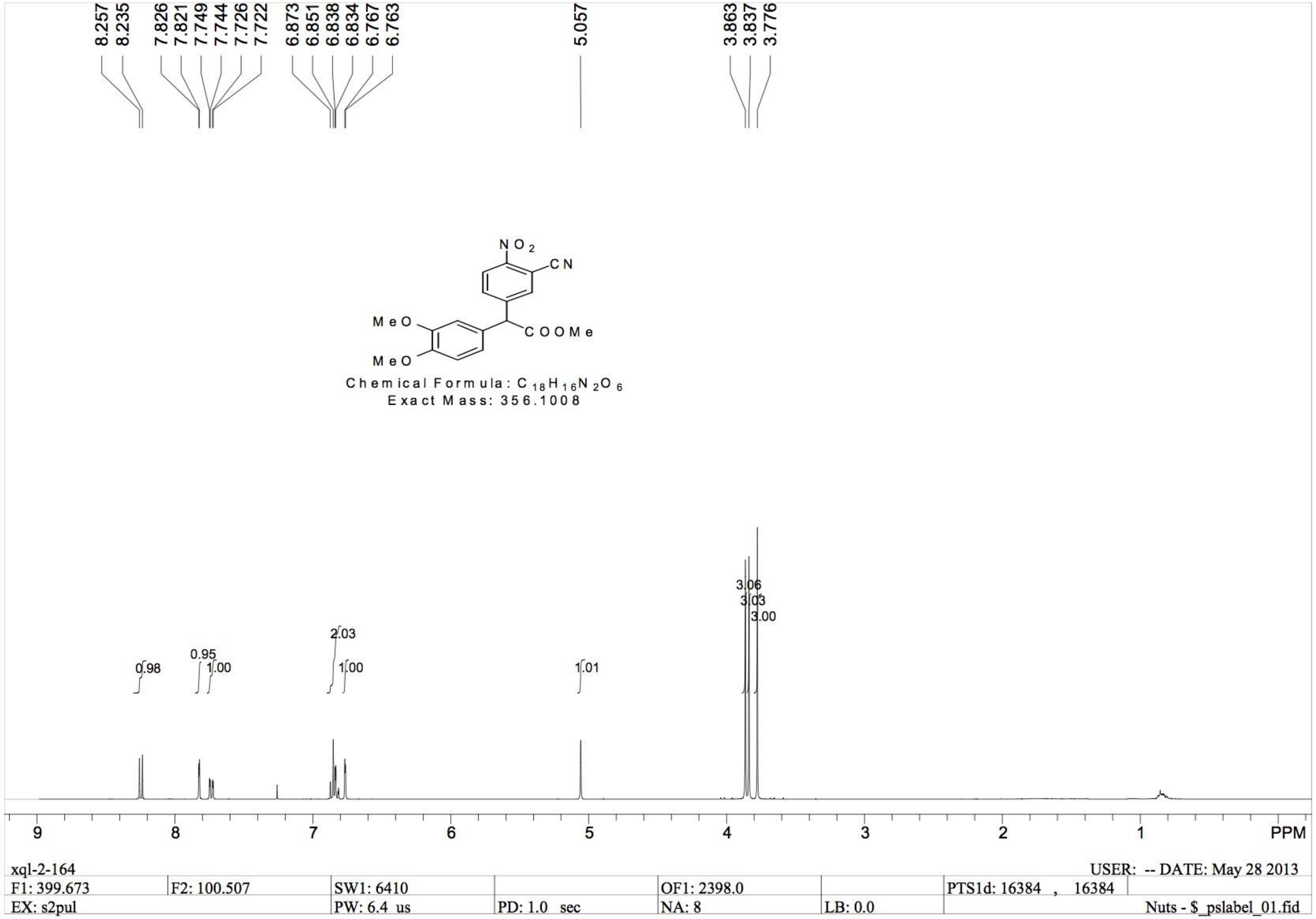
8.257
8.235
7.826
7.821
7.749
7.744
7.726
7.722
6.873
6.851
6.838
6.834
6.767
6.763
5.057
3.863
3.837
3.776
NO2
CN
MeO
MeO
COOMe
Chemical Formula: C18H16N2O6
Exact Mass: 356.1008
0.98
0.95
1.00
2.03
1.00
1.01
3.06
3.03
3.00
9
8
7
6
5
4
3
2
1
PPM
xql-2-164
USER: -- DATE: May 28 2013
F1: 399.673
F2: 100.507
SW1: 6410
OF1: 2398.0
PTS1d: 16384 , 16384
EX: s2pul
PW: 6.4 us
PD: 1.0 sec
NA: 8
LB: 0.0
Nuts - $_pslabel_01.fid

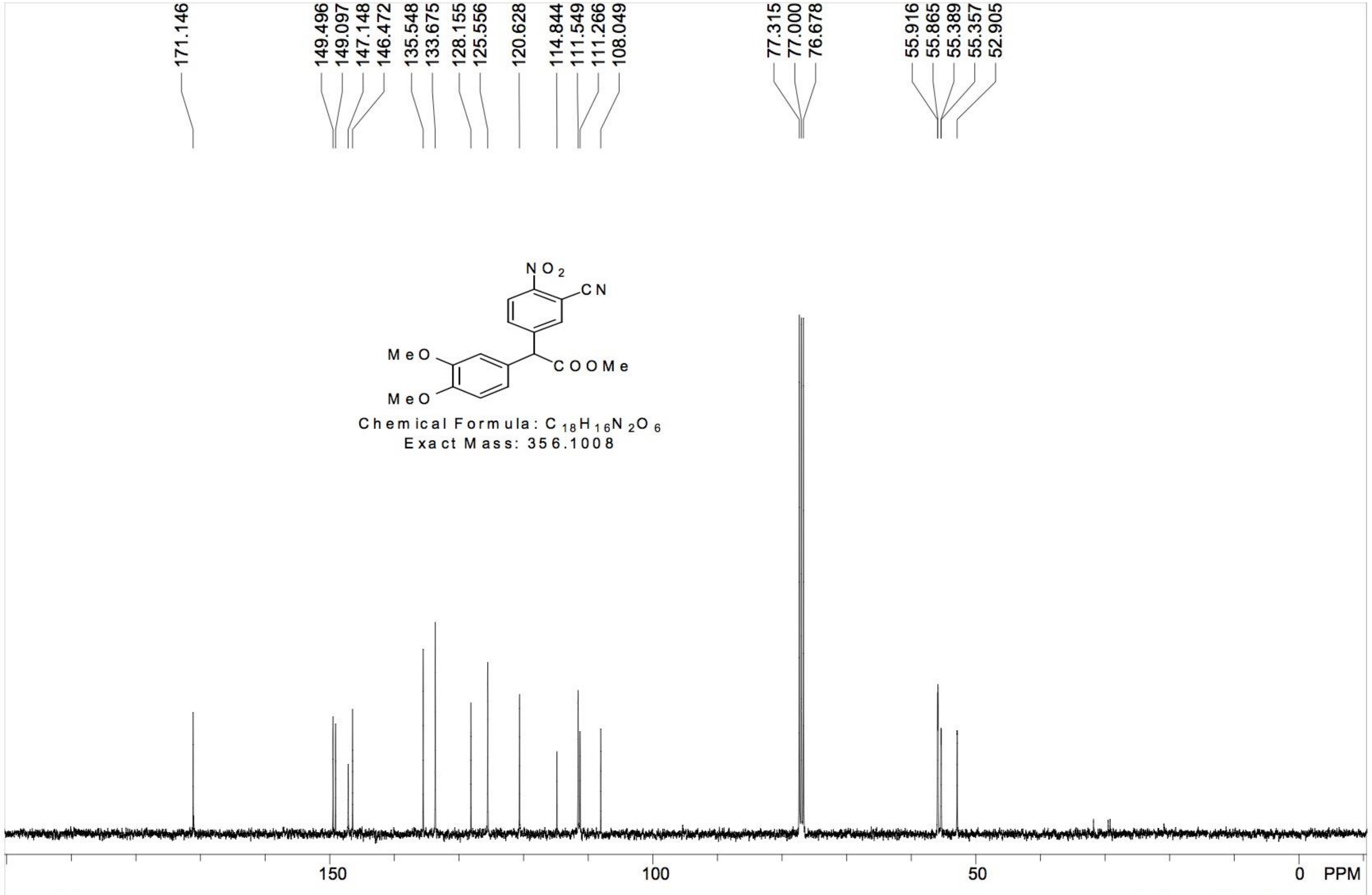

xql-2-164							USER: -- DATE: May 28 2013
F1: 100.507	F2: 399.673	SW1: 21186		OF1: 9539.3		PTS1d: 32768 , 32768	
EX: s2pul		PW: 5.3 us	PD: 5.0 sec	NA: 64	LB: 2.0		Nuts - $_pslabel_02.fid

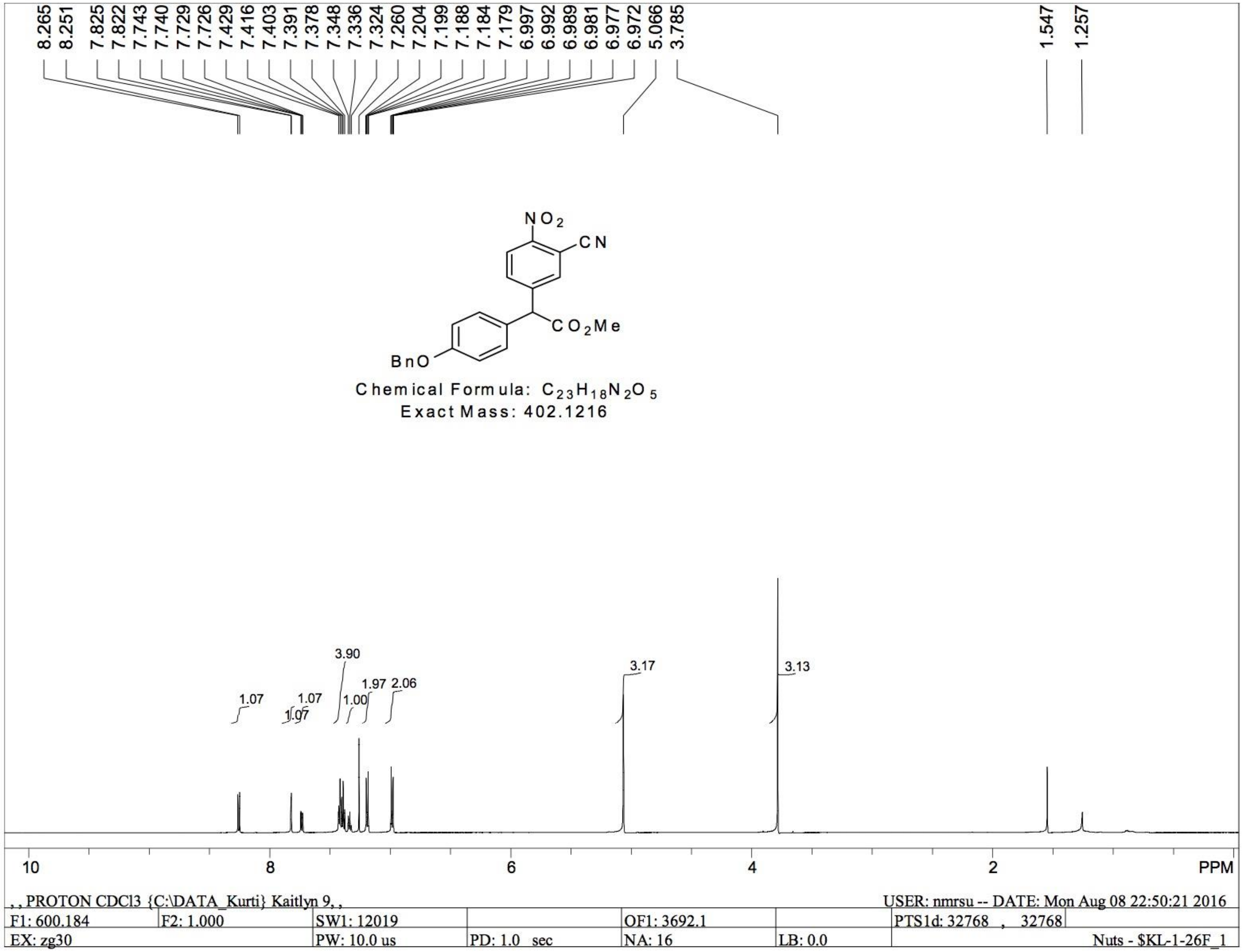
8.265
8.251
7.825
7.822
7.743
7.740
7.729
7.726
7.429
7.416
7.403
7.391
7.378
7.348
7.336
7.324
7.260
7.204
7.199
7.188
7.184
7.179
6.997
6.992
6.989
6.981
6.977
6.972
5.066
3.785
1.547
1.257
NO2
CN
CO2Me
BnO
Chemical Formula: C23H18N2O5
Exact Mass: 402.1216
1.07
1.07
1.07
3.90
1.00
1.97
2.06
3.17
3.13
10
8
6
4
2
PPM
, , PROTON CDCl3 {C:\DATA_Kurti} Kaitlyn 9, ,
USER: nmrsu -- DATE: Mon Aug 08 22:50:21 2016
F1: 600.184
F2: 1.000
SW1: 12019
OF1: 3692.1
PTS1d: 32768 , 32768
EX: zg30
PW: 10.0 us
PD: 1.0 sec
NA: 16
LB: 0.0
Nuts - $KL-1-26F_1

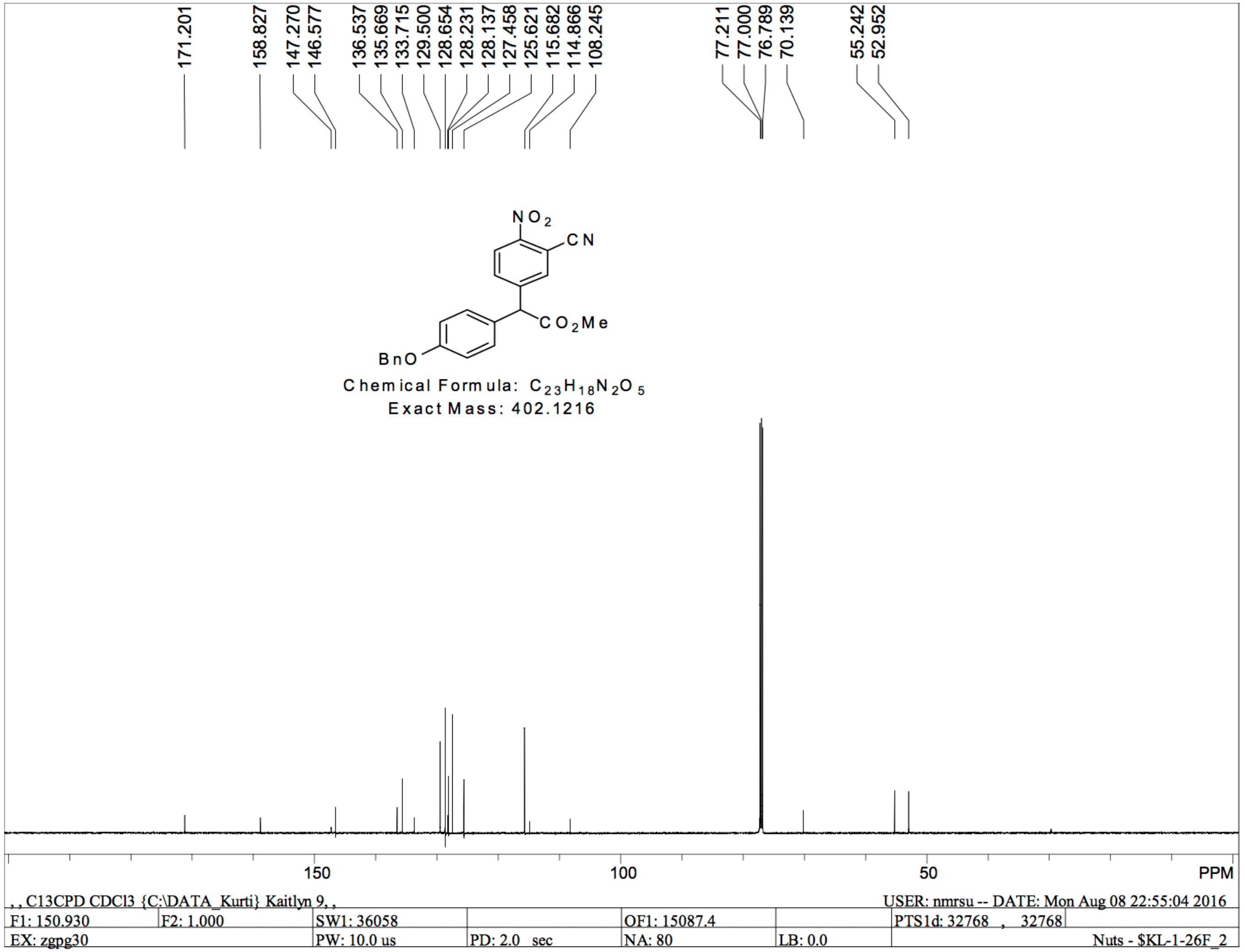

171.201
158.827
147.270
146.577
136.537
135.669
133.715
129.500
128.654
128.231
128.137
127.458
125.621
115.682
114.866
108.245
77.211
77.000
76.789
70.139
55.242
52.952
NO2
CN
CO2Me
BnO
Chemical Formula: C23H18N2O5
Exact Mass: 402.1216
150
100
50
PPM
, , C13CPD CDCl3 {C:\DATA_Kurti} Kaitlyn 9, ,
USER: nmrsu -- DATE: Mon Aug 08 22:55:04 2016
F1: 150.930
F2: 1.000
SW1: 36058
OF1: 15087.4
PTS1d: 32768 , 32768
EX: zgpg30
PW: 10.0 us
PD: 2.0 sec
NA: 80
LB: 0.0
Nuts - $KL-1-26F_2

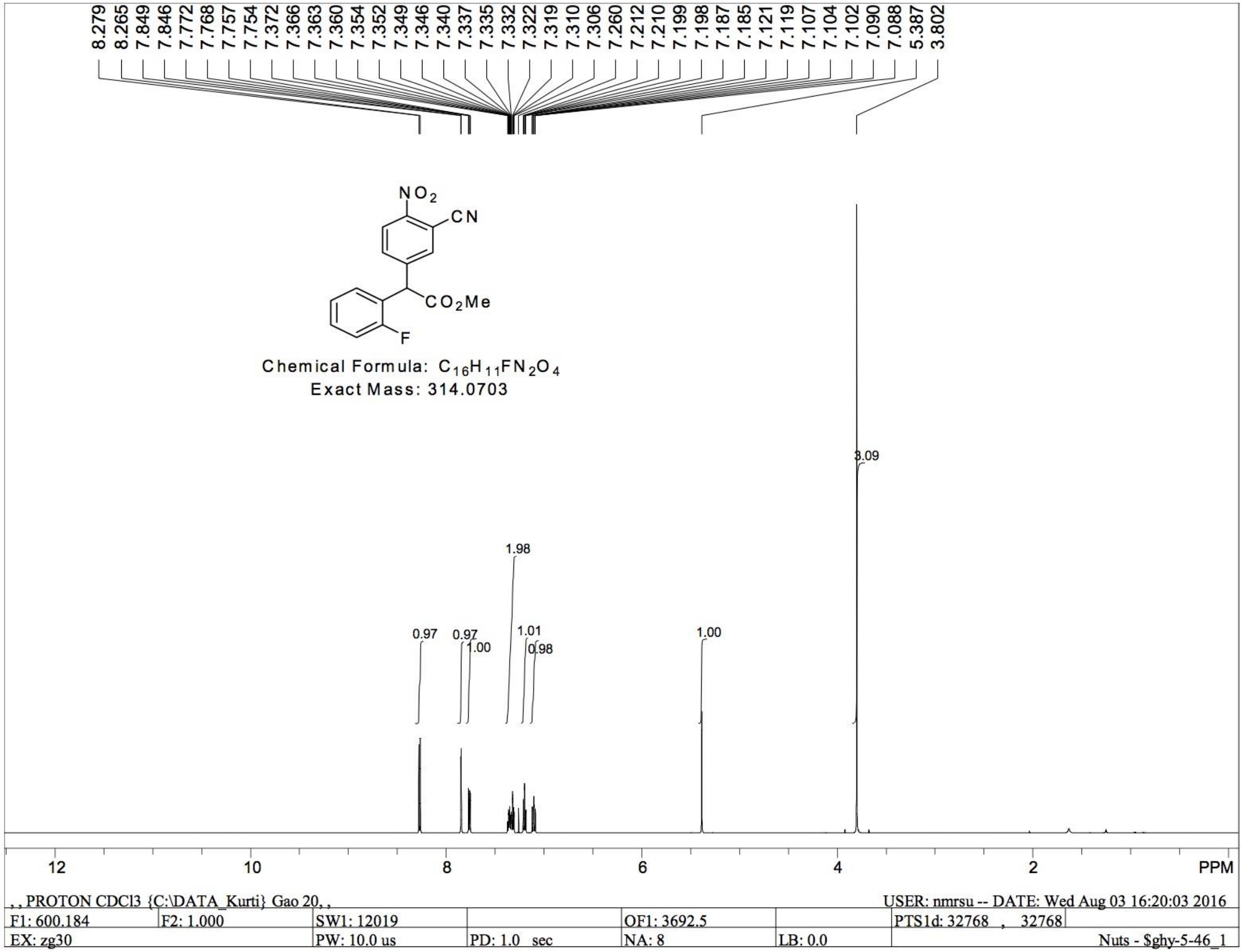
8.279
8.265
7.849
7.846
7.772
7.768
7.757
7.754
7.372
7.366
7.363
7.360
7.354
7.352
7.349
7.346
7.340
7.337
7.335
7.332
7.322
7.319
7.310
7.306
7.260
7.212
7.210
7.199
7.198
7.187
7.185
7.121
7.119
7.107
7.104
7.102
7.090
7.088
5.387
3.802
NO2
CN
CO2Me
F
Chemical Formula: C16H11FN2O4
Exact Mass: 314.0703
3.09
1.98
0.97
0.97
1.00
1.01
0.98
1.00
12
10
8
6
4
2
PPM
, , PROTON CDCl3 {C:\DATA_Kurti} Gao 20, ,
USER: nmrsu -- DATE: Wed Aug 03 16:20:03 2016
F1: 600.184
F2: 1.000
SW1: 12019
OF1: 3692.5
PTS1d: 32768 , 32768
EX: zg30
PW: 10.0 us
PD: 1.0 sec
NA: 8
LB: 0.0
Nuts - $ghy-5-46_1

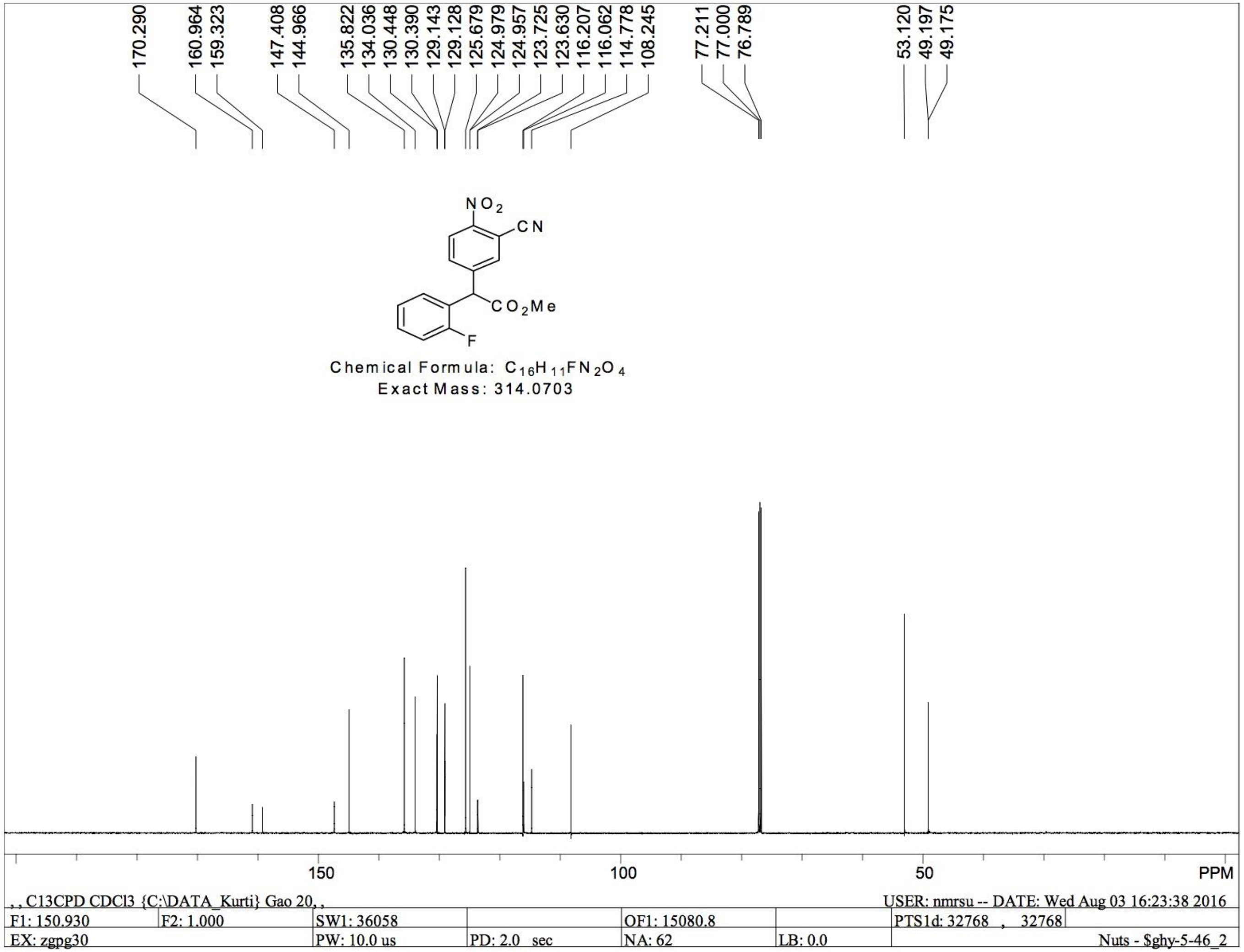
170.290
160.964
159.323
147.408
144.966
135.822
134.036
130.448
130.390
129.143
129.128
125.679
124.979
124.957
123.725
123.630
116.207
116.062
114.778
108.245
77.211
77.000
76.789
53.120
49.197
49.175
NO2
CN
CO2Me
F
Chemical Formula: C16H11FN2O4
Exact Mass: 314.0703
150
100
50
PPM
, , C13CPD CDCl3 {C:\DATA_Kurti} Gao 20, ,
USER: nmrsu -- DATE: Wed Aug 03 16:23:38 2016
F1: 150.930
F2: 1.000
SW1: 36058
OF1: 15080.8
PTS1d: 32768 , 32768
EX: zgpg30
PW: 10.0 us
PD: 2.0 sec
NA: 62
LB: 0.0
Nuts - $ghy-5-46_2

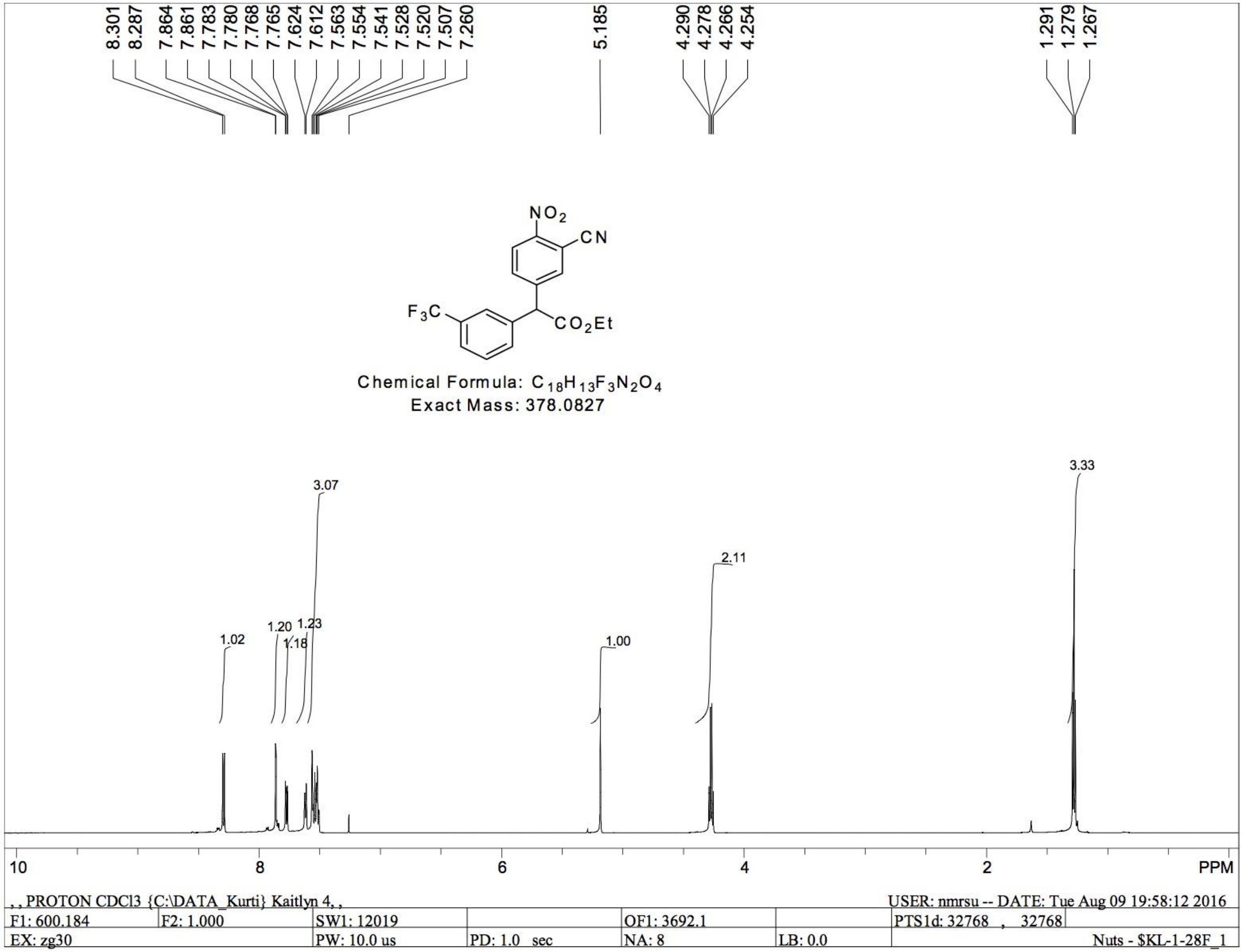
8.301 8.287 7.864 7.861 7.783 7.780 7.768 7.765 7.624 7.612 7.563 7.554 7.541 7.528 7.520 7.507 7.260
5.185
4.290 4.278 4.266 4.254
1.291 1.279 1.267
NO_2
CN
F_3C
CO_2Et
Chemical Formula: $C_{18}H_{13}F_3N_2O_4$
Exact Mass: 378.0827
3.07
3.33
2.11
1.02
1.20
1.23
1.18
1.00
10
8
6
4
2
PPM
, , PROTON CDCl3 {C:\DATA_Kurti} Kaitlyn 4, ,
USER: nmrsu -- DATE: Tue Aug 09 19:58:12 2016
F1: 600.184
F2: 1.000
SW1: 12019
OF1: 3692.1
PTS1d: 32768 , 32768
EX: zg30
PW: 10.0 us
PD: 1.0 sec
NA: 8
LB: 0.0
Nuts - $KL-1-28F_1

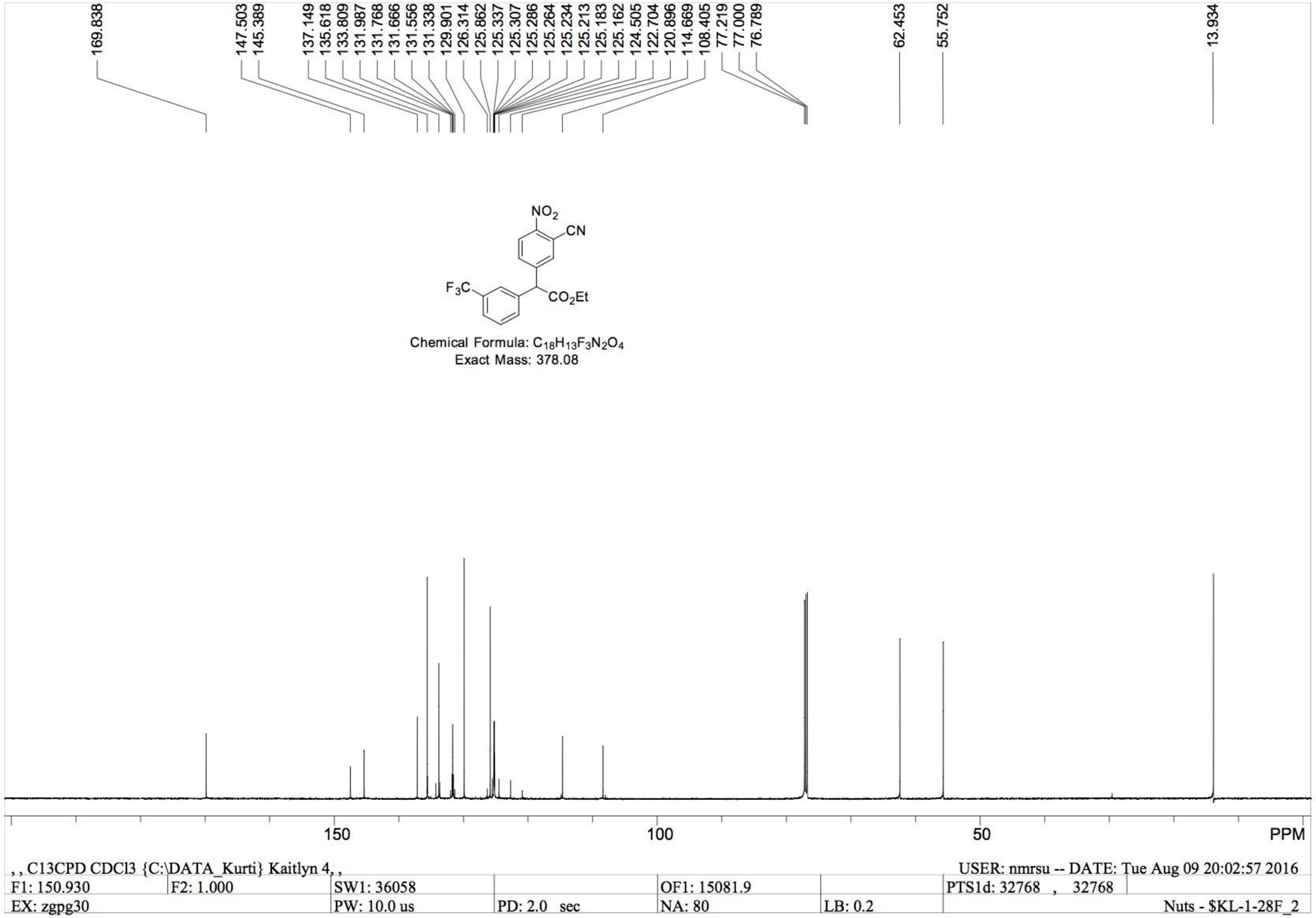
169.838
147.503
145.389
137.149
135.618
133.809
131.987
131.768
131.666
131.556
131.338
129.901
126.314
125.862
125.337
125.307
125.286
125.264
125.234
125.213
125.183
125.162
124.505
122.704
120.896
114.669
108.405
77.219
77.000
76.789
62.453
55.752
13.934
NO2
CN
F3C
CO2Et
Chemical Formula: C18H13F3N2O4
Exact Mass: 378.08
150
100
50
PPM
, , C13CPD CDCl3 {C:\DATA_Kurti} Kaitlyn 4, ,
USER: nmrsu -- DATE: Tue Aug 09 20:02:57 2016
F1: 150.930
F2: 1.000
SW1: 36058
OF1: 15081.9
PTS1d: 32768 , 32768
EX: zgpg30
PW: 10.0 us
PD: 2.0 sec
NA: 80
LB: 0.2
Nuts - $KL-1-28F_2

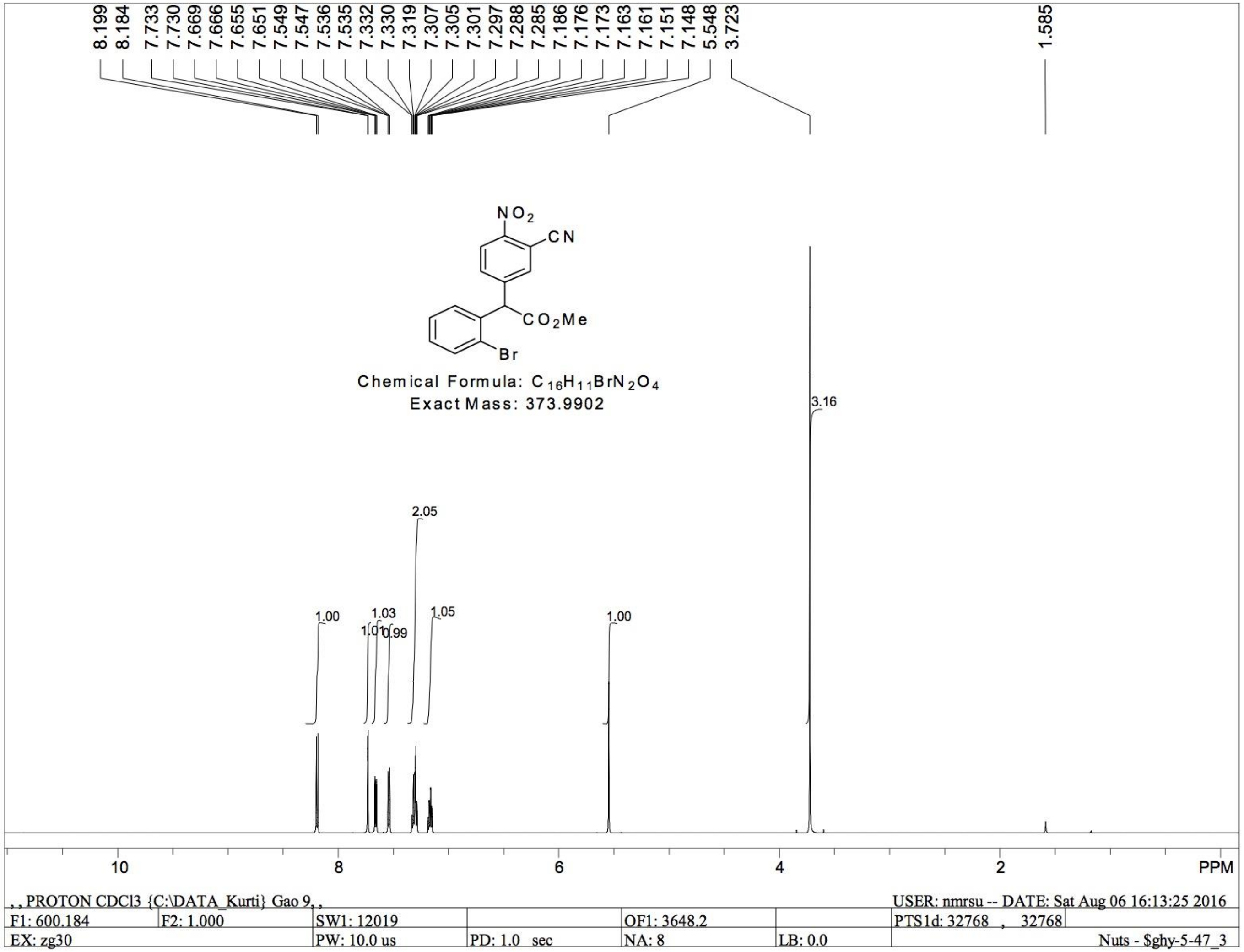
8.199
8.184
7.733
7.730
7.669
7.666
7.655
7.651
7.549
7.547
7.536
7.535
7.332
7.330
7.319
7.307
7.305
7.301
7.297
7.288
7.285
7.186
7.176
7.173
7.163
7.161
7.151
7.148
5.548
3.723
1.585
NO2
CN
CO2Me
Br
Chemical Formula: C16H11BrN2O4
Exact Mass: 373.9902
3.16
2.05
1.00
1.03
1.01
0.99
1.05
1.00
10
8
6
4
2
PPM
, , PROTON CDCl3 {C:\DATA_Kurti} Gao 9, ,
USER: nmrsu -- DATE: Sat Aug 06 16:13:25 2016
F1: 600.184
F2: 1.000
SW1: 12019
OF1: 3648.2
PTS1d: 32768 , 32768
EX: zg30
PW: 10.0 us
PD: 1.0 sec
NA: 8
LB: 0.0
Nuts - $ghy-5-47_3

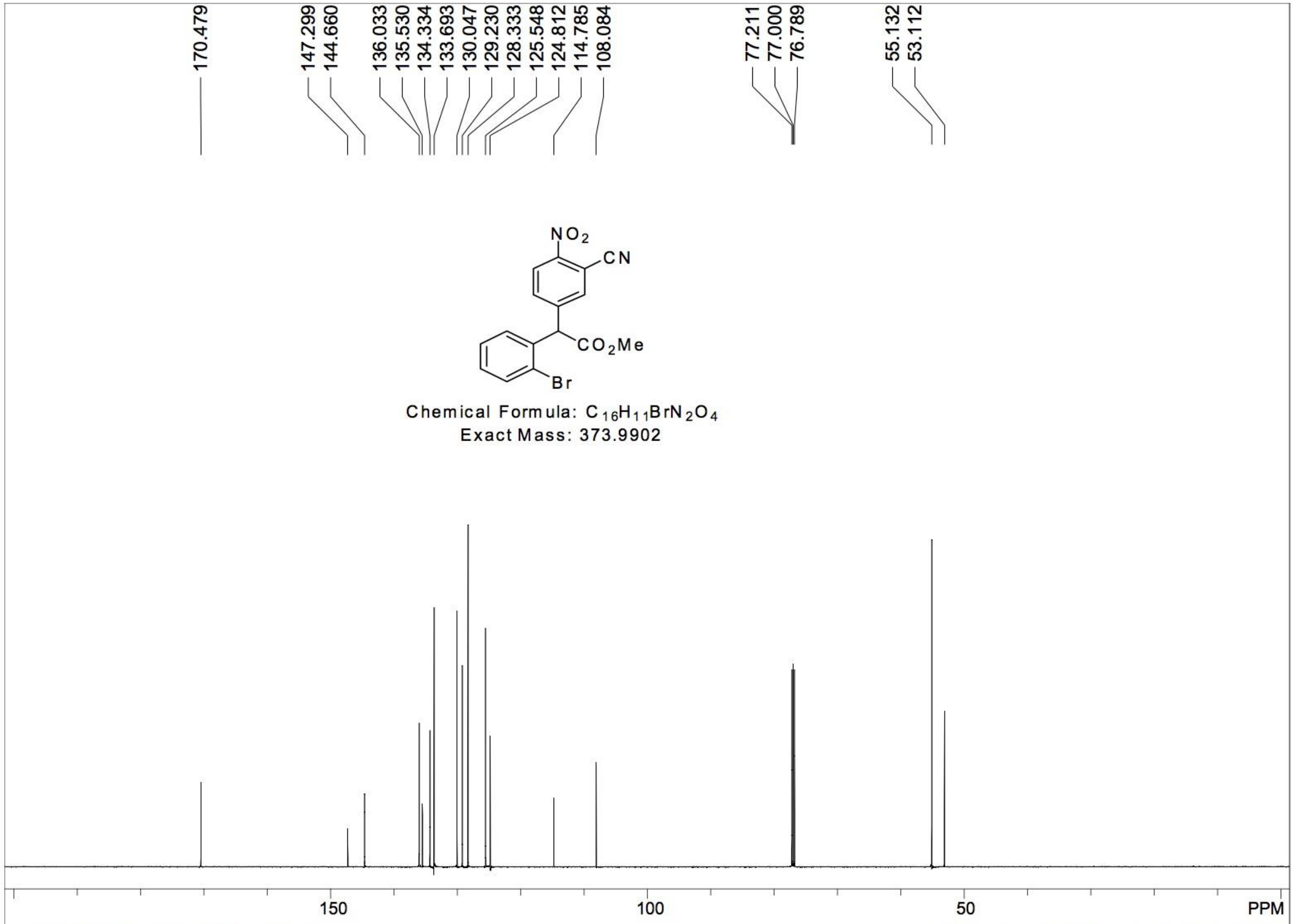

, , C13CPD CDCl3 {C:\DATA_Kurti} Gao 9, , USER: nmrsu -- DATE: Sat Aug 06 16:17:09 2016

F1: 150.930	F2: 1.000	SW1: 36058		OF1: 15073.1		PTS1d: 32768 , 32768
EX: zgpg30		PW: 10.0 us	PD: 2.0 sec	NA: 60	LB: 0.0	Nuts - $ghy-5-47_4

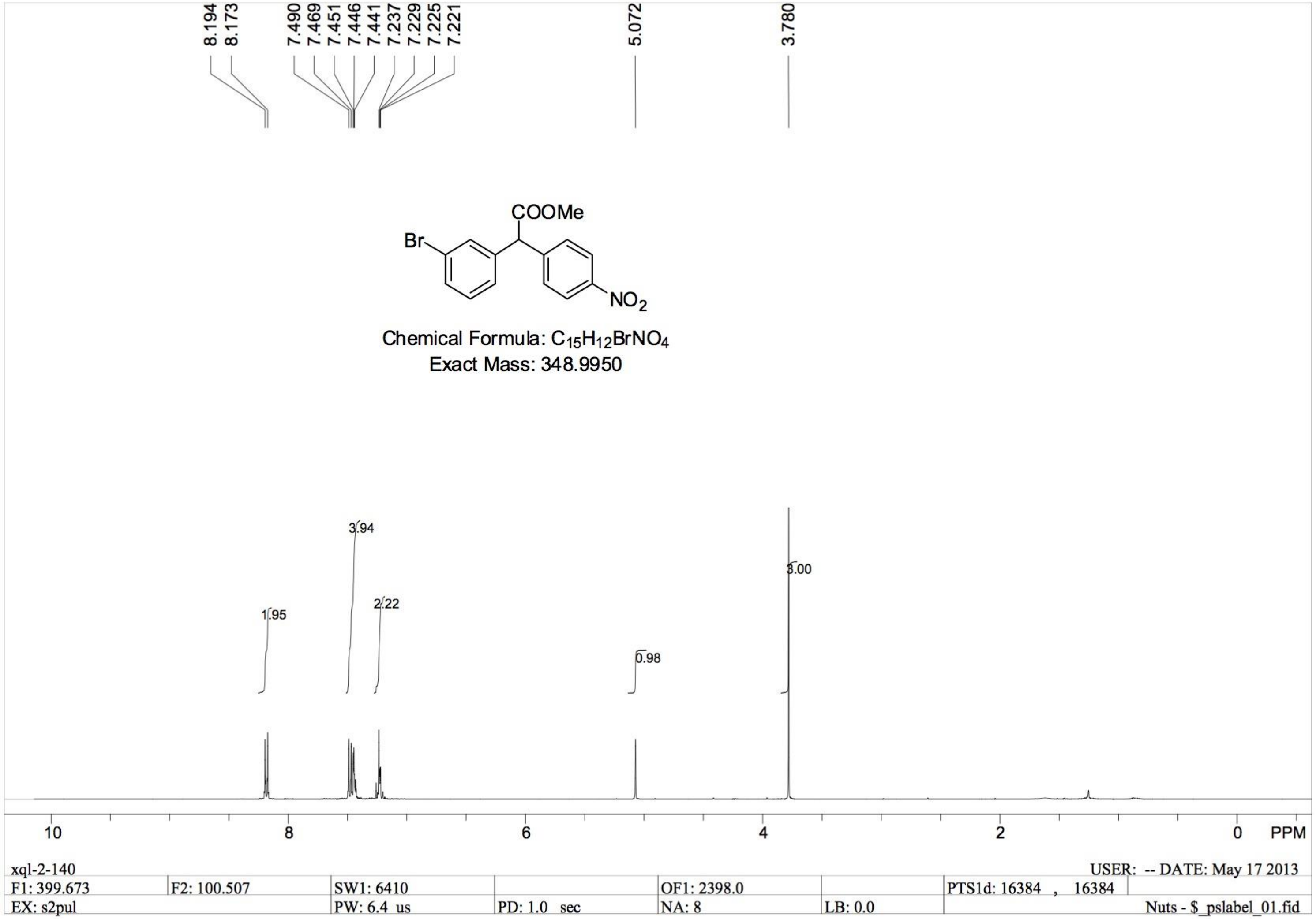
8.194
8.173
7.490
7.469
7.451
7.446
7.441
7.237
7.229
7.225
7.221
5.072
3.780
COOMe
Br
NO_2
Chemical Formula: $C_{15}H_{12}BrNO_4$
Exact Mass: 348.9950
1.95
3.94
2.22
0.98
3.00
10
8
6
4
2
0
PPM
xql-2-140
USER: -- DATE: May 17 2013
F1: 399.673
F2: 100.507
SW1: 6410
OF1: 2398.0
PTS1d: 16384 , 16384
EX: s2pul
PW: 6.4 us
PD: 1.0 sec
NA: 8
LB: 0.0
Nuts - $_pslabel_01.fid

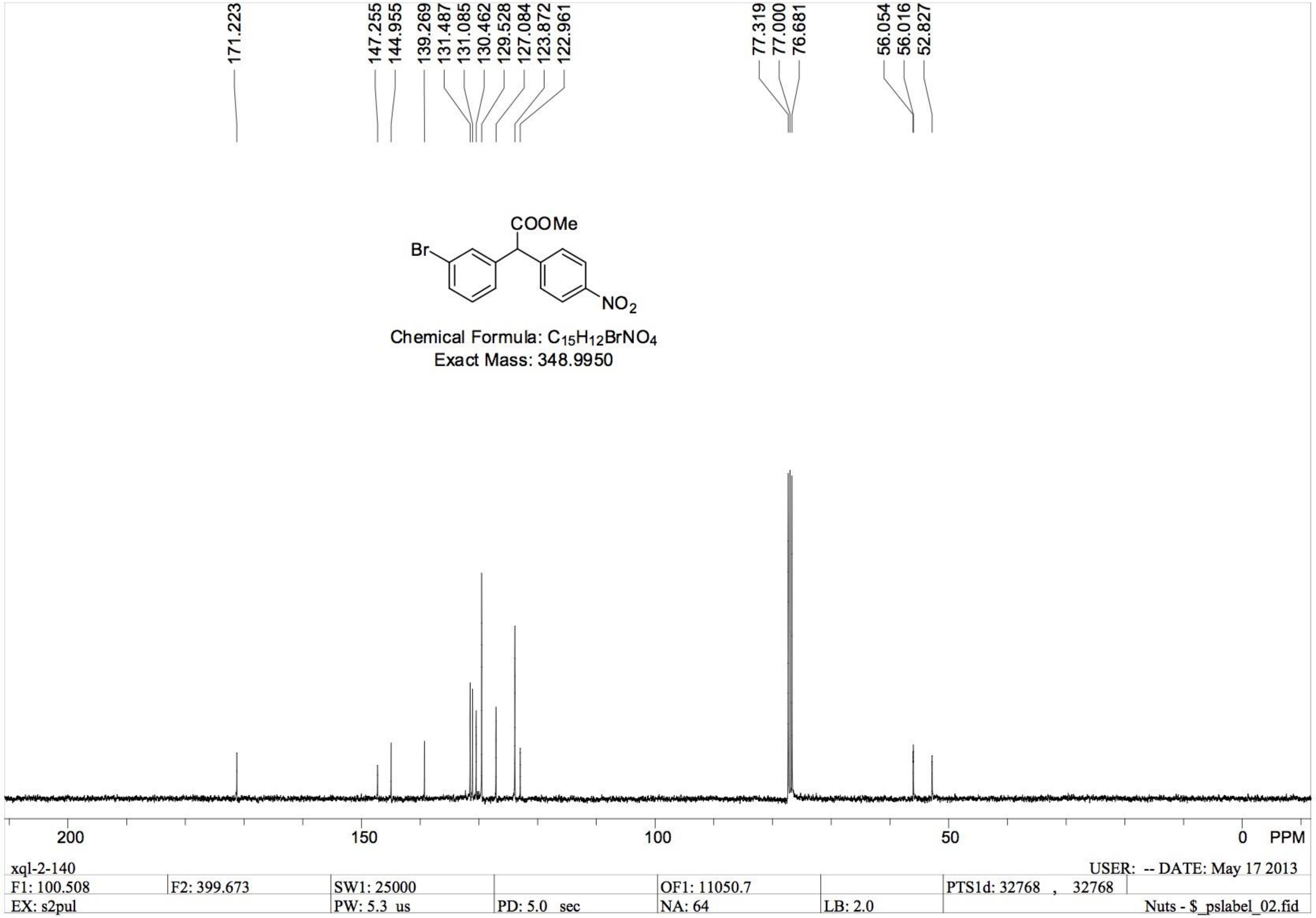
171.223
147.255
144.955
139.269
131.487
131.085
130.462
129.528
127.084
123.872
122.961
77.319
77.000
76.681
56.054
56.016
52.827
COOMe
Br
NO2
Chemical Formula: C15H12BrNO4
Exact Mass: 348.9950
200
150
100
50
0 PPM
xql-2-140
USER: -- DATE: May 17 2013
F1: 100.508
F2: 399.673
SW1: 25000
OF1: 11050.7
PTS1d: 32768 , 32768
EX: s2pul
PW: 5.3 us
PD: 5.0 sec
NA: 64
LB: 2.0
Nuts - $_pslabel_02.fid

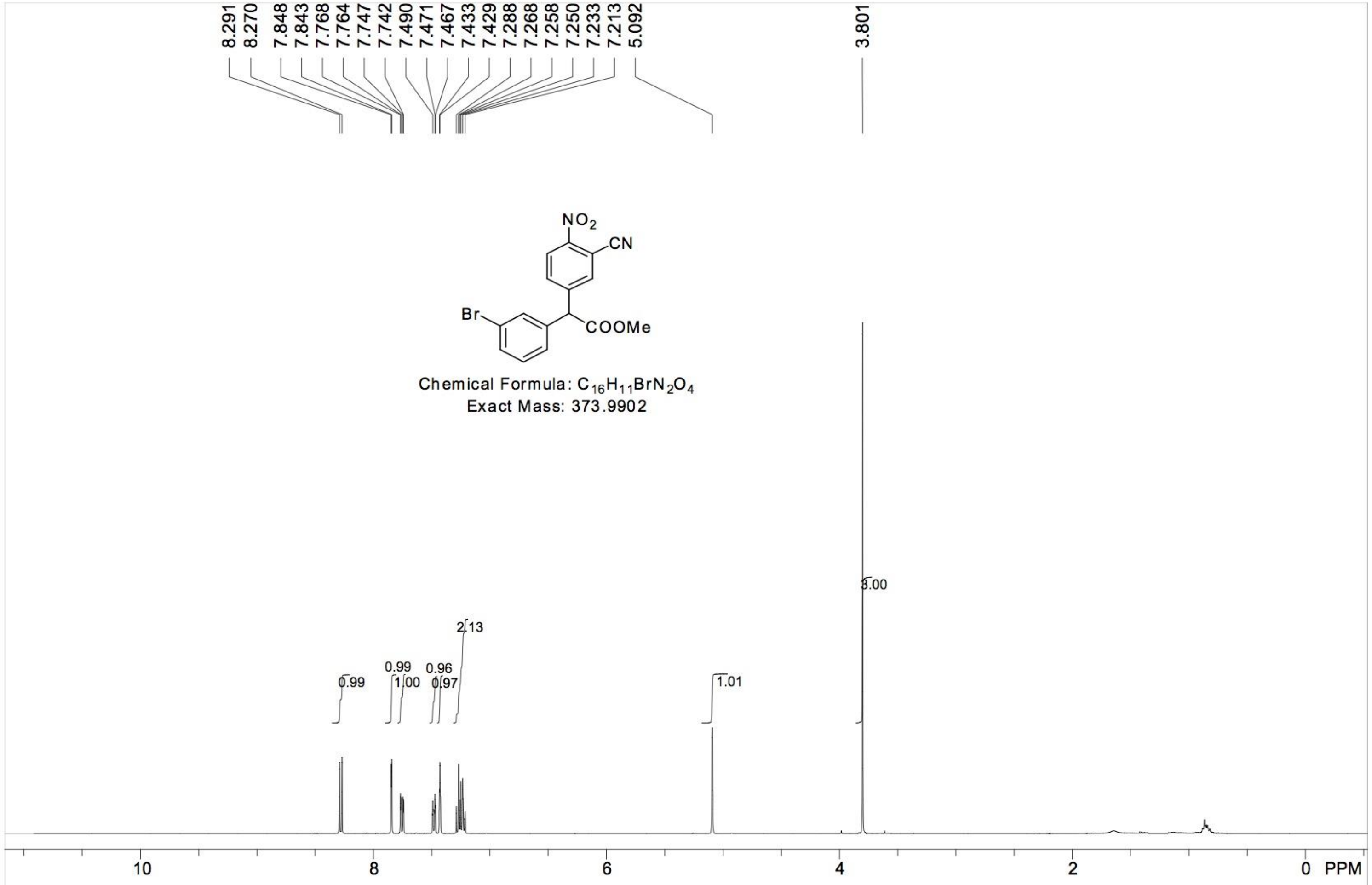

xql-2-163-1 USER: -- DATE: May 28 2013

F1: 399.673	F2: 100.507	SW1: 6410		OF1: 2398.0		PTS1d: 16384 , 16384	
EX: s2pul		PW: 6.4 us	PD: 2.0 sec	NA: 8	LB: 0.0		Nuts - $_pslabel_01.fid

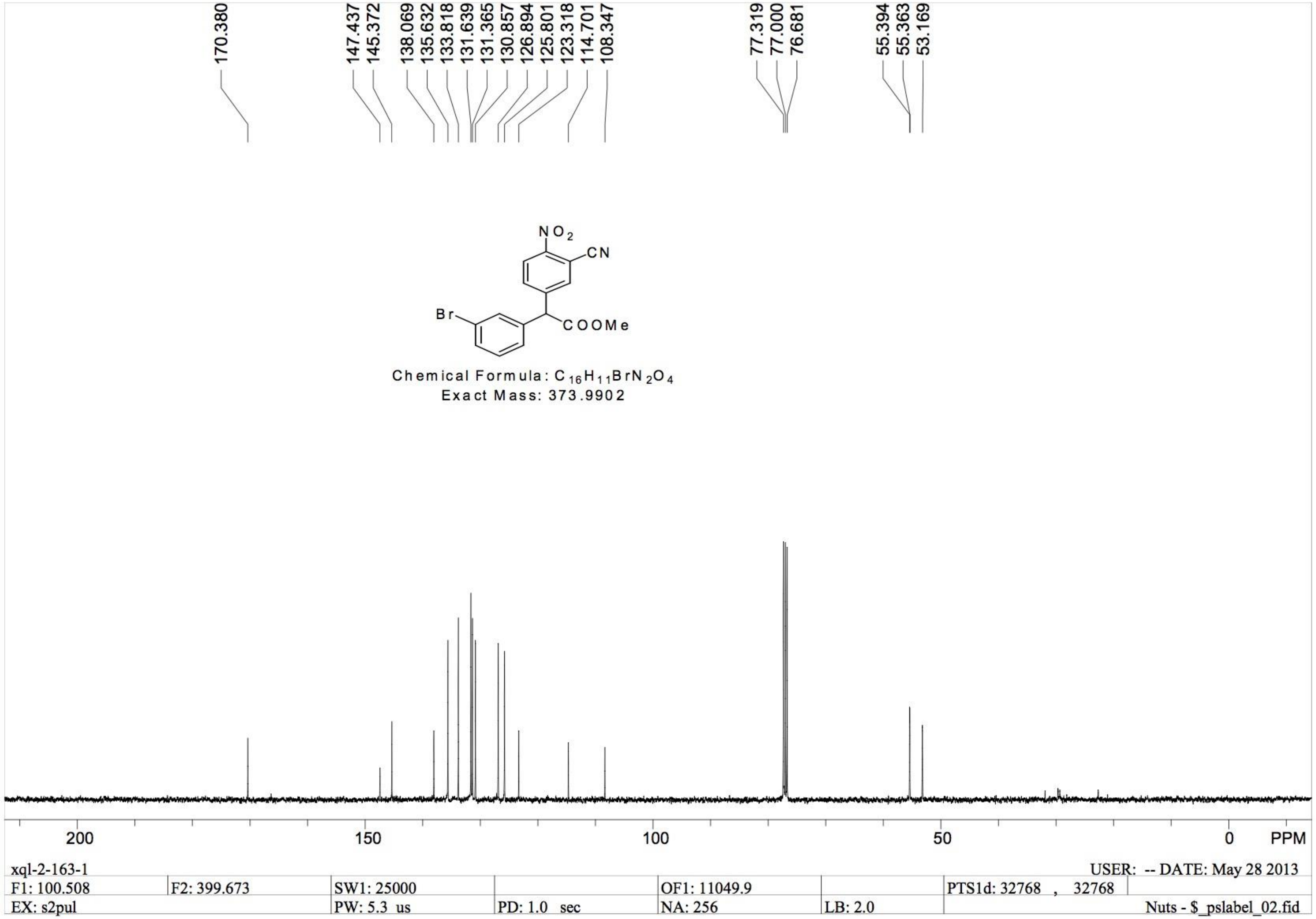
170.380
147.437
145.372
138.069
135.632
133.818
131.639
131.365
130.857
126.894
125.801
123.318
114.701
108.347
77.319
77.000
76.681
55.394
55.363
53.169
NO2
CN
Br
COOMe
Chemical Formula: C16H11BrN2O4
Exact Mass: 373.9902
200
150
100
50
0
PPM
xql-2-163-1
USER: -- DATE: May 28 2013
F1: 100.508
F2: 399.673
SW1: 25000
OF1: 11049.9
PTS1d: 32768 , 32768
EX: s2pul
PW: 5.3 us
PD: 1.0 sec
NA: 256
LB: 2.0
Nuts - $_pslabel_02.fid

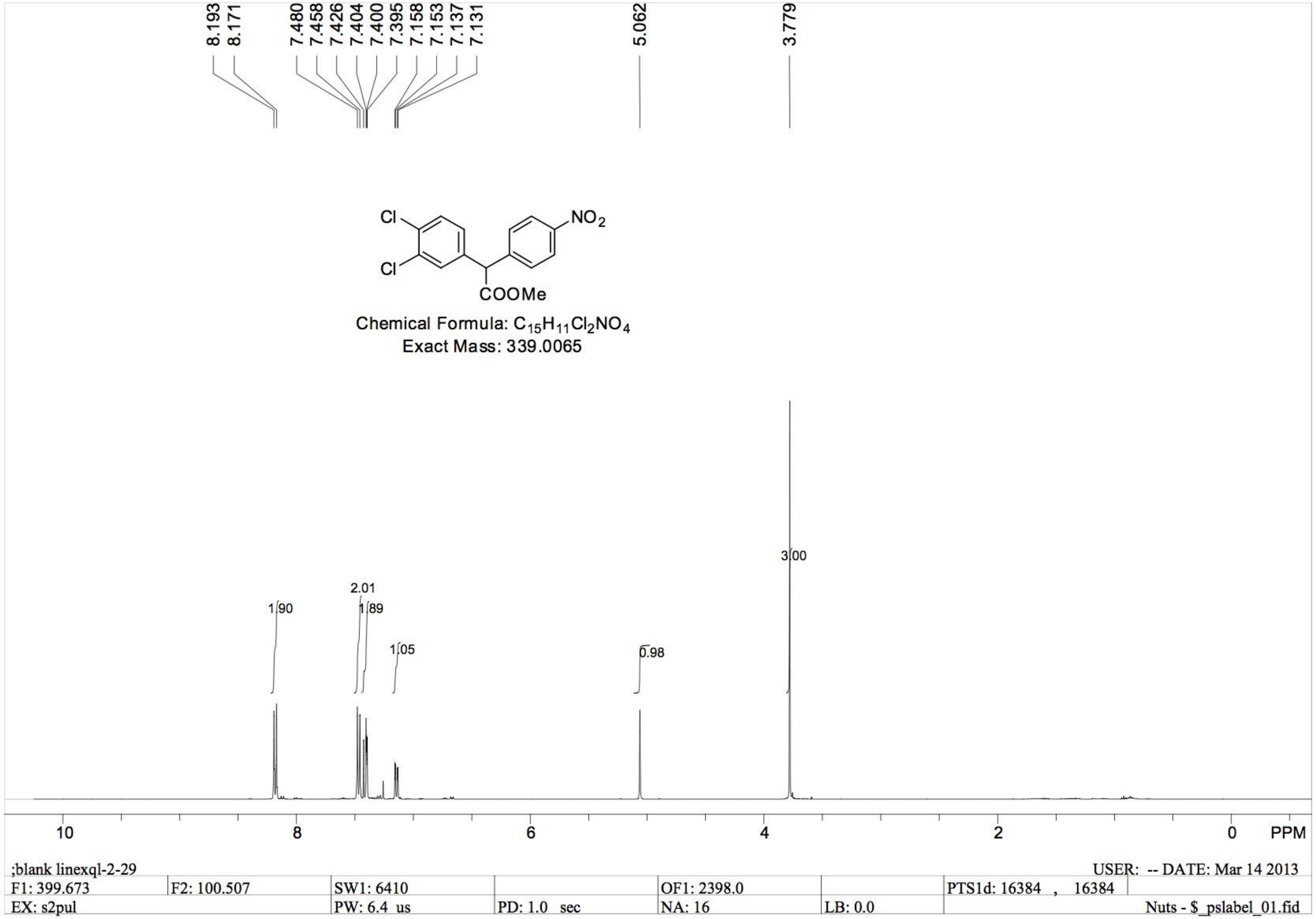
8.193
8.171
7.480
7.458
7.426
7.404
7.400
7.395
7.158
7.153
7.137
7.131
5.062
3.779
Cl
Cl
NO2
COOMe
Chemical Formula: C15H11Cl2NO4
Exact Mass: 339.0065
1.90
2.01
1.89
1.05
0.98
3.00
10
8
6
4
2
0
PPM
;blank linexql-2-29
USER: -- DATE: Mar 14 2013
F1: 399.673
F2: 100.507
SW1: 6410
OF1: 2398.0
PTS1d: 16384 , 16384
EX: s2pul
PW: 6.4 us
PD: 1.0 sec
NA: 16
LB: 0.0
Nuts - $_pslabel_01.fid

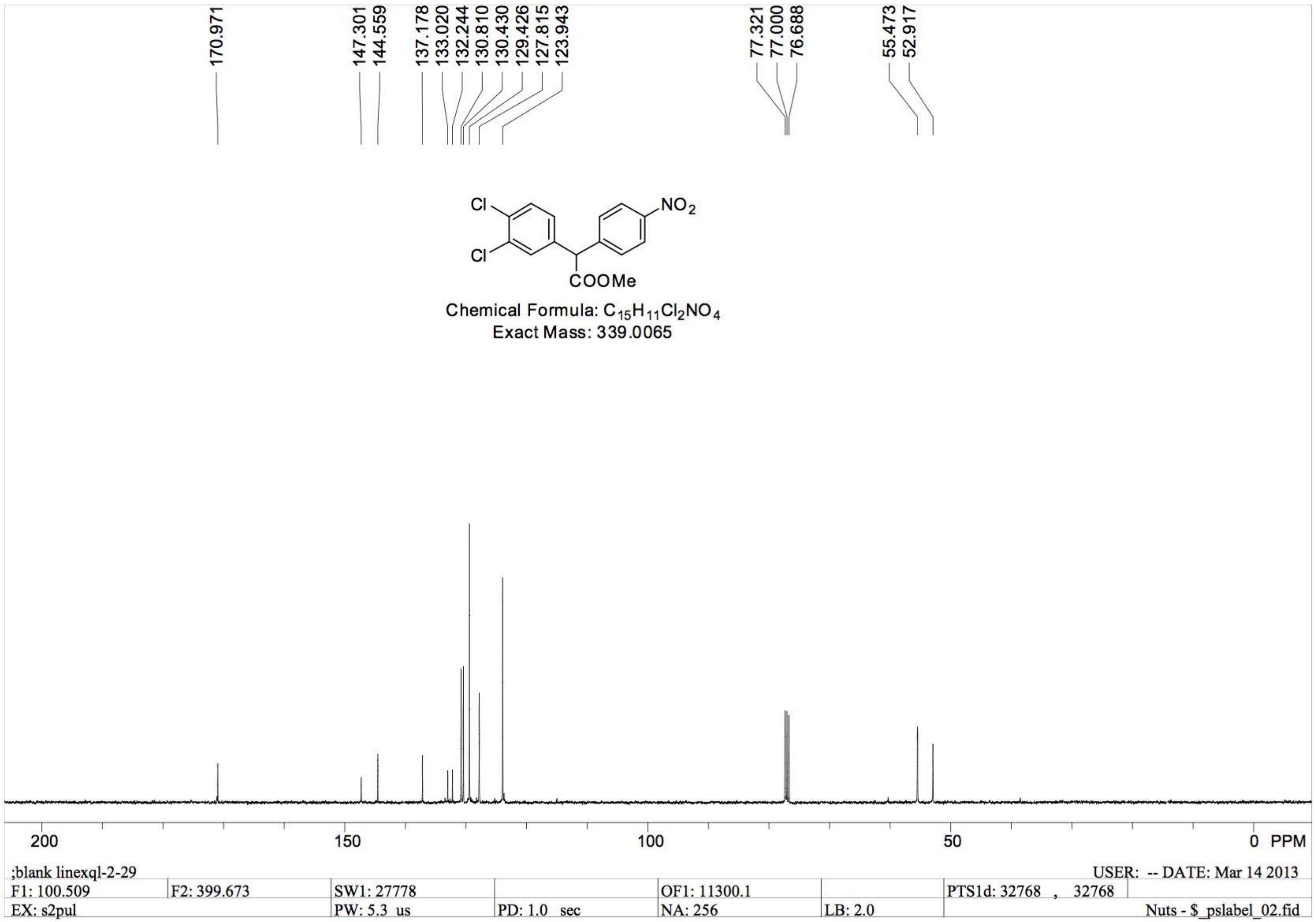
170.971
147.301
144.559
137.178
133.020
132.244
130.810
130.430
129.426
127.815
123.943
77.321
77.000
76.688
55.473
52.917
Cl
Cl
NO2
COOMe
Chemical Formula: C15H11Cl2NO4
Exact Mass: 339.0065
200
150
100
50
0 PPM
;blank linexql-2-29
USER: -- DATE: Mar 14 2013
F1: 100.509
F2: 399.673
SW1: 27778
OF1: 11300.1
PTS1d: 32768 , 32768
EX: s2pul
PW: 5.3 us
PD: 1.0 sec
NA: 256
LB: 2.0
Nuts - $_pslabel_02.fid

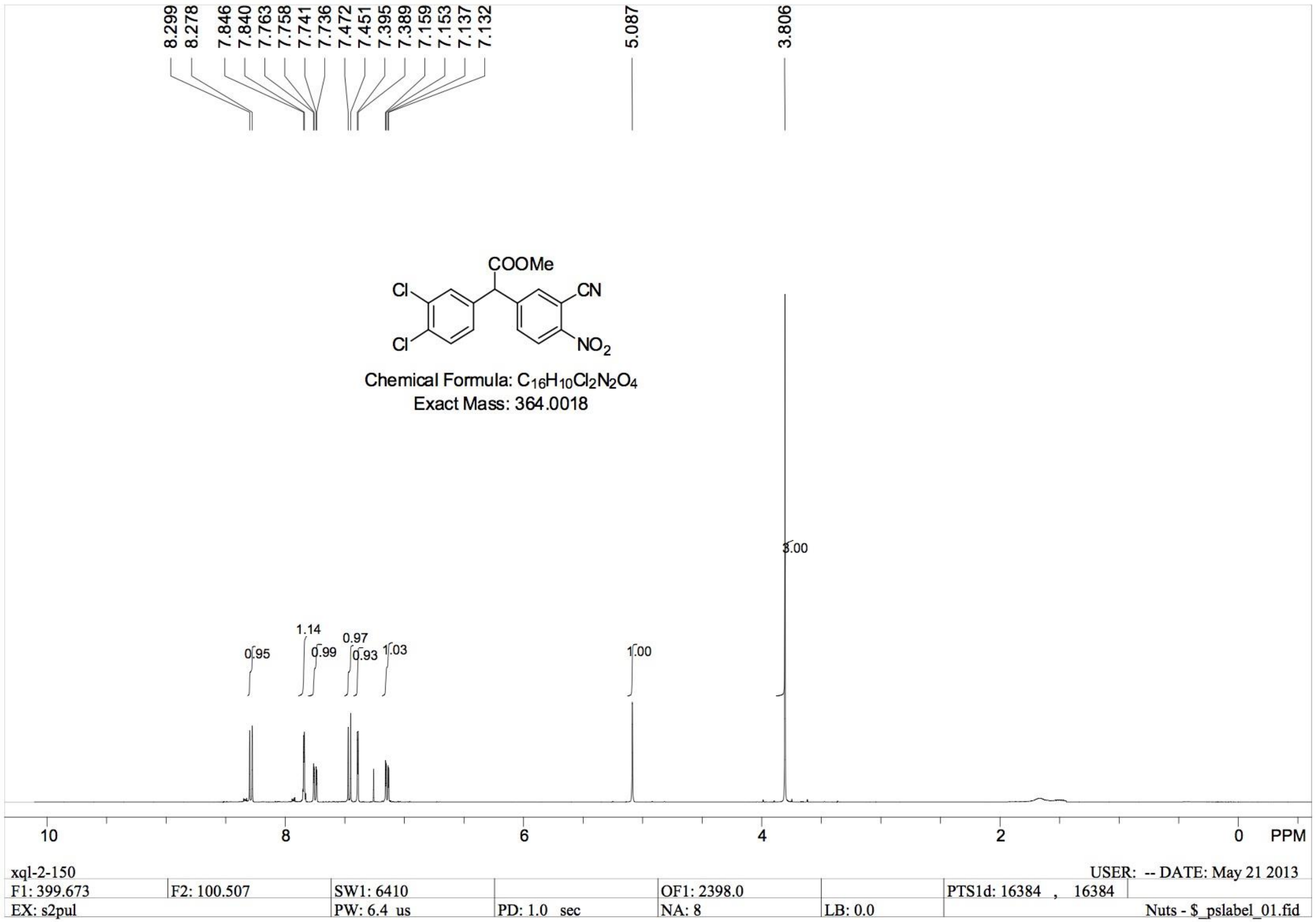
8.299
8.278
7.846
7.840
7.763
7.758
7.741
7.736
7.472
7.451
7.395
7.389
7.159
7.153
7.137
7.132
5.087
3.806
COOMe
Cl
Cl
CN
NO2
Chemical Formula: C16H10Cl2N2O4
Exact Mass: 364.0018
0.95
1.14
0.99
0.97
0.93
1.03
1.00
3.00
10
8
6
4
2
0
PPM
xql-2-150
USER: -- DATE: May 21 2013
F1: 399.673
F2: 100.507
SW1: 6410
OF1: 2398.0
PTS1d: 16384 , 16384
EX: s2pul
PW: 6.4 us
PD: 1.0 sec
NA: 8
LB: 0.0
Nuts - $_pslabel_01.fid

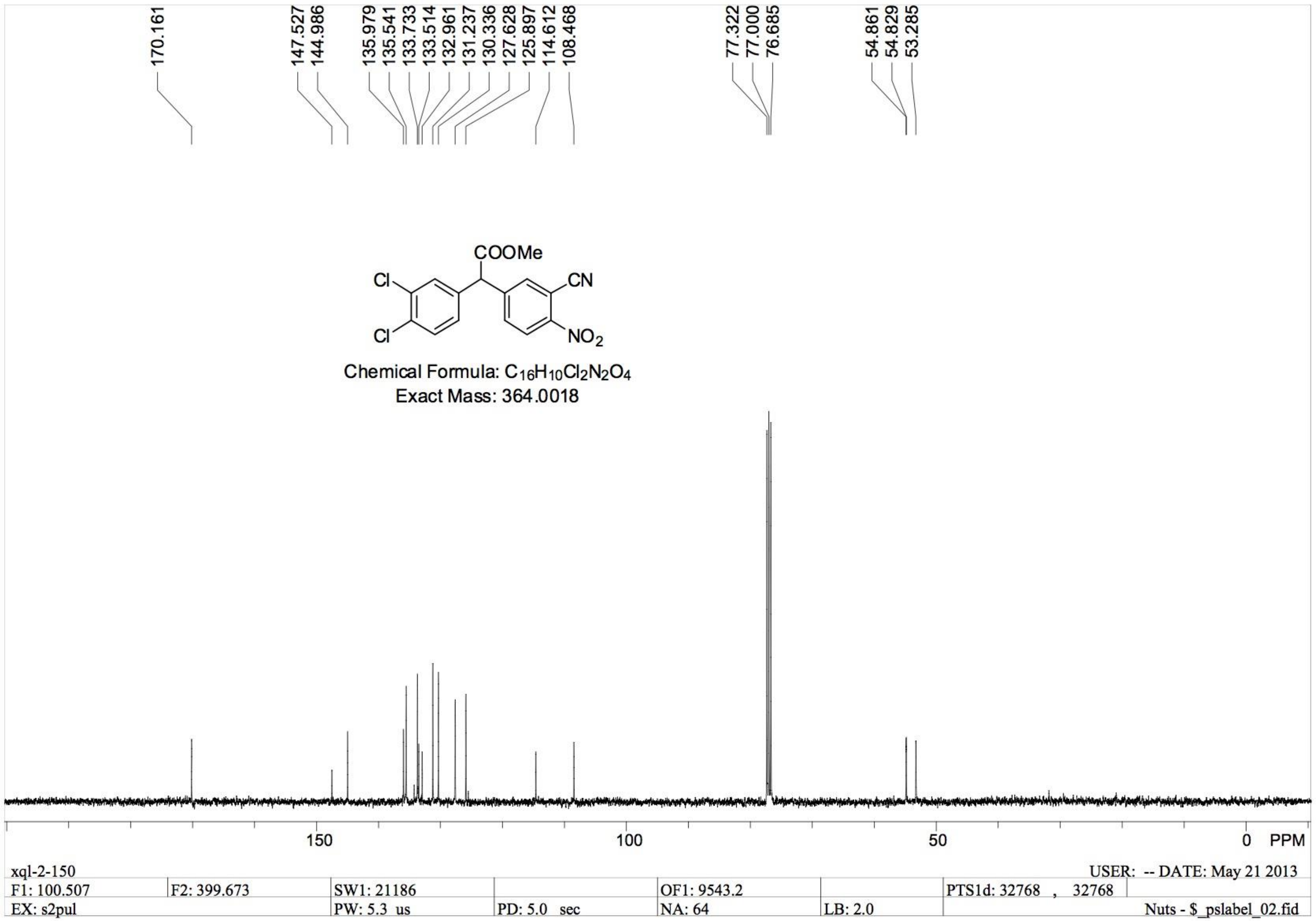
170.161
147.527
144.986
135.979
135.541
133.733
133.514
132.961
131.237
130.336
127.628
125.897
114.612
108.468
77.322
77.000
76.685
54.861
54.829
53.285
COOMe
Cl
CN
Cl
NO2
Chemical Formula: C16H10Cl2N2O4
Exact Mass: 364.0018
150
100
50
0 PPM
xql-2-150
USER: -- DATE: May 21 2013
F1: 100.507
F2: 399.673
SW1: 21186
OF1: 9543.2
PTS1d: 32768 , 32768
EX: s2pul
PW: 5.3 us
PD: 5.0 sec
NA: 64
LB: 2.0
Nuts - $_pslabel_02.fid

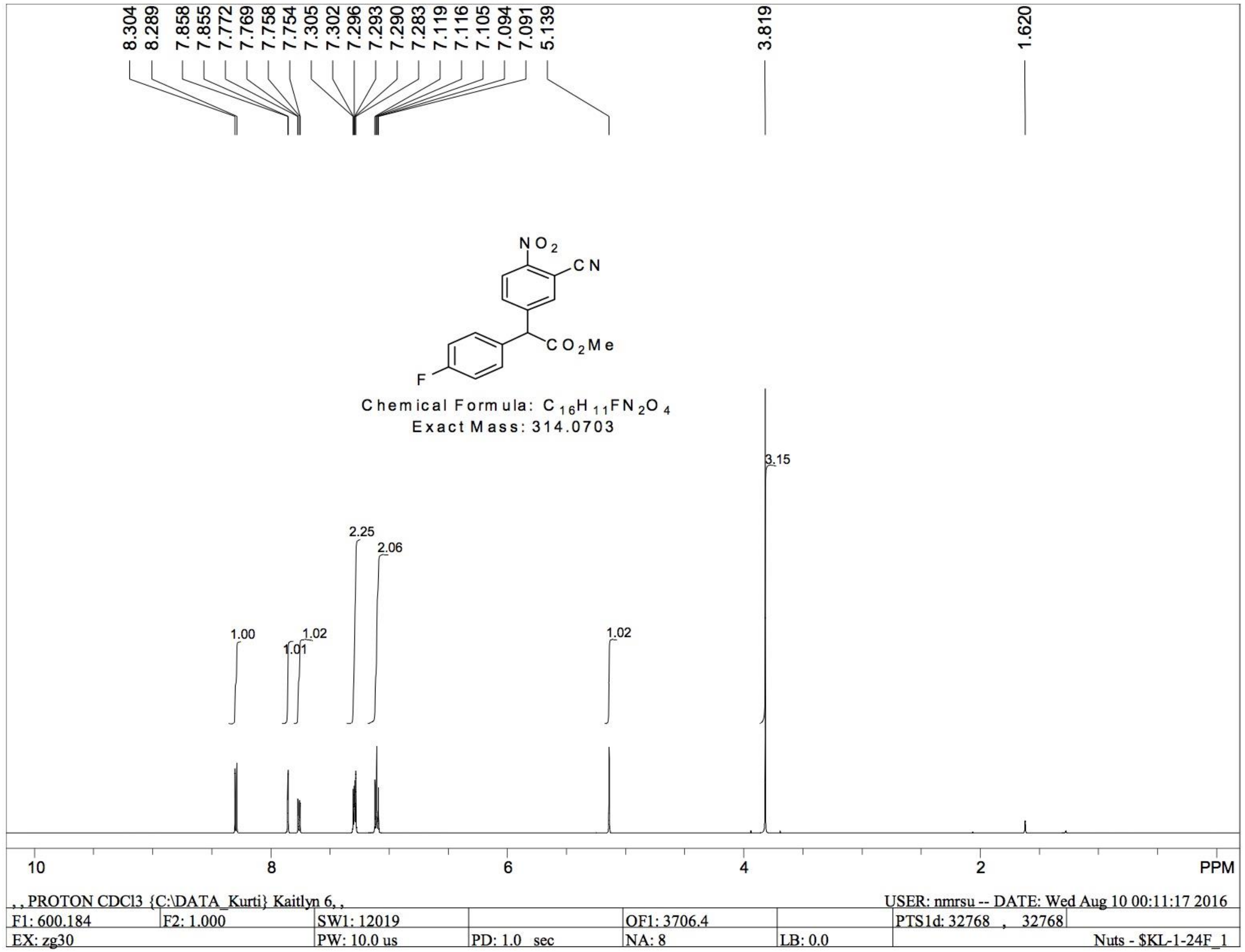
8.304
8.289
7.858
7.855
7.772
7.769
7.758
7.754
7.305
7.302
7.296
7.293
7.290
7.283
7.119
7.116
7.105
7.094
7.091
5.139
3.819
1.620
NO2
CN
CO2Me
F
Chemical Formula: C16H11FN2O4
Exact Mass: 314.0703
1.00
1.01
1.02
2.25
2.06
1.02
3.15
10
8
6
4
2
PPM
, , PROTON CDCl3 {C:\DATA_Kurti} Kaitlyn 6, ,
USER: nmrsu -- DATE: Wed Aug 10 00:11:17 2016
F1: 600.184
F2: 1.000
SW1: 12019
OF1: 3706.4
PTS1d: 32768 , 32768
EX: zg30
PW: 10.0 us
PD: 1.0 sec
NA: 8
LB: 0.0
Nuts - $KL-1-24F_1

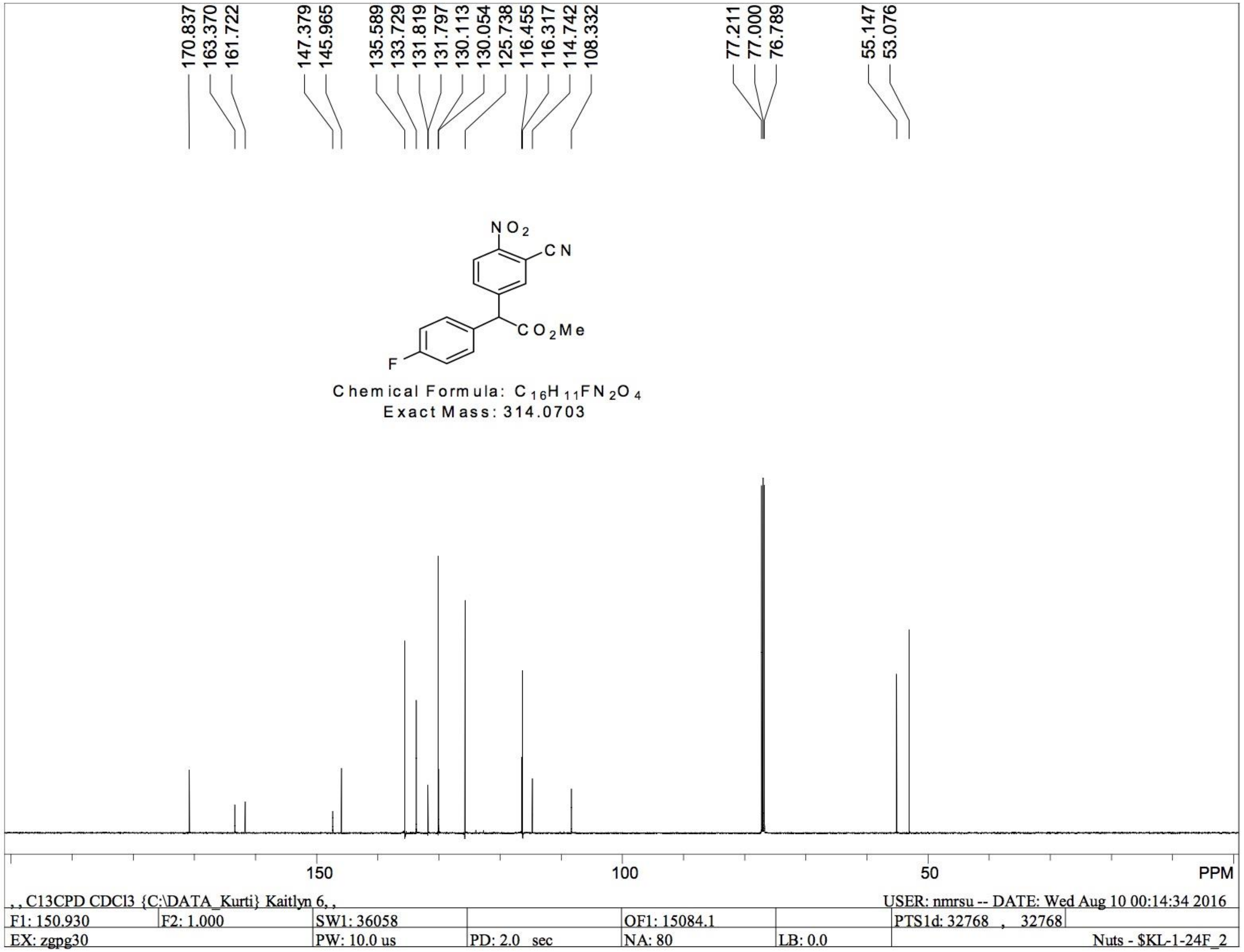
170.837
163.370
161.722
147.379
145.965
135.589
133.729
131.819
131.797
130.113
130.054
125.738
116.455
116.317
114.742
108.332
77.211
77.000
76.789
55.147
53.076
NO2
CN
CO2Me
F
Chemical Formula: C16H11FN2O4
Exact Mass: 314.0703
150
100
50
PPM
, , C13CPD CDCl3 {C:\DATA_Kurti} Kaitlyn 6, ,
USER: nmrsu -- DATE: Wed Aug 10 00:14:34 2016
F1: 150.930
F2: 1.000
SW1: 36058
OF1: 15084.1
PTS1d: 32768 , 32768
EX: zgpg30
PW: 10.0 us
PD: 2.0 sec
NA: 80
LB: 0.0
Nuts - $KL-1-24F_2

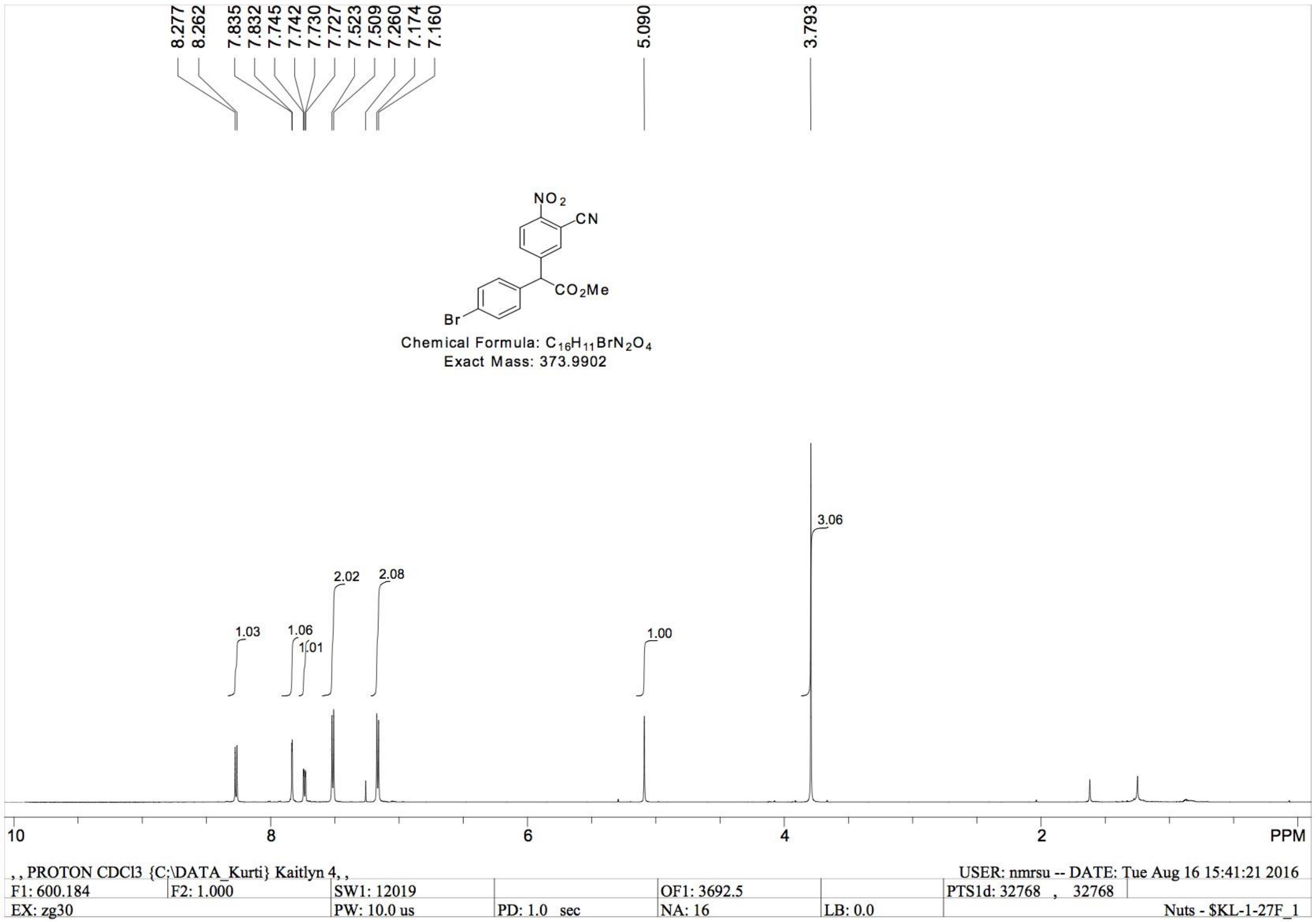
8.277
8.262
7.835
7.832
7.745
7.742
7.730
7.727
7.523
7.509
7.260
7.174
7.160
5.090
3.793
NO2
CN
CO2Me
Br
Chemical Formula: C16H11BrN2O4
Exact Mass: 373.9902
1.03
1.06
1.01
2.02
2.08
1.00
3.06
10
8
6
4
2
PPM
, , PROTON CDCl3 {C:\DATA_Kurti} Kaitlyn 4, ,
USER: nmrsu -- DATE: Tue Aug 16 15:41:21 2016
F1: 600.184
F2: 1.000
SW1: 12019
OF1: 3692.5
PTS1d: 32768 , 32768
EX: zg30
PW: 10.0 us
PD: 1.0 sec
NA: 16
LB: 0.0
Nuts - $KL-1-27F_1

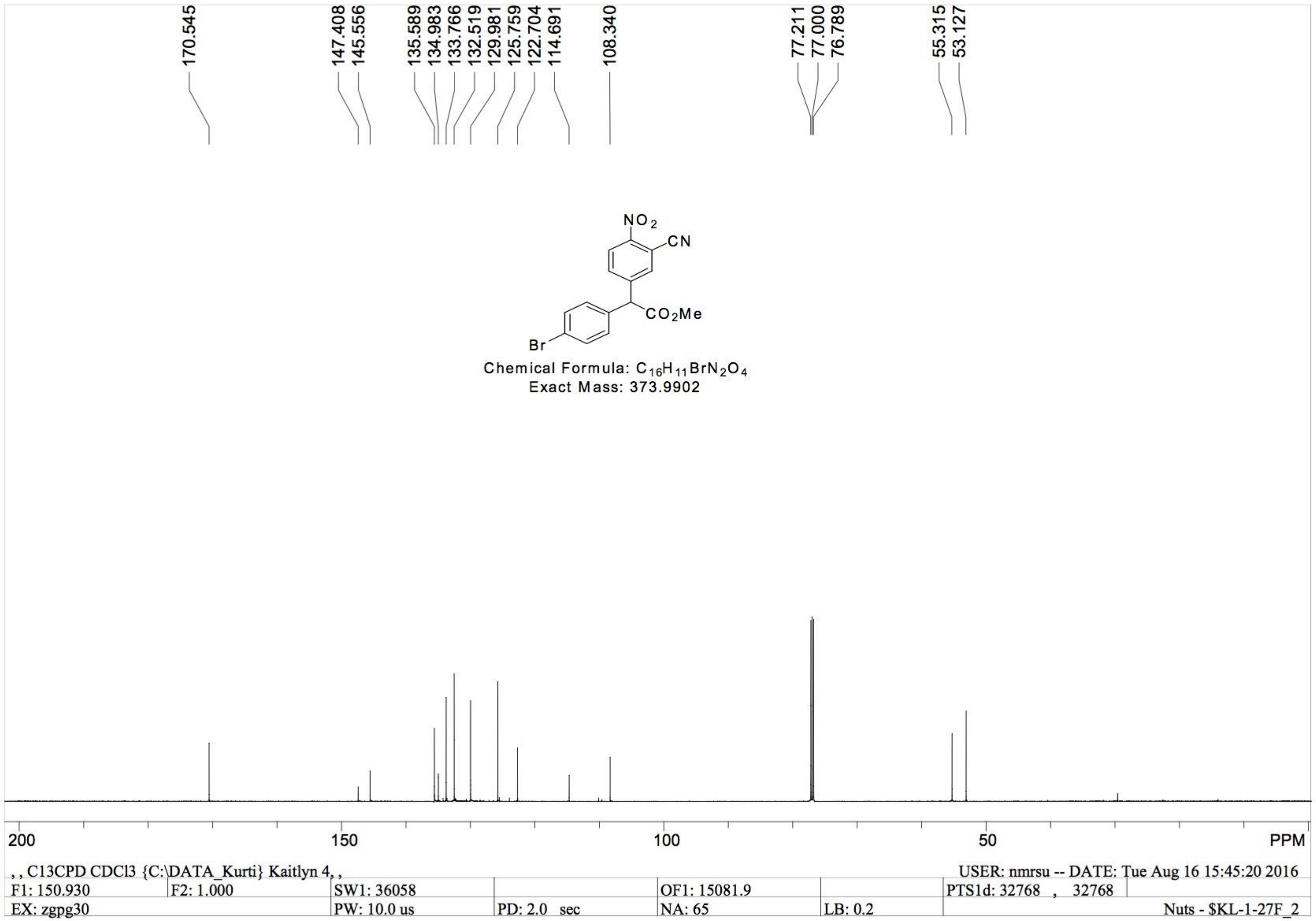
170.545
147.408
145.556
135.589
134.983
133.766
132.519
129.981
125.759
122.704
114.691
108.340
77.211
77.000
76.789
55.315
53.127
NO2
CN
CO2Me
Br
Chemical Formula: C16H11BrN2O4
Exact Mass: 373.9902
200
150
100
50
PPM
, , C13CPD CDCl3 {C:\DATA_Kurti} Kaitlyn 4, ,
USER: nmrsu -- DATE: Tue Aug 16 15:45:20 2016
F1: 150.930
F2: 1.000
SW1: 36058
OF1: 15081.9
PTS1d: 32768 , 32768
EX: zgpg30
PW: 10.0 us
PD: 2.0 sec
NA: 65
LB: 0.2
Nuts - $KL-1-27F_2

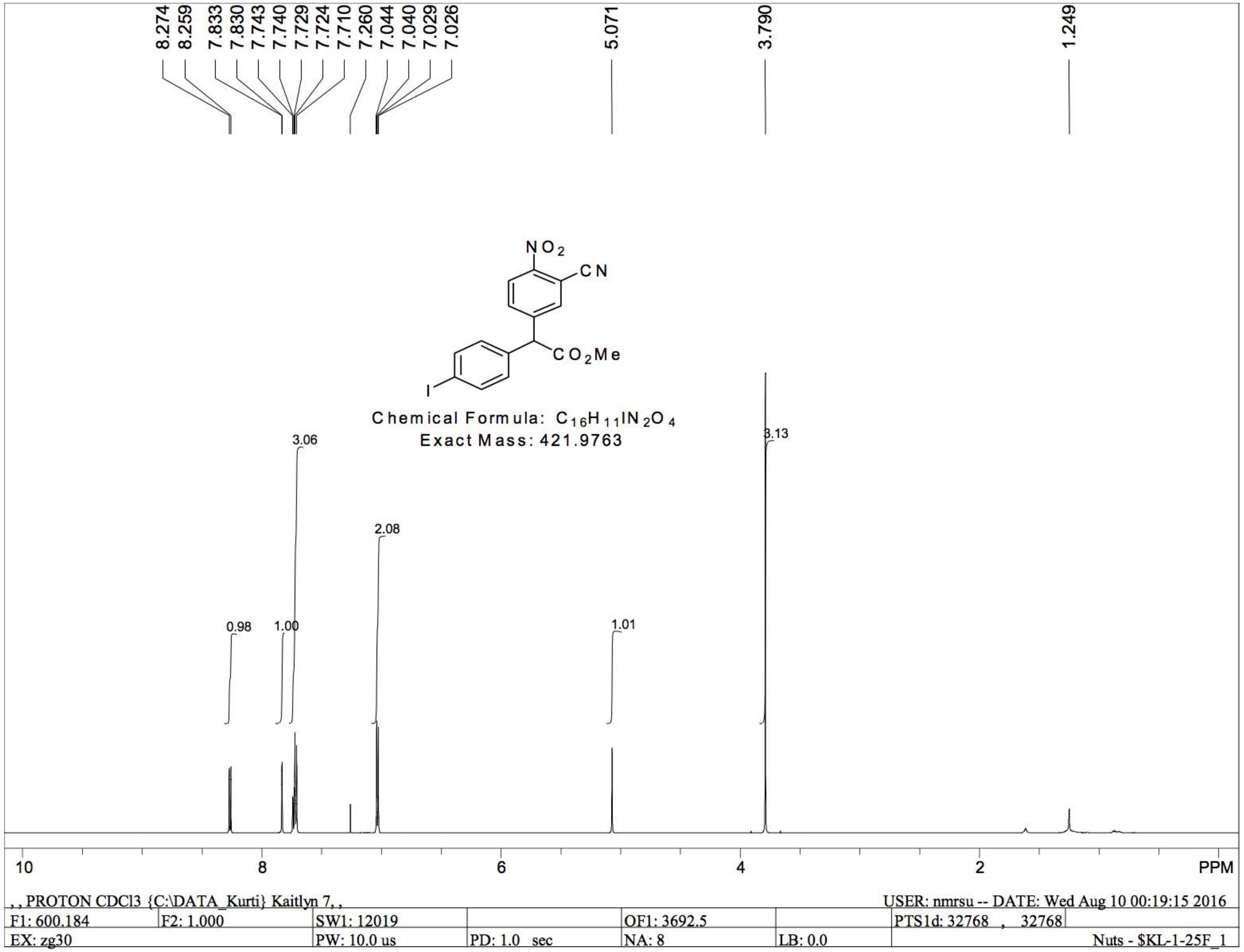
8.274
8.259
7.833
7.830
7.743
7.740
7.729
7.724
7.710
7.260
7.044
7.040
7.029
7.026
5.071
3.790
1.249
NO2
CN
CO2Me
I
Chemical Formula: C16H11IN2O4
Exact Mass: 421.9763
3.06
3.13
2.08
0.98
1.00
1.01
10
8
6
4
2
PPM
, , PROTON CDCl3 {C:\DATA_Kurti} Kaitlyn 7, ,
USER: nmrsu -- DATE: Wed Aug 10 00:19:15 2016
F1: 600.184
F2: 1.000
SW1: 12019
OF1: 3692.5
PTS1d: 32768 , 32768
EX: zg30
PW: 10.0 us
PD: 1.0 sec
NA: 8
LB: 0.0
Nuts - $KL-1-25F_1

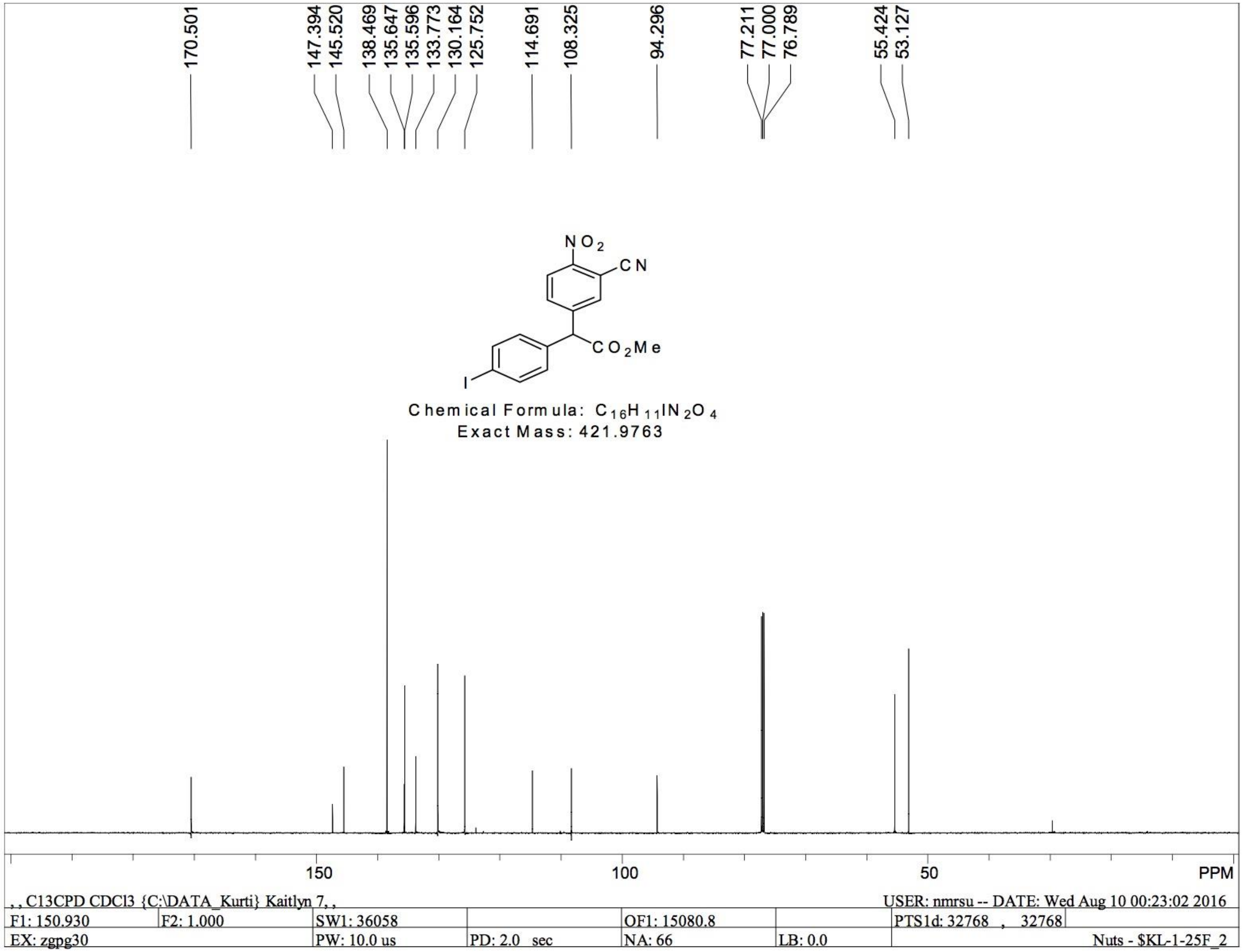
170.501
147.394
145.520
138.469
135.647
135.596
133.773
130.164
125.752
114.691
108.325
94.296
77.211
77.000
76.789
55.424
53.127
NO2
CN
CO2Me
I
Chemical Formula: C16H11IN2O4
Exact Mass: 421.9763
150
100
50
PPM
, , C13CPD CDCl3 {C:\DATA_Kurti} Kaitlyn 7, ,
USER: nmrsu -- DATE: Wed Aug 10 00:23:02 2016
F1: 150.930
F2: 1.000
SW1: 36058
OF1: 15080.8
PTS1d: 32768 , 32768
EX: zgpg30
PW: 10.0 us
PD: 2.0 sec
NA: 66
LB: 0.0
Nuts - $KL-1-25F_2

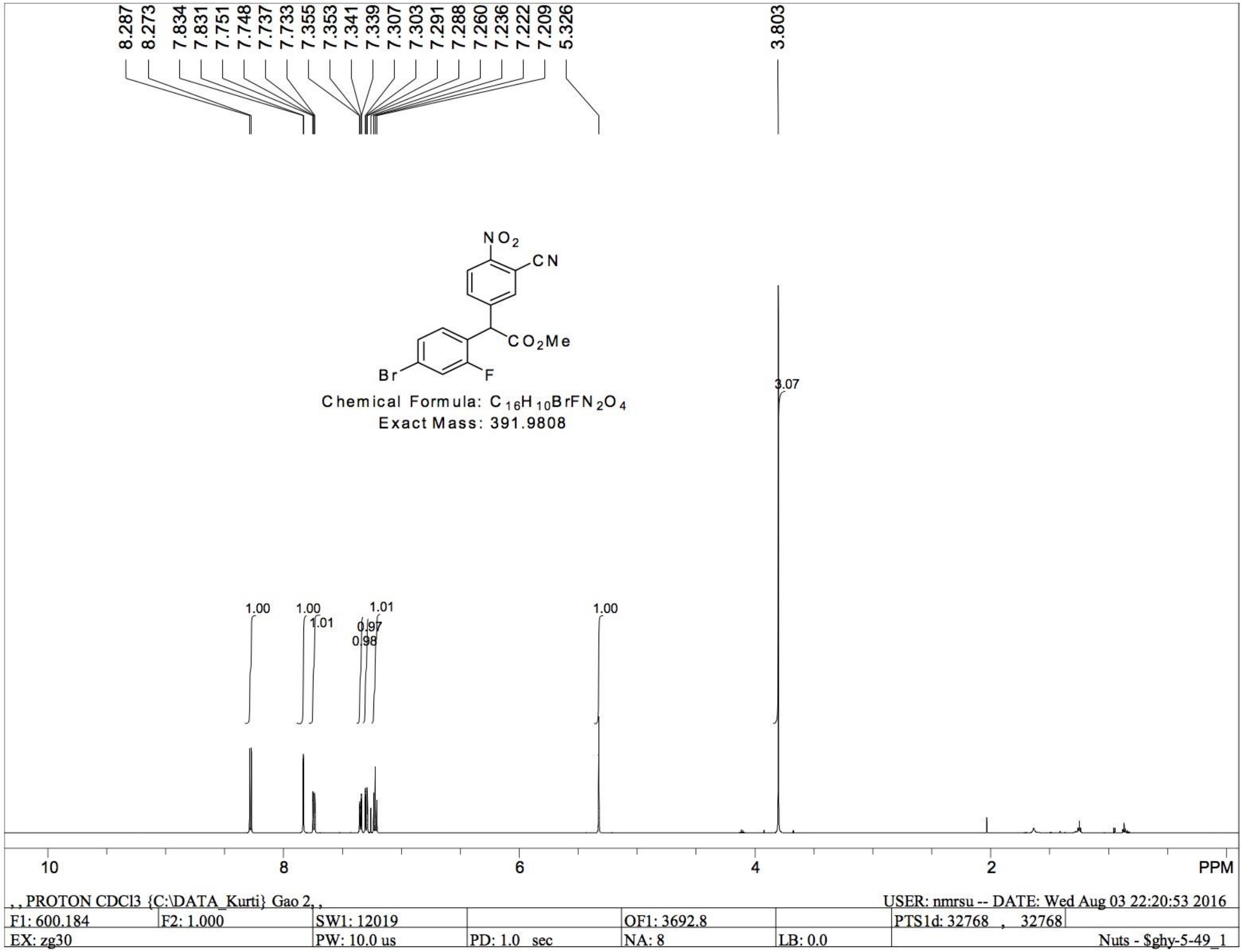
8.287 8.273 7.834 7.831 7.751 7.748 7.737 7.733 7.355 7.353 7.341 7.339 7.307 7.303 7.291 7.288 7.260 7.236 7.222 7.209 5.326
3.803
NO2
CN
CO2Me
Br
F
Chemical Formula: C16H10BrFN2O4
Exact Mass: 391.9808
3.07
1.00
1.00
1.01
0.97
0.98
1.01
1.00
10
8
6
4
2
PPM
, , PROTON CDCl3 {C:\DATA_Kurti} Gao 2, ,
USER: nmrsu -- DATE: Wed Aug 03 22:20:53 2016
F1: 600.184
F2: 1.000
SW1: 12019
OF1: 3692.8
PTS1d: 32768 , 32768
EX: zg30
PW: 10.0 us
PD: 1.0 sec
NA: 8
LB: 0.0
Nuts - $ghy-5-49_1

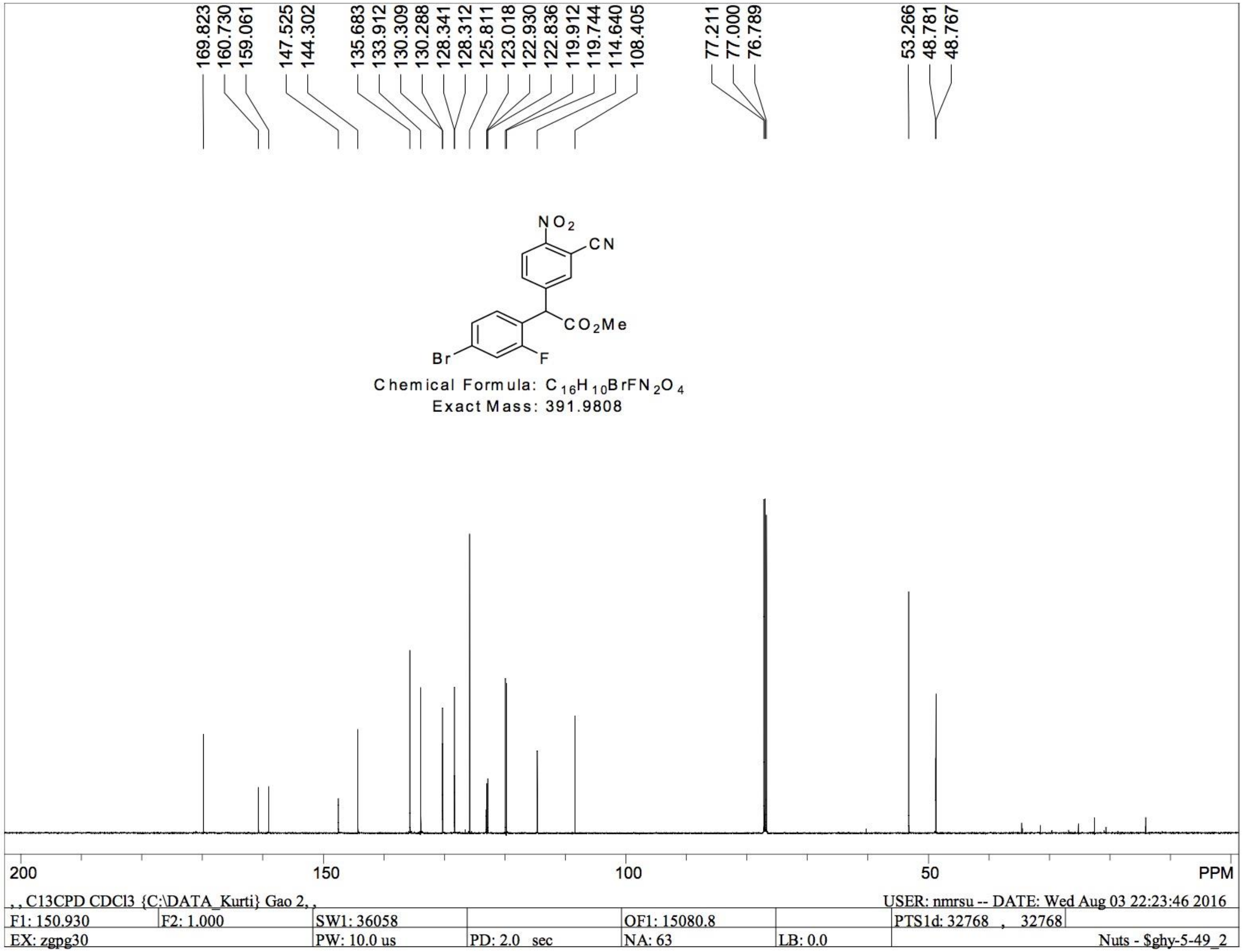
169.823
160.730
159.061
147.525
144.302
135.683
133.912
130.309
130.288
128.341
128.312
125.811
123.018
122.930
122.836
119.912
119.744
114.640
108.405
77.211
77.000
76.789
53.266
48.781
48.767
NO2
CN
CO2Me
Br
F
Chemical Formula: C16H10BrFN2O4
Exact Mass: 391.9808
200
150
100
50
PPM
, , C13CPD CDCl3 {C:\DATA_Kurti} Gao 2, ,
USER: nmrsu -- DATE: Wed Aug 03 22:23:46 2016
F1: 150.930
F2: 1.000
SW1: 36058
OF1: 15080.8
PTS1d: 32768 , 32768
EX: zgpg30
PW: 10.0 us
PD: 2.0 sec
NA: 63
LB: 0.0
Nuts - $ghy-5-49_2

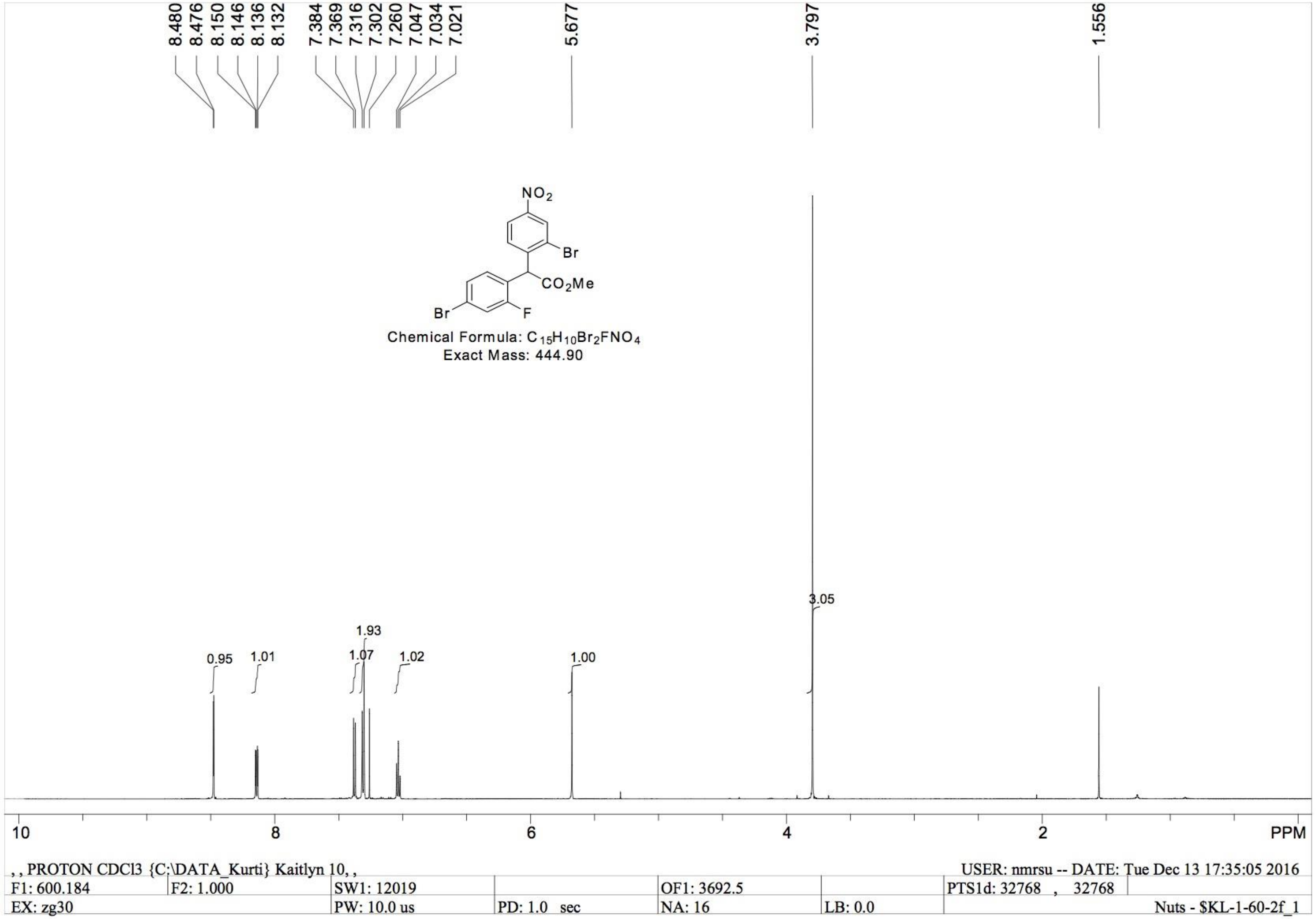
8.480
8.476
8.150
8.146
8.136
8.132
7.384
7.369
7.316
7.302
7.260
7.047
7.034
7.021
5.677
3.797
1.556
NO2
Br
CO2Me
Br
F
Chemical Formula: C15H10Br2FNO4
Exact Mass: 444.90
0.95
1.01
1.93
1.07
1.02
1.00
3.05
10
8
6
4
2
PPM
, , PROTON CDCl3 {C:\DATA_Kurti} Kaitlyn 10, ,
USER: nmrsu -- DATE: Tue Dec 13 17:35:05 2016
F1: 600.184
F2: 1.000
SW1: 12019
OF1: 3692.5
PTS1d: 32768 , 32768
EX: zg30
PW: 10.0 us
PD: 1.0 sec
NA: 16
LB: 0.0
Nuts - $KL-1-60-2f_1

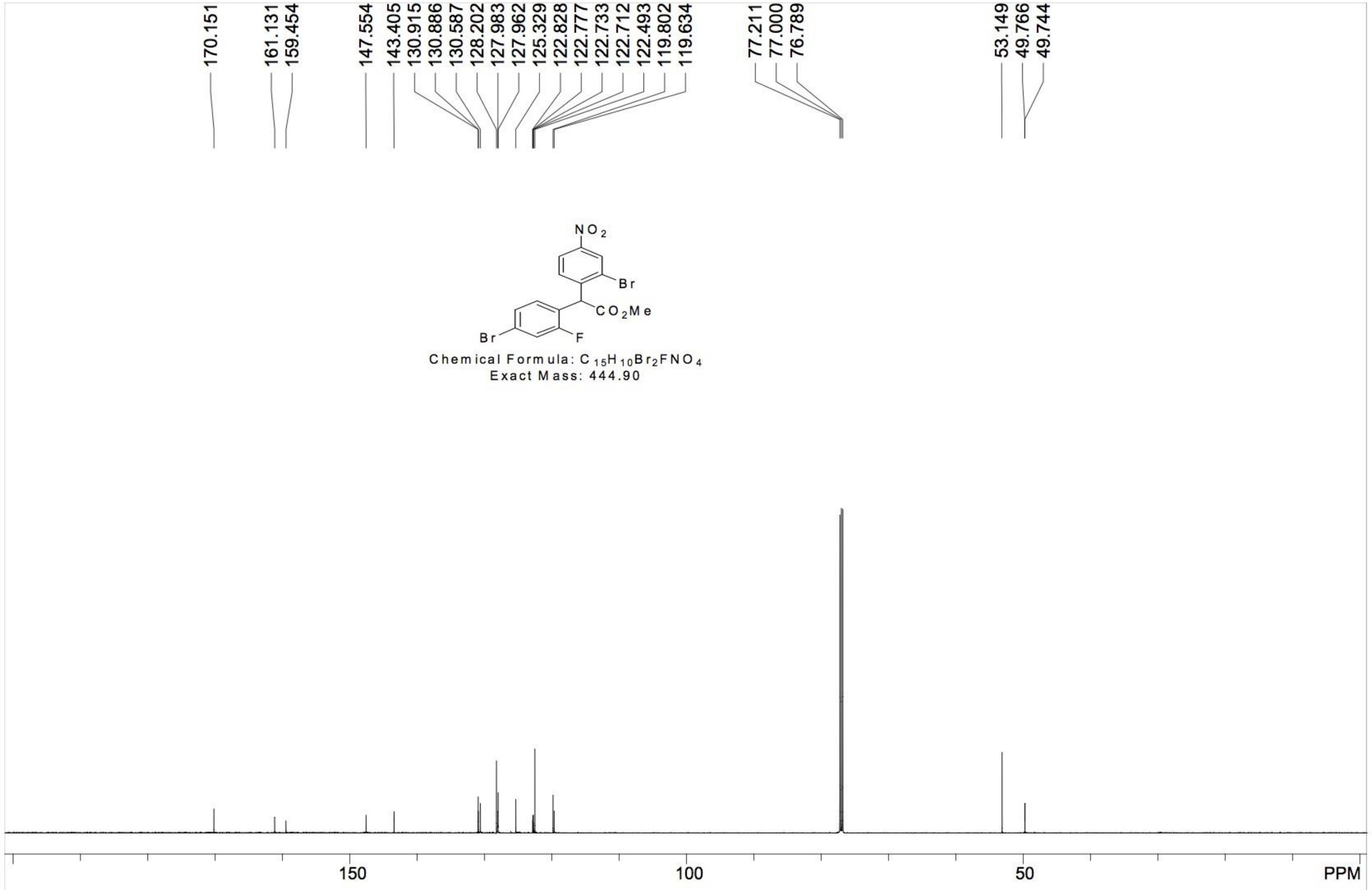

, , C13CPD CDCl3 {C:\DATA_Kurti} Kaitlyn 21, , USER: nmrsu -- DATE: Tue Dec 06 19:52:01 2016

F1: 150.930	F2: 1.000	SW1: 36058		OF1: 15087.4		PTS1d: 32768 , 32768	
EX: zgpg30		PW: 10.0 us	PD: 2.0 sec	NA: 100	LB: 0.2		Nuts - $KL-1-60-2f_2

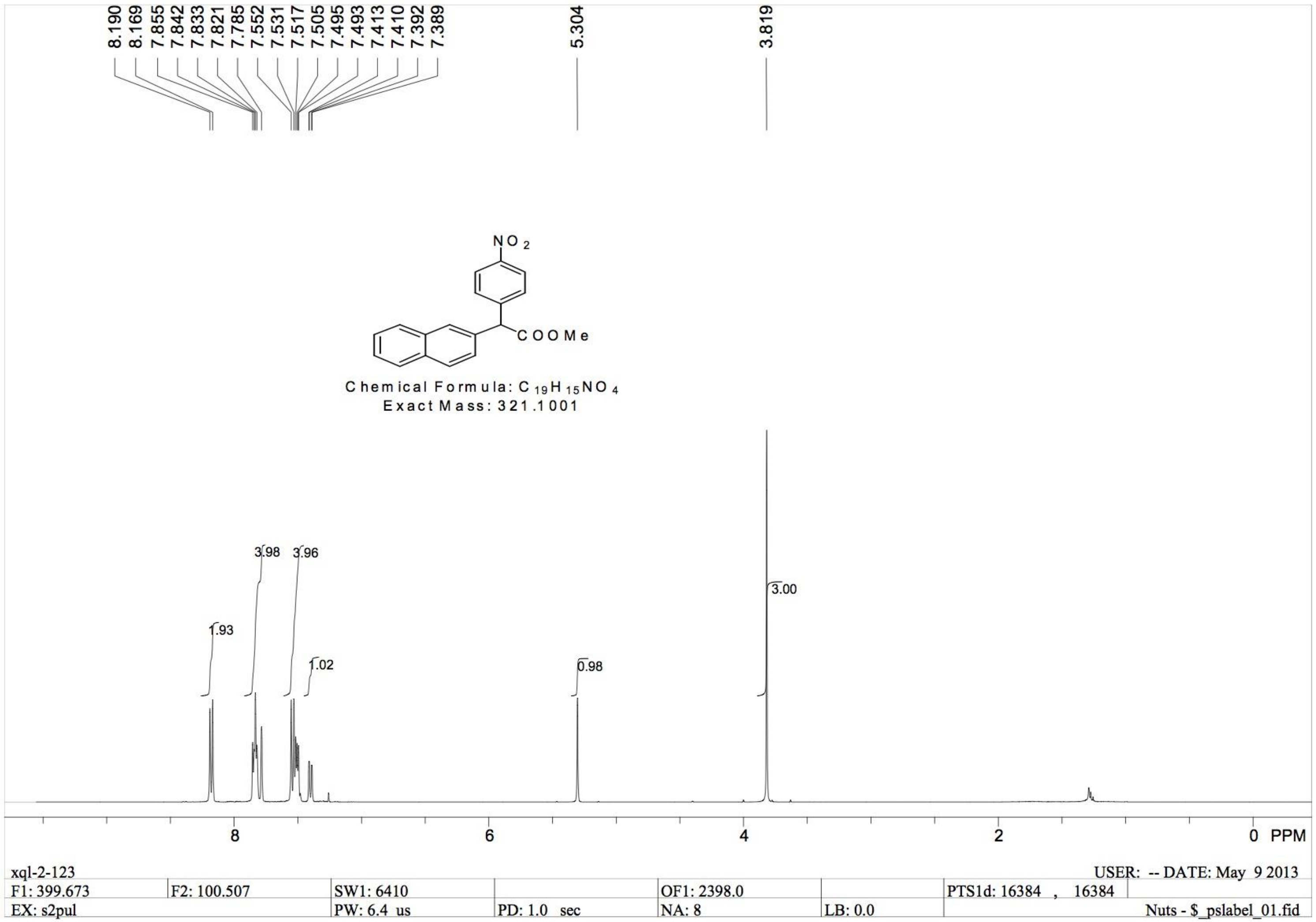
8.190
8.169
7.855
7.842
7.833
7.821
7.785
7.552
7.531
7.517
7.505
7.495
7.493
7.413
7.410
7.392
7.389
5.304
3.819
NO2
COOMe
Chemical Formula: C19H15NO4
Exact Mass: 321.1001
1.93
3.98
3.96
1.02
0.98
3.00
8
6
4
2
0 PPM
xql-2-123
USER: -- DATE: May 9 2013
F1: 399.673
F2: 100.507
SW1: 6410
OF1: 2398.0
PTS1d: 16384 , 16384
EX: s2pul
PW: 6.4 us
PD: 1.0 sec
NA: 8
LB: 0.0
Nuts - $_pslabel_01.fid

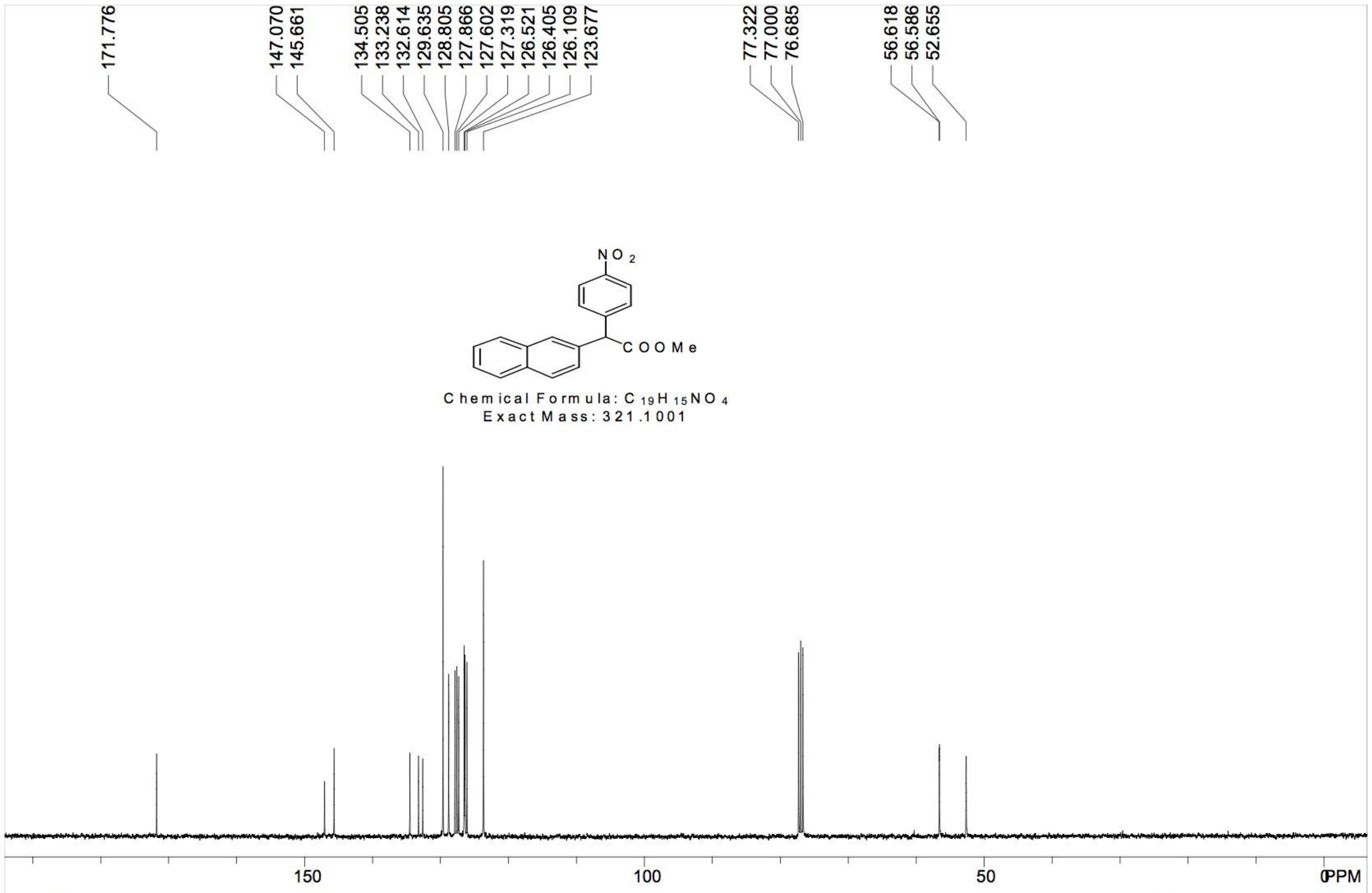

xql-2-123 USER: -- DATE: May 9 2013

F1: 100.507	F2: 399.673	SW1: 21186		OF1: 9536.7		PTS1d: 32768 , 32768	
EX: s2pul		PW: 5.3 us	PD: 3.0 sec	NA: 64	LB: 2.0		Nuts - $_pslabel_02.fid

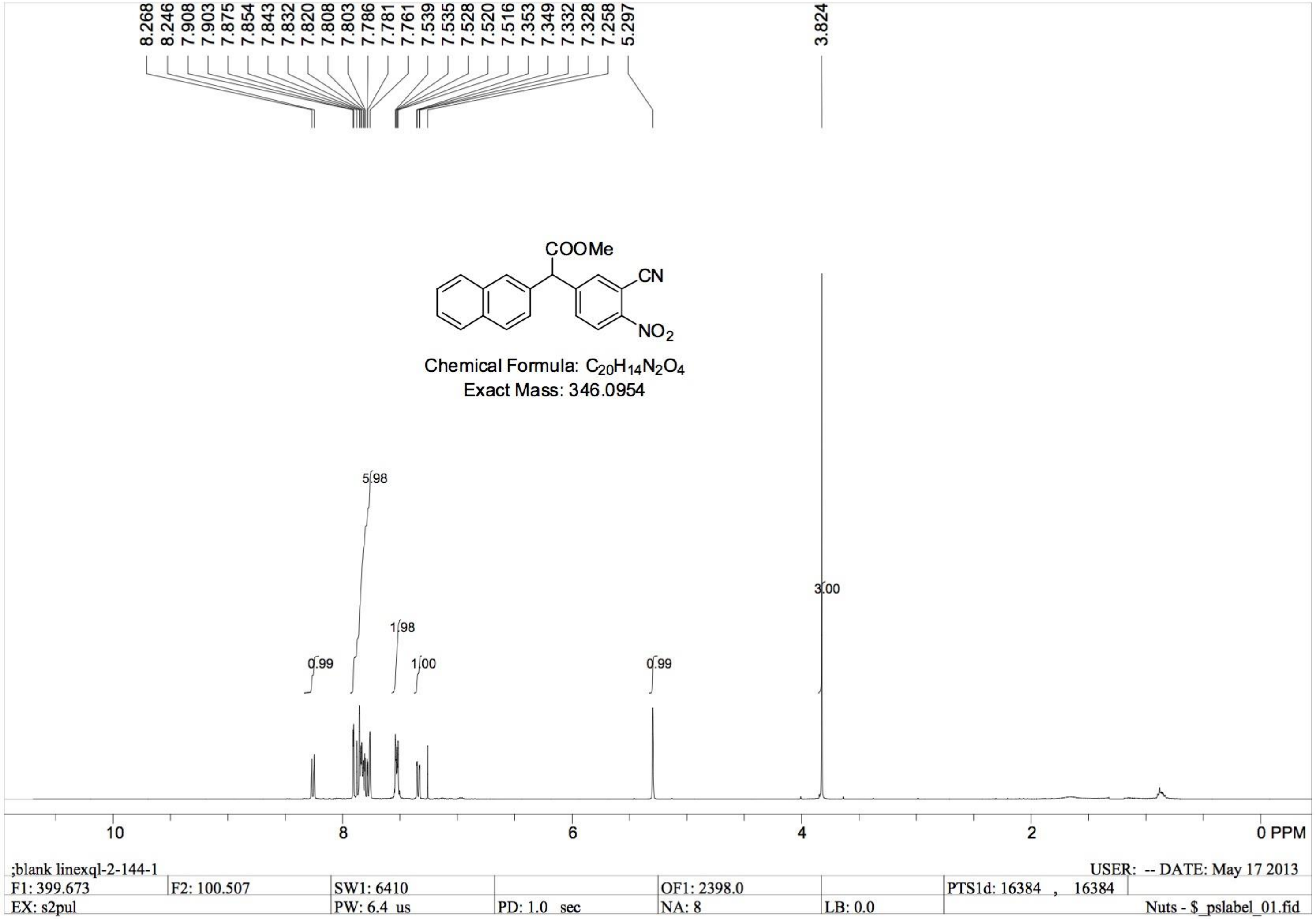
8.268
8.246
7.908
7.903
7.875
7.854
7.843
7.832
7.820
7.808
7.803
7.786
7.781
7.761
7.539
7.535
7.528
7.520
7.516
7.353
7.349
7.332
7.328
7.258
5.297
3.824
COOMe
CN
NO2
Chemical Formula: C20H14N2O4
Exact Mass: 346.0954
5.98
3.00
1.98
0.99
1.00
0.99
10
8
6
4
2
0 PPM
;blank linexql-2-144-1
USER: -- DATE: May 17 2013
F1: 399.673
F2: 100.507
SW1: 6410
OF1: 2398.0
PTS1d: 16384 , 16384
EX: s2pul
PW: 6.4 us
PD: 1.0 sec
NA: 8
LB: 0.0
Nuts - $_pslabel_01.fid

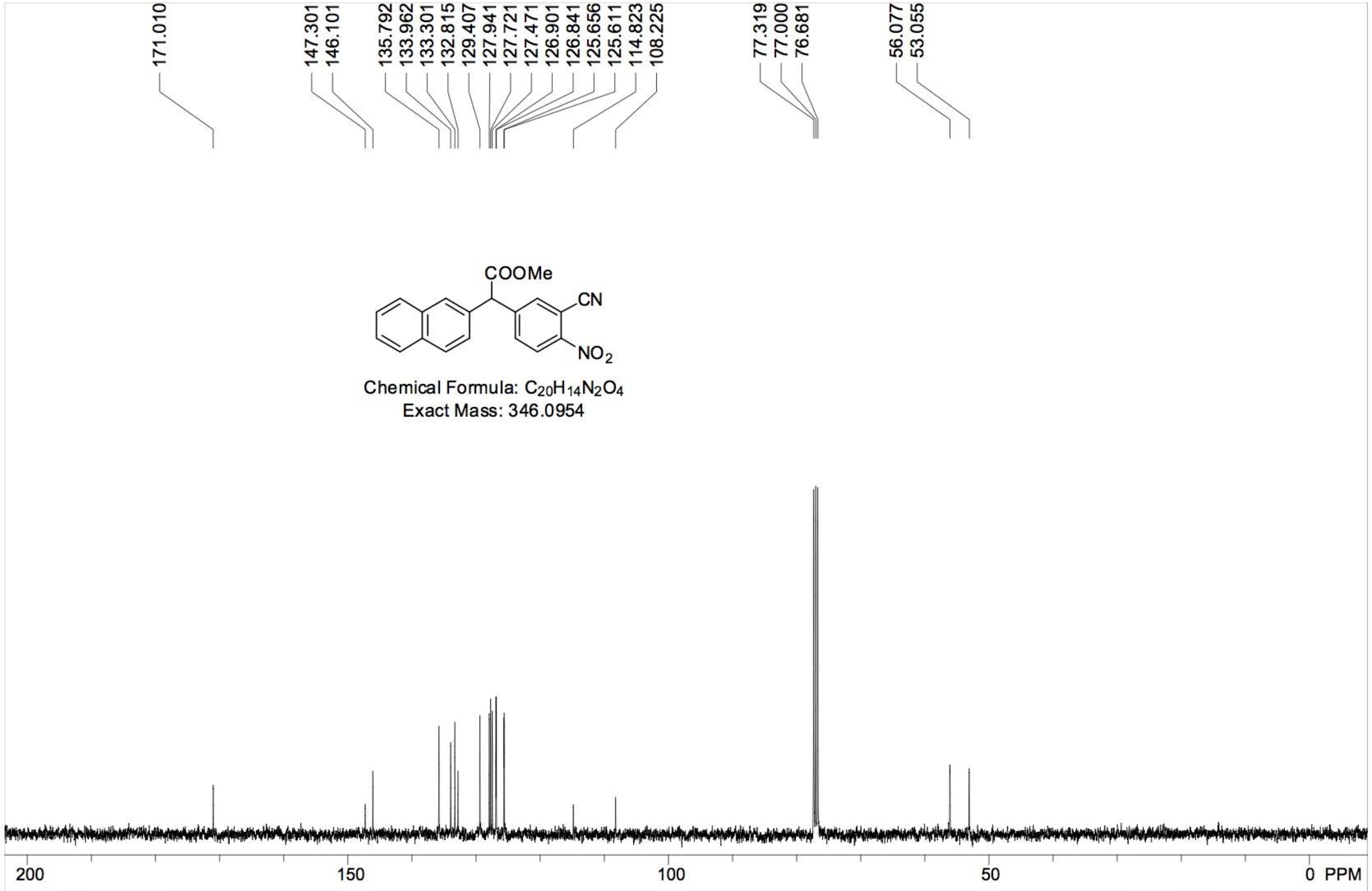

;blank linexql-2-144-1 USER: -- DATE: May 17 2013

F1: 100.508	F2: 399.673	SW1: 25000		OF1: 11051.5		PTS1d: 32768 , 32768	
EX: s2pul		PW: 5.3 us	PD: 5.0 sec	NA: 64	LB: 2.0		Nuts - $_pslabel_02.fid

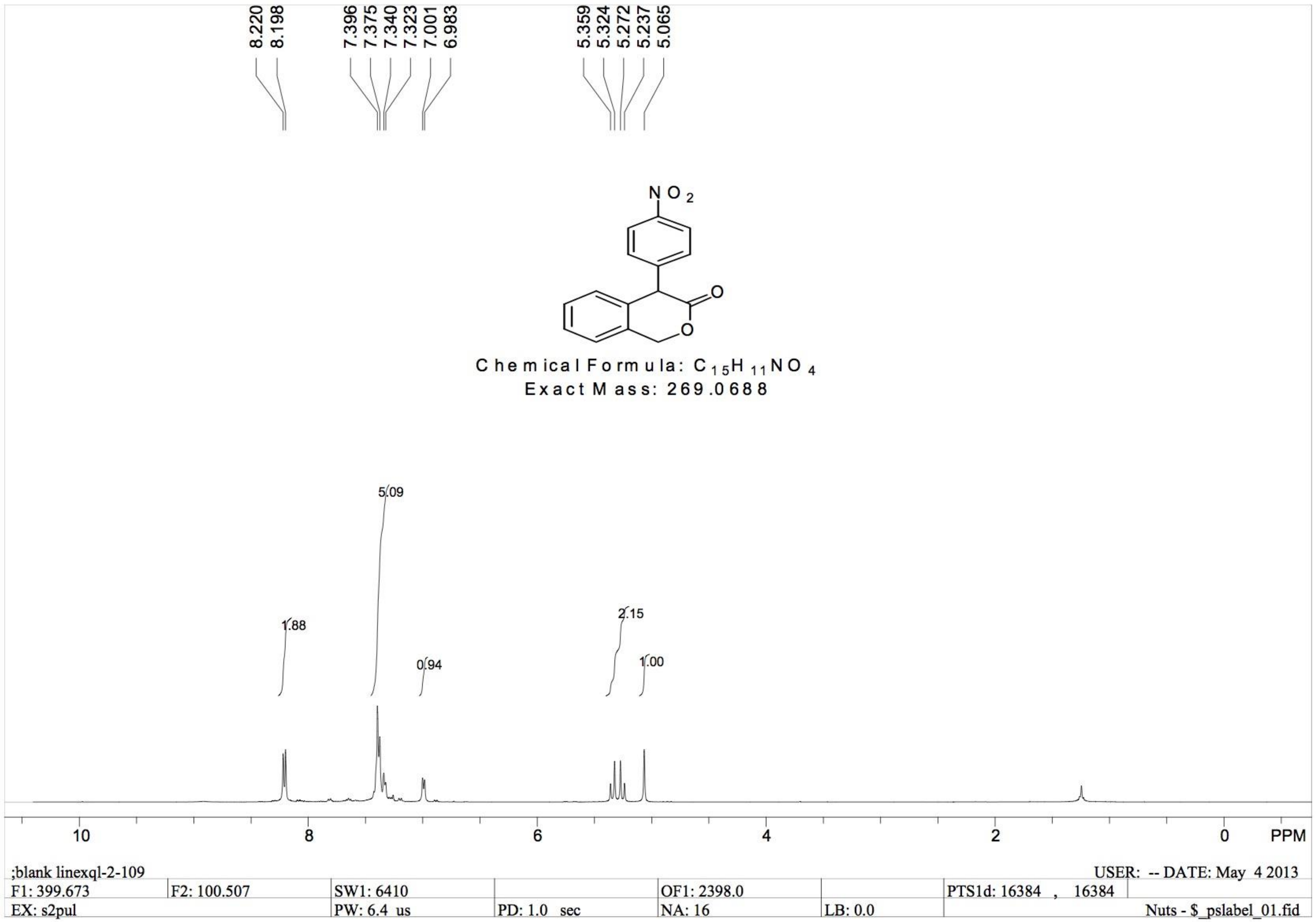
8.220
8.198
7.396
7.375
7.340
7.323
7.001
6.983
5.359
5.324
5.272
5.237
5.065
NO2
O
O
Chemical Formula: C15H11NO4
Exact Mass: 269.0688
5.09
1.88
0.94
2.15
1.00
10
8
6
4
2
0
PPM
;blank linexql-2-109
USER: -- DATE: May 4 2013
F1: 399.673
F2: 100.507
SW1: 6410
OF1: 2398.0
PTS1d: 16384 , 16384
EX: s2pul
PW: 6.4 us
PD: 1.0 sec
NA: 16
LB: 0.0
Nuts - $_pslabel_01.fid

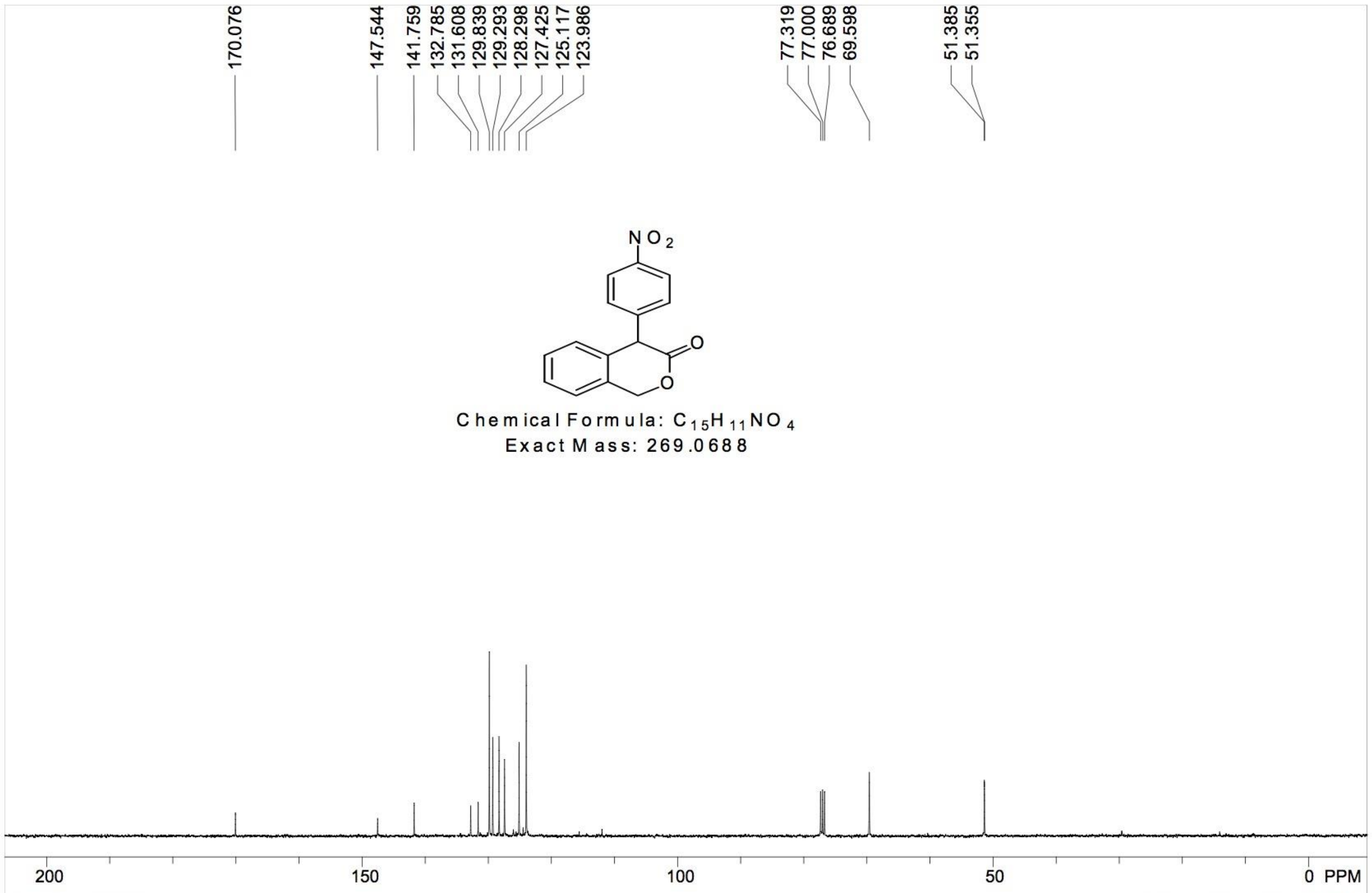

;blank linexql-2-109						USER: -- DATE: May 4 2013
F1: 100.508	F2: 399.673	SW1: 25000		OF1: 11048.4		PTS1d: 32768 , 32768
EX: s2pul		PW: 5.3 us	PD: 1.0 sec	NA: 256	LB: 2.0	Nuts - $_pslabel_02.fid

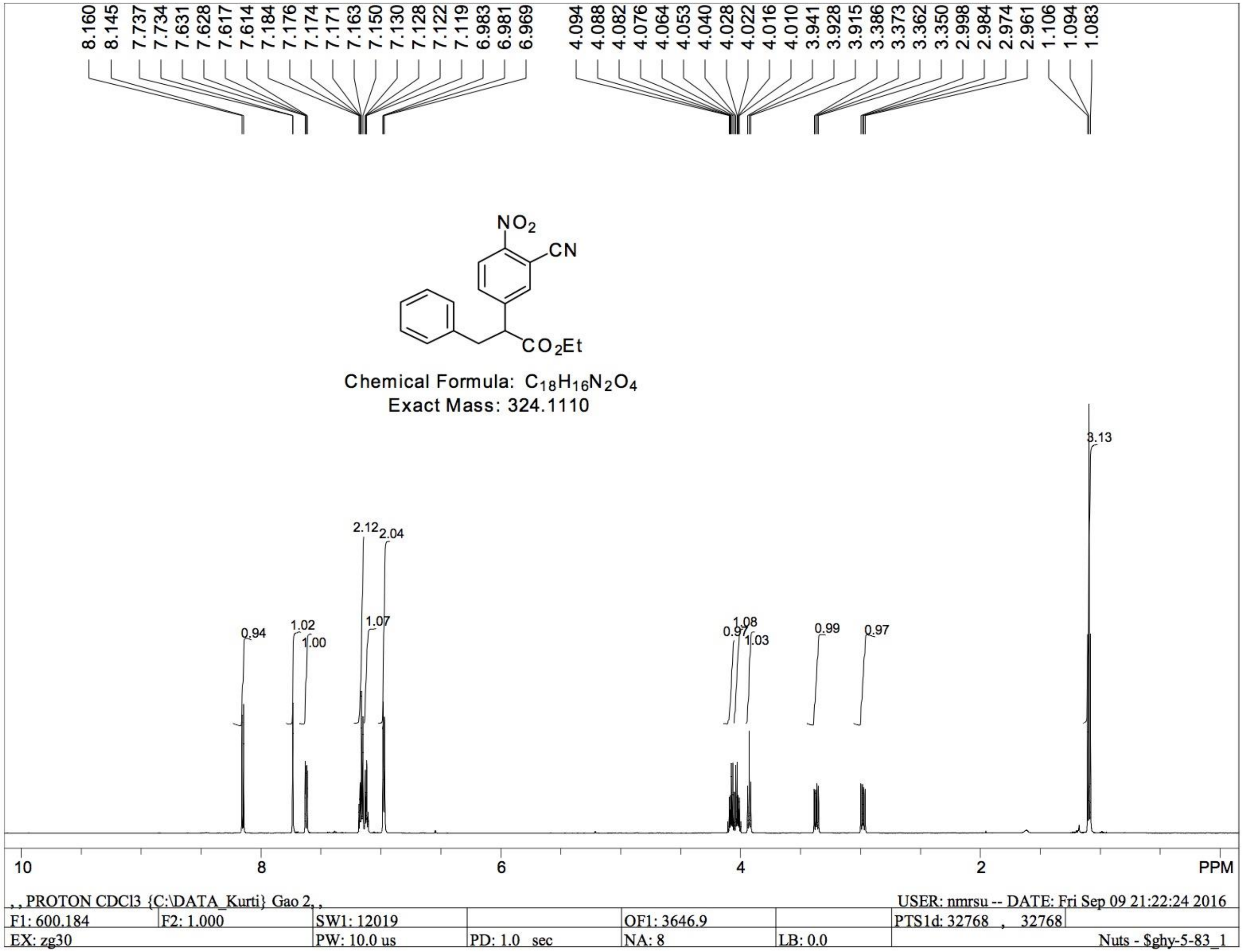
8.160 8.145 7.737 7.734 7.631 7.628 7.617 7.614 7.184 7.176 7.174 7.171 7.163 7.150 7.130 7.128 7.122 7.119 6.983 6.981 6.969
4.094 4.088 4.082 4.076 4.064 4.053 4.040 4.028 4.022 4.016 4.010 3.941 3.928 3.915 3.386 3.373 3.362 3.350 2.998 2.984 2.974 2.961 1.106 1.094 1.083
NO2
CN
CO2Et
Chemical Formula: C18H16N2O4
Exact Mass: 324.1110
0.94
1.02
1.00
2.12
2.04
1.07
0.97
1.08
1.03
0.99
0.97
3.13
10
8
6
4
2
PPM
, , PROTON CDCl3 {C:\DATA_Kurti} Gao 2, ,
USER: nmrsu -- DATE: Fri Sep 09 21:22:24 2016
F1: 600.184
F2: 1.000
SW1: 12019
OF1: 3646.9
PTS1d: 32768 , 32768
EX: zg30
PW: 10.0 us
PD: 1.0 sec
NA: 8
LB: 0.0
Nuts - $ghy-5-83_1

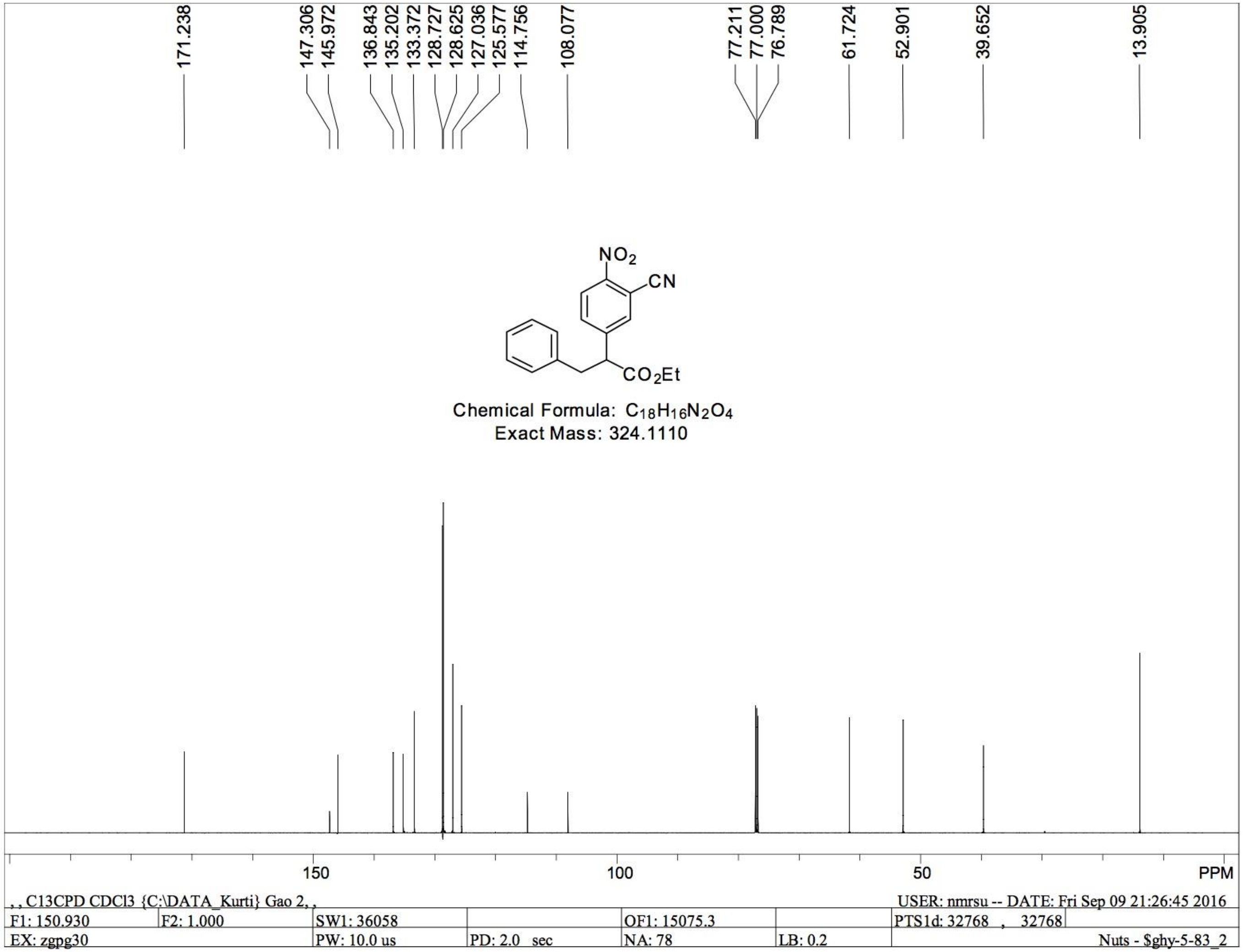
171.238
147.306
145.972
136.843
135.202
133.372
128.727
128.625
127.036
125.577
114.756
108.077
77.211
77.000
76.789
61.724
52.901
39.652
13.905
NO2
CN
CO2Et
Chemical Formula: C18H16N2O4
Exact Mass: 324.1110
150
100
50
PPM
, , C13CPD CDCl3 {C:\DATA_Kurti} Gao 2, ,
USER: nmrsu -- DATE: Fri Sep 09 21:26:45 2016
F1: 150.930
F2: 1.000
SW1: 36058
OF1: 15075.3
PTS1d: 32768 , 32768
EX: zgpg30
PW: 10.0 us
PD: 2.0 sec
NA: 78
LB: 0.2
Nuts - $ghy-5-83_2

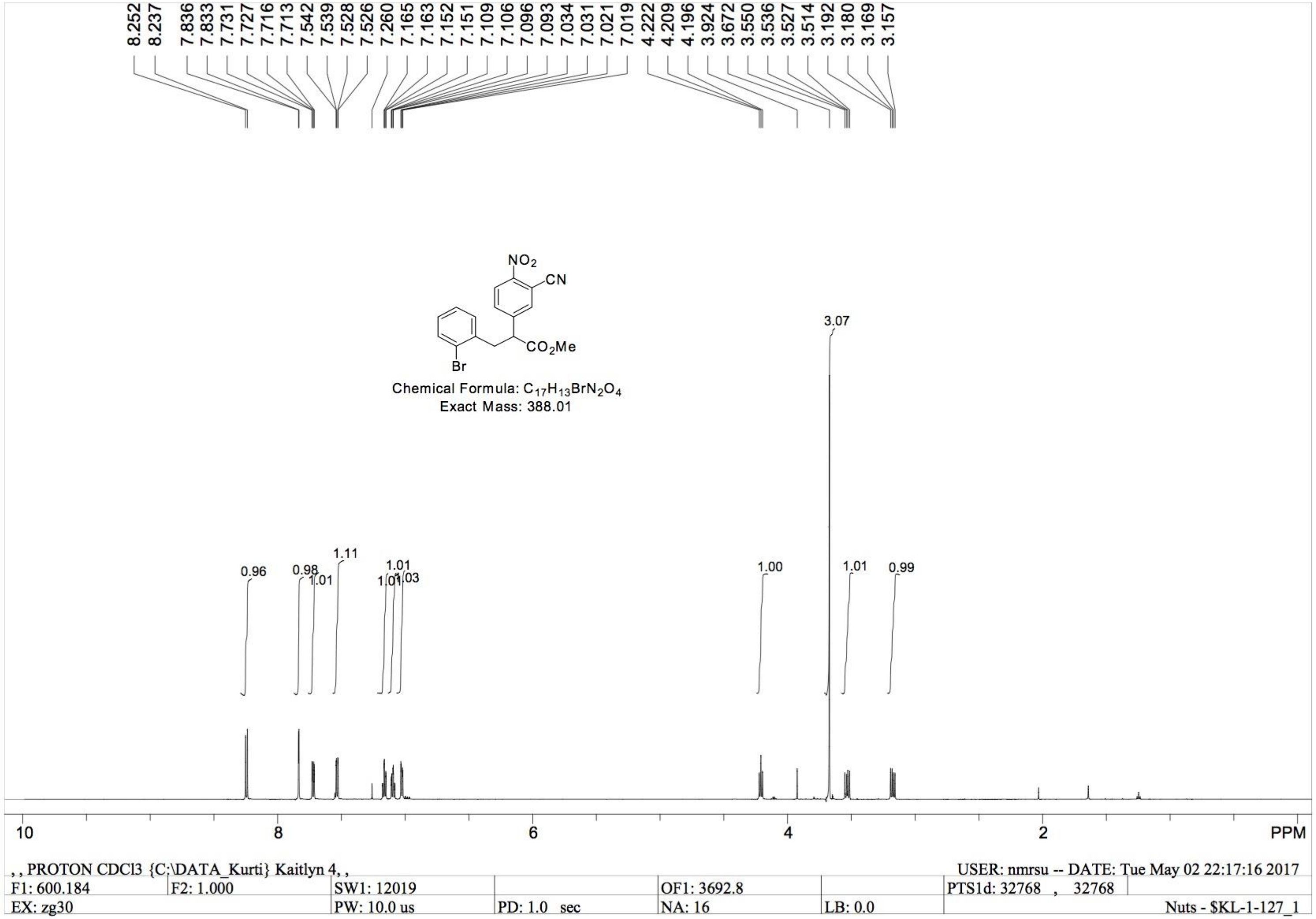

8.252
8.237
7.836
7.833
7.731
7.727
7.716
7.713
7.542
7.539
7.528
7.526
7.260
7.165
7.163
7.152
7.151
7.109
7.106
7.096
7.093
7.034
7.031
7.021
7.019
4.222
4.209
4.196
3.924
3.672
3.550
3.536
3.527
3.514
3.192
3.180
3.169
3.157
NO2
CN
CO2Me
Br
Chemical Formula: C17H13BrN2O4
Exact Mass: 388.01
3.07
0.96
0.98
1.01
1.11
1.01
1.01
1.03
1.00
1.01
0.99
10
8
6
4
2
PPM
, , PROTON CDCl3 {C:\DATA_Kurti} Kaitlyn 4, ,
USER: nmrsu -- DATE: Tue May 02 22:17:16 2017
F1: 600.184
F2: 1.000
SW1: 12019
OF1: 3692.8
PTS1d: 32768 , 32768
EX: zg30
PW: 10.0 us
PD: 1.0 sec
NA: 16
LB: 0.0
Nuts - $KL-1-127_1

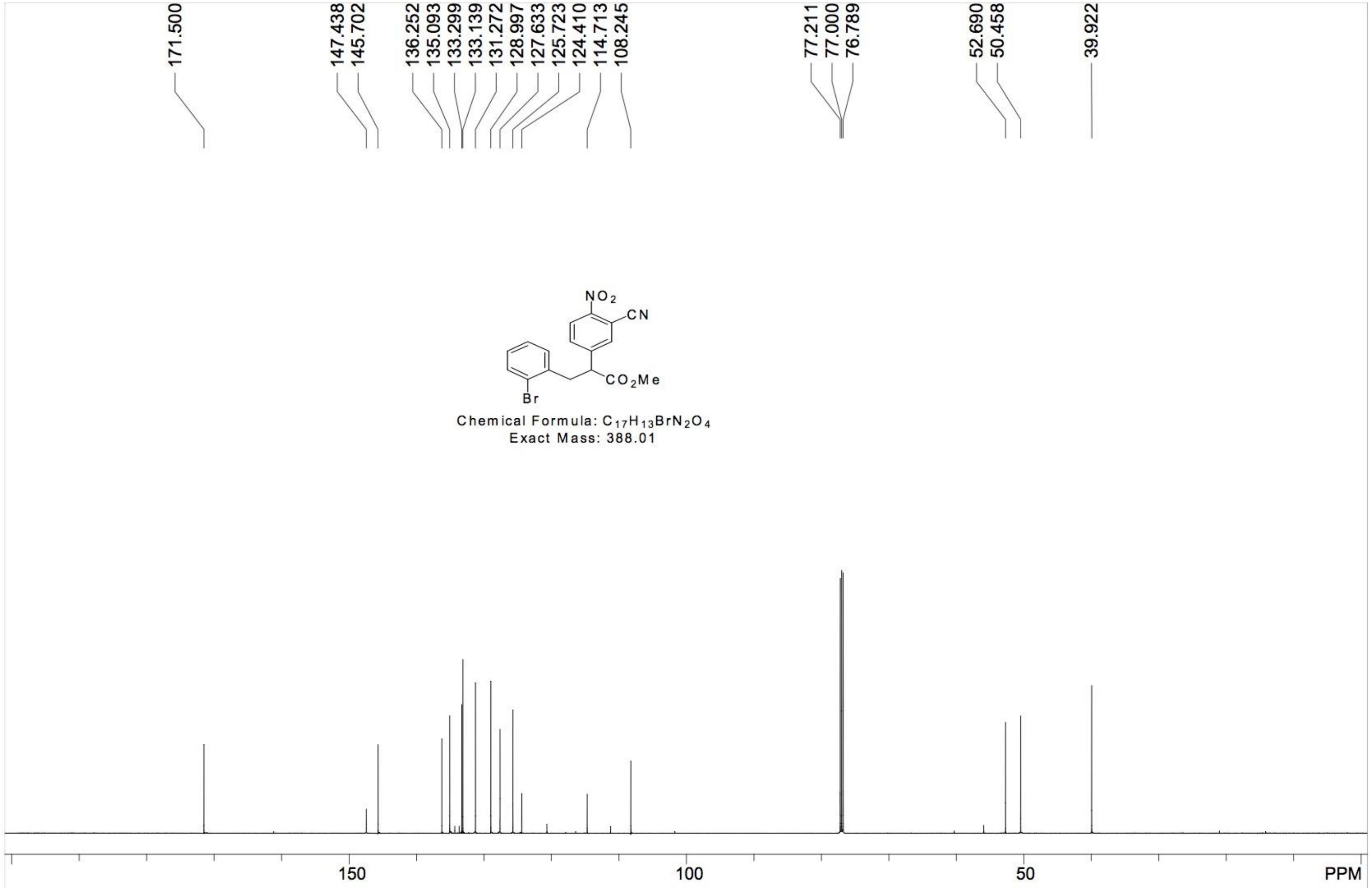

, , C13CPD CDCl3 {C:\DATA_Kurti} Kaitlyn 4, ,						USER: nmrsu -- DATE: Tue May 02 22:22:44 2017
F1: 150.930	F2: 1.000	SW1: 36058		OF1: 15078.6		PTS1d: 32768 , 32768
EX: zgpg30		PW: 10.0 us	PD: 2.0 sec	NA: 100	LB: 0.2	Nuts - $KL-1-127_2

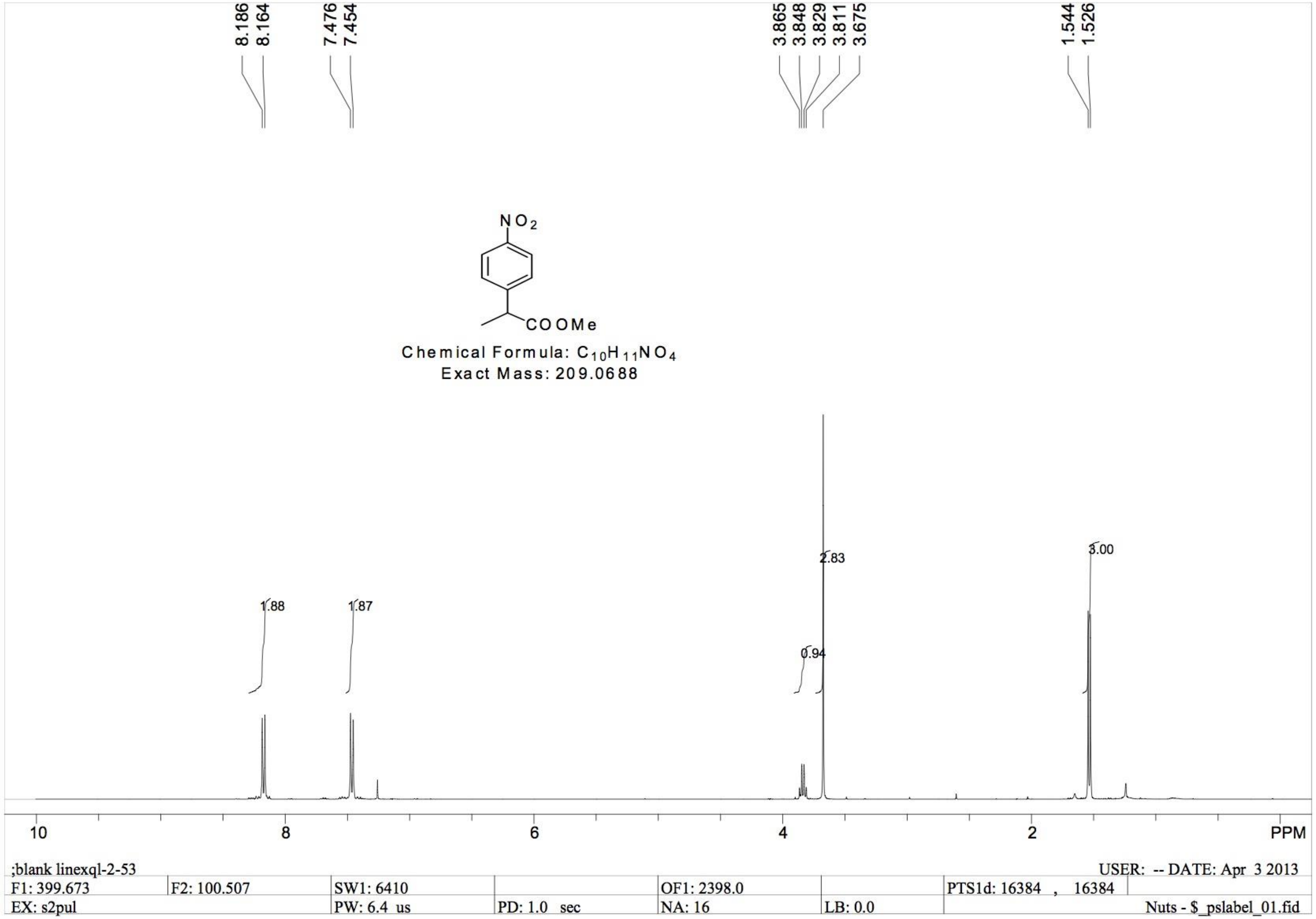
8.186
8.164
7.476
7.454
3.865
3.848
3.829
3.811
3.675
1.544
1.526
NO2
COOMe
Chemical Formula: C10H11NO4
Exact Mass: 209.0688
1.88
1.87
0.94
2.83
3.00
10
8
6
4
2
PPM
;blank linexql-2-53
USER: -- DATE: Apr 3 2013
F1: 399.673
F2: 100.507
SW1: 6410
OF1: 2398.0
PTS1d: 16384 , 16384
EX: s2pul
PW: 6.4 us
PD: 1.0 sec
NA: 16
LB: 0.0
Nuts - $_pslabel_01.fid

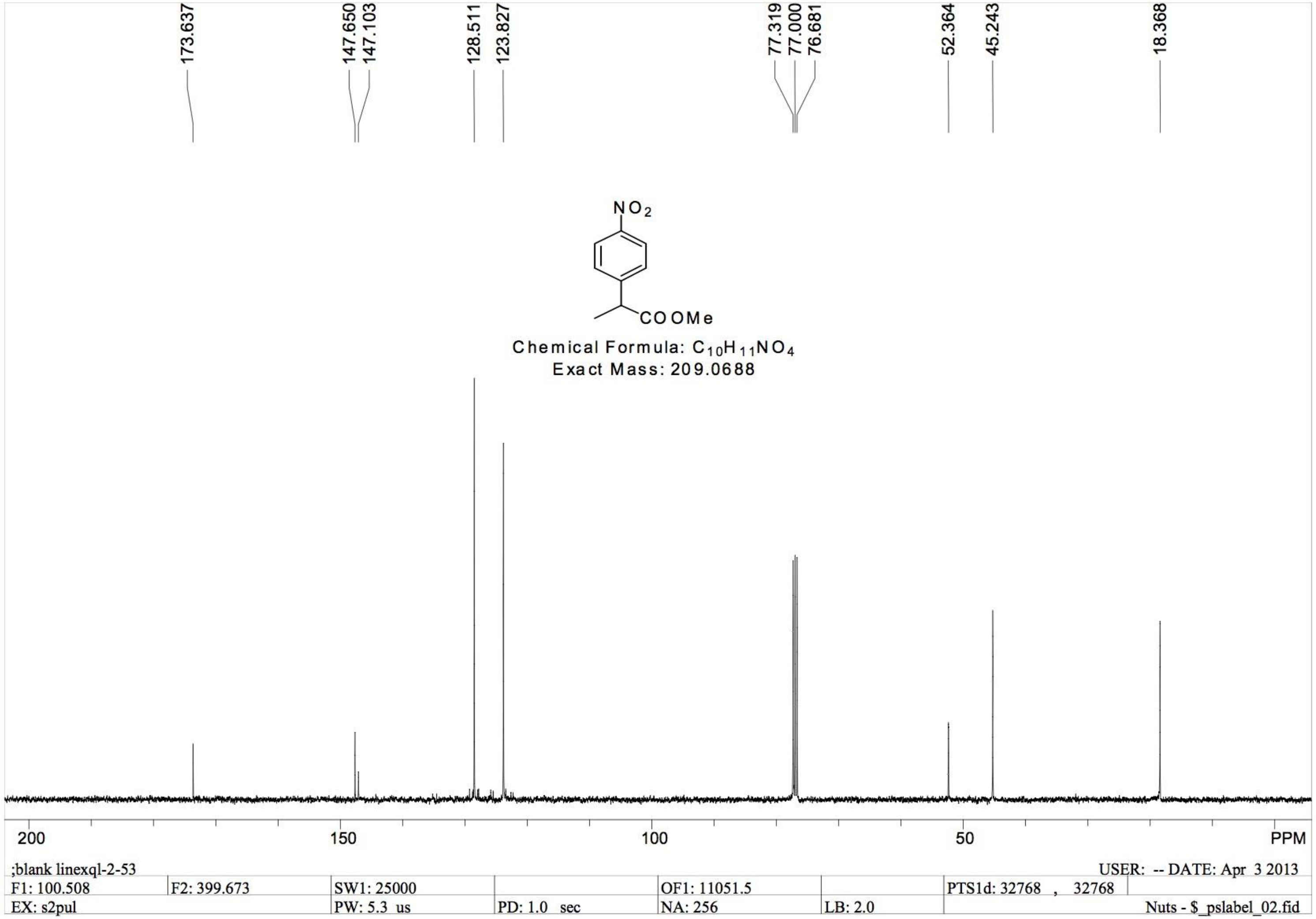
173.637
147.650
147.103
128.511
123.827
77.319
77.000
76.681
52.364
45.243
18.368
NO2
COOMe
Chemical Formula: C10H11NO4
Exact Mass: 209.0688
200
150
100
50
PPM
;blank linexql-2-53
USER: -- DATE: Apr 3 2013
F1: 100.508
F2: 399.673
SW1: 25000
OF1: 11051.5
PTS1d: 32768 , 32768
EX: s2pul
PW: 5.3 us
PD: 1.0 sec
NA: 256
LB: 2.0
Nuts - $_pslabel_02.fid

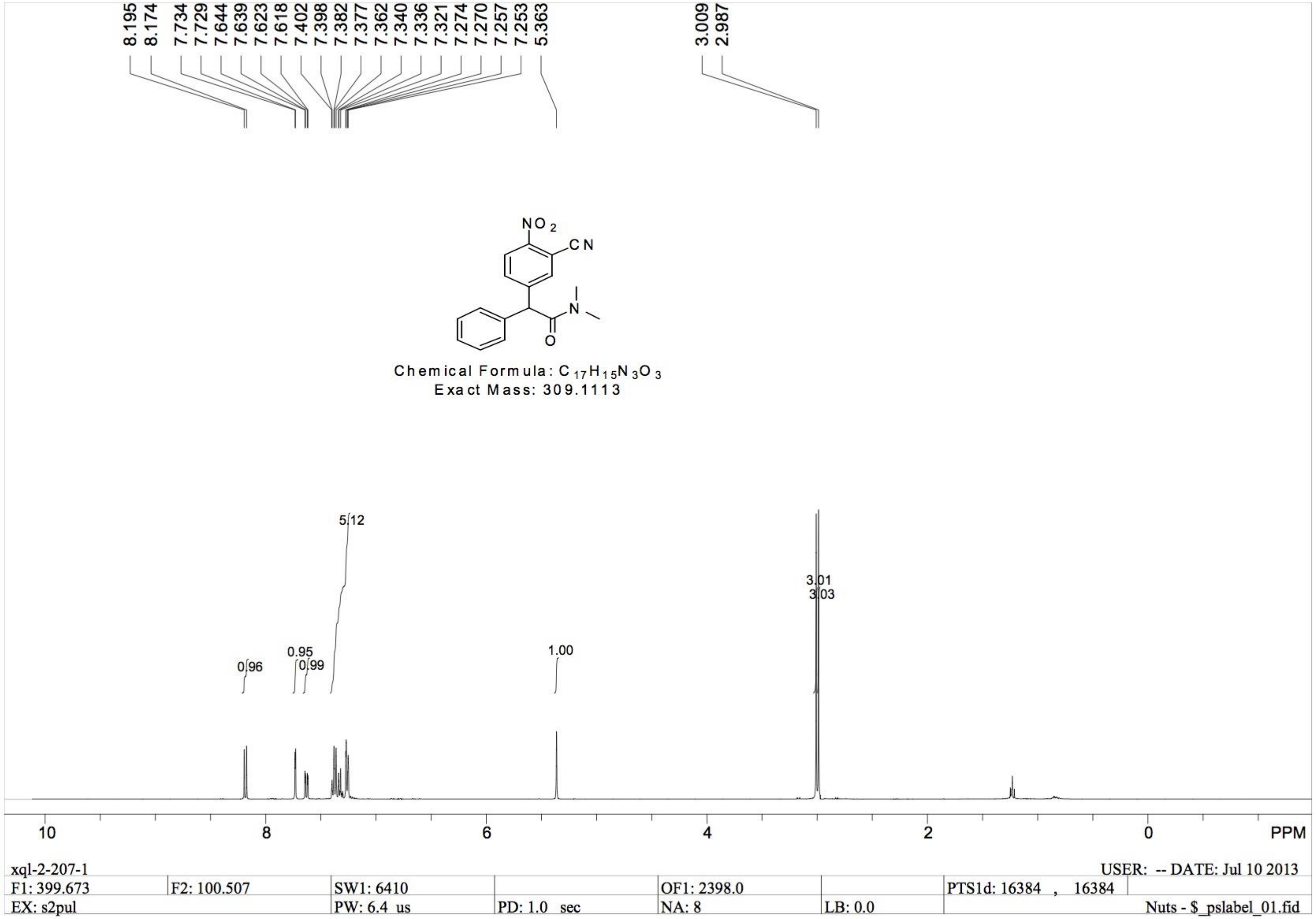
8.195
8.174
7.734
7.729
7.644
7.639
7.623
7.618
7.402
7.398
7.382
7.377
7.362
7.340
7.336
7.321
7.274
7.270
7.257
7.253
5.363
3.009
2.987
NO_2
CN
N
O
Chemical Formula: $C_{17}H_{15}N_3O_3$
Exact Mass: 309.1113
5.12
3.01
3.03
0.96
0.95
0.99
1.00
10
8
6
4
2
0
PPM
xql-2-207-1
USER: -- DATE: Jul 10 2013
F1: 399.673
F2: 100.507
SW1: 6410
OF1: 2398.0
PTS1d: 16384 , 16384
EX: s2pul
PW: 6.4 us
PD: 1.0 sec
NA: 8
LB: 0.0
Nuts - $_pslabel_01.fid

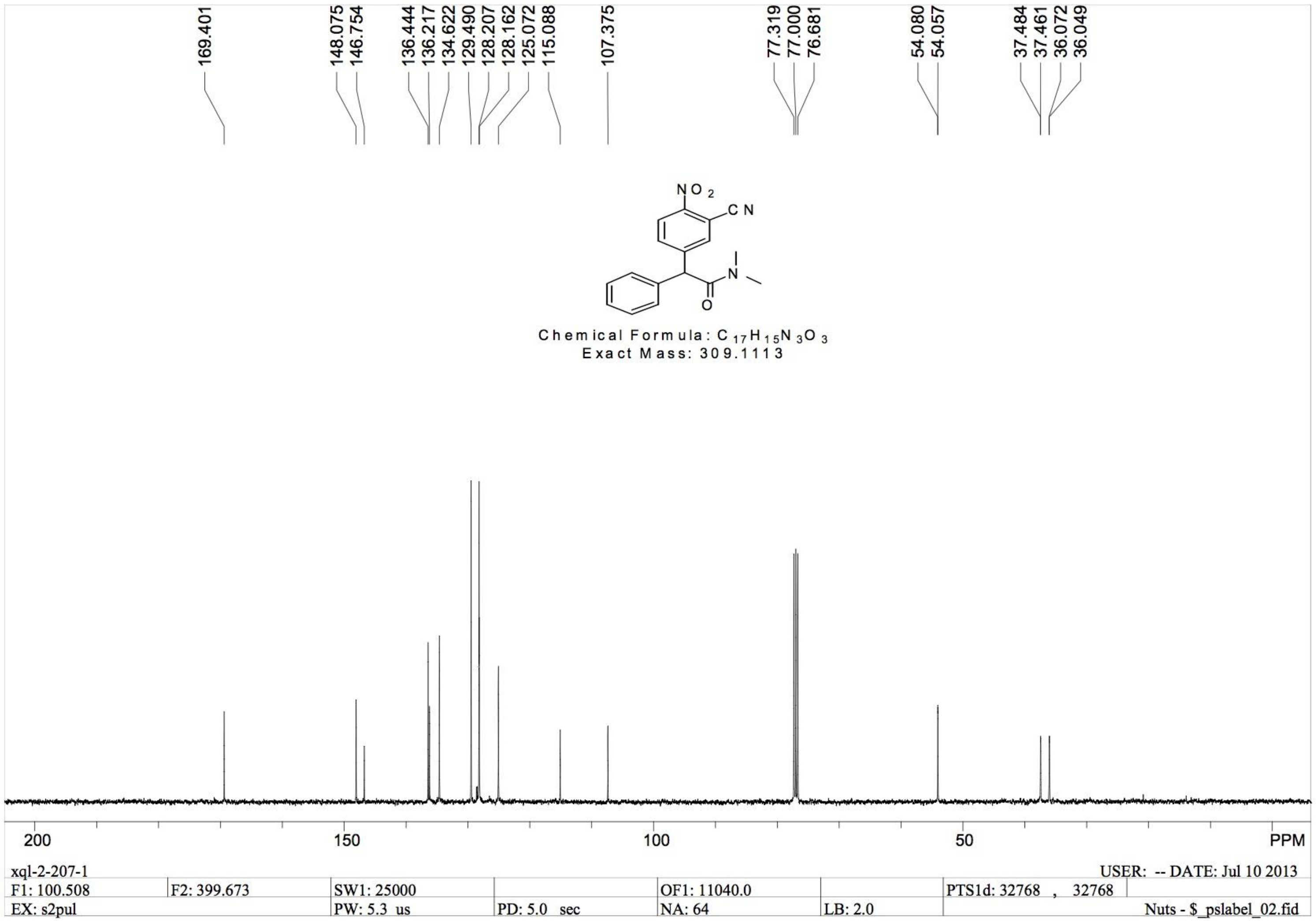
169.401
148.075
146.754
136.444
136.217
134.622
129.490
128.207
128.162
125.072
115.088
107.375
77.319
77.000
76.681
54.080
54.057
37.484
37.461
36.072
36.049
NO2
CN
N
O
Chemical Formula: C17H15N3O3
Exact Mass: 309.1113
200
150
100
50
PPM
xql-2-207-1
USER: -- DATE: Jul 10 2013
F1: 100.508
F2: 399.673
SW1: 25000
OF1: 11040.0
PTS1d: 32768 , 32768
EX: s2pul
PW: 5.3 us
PD: 5.0 sec
NA: 64
LB: 2.0
Nuts - $_pslabel_02.fid

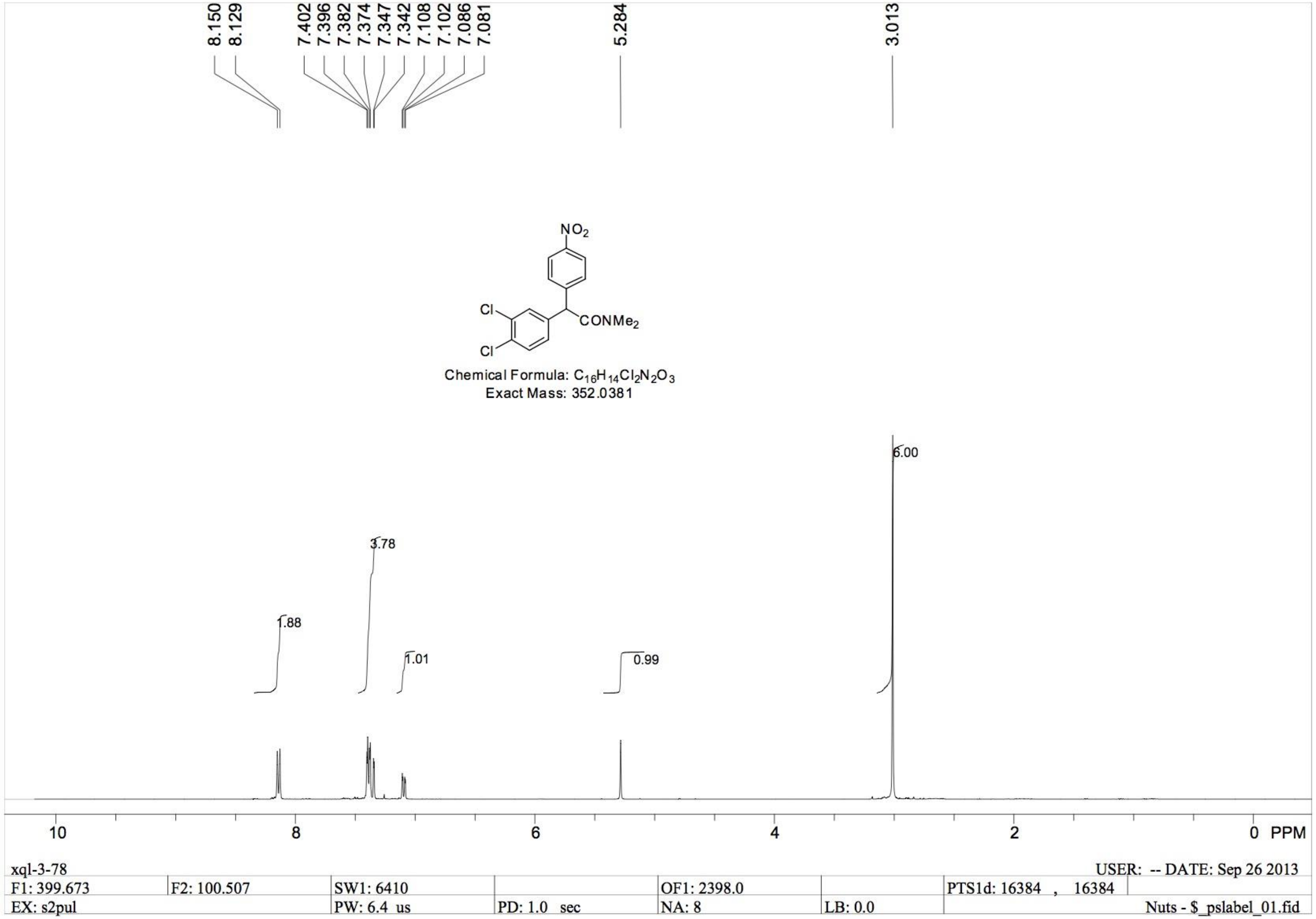
8.150
8.129
7.402
7.396
7.382
7.374
7.347
7.342
7.108
7.102
7.086
7.081
5.284
3.013
NO2
Cl
Cl
CONMe2
Chemical Formula: C16H14Cl2N2O3
Exact Mass: 352.0381
6.00
3.78
1.88
1.01
0.99
10
8
6
4
2
0 PPM
xql-3-78
USER: -- DATE: Sep 26 2013
F1: 399.673
F2: 100.507
SW1: 6410
OF1: 2398.0
PTS1d: 16384 , 16384
EX: s2pul
PW: 6.4 us
PD: 1.0 sec
NA: 8
LB: 0.0
Nuts - $_pslabel_01.fid

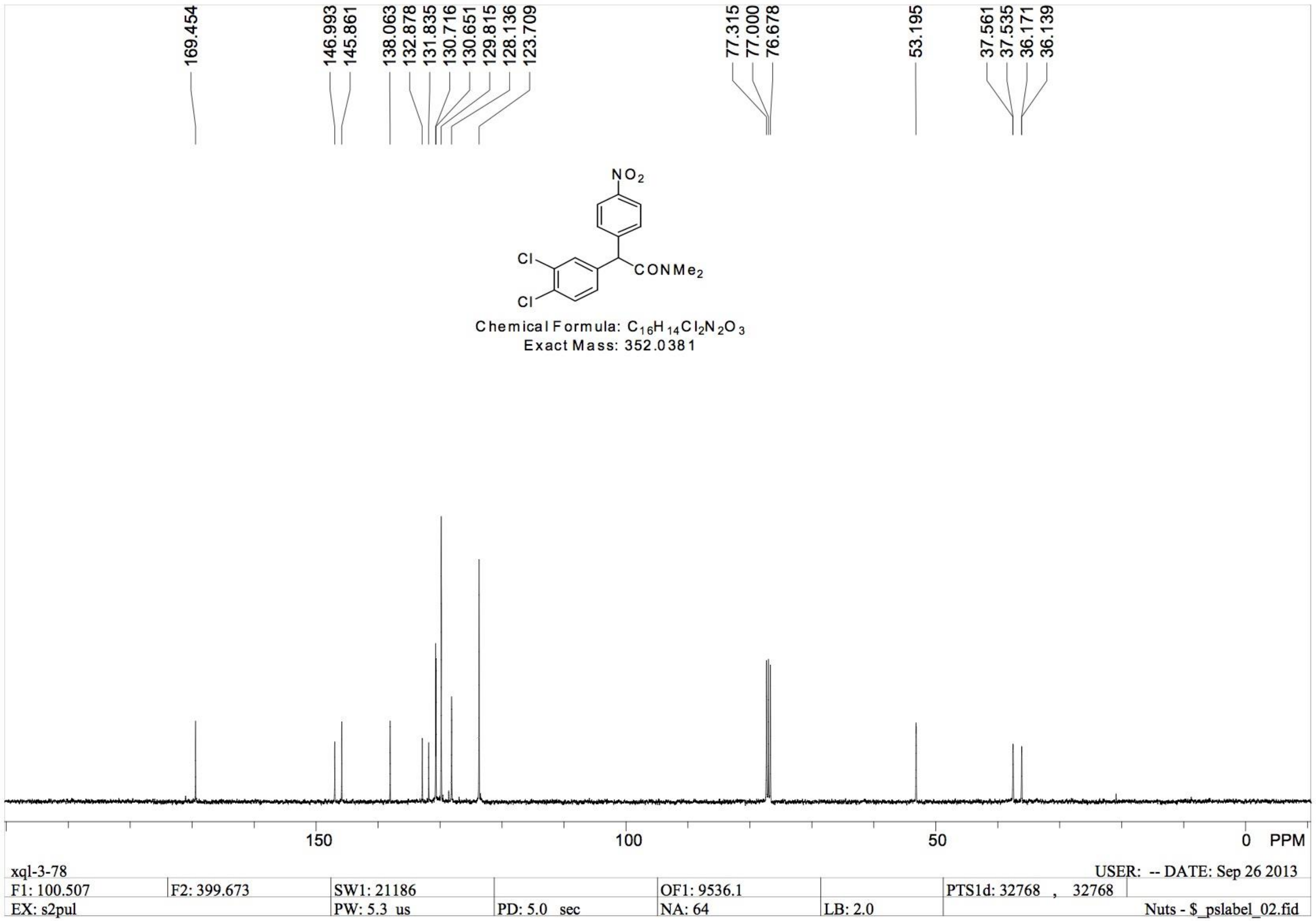
169.454
146.993
145.861
138.063
132.878
131.835
130.716
130.651
129.815
128.136
123.709
77.315
77.000
76.678
53.195
37.561
37.535
36.171
36.139
NO2
Cl
Cl
CONMe2
Chemical Formula: C16H14Cl2N2O3
Exact Mass: 352.0381
150
100
50
0 PPM
xql-3-78
USER: -- DATE: Sep 26 2013
F1: 100.507
F2: 399.673
SW1: 21186
OF1: 9536.1
PTS1d: 32768 , 32768
EX: s2pul
PW: 5.3 us
PD: 5.0 sec
NA: 64
LB: 2.0
Nuts - $_pslabel_02.fid

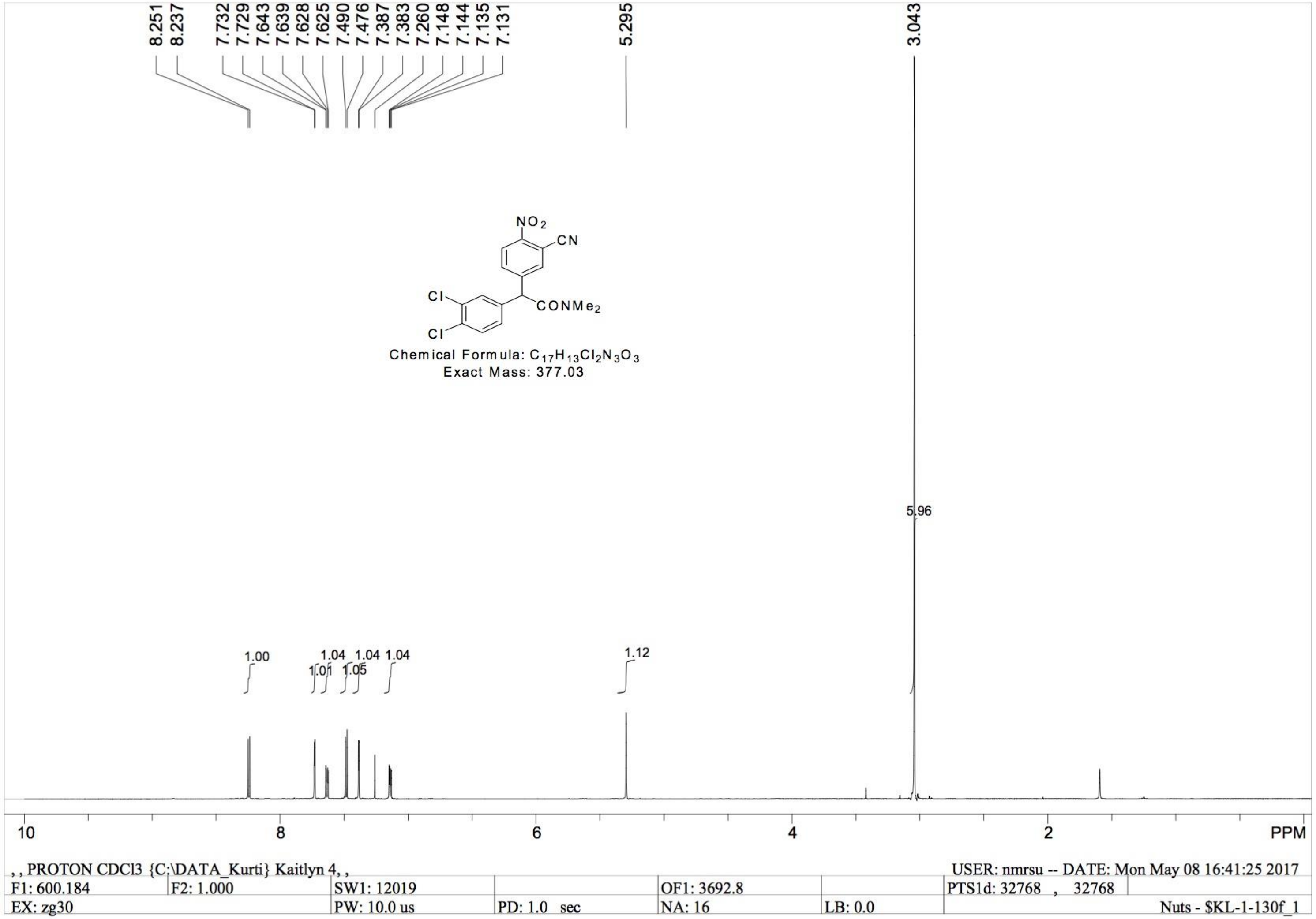
8.251
8.237
7.732
7.729
7.643
7.639
7.628
7.625
7.490
7.476
7.387
7.383
7.260
7.148
7.144
7.135
7.131
5.295
3.043
NO2
CN
Cl
Cl
CONMe2
Chemical Formula: C17H13Cl2N3O3
Exact Mass: 377.03
5.96
1.00
1.04
1.01
1.04
1.05
1.04
1.12
10
8
6
4
2
PPM
, , PROTON CDCl3 {C:\DATA_Kurti} Kaitlyn 4, ,
USER: nmrsu -- DATE: Mon May 08 16:41:25 2017
F1: 600.184
F2: 1.000
SW1: 12019
OF1: 3692.8
PTS1d: 32768 , 32768
EX: zg30
PW: 10.0 us
PD: 1.0 sec
NA: 16
LB: 0.0
Nuts - $KL-1-130f_1

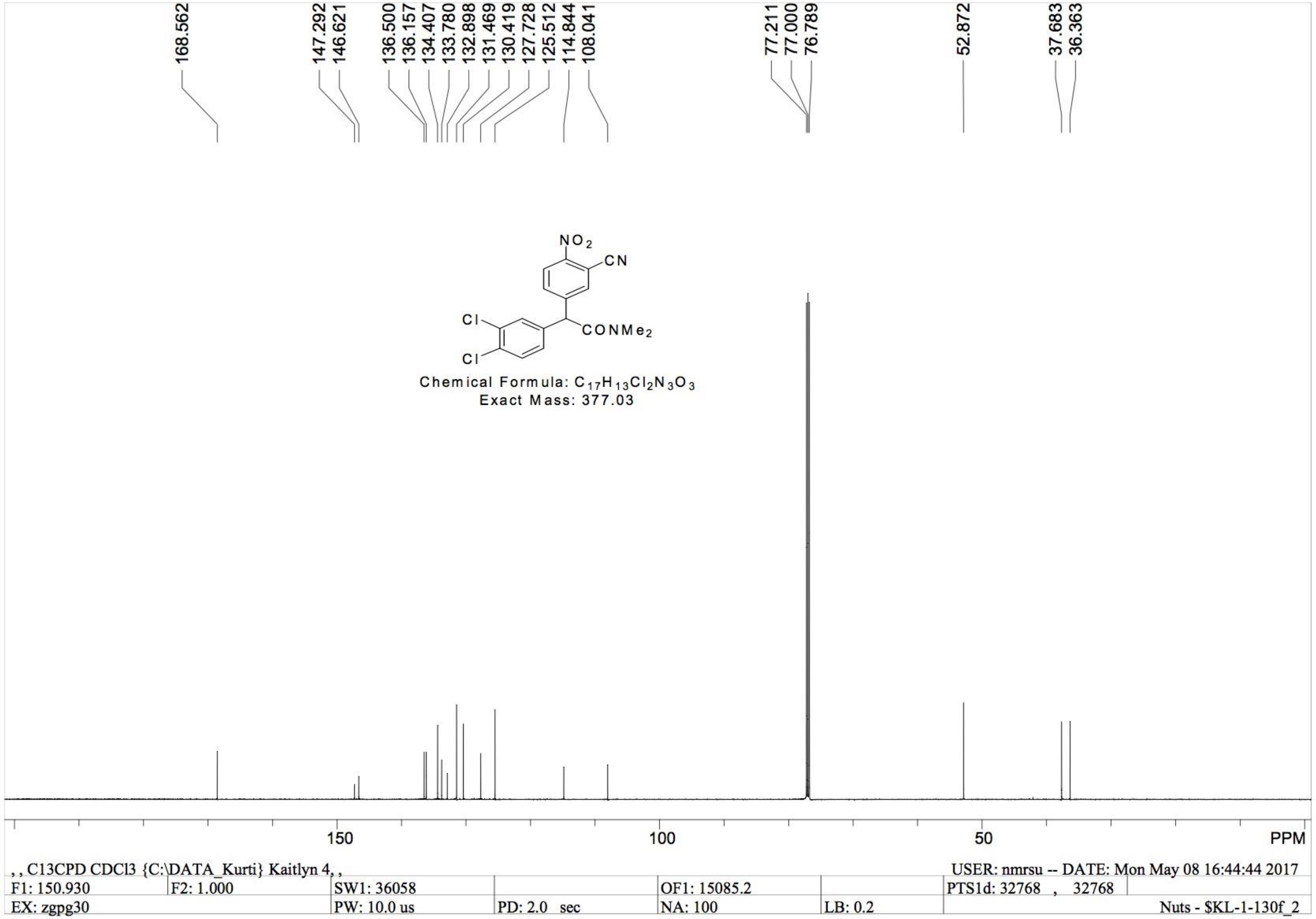
168.562
147.292
146.621
136.500
136.157
134.407
133.780
132.898
131.469
130.419
127.728
125.512
114.844
108.041
77.211
77.000
76.789
52.872
37.683
36.363
NO2
CN
Cl
Cl
CONMe2
Chemical Formula: C17H13Cl2N3O3
Exact Mass: 377.03
150
100
50
PPM
, , C13CPD CDCl3 {C:\DATA_Kurti} Kaitlyn 4, ,
USER: nmrsu -- DATE: Mon May 08 16:44:44 2017
F1: 150.930
F2: 1.000
SW1: 36058
OF1: 15085.2
PTS1d: 32768 , 32768
EX: zgpg30
PW: 10.0 us
PD: 2.0 sec
NA: 100
LB: 0.2
Nuts - $KL-1-130f_2

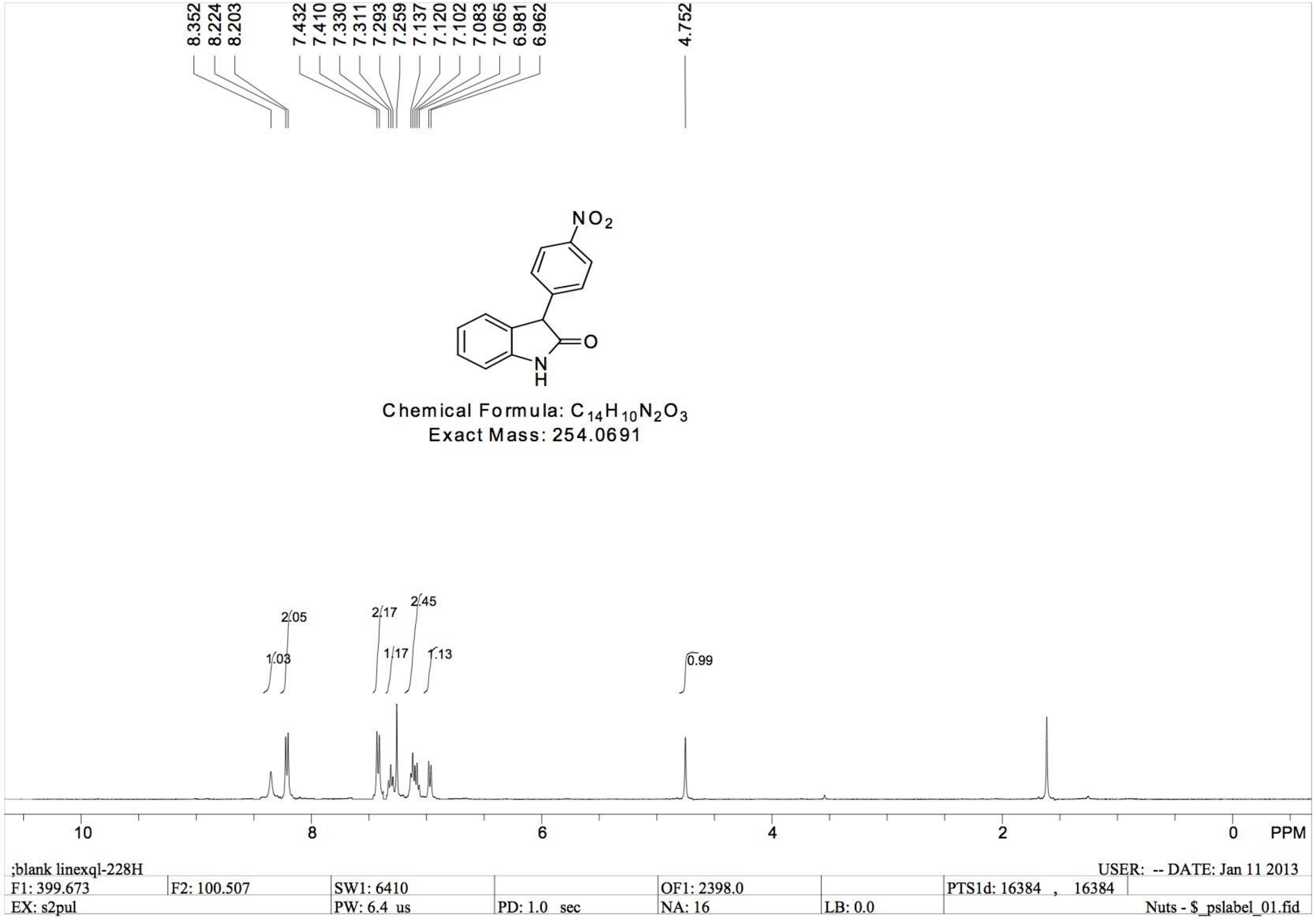
8.352
8.224
8.203
7.432
7.410
7.330
7.311
7.293
7.259
7.137
7.120
7.102
7.083
7.065
6.981
6.962
4.752
NO2
O
N
H
Chemical Formula: C14H10N2O3
Exact Mass: 254.0691
1.03
2.05
2.17
1.17
2.45
1.13
0.99
10
8
6
4
2
0
PPM
;blank linexql-228H
USER: -- DATE: Jan 11 2013
F1: 399.673
F2: 100.507
SW1: 6410
OF1: 2398.0
PTS1d: 16384 , 16384
EX: s2pul
PW: 6.4 us
PD: 1.0 sec
NA: 16
LB: 0.0
Nuts - $_pslabel_01.fid

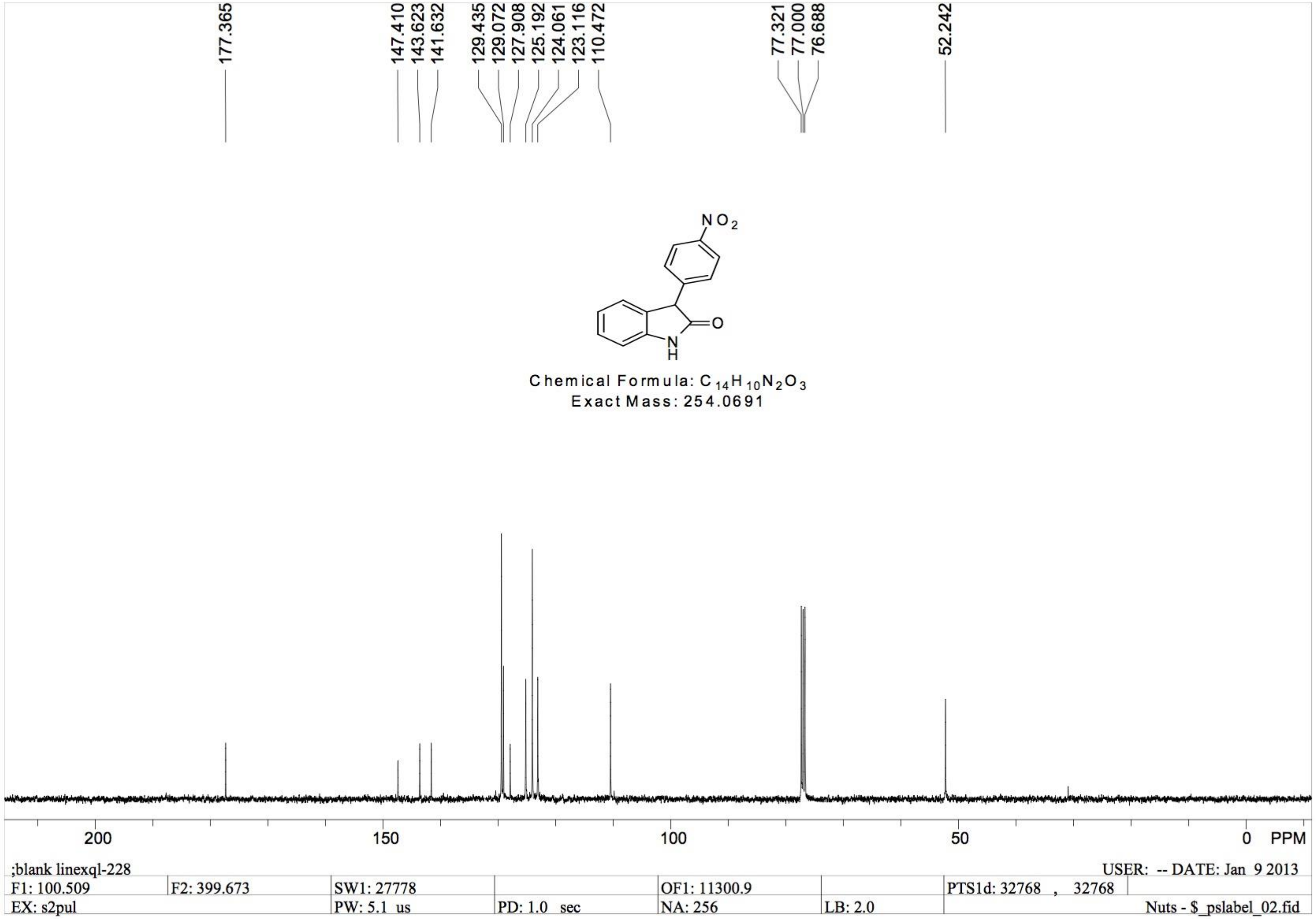
177.365
147.410
143.623
141.632
129.435
129.072
127.908
125.192
124.061
123.116
110.472
77.321
77.000
76.688
52.242
NO2
O
N
H
Chemical Formula: C14H10N2O3
Exact Mass: 254.0691
200
150
100
50
0 PPM
;blank linexql-228
USER: -- DATE: Jan 9 2013
F1: 100.509
F2: 399.673
SW1: 27778
OF1: 11300.9
PTS1d: 32768 , 32768
EX: s2pul
PW: 5.1 us
PD: 1.0 sec
NA: 256
LB: 2.0
Nuts - $_pslabel_02.fid

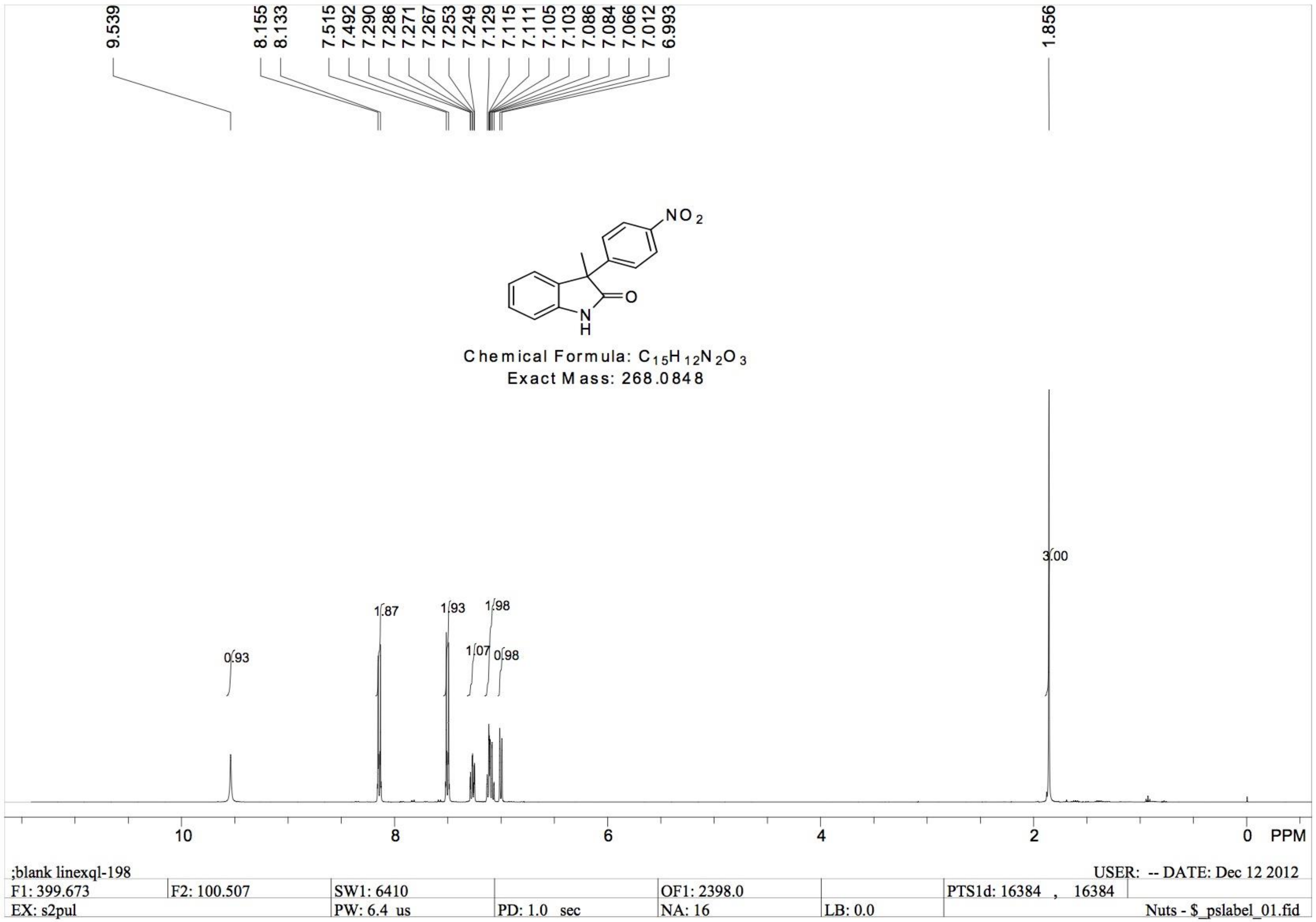

9.539
8.155
8.133
7.515
7.492
7.290
7.286
7.271
7.267
7.253
7.249
7.129
7.115
7.111
7.105
7.103
7.086
7.084
7.066
7.012
6.993
1.856
NO2
O
N
H
Chemical Formula: C15H12N2O3
Exact Mass: 268.0848
0.93
1.87
1.93
1.07
1.98
0.98
3.00
10
8
6
4
2
0
PPM
;blank linexql-198
USER: -- DATE: Dec 12 2012
F1: 399.673
F2: 100.507
SW1: 6410
OF1: 2398.0
PTS1d: 16384 , 16384
EX: s2pul
PW: 6.4 us
PD: 1.0 sec
NA: 16
LB: 0.0
Nuts - $_pslabel_01.fid

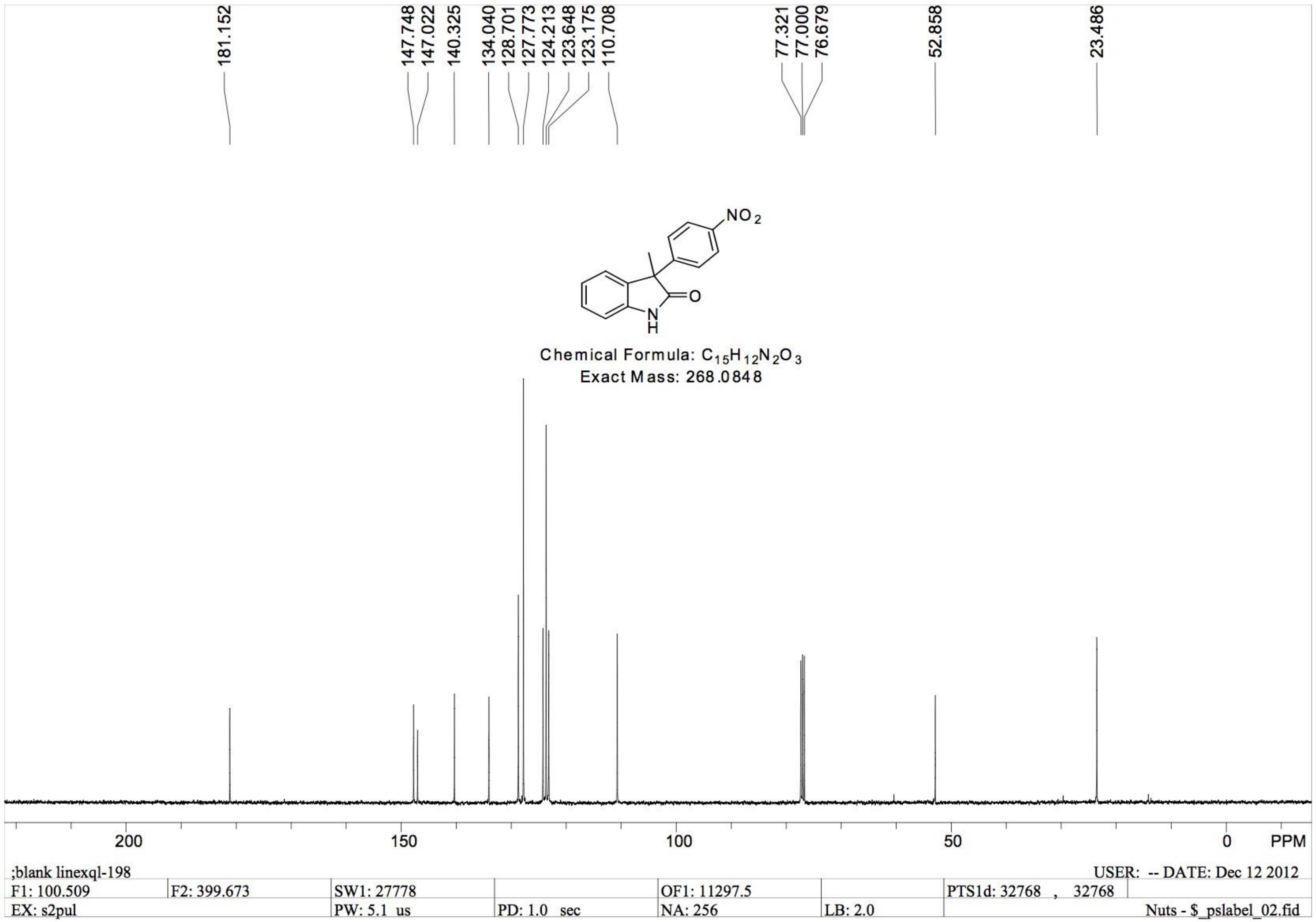

181.152
147.748
147.022
140.325
134.040
128.701
127.773
124.213
123.648
123.175
110.708
77.321
77.000
76.679
52.858
23.486
NO2
O
N
H
Chemical Formula: C15H12N2O3
Exact Mass: 268.0848
200
150
100
50
0
PPM
;blank linexql-198
USER: -- DATE: Dec 12 2012
F1: 100.509
F2: 399.673
SW1: 27778
OF1: 11297.5
PTS1d: 32768 , 32768
EX: s2pul
PW: 5.1 us
PD: 1.0 sec
NA: 256
LB: 2.0
Nuts - $_pslabel_02.fid

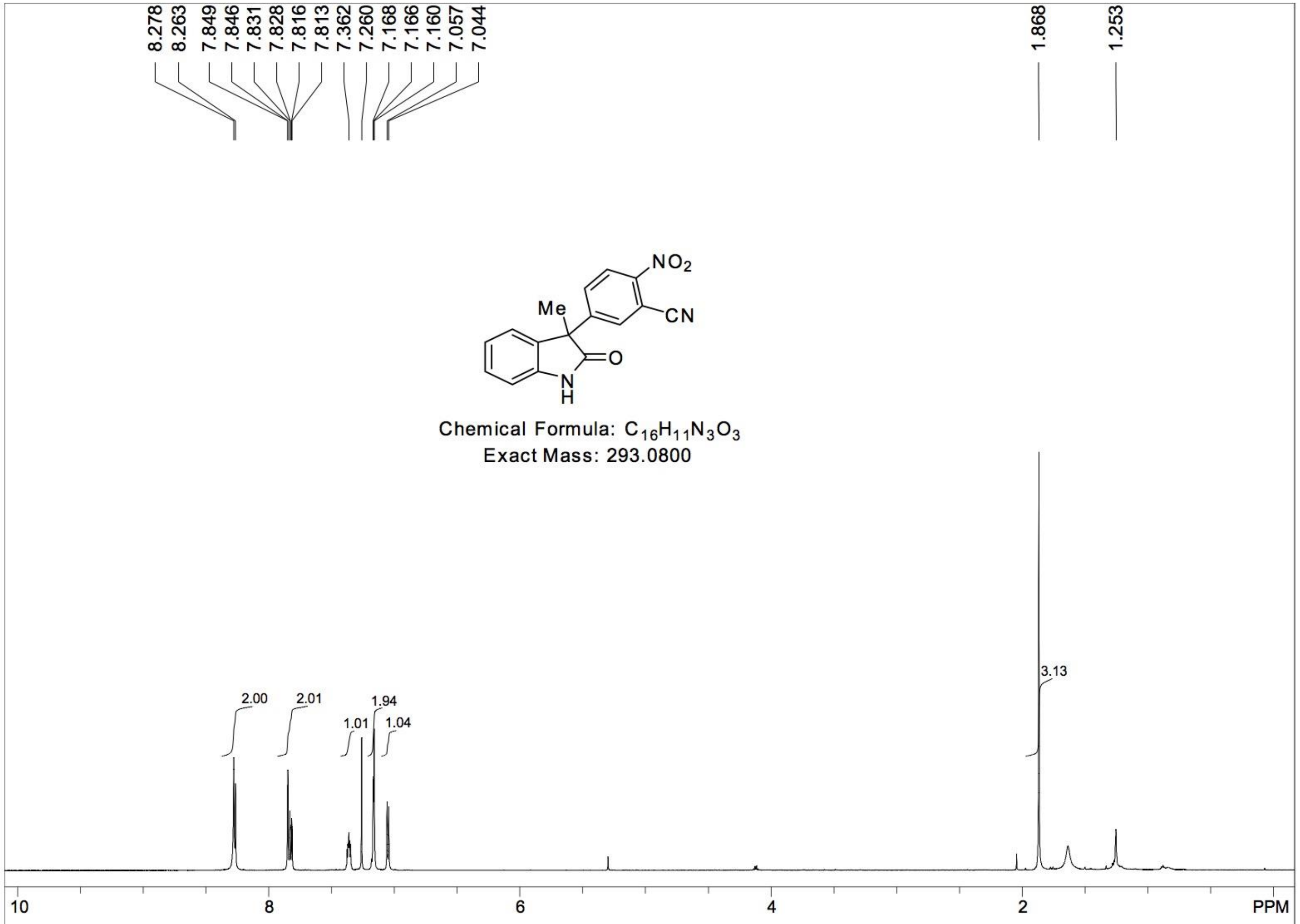

, , PROTON CDCl3 {C:\DATA_Kurti} Gao 6, , USER: nmrsu -- DATE: Fri Sep 09 16:31:10 2016

F1: 600.184	F2: 1.000	SW1: 12019		OF1: 3692.8		PTS1d: 32768 , 32768	
EX: zg30		PW: 10.0 us	PD: 1.0 sec	NA: 8	LB: 0.0		Nuts - $ghy-5-82_1

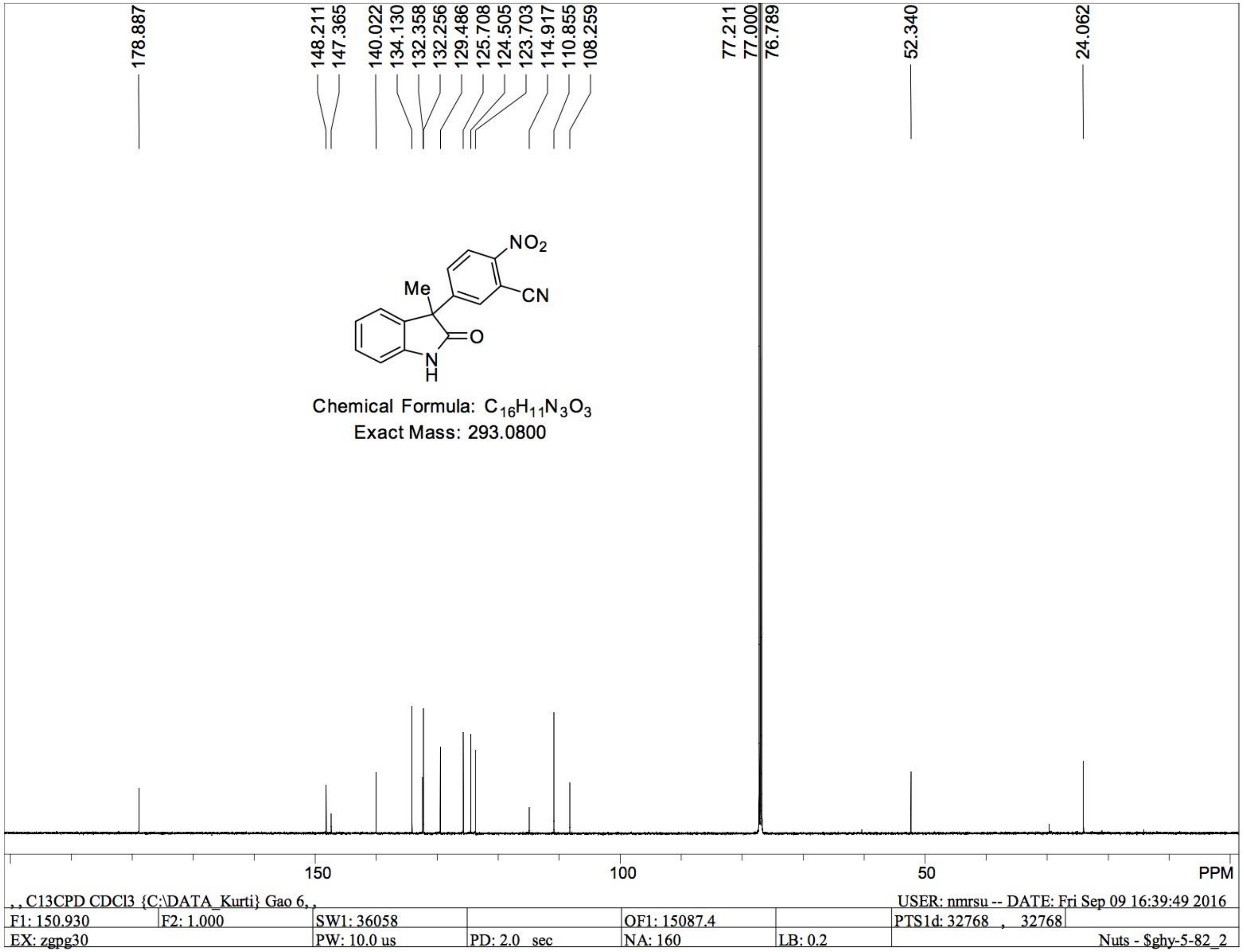
178.887
148.211
147.365
140.022
134.130
132.358
132.256
129.486
125.708
124.505
123.703
114.917
110.855
108.259
77.211
77.000
76.789
52.340
24.062
NO2
Me
CN
O
N
H
Chemical Formula: C16H11N3O3
Exact Mass: 293.0800
150
100
50
PPM
, , C13CPD CDCl3 {C:\DATA_Kurti} Gao 6, ,
USER: nmrsu -- DATE: Fri Sep 09 16:39:49 2016
F1: 150.930
F2: 1.000
SW1: 36058
OF1: 15087.4
PTS1d: 32768 , 32768
EX: zgpg30
PW: 10.0 us
PD: 2.0 sec
NA: 160
LB: 0.2
Nuts - $ghy-5-82_2

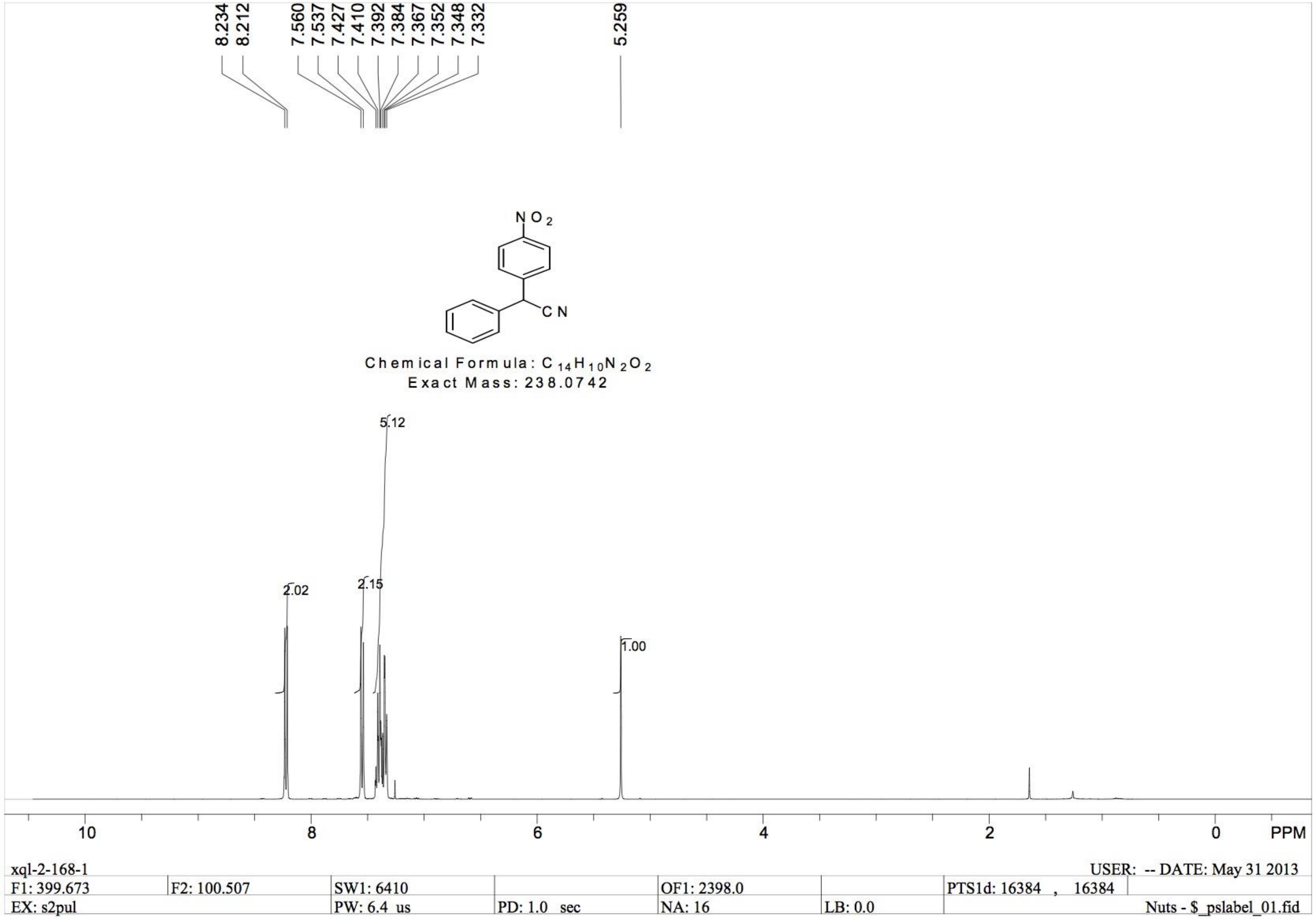
8.234
8.212
7.560
7.537
7.427
7.410
7.392
7.384
7.367
7.352
7.348
7.332
5.259
NO2
CN
Chemical Formula: C14H10N2O2
Exact Mass: 238.0742
5.12
2.02
2.15
1.00
10
8
6
4
2
0
PPM
xql-2-168-1
USER: -- DATE: May 31 2013
F1: 399.673
F2: 100.507
SW1: 6410
OF1: 2398.0
PTS1d: 16384 , 16384
EX: s2pul
PW: 6.4 us
PD: 1.0 sec
NA: 16
LB: 0.0
Nuts - $_pslabel_01.fid

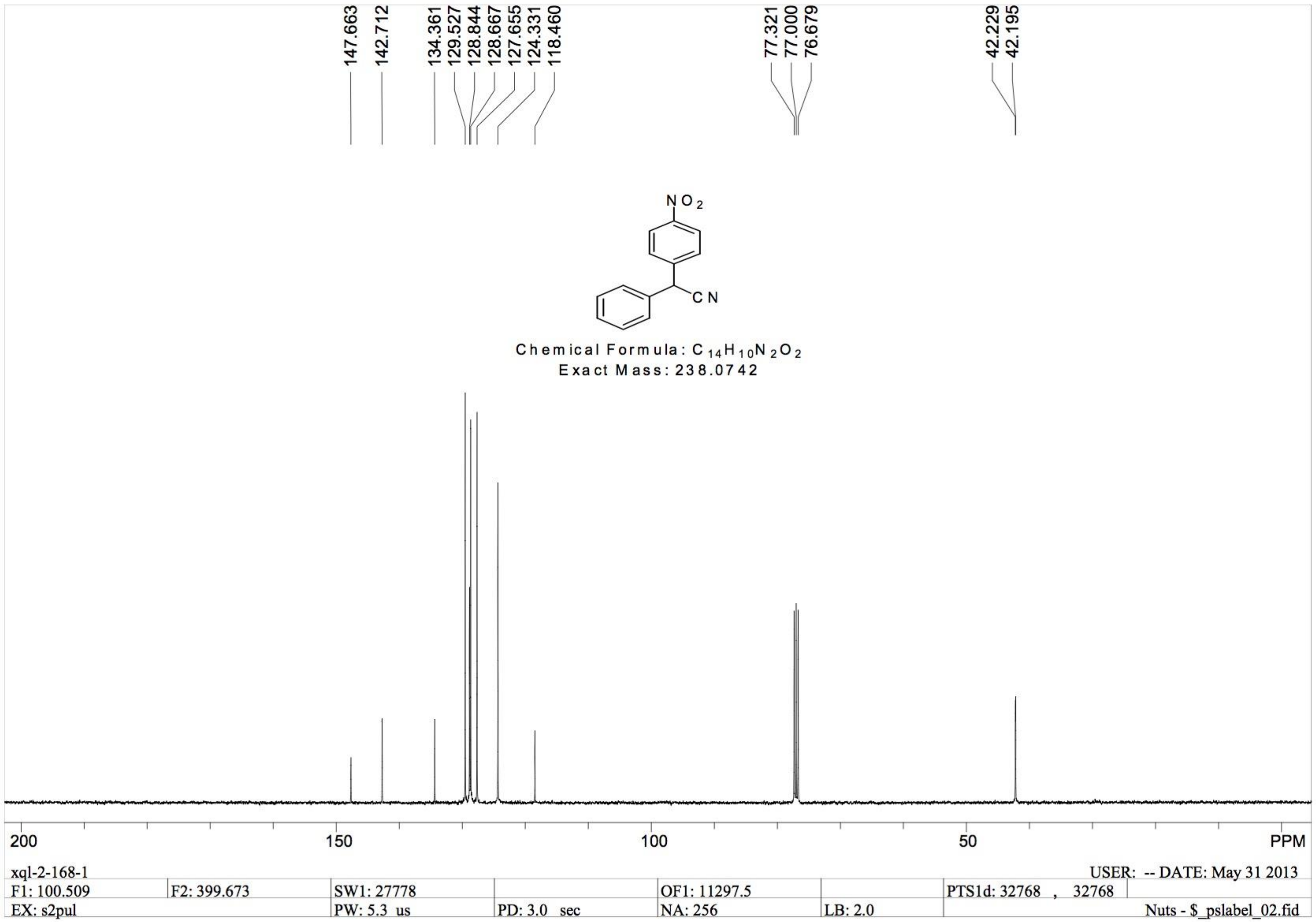
147.663
142.712
134.361
129.527
128.844
128.667
127.655
124.331
118.460
77.321
77.000
76.679
42.229
42.195
NO2
CN
Chemical Formula: C14H10N2O2
Exact Mass: 238.0742
200
150
100
50
PPM
xql-2-168-1
USER: -- DATE: May 31 2013
F1: 100.509
F2: 399.673
SW1: 27778
OF1: 11297.5
PTS1d: 32768 , 32768
EX: s2pul
PW: 5.3 us
PD: 3.0 sec
NA: 256
LB: 2.0
Nuts - $_pslabel_02.fid

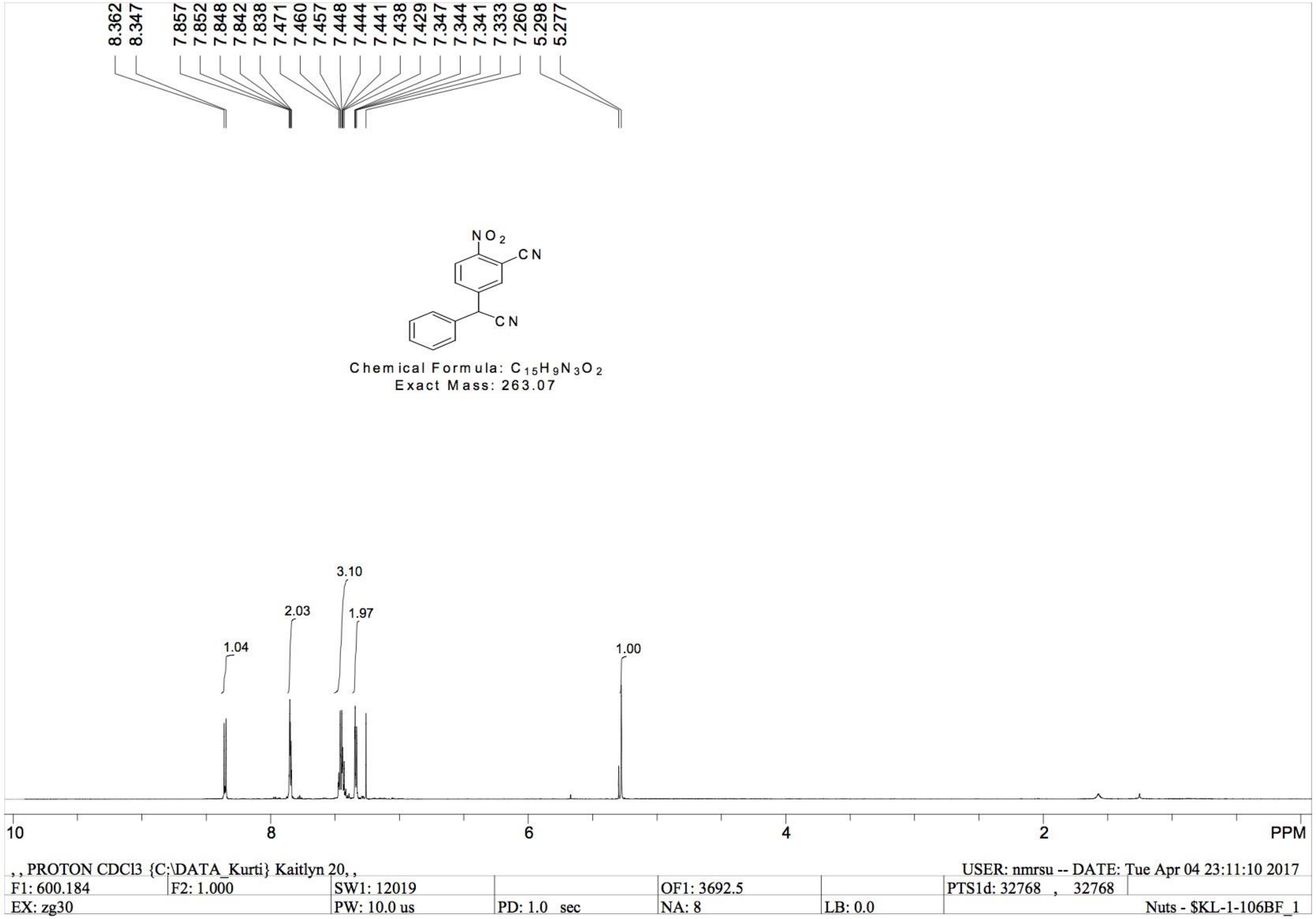
8.362
8.347
7.857
7.852
7.848
7.842
7.838
7.471
7.460
7.457
7.448
7.444
7.441
7.438
7.429
7.347
7.344
7.341
7.333
7.260
5.298
5.277
NO2
CN
CN
Chemical Formula: C15H9N3O2
Exact Mass: 263.07
1.04
2.03
3.10
1.97
1.00
10
8
6
4
2
PPM
, , PROTON CDCl3 {C:\DATA_Kurti} Kaitlyn 20, ,
USER: nmrsu -- DATE: Tue Apr 04 23:11:10 2017
F1: 600.184
F2: 1.000
SW1: 12019
OF1: 3692.5
PTS1d: 32768 , 32768
EX: zg30
PW: 10.0 us
PD: 1.0 sec
NA: 8
LB: 0.0
Nuts - $KL-1-106BF_1

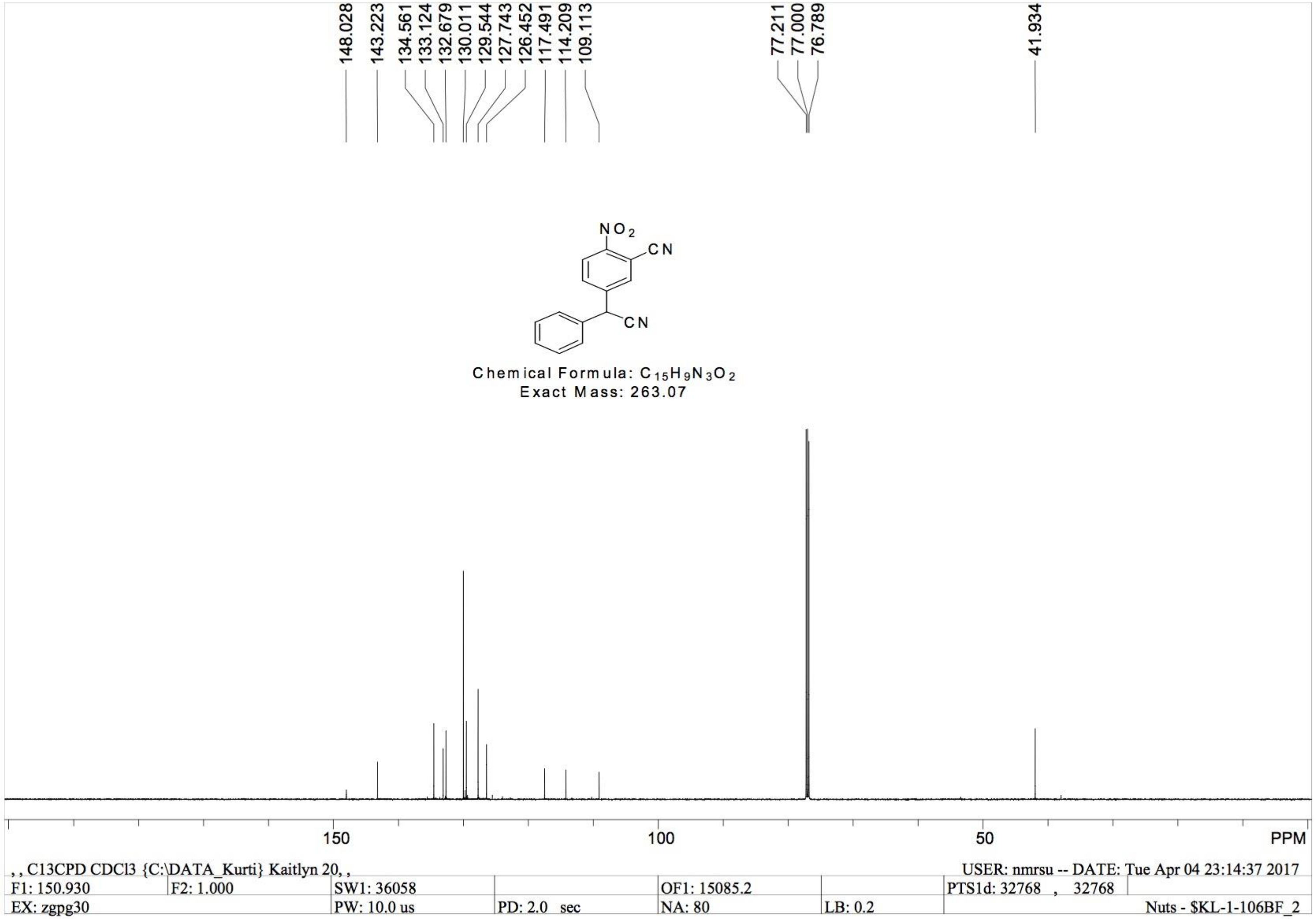
148.028
143.223
134.561
133.124
132.679
130.011
129.544
127.743
126.452
117.491
114.209
109.113
77.211
77.000
76.789
41.934
NO2
CN
CN
Chemical Formula: C15H9N3O2
Exact Mass: 263.07
150
100
50
PPM
, , C13CPD CDCl3 {C:\DATA_Kurti} Kaitlyn 20, ,
USER: nmrsu -- DATE: Tue Apr 04 23:14:37 2017
F1: 150.930
F2: 1.000
SW1: 36058
OF1: 15085.2
PTS1d: 32768 , 32768
EX: zgpg30
PW: 10.0 us
PD: 2.0 sec
NA: 80
LB: 0.2
Nuts - $KL-1-106BF_2

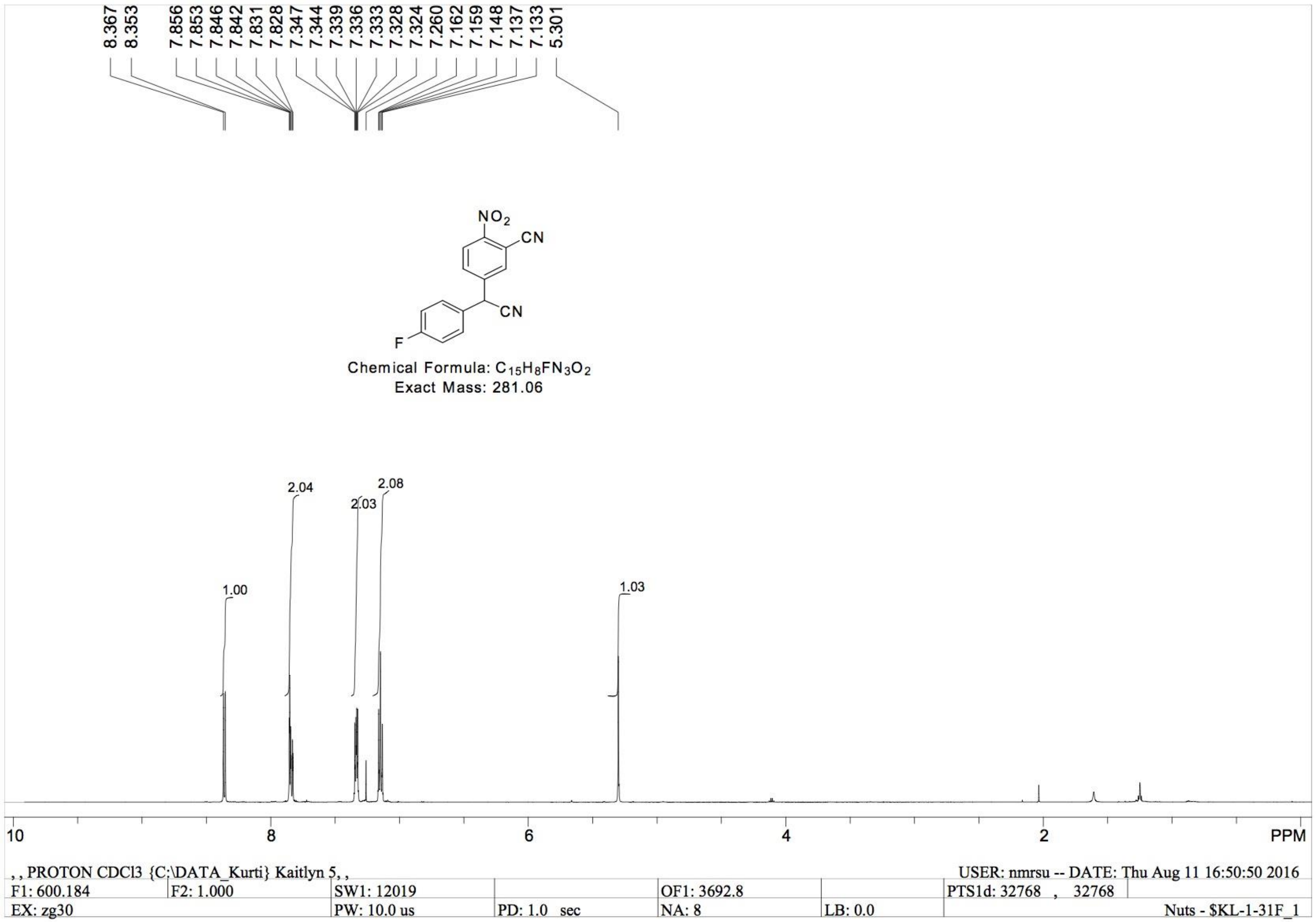
8.367
8.353
7.856
7.853
7.846
7.842
7.831
7.828
7.347
7.344
7.339
7.336
7.333
7.328
7.324
7.260
7.162
7.159
7.148
7.137
7.133
5.301
NO2
CN
CN
F
Chemical Formula: C15H8FN3O2
Exact Mass: 281.06
1.00
2.04
2.03
2.08
1.03
10
8
6
4
2
PPM
, , PROTON CDCl3 {C:\DATA_Kurti} Kaitlyn 5, ,
USER: nmrsu -- DATE: Thu Aug 11 16:50:50 2016
F1: 600.184
F2: 1.000
SW1: 12019
OF1: 3692.8
PTS1d: 32768 , 32768
EX: zg30
PW: 10.0 us
PD: 1.0 sec
NA: 8
LB: 0.0
Nuts - $KL-1-31F_1

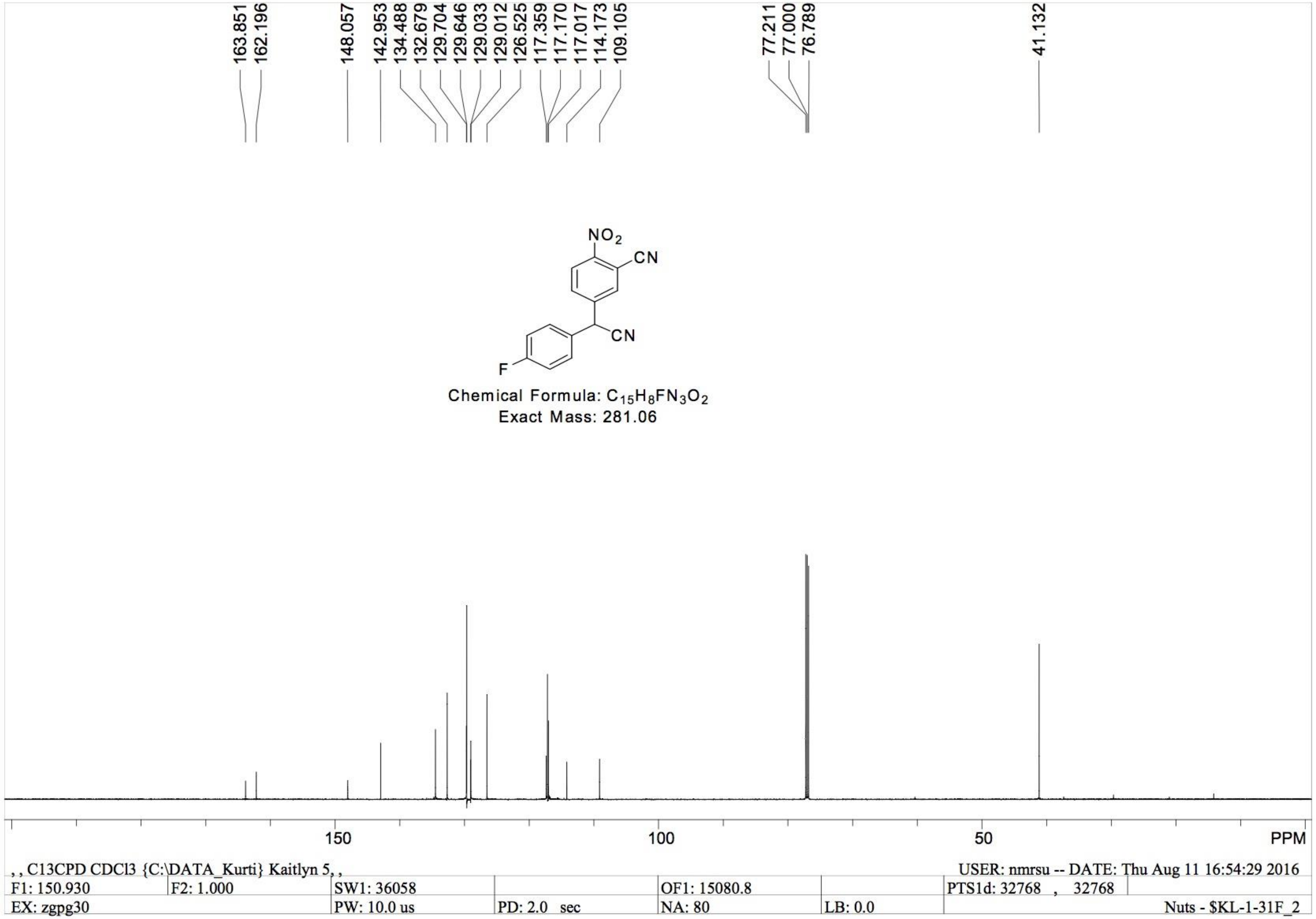
163.851
162.196
148.057
142.953
134.488
132.679
129.704
129.646
129.033
129.012
126.525
117.359
117.170
117.017
114.173
109.105
77.211
77.000
76.789
41.132
NO2
CN
CN
F
Chemical Formula: C15H8FN3O2
Exact Mass: 281.06
150
100
50
PPM
, , C13CPD CDCl3 {C:\DATA_Kurti} Kaitlyn 5, ,
USER: nmrsu -- DATE: Thu Aug 11 16:54:29 2016
F1: 150.930
F2: 1.000
SW1: 36058
OF1: 15080.8
PTS1d: 32768 , 32768
EX: zgpg30
PW: 10.0 us
PD: 2.0 sec
NA: 80
LB: 0.0
Nuts - $KL-1-31F_2

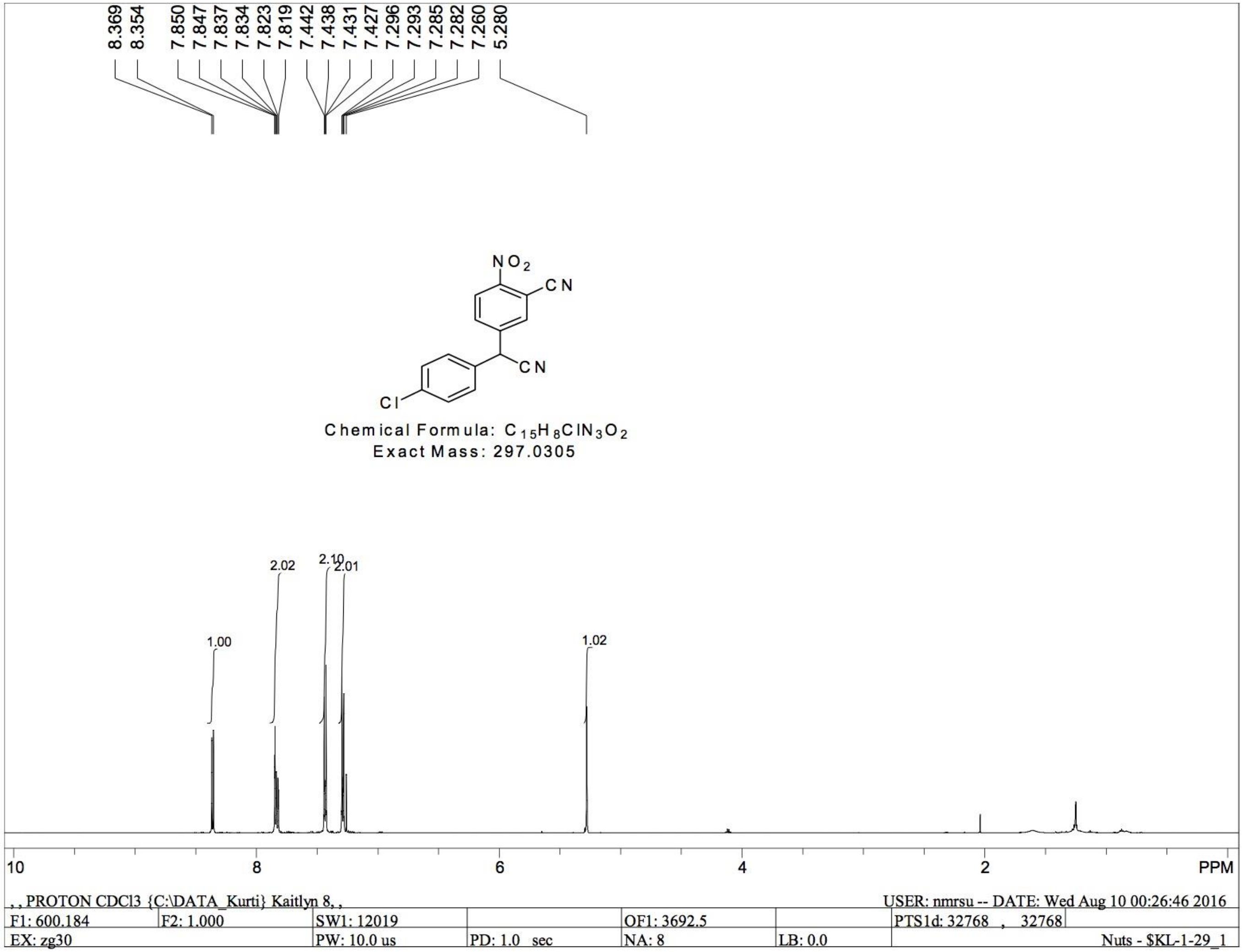
8.369
8.354
7.850
7.847
7.837
7.834
7.823
7.819
7.442
7.438
7.431
7.427
7.296
7.293
7.285
7.282
7.260
5.280
NO2
CN
CN
Cl
Chemical Formula: C15H8ClN3O2
Exact Mass: 297.0305
1.00
2.02
2.10
2.01
1.02
10
8
6
4
2
PPM
, , PROTON CDCl3 {C:\DATA_Kurti} Kaitlyn 8, ,
USER: nmrsu -- DATE: Wed Aug 10 00:26:46 2016
F1: 600.184
F2: 1.000
SW1: 12019
OF1: 3692.5
PTS1d: 32768 , 32768
EX: zg30
PW: 10.0 us
PD: 1.0 sec
NA: 8
LB: 0.0
Nuts - $KL-1-29_1

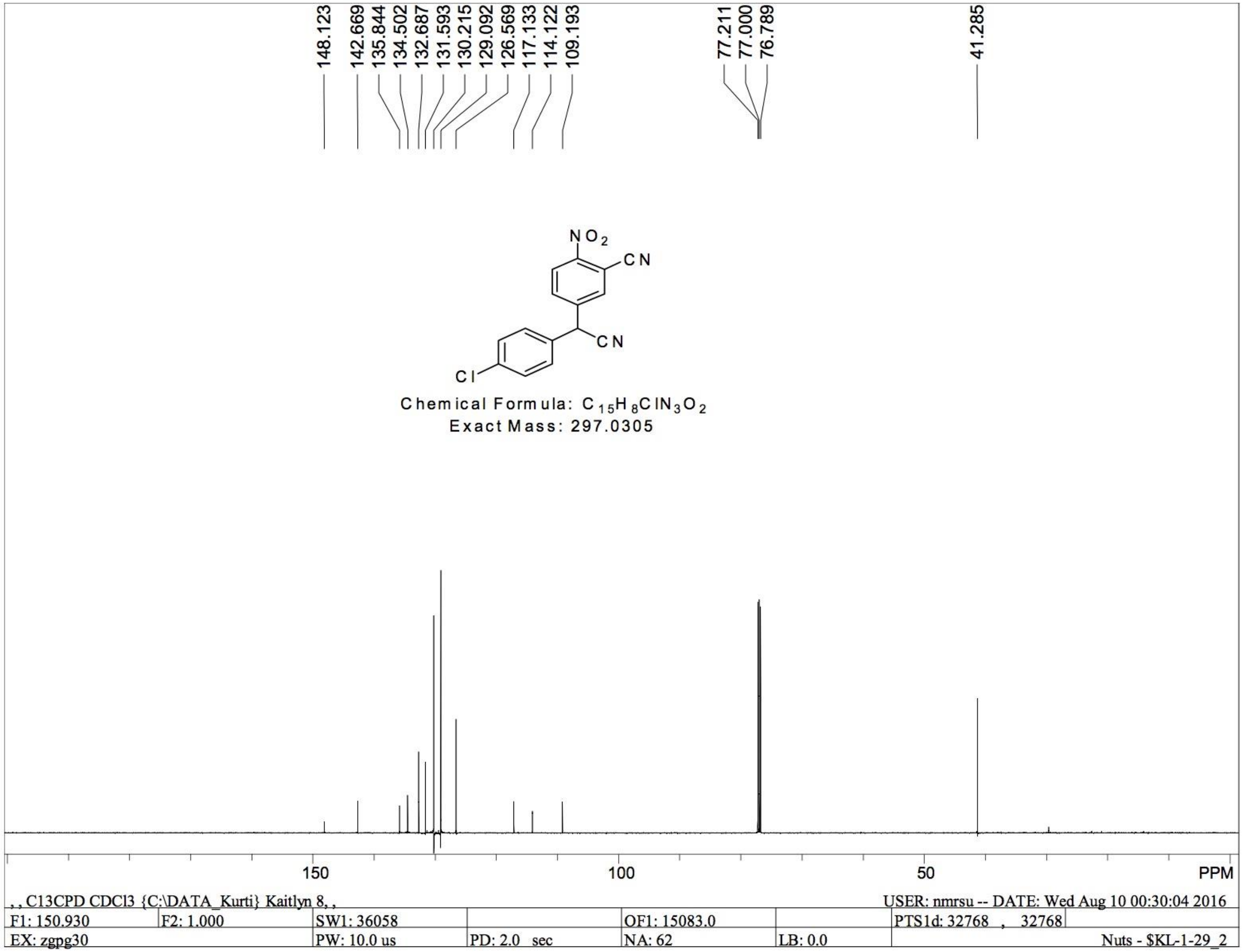
148.123
142.669
135.844
134.502
132.687
131.593
130.215
129.092
126.569
117.133
114.122
109.193
77.211
77.000
76.789
41.285
NO2
CN
CN
Cl
Chemical Formula: C15H8ClN3O2
Exact Mass: 297.0305
150
100
50
PPM
, , C13CPD CDCl3 {C:\DATA_Kurti} Kaitlyn 8, ,
USER: nmrsu -- DATE: Wed Aug 10 00:30:04 2016
F1: 150.930
F2: 1.000
SW1: 36058
OF1: 15083.0
PTS1d: 32768 , 32768
EX: zgpg30
PW: 10.0 us
PD: 2.0 sec
NA: 62
LB: 0.0
Nuts - $KL-1-29_2

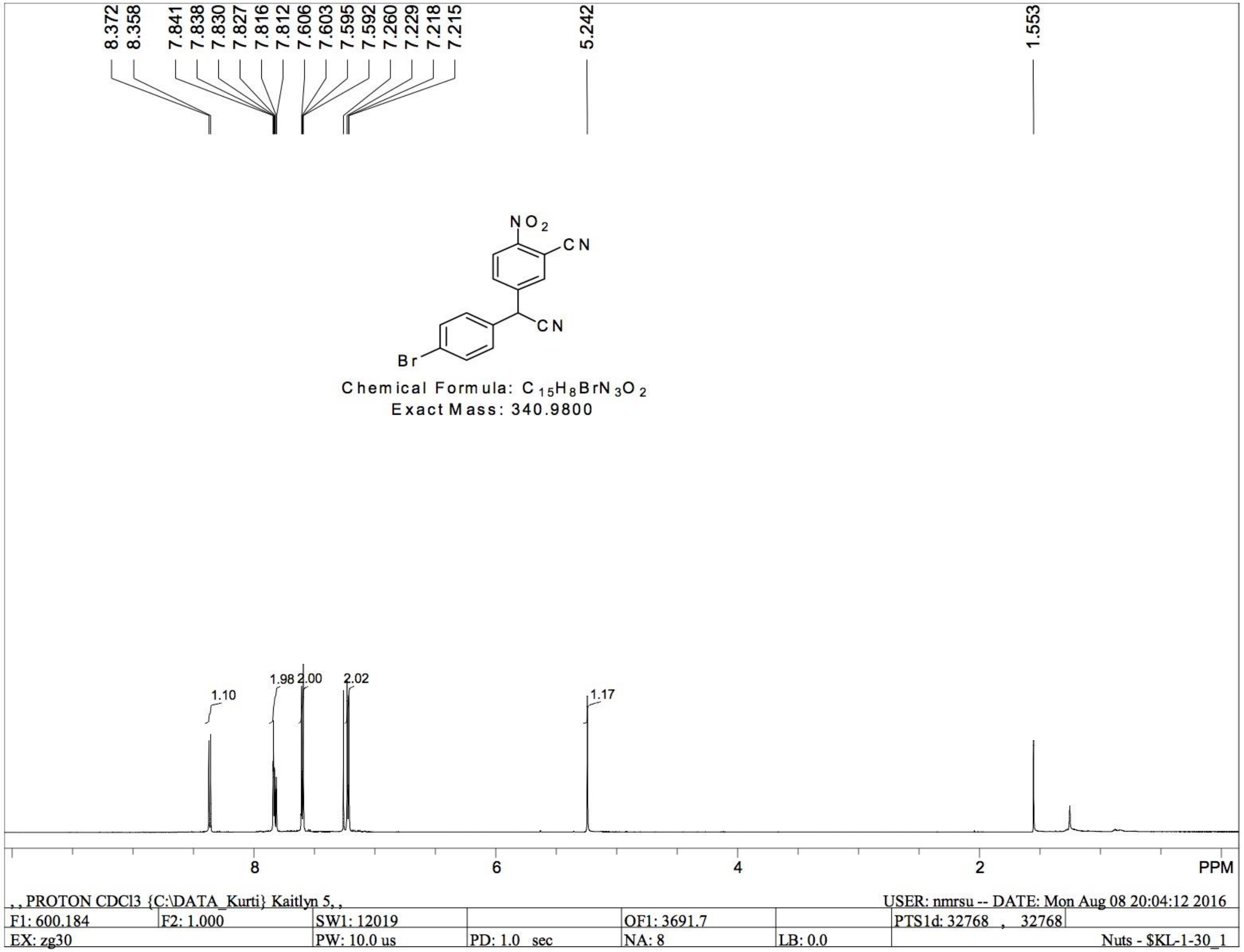
8.372
8.358
7.841
7.838
7.830
7.827
7.816
7.812
7.606
7.603
7.595
7.592
7.260
7.229
7.218
7.215
5.242
1.553
NO2
CN
CN
Br
Chemical Formula: C15H8BrN3O2
Exact Mass: 340.9800
1.10
1.98
2.00
2.02
1.17
8
6
4
2
PPM
, , PROTON CDCl3 {C:\DATA_Kurti} Kaitlyn 5, ,
USER: nmrsu -- DATE: Mon Aug 08 20:04:12 2016
F1: 600.184
F2: 1.000
SW1: 12019
OF1: 3691.7
PTS1d: 32768 , 32768
EX: zg30
PW: 10.0 us
PD: 1.0 sec
NA: 8
LB: 0.0
Nuts - $KL-1-30_1

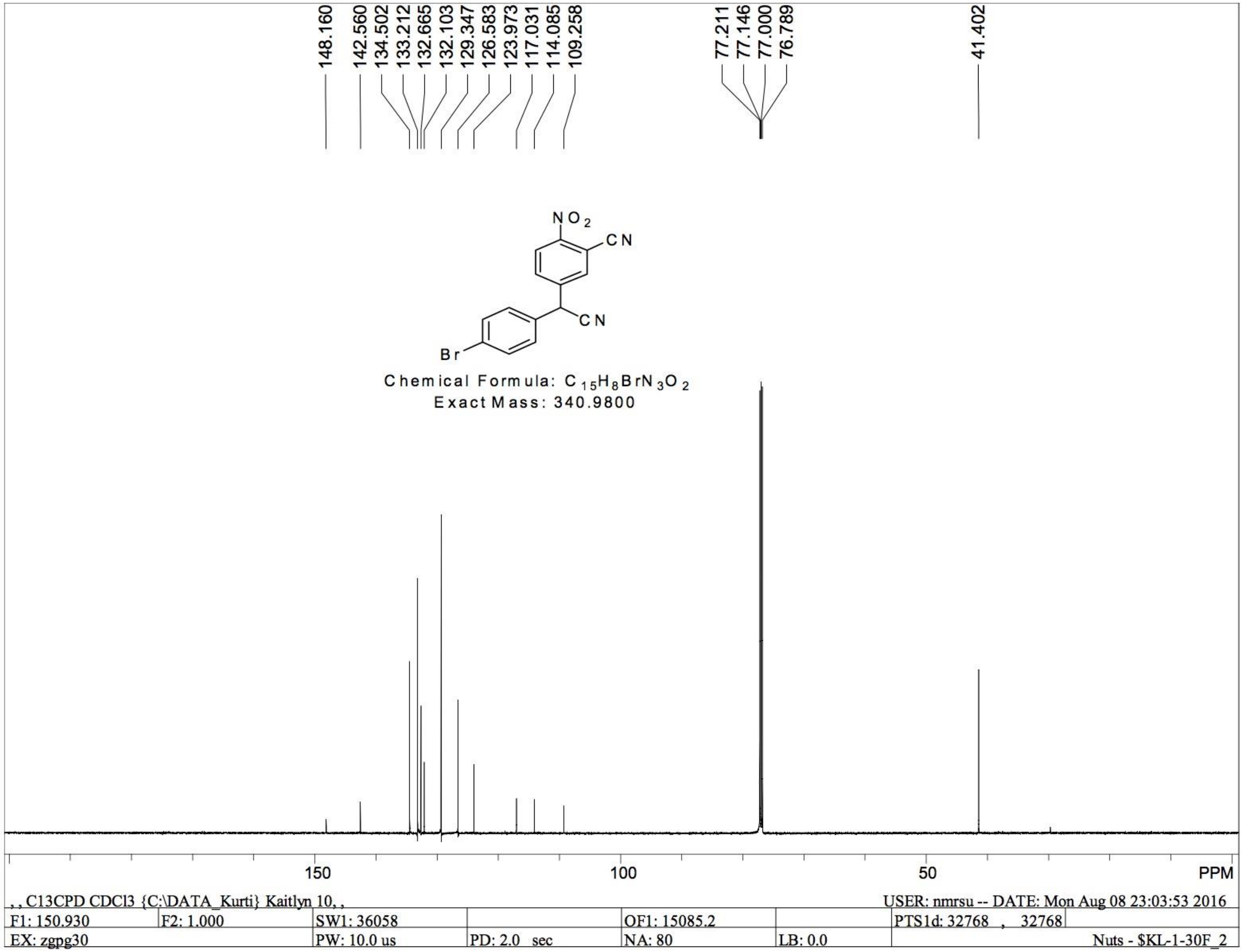
148.160
142.560
134.502
133.212
132.665
132.103
129.347
126.583
123.973
117.031
114.085
109.258
77.211
77.146
77.000
76.789
41.402
NO2
CN
CN
Br
Chemical Formula: C15H8BrN3O2
Exact Mass: 340.9800
150
100
50
PPM
, , C13CPD CDCl3 {C:\DATA_Kurti} Kaitlyn 10, ,
USER: nmrsu -- DATE: Mon Aug 08 23:03:53 2016
F1: 150.930
F2: 1.000
SW1: 36058
OF1: 15085.2
PTS1d: 32768 , 32768
EX: zgpg30
PW: 10.0 us
PD: 2.0 sec
NA: 80
LB: 0.0
Nuts - $KL-1-30F_2

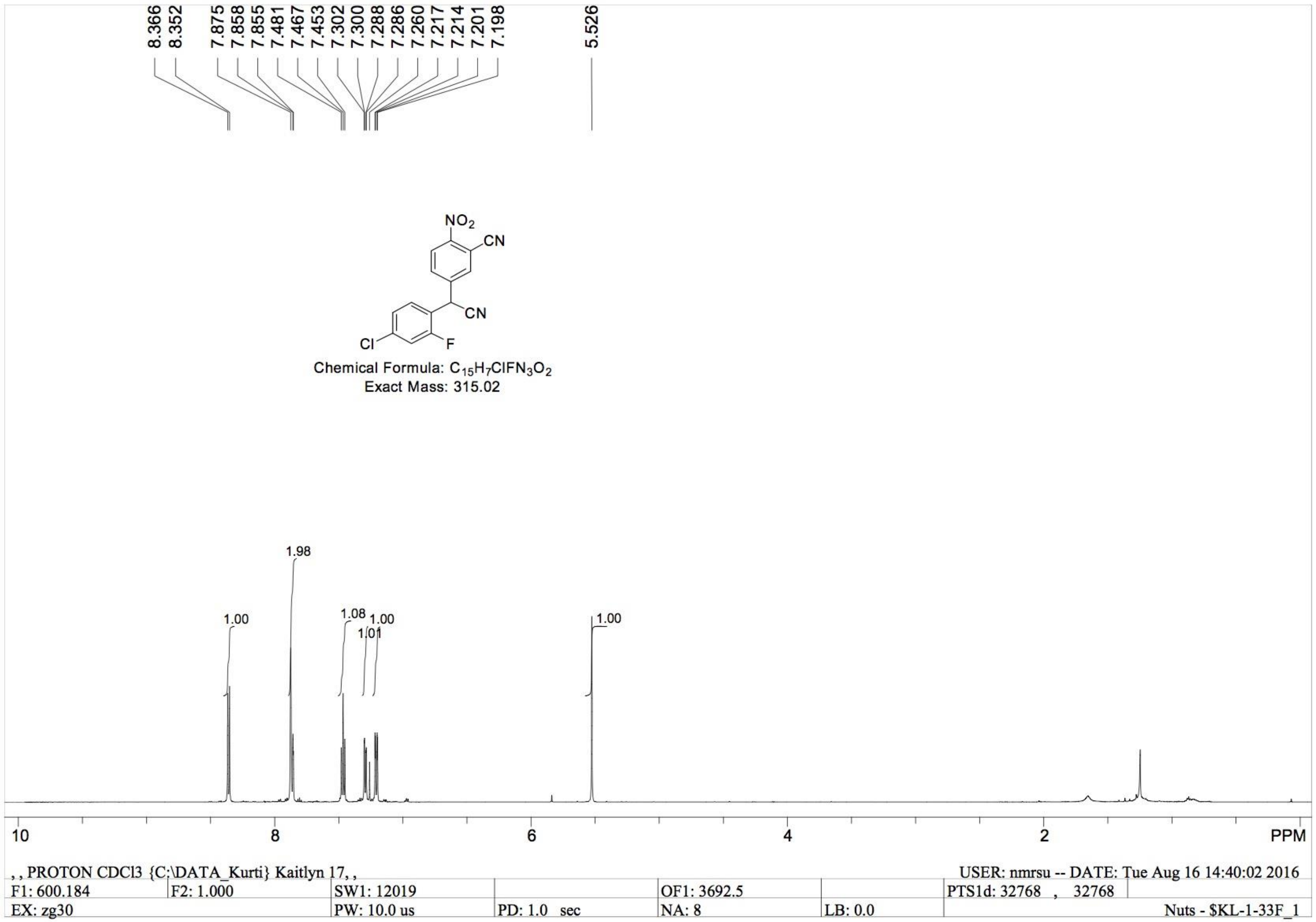

8.366
8.352
7.875
7.858
7.855
7.481
7.467
7.453
7.302
7.300
7.288
7.286
7.260
7.217
7.214
7.201
7.198
5.526
NO2
CN
CN
Cl
F
Chemical Formula: C15H7ClFN3O2
Exact Mass: 315.02
1.98
1.00
1.08
1.00
1.01
1.00
10
8
6
4
2
PPM
, , PROTON CDCl3 {C:\DATA_Kurti} Kaitlyn 17, ,
USER: nmrsu -- DATE: Tue Aug 16 14:40:02 2016
F1: 600.184
F2: 1.000
SW1: 12019
OF1: 3692.5
PTS1d: 32768 , 32768
EX: zg30
PW: 10.0 us
PD: 1.0 sec
NA: 8
LB: 0.0
Nuts - $KL-1-33F_1

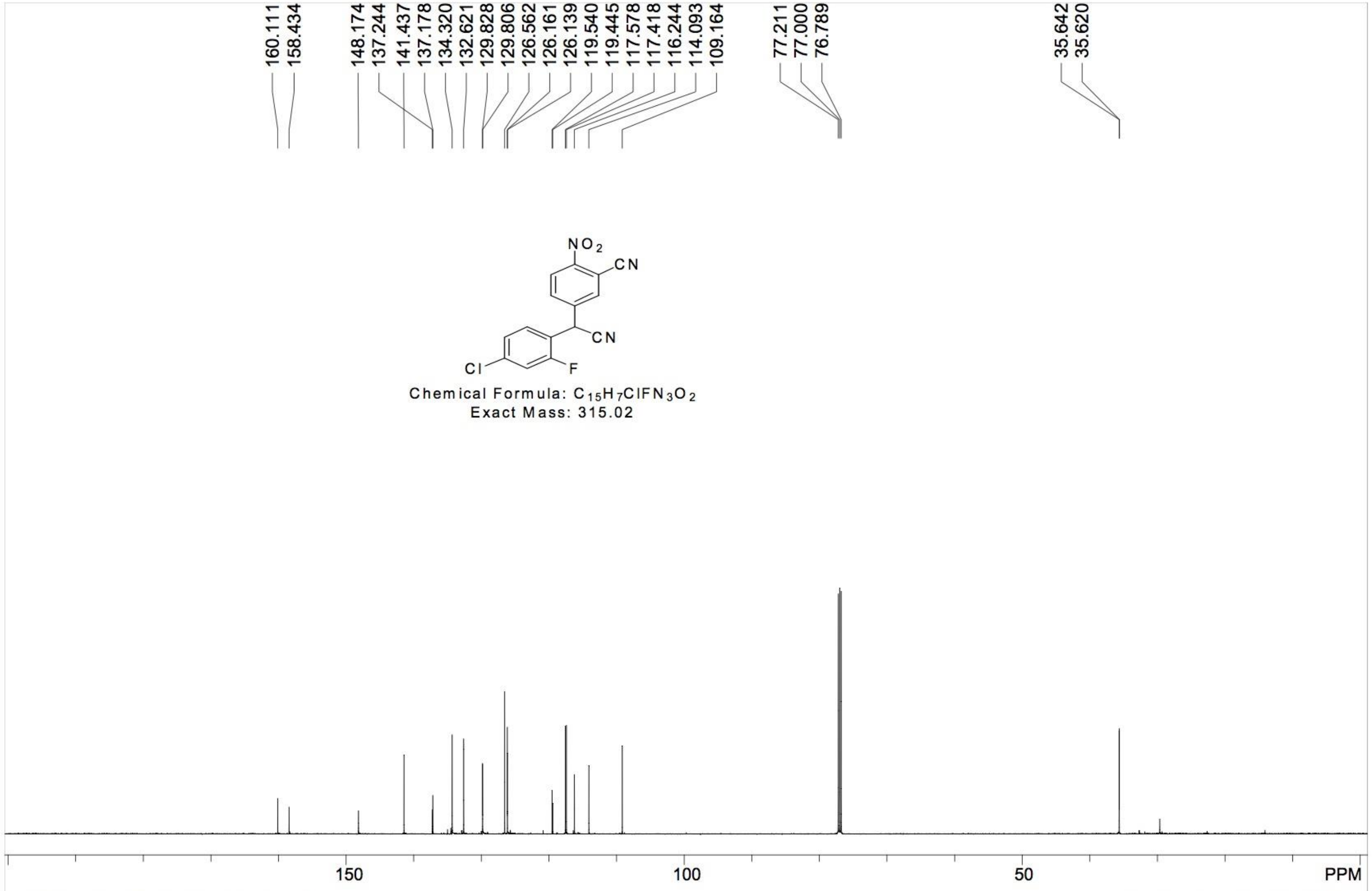

, , C13CPD CDCl3 {C:\DATA_Kurti} Kaitlyn 17, , USER: nmrsu -- DATE: Tue Aug 16 14:43:46 2016

F1: 150.930	F2: 1.000	SW1: 36058		OF1: 15079.7		PTS1d: 32768 , 32768	
EX: zgpg30		PW: 10.0 us	PD: 2.0 sec	NA: 80	LB: 0.2		Nuts - $KL-1-33F_2

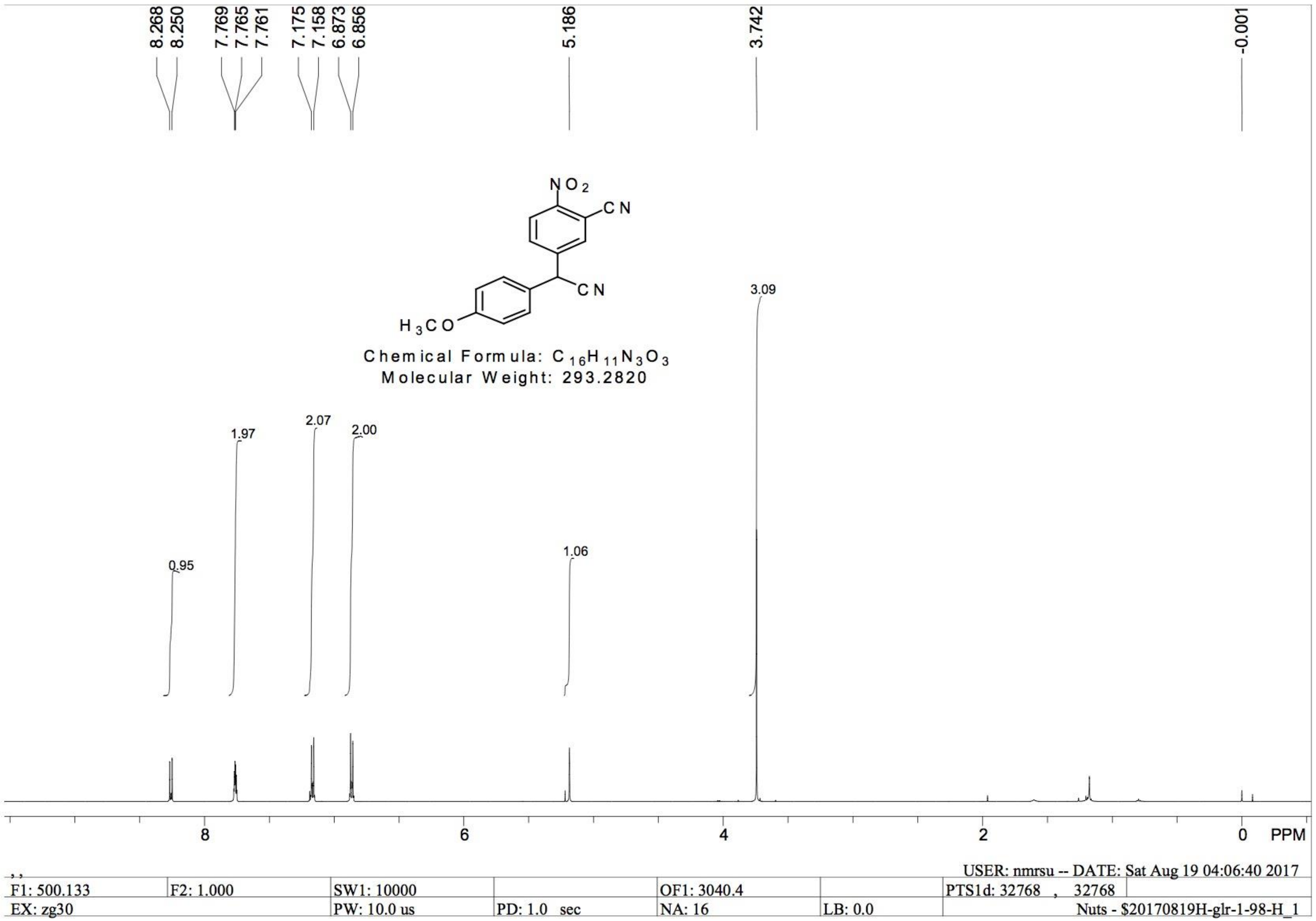
8.268
8.250
7.769
7.765
7.761
7.175
7.158
6.873
6.856
5.186
3.742
-0.001
NO2
CN
CN
H3CO
Chemical Formula: C16H11N3O3
Molecular Weight: 293.2820
3.09
1.97
2.07
2.00
0.95
1.06
8
6
4
2
0
PPM
USER: nmrsu -- DATE: Sat Aug 19 04:06:40 2017
F1: 500.133
F2: 1.000
SW1: 10000
OF1: 3040.4
PTS1d: 32768 , 32768
EX: zg30
PW: 10.0 us
PD: 1.0 sec
NA: 16
LB: 0.0
Nuts - $20170819H-glr-1-98-H_1

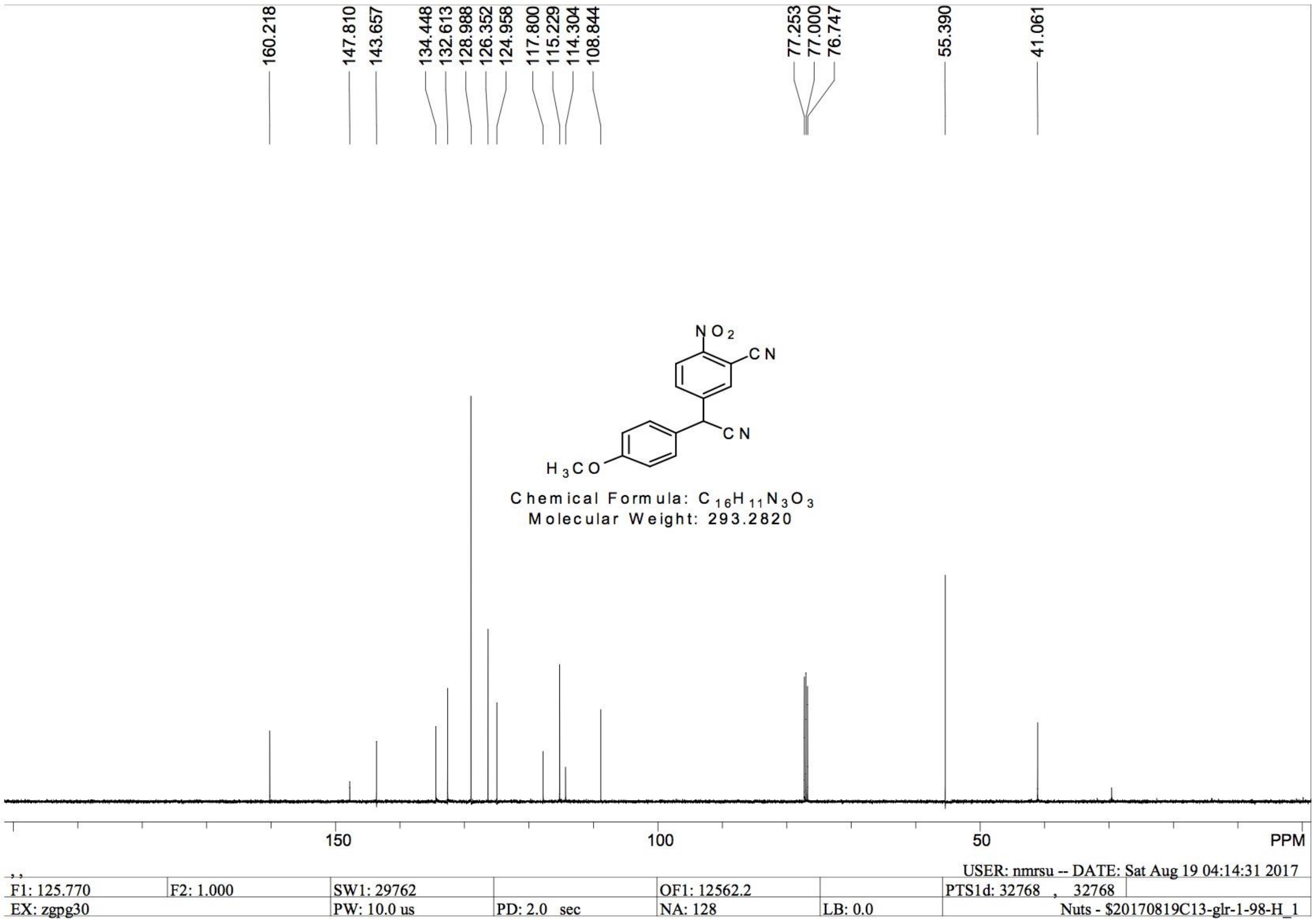
160.218
147.810
143.657
134.448
132.613
128.988
126.352
124.958
117.800
115.229
114.304
108.844
77.253
77.000
76.747
55.390
41.061
NO2
CN
CN
H3CO
Chemical Formula: C16H11N3O3
Molecular Weight: 293.2820
150
100
50
PPM
USER: nmrsu -- DATE: Sat Aug 19 04:14:31 2017
F1: 125.770
F2: 1.000
SW1: 29762
OF1: 12562.2
PTS1d: 32768 , 32768
EX: zgpg30
PW: 10.0 us
PD: 2.0 sec
NA: 128
LB: 0.0
Nuts - $20170819C13-glr-1-98-H_1

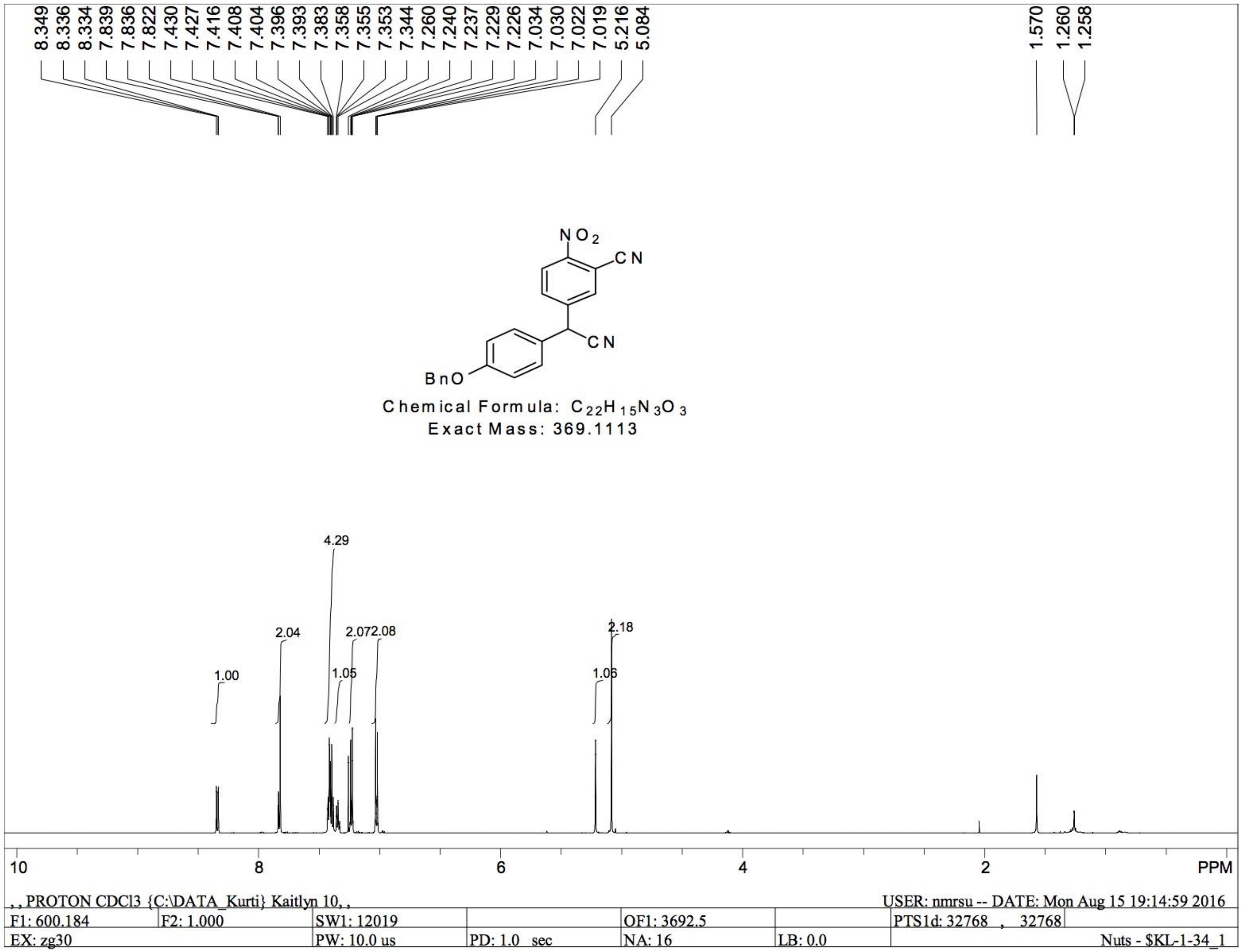
8.349 8.336 8.334 7.839 7.836 7.822 7.430 7.427 7.416 7.408 7.404 7.396 7.393 7.383 7.358 7.355 7.353 7.344 7.260 7.240 7.237 7.229 7.226 7.034 7.030 7.022 7.019 5.216 5.084
1.570 1.260 1.258
NO2
CN
CN
BnO
Chemical Formula: C22H15N3O3
Exact Mass: 369.1113
4.29
2.04
2.072.08
2.18
1.00
1.05
1.06
10
8
6
4
2
PPM
, , PROTON CDCl3 {C:\DATA_Kurti} Kaitlyn 10, ,
USER: nmrsu -- DATE: Mon Aug 15 19:14:59 2016
F1: 600.184
F2: 1.000
SW1: 12019
OF1: 3692.5
PTS1d: 32768 , 32768
EX: zg30
PW: 10.0 us
PD: 1.0 sec
NA: 16
LB: 0.0
Nuts - $KL-1-34_1

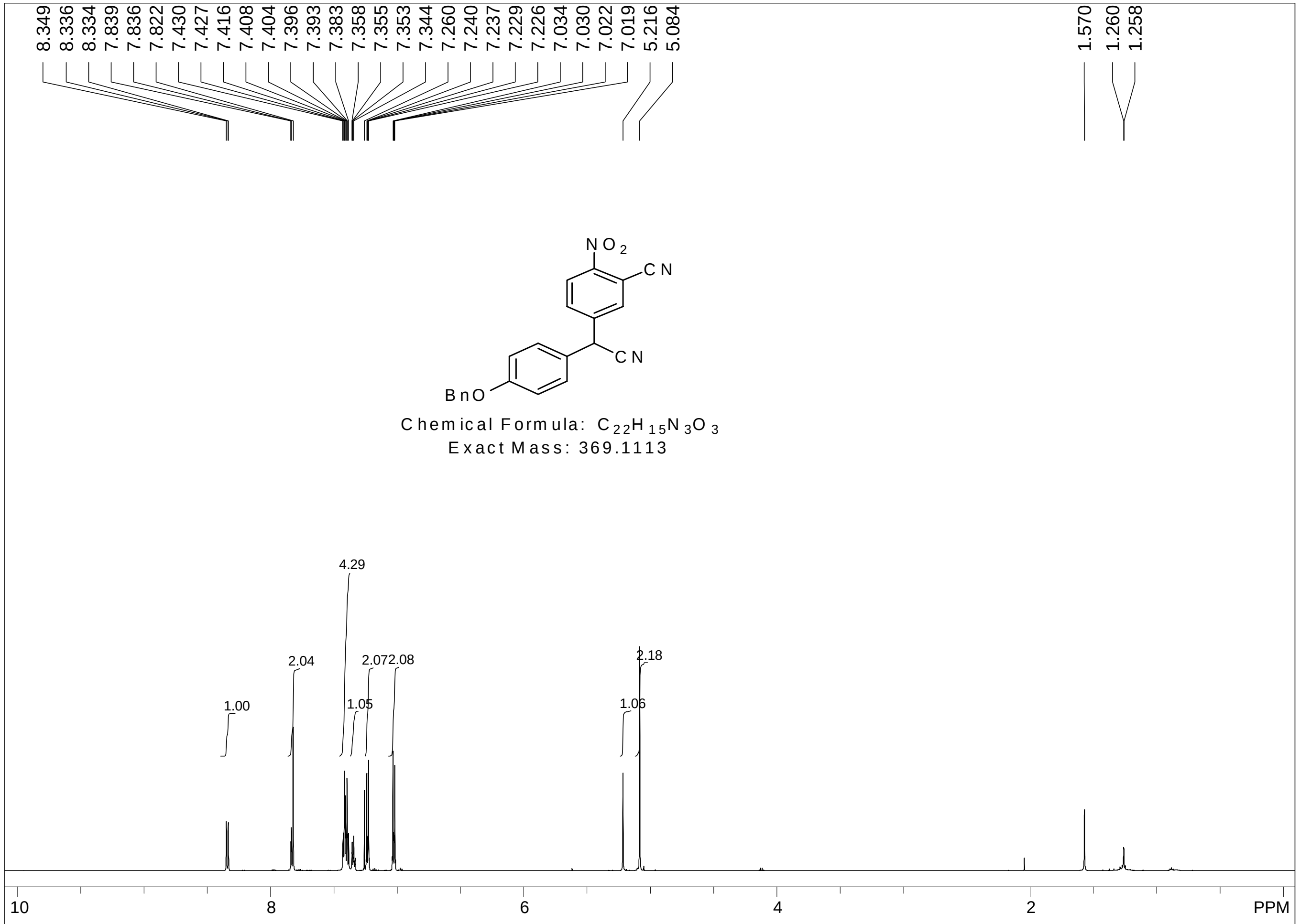

, , PROTON CDCl3 {C:\DATA_Kurti} Kaitlyn 10, , USER: nmrsu -- DATE: Mon Aug 15 19:14:59 2016

F1: 600.184	F2: 1.000	SW1: 12019		OF1: 3692.5		PTS1d: 32768 , 32768	
EX: zg30		PW: 10.0 us	PD: 1.0 sec	NA: 16	LB: 0.0		Nuts - $KL-1-34_1

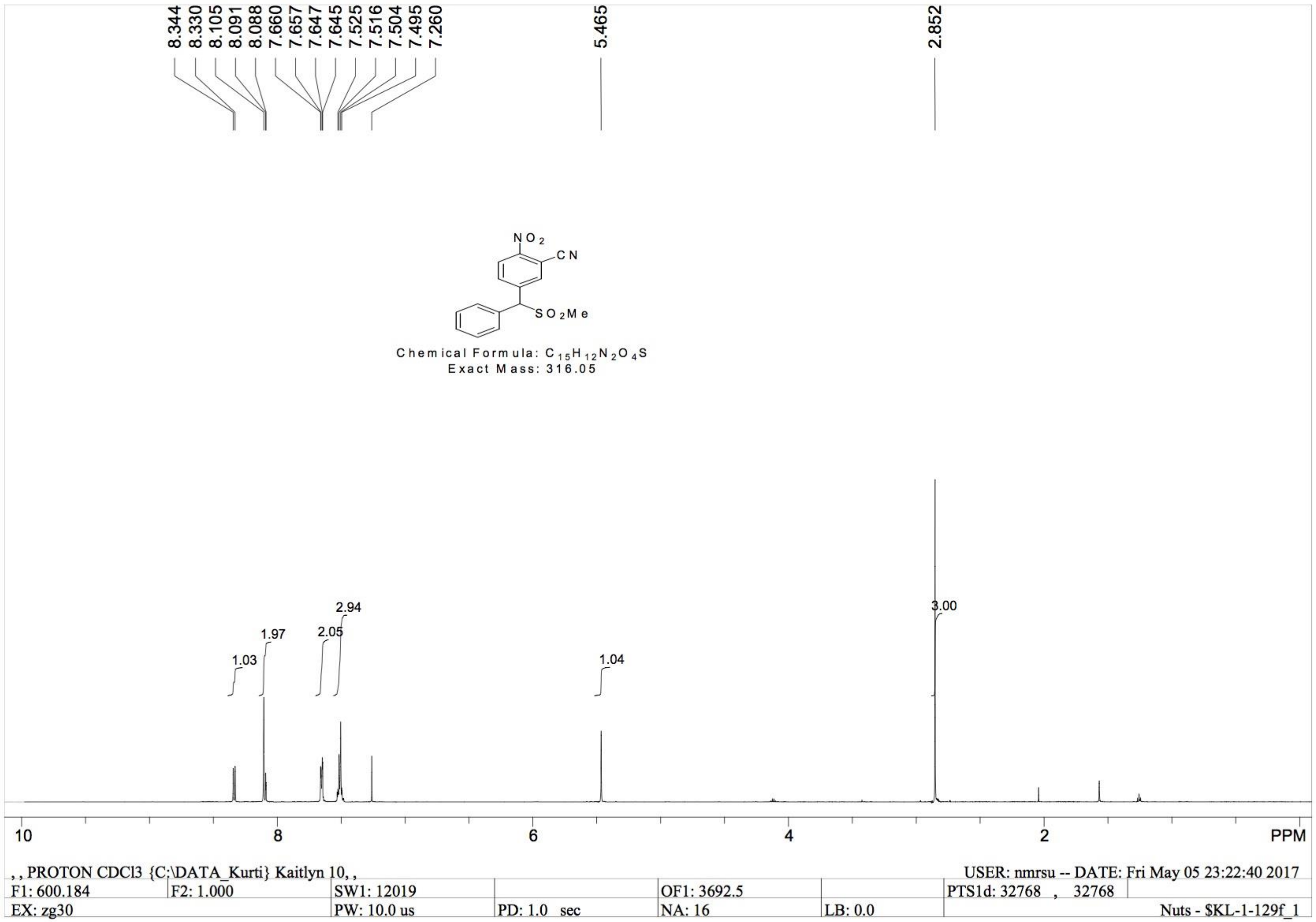
8.344
8.330
8.105
8.091
8.088
7.660
7.657
7.647
7.645
7.525
7.516
7.504
7.495
7.260
5.465
2.852
NO2
CN
SO2Me
Chemical Formula: C15H12N2O4S
Exact Mass: 316.05
1.03
1.97
2.05
2.94
1.04
3.00
10
8
6
4
2
PPM
, , PROTON CDCl3 {C:\DATA_Kurti} Kaitlyn 10, ,
USER: nmrsu -- DATE: Fri May 05 23:22:40 2017
F1: 600.184
F2: 1.000
SW1: 12019
OF1: 3692.5
PTS1d: 32768 , 32768
EX: zg30
PW: 10.0 us
PD: 1.0 sec
NA: 16
LB: 0.0
Nuts - $KL-1-129f_1

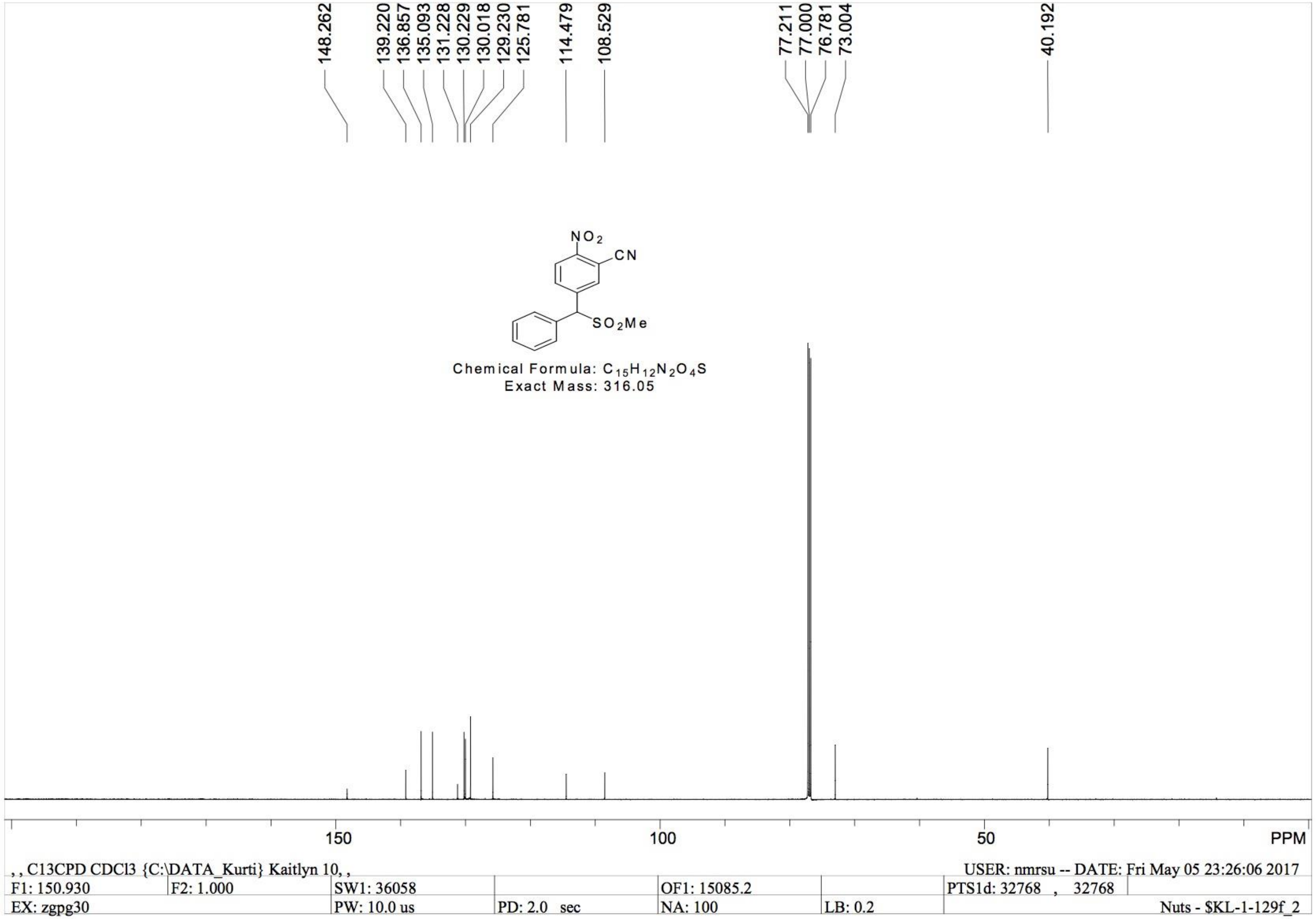

148.262
139.220
136.857
135.093
131.228
130.229
130.018
129.230
125.781
114.479
108.529
77.211
77.000
76.781
73.004
40.192
NO2
CN
SO2Me
Chemical Formula: C15H12N2O4S
Exact Mass: 316.05
150
100
50
PPM
, , C13CPD CDCl3 {C:\DATA_Kurti} Kaitlyn 10, ,
USER: nmrsu -- DATE: Fri May 05 23:26:06 2017
F1: 150.930
F2: 1.000
SW1: 36058
OF1: 15085.2
PTS1d: 32768 , 32768
EX: zgpg30
PW: 10.0 us
PD: 2.0 sec
NA: 100
LB: 0.2
Nuts - $KL-1-129f_2

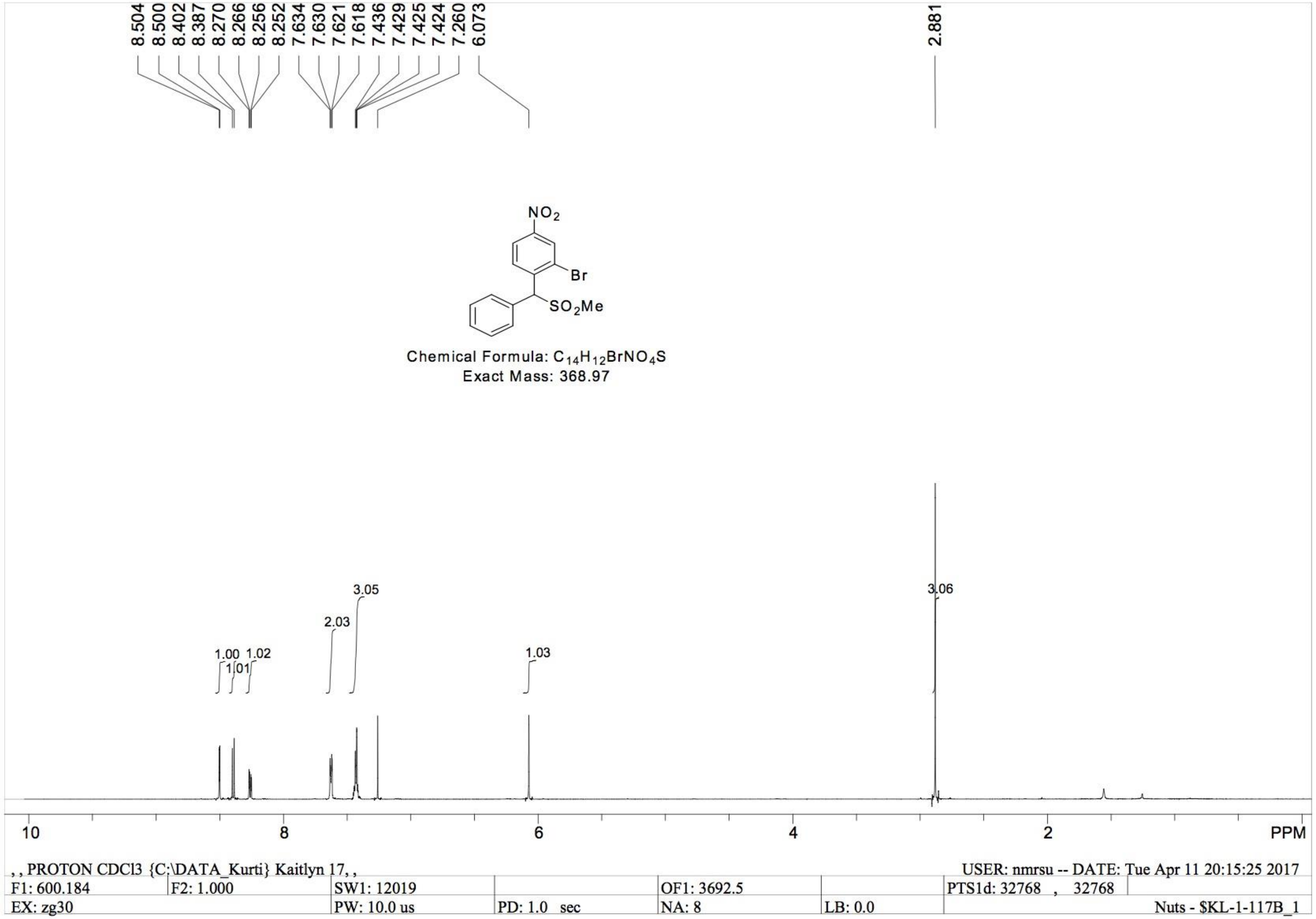

8.504
8.500
8.402
8.387
8.270
8.266
8.256
8.252
7.634
7.630
7.621
7.618
7.436
7.429
7.425
7.424
7.260
6.073
2.881
NO2
Br
SO2Me
Chemical Formula: C14H12BrNO4S
Exact Mass: 368.97
1.00
1.01
1.02
2.03
3.05
1.03
3.06
10
8
6
4
2
PPM
, , PROTON CDCl3 {C:\DATA_Kurti} Kaitlyn 17, ,
USER: nmrsu -- DATE: Tue Apr 11 20:15:25 2017
F1: 600.184
F2: 1.000
SW1: 12019
OF1: 3692.5
PTS1d: 32768 , 32768
EX: zg30
PW: 10.0 us
PD: 1.0 sec
NA: 8
LB: 0.0
Nuts - $KL-1-117B_1

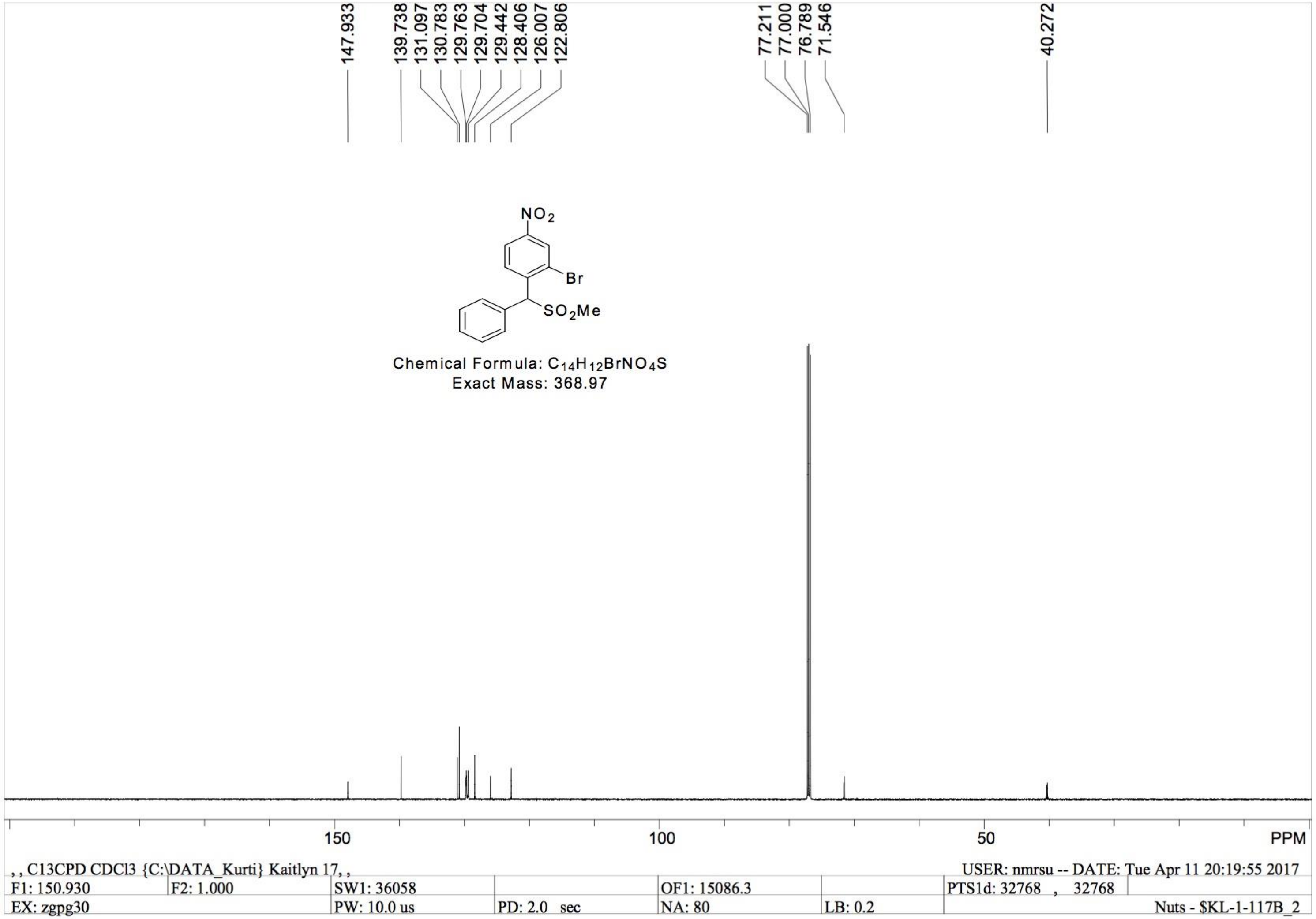
147.933
139.738
131.097
130.783
129.763
129.704
129.442
128.406
126.007
122.806
77.211
77.000
76.789
71.546
40.272
NO2
Br
SO2Me
Chemical Formula: C14H12BrNO4S
Exact Mass: 368.97
150
100
50
PPM
, , C13CPD CDCl3 {C:\DATA_Kurti} Kaitlyn 17, ,
USER: nmrsu -- DATE: Tue Apr 11 20:19:55 2017
F1: 150.930
F2: 1.000
SW1: 36058
OF1: 15086.3
PTS1d: 32768 , 32768
EX: zgpg30
PW: 10.0 us
PD: 2.0 sec
NA: 80
LB: 0.2
Nuts - $KL-1-117B_2

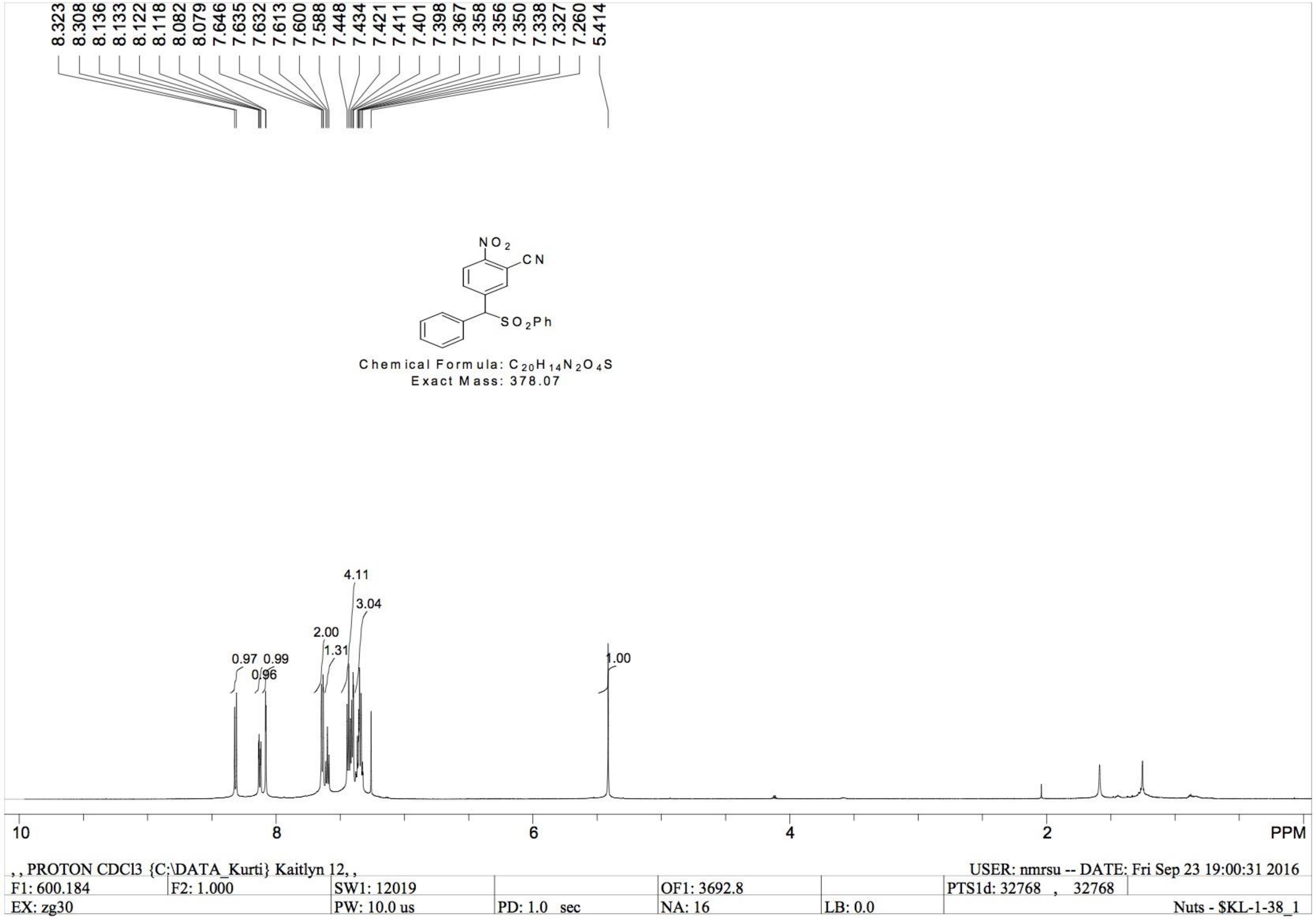
8.323 8.308 8.136 8.133 8.122 8.118 8.082 8.079 7.646 7.635 7.632 7.613 7.600 7.588 7.448 7.434 7.421 7.411 7.401 7.398 7.367 7.358 7.356 7.350 7.338 7.327 7.260 5.414
NO2
CN
SO2Ph
Chemical Formula: C20H14N2O4S
Exact Mass: 378.07
4.11
3.04
2.00
1.31
0.97 0.99
0.96
1.00
10
8
6
4
2
PPM
, , PROTON CDCl3 {C:\DATA_Kurti} Kaitlyn 12, ,
USER: nmrsu -- DATE: Fri Sep 23 19:00:31 2016
F1: 600.184
F2: 1.000
SW1: 12019
OF1: 3692.8
PTS1d: 32768 , 32768
EX: zg30
PW: 10.0 us
PD: 1.0 sec
NA: 16
LB: 0.0
Nuts - $KL-1-38_1

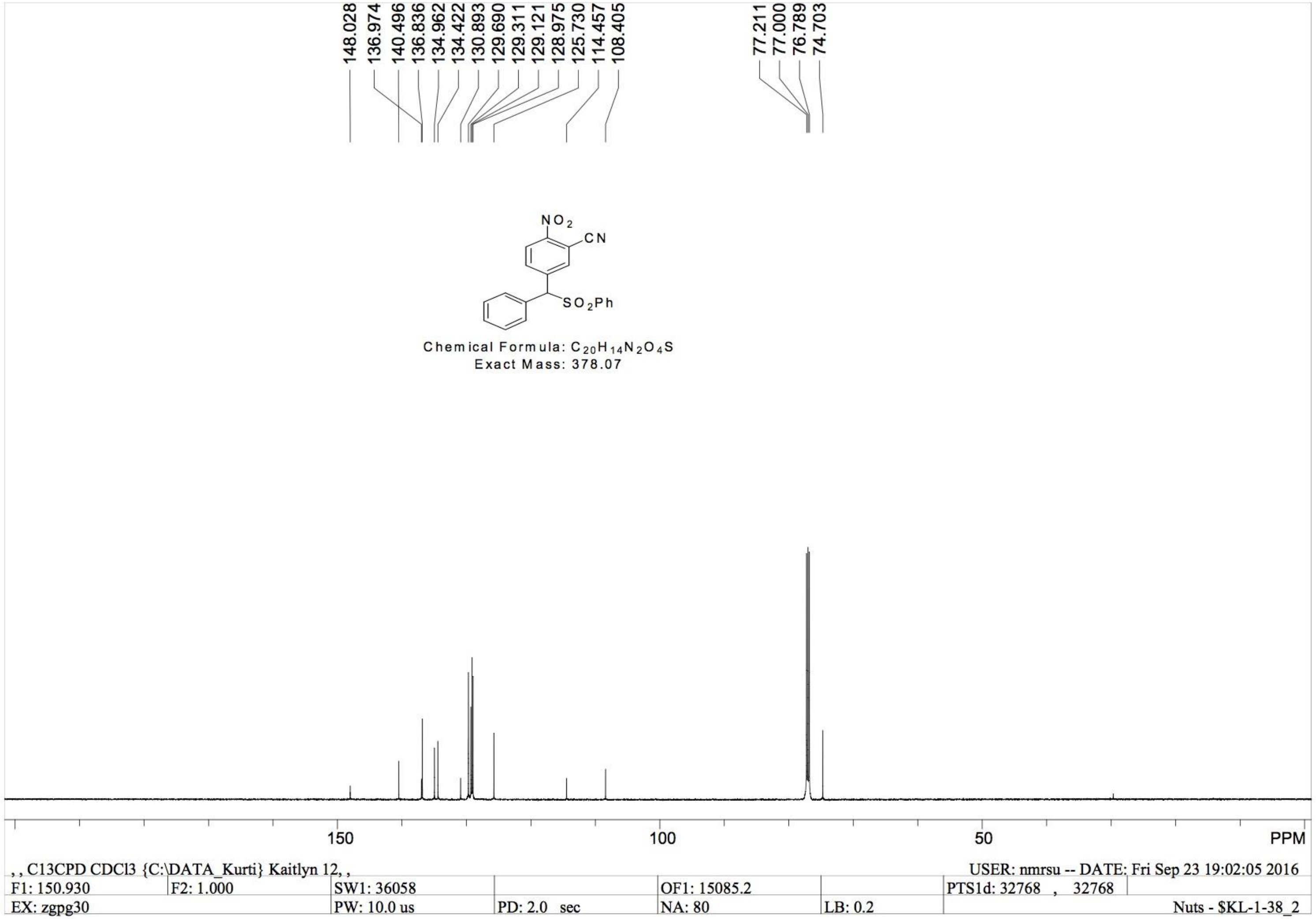
148.028
136.974
140.496
136.836
134.962
134.422
130.893
129.690
129.311
129.121
128.975
125.730
114.457
108.405
77.211
77.000
76.789
74.703
NO2
CN
SO2Ph
Chemical Formula: C20H14N2O4S
Exact Mass: 378.07
150
100
50
PPM
, , C13CPD CDCl3 {C:\DATA_Kurti} Kaitlyn 12, ,
USER: nmrsu -- DATE: Fri Sep 23 19:02:05 2016
F1: 150.930
F2: 1.000
SW1: 36058
OF1: 15085.2
PTS1d: 32768 , 32768
EX: zgpg30
PW: 10.0 us
PD: 2.0 sec
NA: 80
LB: 0.2
Nuts - $KL-1-38_2

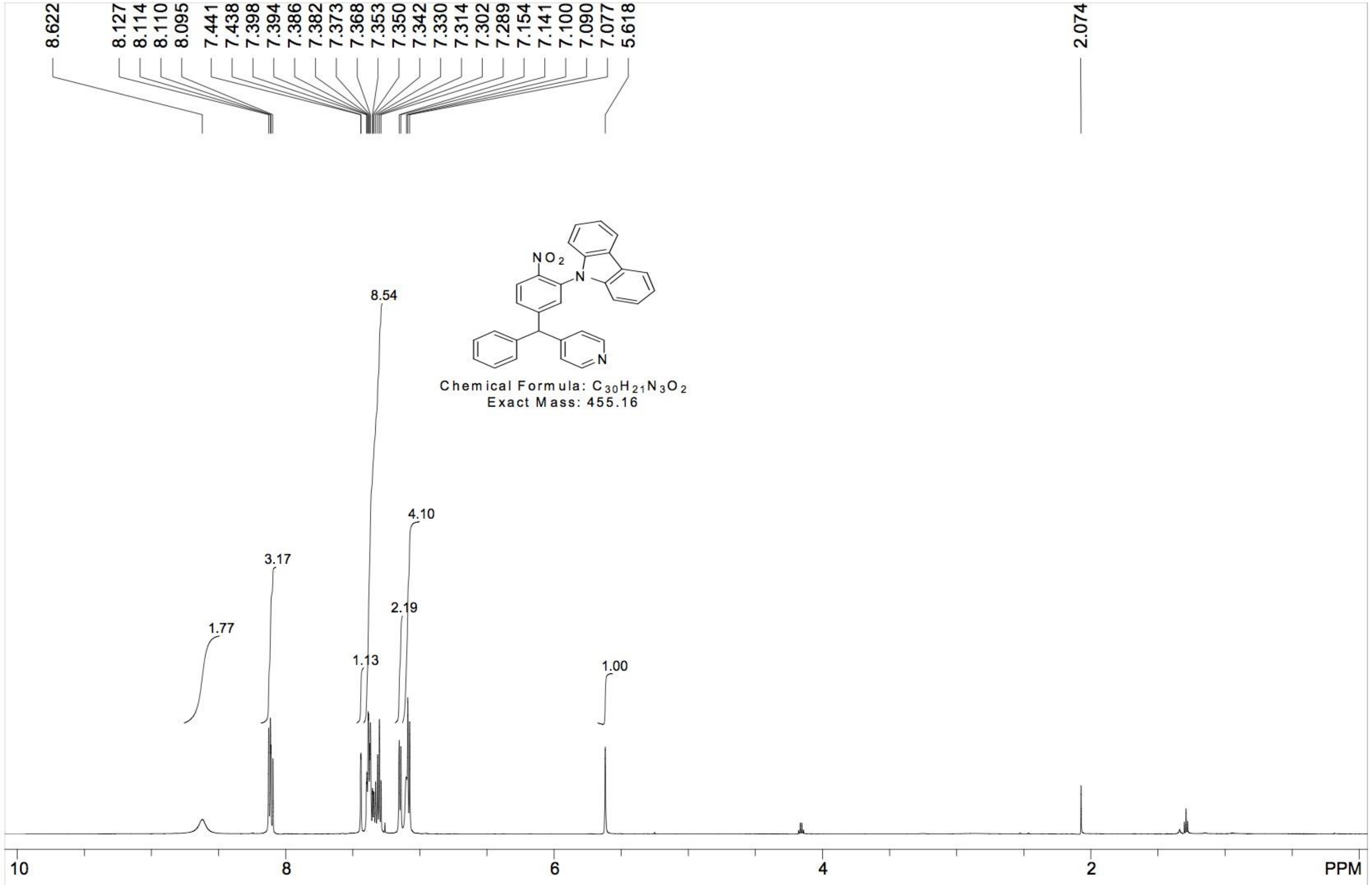

, , PROTON CDCl3 {C:\DATA_Kurti} Gao 21, , USER: nmrsu -- DATE: Wed Sep 28 20:08:23 2016

F1: 600.184	F2: 1.000	SW1: 12019		OF1: 3693.2		PTS1d: 32768 , 32768	
EX: zg30		PW: 10.0 us	PD: 1.0 sec	NA: 8	LB: 0.0		Nuts - $cab-1-7_1

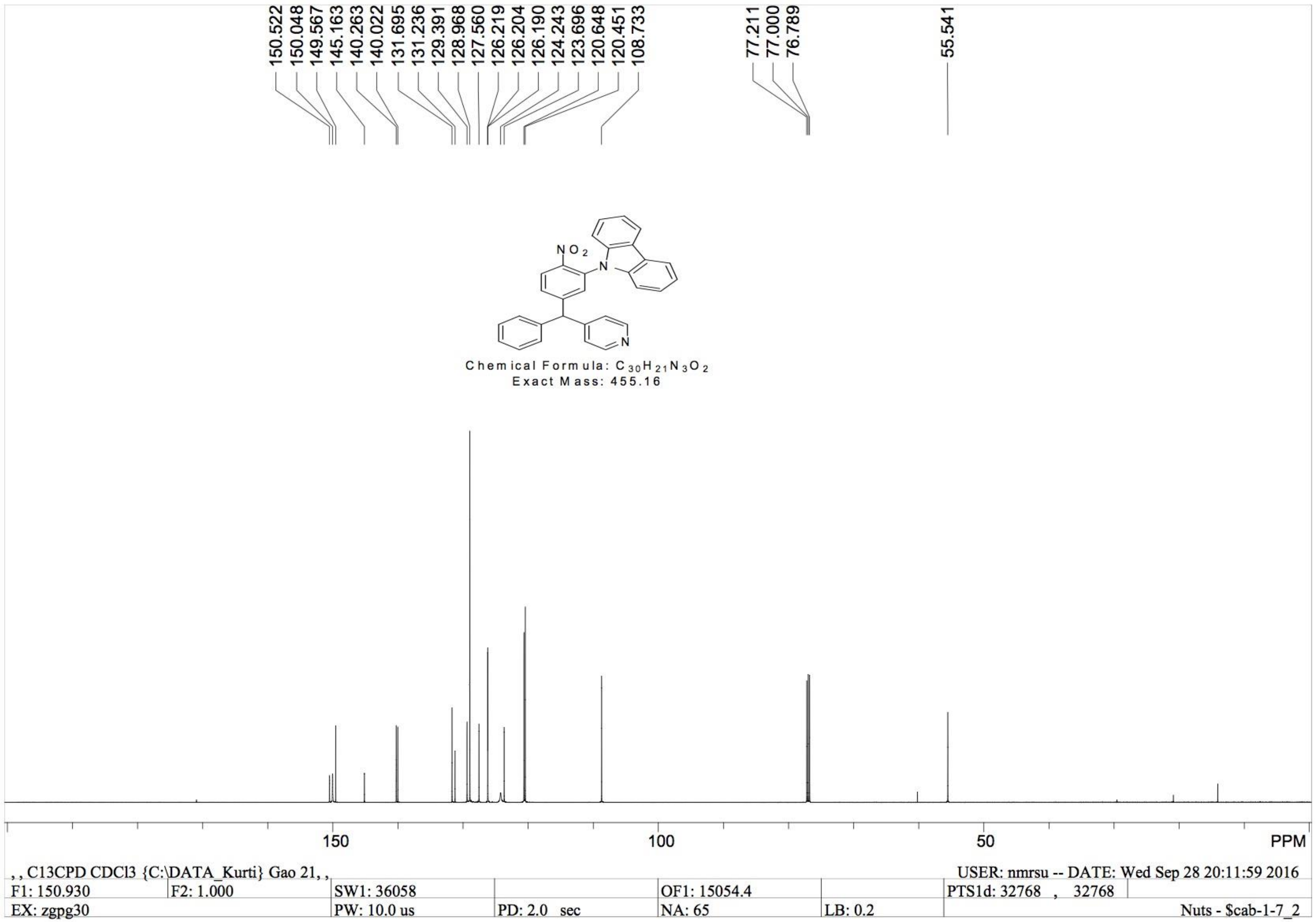
150.522
150.048
149.567
145.163
140.263
140.022
131.695
131.236
129.391
128.968
127.560
126.219
126.204
126.190
124.243
123.696
120.648
120.451
108.733
77.211
77.000
76.789
55.541
NO_2
N
N
Chemical Formula: $C_{30}H_{21}N_3O_2$
Exact Mass: 455.16
150
100
50
PPM
, , C13CPD CDCl3 {C:\DATA_Kurti} Gao 21, ,
USER: nmrsu -- DATE: Wed Sep 28 20:11:59 2016
F1: 150.930
F2: 1.000
SW1: 36058
OF1: 15054.4
PTS1d: 32768 , 32768
EX: zgpg30
PW: 10.0 us
PD: 2.0 sec
NA: 65
LB: 0.2
Nuts - $cab-1-7_2

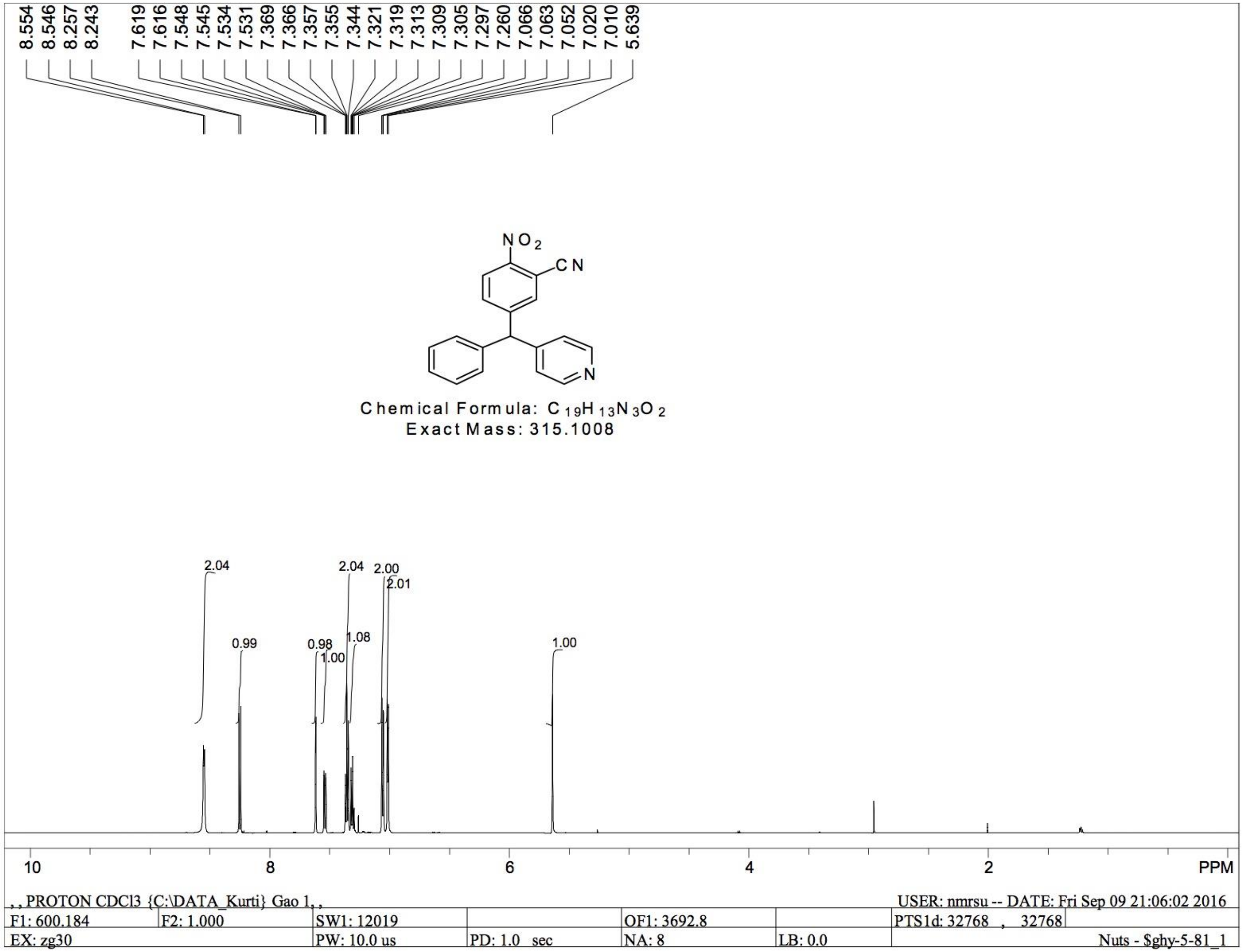
8.554
8.546
8.257
8.243
7.619
7.616
7.548
7.545
7.534
7.531
7.369
7.366
7.357
7.355
7.344
7.321
7.319
7.313
7.309
7.305
7.297
7.260
7.066
7.063
7.052
7.020
7.010
5.639
NO2
CN
N
Chemical Formula: C19H13N3O2
Exact Mass: 315.1008
2.04
0.99
0.98
1.00
2.04
1.08
2.00
2.01
1.00
10
8
6
4
2
PPM
, , PROTON CDCl3 {C:\DATA_Kurti} Gao 1, ,
USER: nmrsu -- DATE: Fri Sep 09 21:06:02 2016
F1: 600.184
F2: 1.000
SW1: 12019
OF1: 3692.8
PTS1d: 32768 , 32768
EX: zg30
PW: 10.0 us
PD: 1.0 sec
NA: 8
LB: 0.0
Nuts - $ghy-5-81_1

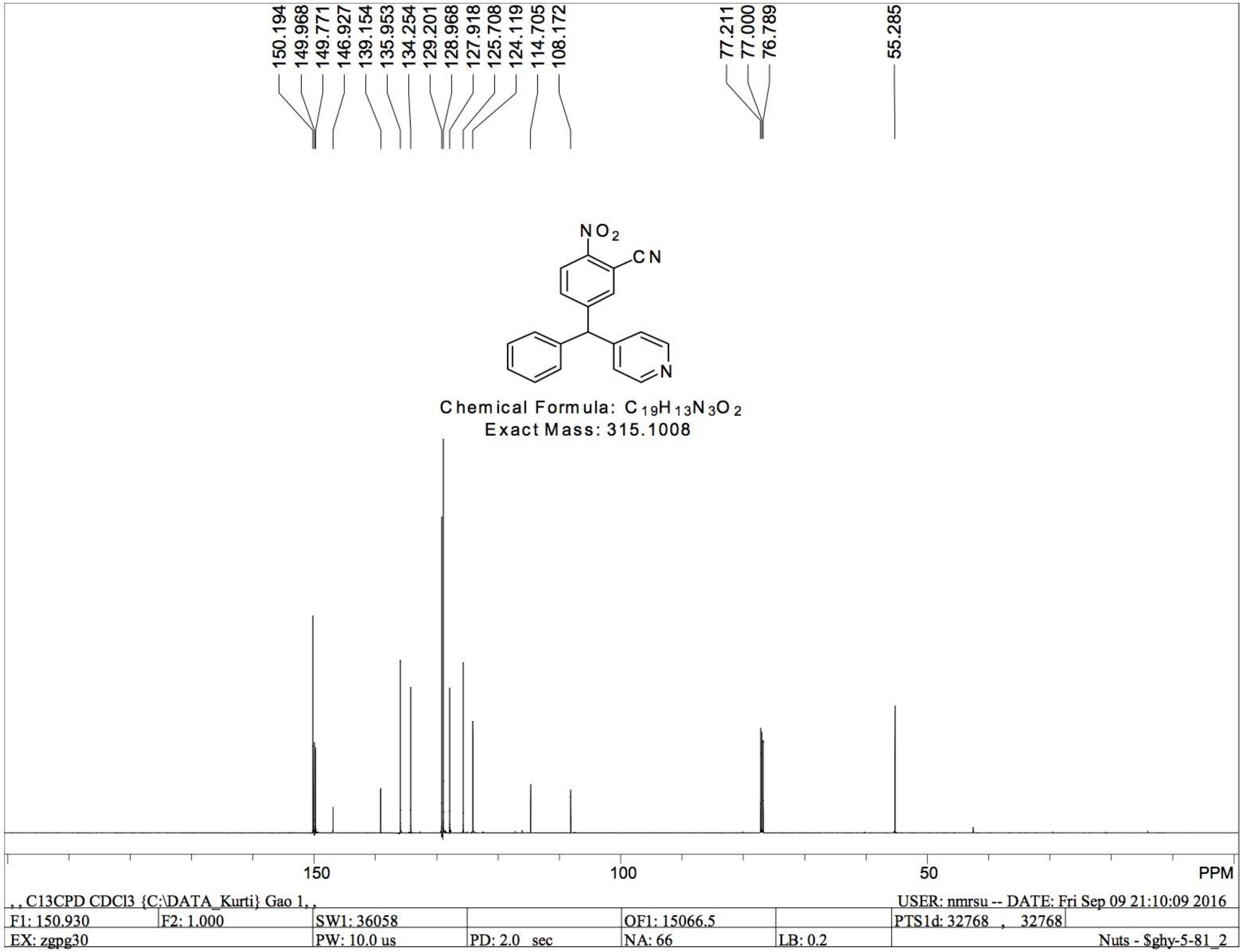
150.194
149.968
149.771
146.927
139.154
135.953
134.254
129.201
128.968
127.918
125.708
124.119
114.705
108.172
77.211
77.000
76.789
55.285
NO2
CN
N
Chemical Formula: C19H13N3O2
Exact Mass: 315.1008
150
100
50
PPM
, , C13CPD CDCl3 {C:\DATA_Kurti} Gao 1, ,
USER: nmrsu -- DATE: Fri Sep 09 21:10:09 2016
F1: 150.930
F2: 1.000
SW1: 36058
OF1: 15066.5
PTS1d: 32768 , 32768
EX: zgpg30
PW: 10.0 us
PD: 2.0 sec
NA: 66
LB: 0.2
Nuts - $ghy-5-81_2

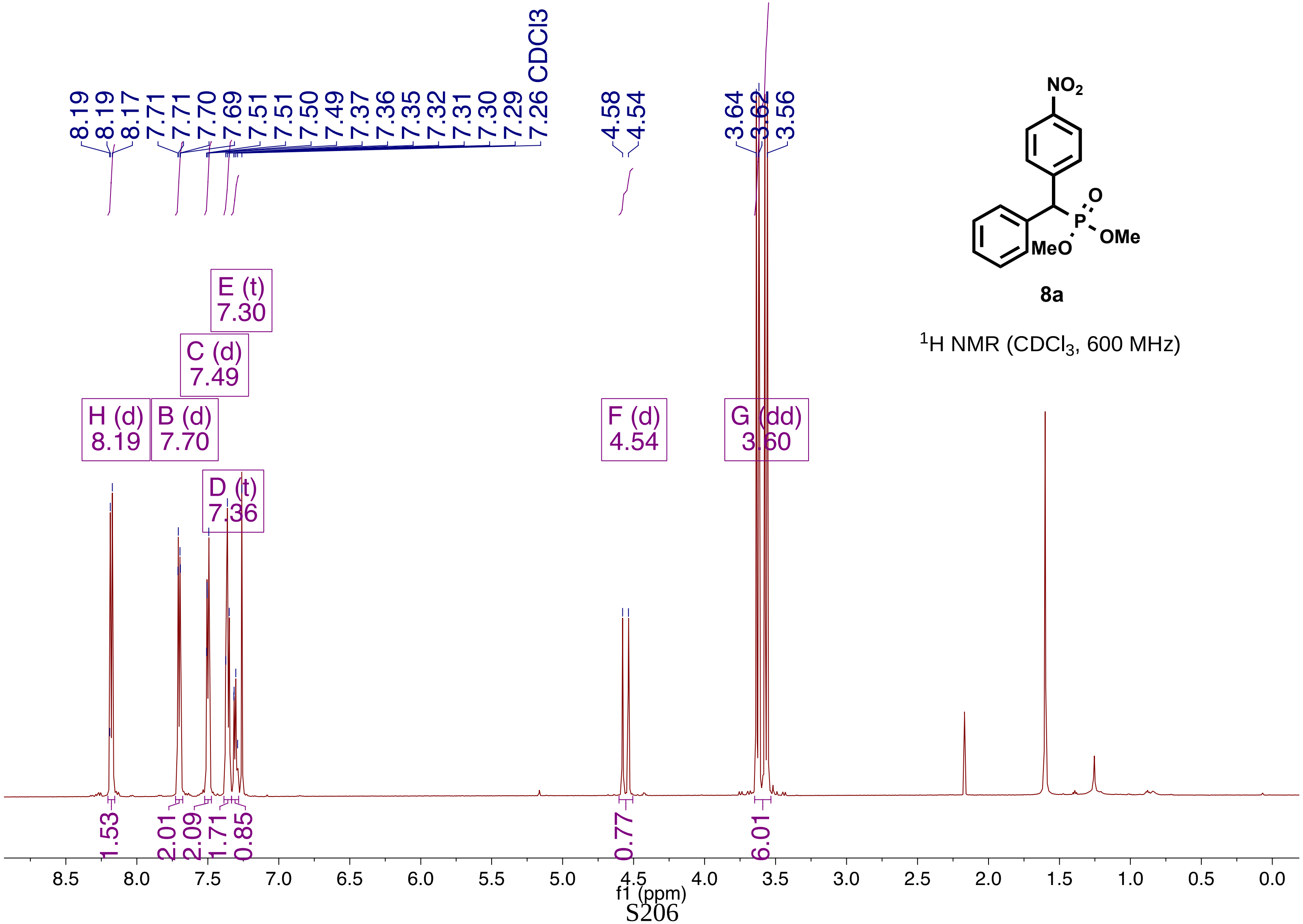
8.19
8.19
8.17
7.71
7.71
7.70
7.69
7.51
7.51
7.50
7.49
7.37
7.36
7.35
7.32
7.31
7.30
7.29
7.26 CDCl3
4.58
4.54
3.64
3.62
3.56
NO2
O
P
OMe
MeO
8a
1H NMR (CDCl3, 600 MHz)
E (t) 7.30
C (d) 7.49
H (d) 8.19
B (d) 7.70
D (t) 7.36
F (d) 4.54
G (dd) 3.60
1.53
2.01
2.09
1.71
0.85
0.77
6.01
8.5
8.0
7.5
7.0
6.5
6.0
5.5
5.0
4.5
4.0
3.5
3.0
2.5
2.0
1.5
1.0
0.5
0.0
f1 (ppm)

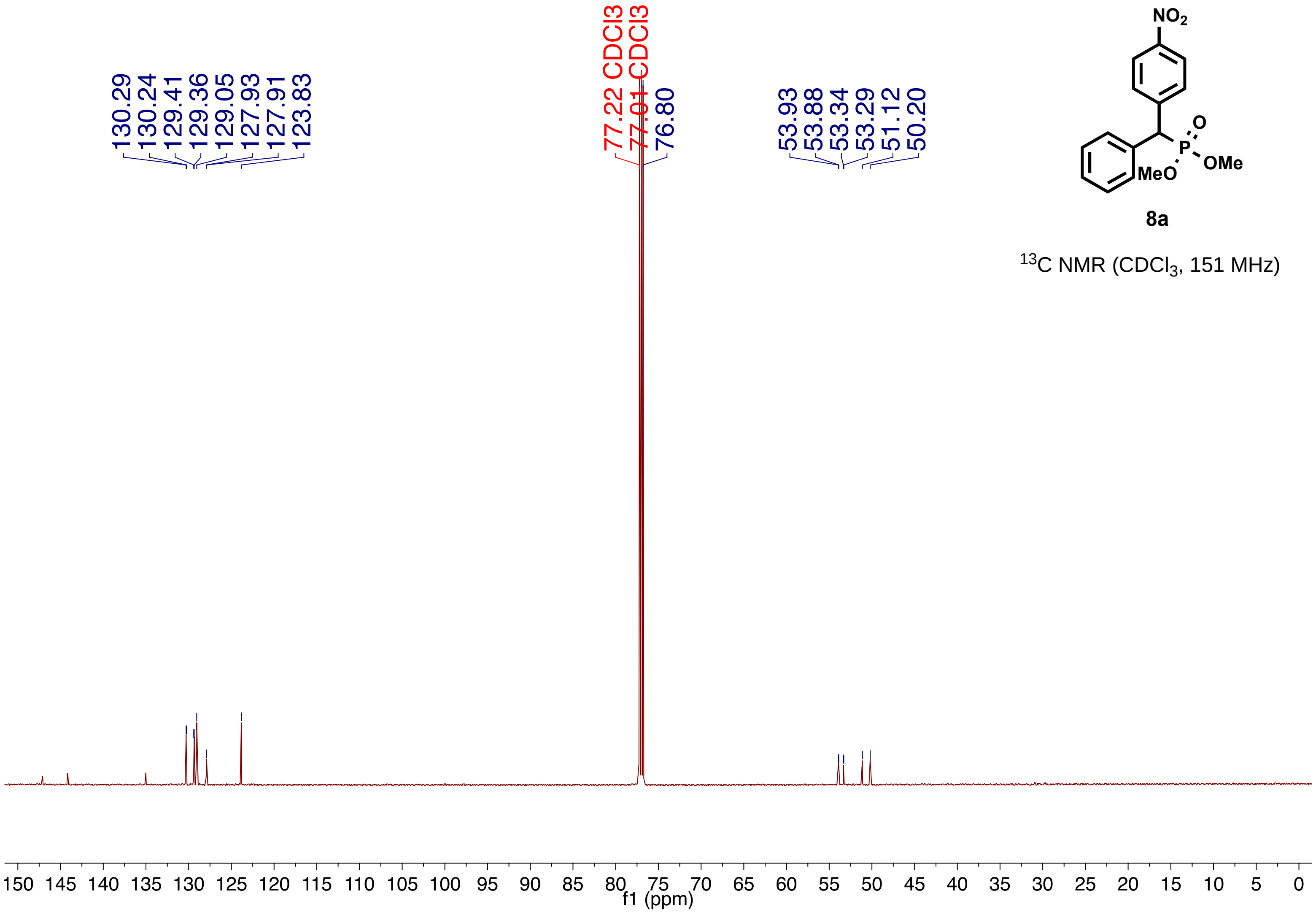

8a

^{13}C NMR ($CDCl_3$, 151 MHz)

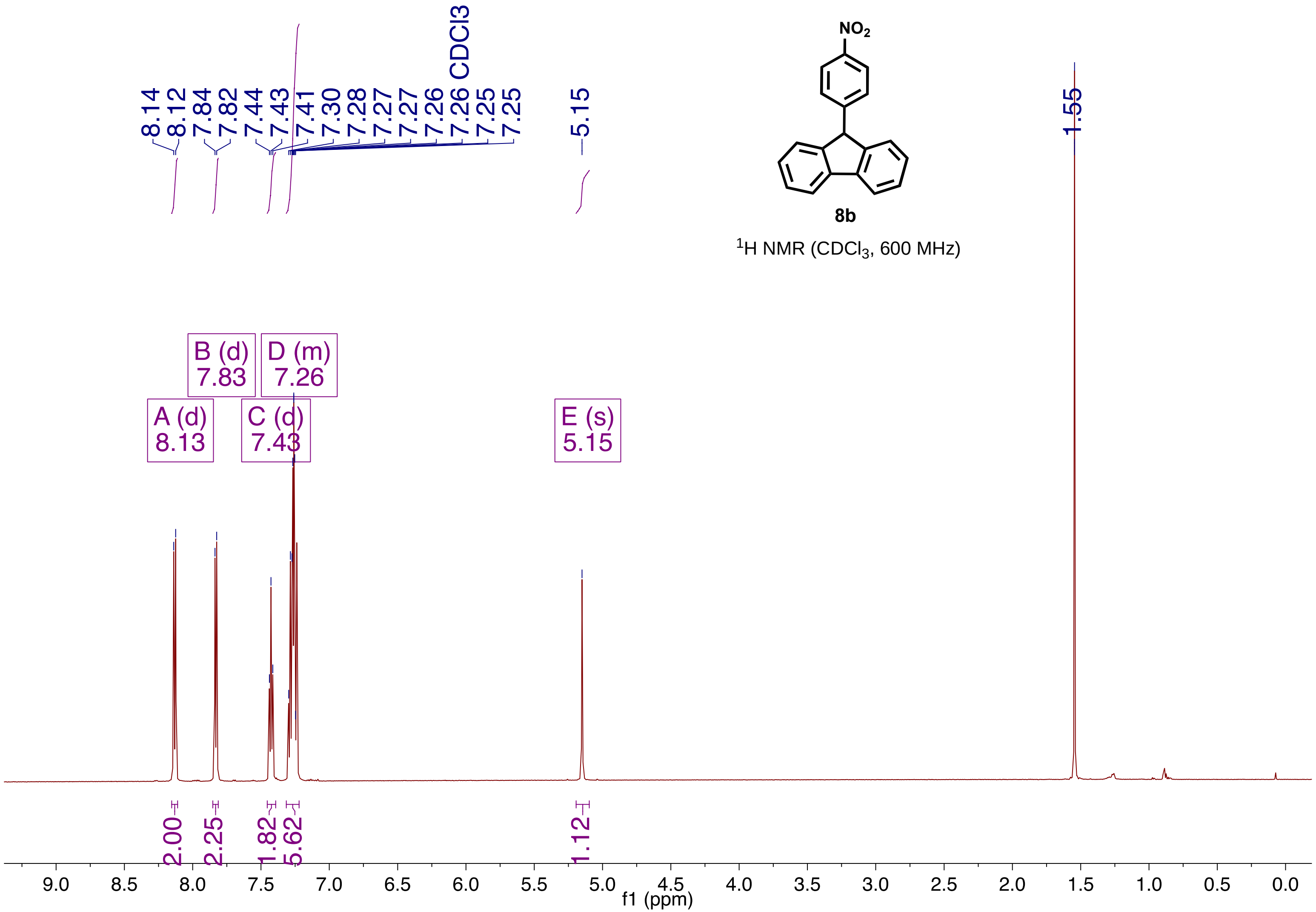

8.14
8.12
7.84
7.82
7.44
7.43
7.41
7.30
7.28
7.27
7.27
7.26
7.26 CDCl3
7.25
7.25
5.15
1.55
NO2
8b
1H NMR (CDCl3, 600 MHz)
B (d)
7.83
D (m)
7.26
A (d)
8.13
C (d)
7.43
E (s)
5.15
2.00
2.25
1.82
5.62
1.12
9.0
8.5
8.0
7.5
7.0
6.5
6.0
5.5
5.0
4.5
4.0
3.5
3.0
2.5
2.0
1.5
1.0
0.5
0.0
f1 (ppm)

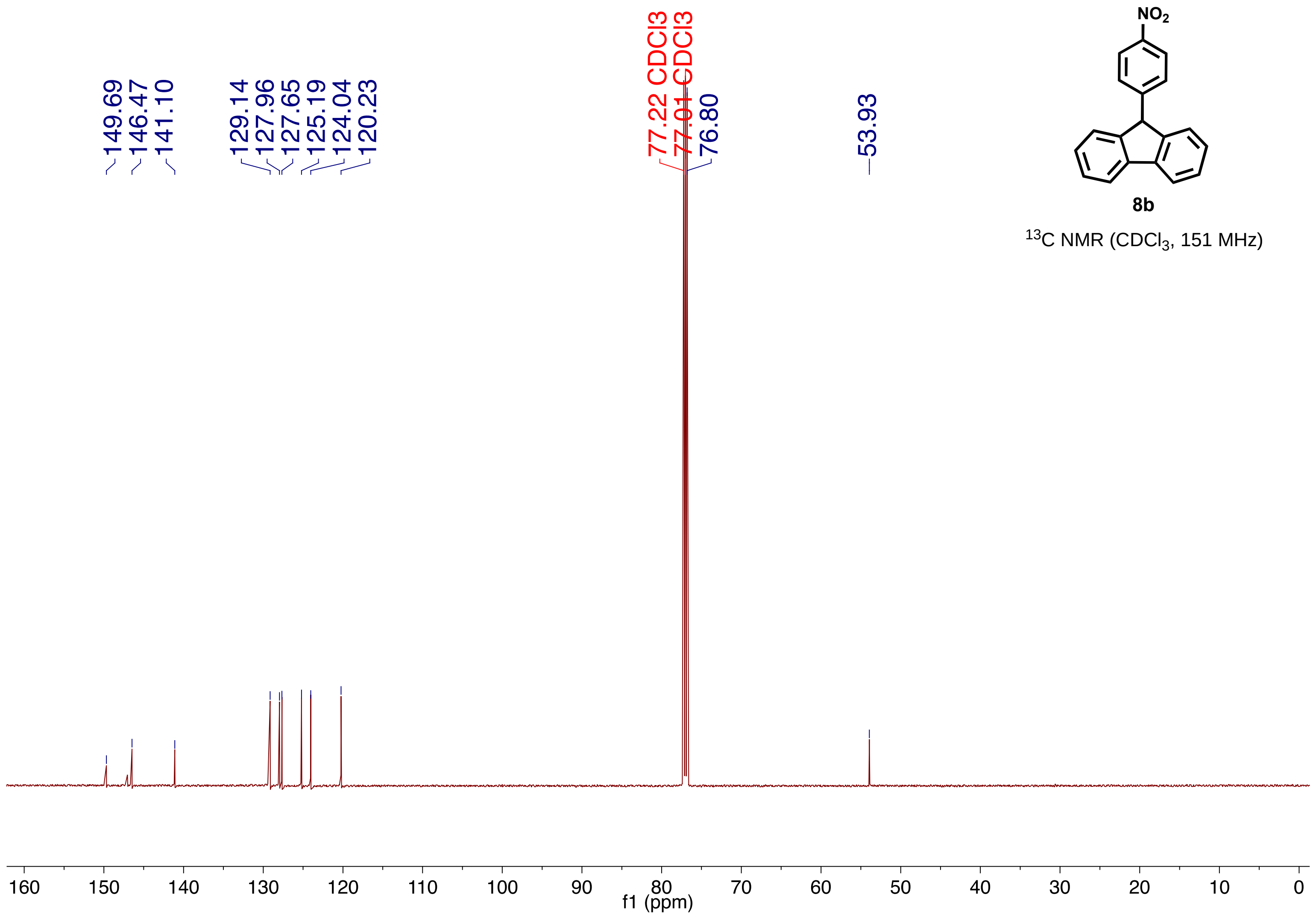
149.69
146.47
141.10
129.14
127.96
127.65
125.19
124.04
120.23
77.22 CDCl3
77.01 CDCl3
76.80
53.93
NO2
8b
13C NMR (CDCl3, 151 MHz)
160
150
140
130
120
110
100
90
80
70
60
50
40
30
20
10
0
f1 (ppm)

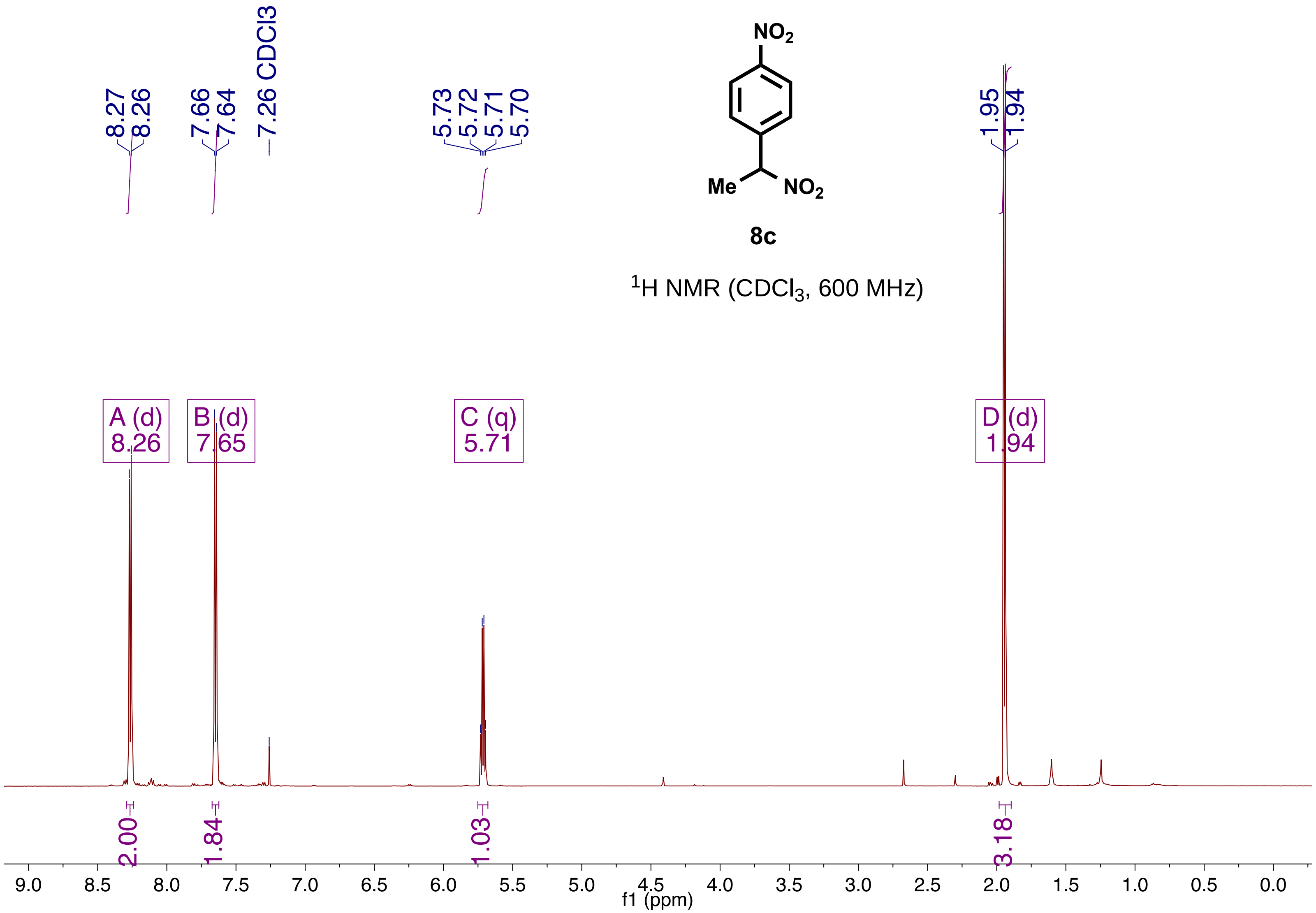

8.27
8.26
7.66
7.64
7.26 CDCl3
5.73
5.72
5.71
5.70
1.95
1.94
NO2
Me
NO2
8c
1H NMR (CDCl3, 600 MHz)
A (d) 8.26
B (d) 7.65
C (q) 5.71
D (d) 1.94
2.00
1.84
1.03
3.18
9.0
8.5
8.0
7.5
7.0
6.5
6.0
5.5
5.0
4.5
4.0
3.5
3.0
2.5
2.0
1.5
1.0
0.5
0.0
f1 (ppm)

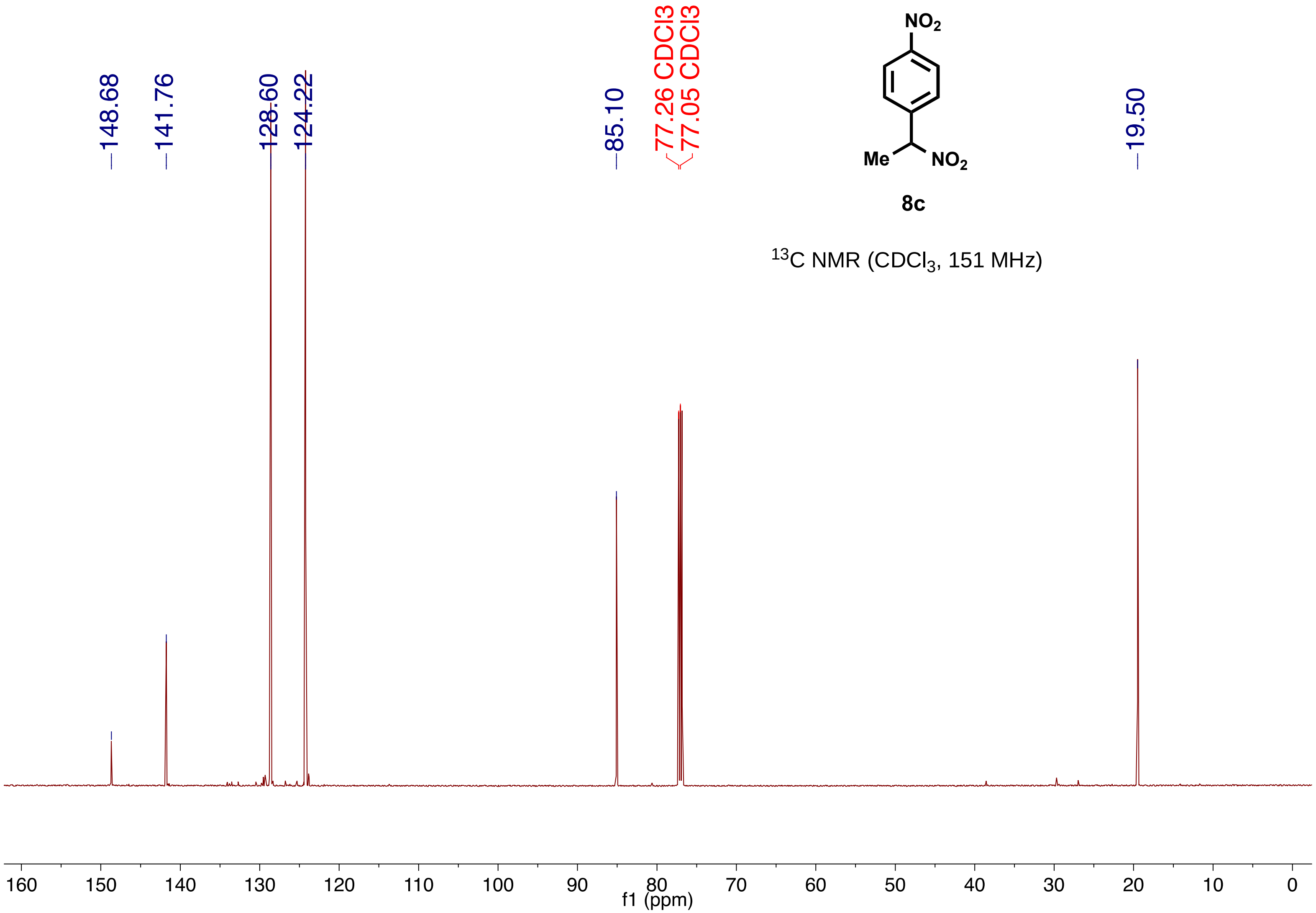

148.68
141.76
128.60
124.22
85.10
77.26 CDCl3
77.05 CDCl3
19.50
NO2
Me
NO2
8c
13C NMR (CDCl3, 151 MHz)
160
150
140
130
120
110
100
90
80
70
60
50
40
30
20
10
0
f1 (ppm)

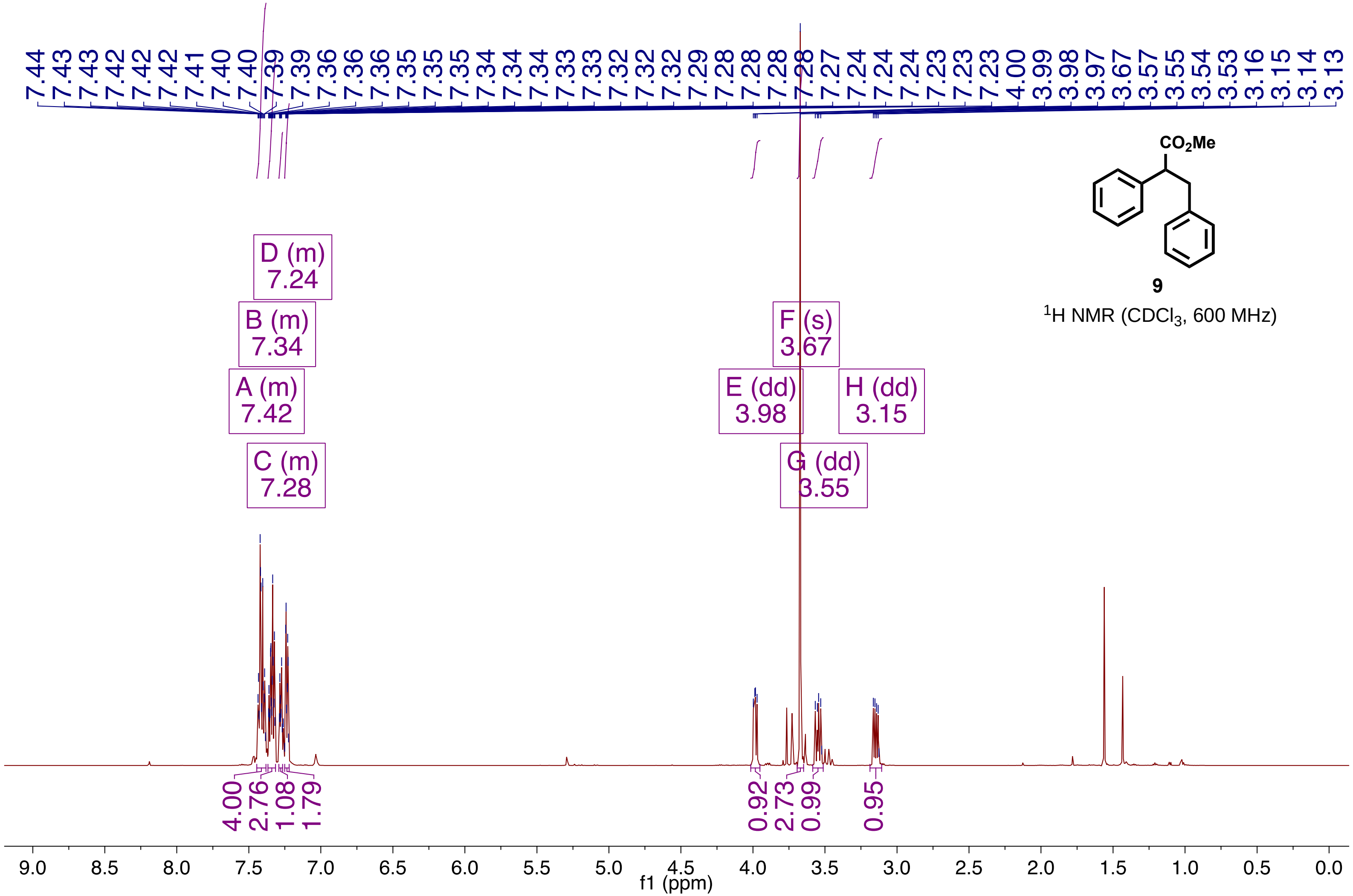

7.44 7.43 7.43 7.42 7.42 7.42 7.41 7.40 7.40 7.39 7.39 7.36 7.36 7.36 7.35 7.35 7.35 7.34 7.34 7.34 7.33 7.33 7.32 7.32 7.32 7.29 7.28 7.28 7.28 7.28 7.27 7.24 7.24 7.24 7.23 7.23 7.23 4.00 3.99 3.98 3.97 3.67 3.57 3.55 3.54 3.53 3.16 3.15 3.14 3.13
D (m) 7.24
B (m) 7.34
A (m) 7.42
C (m) 7.28
F (s) 3.67
E (dd) 3.98
H (dd) 3.15
G (dd) 3.55
CO_2Me
9
^{1}H NMR ($CDCl_3$, 600 MHz)
4.00 2.76 1.08 1.79 0.92 2.73 0.99 0.95
9.0 8.5 8.0 7.5 7.0 6.5 6.0 5.5 5.0 4.5 4.0 3.5 3.0 2.5 2.0 1.5 1.0 0.5 0.0
f1 (ppm)

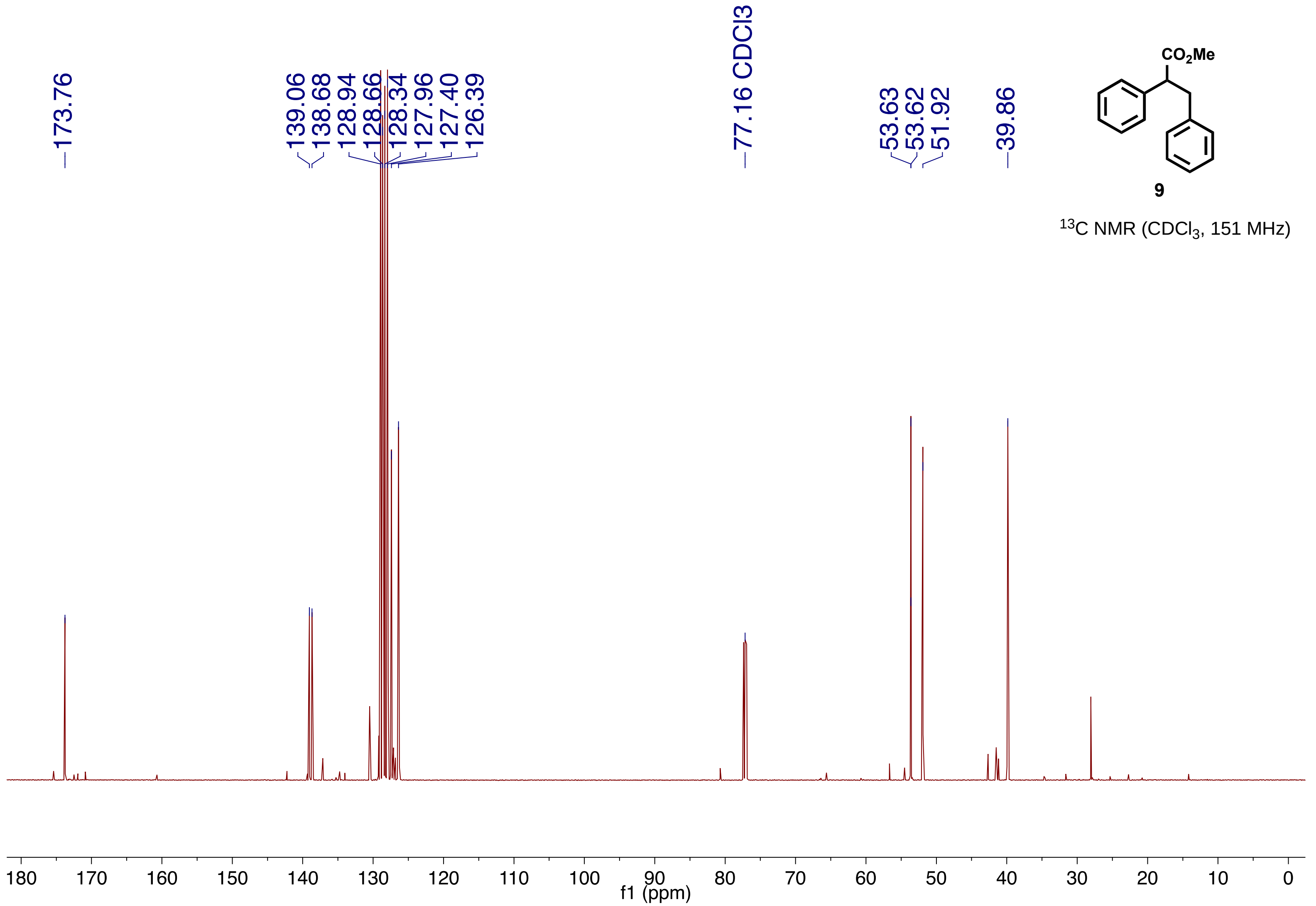
173.76
139.06
138.68
128.94
128.66
128.34
127.96
127.40
126.39
77.16 CDCl3
53.63
53.62
51.92
39.86
CO2Me
9
13C NMR (CDCl3, 151 MHz)
180 170 160 150 140 130 120 110 100 90 80 70 60 50 40 30 20 10 0
f1 (ppm)

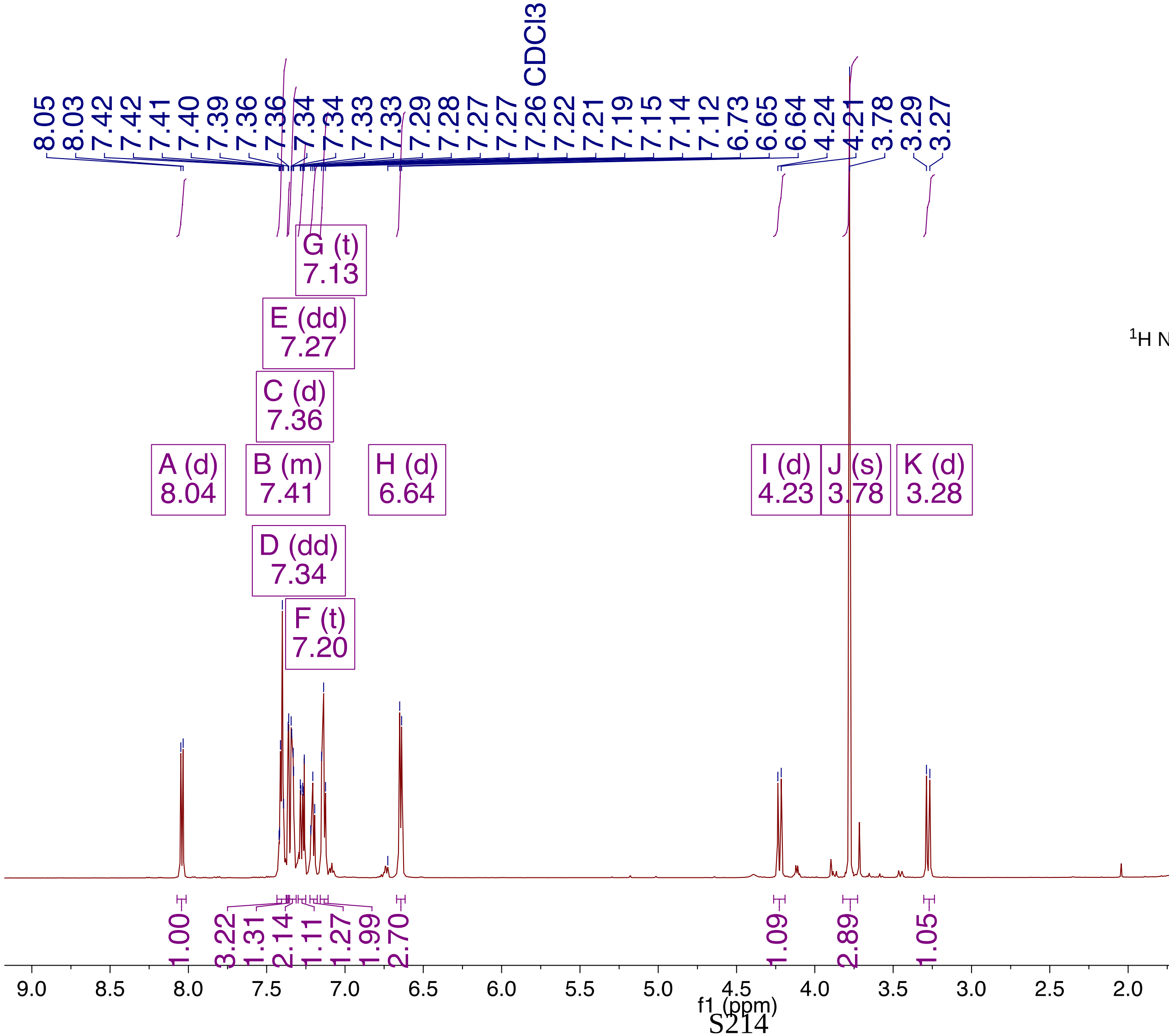

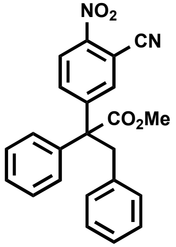

10

^{1}H NMR ($CDCl_3$, 600 MHz)

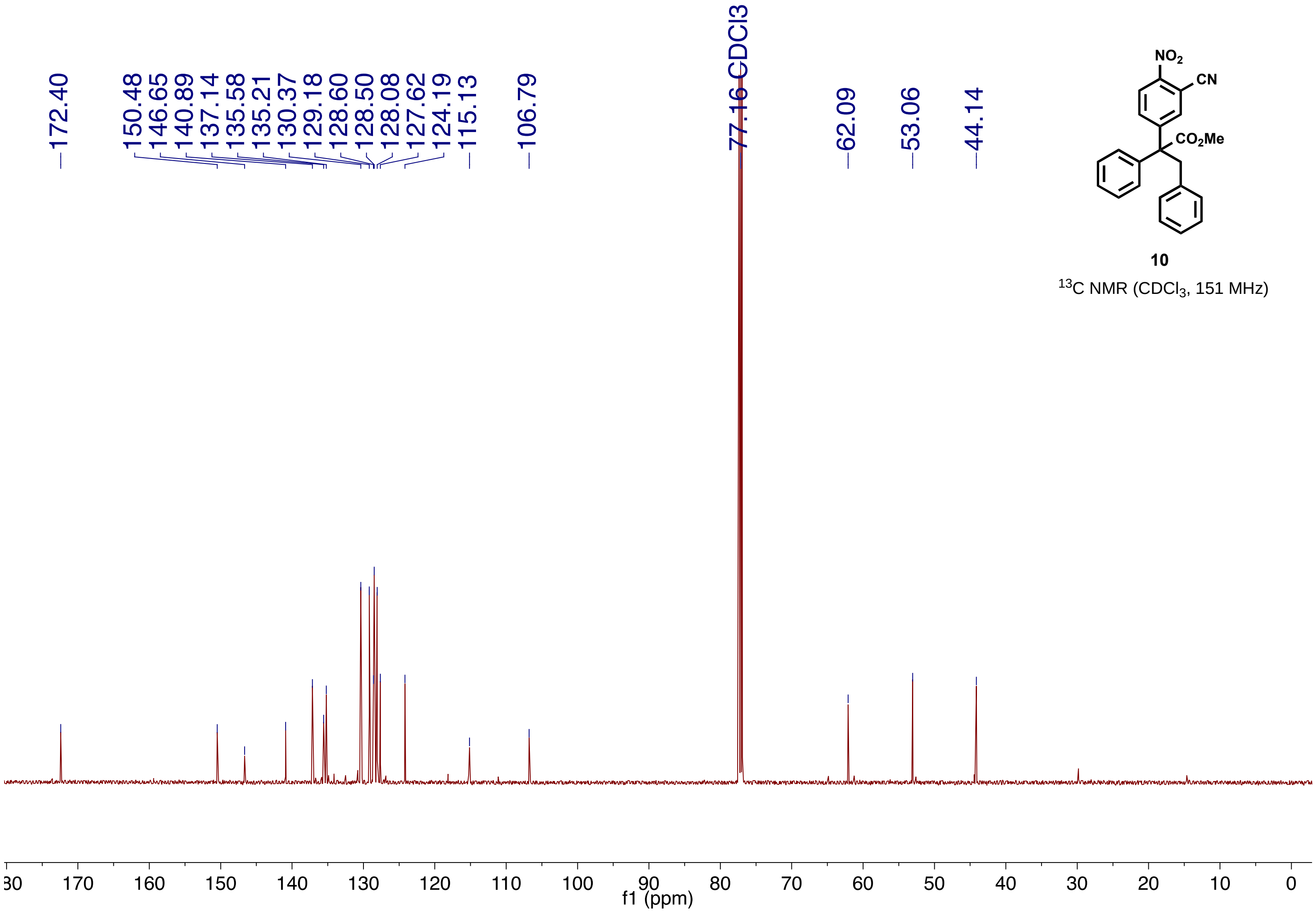

10

^{13}C NMR ($CDCl_3$, 151 MHz)

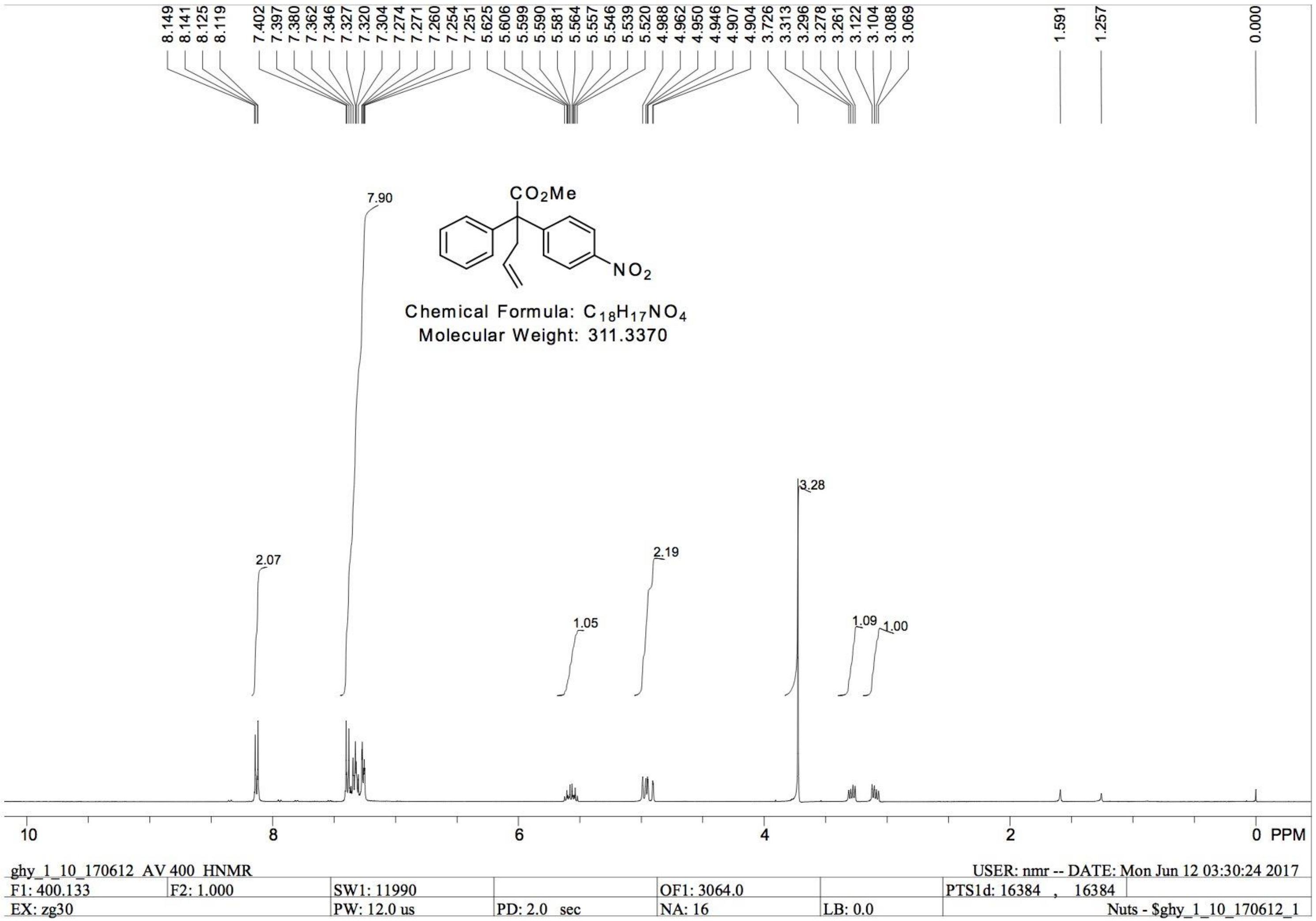
8.149 8.141 8.125 8.119 7.402 7.397 7.380 7.362 7.346 7.327 7.320 7.304 7.274 7.271 7.260 7.254 7.251 5.625 5.606 5.599 5.590 5.581 5.564 5.557 5.546 5.539 5.520 4.988 4.962 4.950 4.946 4.907 4.904 3.726 3.313 3.296 3.278 3.261 3.122 3.104 3.088 3.069 1.591 1.257 0.000
7.90
2.07
1.05
2.19
3.28
1.09
1.00
CO2Me
NO2
Chemical Formula: C18H17NO4
Molecular Weight: 311.3370
10
8
6
4
2
0 PPM
ghy_1_10_170612 AV 400 HNMR
USER: nmr -- DATE: Mon Jun 12 03:30:24 2017
F1: 400.133
F2: 1.000
SW1: 11990
OF1: 3064.0
PTS1d: 16384 , 16384
EX: zg30
PW: 12.0 us
PD: 2.0 sec
NA: 16
LB: 0.0
Nuts - $ghy_1_10_170612_1

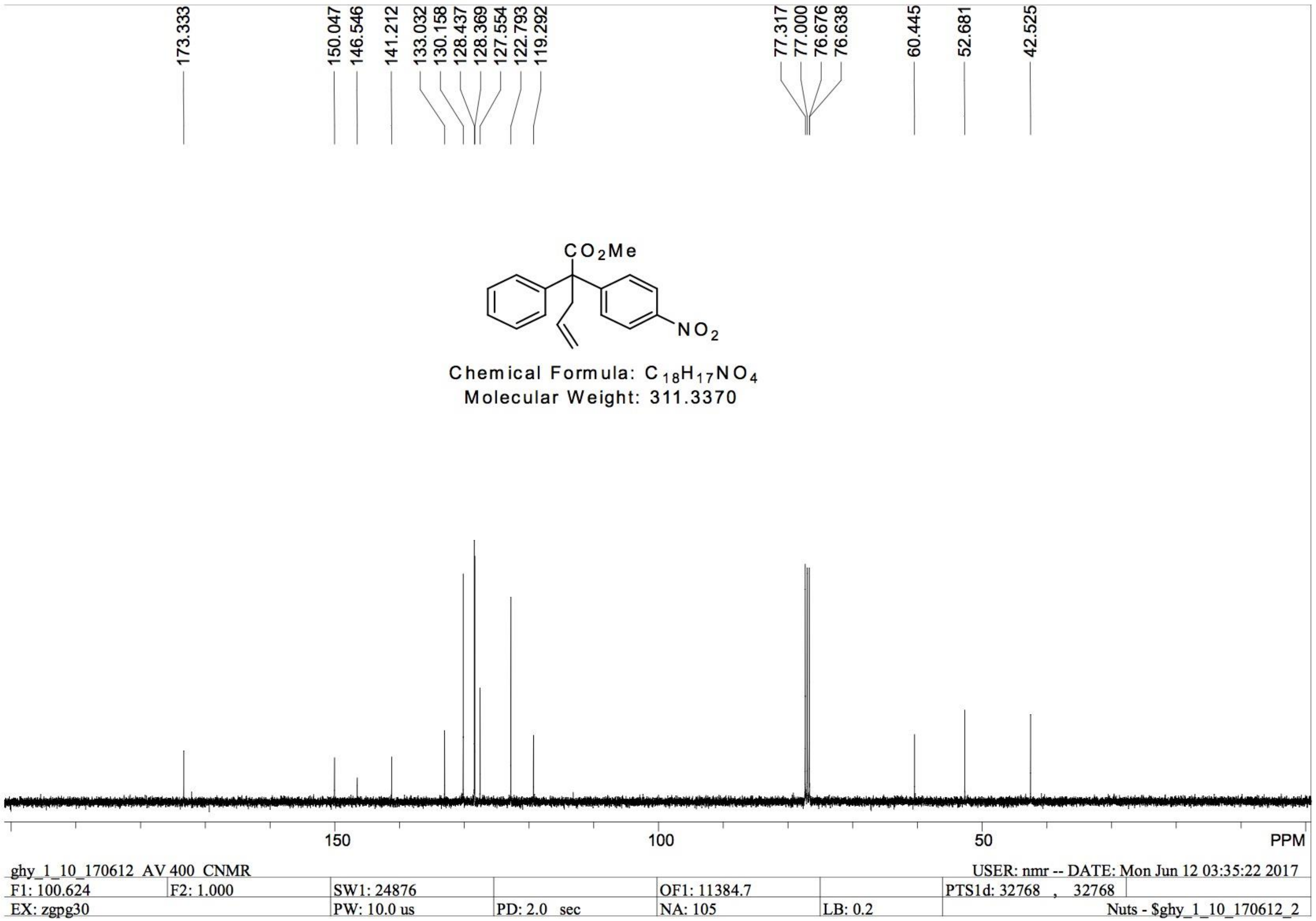
173.333
150.047
146.546
141.212
133.032
130.158
128.437
128.369
127.554
122.793
119.292
77.317
77.000
76.676
76.638
60.445
52.681
42.525
CO2Me
NO2
Chemical Formula: C18H17NO4
Molecular Weight: 311.3370
150
100
50
PPM
ghy_1_10_170612 AV 400 CNMR
USER: nmr -- DATE: Mon Jun 12 03:35:22 2017
F1: 100.624
F2: 1.000
SW1: 24876
OF1: 11384.7
PTS1d: 32768 , 32768
EX: zgpg30
PW: 10.0 us
PD: 2.0 sec
NA: 105
LB: 0.2
Nuts - $ghy_1_10_170612_2

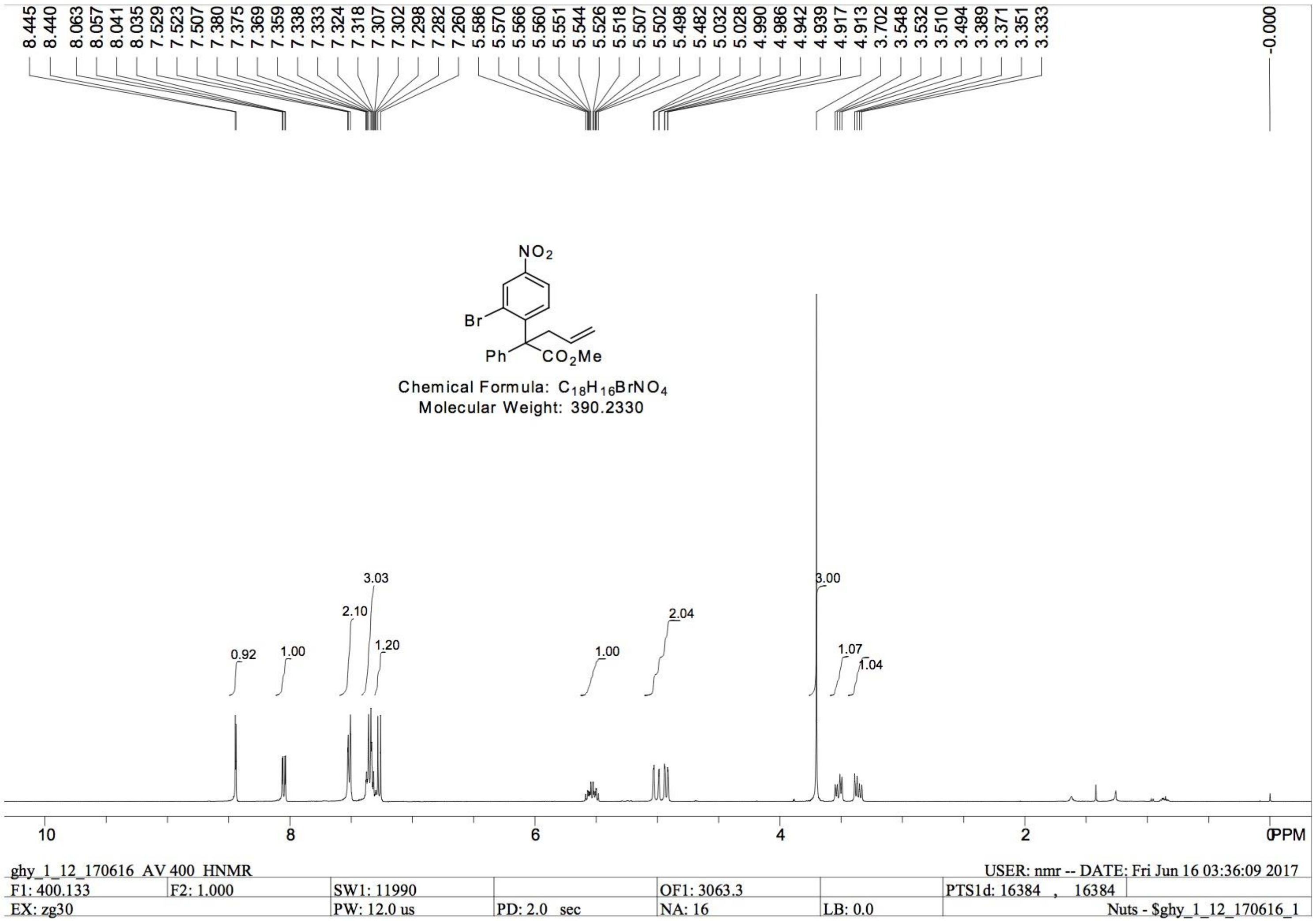

8.445
8.440
8.063
8.057
8.041
8.035
7.529
7.523
7.507
7.380
7.375
7.369
7.359
7.338
7.333
7.324
7.318
7.307
7.302
7.298
7.282
7.260
5.586
5.570
5.566
5.560
5.551
5.544
5.526
5.518
5.507
5.502
5.498
5.482
5.032
5.028
4.990
4.986
4.942
4.939
4.917
4.913
3.702
3.548
3.532
3.510
3.494
3.389
3.371
3.351
3.333
-0.000
NO_2
Br
Ph
CO_2Me
Chemical Formula: $C_{18}H_{16}BrNO_4$
Molecular Weight: 390.2330
0.92
1.00
2.10
3.03
1.20
1.00
2.04
3.00
1.07
1.04
10
8
6
4
2
0
PPM
ghy_1_12_170616 AV 400 HNMR
USER: nmr -- DATE: Fri Jun 16 03:36:09 2017
F1: 400.133
F2: 1.000
SW1: 11990
OF1: 3063.3
PTS1d: 16384 , 16384
EX: zg30
PW: 12.0 us
PD: 2.0 sec
NA: 16
LB: 0.0
Nuts - $ghy_1_12_170616_1

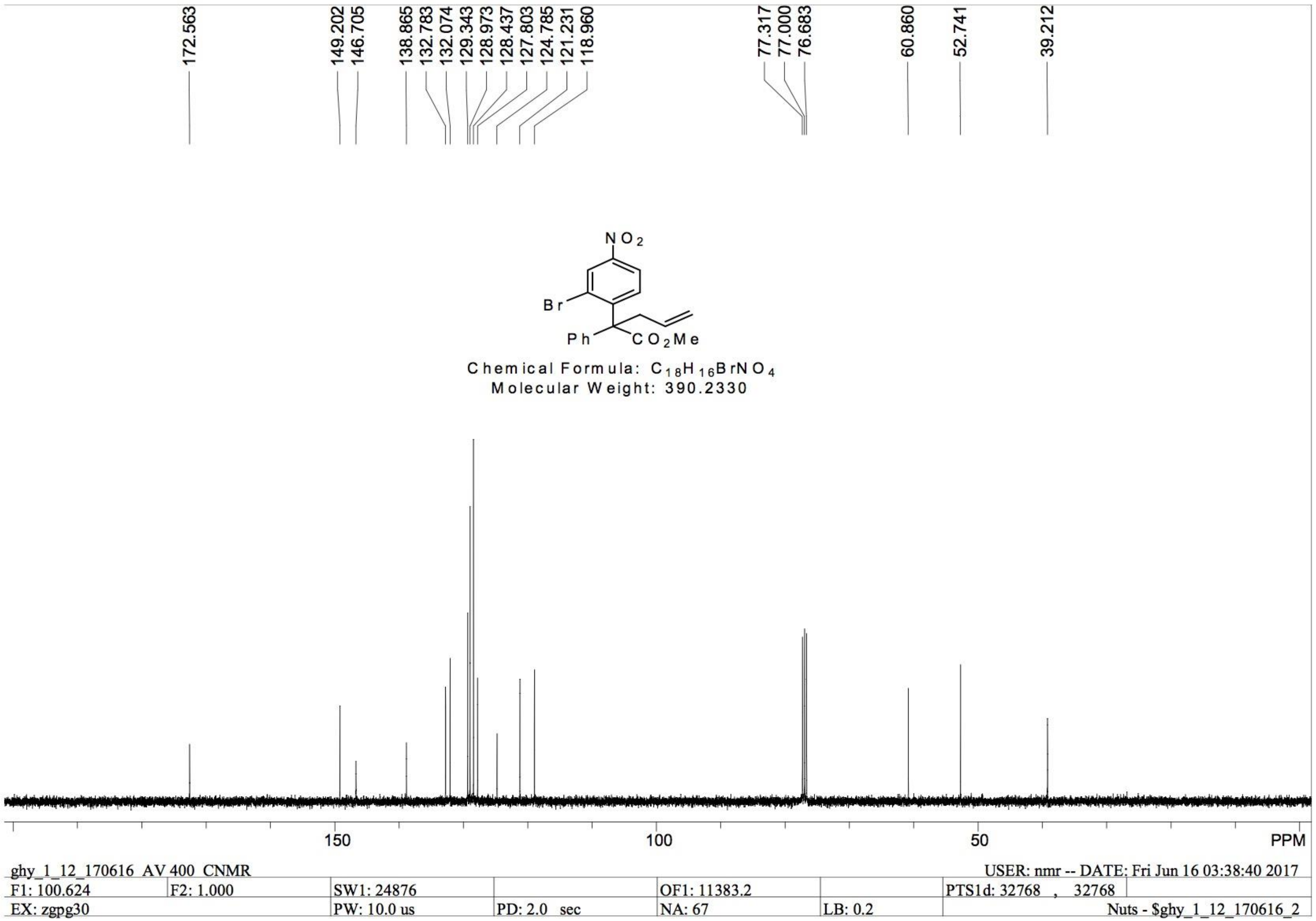
172.563
149.202
146.705
138.865
132.783
132.074
129.343
128.973
128.437
127.803
124.785
121.231
118.960
77.317
77.000
76.683
60.860
52.741
39.212
NO2
Br
Ph
CO2Me
Chemical Formula: C18H16BrNO4
Molecular Weight: 390.2330
150
100
50
PPM
ghy_1_12_170616 AV 400 CNMR
USER: nmr -- DATE: Fri Jun 16 03:38:40 2017
F1: 100.624
F2: 1.000
SW1: 24876
OF1: 11383.2
PTS1d: 32768 , 32768
EX: zgpg30
PW: 10.0 us
PD: 2.0 sec
NA: 67
LB: 0.2
Nuts - $ghy_1_12_170616_2

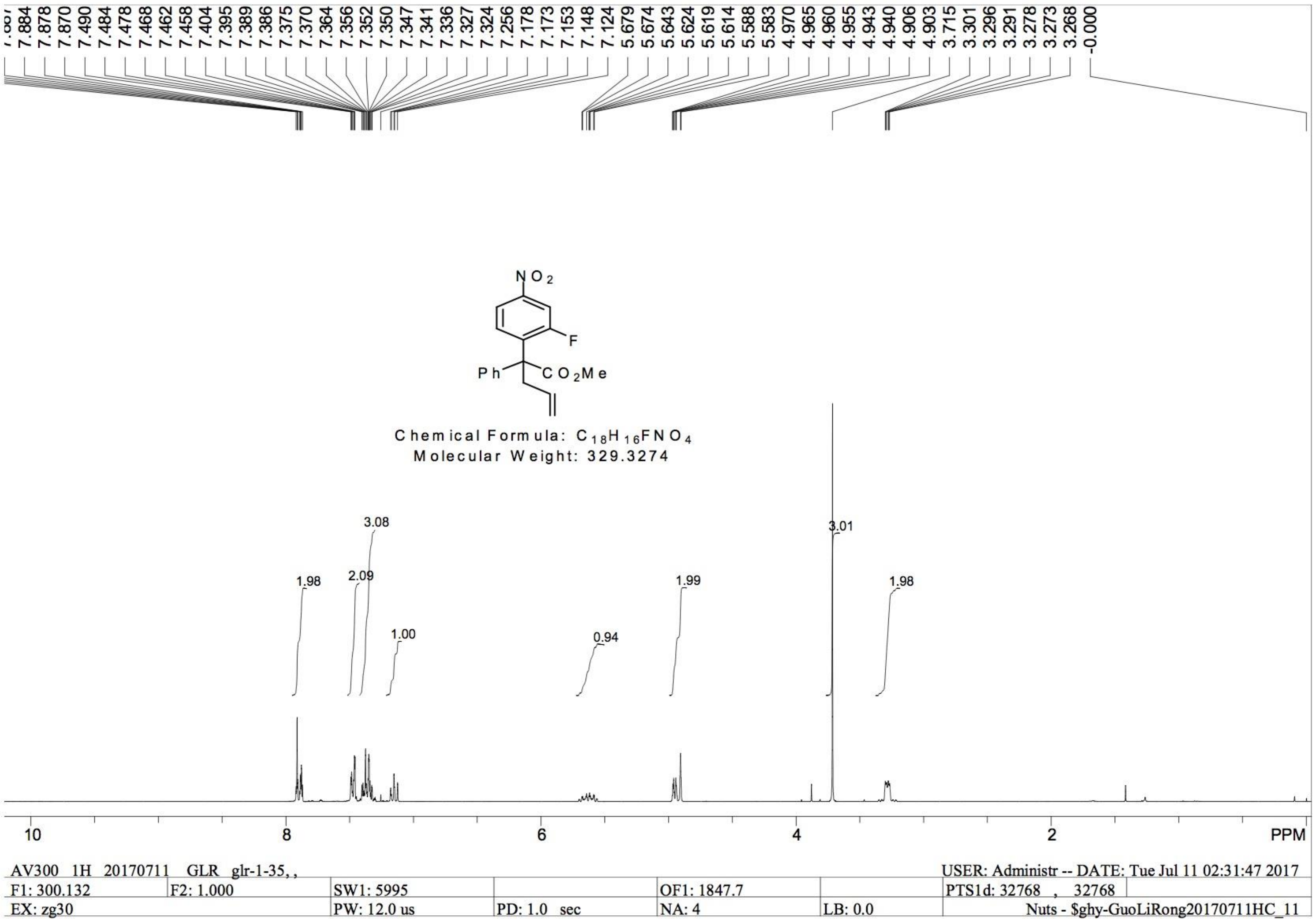

NO2
F
Ph
CO2Me
Chemical Formula: C18H16FNO4
Molecular Weight: 329.3274
7.884
7.878
7.870
7.490
7.484
7.478
7.468
7.462
7.458
7.404
7.395
7.389
7.386
7.375
7.370
7.364
7.356
7.352
7.350
7.347
7.341
7.336
7.327
7.324
7.256
7.178
7.173
7.153
7.148
7.124
5.679
5.674
5.643
5.624
5.619
5.614
5.588
5.583
4.970
4.965
4.960
4.955
4.943
4.940
4.906
4.903
3.715
3.301
3.296
3.291
3.278
3.273
3.268
-0.000
1.98
2.09
3.08
1.00
0.94
1.99
3.01
1.98
10
8
6
4
2
PPM
AV300 1H 20170711 GLR glr-1-35, ,
USER: Administr -- DATE: Tue Jul 11 02:31:47 2017
F1: 300.132
F2: 1.000
SW1: 5995
OF1: 1847.7
PTS1d: 32768 , 32768
EX: zg30
PW: 12.0 us
PD: 1.0 sec
NA: 4
LB: 0.0
Nuts - $ghy-GuoLiRong20170711HC_11

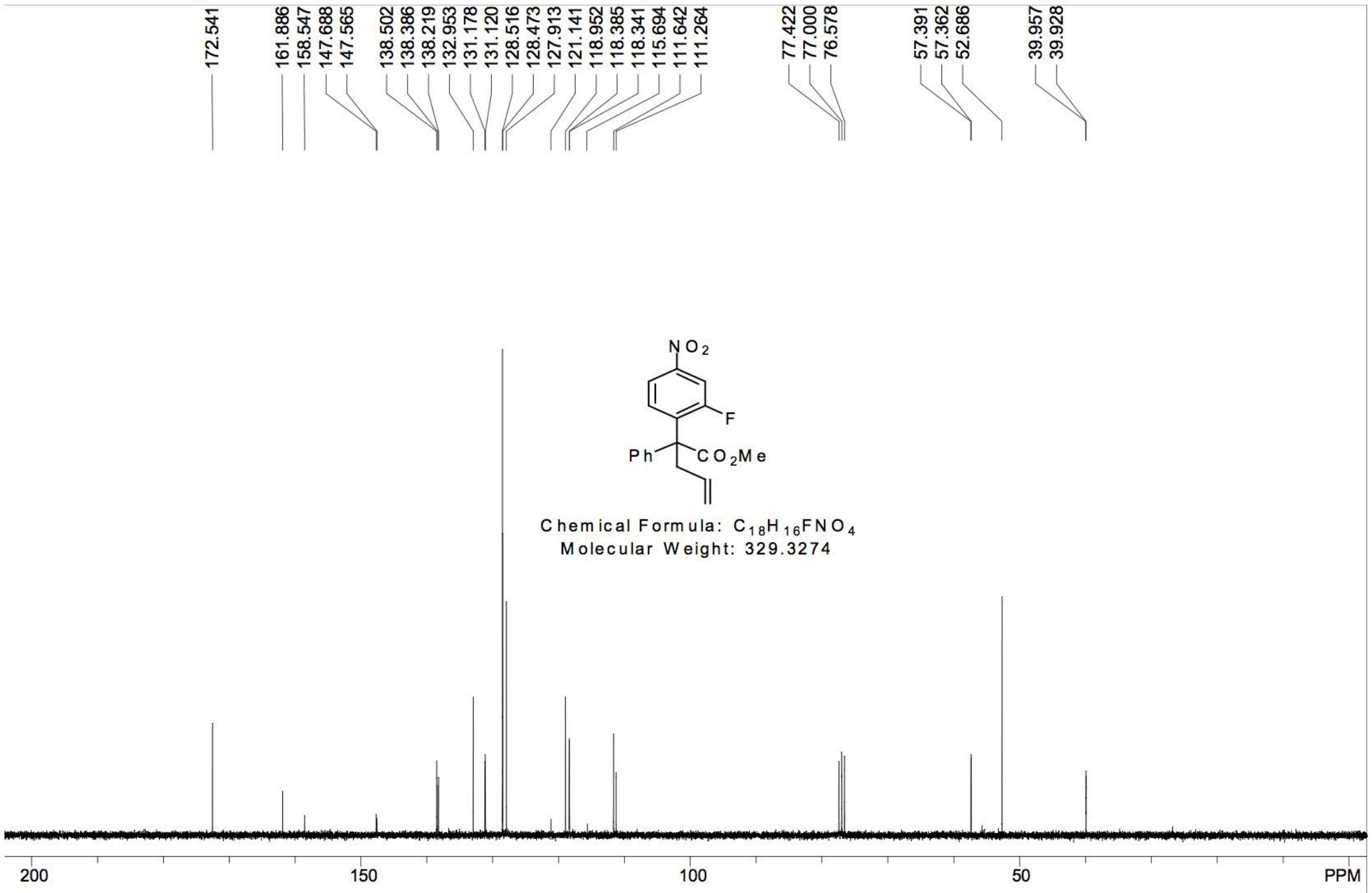

AV300 13C 20170711 GLR glr-1-35, , USER: Administr -- DATE: Tue Jul 11 02:41:11 2017

F1: 75.475	F2: 1.000	SW1: 17986		OF1: 7540.3		PTS1d: 32768 , 32768	
EX: zgpg30		PW: 14.3 us	PD: 2.0 sec	NA: 128	LB: 0.0	Nuts - $ghy-GuoLiRong20170711HC_12	

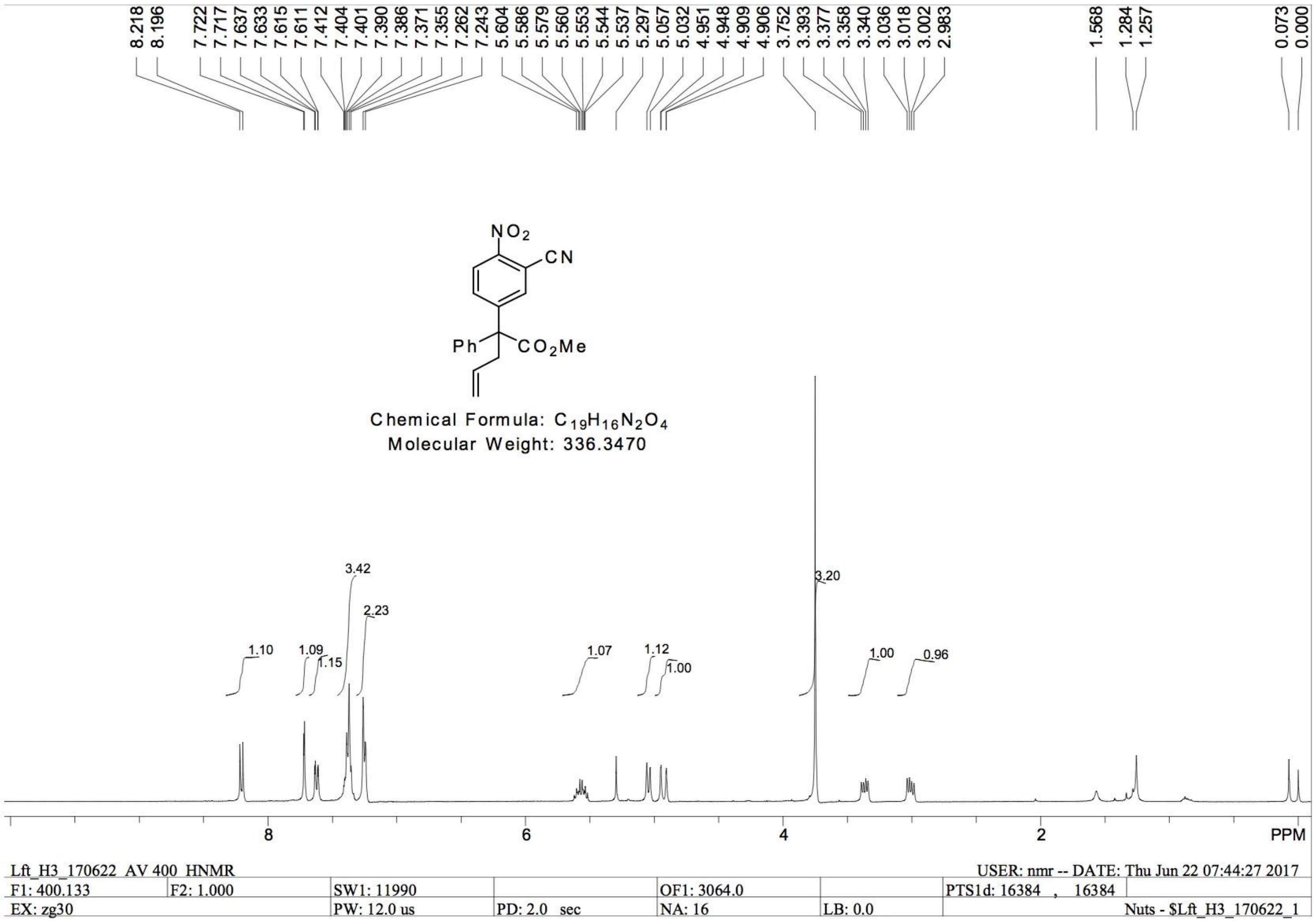
8.218 8.196 7.722 7.717 7.637 7.633 7.615 7.611 7.412 7.404 7.401 7.390 7.386 7.371 7.355 7.262 7.243 5.604 5.586 5.579 5.560 5.553 5.544 5.537 5.297 5.057 5.032 4.951 4.948 4.909 4.906 3.752 3.393 3.377 3.358 3.340 3.036 3.018 3.002 2.983 1.568 1.284 1.257 0.073 0.000
NO2
CN
Ph
CO2Me
Chemical Formula: C19H16N2O4
Molecular Weight: 336.3470
1.10 1.09 1.15 3.42 2.23 1.07 1.12 1.00 3.20 1.00 0.96
8 6 4 2 PPM
Lft_H3_170622_AV 400_HNMR
USER: nmr -- DATE: Thu Jun 22 07:44:27 2017
F1: 400.133
F2: 1.000
SW1: 11990
OF1: 3064.0
PTS1d: 16384 , 16384
EX: zg30
PW: 12.0 us
PD: 2.0 sec
NA: 16
LB: 0.0
Nuts - $Lft_H3_170622_1

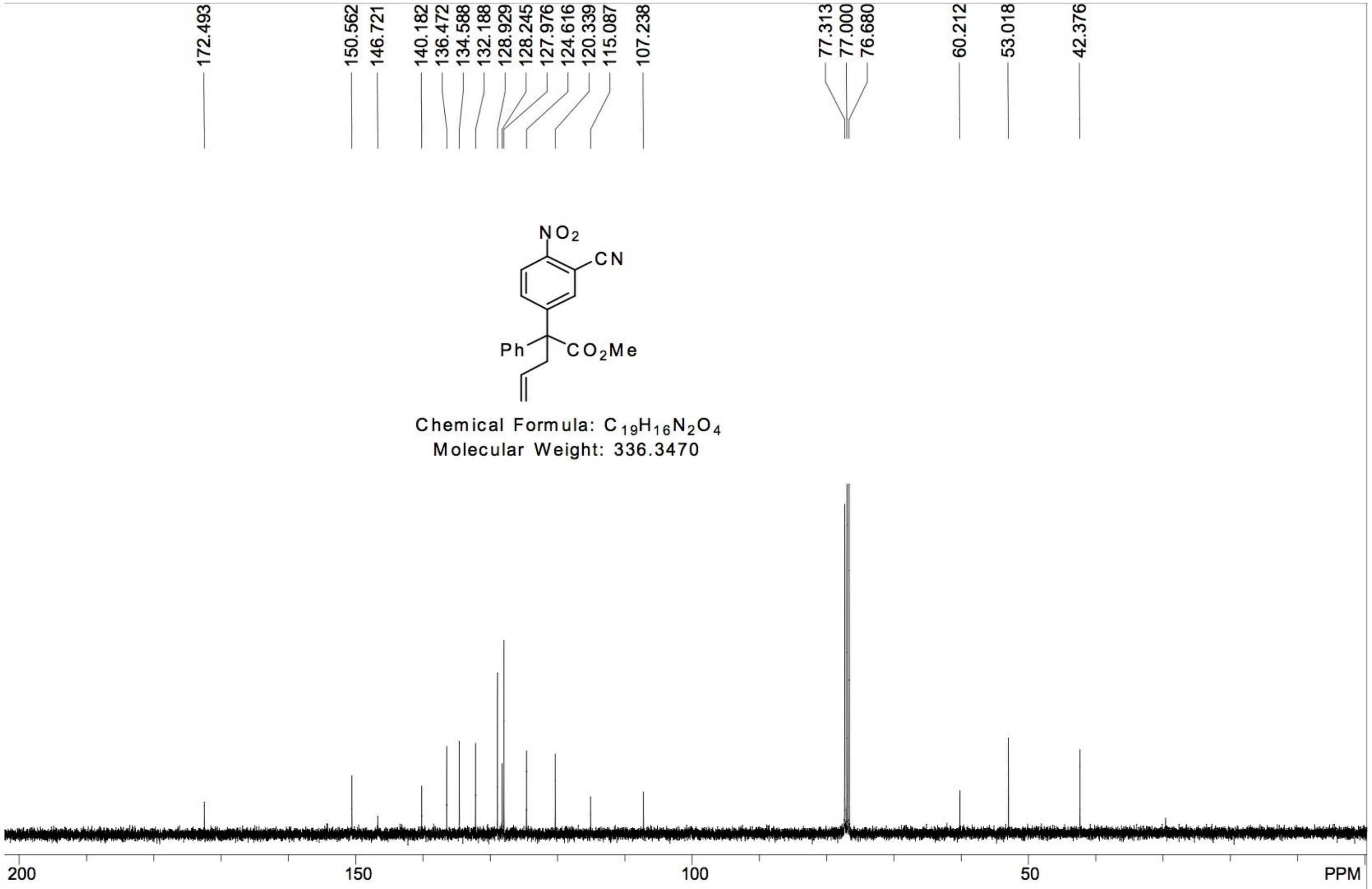

LFT_H3_170622_AV 400_CNMR						USER: nmr -- DATE: Thu Jun 22 07:35:25 2017
F1: 100.624	F2: 1.000	SW1: 23981		OF1: 10863.8		PTS1d: 32768 , 32768
EX: zgpg30		PW: 10.0 us	PD: 2.0 sec	NA: 218	LB: 0.2	Nuts - $Lft_H3_170622_2

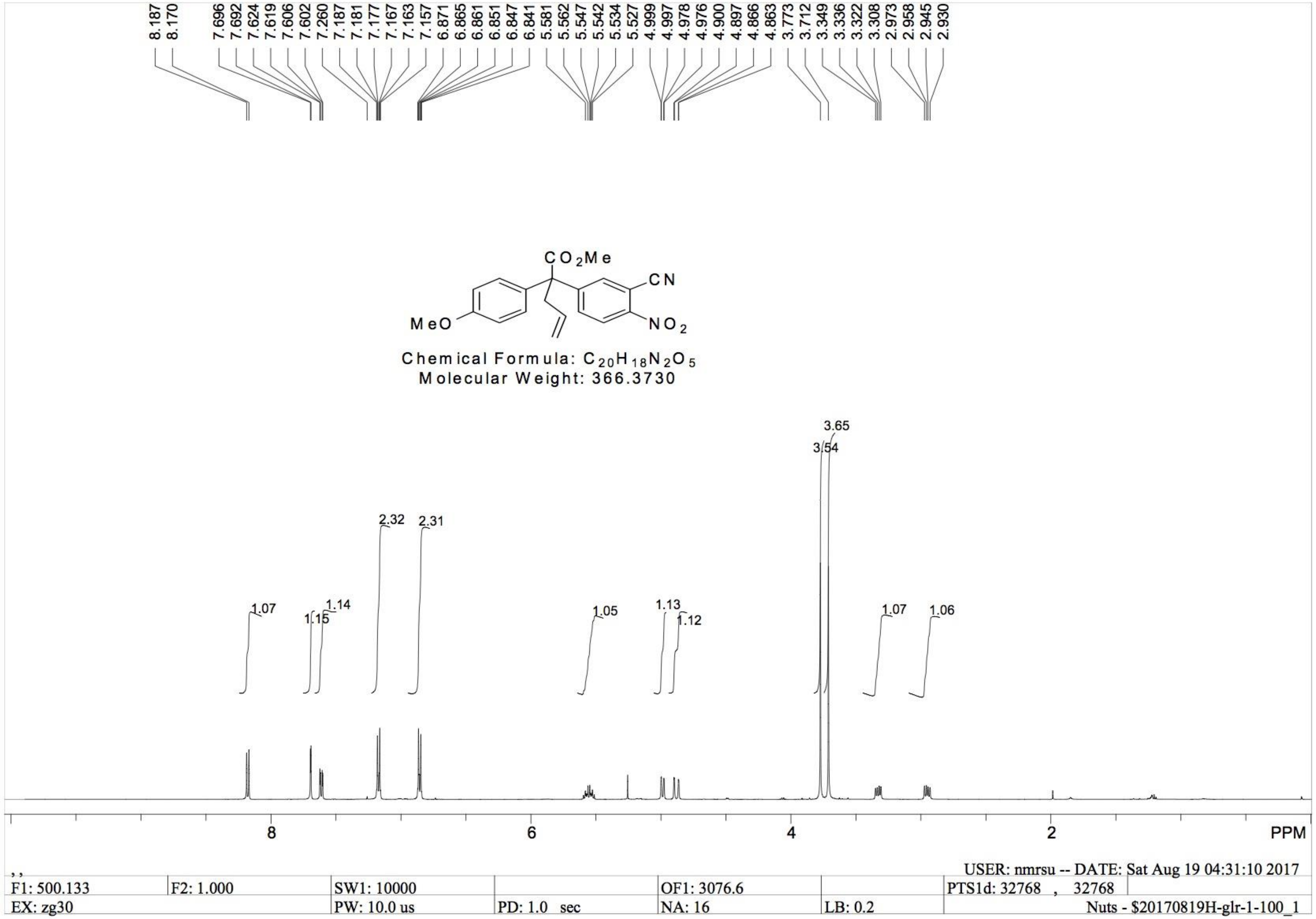
8.187
8.170
7.696
7.692
7.624
7.619
7.606
7.602
7.260
7.187
7.181
7.177
7.167
7.163
7.157
6.871
6.865
6.861
6.851
6.847
6.841
5.581
5.562
5.547
5.542
5.534
5.527
4.999
4.997
4.978
4.976
4.900
4.897
4.866
4.863
3.773
3.712
3.349
3.336
3.322
3.308
2.973
2.958
2.945
2.930
CO2Me
CN
MeO
NO2
Chemical Formula: C20H18N2O5
Molecular Weight: 366.3730
3.65
3.54
2.32
2.31
1.07
1.15
1.14
1.05
1.13
1.12
1.07
1.06
8
6
4
2
PPM
USER: nmrsu -- DATE: Sat Aug 19 04:31:10 2017
F1: 500.133
F2: 1.000
SW1: 10000
OF1: 3076.6
PTS1d: 32768 , 32768
EX: zg30
PW: 10.0 us
PD: 1.0 sec
NA: 16
LB: 0.2
Nuts - $20170819H-glr-1-100_1

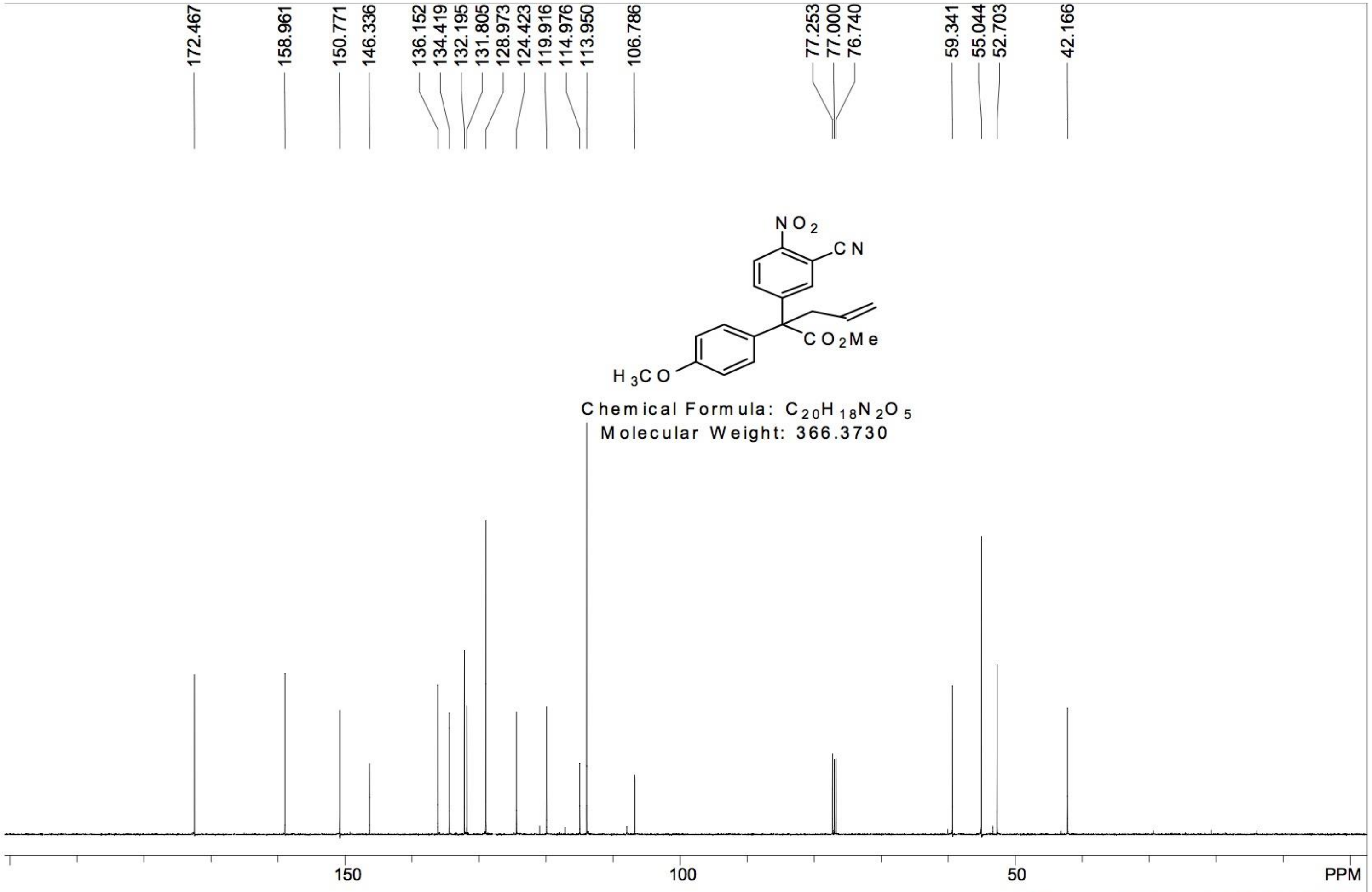

USER: nmrsu -- DATE: Sat Aug 19 04:39:04 2017

F1: 125.770	F2: 1.000	SW1: 29762		OF1: 12541.3		PTS1d: 32768 , 32768	
EX: zgpg30		PW: 10.0 us	PD: 2.0 sec	NA: 128	LB: 0.0	Nuts - $20170819C13-glr-1-100_1	

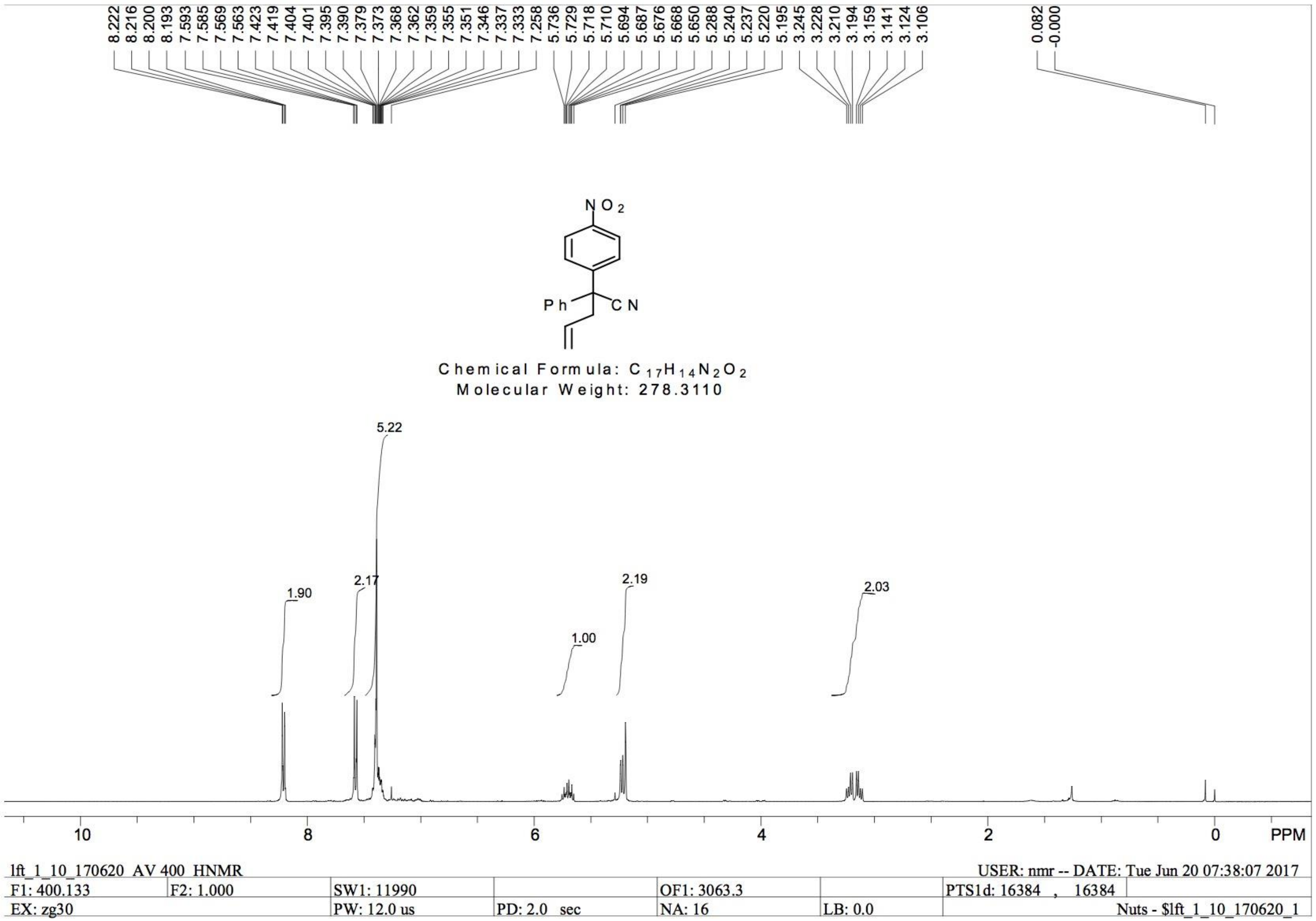
8.222 8.216 8.200 8.193 7.593 7.585 7.569 7.563 7.423 7.419 7.404 7.401 7.395 7.390 7.379 7.373 7.368 7.362 7.359 7.355 7.351 7.346 7.337 7.333 7.258 5.736 5.729 5.718 5.710 5.694 5.687 5.676 5.668 5.650 5.288 5.240 5.237 5.220 5.195 3.245 3.228 3.210 3.194 3.159 3.141 3.124 3.106
0.082 -0.000
NO2
Ph
CN
Chemical Formula: C17H14N2O2
Molecular Weight: 278.3110
5.22
1.90
2.17
2.19
1.00
2.03
10
8
6
4
2
0
PPM
lft_1_10_170620 AV 400 HNMR
USER: nmr -- DATE: Tue Jun 20 07:38:07 2017
F1: 400.133
F2: 1.000
SW1: 11990
OF1: 3063.3
PTS1d: 16384 , 16384
EX: zg30
PW: 12.0 us
PD: 2.0 sec
NA: 16
LB: 0.0
Nuts - $lft_1_10_170620_1

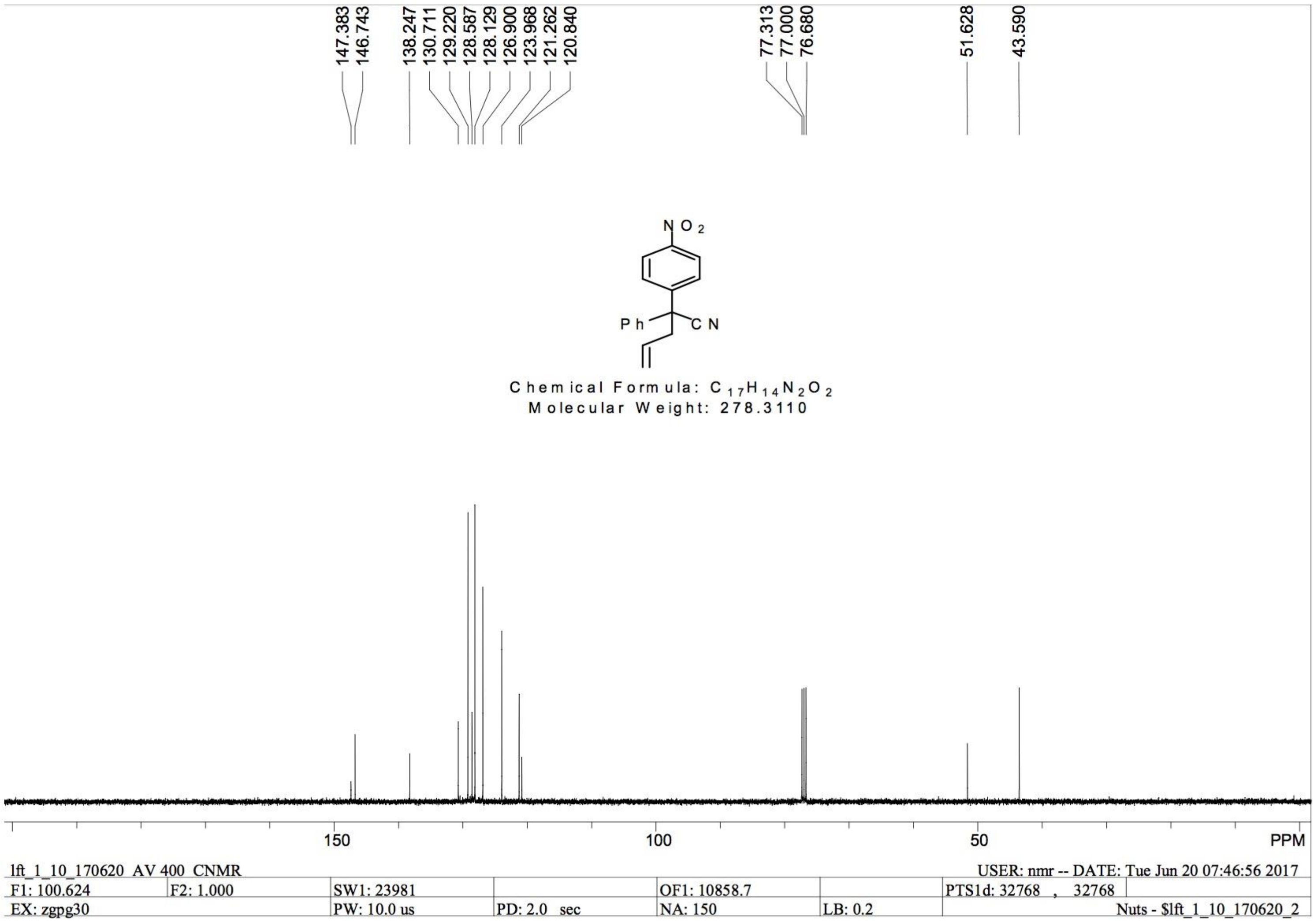

147.383
146.743
138.247
130.711
129.220
128.587
128.129
126.900
123.968
121.262
120.840
77.313
77.000
76.680
51.628
43.590
NO2
Ph
CN
Chemical Formula: C17H14N2O2
Molecular Weight: 278.3110
150
100
50
PPM
lft_1_10_170620 AV 400 CNMR
USER: nmr -- DATE: Tue Jun 20 07:46:56 2017
F1: 100.624
F2: 1.000
SW1: 23981
OF1: 10858.7
PTS1d: 32768 , 32768
EX: zgpg30
PW: 10.0 us
PD: 2.0 sec
NA: 150
LB: 0.2
Nuts - $lft_1_10_170620_2

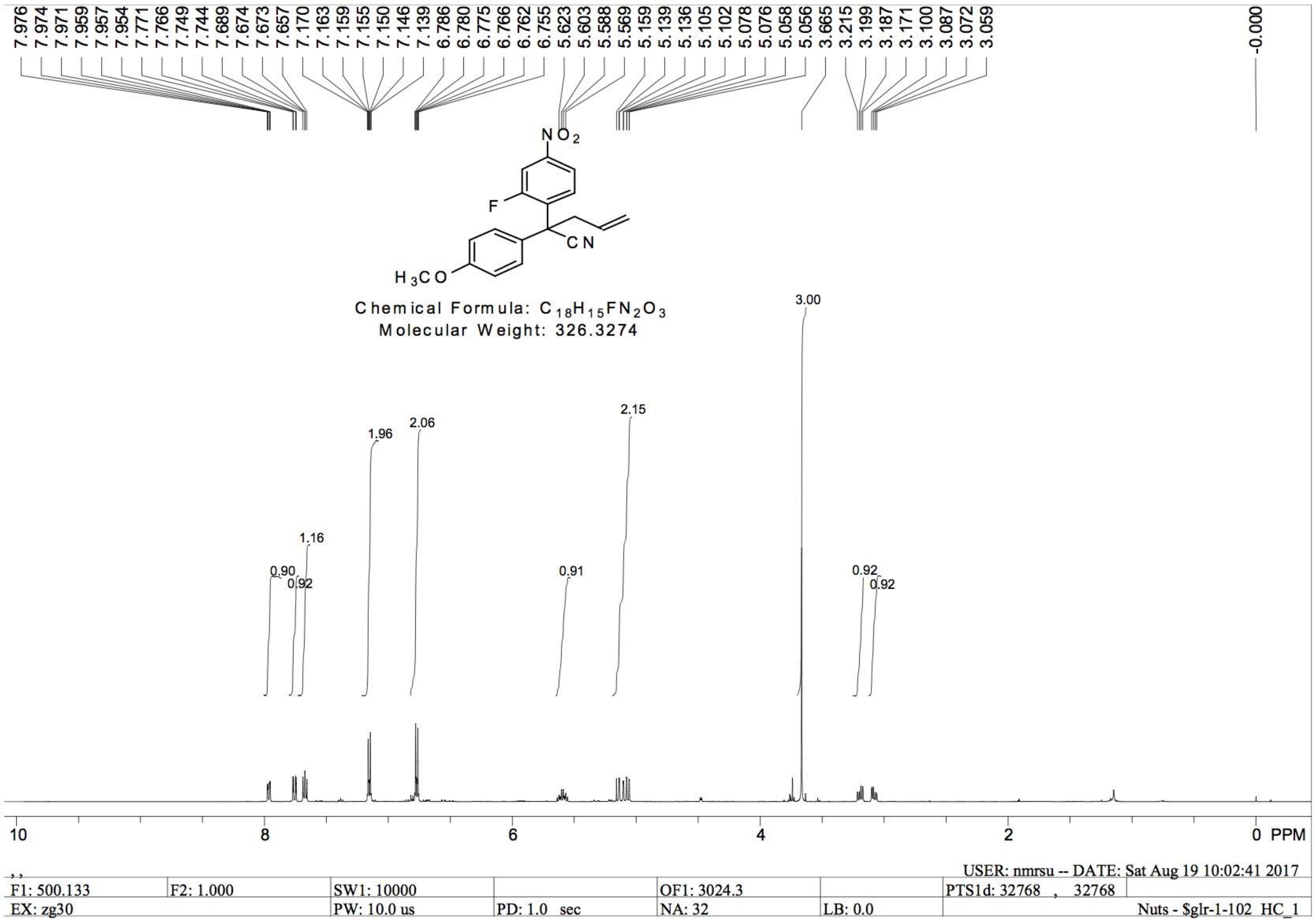
7.976
7.974
7.971
7.959
7.957
7.954
7.771
7.766
7.749
7.744
7.689
7.674
7.673
7.657
7.170
7.163
7.159
7.155
7.150
7.146
7.139
6.786
6.780
6.775
6.766
6.762
6.755
5.623
5.603
5.588
5.569
5.159
5.139
5.136
5.105
5.102
5.078
5.076
5.058
5.056
3.665
3.215
3.199
3.187
3.171
3.100
3.087
3.072
3.059
-0.000
NO2
F
CN
H3CO
Chemical Formula: C18H15FN2O3
Molecular Weight: 326.3274
3.00
2.15
1.96
2.06
1.16
0.90
0.92
0.91
0.92
0.92
10
8
6
4
2
0 PPM
USER: nmrsu -- DATE: Sat Aug 19 10:02:41 2017
F1: 500.133
F2: 1.000
SW1: 10000
OF1: 3024.3
PTS1d: 32768 , 32768
EX: zg30
PW: 10.0 us
PD: 1.0 sec
NA: 32
LB: 0.0
Nuts - $glr-1-102 HC_1

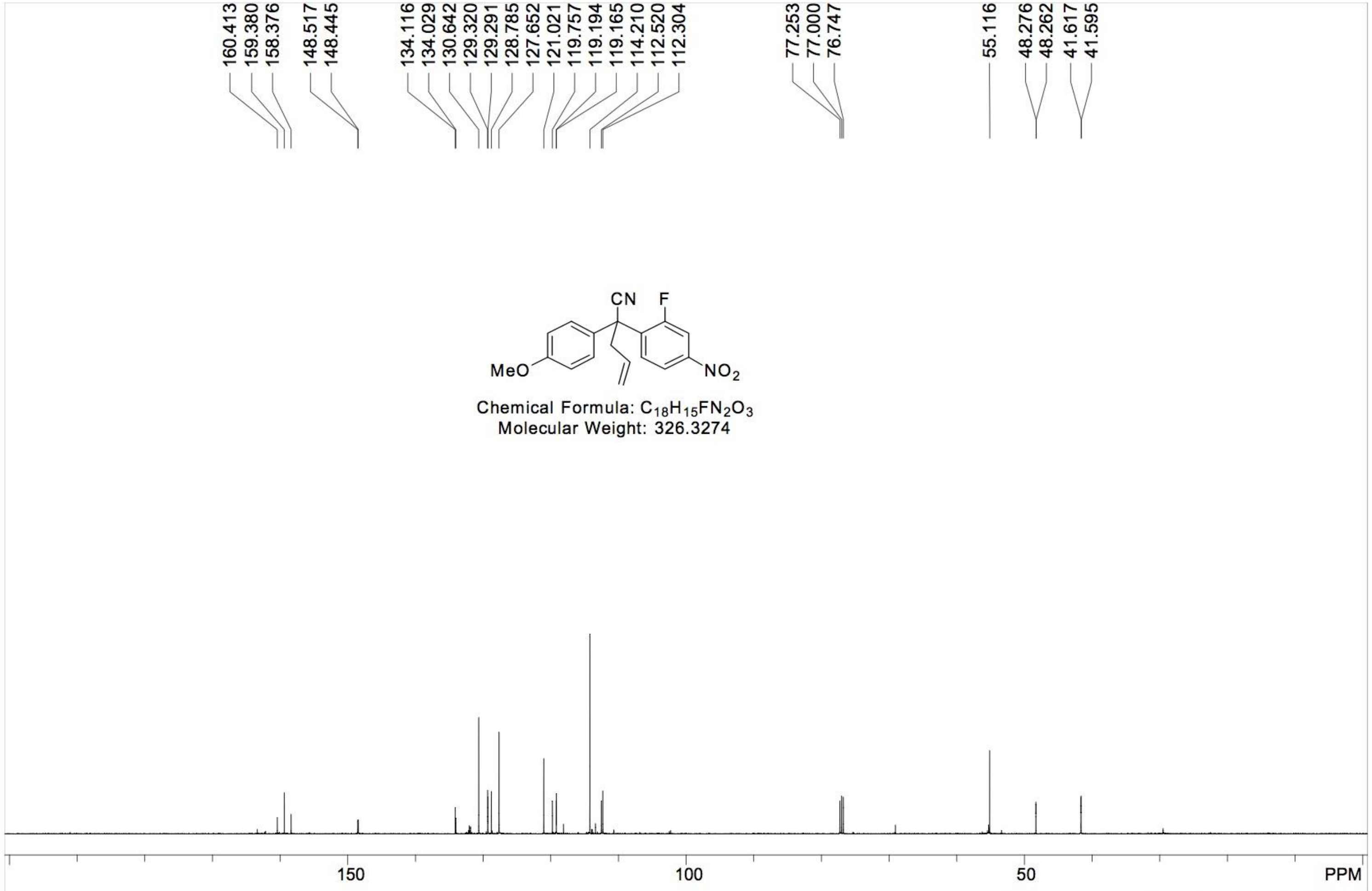

Chemical Formula: $C_{18}H_{15}FN_2O_3$
Molecular Weight: 326.3274

USER: nmrsu -- DATE: Sat Aug 19 10:10:31 2017

F1: 125.770	F2: 1.000	SW1: 29762		OF1: 12547.6		PTS1d: 32768 , 32768	
EX: zgpg30		PW: 10.0 us	PD: 2.0 sec	NA: 128	LB: 0.2		Nuts - $glr-1-102_HC_2

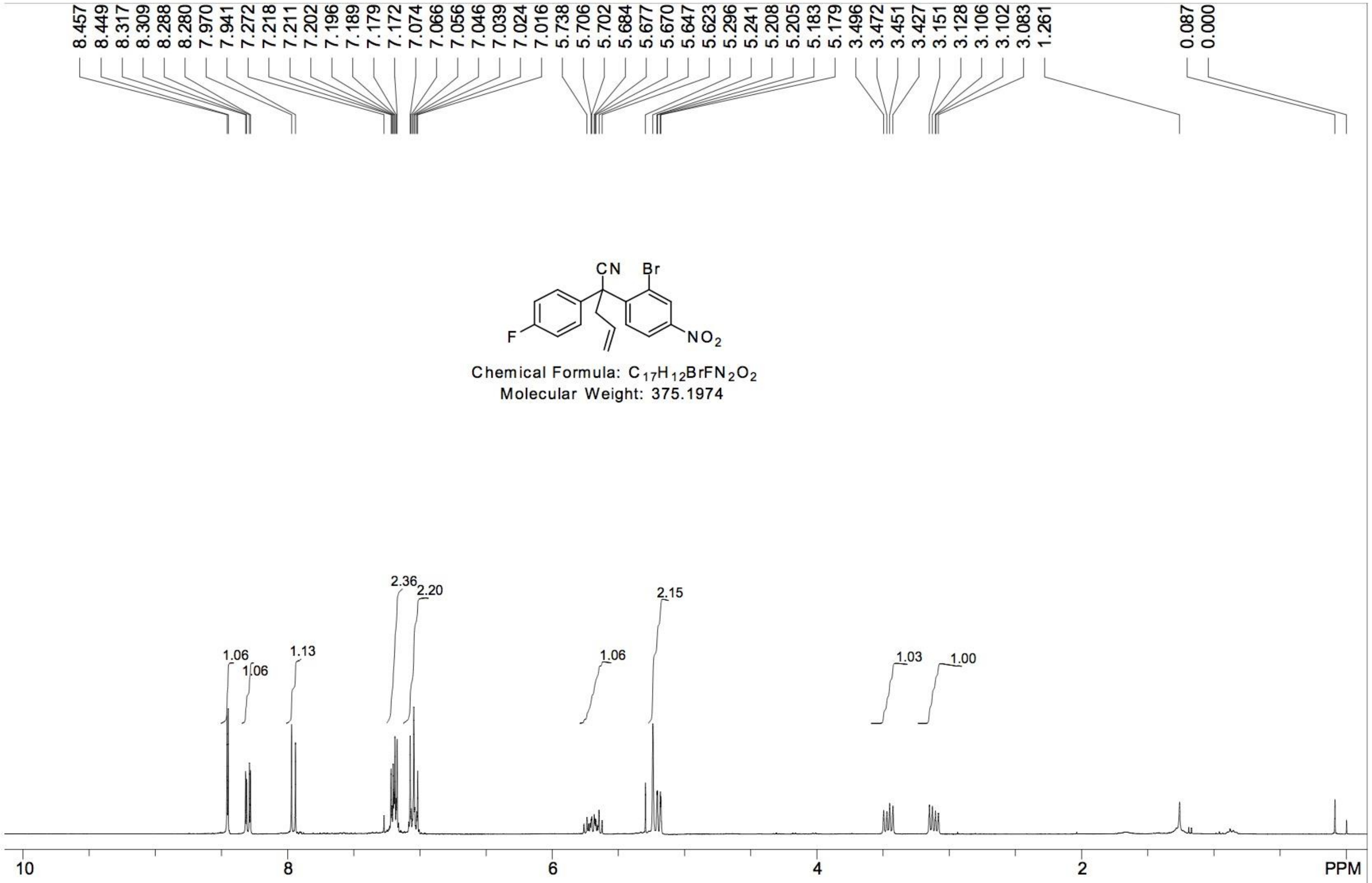

AV300 1H 20170708 LFT-1-21, , USER: Administr -- DATE: Sat Jul 08 04:03:30 2017

F1: 300.132	F2: 1.000	SW1: 6173		OF1: 1852.1		PTS1d: 32768 , 32768	
EX: zg30		PW: 12.0 us	PD: 1.0 sec	NA: 8	LB: 0.0		Nuts - $NMR_3

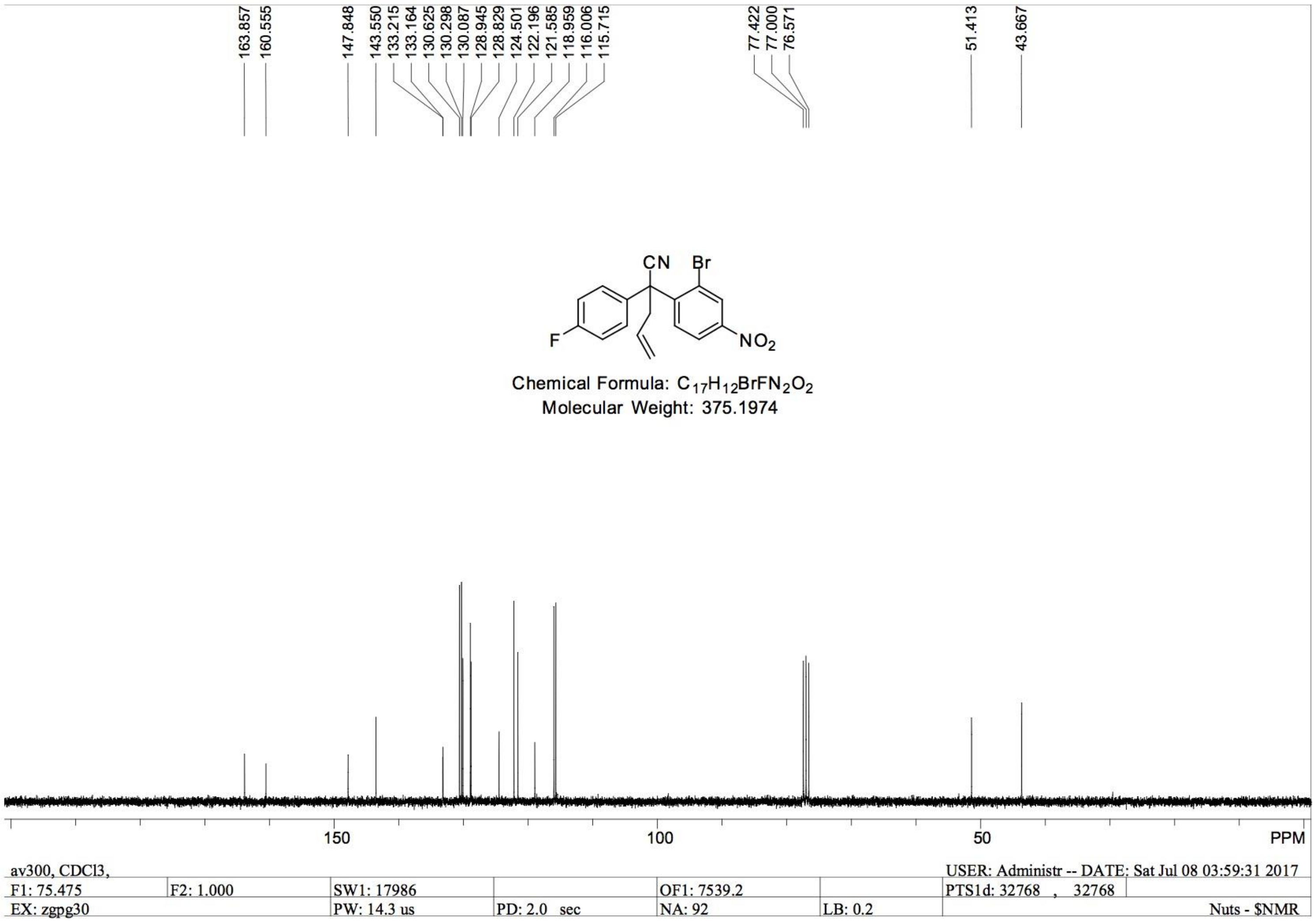
163.857
160.555
147.848
143.550
133.215
133.164
130.625
130.298
130.087
128.945
128.829
124.501
122.196
121.585
118.959
116.006
115.715
77.422
77.000
76.571
51.413
43.667
CN
Br
F
NO2
Chemical Formula: C17H12BrFN2O2
Molecular Weight: 375.1974
150
100
50
PPM
av300, CDCl3,
USER: Administr -- DATE: Sat Jul 08 03:59:31 2017
F1: 75.475
F2: 1.000
SW1: 17986
OF1: 7539.2
PTS1d: 32768 , 32768
EX: zgpg30
PW: 14.3 us
PD: 2.0 sec
NA: 92
LB: 0.2
Nuts - $NMR

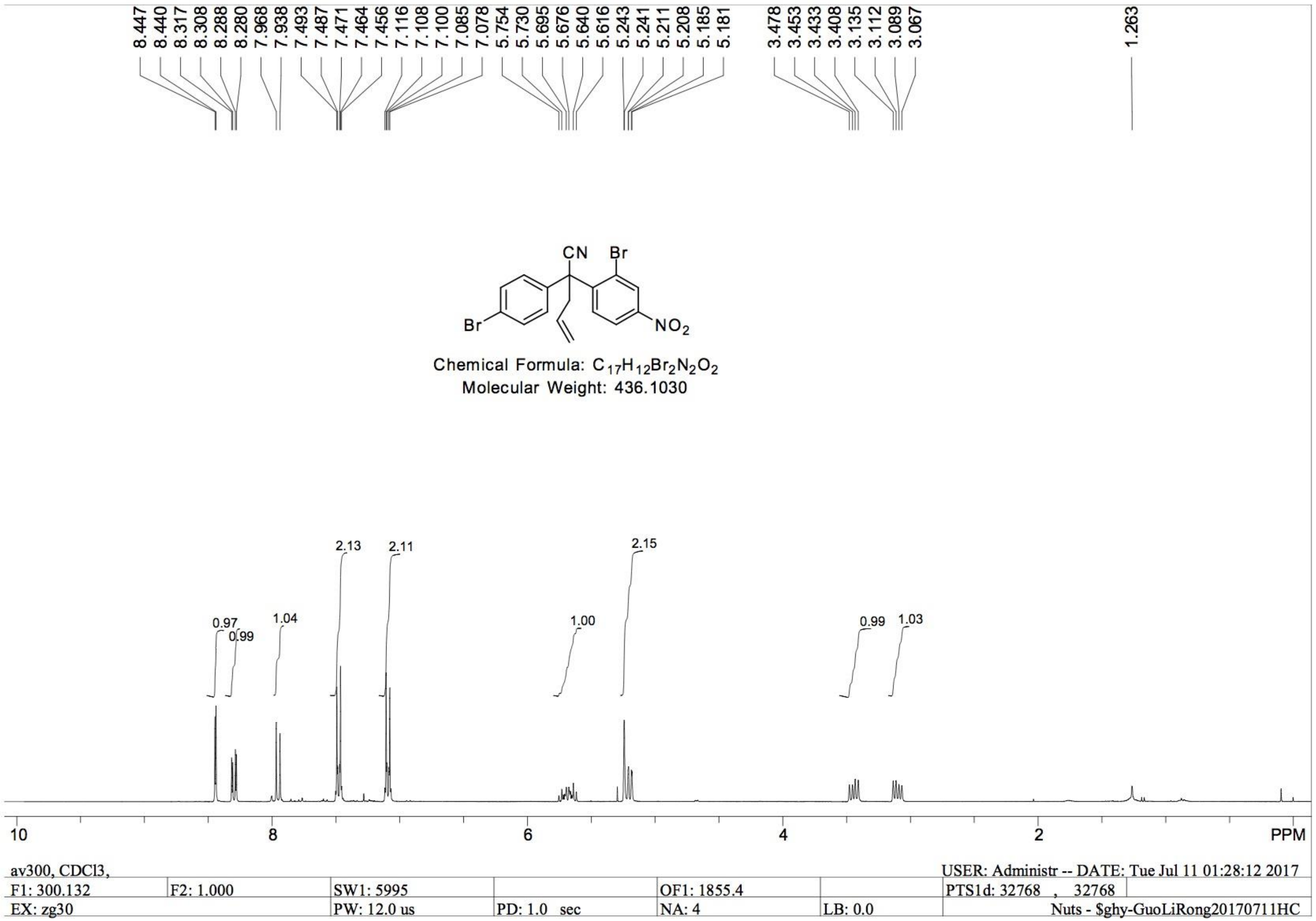
8.447 8.440 8.317 8.308 8.288 8.280 7.968 7.938 7.493 7.487 7.471 7.464 7.456 7.116 7.108 7.100 7.085 7.078 5.754 5.730 5.695 5.676 5.640 5.616 5.243 5.241 5.211 5.208 5.185 5.181 3.478 3.453 3.433 3.408 3.135 3.112 3.089 3.067 1.263
CN
Br
Br
NO2
Chemical Formula: C17H12Br2N2O2
Molecular Weight: 436.1030
0.97 0.99 1.04 2.13 2.11 1.00 2.15 0.99 1.03
10 8 6 4 2 PPM
av300, CDCl3,
USER: Administr -- DATE: Tue Jul 11 01:28:12 2017
F1: 300.132
F2: 1.000
SW1: 5995
OF1: 1855.4
PTS1d: 32768 , 32768
EX: zg30
PW: 12.0 us
PD: 1.0 sec
NA: 4
LB: 0.0
Nuts - $ghy-GuoLiRong20170711HC

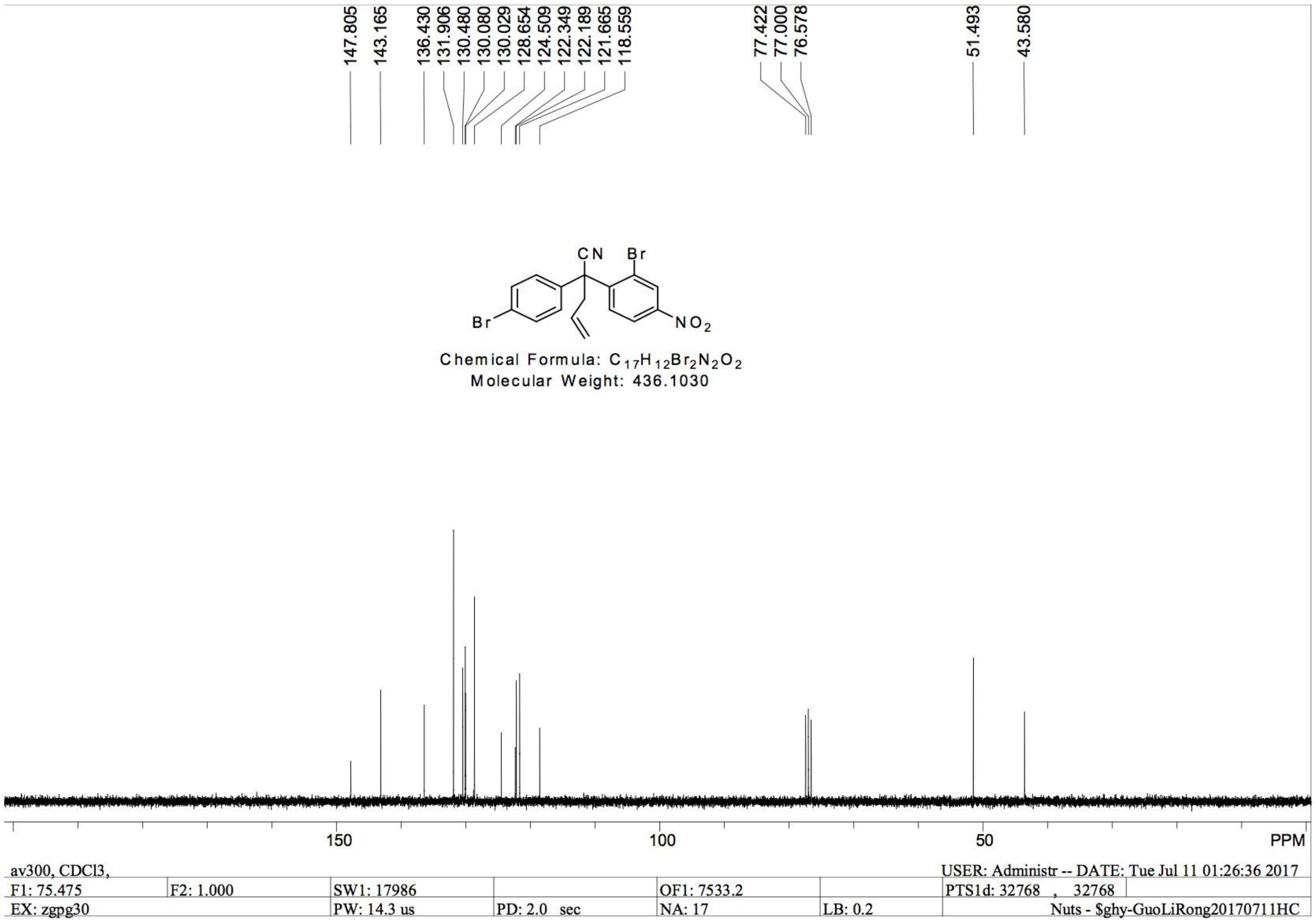
147.805
143.165
136.430
131.906
130.480
130.080
130.029
128.654
124.509
122.349
122.189
121.665
118.559
77.422
77.000
76.578
51.493
43.580
CN
Br
Br
NO2
Chemical Formula: C17H12Br2N2O2
Molecular Weight: 436.1030
150
100
50
PPM
av300, CDCl3,
USER: Administr -- DATE: Tue Jul 11 01:26:36 2017
F1: 75.475
F2: 1.000
SW1: 17986
OF1: 7533.2
PTS1d: 32768 , 32768
EX: zgpg30
PW: 14.3 us
PD: 2.0 sec
NA: 17
LB: 0.2
Nuts - $ghy-GuoLiRong20170711HC

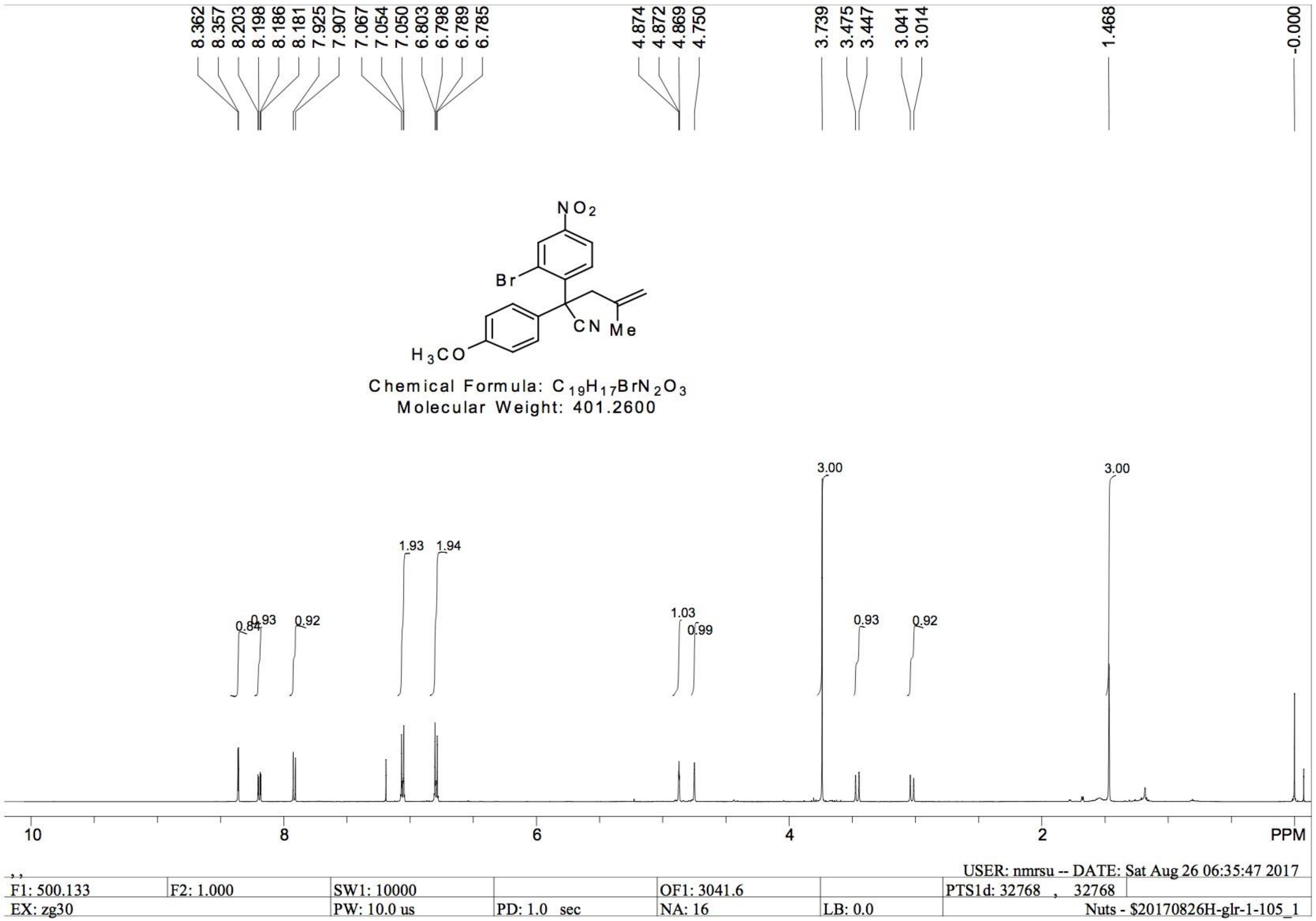
8.362
8.357
8.203
8.198
8.186
8.181
7.925
7.907
7.067
7.054
7.050
6.803
6.798
6.789
6.785
4.874
4.872
4.869
4.750
3.739
3.475
3.447
3.041
3.014
1.468
-0.000
NO2
Br
CN
Me
H3CO
Chemical Formula: C19H17BrN2O3
Molecular Weight: 401.2600
0.84
0.93
0.92
1.93
1.94
1.03
0.99
3.00
0.93
0.92
3.00
10
8
6
4
2
PPM
USER: nmrsu -- DATE: Sat Aug 26 06:35:47 2017
F1: 500.133
F2: 1.000
SW1: 10000
OF1: 3041.6
PTS1d: 32768 , 32768
EX: zg30
PW: 10.0 us
PD: 1.0 sec
NA: 16
LB: 0.0
Nuts - $20170826H-glr-1-105_1

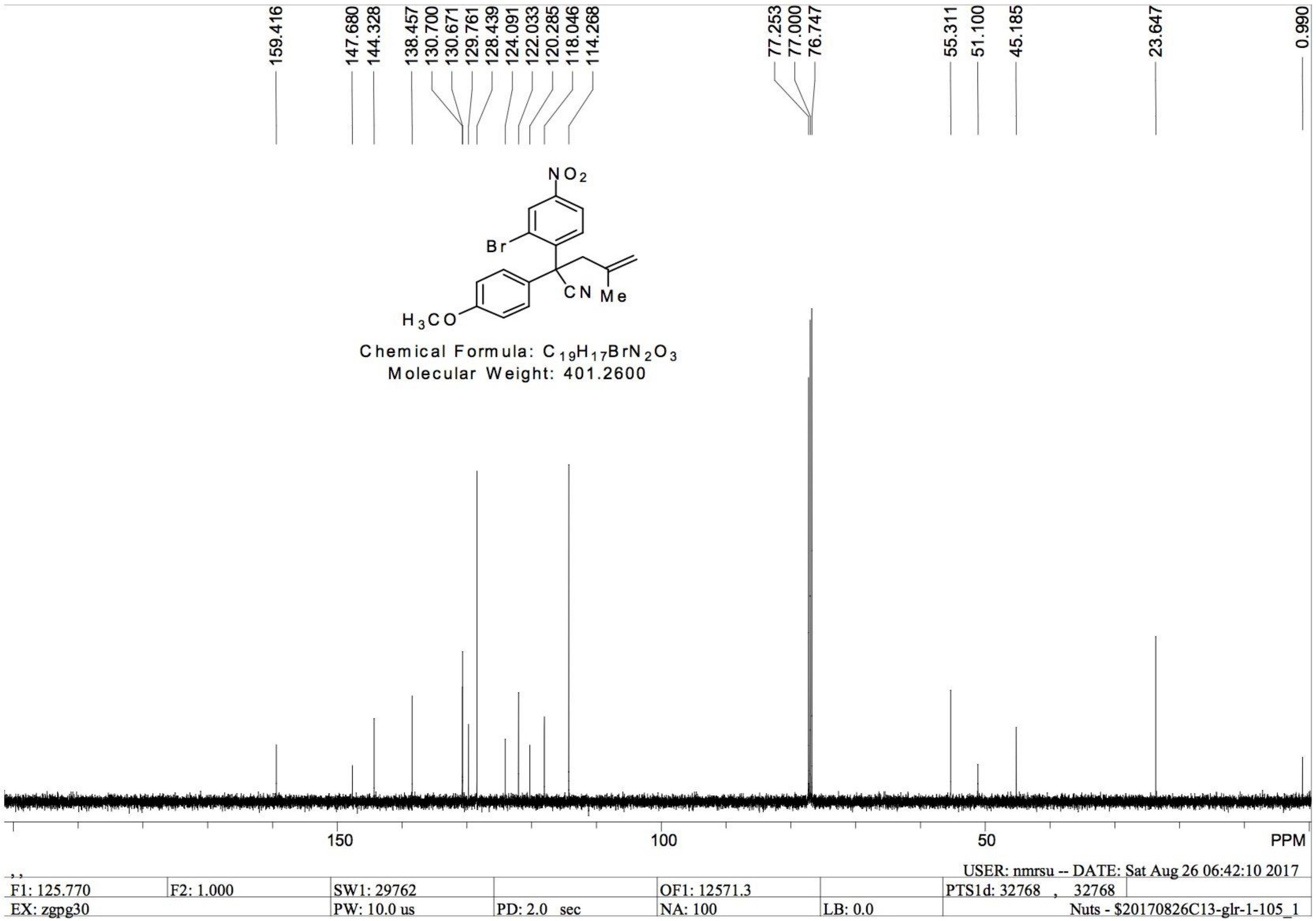

159.416
147.680
144.328
138.457
130.700
130.671
129.761
128.439
124.091
122.033
120.285
118.046
114.268
77.253
77.000
76.747
55.311
51.100
45.185
23.647
0.990
NO2
Br
CN
Me
H3CO
Chemical Formula: C19H17BrN2O3
Molecular Weight: 401.2600
150
100
50
PPM
USER: nmrsu -- DATE: Sat Aug 26 06:42:10 2017
F1: 125.770
F2: 1.000
SW1: 29762
OF1: 12571.3
PTS1d: 32768 , 32768
EX: zgpg30
PW: 10.0 us
PD: 2.0 sec
NA: 100
LB: 0.0
Nuts - $20170826C13-glr-1-105_1

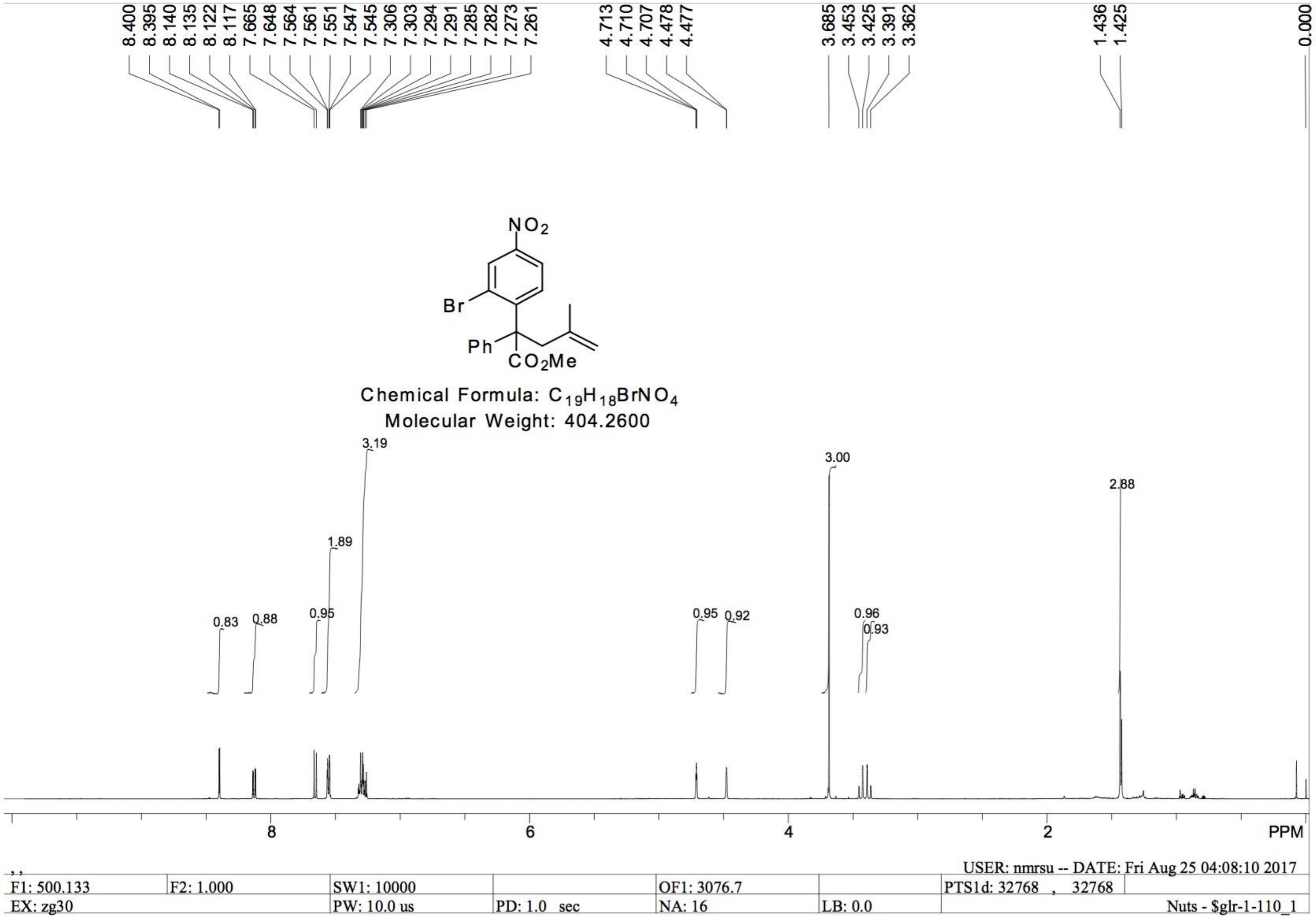

8.400 8.395 8.140 8.135 8.122 8.117 7.665 7.648 7.564 7.561 7.551 7.547 7.545 7.306 7.303 7.294 7.291 7.285 7.282 7.273 7.261
4.713 4.710 4.707 4.478 4.477
3.685 3.453 3.425 3.391 3.362
1.436 1.425
0.000
NO2
Br
Ph
CO2Me
Chemical Formula: C19H18BrNO4
Molecular Weight: 404.2600
0.83 0.88 0.95 1.89 3.19 0.95 0.92 3.00 0.96 0.93 2.88
8 6 4 2 PPM
USER: nmrsu -- DATE: Fri Aug 25 04:08:10 2017
F1: 500.133
F2: 1.000
SW1: 10000
OF1: 3076.7
PTS1d: 32768 , 32768
EX: zg30
PW: 10.0 us
PD: 1.0 sec
NA: 16
LB: 0.0
Nuts - $glr-1-110_1

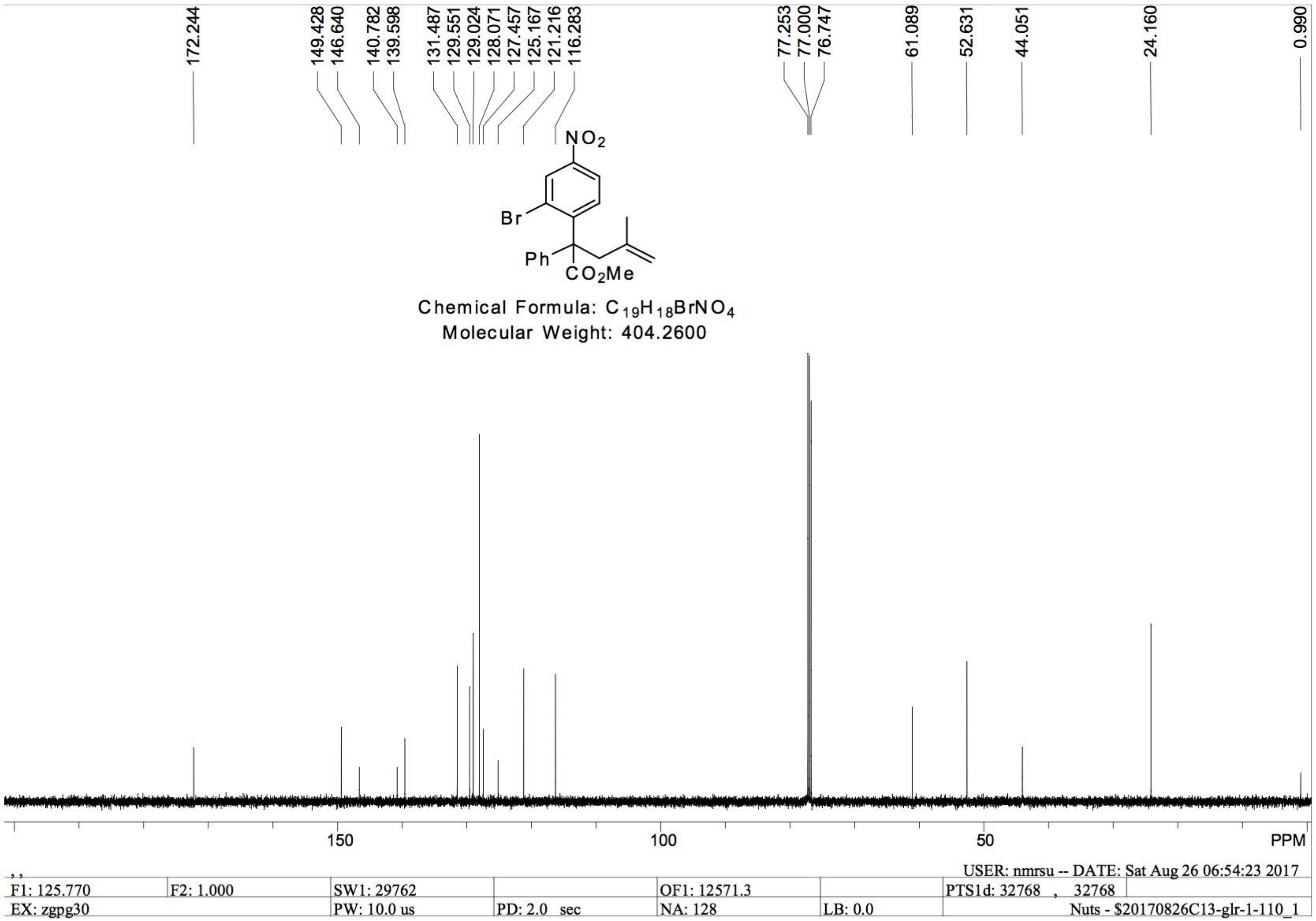

172.244
149.428
146.640
140.782
139.598
131.487
129.551
129.024
128.071
127.457
125.167
121.216
116.283
77.253
77.000
76.747
61.089
52.631
44.051
24.160
0.990
NO2
Br
Ph
CO2Me
Chemical Formula: C19H18BrNO4
Molecular Weight: 404.2600
150
100
50
PPM
USER: nmrsu -- DATE: Sat Aug 26 06:54:23 2017
F1: 125.770
F2: 1.000
SW1: 29762
OF1: 12571.3
PTS1d: 32768 , 32768
EX: zgpg30
PW: 10.0 us
PD: 2.0 sec
NA: 128
LB: 0.0
Nuts - $20170826C13-glr-1-110_1

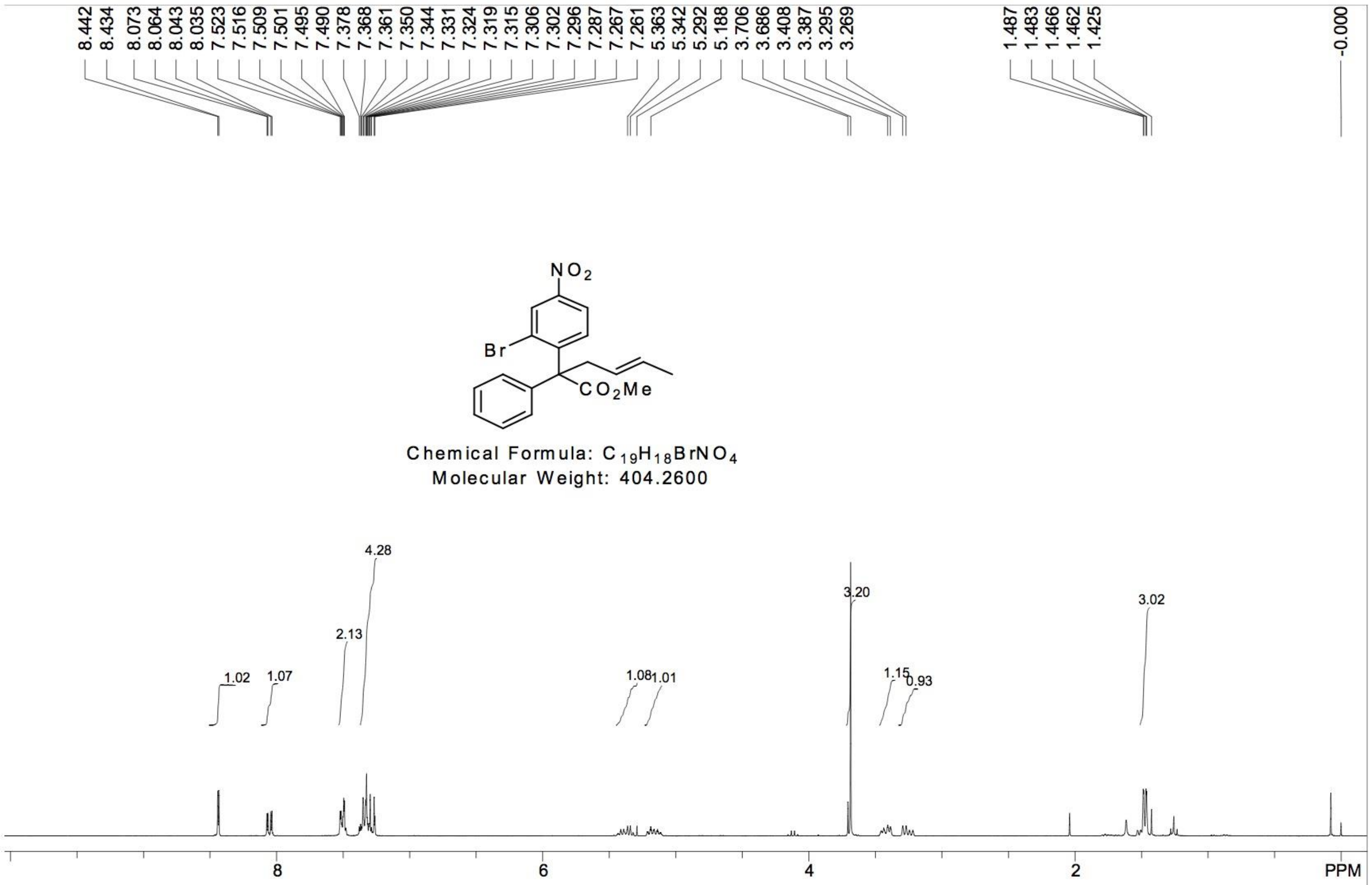

AV300 1H 20170731 GLR-1-73, , USER: Administr -- DATE: Thu Aug 03 00:50:07 2017

F1: 300.132	F2: 1.000	SW1: 6173		OF1: 1848.9		PTS1d: 32768 , 32768	
EX: zg30		PW: 12.0 us	PD: 1.0 sec	NA: 9	LB: 0.0	Nuts - $ghy-LiuFengTing20170731HC-GLR-1-73_1	

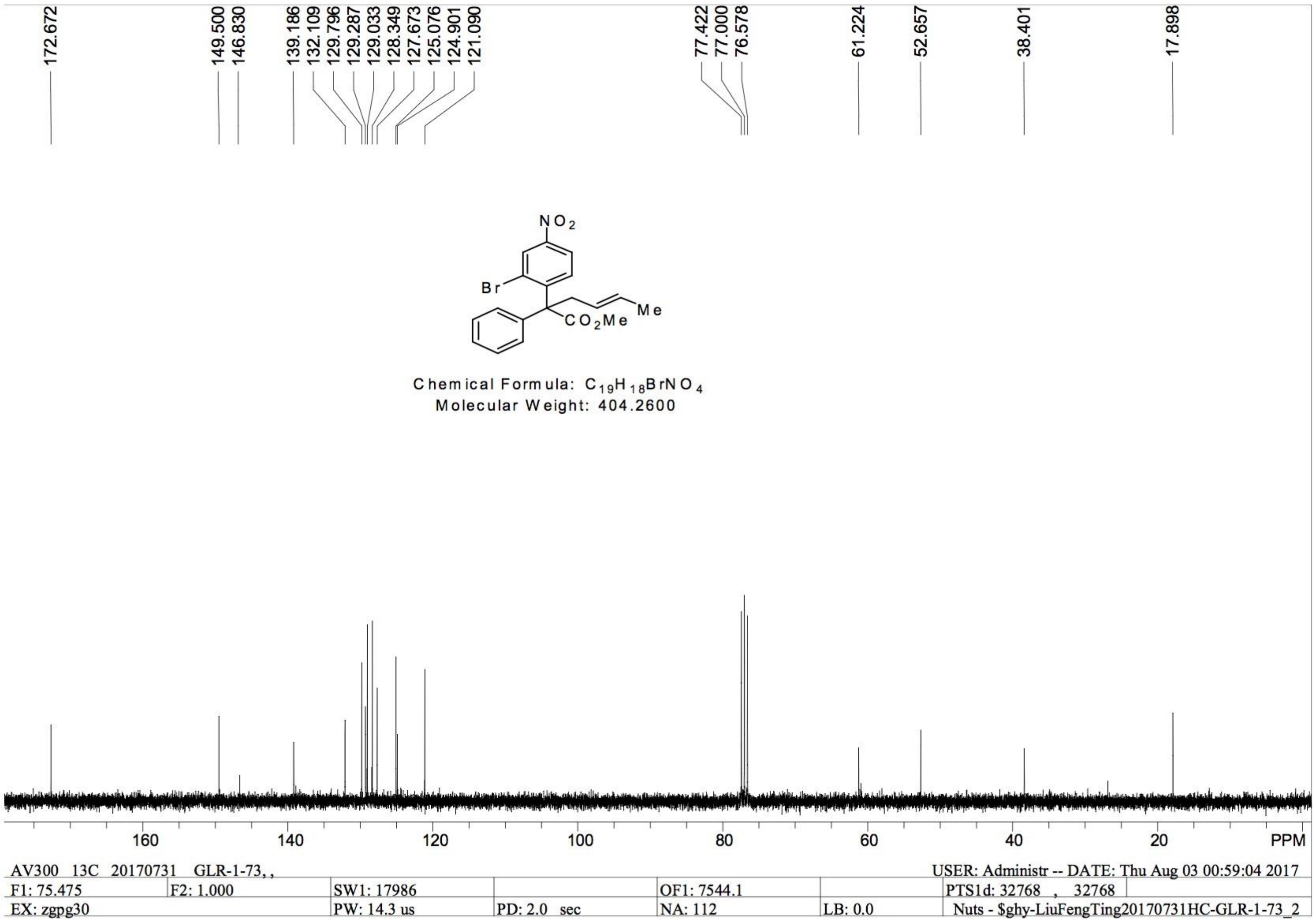

172.672
149.500
146.830
139.186
132.109
129.796
129.287
129.033
128.349
127.673
125.076
124.901
121.090
77.422
77.000
76.578
61.224
52.657
38.401
17.898
NO2
Br
CO2Me
Me
Chemical Formula: C19H18BrNO4
Molecular Weight: 404.2600
160
140
120
100
80
60
40
20
PPM
AV300 13C 20170731 GLR-1-73, ,
USER: Administr -- DATE: Thu Aug 03 00:59:04 2017
F1: 75.475
F2: 1.000
SW1: 17986
OF1: 7544.1
PTS1d: 32768 , 32768
EX: zgpg30
PW: 14.3 us
PD: 2.0 sec
NA: 112
LB: 0.0
Nuts - $ghy-LiuFengTing20170731HC-GLR-1-73_2

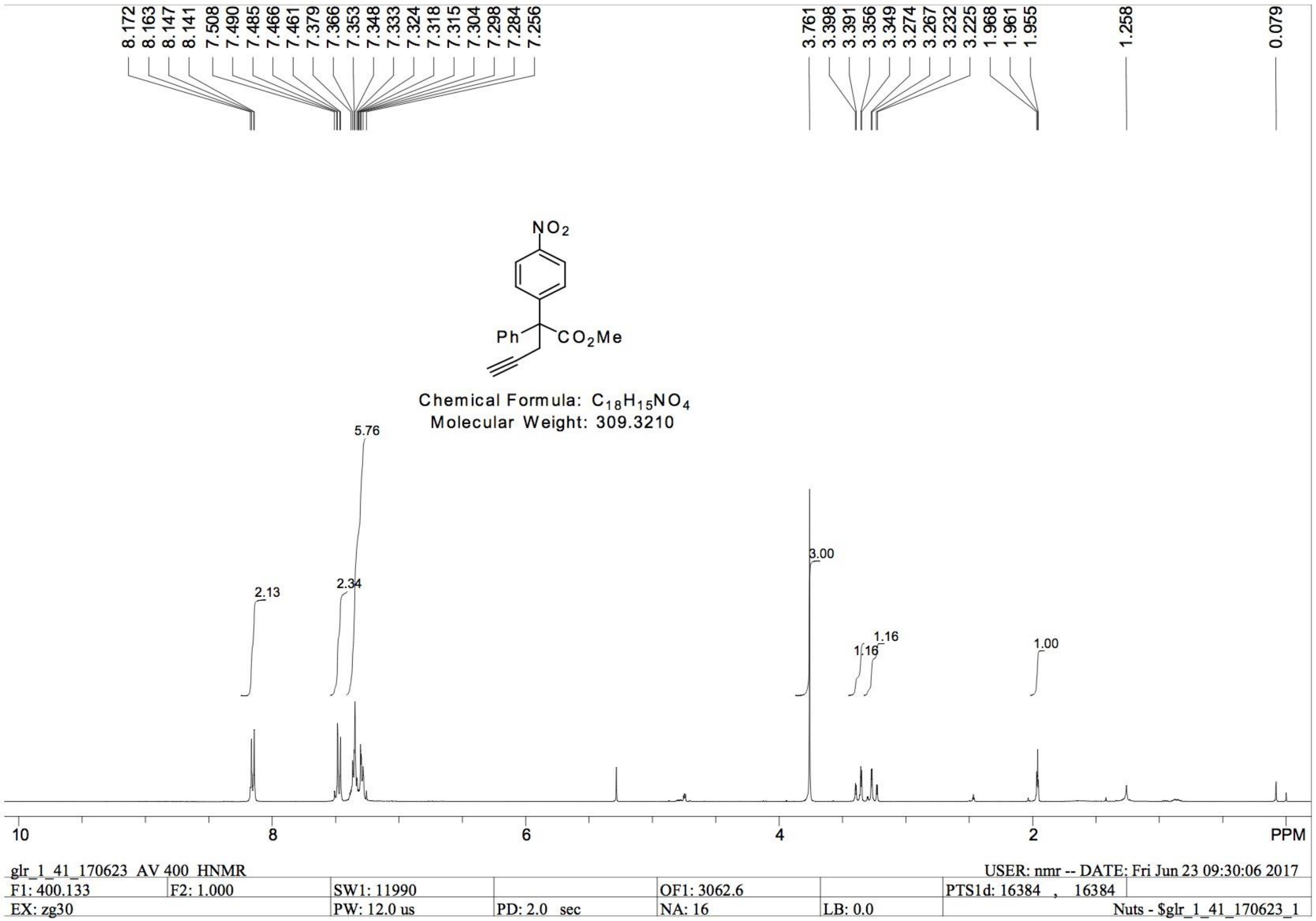
8.172 8.163 8.147 8.141 7.508 7.490 7.485 7.466 7.461 7.379 7.366 7.353 7.348 7.333 7.324 7.318 7.315 7.304 7.298 7.284 7.256
3.761 3.398 3.391 3.356 3.349 3.274 3.267 3.232 3.225 1.968 1.961 1.955
1.258
0.079
NO2
Ph
CO2Me
Chemical Formula: C18H15NO4
Molecular Weight: 309.3210
5.76
2.13
2.34
3.00
1.16
1.16
1.00
10
8
6
4
2
PPM
glr_1_41_170623 AV 400 HNMR
USER: nmr -- DATE: Fri Jun 23 09:30:06 2017
F1: 400.133
F2: 1.000
SW1: 11990
OF1: 3062.6
PTS1d: 16384 , 16384
EX: zg30
PW: 12.0 us
PD: 2.0 sec
NA: 16
LB: 0.0
Nuts - $glr_1_41_170623_1

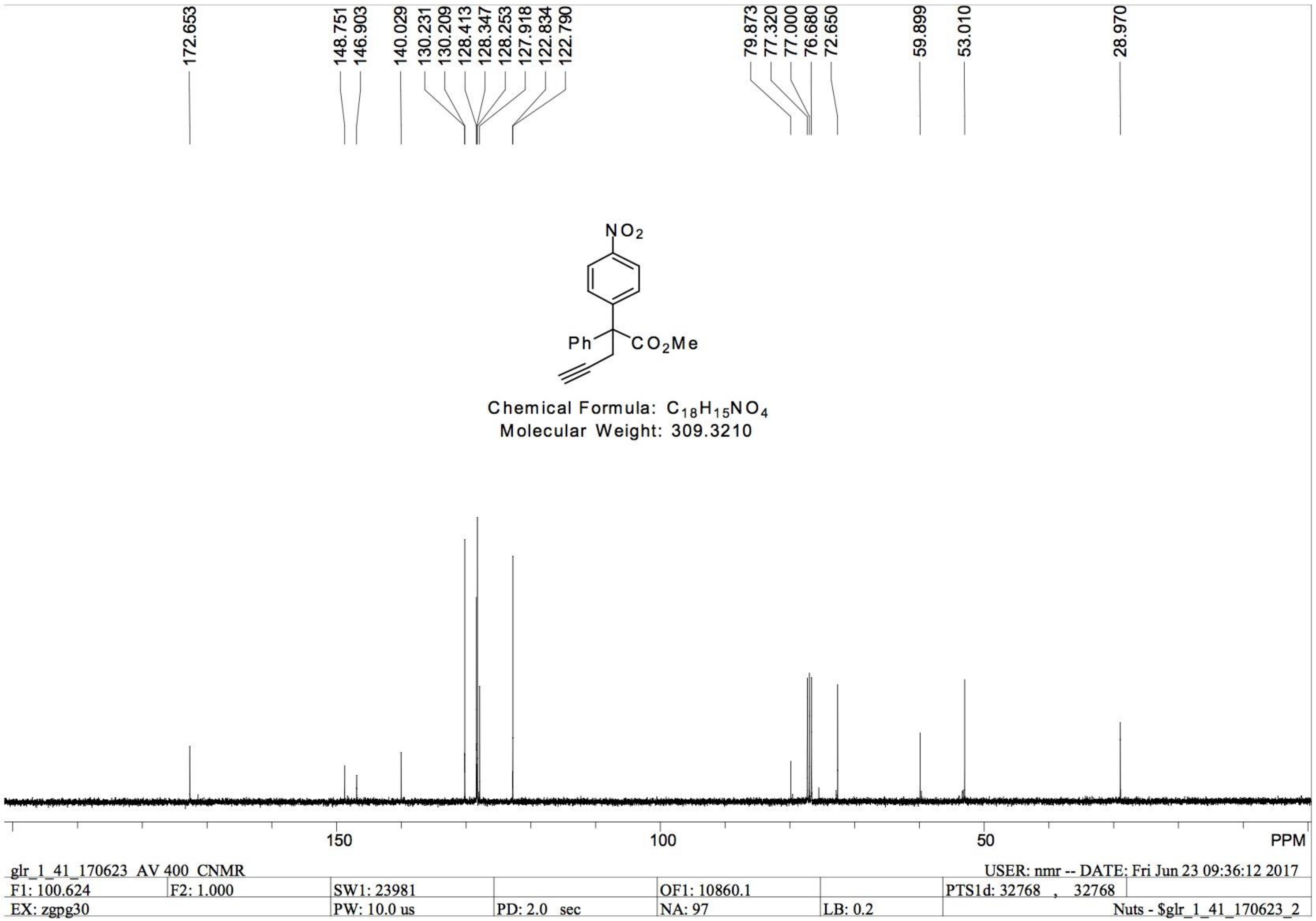
172.653
148.751
146.903
140.029
130.231
130.209
128.413
128.347
128.253
127.918
122.834
122.790
79.873
77.320
77.000
76.680
72.650
59.899
53.010
28.970
NO2
Ph
CO2Me
Chemical Formula: C18H15NO4
Molecular Weight: 309.3210
150
100
50
PPM
glr_1_41_170623 AV 400 CNMR
USER: nmr -- DATE: Fri Jun 23 09:36:12 2017
F1: 100.624
F2: 1.000
SW1: 23981
OF1: 10860.1
PTS1d: 32768 , 32768
EX: zgpg30
PW: 10.0 us
PD: 2.0 sec
NA: 97
LB: 0.2
Nuts - $glr_1_41_170623_2

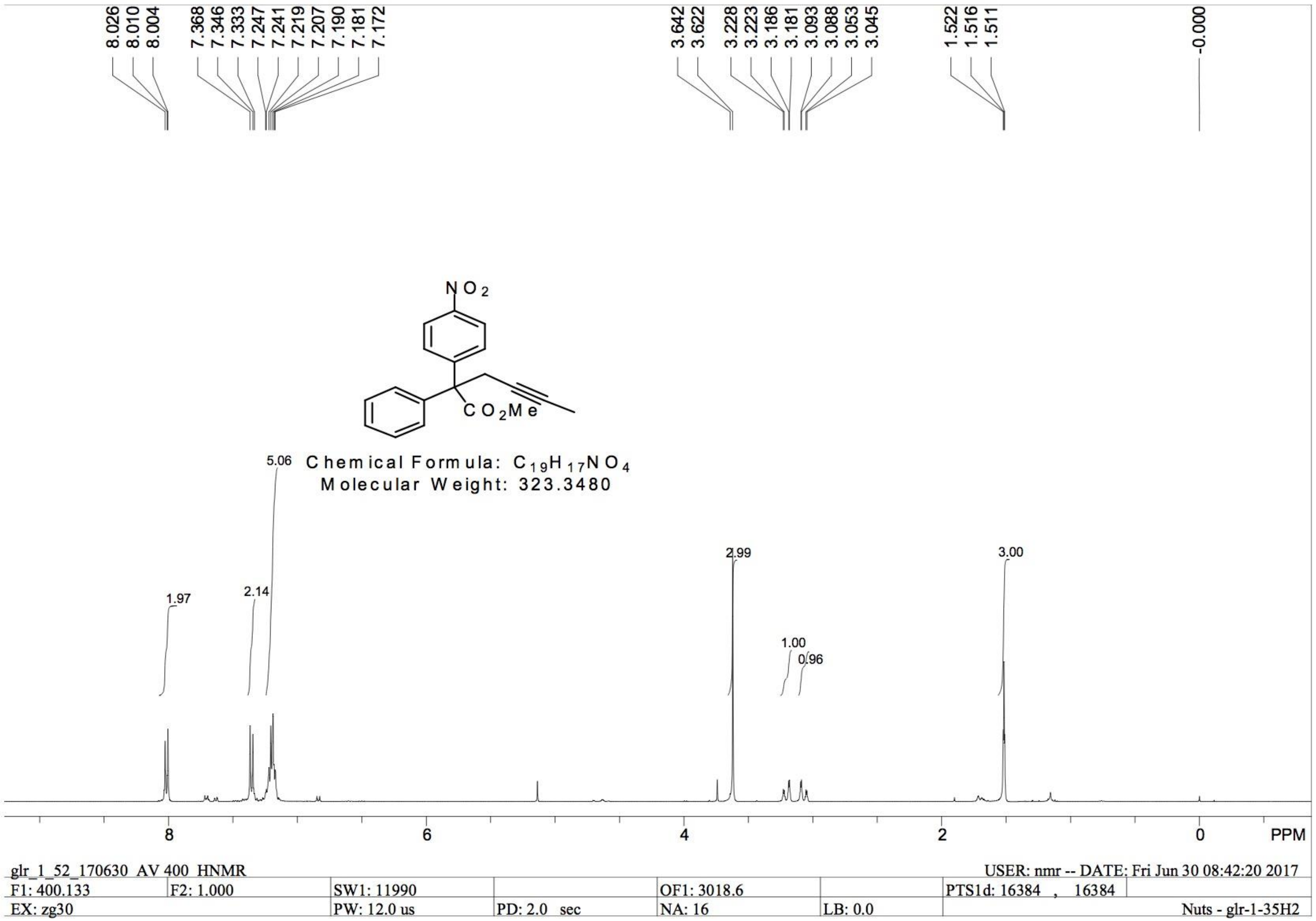
8.026
8.010
8.004
7.368
7.346
7.333
7.247
7.241
7.219
7.207
7.190
7.181
7.172
3.642
3.622
3.228
3.223
3.186
3.181
3.093
3.088
3.053
3.045
1.522
1.516
1.511
-0.000
NO2
CO2Me
Chemical Formula: C19H17NO4
Molecular Weight: 323.3480
1.97
2.14
5.06
2.99
1.00
0.96
3.00
8
6
4
2
0
PPM
glr_1_52_170630 AV 400 HNMR
USER: nmr -- DATE: Fri Jun 30 08:42:20 2017
F1: 400.133
F2: 1.000
SW1: 11990
OF1: 3018.6
PTS1d: 16384 , 16384
EX: zg30
PW: 12.0 us
PD: 2.0 sec
NA: 16
LB: 0.0
Nuts - glr-1-35H2

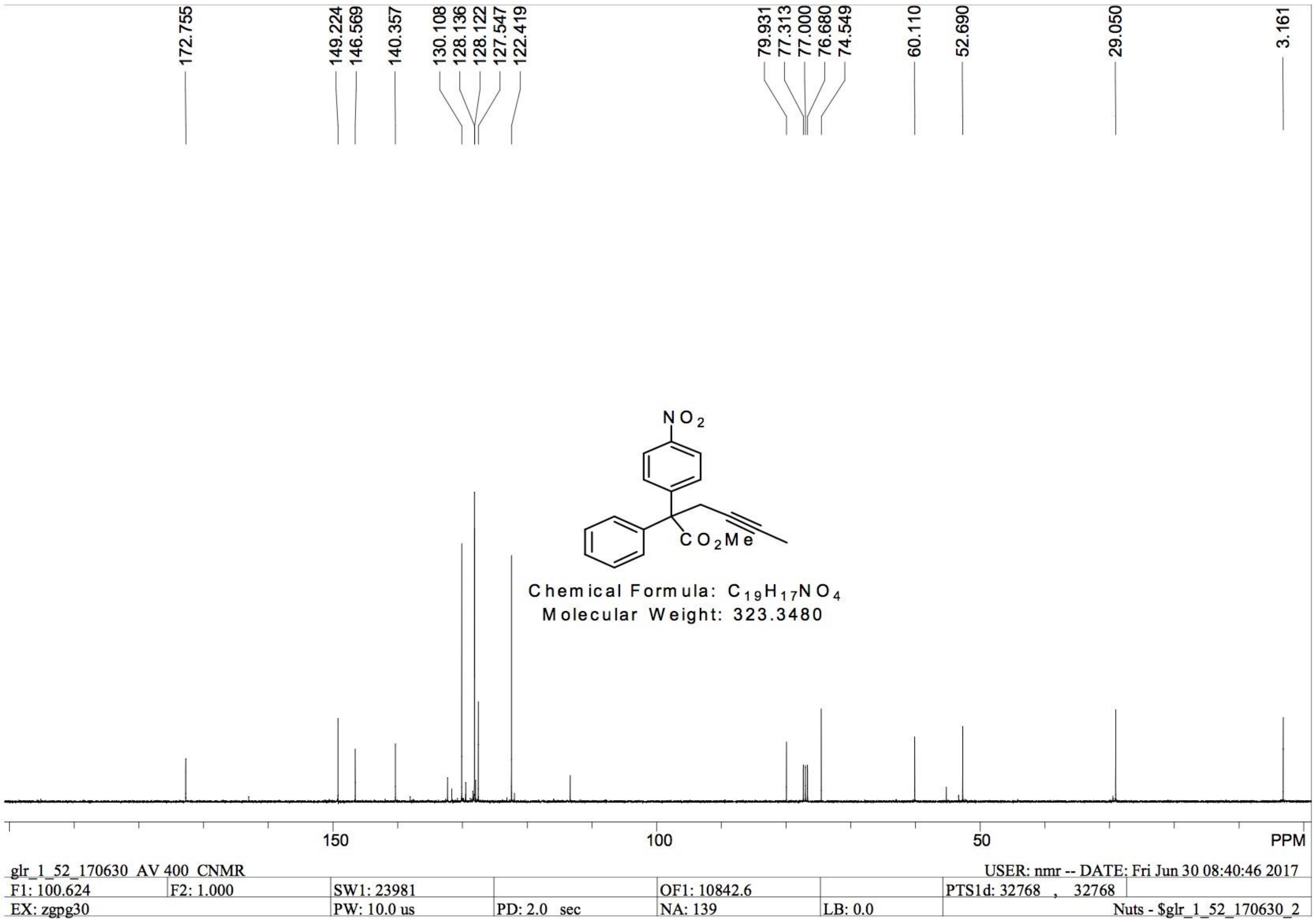

172.755
149.224
146.569
140.357
130.108
128.136
128.122
127.547
122.419
79.931
77.313
77.000
76.680
74.549
60.110
52.690
29.050
3.161
NO2
CO2Me
Chemical Formula: C19H17NO4
Molecular Weight: 323.3480
150
100
50
PPM
glr_1_52_170630 AV 400 CNMR
USER: nmr -- DATE: Fri Jun 30 08:40:46 2017
F1: 100.624
F2: 1.000
SW1: 23981
OF1: 10842.6
PTS1d: 32768 , 32768
EX: zgpg30
PW: 10.0 us
PD: 2.0 sec
NA: 139
LB: 0.0
Nuts - $glr_1_52_170630_2

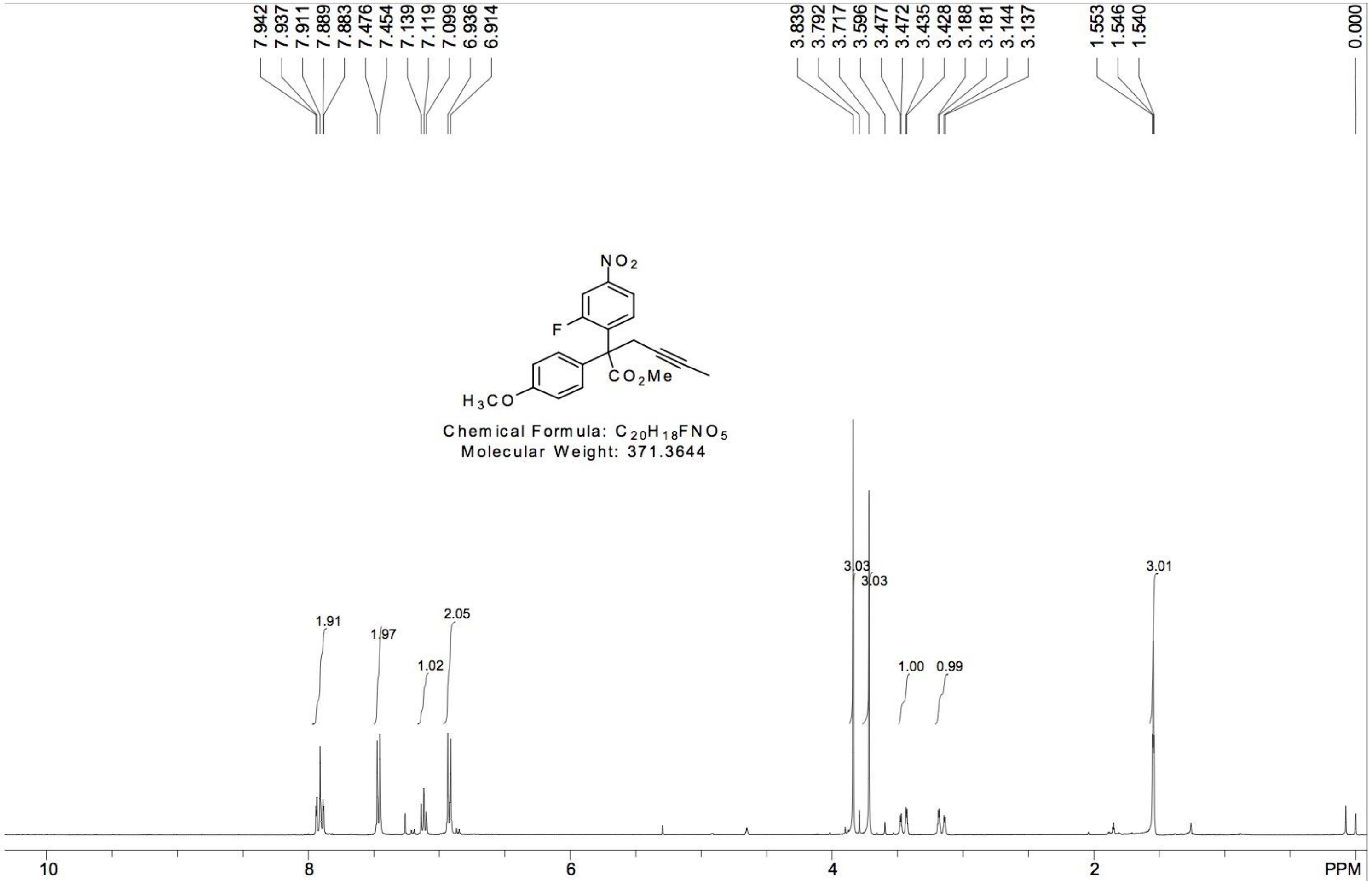

glr_1_48_170630 AV 400 HNMR						USER: nmr -- DATE: Fri Jun 30 09:42:48 2017	
F1: 400.133	F2: 1.000	SW1: 11990		OF1: 3064.8		PTS1d: 16384 , 16384	
EX: zg30		PW: 12.0 us	PD: 2.0 sec	NA: 8	LB: 0.0	Nuts - $glr_1_48_170630_1	

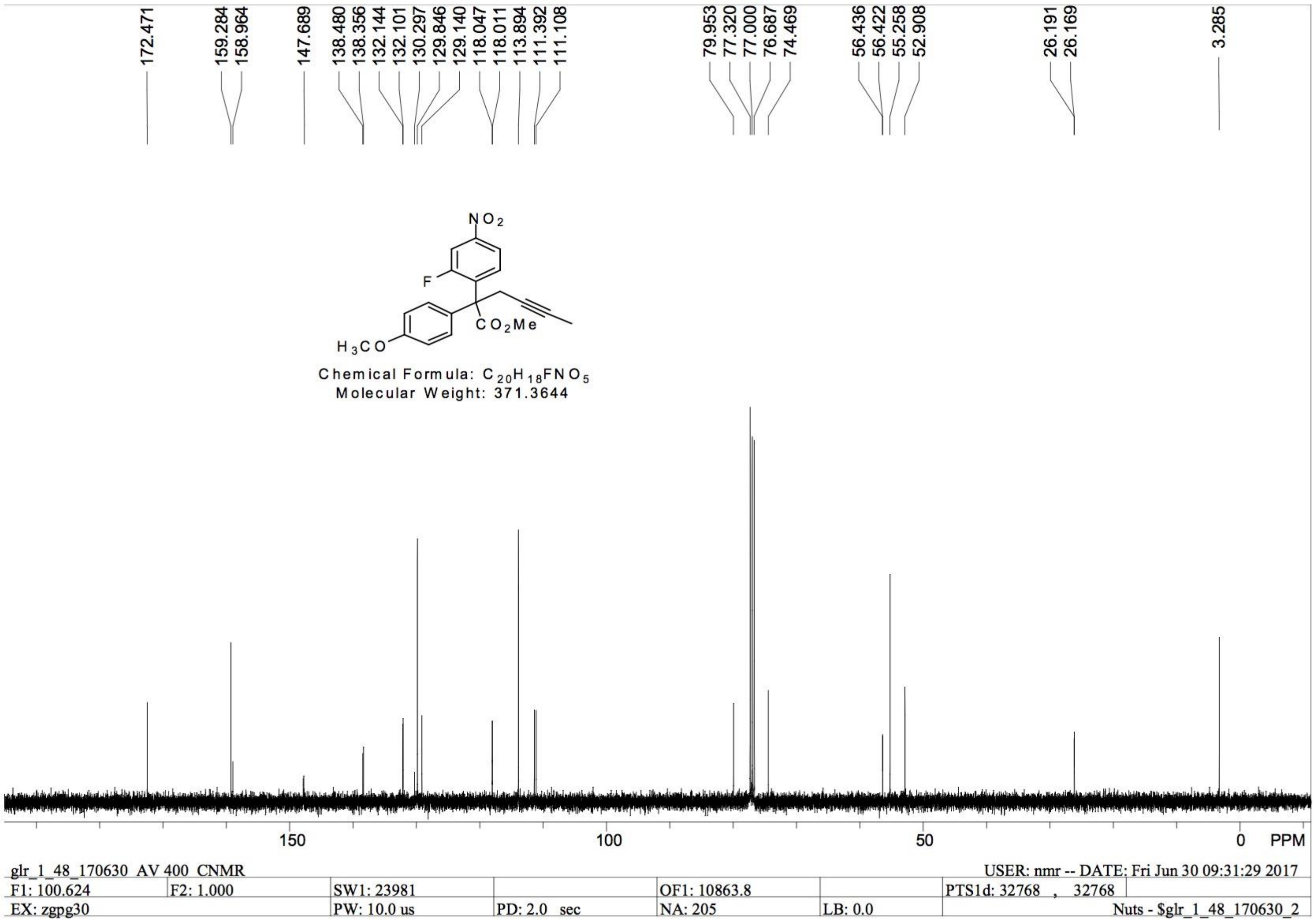
172.471
159.284
158.964
147.689
138.480
138.356
132.144
132.101
130.297
129.846
129.140
118.047
118.011
113.894
111.392
111.108
79.953
77.320
77.000
76.687
74.469
56.436
56.422
55.258
52.908
26.191
26.169
3.285
NO2
F
H3CO
CO2Me
Chemical Formula: C20H18FNO5
Molecular Weight: 371.3644
150
100
50
0
PPM
glr_1_48_170630 AV 400 CNMR
USER: nmr -- DATE: Fri Jun 30 09:31:29 2017
F1: 100.624
F2: 1.000
SW1: 23981
OF1: 10863.8
PTS1d: 32768 , 32768
EX: zgpg30
PW: 10.0 us
PD: 2.0 sec
NA: 205
LB: 0.0
Nuts - $glr_1_48_170630_2

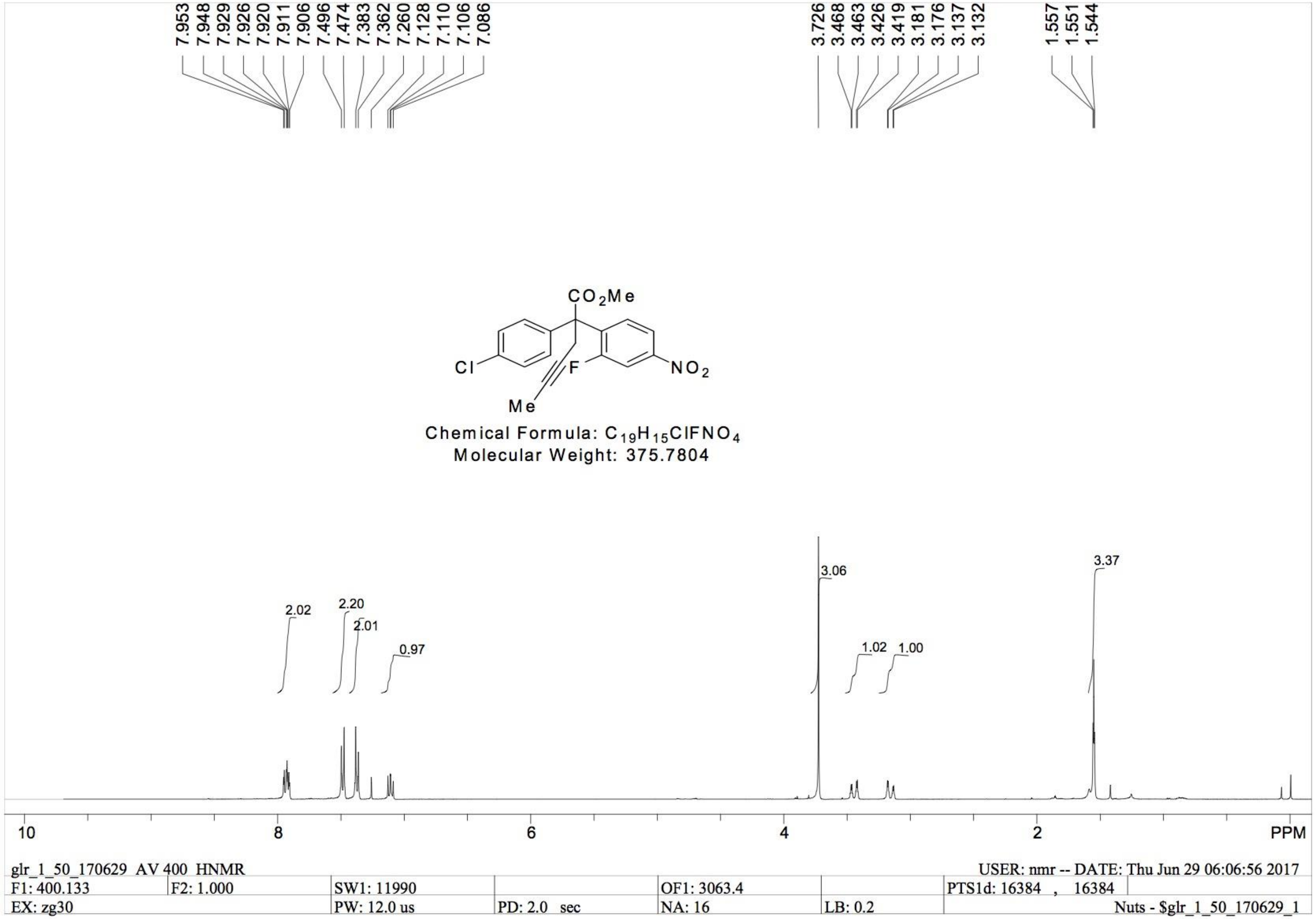
7.953 7.948 7.929 7.926 7.920 7.911 7.906 7.496 7.474 7.383 7.362 7.260 7.128 7.110 7.106 7.086
3.726 3.468 3.463 3.426 3.419 3.181 3.176 3.137 3.132
1.557 1.551 1.544
CO2Me
Cl
F
NO2
Me
Chemical Formula: C19H15ClFNO4
Molecular Weight: 375.7804
2.02
2.20
2.01
0.97
3.06
1.02
1.00
3.37
10
8
6
4
2
PPM
glr_1_50_170629 AV 400 HNMR
USER: nmr -- DATE: Thu Jun 29 06:06:56 2017
F1: 400.133
F2: 1.000
SW1: 11990
OF1: 3063.4
PTS1d: 16384 , 16384
EX: zg30
PW: 12.0 us
PD: 2.0 sec
NA: 16
LB: 0.2
Nuts - $glr_1_50_170629_1

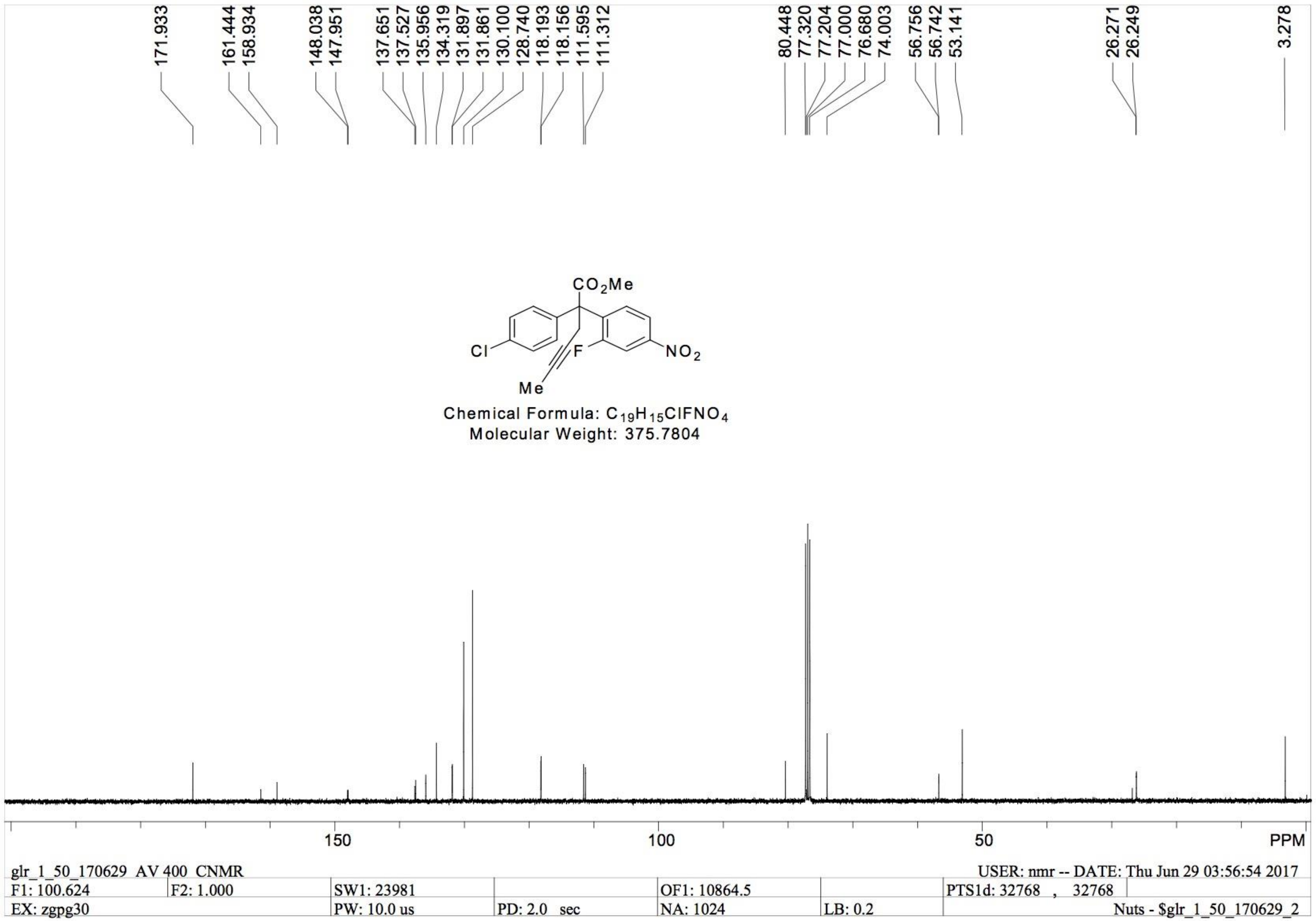
171.933
161.444
158.934
148.038
147.951
137.651
137.527
135.956
134.319
131.897
131.861
130.100
128.740
118.193
118.156
111.595
111.312
80.448
77.320
77.204
77.000
76.680
74.003
56.756
56.742
53.141
26.271
26.249
3.278
CO2Me
Cl
F
NO2
Me
Chemical Formula: C19H15ClFNO4
Molecular Weight: 375.7804
150
100
50
PPM
glr_1_50_170629 AV 400 CNMR
USER: nmr -- DATE: Thu Jun 29 03:56:54 2017
F1: 100.624
F2: 1.000
SW1: 23981
OF1: 10864.5
PTS1d: 32768 , 32768
EX: zgpg30
PW: 10.0 us
PD: 2.0 sec
NA: 1024
LB: 0.2
Nuts - $glr_1_50_170629_2

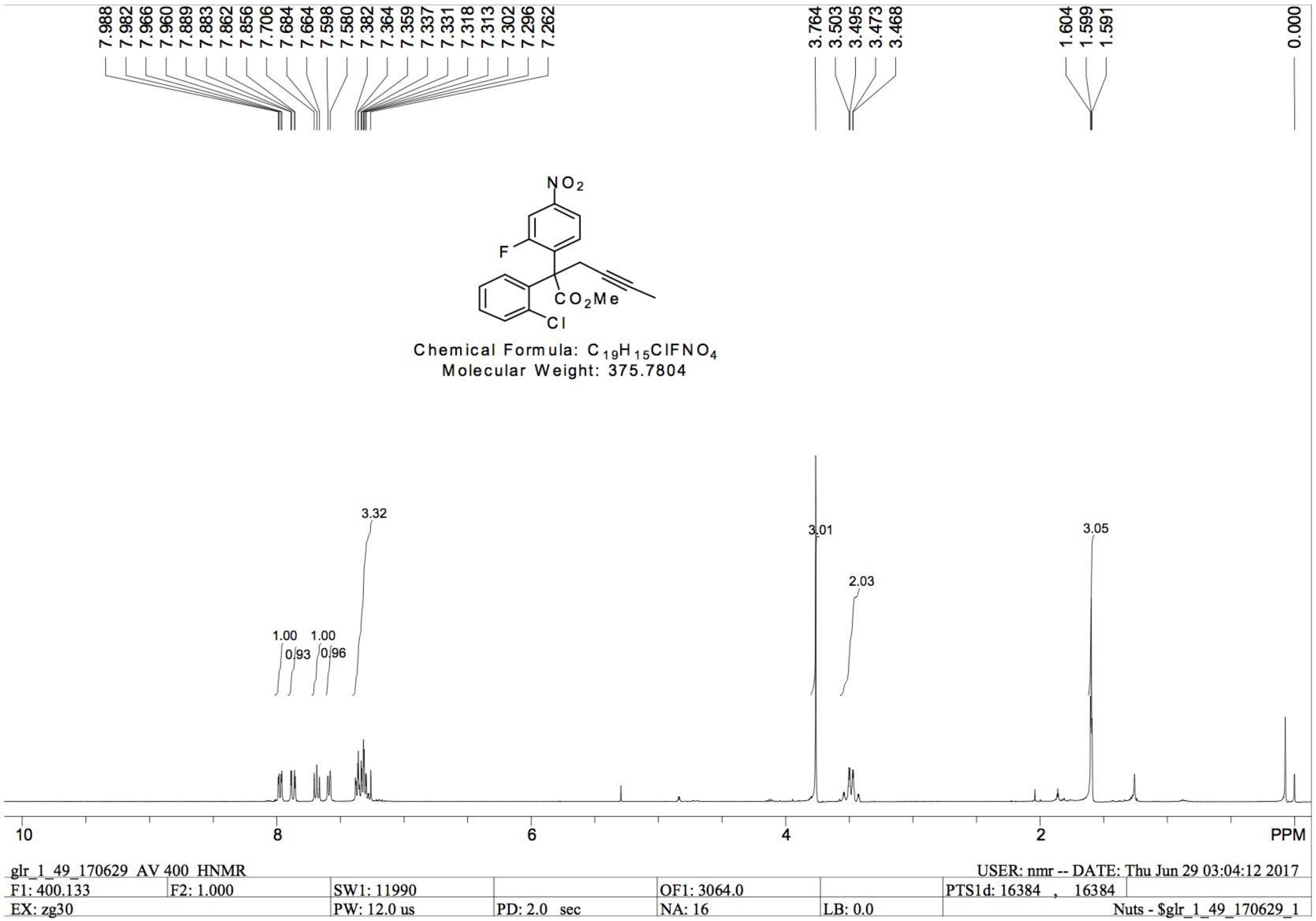
7.988 7.982 7.966 7.960 7.889 7.883 7.862 7.856 7.706 7.684 7.664 7.598 7.580 7.382 7.364 7.359 7.337 7.331 7.318 7.313 7.302 7.296 7.262
3.764 3.503 3.495 3.473 3.468
1.604 1.599 1.591
0.000
NO2
F
CO2Me
Cl
Chemical Formula: C19H15ClFNO4
Molecular Weight: 375.7804
1.00 0.93 1.00 0.96 3.32 3.01 2.03 3.05
10 8 6 4 2 PPM
glr_1_49_170629 AV 400 HNMR
USER: nmr -- DATE: Thu Jun 29 03:04:12 2017
F1: 400.133 F2: 1.000 SW1: 11990 OF1: 3064.0 PTS1d: 16384 , 16384
EX: zg30 PW: 12.0 us PD: 2.0 sec NA: 16 LB: 0.0 Nuts - $glr_1_49_170629_1

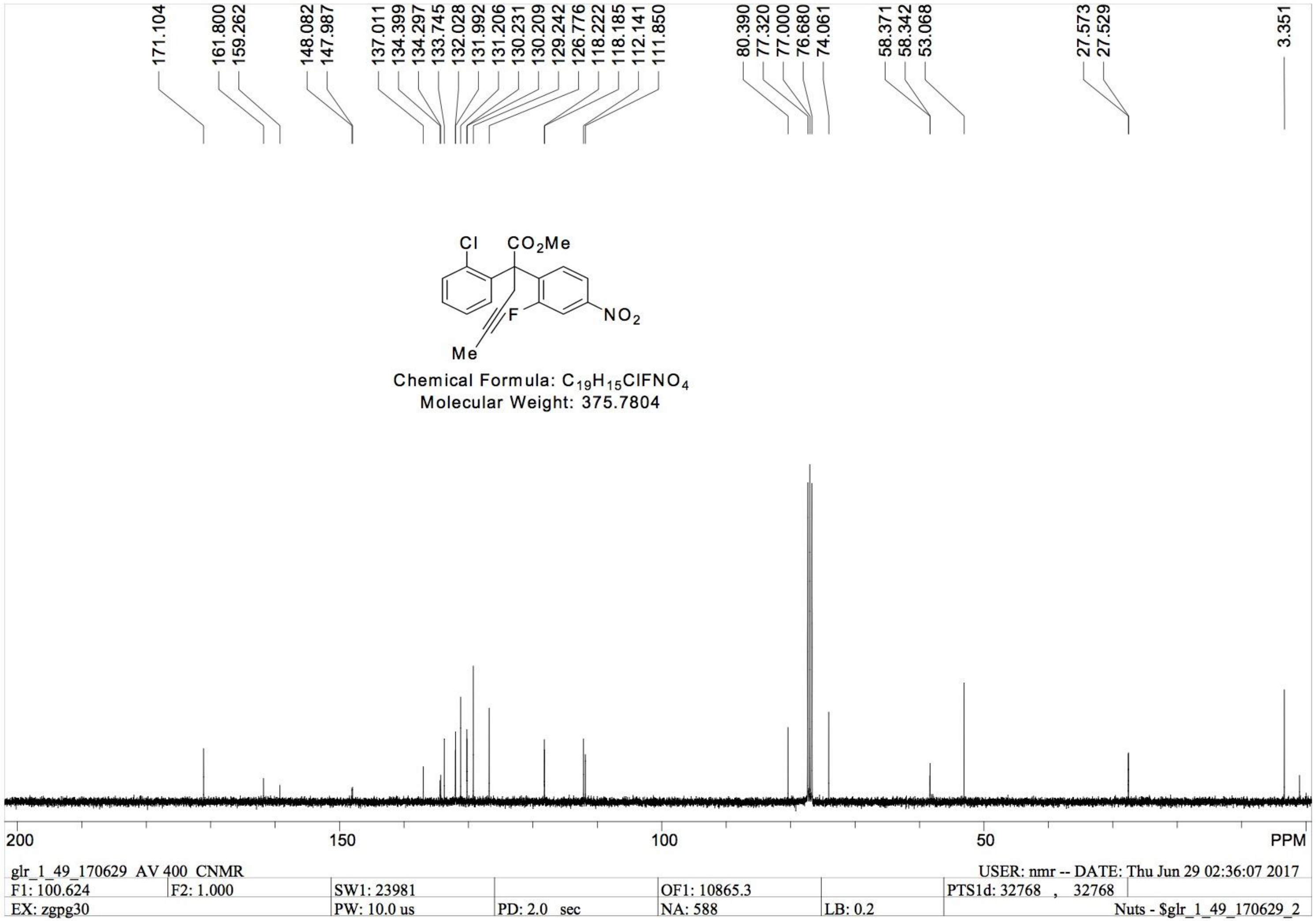
171.104
161.800
159.262
148.082
147.987
137.011
134.399
134.297
133.745
132.028
131.992
131.206
130.231
130.209
129.242
126.776
118.222
118.185
112.141
111.850
80.390
77.320
77.000
76.680
74.061
58.371
58.342
53.068
27.573
27.529
3.351
Cl
CO2Me
F
NO2
Me
Chemical Formula: C19H15ClFNO4
Molecular Weight: 375.7804
200
150
100
50
PPM
glr_1_49_170629 AV 400 CNMR
USER: nmr -- DATE: Thu Jun 29 02:36:07 2017
F1: 100.624
F2: 1.000
SW1: 23981
OF1: 10865.3
PTS1d: 32768 , 32768
EX: zgpg30
PW: 10.0 us
PD: 2.0 sec
NA: 588
LB: 0.2
Nuts - $glr_1_49_170629_2

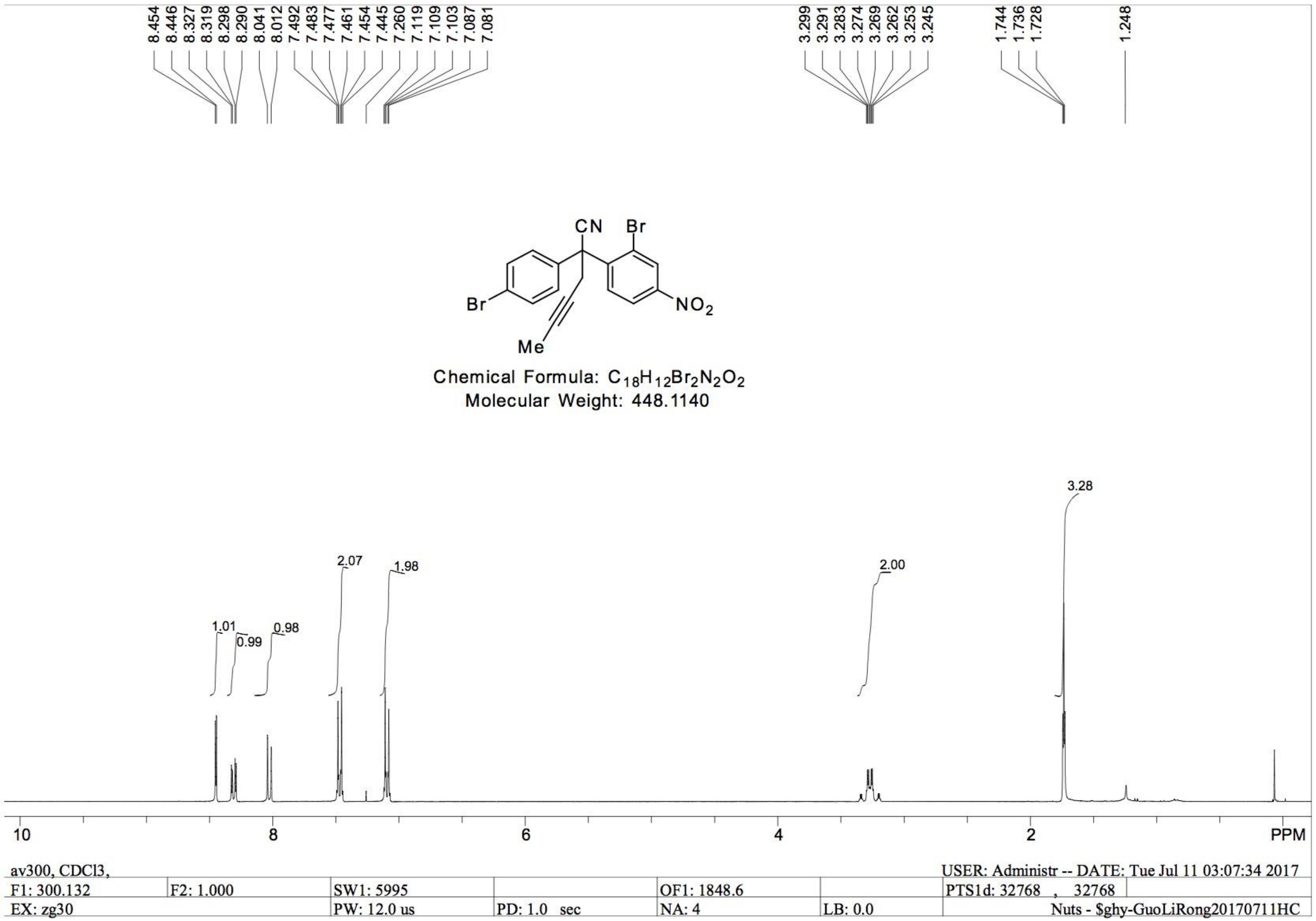
8.454
8.446
8.327
8.319
8.298
8.290
8.041
8.012
7.492
7.483
7.477
7.461
7.454
7.445
7.260
7.119
7.109
7.103
7.087
7.081
3.299
3.291
3.283
3.274
3.269
3.262
3.253
3.245
1.744
1.736
1.728
1.248
CN
Br
Br
NO2
Me
Chemical Formula: C18H12Br2N2O2
Molecular Weight: 448.1140
1.01
0.99
0.98
2.07
1.98
2.00
3.28
10
8
6
4
2
PPM
av300, CDCl3,
USER: Administr -- DATE: Tue Jul 11 03:07:34 2017
F1: 300.132
F2: 1.000
SW1: 5995
OF1: 1848.6
PTS1d: 32768 , 32768
EX: zg30
PW: 12.0 us
PD: 1.0 sec
NA: 4
LB: 0.0
Nuts - $ghy-GuoLiRong20170711HC

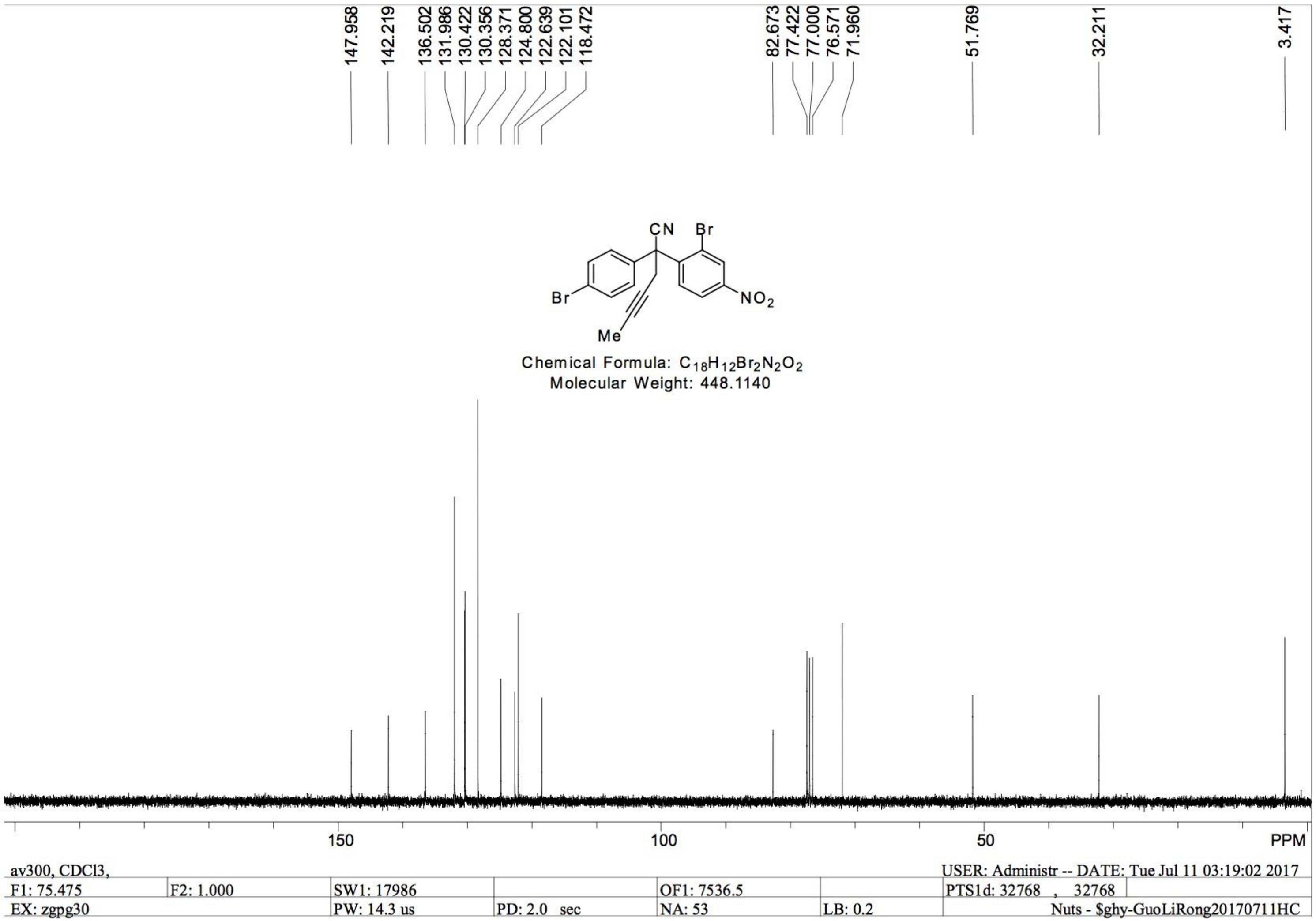
147.958
142.219
136.502
131.986
130.422
130.356
128.371
124.800
122.639
122.101
118.472
82.673
77.422
77.000
76.571
71.960
51.769
32.211
3.417
CN
Br
Br
NO2
Me
Chemical Formula: C18H12Br2N2O2
Molecular Weight: 448.1140
150
100
50
PPM
av300, CDCl3,
USER: Administr -- DATE: Tue Jul 11 03:19:02 2017
F1: 75.475
F2: 1.000
SW1: 17986
OF1: 7536.5
PTS1d: 32768 , 32768
EX: zgpg30
PW: 14.3 us
PD: 2.0 sec
NA: 53
LB: 0.2
Nuts - $ghy-GuoLiRong20170711HC

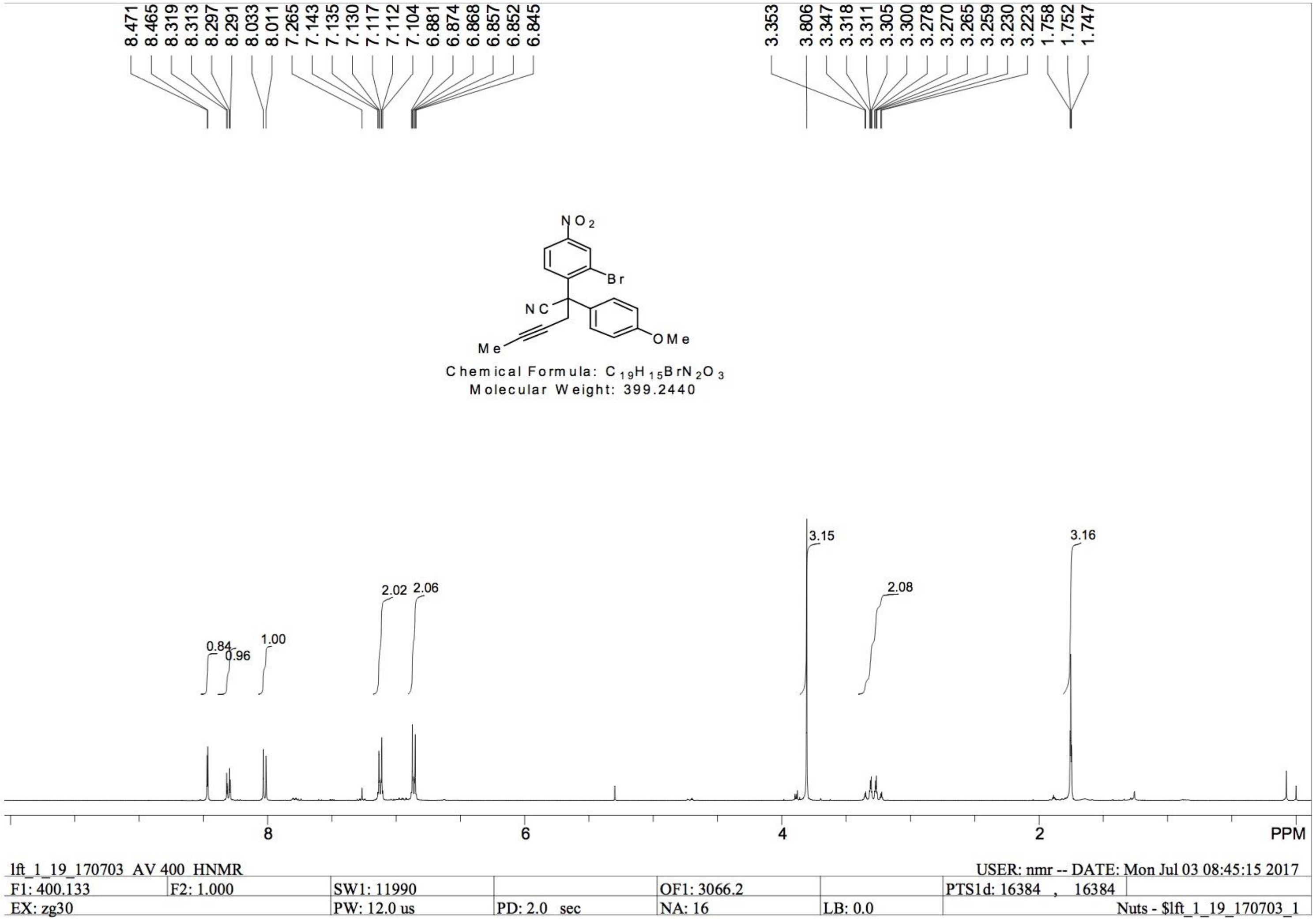

8.471 8.465 8.319 8.313 8.297 8.291 8.033 8.011 7.265 7.143 7.135 7.130 7.117 7.112 7.104 6.881 6.874 6.868 6.857 6.852 6.845
3.353 3.806 3.347 3.318 3.311 3.305 3.300 3.278 3.270 3.265 3.259 3.230 3.223 1.758 1.752 1.747
NO_2
Br
NC
OMe
Me
Chemical Formula: $C_{19}H_{15}BrN_2O_3$
Molecular Weight: 399.2440
0.84 0.96 1.00 2.02 2.06 3.15 2.08 3.16
8 6 4 2 PPM
lft_1_19_170703 AV 400 HNMR
USER: nmr -- DATE: Mon Jul 03 08:45:15 2017
F1: 400.133
F2: 1.000
SW1: 11990
OF1: 3066.2
PTS1d: 16384 , 16384
EX: zg30
PW: 12.0 us
PD: 2.0 sec
NA: 16
LB: 0.0
Nuts - $lft_1_19_170703_1

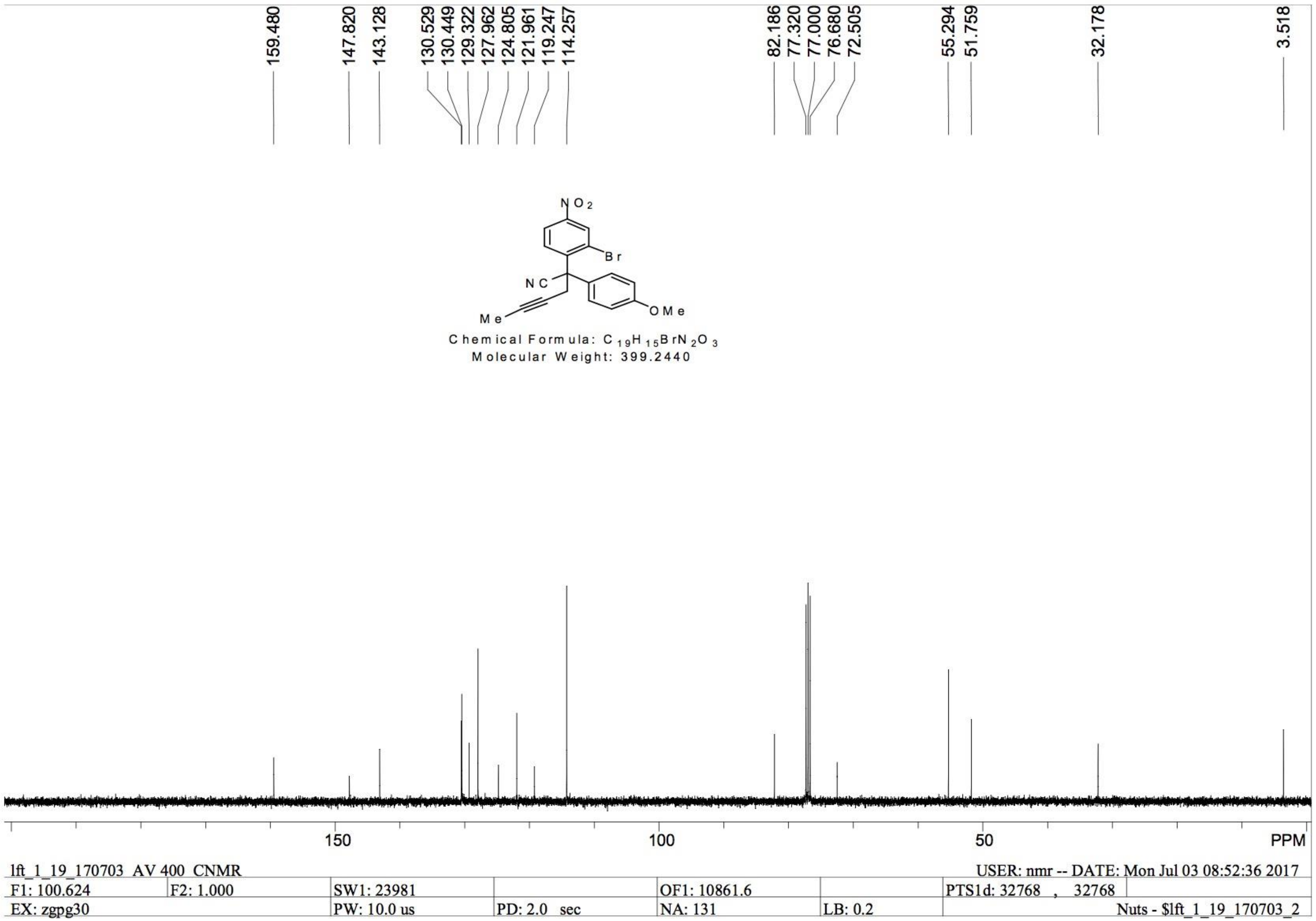
159.480
147.820
143.128
130.529
130.449
129.322
127.962
124.805
121.961
119.247
114.257
82.186
77.320
77.000
76.680
72.505
55.294
51.759
32.178
3.518
NO2
Br
NC
OMe
Me
Chemical Formula: C19H15BrN2O3
Molecular Weight: 399.2440
150
100
50
PPM
lft_1_19_170703 AV 400 CNMR
USER: nmr -- DATE: Mon Jul 03 08:52:36 2017
F1: 100.624
F2: 1.000
SW1: 23981
OF1: 10861.6
PTS1d: 32768 , 32768
EX: zgpg30
PW: 10.0 us
PD: 2.0 sec
NA: 131
LB: 0.2
Nuts - $lft_1_19_170703_2

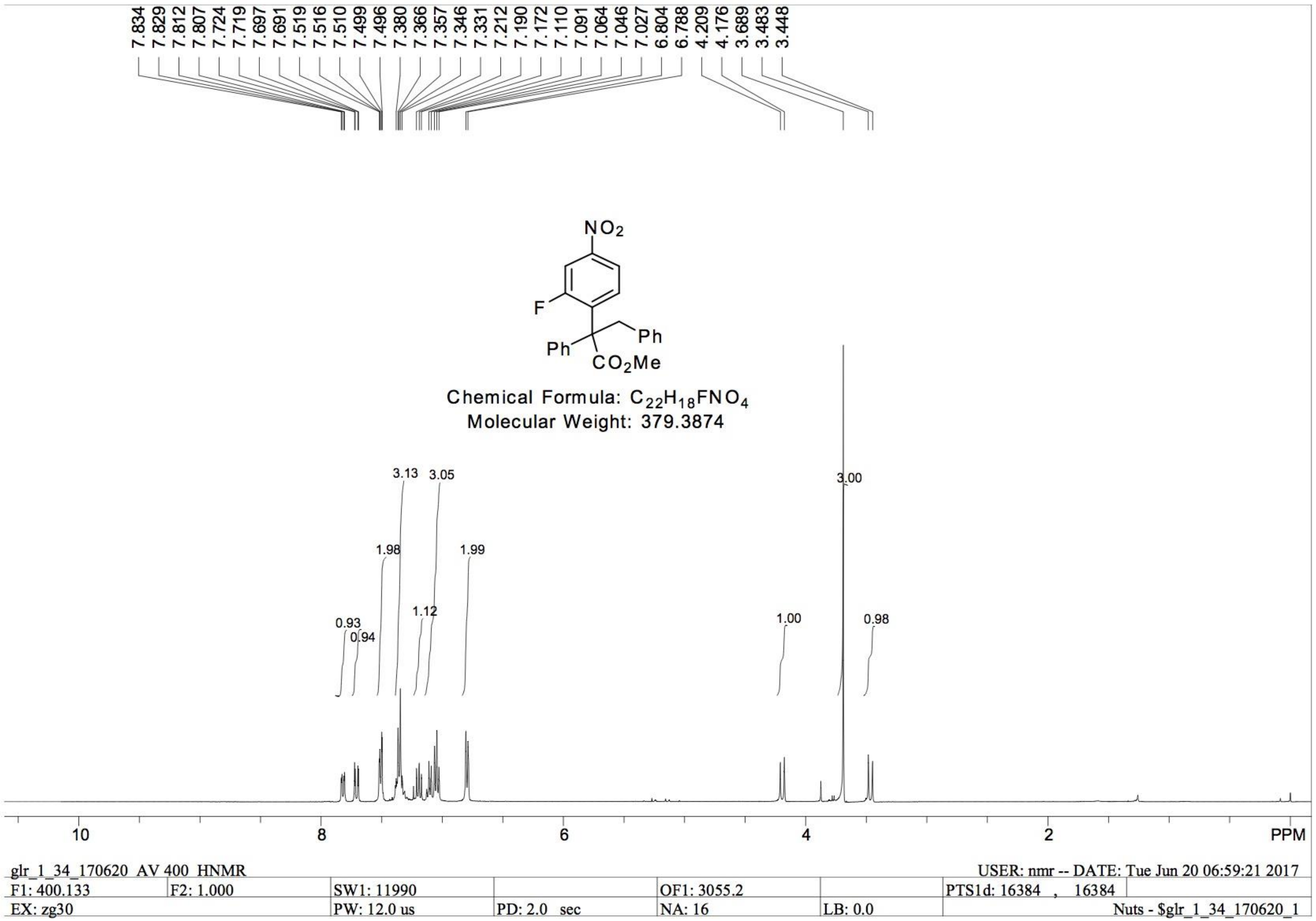

7.834
7.829
7.812
7.807
7.724
7.719
7.697
7.691
7.519
7.516
7.510
7.499
7.496
7.380
7.366
7.357
7.346
7.331
7.212
7.190
7.172
7.110
7.091
7.064
7.046
7.027
6.804
6.788
4.209
4.176
3.689
3.483
3.448
NO2
F
Ph
Ph
CO2Me
Chemical Formula: C22H18FNO4
Molecular Weight: 379.3874
0.93
0.94
1.98
3.13
1.12
3.05
1.99
1.00
3.00
0.98
10
8
6
4
2
PPM
glr_1_34_170620 AV 400 HNMR
USER: nmr -- DATE: Tue Jun 20 06:59:21 2017
F1: 400.133
F2: 1.000
SW1: 11990
OF1: 3055.2
PTS1d: 16384 , 16384
EX: zg30
PW: 12.0 us
PD: 2.0 sec
NA: 16
LB: 0.0
Nuts - $glr_1_34_170620_1

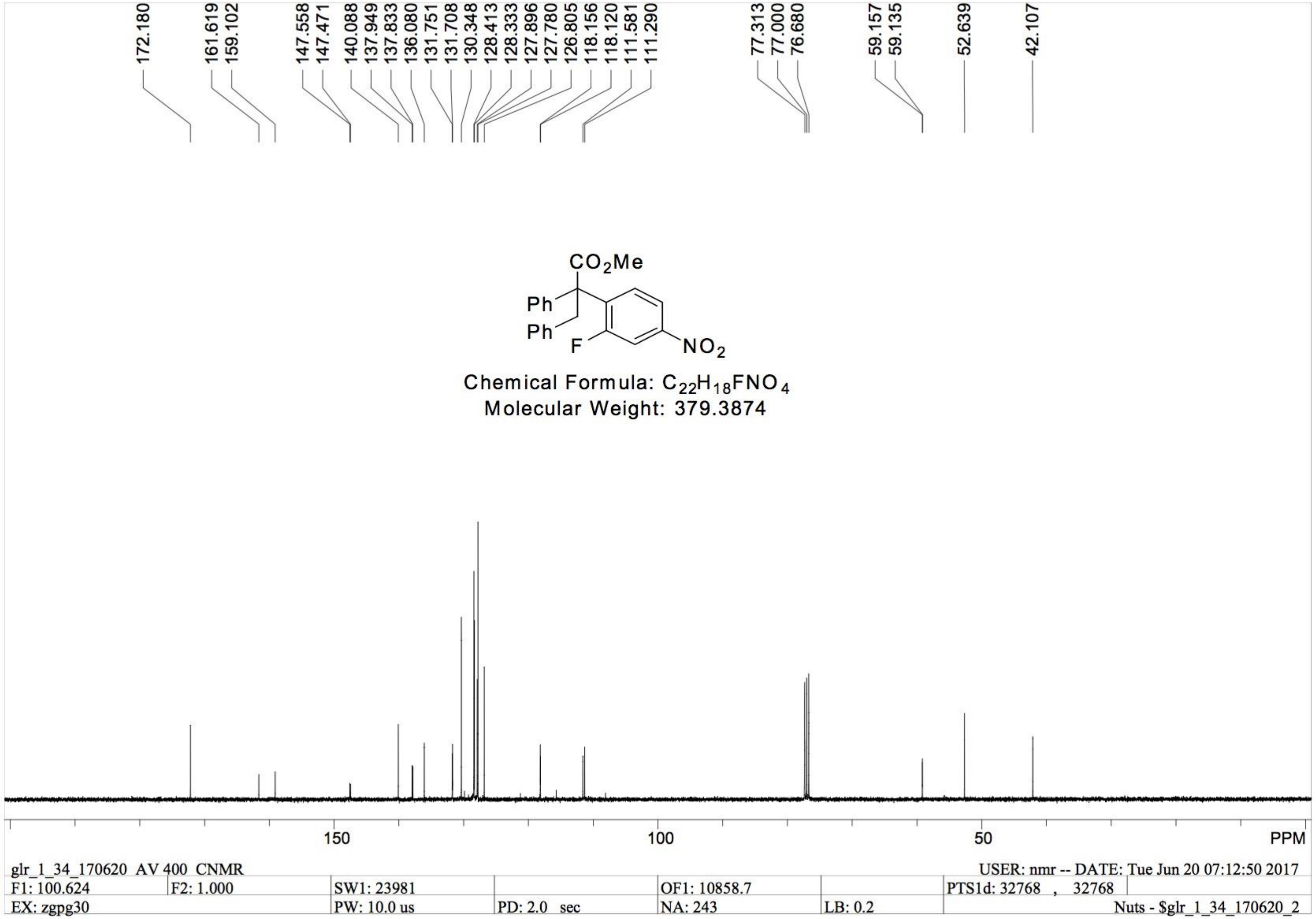
172.180
161.619
159.102
147.558
147.471
140.088
137.949
137.833
136.080
131.751
131.708
130.348
128.413
128.333
127.896
127.780
126.805
118.156
118.120
111.581
111.290
77.313
77.000
76.680
59.157
59.135
52.639
42.107
CO2Me
Ph
Ph
F
NO2
Chemical Formula: C22H18FNO4
Molecular Weight: 379.3874
150
100
50
PPM
glr_1_34_170620 AV 400 CNMR
USER: nmr -- DATE: Tue Jun 20 07:12:50 2017
F1: 100.624
F2: 1.000
SW1: 23981
OF1: 10858.7
PTS1d: 32768 , 32768
EX: zgpg30
PW: 10.0 us
PD: 2.0 sec
NA: 243
LB: 0.2
Nuts - $glr_1_34_170620_2

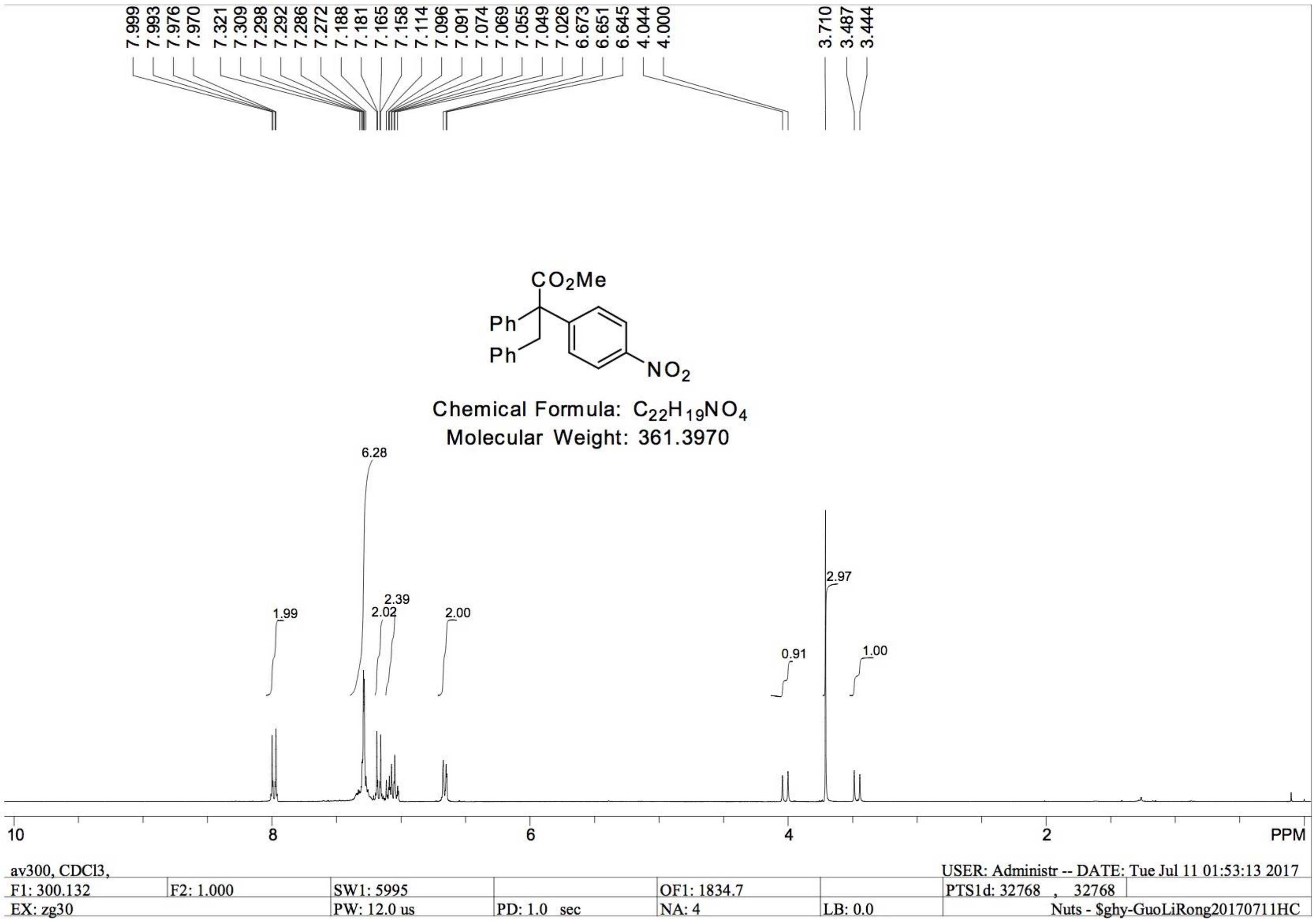
7.999 7.993 7.976 7.970 7.321 7.309 7.298 7.292 7.286 7.272 7.188 7.181 7.165 7.158 7.114 7.096 7.091 7.074 7.069 7.055 7.049 7.026 6.673 6.651 6.645 4.044 4.000 3.710 3.487 3.444
CO2Me
Ph
Ph
NO2
Chemical Formula: C22H19NO4
Molecular Weight: 361.3970
6.28
1.99
2.02
2.39
2.00
0.91
2.97
1.00
10
8
6
4
2
PPM
av300, CDCl3,
USER: Administr -- DATE: Tue Jul 11 01:53:13 2017
F1: 300.132
F2: 1.000
SW1: 5995
OF1: 1834.7
PTS1d: 32768 , 32768
EX: zg30
PW: 12.0 us
PD: 1.0 sec
NA: 4
LB: 0.0
Nuts - $ghy-GuoLiRong20170711HC

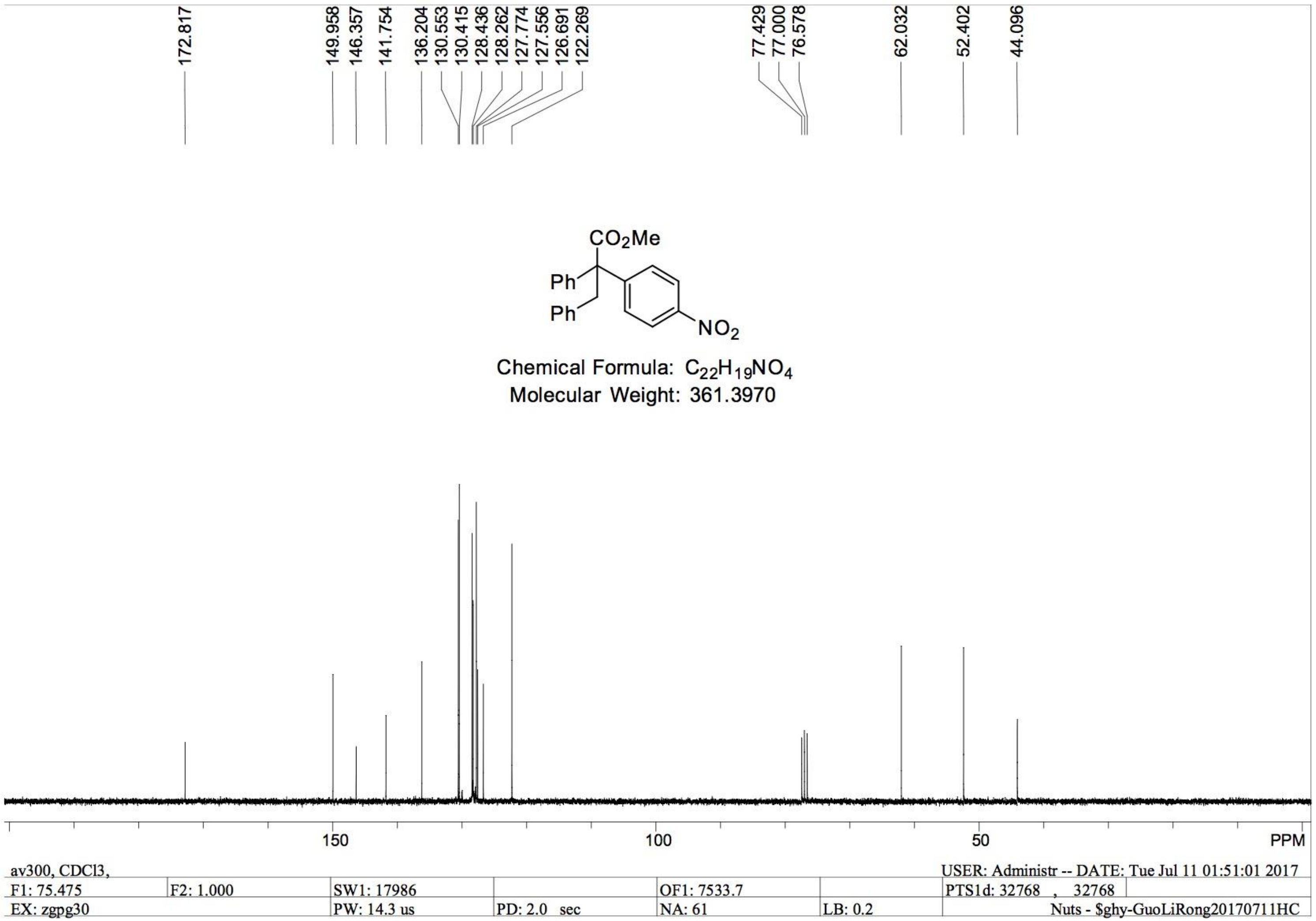

172.817
149.958
146.357
141.754
136.204
130.553
130.415
128.436
128.262
127.774
127.556
126.691
122.269
77.429
77.000
76.578
62.032
52.402
44.096
CO2Me
Ph
Ph
NO2
Chemical Formula: C22H19NO4
Molecular Weight: 361.3970
150
100
50
PPM
av300, CDCl3,
USER: Administr -- DATE: Tue Jul 11 01:51:01 2017
F1: 75.475
F2: 1.000
SW1: 17986
OF1: 7533.7
PTS1d: 32768 , 32768
EX: zgpg30
PW: 14.3 us
PD: 2.0 sec
NA: 61
LB: 0.2
Nuts - $ghy-GuoLiRong20170711HC

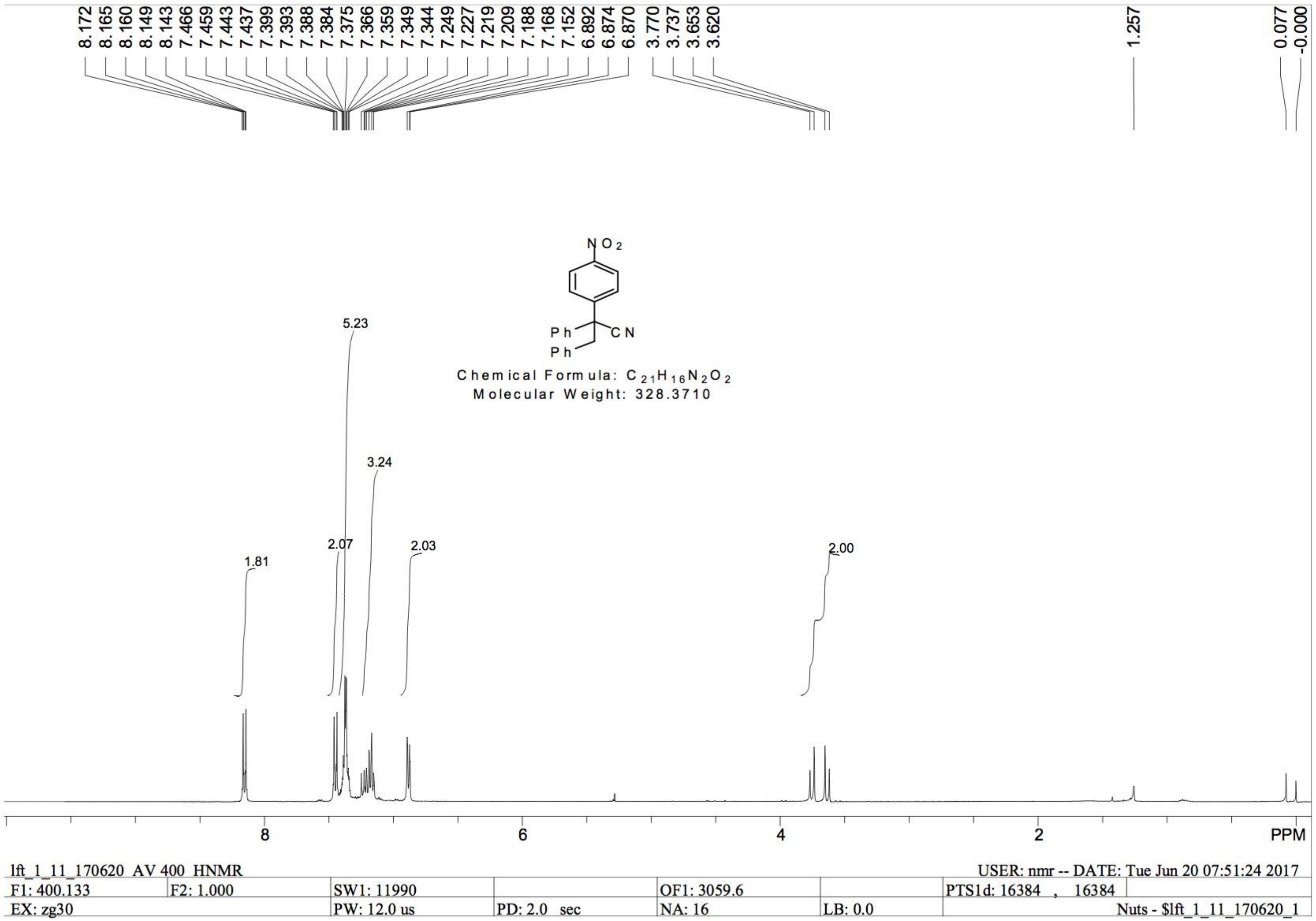
8.172
8.165
8.160
8.149
8.143
7.466
7.459
7.443
7.437
7.399
7.393
7.388
7.384
7.375
7.366
7.359
7.349
7.344
7.249
7.227
7.219
7.209
7.188
7.168
7.152
6.892
6.874
6.870
3.770
3.737
3.653
3.620
1.257
0.077
-0.000
NO2
Ph
CN
Ph
Chemical Formula: C21H16N2O2
Molecular Weight: 328.3710
5.23
3.24
2.07
2.03
1.81
2.00
8
6
4
2
PPM
lft_1_11_170620 AV 400 HNMR
USER: nmr -- DATE: Tue Jun 20 07:51:24 2017
F1: 400.133
F2: 1.000
SW1: 11990
OF1: 3059.6
PTS1d: 16384 , 16384
EX: zg30
PW: 12.0 us
PD: 2.0 sec
NA: 16
LB: 0.0
Nuts - $lft_1_11_170620_1

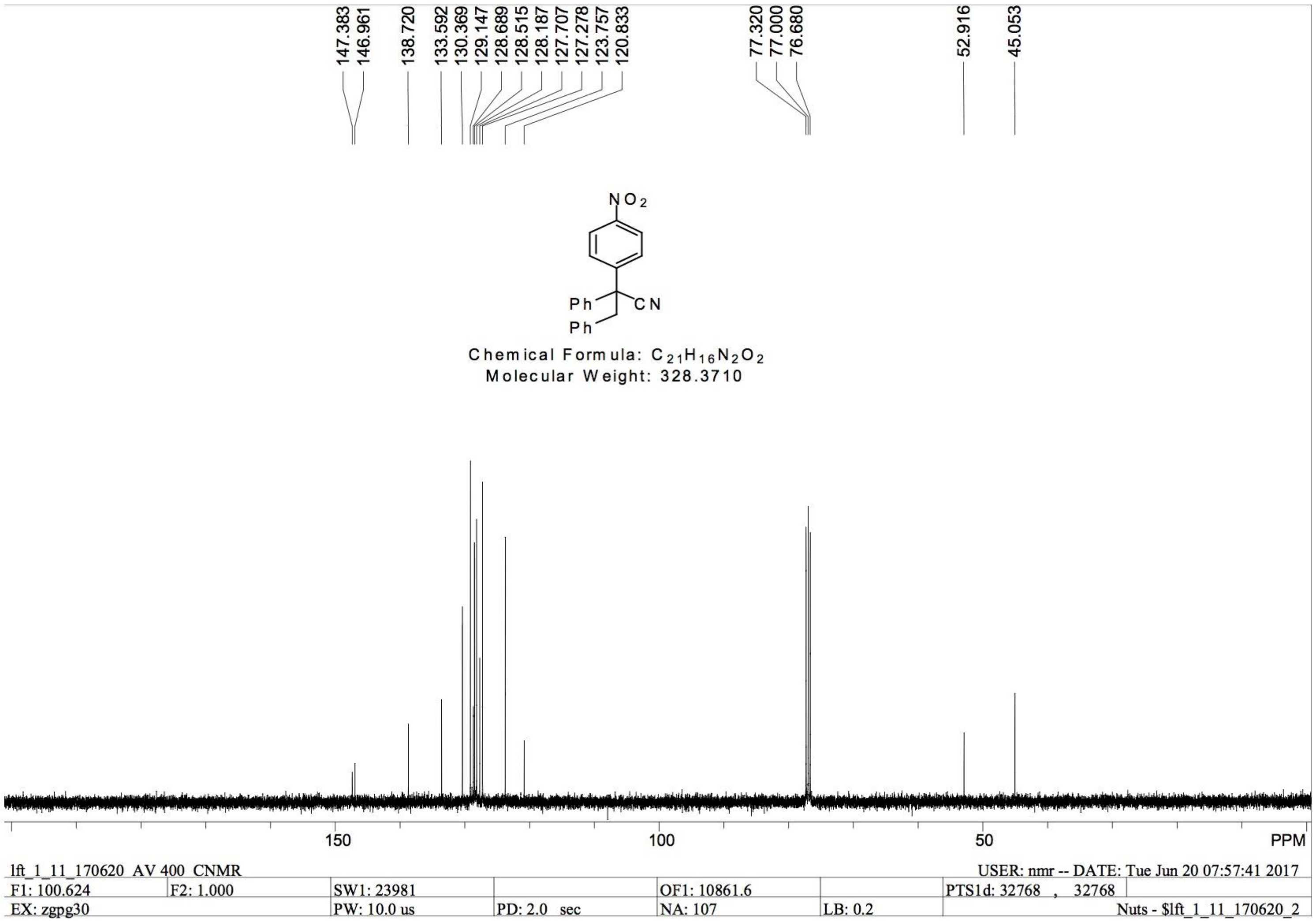
147.383
146.961
138.720
133.592
130.369
129.147
128.689
128.515
128.187
127.707
127.278
123.757
120.833
77.320
77.000
76.680
52.916
45.053
NO2
Ph
CN
Ph
Chemical Formula: C21H16N2O2
Molecular Weight: 328.3710
150
100
50
PPM
lft_1_11_170620 AV 400 CNMR
USER: nmr -- DATE: Tue Jun 20 07:57:41 2017
F1: 100.624
F2: 1.000
SW1: 23981
OF1: 10861.6
PTS1d: 32768 , 32768
EX: zgpg30
PW: 10.0 us
PD: 2.0 sec
NA: 107
LB: 0.2
Nuts - $lft_1_11_170620_2

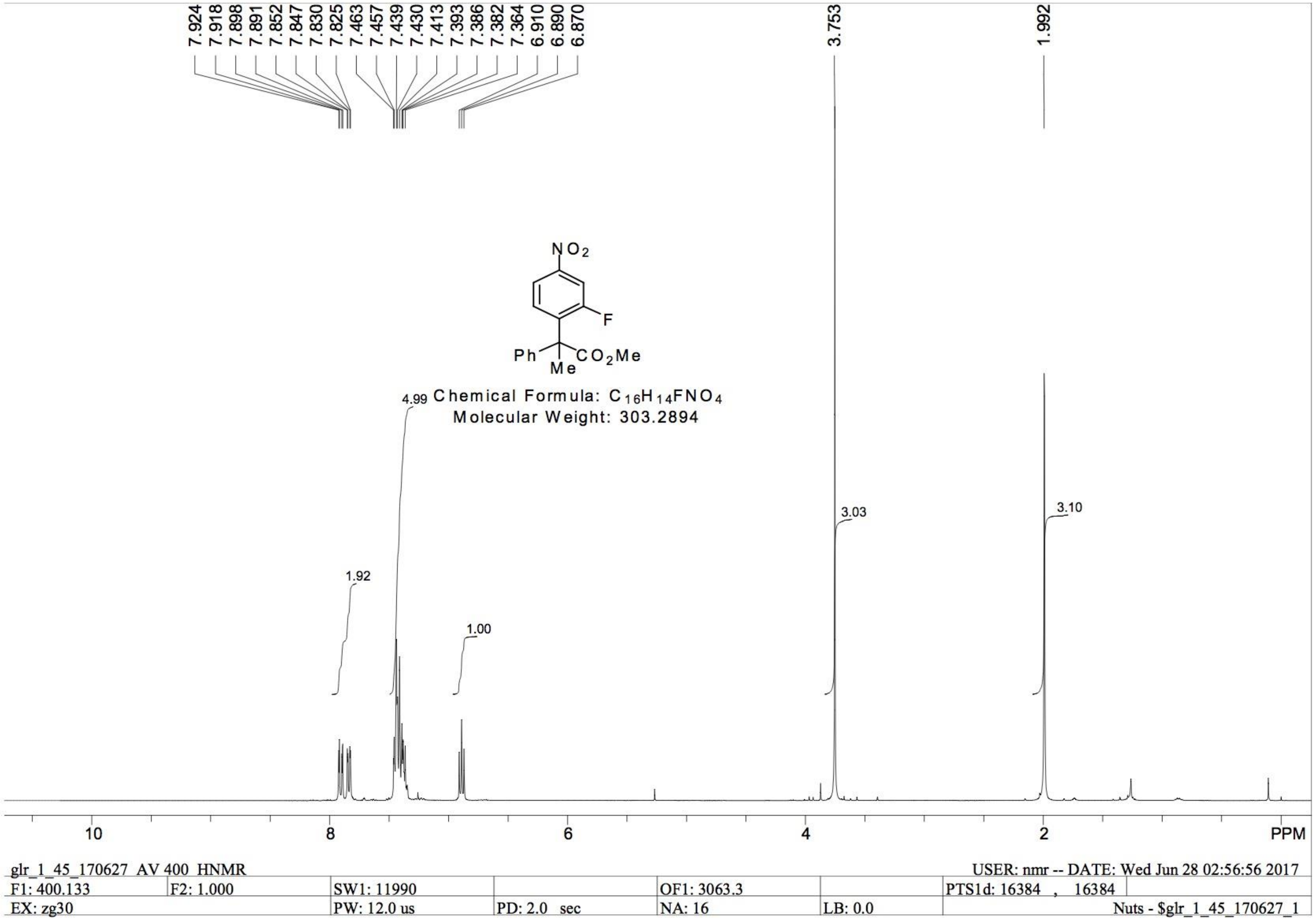
7.924
7.918
7.898
7.891
7.852
7.847
7.830
7.825
7.463
7.457
7.439
7.430
7.413
7.393
7.386
7.382
7.364
6.910
6.890
6.870
3.753
1.992
NO2
F
Ph
Me
CO2Me
Chemical Formula: C16H14FNO4
Molecular Weight: 303.2894
1.92
4.99
1.00
3.03
3.10
10
8
6
4
2
PPM
glr_1_45_170627 AV 400 HNMR
USER: nmr -- DATE: Wed Jun 28 02:56:56 2017
F1: 400.133
F2: 1.000
SW1: 11990
OF1: 3063.3
PTS1d: 16384 , 16384
EX: zg30
PW: 12.0 us
PD: 2.0 sec
NA: 16
LB: 0.0
Nuts - $glr_1_45_170627_1

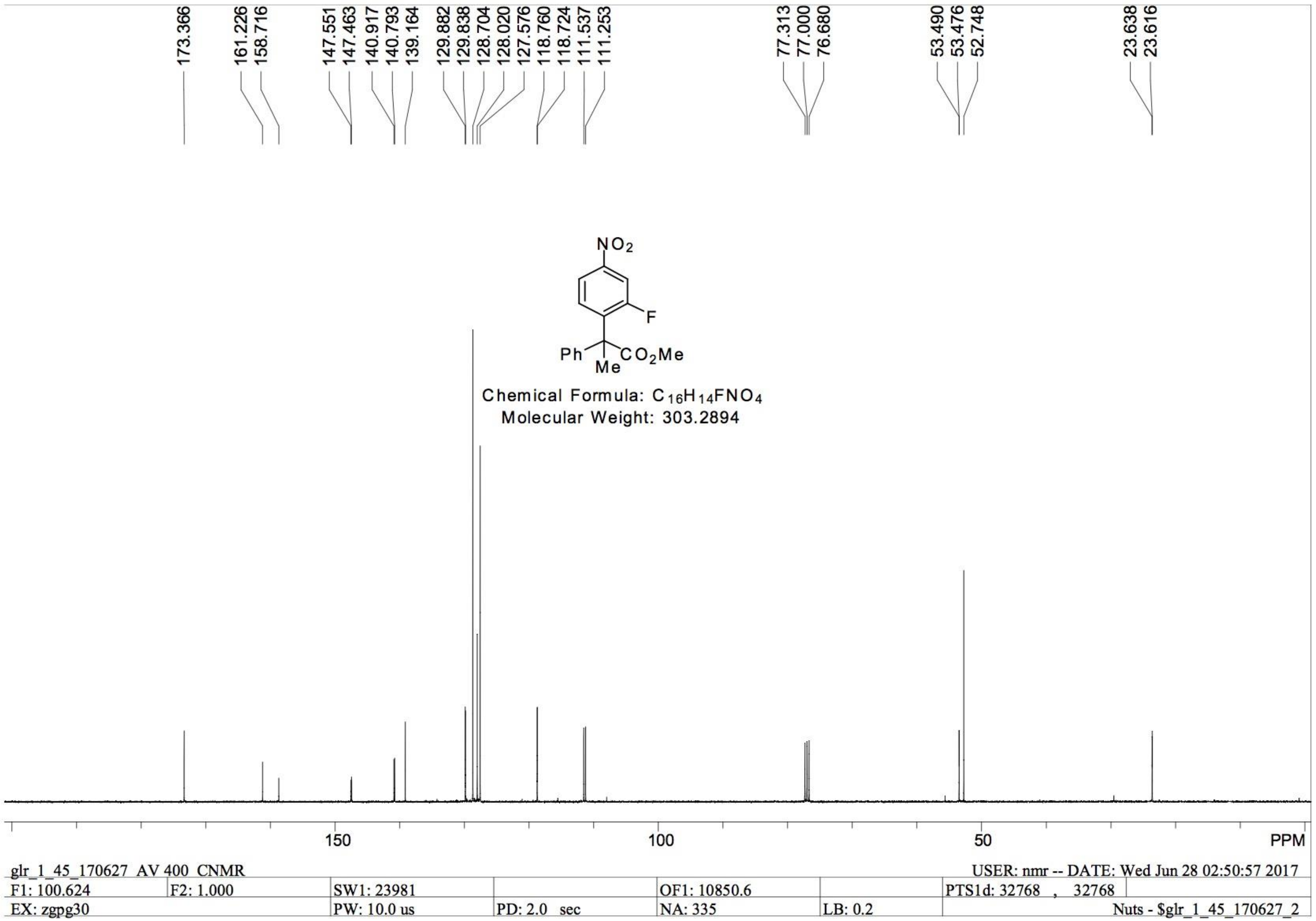
173.366
161.226
158.716
147.551
147.463
140.917
140.793
139.164
129.882
129.838
128.704
128.020
127.576
118.760
118.724
111.537
111.253
77.313
77.000
76.680
53.490
53.476
52.748
23.638
23.616
NO2
F
Ph
CO2Me
Me
Chemical Formula: C16H14FNO4
Molecular Weight: 303.2894
150
100
50
PPM
glr_1_45_170627 AV 400 CNMR
USER: nmr -- DATE: Wed Jun 28 02:50:57 2017
F1: 100.624
F2: 1.000
SW1: 23981
OF1: 10850.6
PTS1d: 32768 , 32768
EX: zgpg30
PW: 10.0 us
PD: 2.0 sec
NA: 335
LB: 0.2
Nuts - $glr_1_45_170627_2

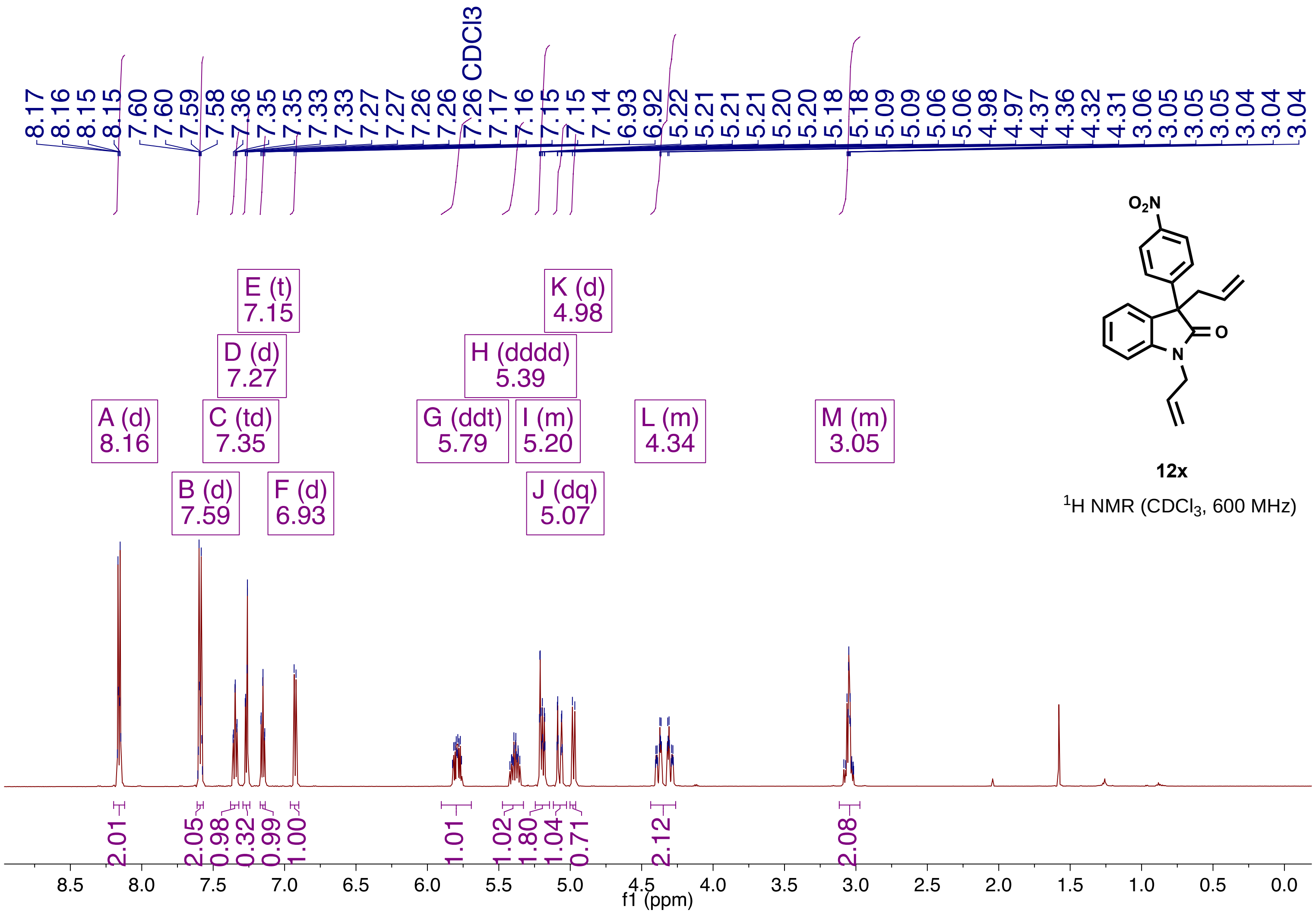

12x

^{1}H NMR ($CDCl_3$, 600 MHz)

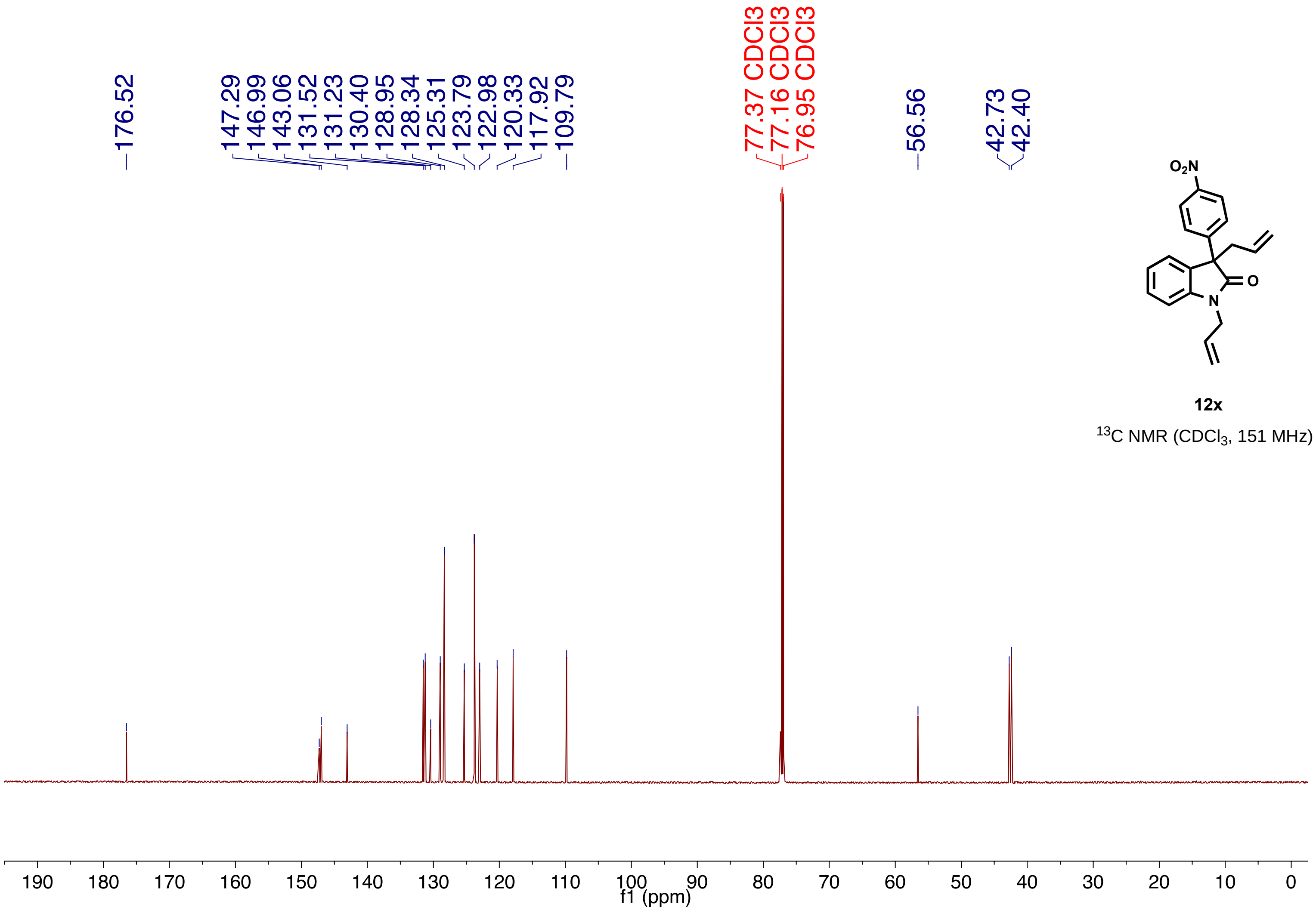

12x

^{13}C NMR ($CDCl_3$, 151 MHz)

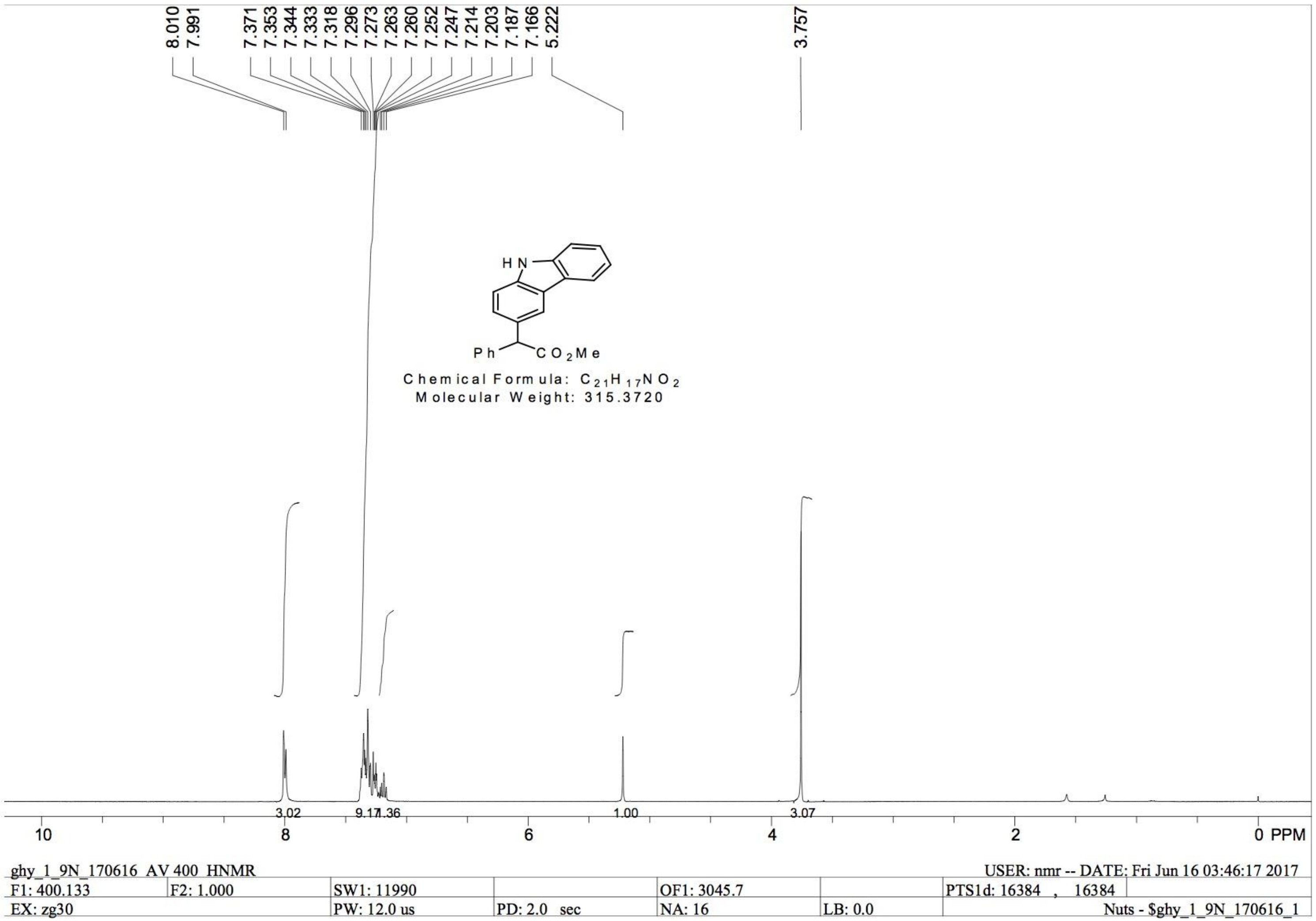
8.010
7.991
7.371
7.353
7.344
7.333
7.318
7.296
7.273
7.263
7.260
7.252
7.247
7.214
7.203
7.187
7.166
5.222
3.757
HN
Ph
CO2Me
Chemical Formula: C21H17NO2
Molecular Weight: 315.3720
3.02
9.17
1.36
1.00
3.07
10
8
6
4
2
0 PPM
ghy_1_9N_170616 AV 400 HNMR
USER: nmr -- DATE: Fri Jun 16 03:46:17 2017
F1: 400.133
F2: 1.000
SW1: 11990
OF1: 3045.7
PTS1d: 16384 , 16384
EX: zg30
PW: 12.0 us
PD: 2.0 sec
NA: 16
LB: 0.0
Nuts - $ghy_1_9N_170616_1

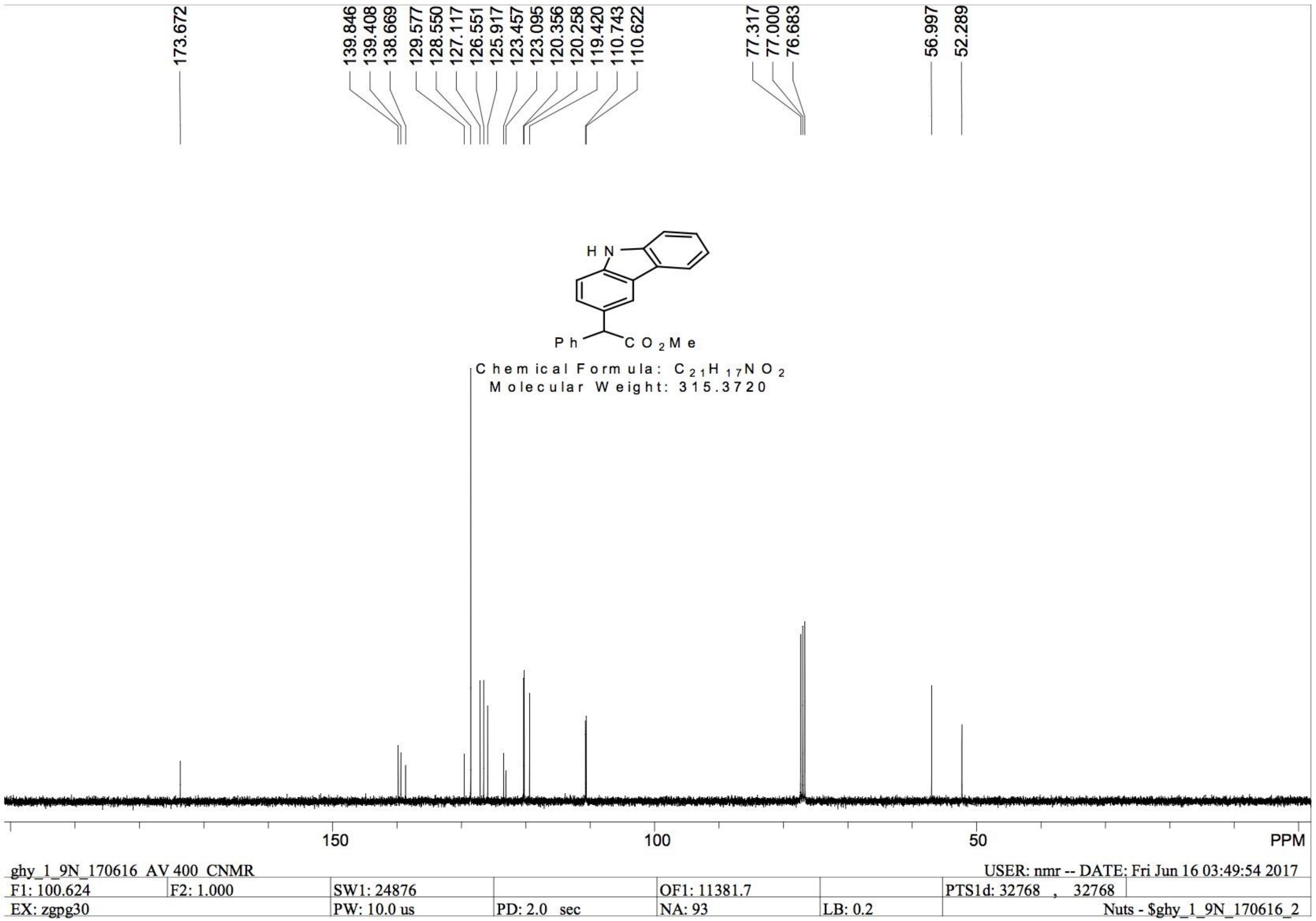
173.672
139.846
139.408
138.669
129.577
128.550
127.117
126.551
125.917
123.457
123.095
120.356
120.258
119.420
110.743
110.622
77.317
77.000
76.683
56.997
52.289
HN
Ph
CO2Me
Chemical Formula: C21H17NO2
Molecular Weight: 315.3720
150
100
50
PPM
ghy_1_9N_170616 AV 400 CNMR
USER: nmr -- DATE: Fri Jun 16 03:49:54 2017
F1: 100.624
F2: 1.000
SW1: 24876
OF1: 11381.7
PTS1d: 32768 , 32768
EX: zgpg30
PW: 10.0 us
PD: 2.0 sec
NA: 93
LB: 0.2
Nuts - $ghy_1_9N_170616_2

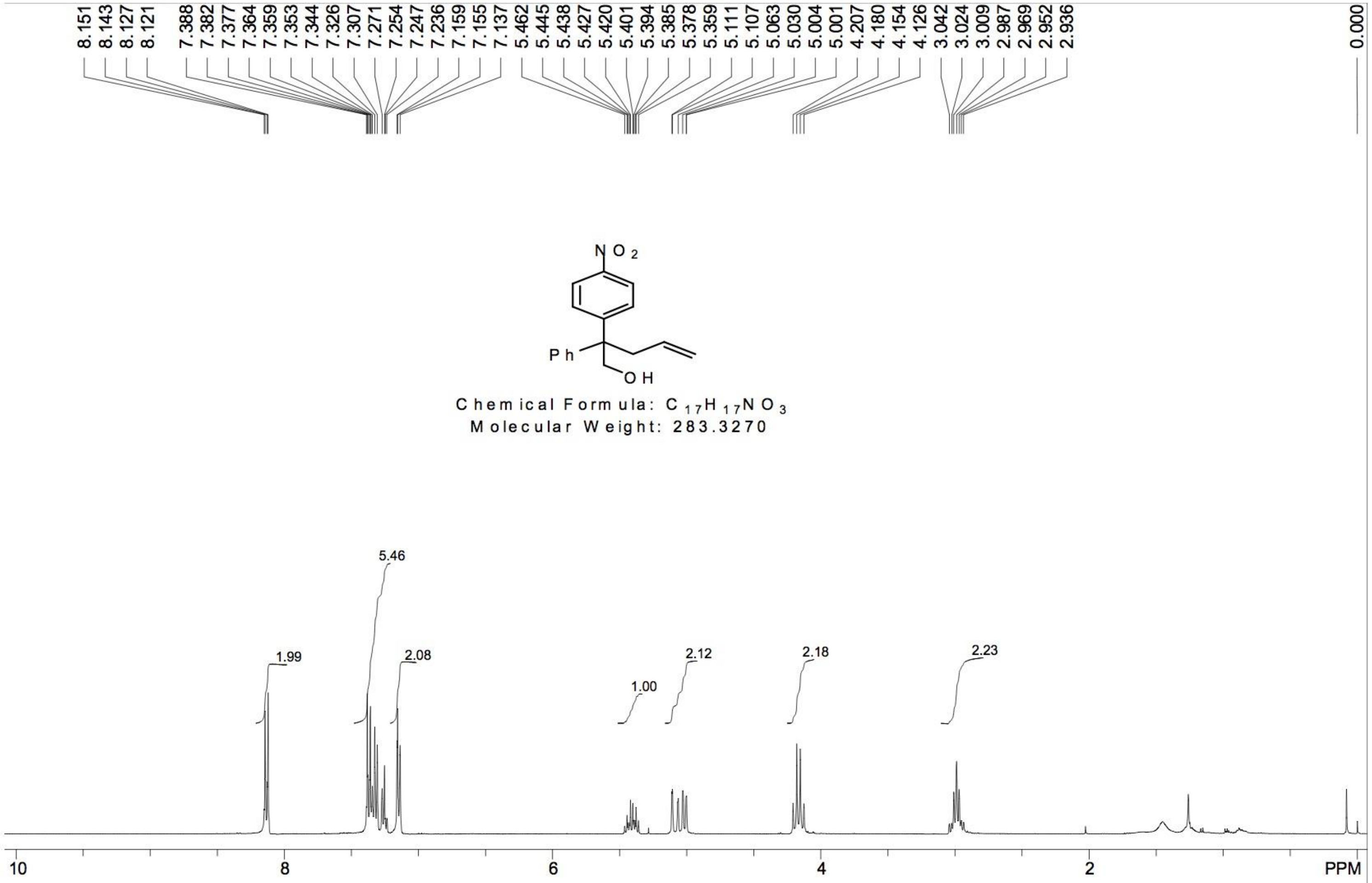

glr_1_59_170705 AV 400 HNMR						USER: nmr -- DATE: Wed Jul 05 03:17:05 2017
F1: 400.133	F2: 1.000	SW1: 11990		OF1: 3061.8		PTS1d: 16384 , 16384
EX: zg30		PW: 12.0 us	PD: 2.0 sec	NA: 8	LB: 0.0	Nuts - $glr_1_59_170705_1

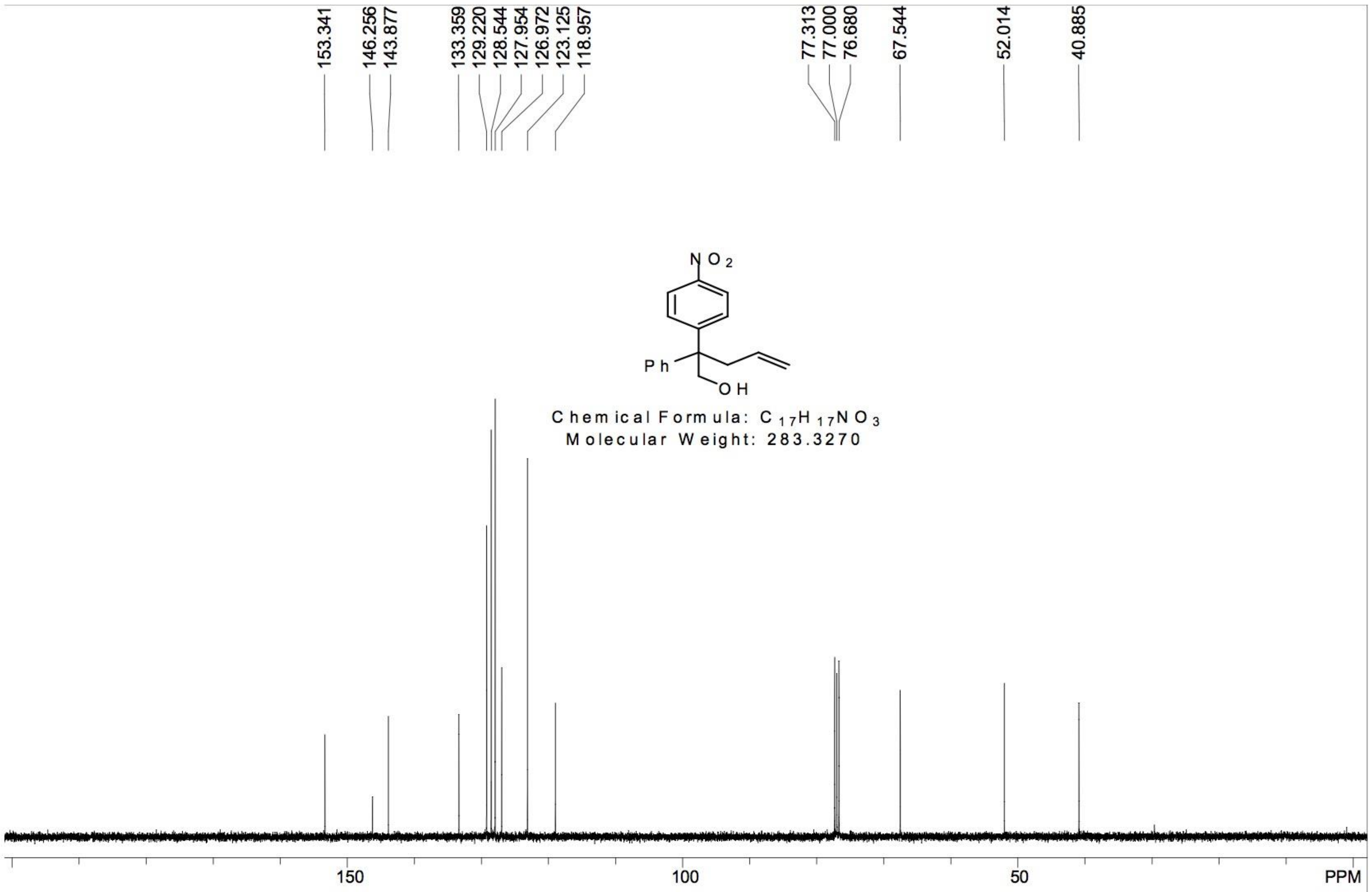

glr_1_59_170705 AV 400 CNMR							USER: nmr -- DATE: Wed Jul 05 03:16:05 2017
F1: 100.624	F2: 1.000	SW1: 23981		OF1: 10858.7		PTS1d: 32768 , 32768	
EX: zgpg30		PW: 10.0 us	PD: 2.0 sec	NA: 94	LB: 0.2		Nuts - $glr_1_59_170705_2

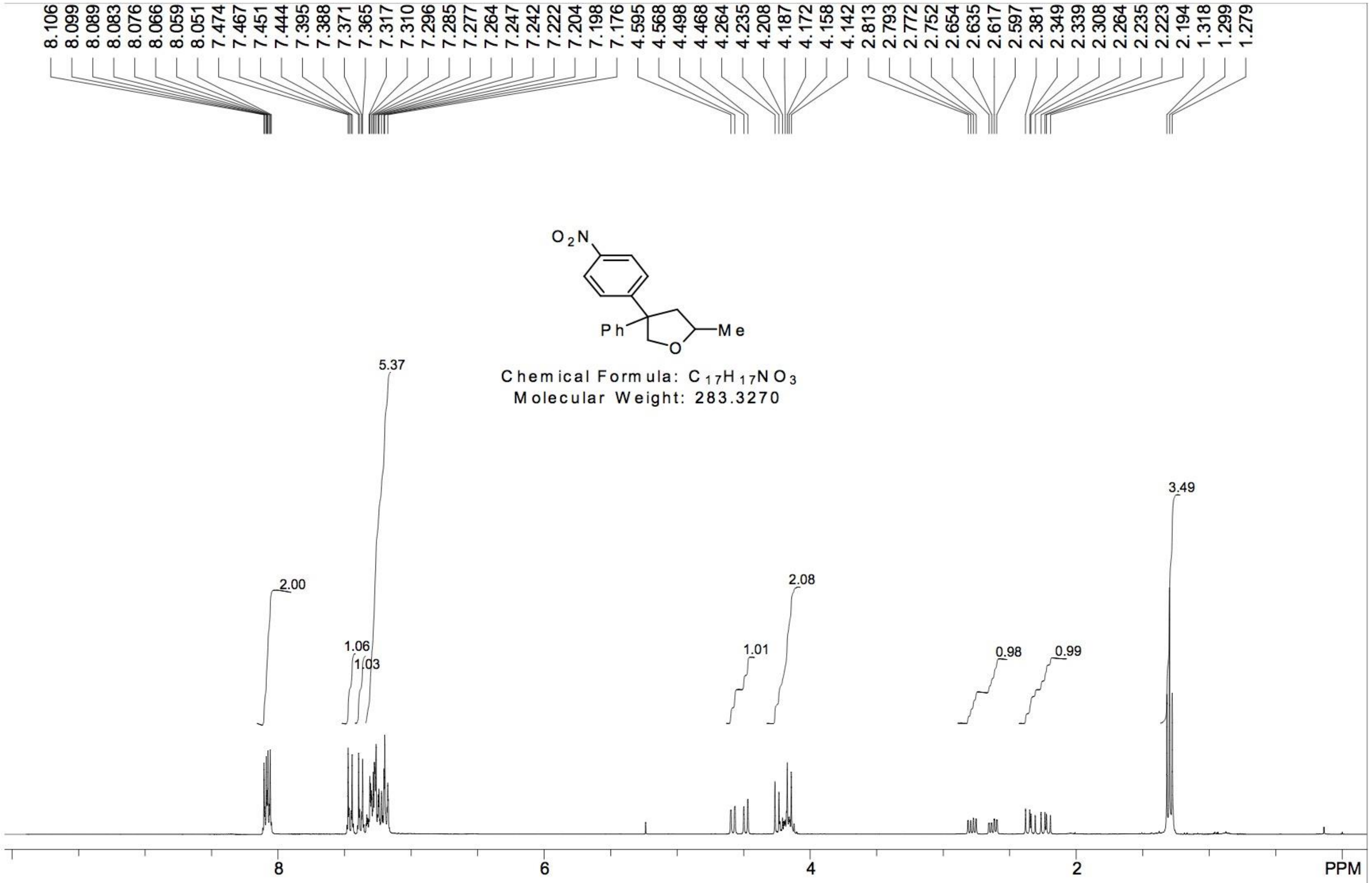

AV300 1H 20170720 LFT-1-31, ,						USER: Administr -- DATE: Thu Jul 20 02:51:15 2017
F1: 300.132	F2: 1.000	SW1: 6173		OF1: 1847.8		PTS1d: 32768 , 32768
EX: zg30		PW: 12.0 us	PD: 1.0 sec	NA: 6	LB: 0.0	Nuts - $ghy-LiuFengTing20170720HC-LFT-1-31_1

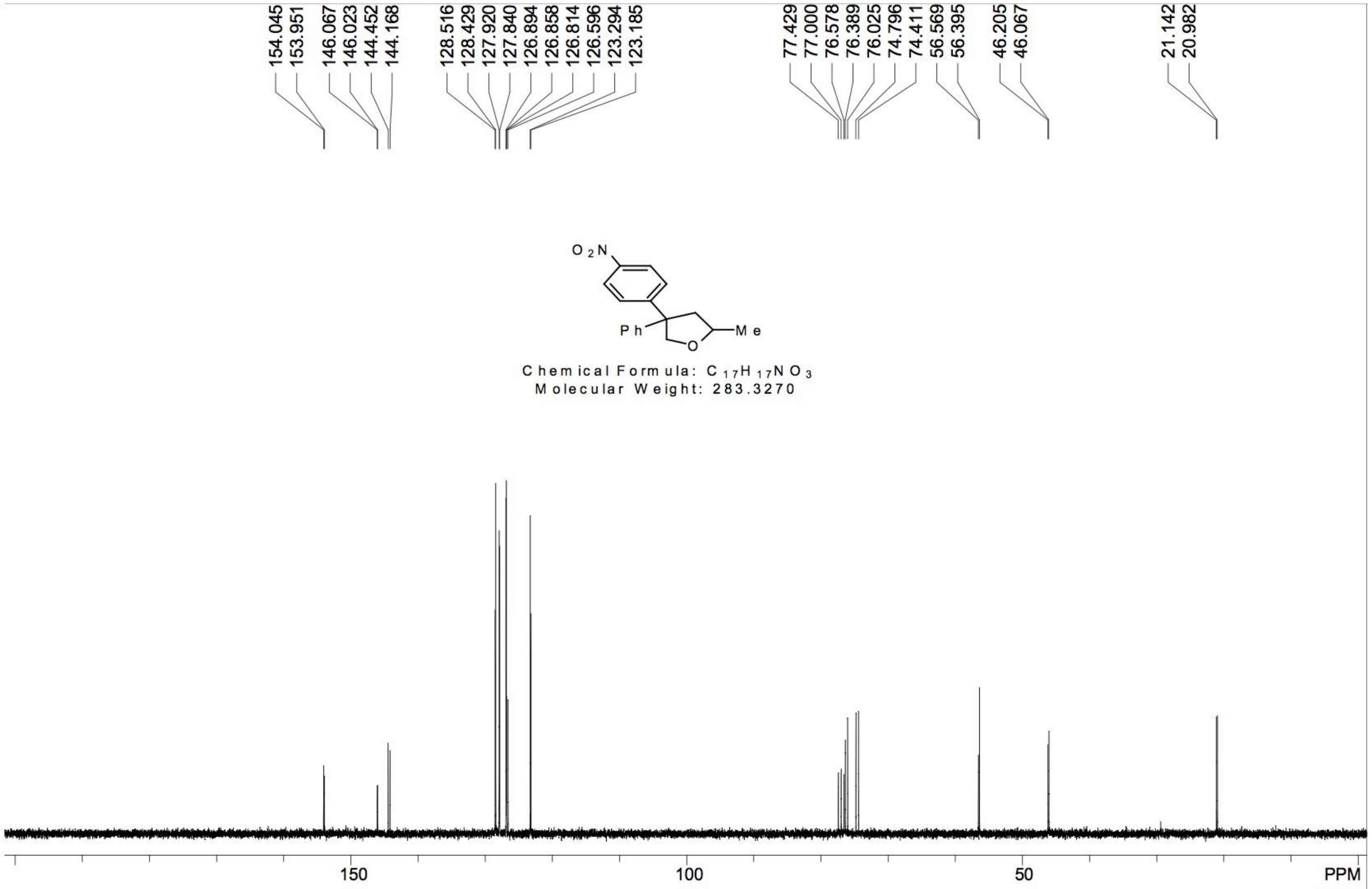

AV300 13C 20170720 LFT-1-31, ,						USER: Administr -- DATE: Thu Jul 20 02:53:15 2017
F1: 75.475	F2: 1.000	SW1: 17986		OF1: 7525.5		PTS1d: 32768 , 32768
EX: zgpg30		PW: 14.3 us	PD: 2.0 sec	NA: 18	LB: 0.2	Nuts - $ghy-LiuFengTing20170720HC-LFT-1-31_2

\

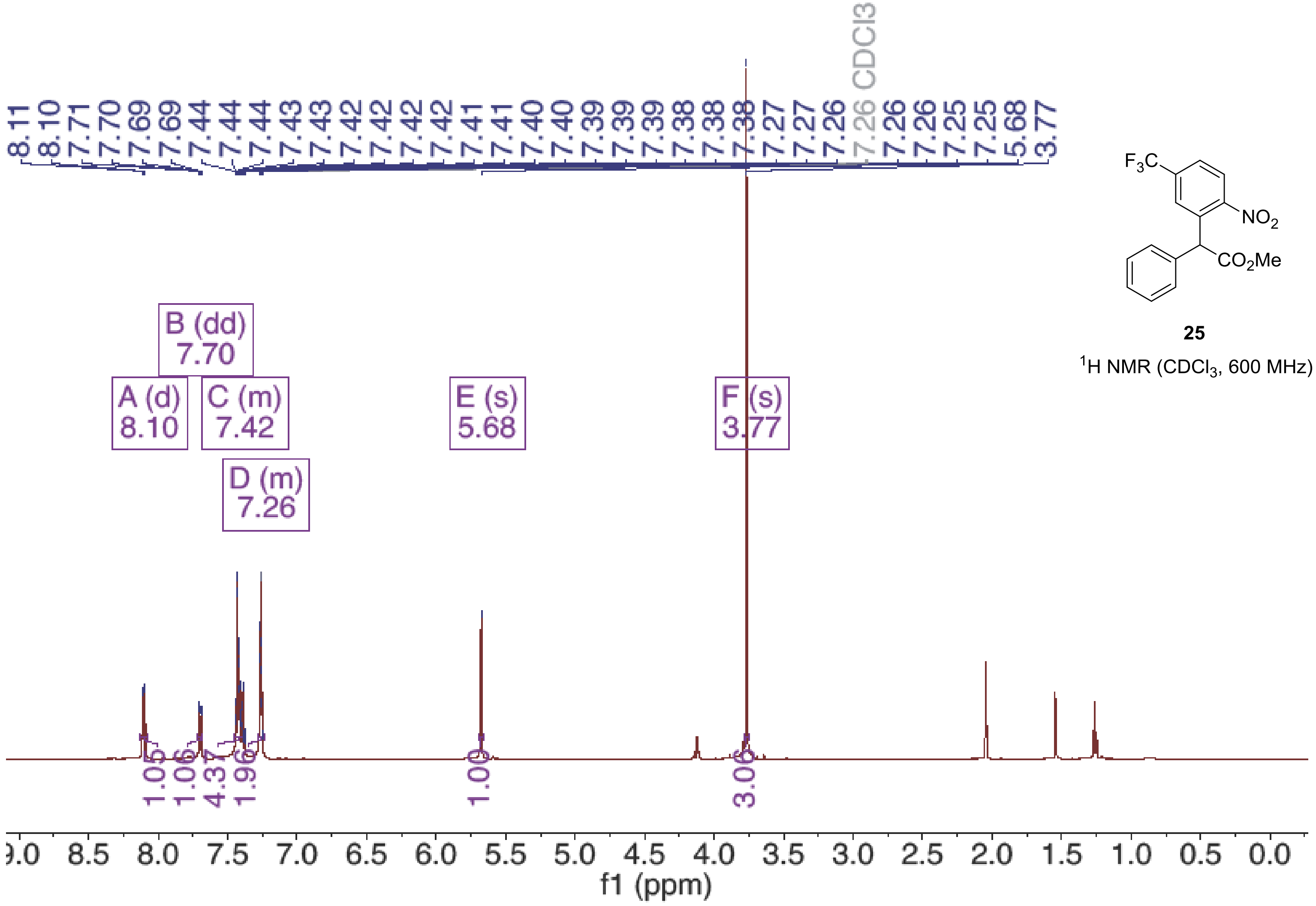

25

^{1}H NMR ($CDCl_3$, 600 MHz)

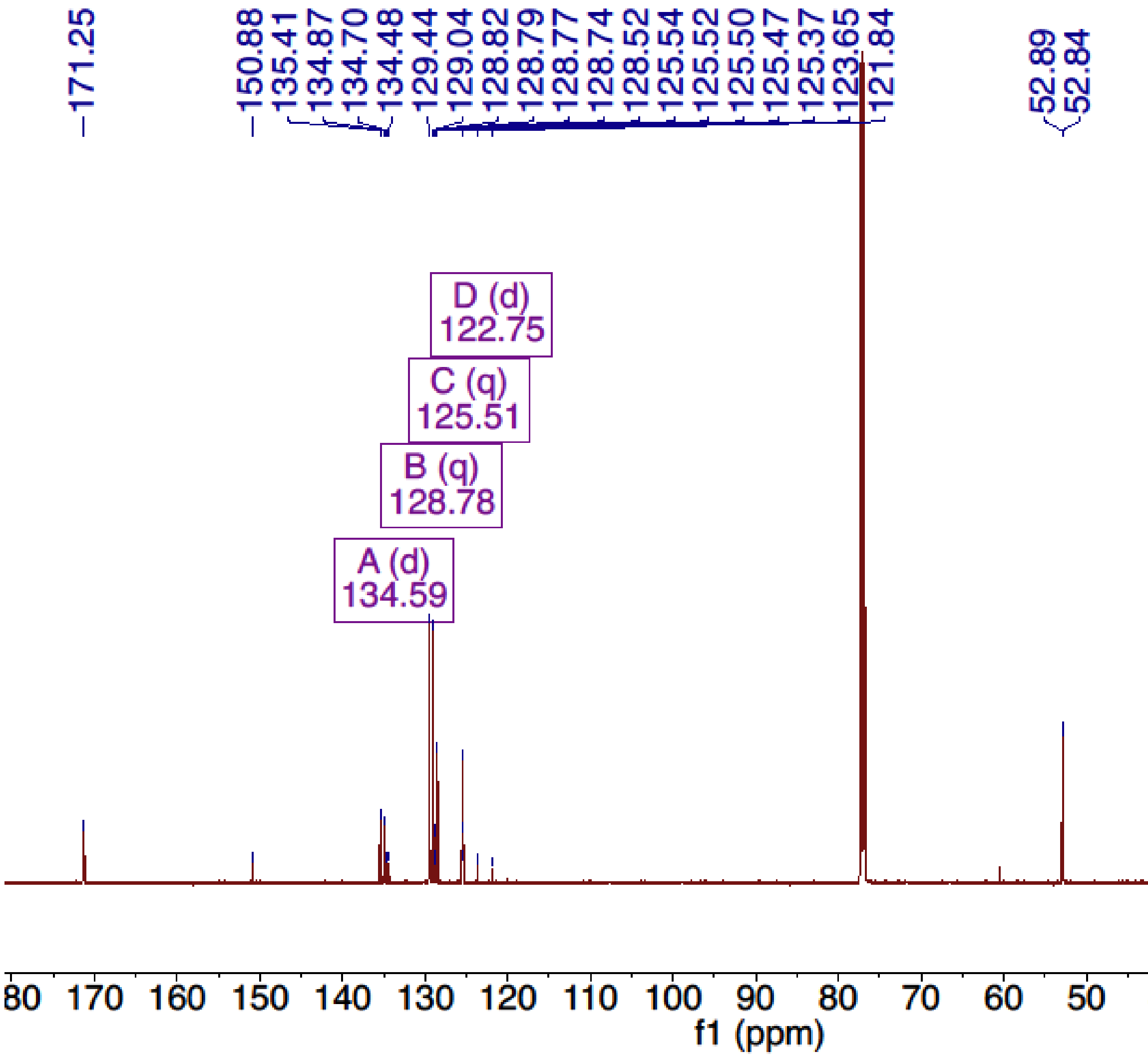

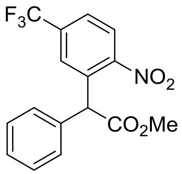

25

^{13}C NMR ($CDCl_3$, 151 MHz)

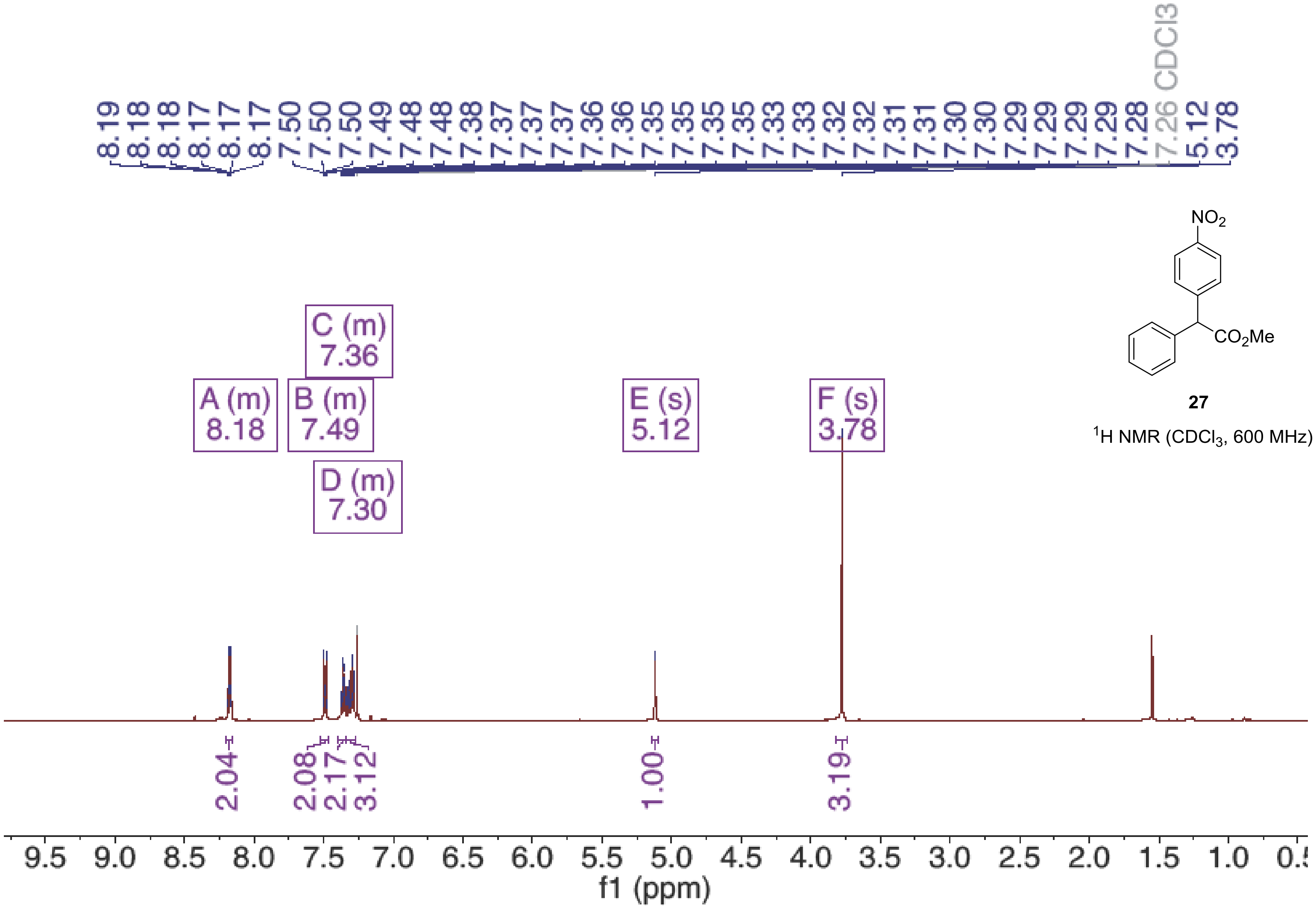

27

^{1}H NMR ($CDCl_3$, 600 MHz)

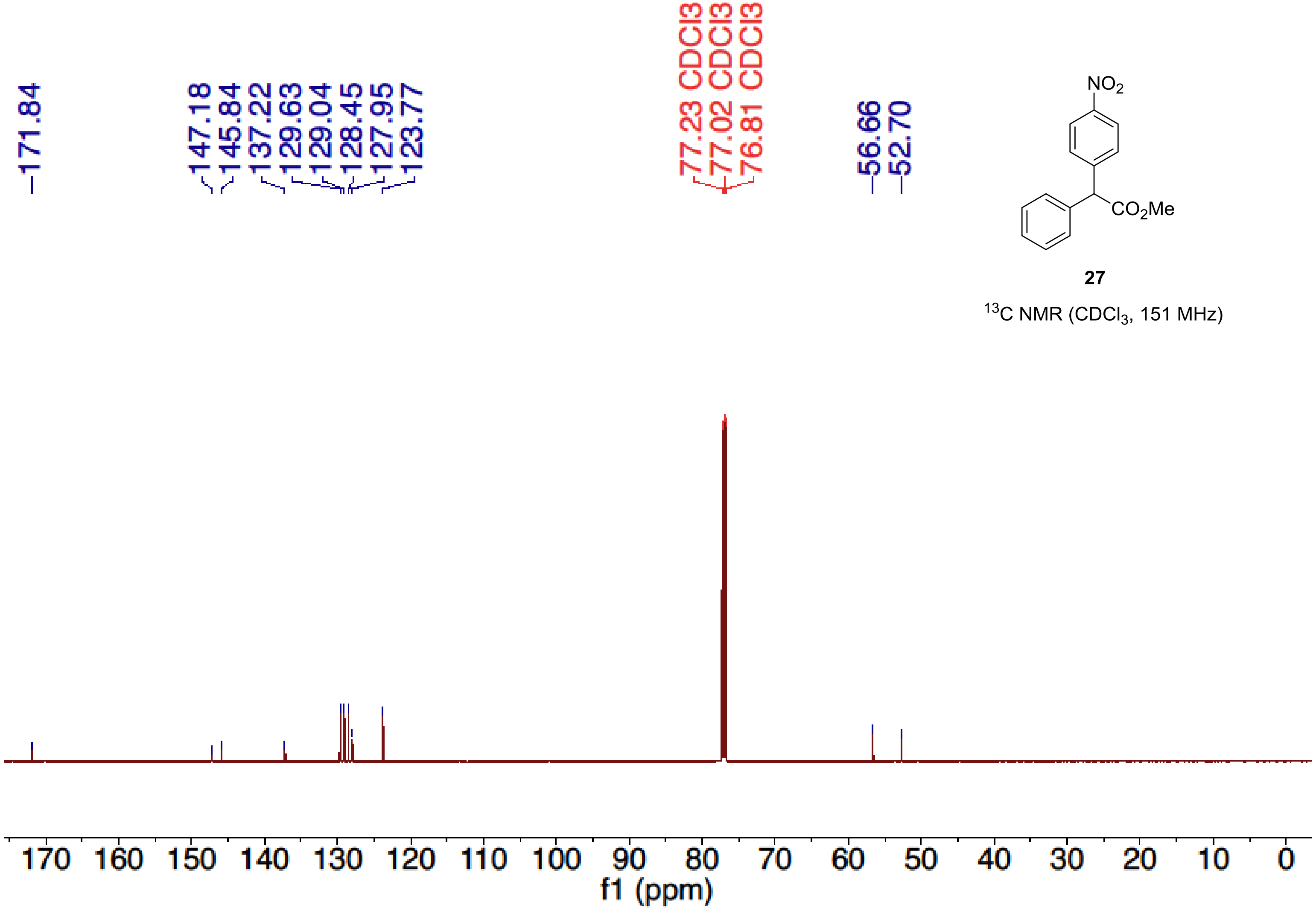
171.84
147.18
145.84
137.22
129.63
129.04
128.45
127.95
123.77
77.23 CDCl3
77.02 CDCl3
76.81 CDCl3
56.66
52.70
NO2
CO2Me
27
13C NMR (CDCl3, 151 MHz)
170 160 150 140 130 120 110 100 90 80 70 60 50 40 30 20 10 0
f1 (ppm)

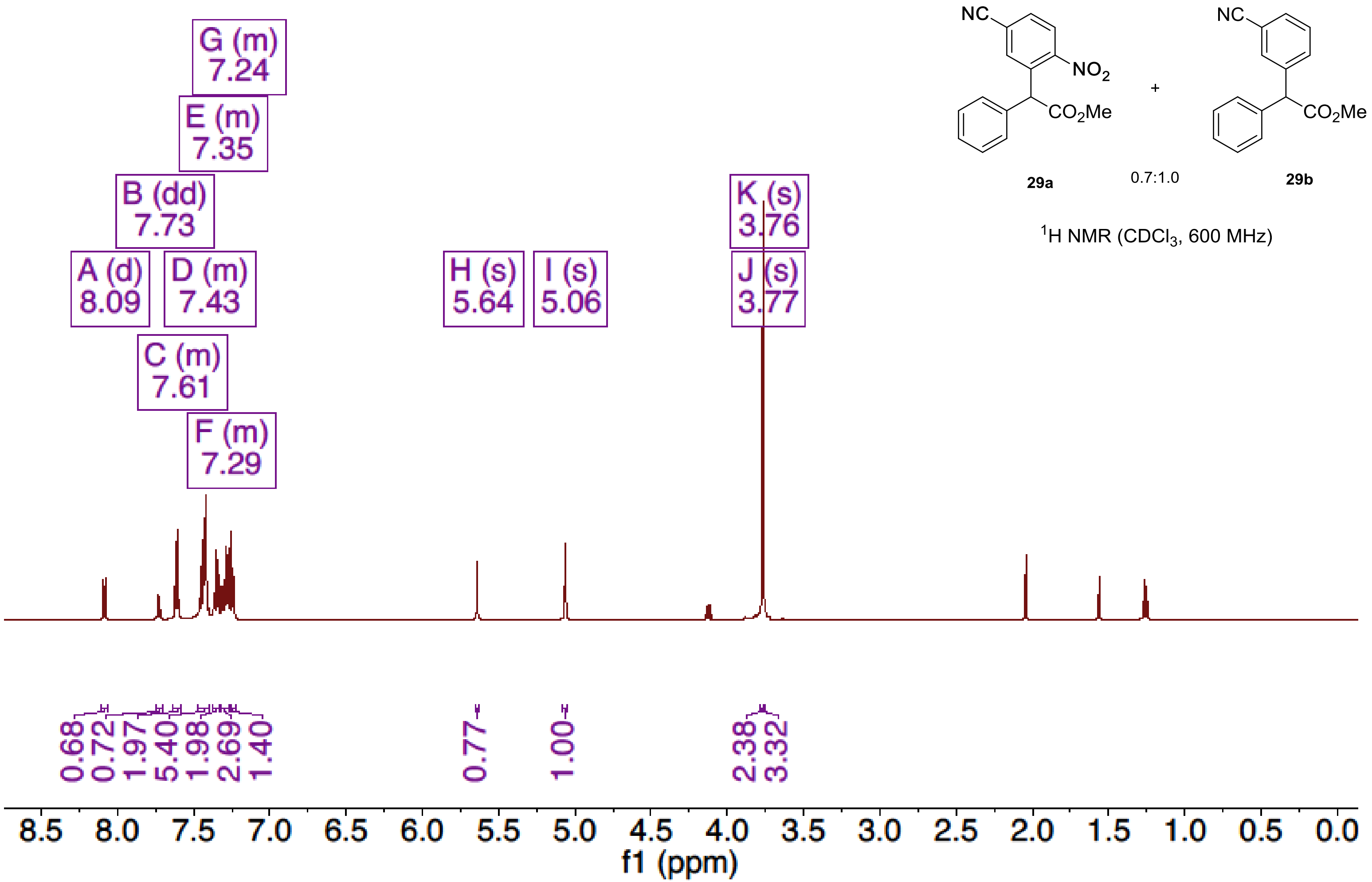

29a 0.7:1.0 **29b**

^{1}H NMR ($CDCl_3$, 600 MHz)

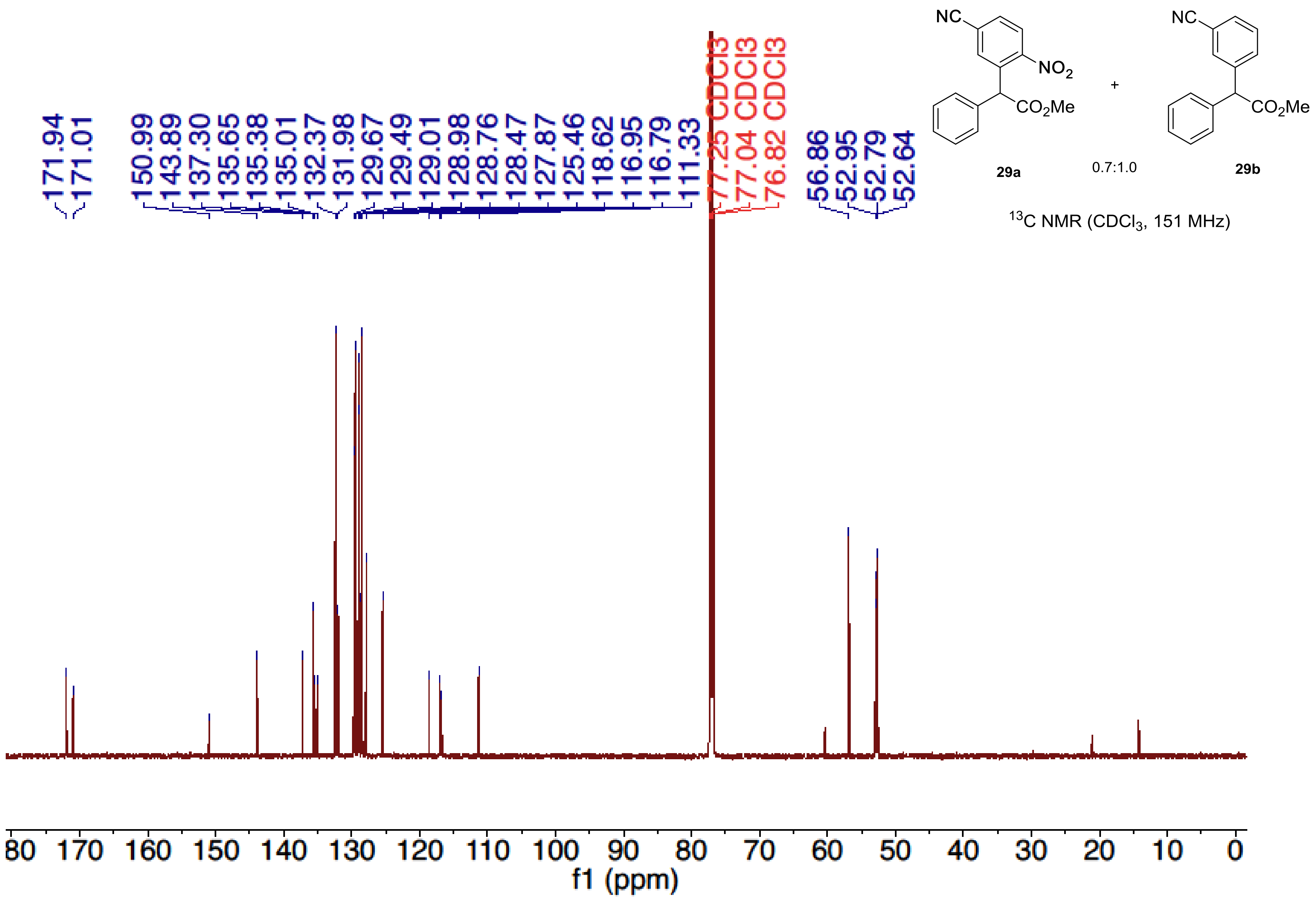

171.94
171.01
150.99
143.89
137.30
135.65
135.38
135.01
132.37
131.98
129.67
129.49
129.01
128.98
128.76
128.47
127.87
125.46
118.62
116.95
116.79
111.33
77.25 CDCl3
77.04 CDCl3
76.82 CDCl3
56.86
52.95
52.79
52.64
NC
NO2
CO2Me
+
NC
CO2Me
29a
0.7:1.0
29b
13C NMR (CDCl3, 151 MHz)
f1 (ppm)
